# Explorations of the Hydrogenation of D_2_ by Nitrogenase

**DOI:** 10.1002/cbic.70570

**Published:** 2026-10-28

**Authors:** Ian Dance

**Affiliations:** ^1^ School of Chemistry UNSW Sydney Sydney New South Wales Australia

**Keywords:** density functional calculations, HD reaction, hydrogen atom transfer, nitrogenase, reaction mechanisms

## Abstract

A non‐physiological substrate of the enzyme nitrogenase, D_2_, is doubly hydrogenated by the enzyme to produce 2HD. This ‘HD reaction’ is in competition with the native function of the enzyme, hydrogenating N_2_ to NH_3_. At the active site of the enzyme, a gallery of H atom donors is identified, and the proposed complete cycle mechanism for the N_2_ reaction uses these donors eight times to generate the 2NH_3_ + H_2_ products. Here, the functionality of these H atom donors in hydrogenating D_2_ to 2HD is investigated, applying density functional simulations to a 480+ atom model of the active site including all important surrounds. Many possible reaction trajectories were explored, and a revised mechanism for the HD reaction is presented. The mechanisms for hydrogenation of D_2_ and of N_2_ have common components, accounting for their experimentally known competition. Consideration of ten items of relevant experimental data about the HD reaction shows how the proposed mechanism complies with and interprets these data, although two experimental observations are still to be checked.

## Graphical abstract

What happens when D_2_ enters the nitrogenase gallery of H atom donors?

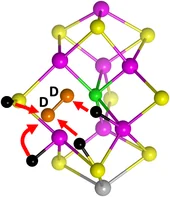

## 1 Introduction

Nitrogenase is the intriguing enzyme that converts unreactive atmospheric nitrogen to biologically usable ammonia. It activates and hydrogenates the remarkably unreactive N_2_ molecule, under mild conditions sought but not yet achieved by human enterprise. The enzyme is well understood structurally [1] and biochemically [1, 2, 3, 4, 5, 6, 7, 8, 9, 10, 11, 12], but isolation and characterisation of intermediates is difficult, and experimentally the chemical mechanism is elusive [13, 15]. The stoichiometry (Equation (1)) implies a mechanistic cycle with at least 27 chemical steps: eight introductions of a proton, eight additions of an electron, N_2_ binding, breaking the N─N bond, formation of six N─H bonds, two dissociations of NH_3_ and formation of one H─H bond. The sequence of intermediates is frequently described in terms of the number of added electrons, as En states, originating in the kinetic analyses of Thorneley and Lowe [16], and recently updated [17, 18]. The active site of Mo‐nitrogenase is an unprecedented cluster with the composition Fe_7_MoS_9_C(homocitrate), called FeMo‐co. This cluster is shown, together with relevant surrounds and ancillary components of the mechanism, in Figure 1.

**FIGURE 1:**
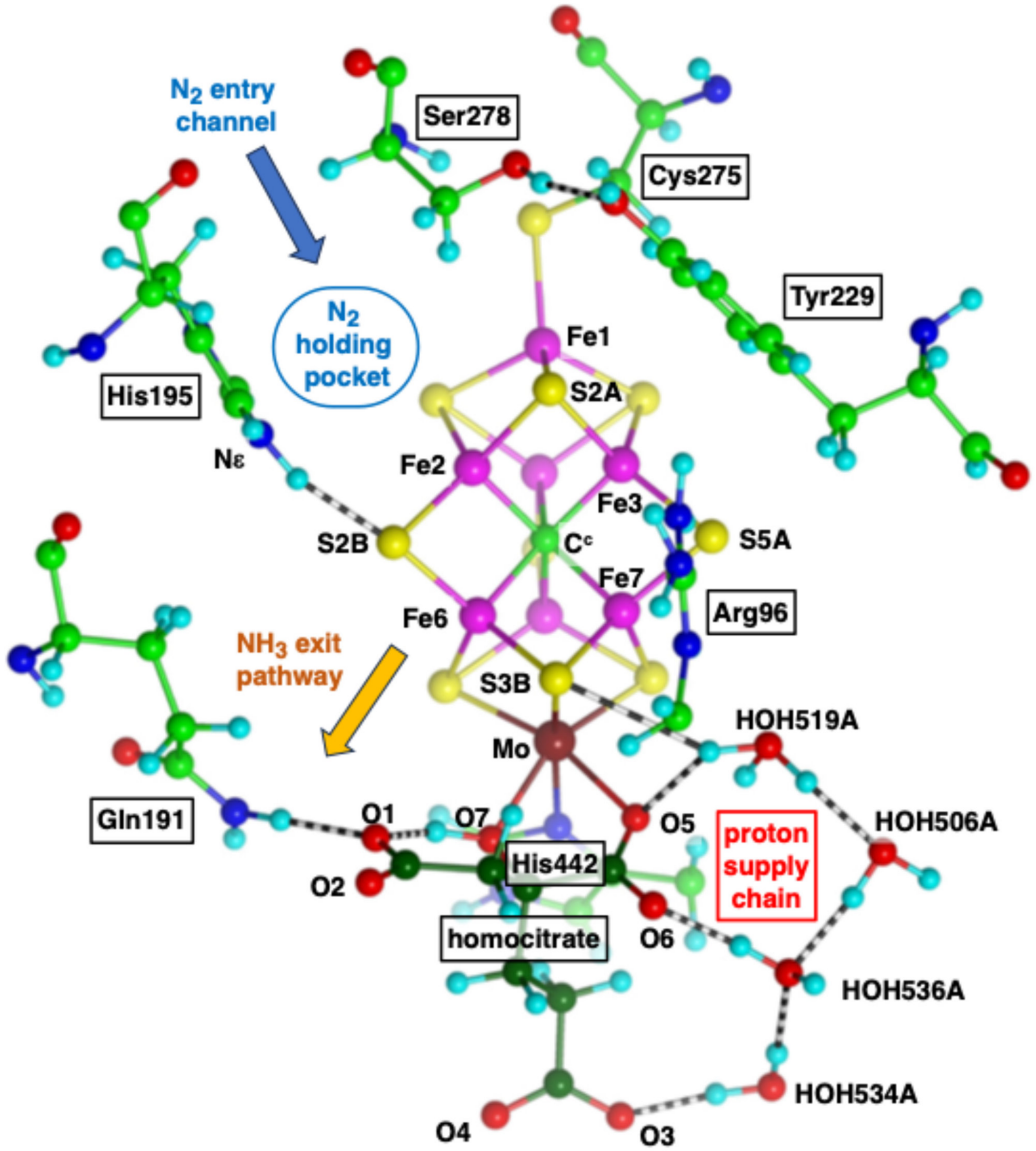
Structure of the active site of Mo‐nitrogenase, with FeMo‐co at the centre. Significant surrounding amino acids and water molecules are included, with labels for *Azotobacter vinelandii* protein, crystal PDB 3U7Q. Val70, omitted for clarity, covers the Fe2─S2B─Fe6 reaction zone, which is bounded on the right side by the side chain of Arg96. Homocitrate C atoms are dark green, and hydrogen bonds are striped. The N_2_ entry channel is between His195 and Ser278, with a proposed N_2_ holding pocket approaching Fe2. The four penultimate water molecules of the extended proton supply chain [19] are shown, with hydrogen bonds to O3, O6 and O5 of homocitrate and with the final proton directed towards S3B. The proposed direction of NH_3_ exit [20] from the reaction zone is indicated.

(1) $$N_{2}\text{ + }8H^{+}\text{ + }8e^{‐}\text{ → }2{\mathrm{NH}}_{3}\text{ + }H_{2}\;$$

### 1.1 The HD Reaction

In 1960, it was discovered that soybean root nodules containing nitrogenase generated HD when exposed to a D_2_/N_2_ atmosphere [21]. This is known as the ‘HD reaction’ of nitrogenase (Equation (2)). While not part of the natural repertoire of nitrogenase, the reaction is significant because its discovery spurred the accumulation of a substantial amount of experimental information about the reactivity of the enzyme [16, 21, 22, 23, 24, 25, 26, 27, 28, 29, 30, 31, 32]. The HD reaction requires N_2_ and competes with the N_2_ → NH_3_ conversion and therefore an understanding of the HD reaction contributes to an understanding of the mechanism of the physiological reaction.

(2) $$D_{2}\text{ + }2H^{+}\text{ + }2e^{‐}\text{ → }2\mathrm{HD}\;$$

### 1.2 Previous Mechanism Proposed for the HD Reaction

I recently proposed a mechanism for the HD reaction and described the intermediates and reaction steps with density functional simulations [33]. As shown in Scheme 1, the key step in this mechanism is an exergonic concerted double hydrogenation of D_2_ bound at the endo‐Fe6 position, forming two HD molecules coordinated at the endo‐Fe2 and exo‐Fe6 positions. Sequential dissociation of these HD completes the reaction. The exo/endo descriptors refer to coordination positions respectively trans and cis to the Fe─C^c^ bond. In Scheme 1, the N_2_ molecule shown at the exo position of Fe2 is in a region devoid of H atom donors [34], and therefore this N_2_ is not susceptible to hydrogenation: it is dubbed ‘non‐reducible’ N_2_ [35].

**SCHEME 1:**
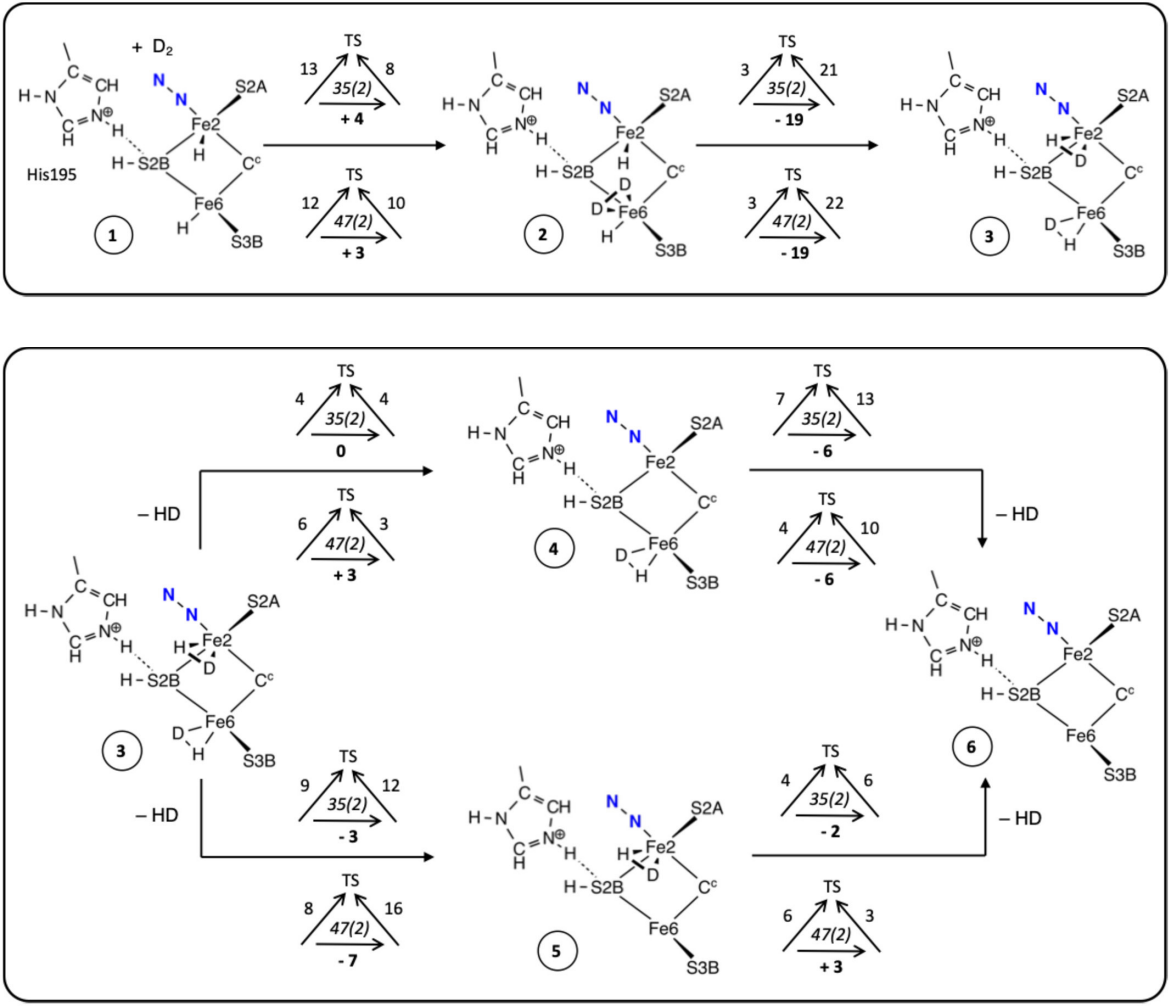
The mechanism previously proposed [33] for the HD reaction, showing the binding of D_2_ at the endo position of Fe6 (**2**) and the concerted double hydrogenation of D_2_ forming two bound HD (**3**). The two routes for sequential dissociation of the two Fe‐bound HD molecules are shown in the lower box. The sketches show only the essential components. Non‐reducible N_2_ at exo‐Fe2 [35] is included. The triangular symbols report electronic (S) states and calculated energies (kcal mol^−1^): the reaction energy is on the horizontal arrow, and barrier energies are oblique to the transition state (TS).

### 1.3 Mechanism Proposed for N_2_ to NH_3_

Concerted double hydrogenation of a substrate also occurs as the crucial step in my proposed mechanism for the conversion of N_2_ to 2NH_3_ [36, 37, 38, 39]. This double hydrogenation is the N_2_‐capture step illustrated in Figure 2. Here, N_2_, tumbling in the reaction space between Fe2 and Fe6, is hydrogenated at each end by the H atom on S2B and the H atom at the endo position of Fe7. This exergonic reaction step forms two H─N bonds and three N─Fe bonds, at the expense of one S─H and one H─Fe bond. In this way, notoriously unreactive N_2_ is doubly activated in one step, avoiding the need to first bind N_2_ and then form bound N_2_H [36]. This N_2_‐capture to form bound HNNH is followed by further hydrogenation steps to complete the formation of two NH_3_ molecules [37, 38, 39].

**FIGURE 2:**
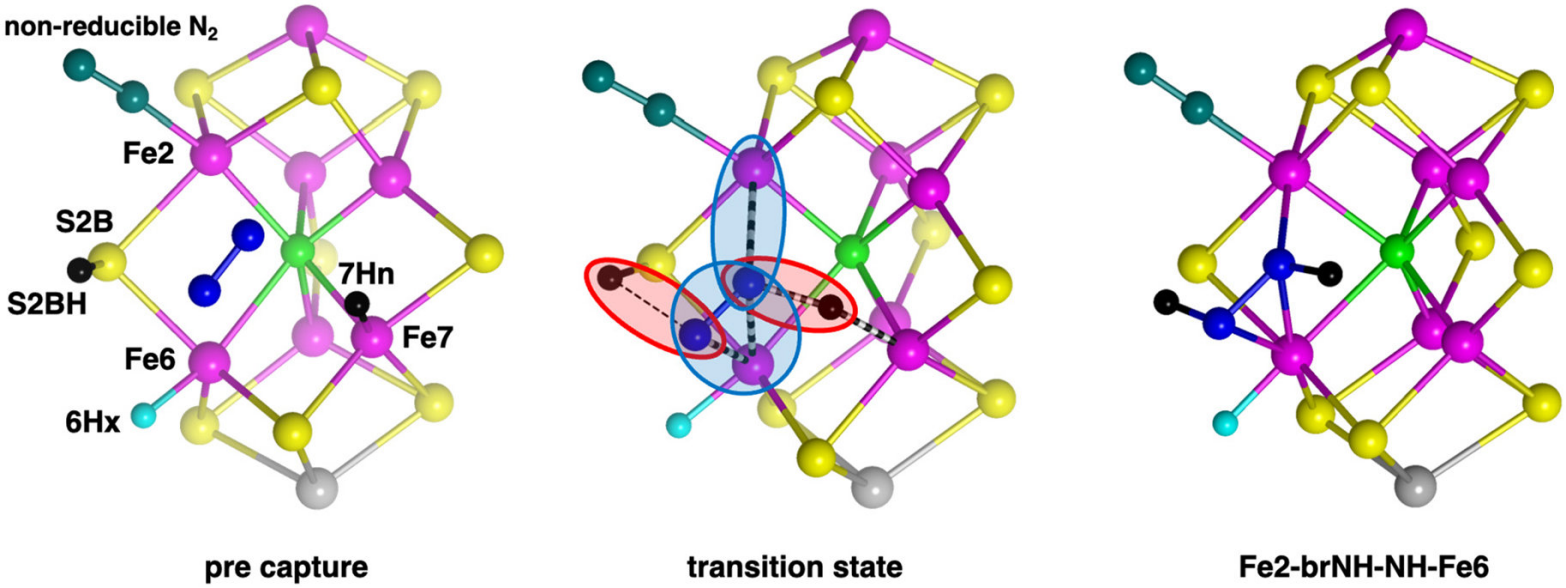
Detail of the N_2_ capture step: the two capturing H atoms are black. In **pre‐capture**, N_2_ is >3.2 Å from Fe2 and Fe6. At the **transition state**, red enclosures emphasise the two H─N bonds forming and blue enclosures emphasise the three N─Fe bonds forming.

## 2 Catalytic Reaction Domain

All of the hydrogenations proposed for the HD reaction and N_2_ conversion are effected by H atom donors that surround the catalytic reaction domain. The architecture of the nitrogenase active space provides an elaborate hydrogenation chamber. This chamber, viewed in Figure 3 along the Fe1─Mo direction, abuts the Fe2─S2B─Fe6─S3B─Fe7 sequence of FeMo‐co and is walled by the side chains of His195, Arg96 and Val70, three residues known experimentally to be influential [40, 41, 42, 43, 44, 45]. The H atom donors that surround this chamber are shown in black on Figure 4. The six H to N donations required for the proposed complete mechanism of the N_2_ → 2NH_3_ catalysis are identified on Figure 4. S2BH can form H─N bonds twice during the full mechanistic cycle, because Nε of His195 functions as a proton buffer, storing a proton from S2B and returning it when required [34, 39]. Details of this buffer mechanism are in Section S7.

**FIGURE 3:**
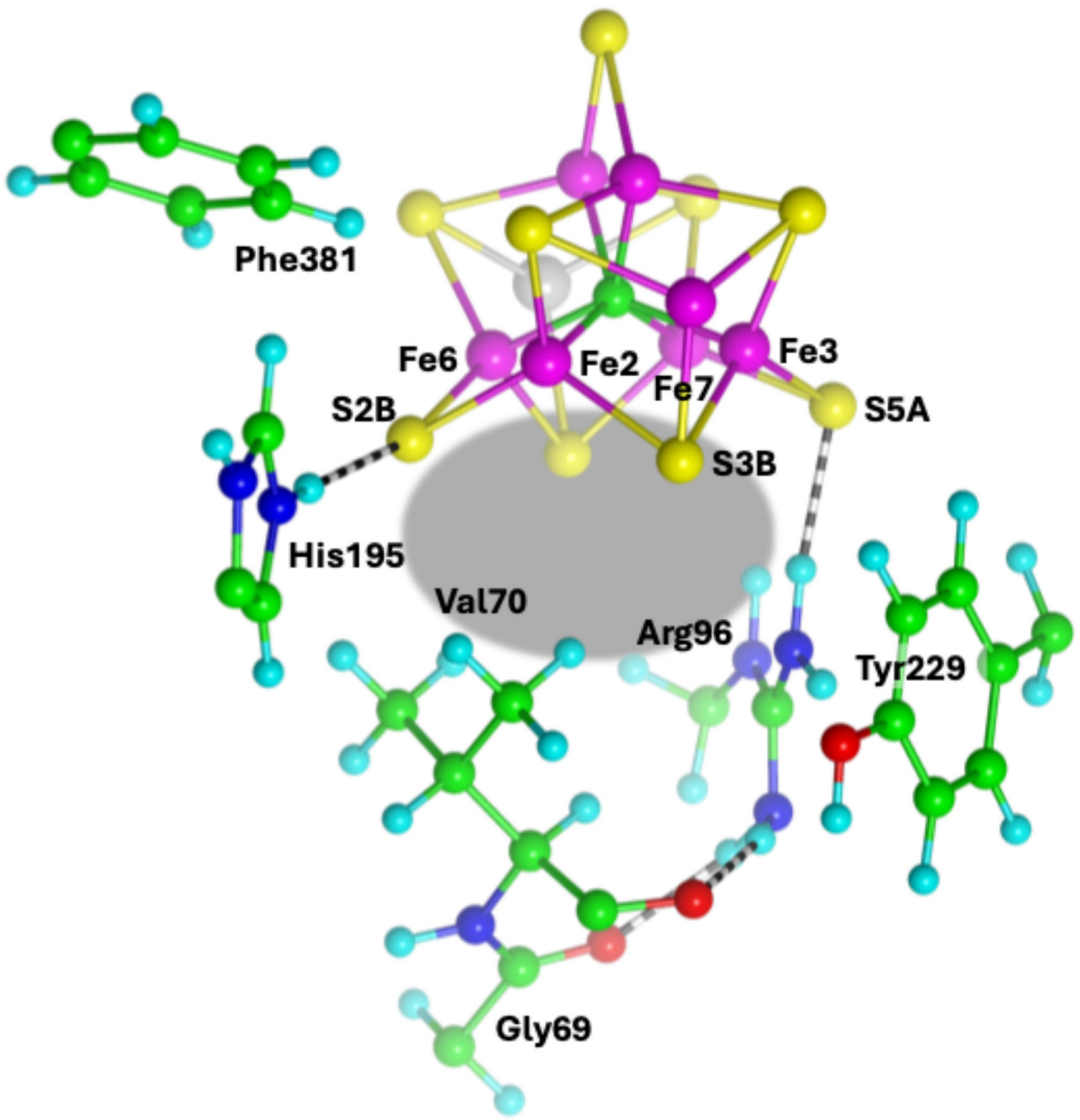
The catalytic reaction domain of Mo‐nitrogenase, with flanking sidechains. The view direction is close to the Fe1─C^c^─Mo axis of FeMo‐co.

**FIGURE 4:**
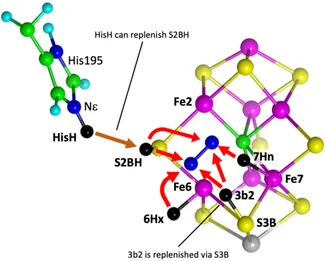
The H atoms, black, that form H─N bonds in the proposed complete mechanism for conversion of N_2_ (blue) to 2NH_3_ [39]. Red arrows show the hydrogenation steps, three at each of the N atoms. After the first donation from S2BH, it is replenished from the Nε site of His195. H at the 3b2 site on S3B is replenished from the proton wire that terminates at S3B [19]. Suffixes *x* and *n* denote exo and endo coordination positions.

The HD reaction and the N_2_‐capture reaction are both concerted double hydrogenations, and they compete for substrate, D_2_ or N_2_, at the same reaction space. The essential concept is illustrated in Scheme 2. Early stages in the mechanism accumulate H atoms on FeMo‐co, reaching the E4 intermediate in which H_2_ has been generated at the endo position of Fe6. Dissociation of this H_2_ creates a void in the reaction domain between Fe6 and Fe2. This allows N_2_ to enter this domain and be captured to form the Fe2─NH─NH─Fe6 intermediate, which subsequently proceeds through many steps to product NH_3_. The experimentally characterised H_2_/N_2_ exchange reaction is incorporated prior to the formation of Fe2─NH─NH─Fe6. Exogenous D_2_ can compete for the void position, binding reversibly at endo‐Fe6, prior to the double hydrogenation to 2HD. This is the contemporary mechanistic interpretation of the experimental information reported by Guth and Burris in 1983 [27] who also proposed a model in which D_2_ and N_2_ compete for the same form of the enzyme.

**SCHEME 2:**
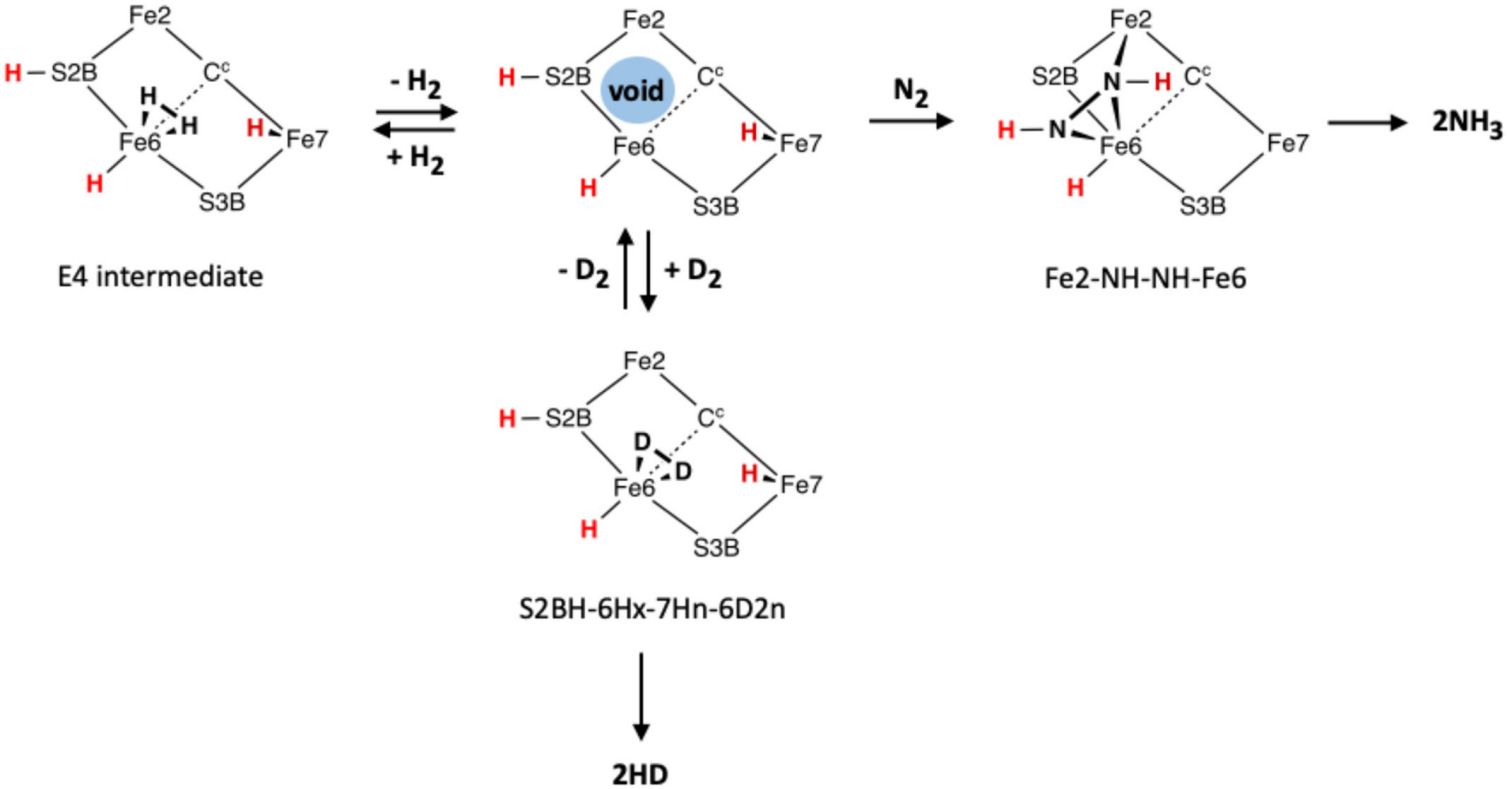
Fundamental mechanistic framework for the NH_3_ and HD reactions of nitrogenase.

The HD mechanism in Scheme 1 is hydrogenation of D_2_ by H donors at Fe2 and Fe6. But the donor H gallery presents alternatives. Could concerted hydrogenation of D_2_ occur with S2BH and 7Hn, analogous to the N_2_‐capture step (Figure 2)? In this paper, I explore these other possibilities for hydrogenation of D_2_ and for the mechanism of the HD reaction. A large collection of experimental data, itemised in ref. [33], constrains and challenges mechanisms proposed for the HD reaction. After reporting the new results and the mechanism that evolves from them, I describe its compliance with, and interpretation of, the experimental data.

## 3 Methodology

These questions are investigated through density functional simulations of reaction trajectories using my 480+ atom model of the active site and all relevant surrounds. Details of the DMol3 computational methodology and its validation [46, 47, 48, 49, 50, 51, 52, 53, 54, 55, 56] are provided in the Supporting Information. The electronic state of the FeMo‐co cluster was ‘235’, with input spin densities specified as negative on atoms Fe2, Fe3 and Fe5. The rationale for this as one of the most stable electronic states [56, 57, 58, 59] is provided in the Supporting Information. The optimised spin densities and total spin S for each of the intermediates and transition states are reported with the atomic coordinates in the Supporting information.

## 4 Results

All calculated models contain H, not D: zero point vibrational energies are not included. Atoms derived from D_2_ are labelled D in the Schemes to show their pathways and avoid confusion with the H atom donors (coloured red). At each Fe, the coordination positions are named exo when trans to C^c^ and endo when cis the Fe─C^c^ bond. Suffixes *‘x*’ and ‘*n*’ in the labels of intermediates denote exo and endo. H_2_ bound at endo‐Fe6 is labelled transverse or parallel relative to the enclosing S2B─Fe6─S3B angle (Figure 5). Labels for intermediates in the reaction schemes are constructed as an abbreviated list of the components, using symbol H in place of D. Because the labels are unwieldy in the text, the intermediates are also numbered and numbers are used in the text to simplify reference to the schemes.

**FIGURE 5:**
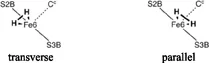
Conformations of H_2_ bound at endo‐Fe6.

### 4.1 Reaction Sequences

The sequences here start with H_2_ bound to FeMo‐co, at the E4 stage, generated from an H atom at endo‐Fe6 and an H atom on S3B in the 3b2 conformation [60]. This intermediate with H_2_ coordinated at the endo‐Fe6 position (6H_2_n) is the precursor to the established H_2_/N_2_ exchange, in which H_2_ dissociates to create the vacant position used in the N_2_ capture step (Figure 2, Scheme 2). This E4 intermediate occurs after the addition of 4 electrons and 5 protons. Although it is commonly assumed that the numbers of added protons and electrons at each stage are equal, there is no experimental support for this equality. The reason for accumulation of five protons at the E4 stage in my N_2_ → NH_3_ mechanism is the required sum of two H for dissociating H_2_ in the H_2_/N_2_ exchange step, two H atoms at S2B and Fe7n for the double hydrogenation of N_2_ in the capture step, plus 6Hx at the exo position of Fe6 [39]. This 6Hx is required to be in place at this early stage so that the subsequent steps—H_2_ formation, H_2_/N_2_ exchange, and then N_2_ capture—are forced to use the endo‐Fe6 position because exo‐Fe6 is blocked. In the following descriptions of hydrogenation of D_2_ all intermediates have the E4 5H^+^ status.

Referring to Scheme 3, the parallel conformation of 6H_2_n **7** rotates without barrier to the transverse conformation **8**. This rotation, and the weakly exergonic dissociation of 6H_2_n (**7d**) have very small barriers. Intermediate **8** can undergo exergonic concerted double hydrogenation using H atoms 6Hx and 7Hn to form **10**. The reaction energy and barrier are −14 and 8 kcal mol^−1^ respectively. The corresponding reaction without non‐reducible N_2_ at exo‐Fe2 (**8x** → **10x**) has a reaction energy −16 kcal mol^−1^ and barrier 10 kcal mol^−1^. There is an alternative route from **8** to **10**. The first step is an exergonic D atom exchange between the endo and exo positions of Fe6, forming **9**, followed by exergonic capture of 6Dn by 7Hn to generate 7DHn (Scheme 3). Also shown on Scheme 3 are dynamics for H_2_ dissociation from the endo‐Fe6 position (**8** → **8d**) and from the exo‐Fe6 position (**9** → **9d**). The additional entropy contribution for H_2_ dissociation into the protein environment is estimated to be −2 kcal mol^−1^ [33].

**SCHEME 3:**
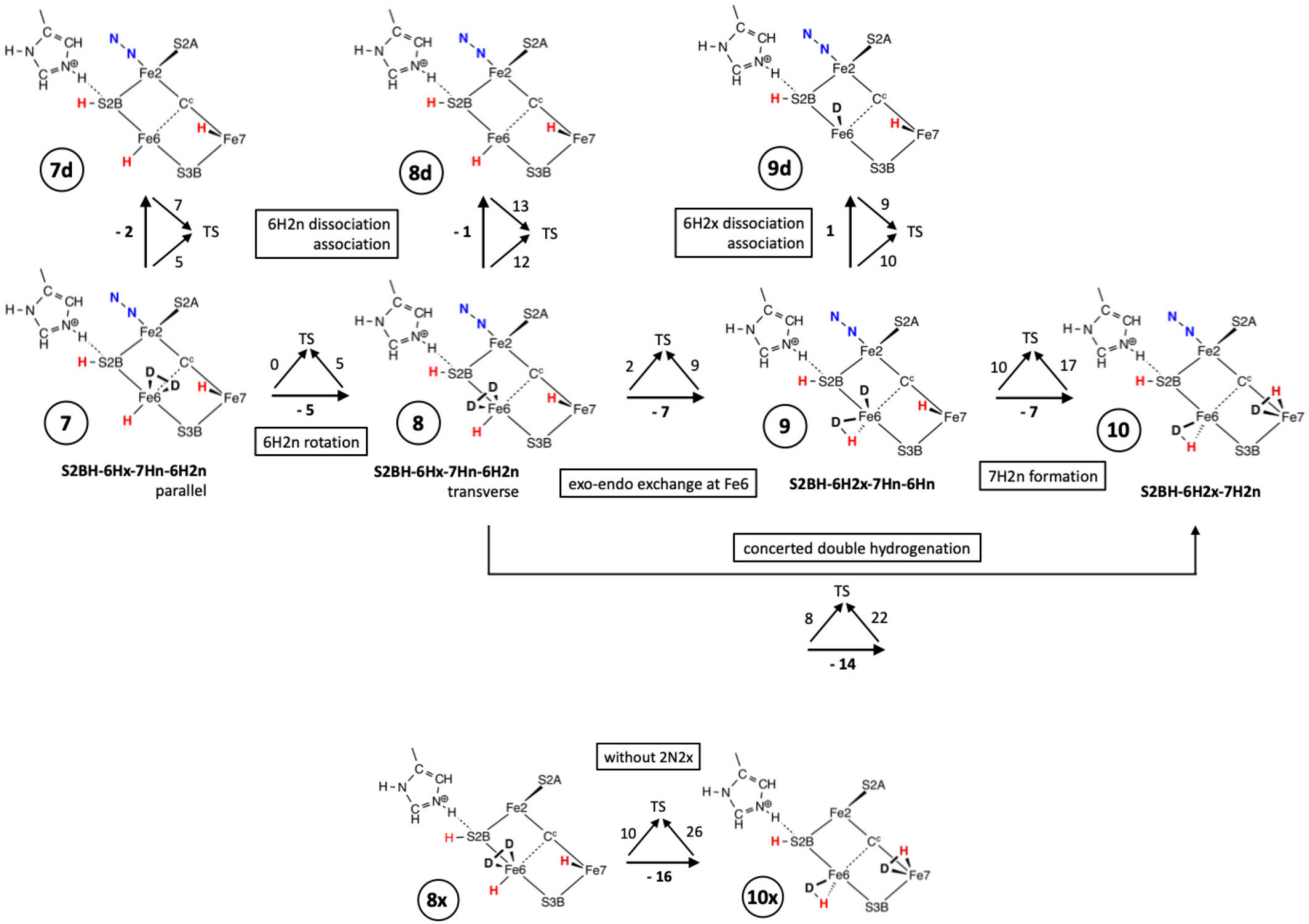
Reactions of **7**. H atom transfer steps are horizontal, and H_2_ dissociation steps are vertical. Triangles show the reaction potential energy with forward and reverse barriers (kcal mol^−1^).

Intermediate **10** contains two HD molecules, coordinated at exo‐Fe6 and endo‐Fe7. The dissociation dynamics for each of these are presented in Scheme 4. Both are exergonic and will benefit from the entropy contribution. Note that dissociation of 7H_2_n has a very small kinetic barrier of 2 kcal mol^−1^.

**SCHEME 4:**
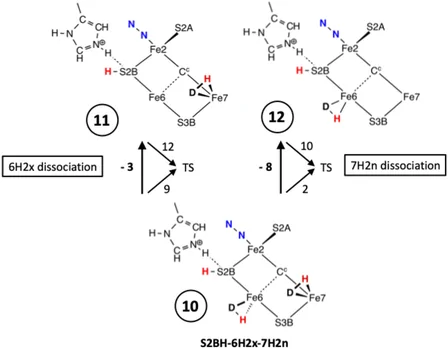
Energies (kcal mol^−1^) for dissociation of the two HD molecules in **10**.

The reactions in Scheme 3 do not involve the H donor on S2B. Two hydrogenations incorporating S2BH have been found and are shown in Scheme 5. Both are endergonic and have large kinetic barriers of 23 and 26 kcal mol^−1^. All explorations of H─H bond formation by S2BH yielded difficult trajectories, caused in part by the geometry of the bridging S2B‐H entity.

**SCHEME 5:**
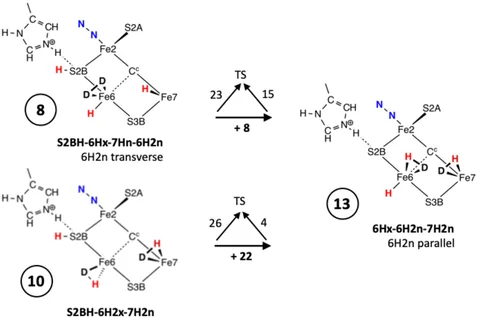
Hydrogenation steps involving S2BH. Reaction energies in kcal mol^−1^.

Of the three H atom donors surrounding D_2_, the 6Hx plus 7Hn pair are active in the Scheme 3 reactions, and the S2BH plus 7Hn pair are used in Scheme 5. The third combination is S2BH plus 6Hx, but the double hydrogenation of D_2_ at endo‐Fe6 using S2BH and 6Hx as H atom donors does not occur, because the interchange of H and H_2_ at the endo and exo positions of Fe6 is dominant (Scheme 6). This formation of **15** from **14** is analogous to **8** → **9** the exchange step shown in Scheme 3 and occurs with very similar energies.

**SCHEME 6:**
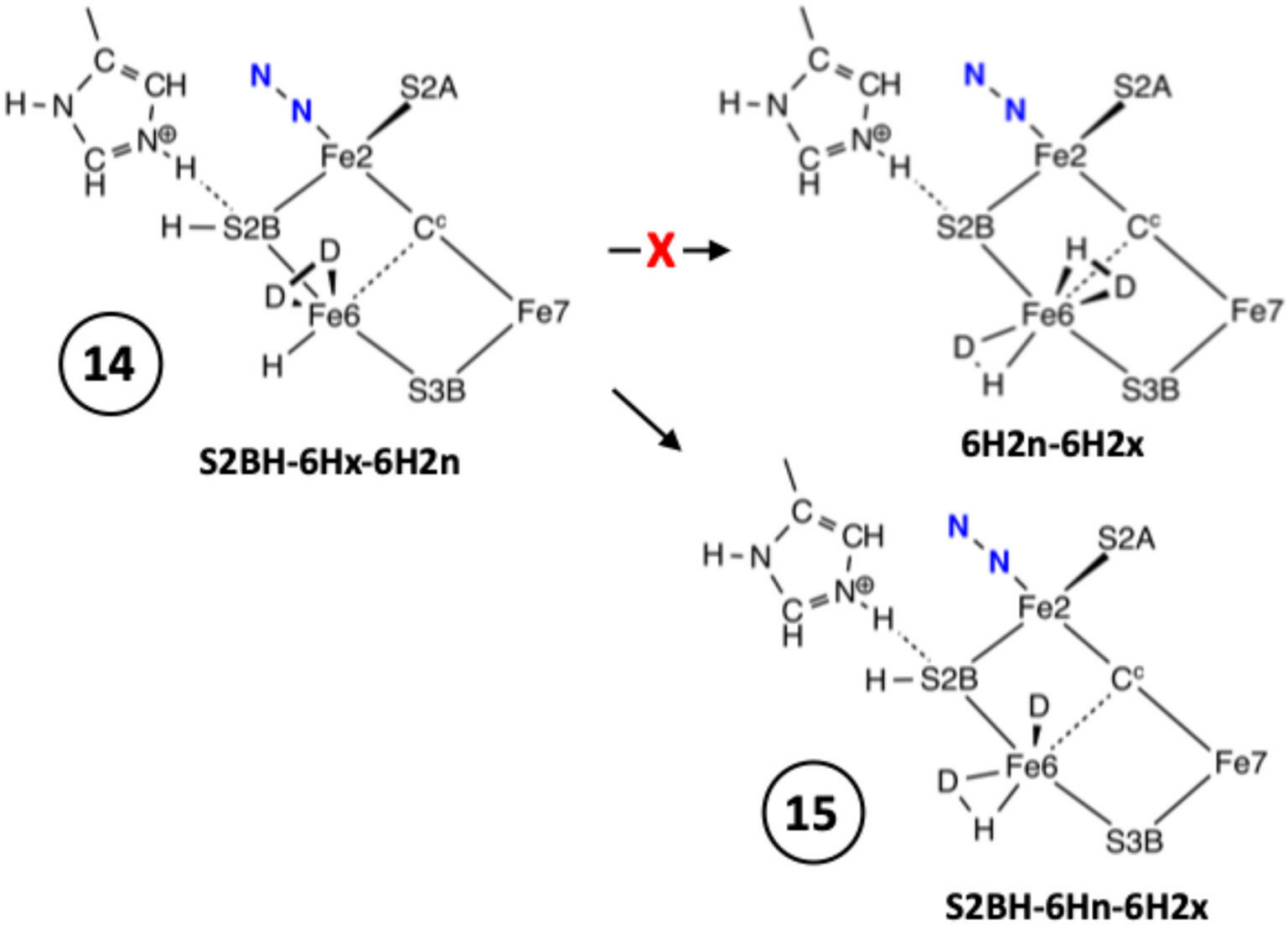
Double hydrogenation of D_2_ at endo‐Fe6 using S2BH and 6Hx as H atom donors does not occur.

### 4.2 Geometries

The foregoing schematic presentations of the hydrogenation reactions do not reveal the geometries of the reactants, and the characteristic plasticity of FeMo‐co that is involved. Figure 6 shows the essential geometries of intermediates and transition states for reactions in Scheme 3. The salient feature is variable elongation of the distance of Fe6 from the central C atom. In the double hydrogenation of D_2_, **8** to **10**, this distance changes from 2.40 to 2.52 Å at the transition state, to 2.83 Å in the product. These distances correlate with the extent and stereochemistry of the Fe6 ligation. Figure 7 shows the essential features of the hydrogenation reactions using S2BH, and Figure 8 shows the changes that occur during dissociation/association of H_2_. There is a very large change in the C^c^─Fe6 distance from 2.88 to 1.96 Å on dissociation of H_2_ from exo‐Fe6, but minor change when H_2_ dissociated from endo‐Fe7. The dissociation of H_2_ from endo‐Fe6 is associated with decrease in the enclosing endo angle from 161° to 141°. In all of these reactions, the geometry changes at Fe6 maintain it’s symmetrical coordination stereochemistry as the coordination number varies between 4, 5 and 6. As previously described [36], there is an adaptation energy associated with these modifications of the FeMo‐co geometry, and the total energy calculated is the sum of the adaptation energy and the bond breaking/making energies. These different components of the total energy can often be discerned as such when following the details of reaction trajectories in my computational procedure.

**FIGURE 6:**
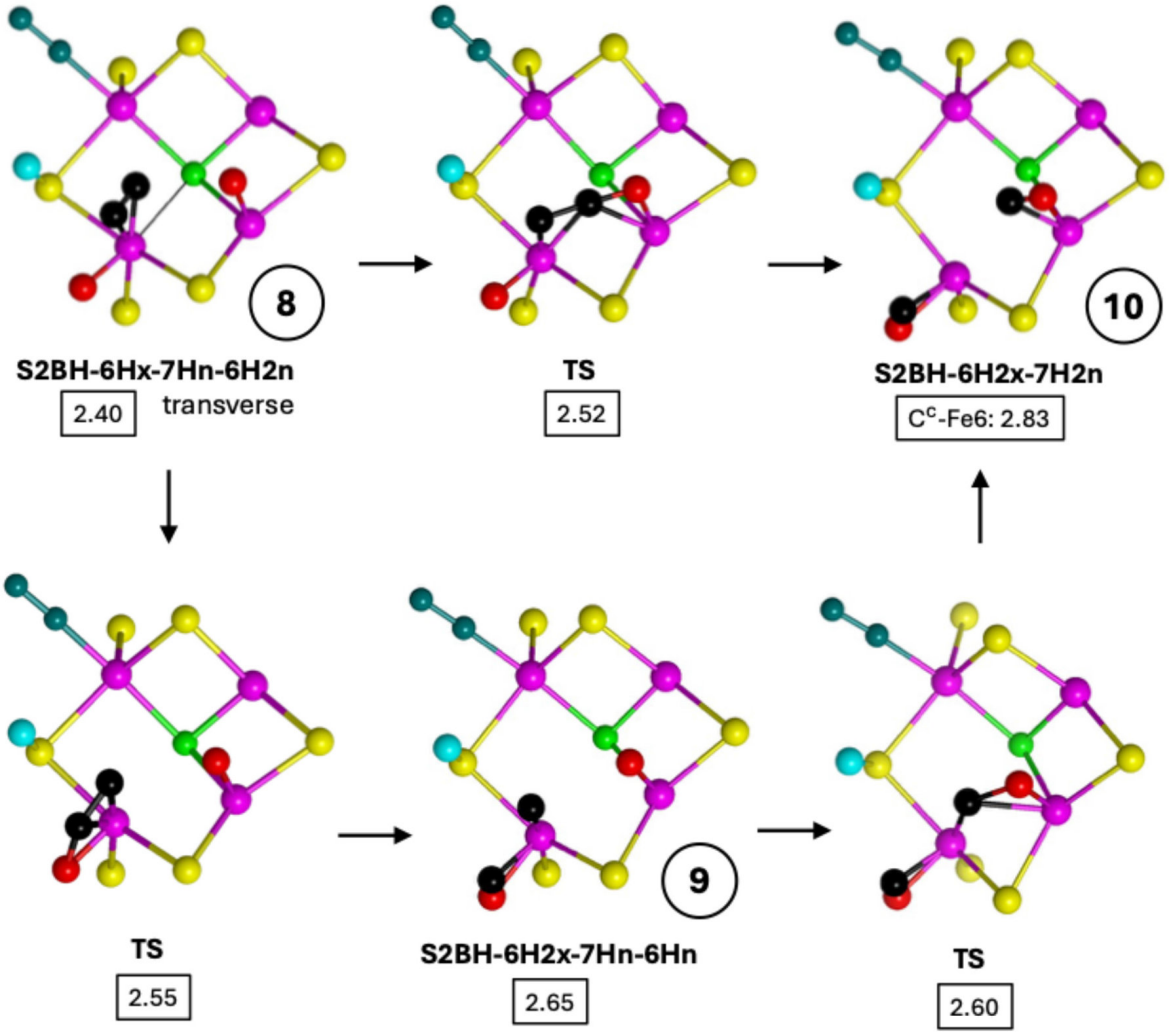
Core geometries for reactions in Scheme 3. H atoms (enlarged for emphasis) are coloured red for active H donor, black for D, cyan for inactive H donor. The varying C^c^─Fe6 distances (Å) are boxed. View directions are varied to maintain clarity.

**FIGURE 7:**
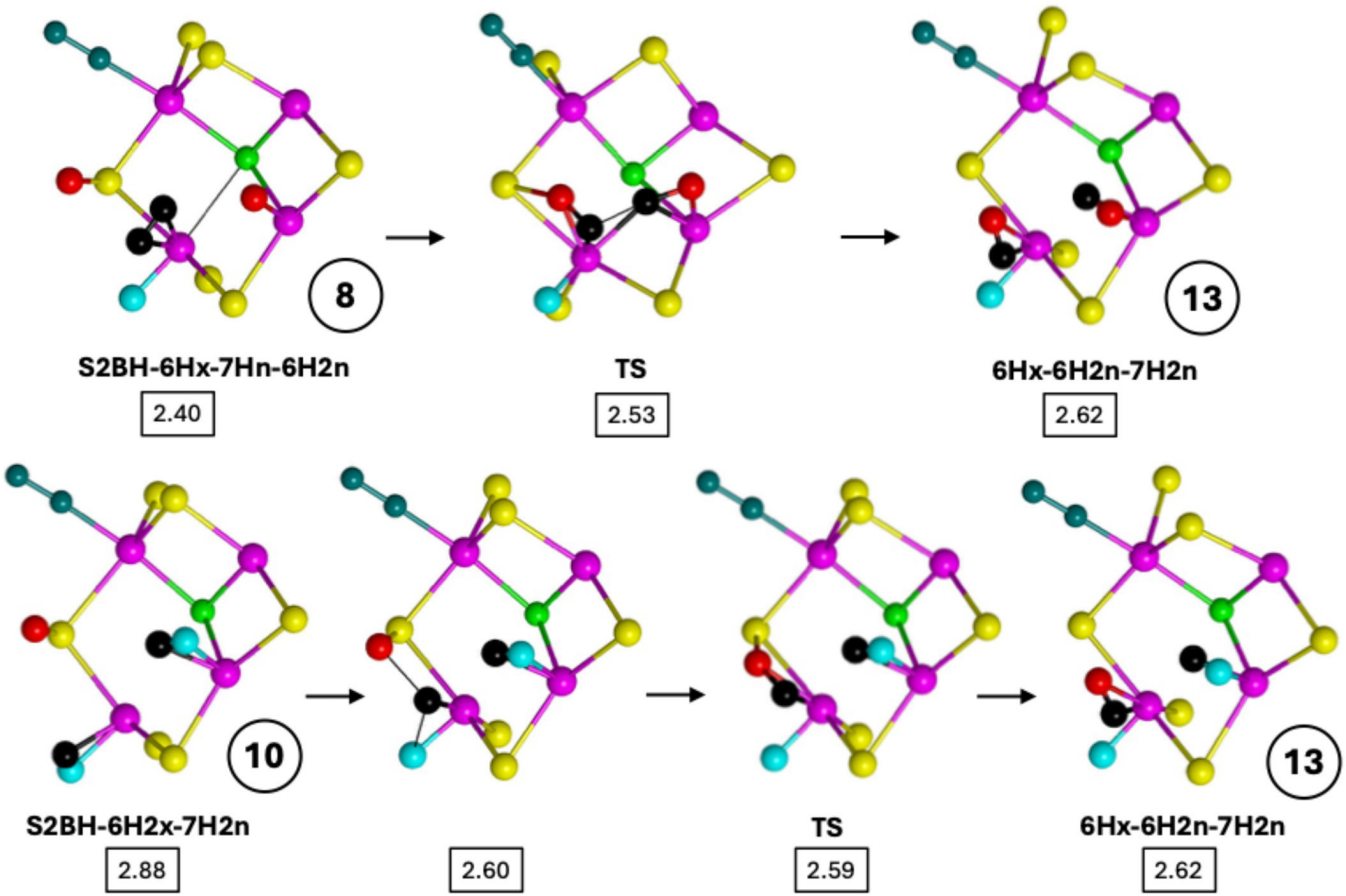
Core geometries for hydrogenations involving S2BH. H atoms (enlarged for emphasis) are coloured red for active H donor, black for D, cyan for inactive H donor. The varying C^c^─Fe6 distances (Å) are boxed. View directions are varied to maintain clarity.

**FIGURE 8:**
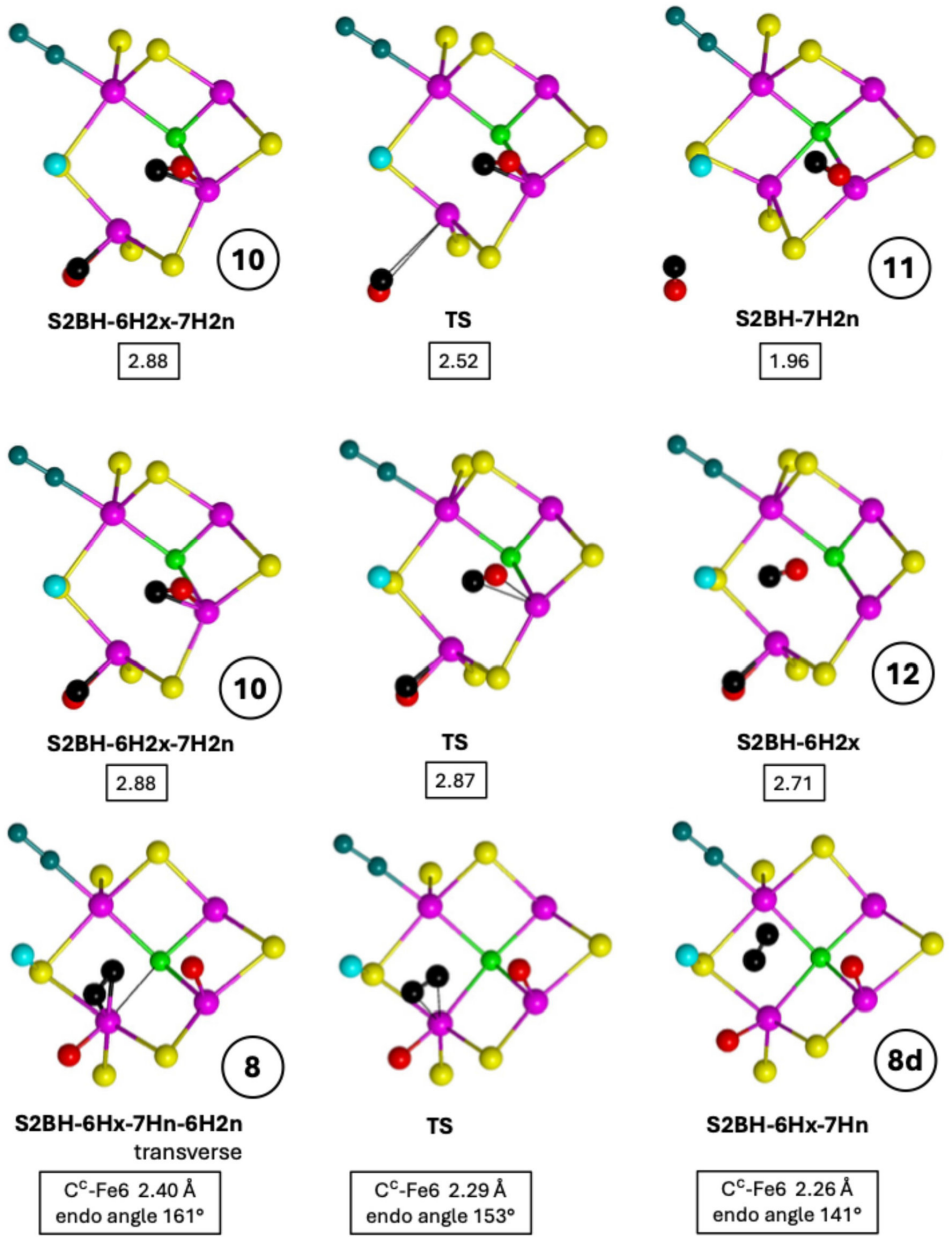
Geometries for dissociation of H_2_ from the exo‐Fe6, endo‐Fe7 and endo‐Fe6 positions. Thin lines represent elongated Fe–H distances at the transition states. Endo angle is S2B─Fe6─S3B, enclosing the moving H_2_.

The many calculated reaction trajectories testing the multiple variables in this reaction space reveal several reactivity principles. One is that the S2B─H bond is energetically difficult to break when forming a new H─H bond. For example, in the transition state for the **8**–**13** reaction (Figure 7), the 2B─H bond is stretched to 1.61 Å and the H─H distance is shortened to 1.06 Å, close to the product geometry. A second principle is that 7Hn is effective in stealing an H atom from nearby H_2_. A third principle is that generation of H_2_ bound at the exo position of Fe6 is strongly stabilising. 6H_2_x is the principal contributor to the large energy advantage of **10** over **13**.

### 4.3 Exo‐Fe6‐N_2_

The previously proposed mechanisms for the HD reaction and for the N_2_ → NH_3_ catalysis involved H occupying the exo position of Fe6, and in both mechanisms this 6Hx functions as an H atom transfer agent. The possibility that the exo‐Fe6 position is occupied instead by non‐reducible but possibly influential N_2_ is now considered. The questions investigated are the access of N_2_ to exo‐Fe6, its binding dynamics and mutual influences of 2N_2_x and 6N_2_x.

It is known that CO can bind at exo‐Fe6 [61, 62], and since the diffusion of CO and of N_2_ through the protein are expected to be very similar [63], this can be taken as experimental indication that N_2_ can access and bind at exo‐Fe6. Exo‐Fe6‐N_2_ is away from the hydrogenation chamber and is unlikely to be transformed to NH_3_, but it could influence H_2_ reactivity within the chamber. In addition, the binding dynamics of N_2_ at exo‐Fe2 and exo‐Fe6 are expected to be connected through the coordinative allosterism effected by C^c^ [64].

Using the full computational model, I have tracked the diffusion of a second N_2_ from the N_2_ entry channel [35, 65], past non‐reducible 2N2x, into the reaction space, and then further towards the exo coordination position of Fe6. Three positions for this N_2_ along the diffusion pathway are shown on Figure 9, coloured light grey, to grey, to black. Energy variations along this pathway are less than 5 kcal mol^−1^. At the middle grey position N_2_ squeezes between S3B and a methyl group of Val70: these contacts are marked as thin lines on Figure 9. This tightness for N_2_ diffusion is consistent with the results of George et al. who found that substituting Val70 with larger isoleucine inhibits and slows, but does not substantially change, the binding of CO to FeMo‐co [62]. Figure 9 also shows how hydrogen bonding between the carbonyls of residues 69 and 70 and guanidine NH_2_ of Arg96 maintain a canopy over the reaction space.

**FIGURE 9:**
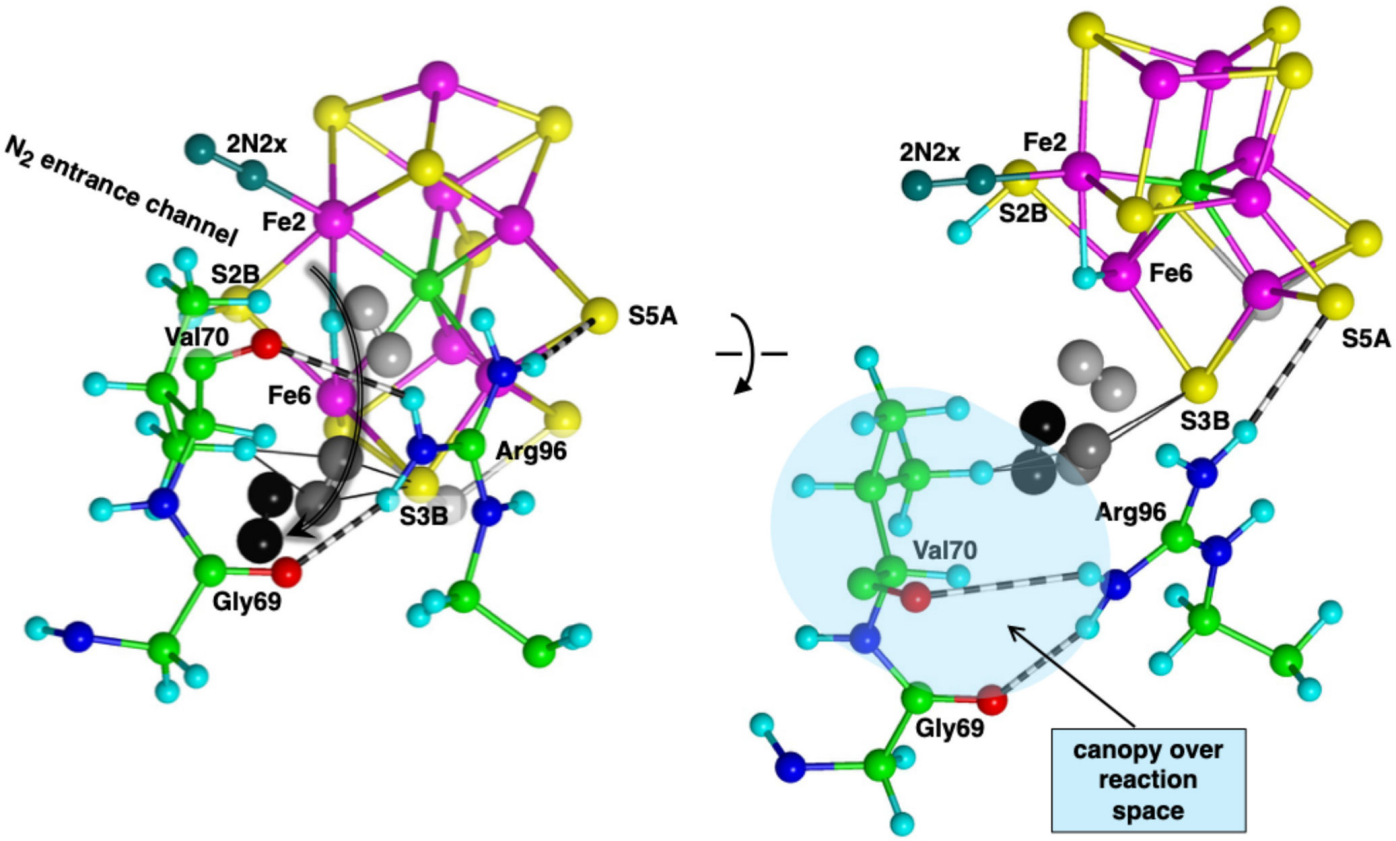
Aspects of the diffusion (curved arrow) of N_2_ (light grey, to grey, to black) through the reaction space towards the exo‐Fe6 position. The computational model contains S2BH and H bridging Fe2 and Fe6 (to prevent N_2_‐capture steps or N_2_ binding at endo‐Fe2 or endo‐Fe6). Separate 2N2x (turquoise) indicates the general vicinity of the N_2_ entrance channel. The thin lines at the grey position identify the closest contacts of N_2_ with S3B and the side chain of Val70. Hydrogen bonds are black‐white striped. The two C ═ O···H_2_N hydrogen bonds connecting Arg96 to Gly69 and Val70 are the canopy (pale blue) over the reaction space containing the diffusing N_2_.

Scheme 7 presents two sets of data comparing the binding dynamics of N_2_ at the exo positions of Fe2 and Fe6. Exo‐Fe2─N_2_ and exo‐Fe6─N_2_ are both stable in the test structures **16** and **17**. Dissociation of Fe6‐N_2_ is marked in red and dissociation of Fe2─N_2_ is blue on Scheme 7. The binding of N_2_ at exo‐Fe6 is weaker than the binding at exo‐Fe2. The entropy contribution for dissociation of coordinated N_2_ into an intra‐protein environment is estimated to be −4 kcal mol^−1^ [35], essentially cancelling the potential energies for dissociation of N_2_ from Fe6 in the two cases shown in Scheme 7. As is frequently found the C^c^─Fe distance varies when N_2_ binds in the trans exo position. For **17** → **17d** in Scheme 7, the C^c^─Fe6 distance changes from 2.38 Å (associated) to 2.07 Å (transition state) to 1.88 Å (dissociated). With H bridging Fe2 and Fe6, **16**, extension of C^c^─Fe6 is restricted: 2.25 Å (associated) to 2.08 Å (transition state) to 1.94 Å (dissociated, **16d**).

**SCHEME 7:**
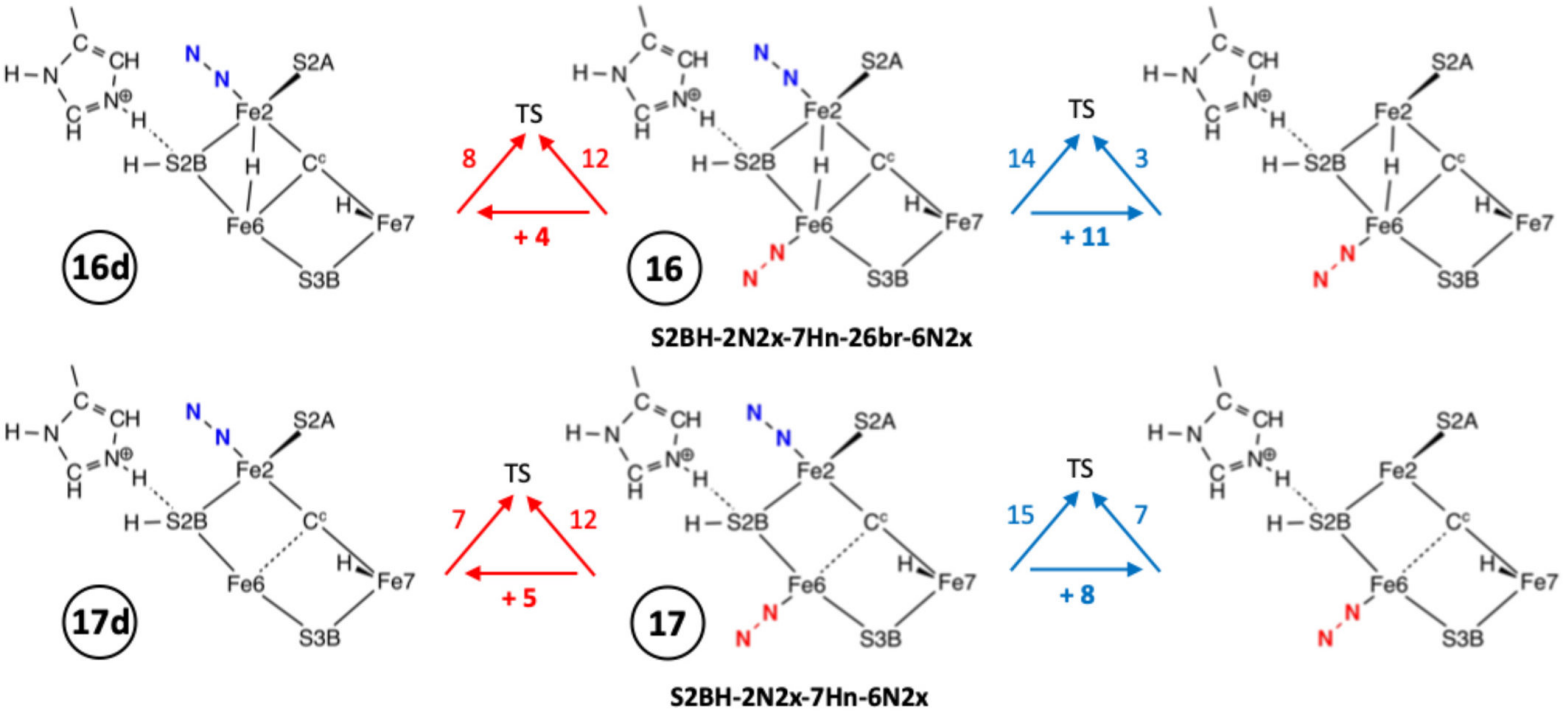
Potential energies and barriers (kcal mol^−1^) for the binding of N_2_ at the exo positions of Fe2 (blue) and Fe6 (red).

What is the effect of 6N_2_x on hydrogenation of H_2_ at endo‐Fe6? Results, shown in Scheme 8, reveal that the reactivity is different from the systems with H at exo‐Fe6. Binding of H_2_ at the endo‐Fe6 position is now more endergonic (+7 kcal mol^−1^) and when bound (**19**) undergoes barrierless H atom exchange with 7Hn forming 7H_2_n (**20**) which is 16 kcal mol^−1^ more stable. Dissociation of this 7H_2_n is exergonic, −10 kcal mol^−1^. H transfer from S2BH (to **21**) is again unfavourable (compare Scheme 5). Overall, with 6N_2_x present, the precursor for H_2_ involvement, **19d**, should it bind H_2_ at endo‐Fe6, would be transported to the deeper energy well of **20**. This system is not able to effect the D_2_ → 2HD conversion using H donors S2BH and 7Hn. The substantial influence of 6N_2_x in deactivating the endo‐Fe6 position (relative to 6Hx) is interpreted in terms of its larger contribution to the coordinative capacity of Fe6.

**SCHEME 8:**
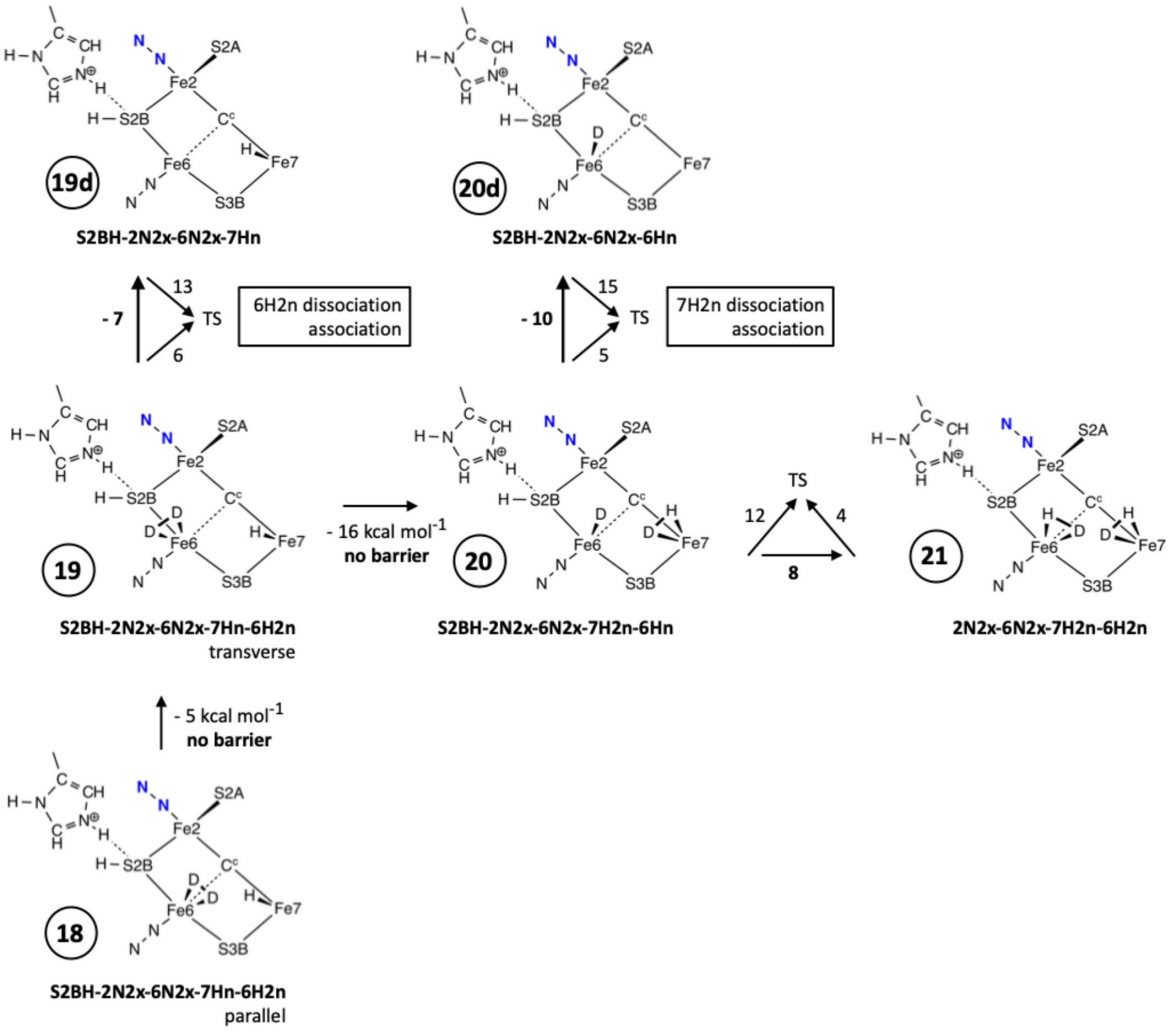
Hydrogenation reactions with N_2_ at the exo‐Fe6 position.

## 5 Discussion

Here, I have explored how the enzyme can hydrogenate D_2_ using H atom donors surrounding the reaction cavity, analogous to the physiological hydrogenation of N_2_ and its subsequent intermediates in the same space [36, 37, 38, 39]. Collectively, these results have confirmed reaction and reactivity characteristics previously described [66, 67], including the plasticity of FeMo‐co (without disruption [68]) evident in variations of one Fe─C^c^ distance, variable endo angles at Fe and coordinative allosterism [64, 69]. Fe coordination maintains spherical symmetry. One result about electronic structure, not reported explicitly (but evident in the Supporting Information) is that changes in the spin densities on Fe atoms associated with H or H_2_ coordination are minor relative to the larger changes that occur with N‐containing ligands.

The results reveal a feasible new mechanism for concerted double hydrogenation of D_2_ to 2HD. The reaction step is **8** → **10**, exergonic by 14 kcal mol^−1^ with a kinetic barrier of 8 kcal mol^−1^ (Scheme 3). Exergonic dissociations 6H_2_x and 7H_2_n (Scheme 4) complete the reaction.

### 5.1 Testing Against Experiment

Here, I consider the ten items of relevant experimental data about the HD reaction, showing how the proposed mechanism complies with and interprets these data. Items 8 and 9 retain some uncertainties.

1. **The HD reaction requires N_2_**. N_2_‐independent HD formation is <1% [26, 27]. While it might appear that 2N_2_x could be the mechanism enabler, the **8x** → **10x** dynamics (Scheme 3) show that with 2N_2_x absent, concerted double hydrogenation of D_2_ at endo‐Fe6 proceeds with essentially unchanged dynamics. The results in Scheme 8 show that N_2_ bound at the exo‐Fe6 position does not promote double hydrogenation of H_2_. Instead, I propose that 2N_2_x is actually the HD reaction enabler, by blocking the exo‐Fe2 position as binding site for D_2_. Double hydrogenation of D_2_ at the exo‐Fe2 position is very unlikely because there are no H donors available. Therefore, the favourable binding of N_2_ at exo‐Fe2 forces D_2_ into the reaction space (Figures 3 and 4), where, as demonstrated here, it can be readily hydrogenated to 2HD. This proposed role of 2N_2_x in forcing substrates into the hydrogenating zone operates also with N_2_ as substrate and would be relevant with acetylene and other non‐physiological substrates.
2. **The HD reaction is inhibited by larger pN** **_2_** [27, 70]. This occurs because reducible N_2_ and D_2_ are competing for the same reaction space.
3. **The K** **_m_** **(N** **_2_** **) for HD formation and the K** **_m_** **(N** **_2_** **) for NH** **_3_** **production are different** [27]. The two different processes are postulated to involve different N_2_ binding functions. The N_2_ that enables the HD reaction binds at exo‐Fe2 and is non‐reducible, while reducible N_2_ that proceeds to NH_3_ is captured in the reaction space between Fe2 and Fe6.
4. **The HD reaction and N** **_2_** **conversion to NH** **_3_** **compete for the same electrons** [27]. The contemporary interpretation is that both reactions are competing for the same H atom donors around the reaction domain.
5. **The HD reaction stoichiometry is D_2_ + 2H^+^ + 2e^–^ → 2HD**. This is embodied in the proposed mechanism.
6. **HD formation is inhibited by CO** [22, 23, 25, 27], and the D_2_ → HD and N_2_ → NH_3_ pathways are inhibited equally by CO [27]. This is readily understood mechanistically because CO easily forms an Fe2─CO─Fe6 bridge [61, 62], blocking the endo‐Fe6 and endo‐Fe2 positions.
7. **There is negligible formation of TOH when T** **_2_** **is used instead of D** **_2_** [25]. The non‐leakage of D to solvent during the HD reaction follows from the anhydrous and hydrophobic environment of the reaction domain, and the irreversibility of proton introduction along the proton wire [19].
8. **Different reactivities for the 195** **^Gln^** **and 195** **^Asn^** **mutated proteins**. The protein modified as 195^His^ → 195^Gln^ [29] converts D_2_ to HD and binds N_2_ (evinced by N_2_ inhibition of H_2_ evolution and C_2_H_2_ reduction) but has very low conversion of N_2_ to NH_3_ [30, 31]. The 195^Asn^ mutant does not convert D_2_ to HD or convert N_2_ to NH_3_ but does bind N_2_ [30]. The present results provide no insight into why 195^Gln^ supports the HD reaction but 195^Asn^ does not. The 195^Gln^ side chain can form a distorted hydrogen bond from NH_2_ to S2B [29], but the 195^Asn^ side chain cannot hydrogen bond to S2B.
9. **Formation of C** **_2_** **H** **_3_** **D in the presence of C** **_2_** **H** **_2_**. Nitrogenase turning over with C_2_H_2_ + N_2_ + D_2_ forms C_2_H_3_D with minor amounts of C_2_H_2_D_2_ [32]. Several mechanisms for this can be postulated. C_2_H_2_ binding at the vacant positions on Fe6 in intermediates **11** or **12** would be exposed to contiguous HD that could easily add to C_2_H_2_ in one or two steps. C_2_H_2_ bound at exo‐Fe6 in **9d** could add D, then H.
10. **Nitrogenase turning over with N** **_2_** **and HD does not form D** **_2_** [16, 28]. A cyclic reaction sequence that has the net effect of converting 2DH to H_2_ + D_2_ is shown in Scheme 9. The cycle, anti‐clockwise, involves the dissociated forms of intermediates **8** and **9** from Scheme 3, and depends on two occurrences of endo‐exo exchange steps at Fe6 (yellow in Scheme 9). Three aspects of this cycle need to be recognised. First, at the two stages where HD binds, it can bind in two orientations (enclosed in Scheme 9) relative to the contiguous single atom, but only one of these orientations can generate H_2_ (**22a** → **23**) or D_2_ (**9a** → **8**) in the following endo–exo exchange step. Assuming there is negligible isotope influence on the binding orientation, only one quarter of the journeys around the cycle will result in 2DH → H_2_ + D_2_. Secondly, the reaction energies for all steps in the cycle are small, 1 or 7 kcal mol^−1^, so there is no deep energy well that would interrupt the cycle. Thirdly, the reaction barriers are 13, 2, 10, 9, 9 and 12 kcal mol^−1^ in sequence, limiting the rate of completion of the cycle. From this, and the statistical factor of one quarter, it is concluded that this cycle will have little or no ability to prosecute the forbidden reactivity at a competitive rate.

**SCHEME 9:**
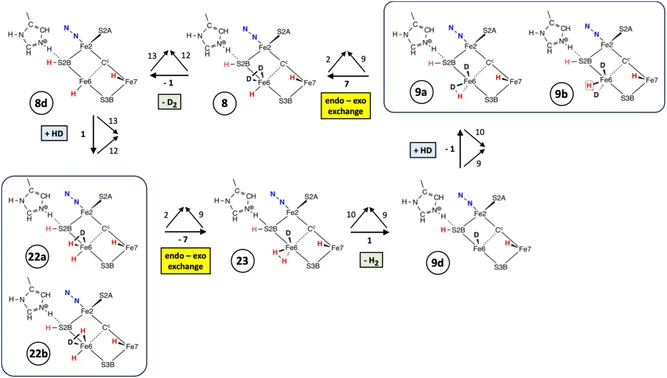
A reaction cycle (anti‐clockwise) that converts 2HD to H_2_ plus D_2_. The two possible orientations (**a** or **b**) of HD in each of the two HD binding steps are enclosed.

### 5.2 Concerted Double Hydrogenations

There is a difference between the concerted double hydrogenations of N_2_ and of D_2_. For N_2_, S2BH and 7Hn are the H donors, while for D_2_, the donors are 6Hx and 7Hn. The present results demonstrate that S2BH transfer to H_2_ is energetically unfavourable. This is attributed to the energy differences between a breaking H─S bond and a forming H─H bond, and to geometrical awkwardness. In the case of S2BH hydrogenating N_2_ in the N_2_‐capture step, the H─N bond that is forming competes better energetically with the breaking H─S bond. In addition, in the N_2_ capture reaction N_2_ freely tumbles into the reaction space and is well conformed to accept H from S2B (see Figure 2). There is a flaw in the calculated barriers for two of the steps in the N_2_ → 2NH_3_ reaction. For the N_2_ capture step, the barrier was computed as 26–29 kcal mol^−1^ [36, 39], but this is larger than allowed by the turnover rate of the enzyme (ca 1 s^−1^). I have suggested that inaccuracy in the computational methods is responsible for this discrepancy and encouraged independent checking of the N_2_ reaction steps. It is also probable that unaccounted H atom quantum tunneling occurs in the H atom transfer steps [39].

## 6 Conclusions

I conclude that the proposed **8** → **10** → **11** and **12** mechanism for the HD reaction is feasible and consistent with experiment. The difference with the previous sequence in Scheme 1 is the use endo‐Fe7─H instead of endo‐Fe2─H. Both mechanisms are concerted double hydrogenations of D_2_ (using exo‐Fe6─H as the other donor). The dynamics are similar in both mechanisms. The present mechanism is better aligned with the concerted N_2_‐capture step which uses endo‐Fe7─H and where endo‐Fe2─H would be obstructive. All of these proposals are components of an overall model of nitrogenase as a hydrogenator using H atom donors surrounding the reaction chamber between Fe2, Fe6 and Fe7, capped by Val70. I expect that hydrogenation of acetylene (with deuteration in the presence of D_2_) will also use H (D) atom donors around the reaction chamber.

## Conflicts of Interest

The author declares no conflicts of interest.

## Supporting information

Supplementary Material

## Data Availability

The data that supports the findings of this study are available in the Supporting Information of this article.
