# Supplementary material for: Explorations of the Hydrogenation of D2 by Nitrogenase
Source: Chembiochem. 2026 Oct 28;27(21):e70570. doi: 10.1002/cbic.70570 (PMC13717048; doi:10.1002/cbic.70570)
Supplement: Supplementary file 1 [file CBIC-27-e70570-s001.pdf]

Explorations of the Hydrogenation of D_2 by Nitrogenase

Ian Dance

School of Chemistry, UNSW Sydney, NSW 2052, Australia

E-mail: i.dance@unsw.edu.au

Supplementary Information

Atomic coordinates, Fe spin densities and S for reactants, transition states and products of the reaction steps

Translation of Fe numbering

Fe1 = Fe143
Fe2 = Fe140
Fe3 = Fe141
Fe4 = Fe142
Fe5 = Fe139
Fe6 = Fe145
Fe7 = Fe144

Table format

Fe spin densities<br>S	filename<br>coordinates

S2BH-6Hx-7Hn-6H2n parallel (Scheme 3) intermediate 7

Fe(139) -2.366<br>Fe(140) -1.040<br>Fe(141) -2.594<br>Fe(142) 2.675<br>Fe(143) 2.762<br>Fe(144) 1.401<br>Fe(145) 0.098<br><br>**S=1/2**	7hns2b6hx6h2ndtba_1_53154.03<br>C1 -7.684509308 -5.452697901 2.149964254<br>C2 -8.262888861 -6.570127597 1.275822791<br>C3 -8.185983569 -3.469065527 3.501506925<br>C4 -7.314773661 -3.732078975 4.726121669<br>C5 -6.664528221 -5.278920229 6.501573945<br>C6 -5.161545595 -5.345706302 6.219796283<br>C7 -3.375833766 -5.697283176 4.598233023<br>C8 -2.808371450 -4.281064409 4.475652811<br>C9 -3.264024858 -1.958610359 3.849175678<br>C10 -3.012242048 -1.327388772 5.226440401<br>C11 -3.748464350 -1.076013940 7.525614269<br>C12 -2.605494220 -1.695286486 8.341361378<br>C13 -1.628693304 -3.749157398 9.246528709<br>C14 -0.236346006 -4.058726275 8.679614694<br>C15 1.050462440 -4.753477959 6.698477725<br>C16 1.761180692 -3.667968865 5.886341811<br>C17 1.527919709 -1.621072134 4.522103732<br>C18 1.936539900 -0.433438547 5.423588466<br>C19 0.554520430 -1.195355344 3.400966814<br>C20 1.085921710 0.024518985 2.640442316<br>C21 0.278909797 -2.376575211 2.464174211<br>C22 1.181734258 1.289821428 7.025964002<br>C23 1.691021668 0.903208151 8.430423758<br>C24 -0.074537468 2.194622298 7.073585241<br>C25 0.108286669 3.341105080 8.075925073<br>C26 -0.393555008 2.747409332 5.678402257<br>C27 1.335252841 -0.317034428 10.501461823<br>C28 2.796149199 -0.690918260 10.645626827<br>C29 6.348093851 -11.692151143 2.961990815<br>C30 6.816101477 -10.387183844 2.315554570<br>C31 7.068648615 -7.941517001 2.745248870

C32	8.374766899	-7.588125895	3.483773694
C33	5.940996484	-6.955394796	3.101147179
C34	6.273199405	-5.505987621	2.739646500
C35	5.054175627	-4.593185197	2.882616085
C36	5.133357013	-2.070495991	2.972477930
C37	10.653869372	-6.721329292	3.243281980
C38	10.924301078	-5.234093250	3.209478180
C39	-5.609120370	-2.676556427	-0.932220235
C40	-5.911415248	-1.255504970	-0.417343638
C41	-4.074007496	-2.819284517	-1.052408772
C42	-3.538878218	-4.250774840	-1.264637515
C43	-3.431765142	-4.703490195	-2.720892858
C44	-6.458833071	1.017198518	-1.212476122
C45	-7.436827937	1.464651708	-0.117802149
C46	-6.763928874	1.657043016	-2.574949650
C47	-9.452499846	0.926792329	1.164718622
C48	-8.872631404	0.925854520	2.583695953
C49	-6.968109729	0.372790266	4.029745872
C50	-5.768460466	1.315476038	4.113125619
C51	-4.388639616	3.039056600	3.063558179
C52	-4.766271394	4.324527076	3.838226445
C53	-3.952219375	3.501495560	1.665774080
C54	-3.446117500	2.449677848	0.735238619
C55	-2.994625872	1.164872273	0.902864037
C56	-2.868416465	1.648558573	-1.269608665
C57	-6.563831520	5.965888113	4.221650320
C58	-6.307457863	6.074354760	5.724297519
C59	-8.080702954	6.034621503	3.923487356
C60	-8.687476774	7.406967239	3.887934404
C61	-8.489854761	8.502569956	4.706503193
C62	-9.989362795	8.966036983	3.142304934
C63	9.523741629	1.524814323	2.516462605
C64	10.530686876	0.429868862	2.216917427
C65	8.115746755	0.880468193	2.446756107
C66	7.012690064	1.854575029	2.787771369
C67	6.606693145	2.062142946	4.119782801
C68	6.362501940	2.588115892	1.780154629
C69	5.579221974	2.958380719	4.435706025
C70	5.346483893	3.496517191	2.081540269
C71	4.948954357	3.672038006	3.410477290
C72	5.129353600	6.849067667	-1.795990816
C73	4.062742945	7.954507980	-1.876043977
C74	4.499348550	5.534173716	-1.341389305
C75	2.892333243	9.566614204	-3.332088184
C76	1.408710713	9.192776495	-3.317569310
C77	-0.201619777	7.303510187	-3.134397986
C78	-0.516593225	6.946764080	-1.659794994
C79	-0.275627456	6.066084137	-4.058410541
C80	-1.540702844	5.201309161	-4.043443476
C81	-2.768129755	5.760835739	-4.782161139
C82	-4.222166386	7.673032794	-4.097478100
C83	0.220834588	5.982701824	0.462483808
C84	0.174917506	7.200893801	1.396251313
C85	1.264021344	4.998682277	1.027285435
C86	1.371682610	9.238857382	2.033512527
C87	0.701120250	10.587973598	1.826701793
C88	-0.888662851	11.873859335	0.500989377
C89	-2.079411869	12.427221686	1.325586113
C90	-1.217054259	11.904167503	-0.993830763
C91	-2.412310490	11.045311668	-1.383519673
C92	-3.836899235	11.936962136	2.972345959
C93	-3.633215219	13.007950997	4.034927209
C94	-4.419730726	10.646741260	3.617219695
C95	-4.781932287	9.595932593	2.590724545
C96	-6.053337025	9.590231948	1.993699037
C97	-3.850359105	8.634012301	2.165454502
C98	-6.376203874	8.675195533	0.987778449
C99	-4.145047599	7.726803079	1.142382534
C100	-5.409460380	7.765002621	0.559001653
C101	3.010026049	-3.752725393	-9.327140048
C102	1.637359396	-3.535164019	-8.717031910
C103	0.315122904	-2.056023565	-7.221741161
C104	-0.225075437	-0.690472792	-7.662184947
C105	0.202861393	1.726057852	-7.642448629
C106	1.359846504	2.684911133	-7.906879139
C107	3.689023075	3.314619264	-7.609620887
C108	4.130005774	3.531128550	-9.043692166
C109	-3.820077815	-3.302176318	-8.752917748
C110	-4.802814072	-2.229130282	-8.271626266
C111	-3.194029042	-4.022617038	-7.536531550

	C112	-2.524239204	-5.344093494	-7.956913728
	C113	-1.708000710	-5.905731335	-6.815432215
	C114	-5.130050707	0.078822979	-7.533625278
	C115	-4.725875777	1.372541387	-8.212713197
	C116	-5.323579960	0.185895631	-6.000627936
	C117	-4.000090531	0.166110212	-5.290406698
	C118	-3.238430966	1.339928005	-5.158323048
	C119	-3.435522791	-1.059653975	-4.894722304
	C120	-1.913839804	1.273964942	-4.716267287
	C121	-2.110984086	-1.123723094	-4.455683687
	C122	-1.337551574	0.039229078	-4.402036602
	C123	7.238179066	-6.123543729	-5.739122472
	C124	8.527764556	-6.229429912	-4.909498424
	C125	6.019274277	-6.498618477	-4.885493004
	C126	4.674227087	-6.250551712	-5.491350601
	C127	4.168701106	-6.776926044	-6.661636409
	C128	2.554872380	-5.708719979	-5.586072999
	C129	9.922793505	-7.486874793	-3.379130543
	C130	11.185049353	-7.211660858	-4.165818969
	C131	0.483332270	-7.013622906	-0.371629600
	C132	2.002269364	-7.079133965	-0.240626396
	C133	2.765465368	-7.153507598	-1.595674366
	C134	2.604826211	-8.569397306	-2.213445091
	C135	3.511513083	-8.876123465	-3.408873222
	C136	3.505375049	-10.360950780	-3.836416304
	C137	4.263538943	-6.919636463	-1.350034108
	C138	3.205742211	-0.693002151	-2.438643194
	Fe139	3.311658465	-1.950849542	-3.958635689
	Fe140	1.792863100	0.711883313	-1.686633948
	Fe141	4.753632090	0.425987607	-1.839164906
	Fe142	3.309233459	0.563590875	-3.967479862
	Fe143	3.471905321	2.678977010	-2.370746002
	Fe144	4.474515317	-2.083953299	-1.700991065
	Fe145	1.923813174	-2.407110011	-1.483393934
	H146	-6.527677961	-7.585548071	0.923970115
	H147	-6.935965653	-6.561034047	-0.277602343
	H148	-9.585126889	-4.764881347	2.543819821
	H149	-9.100159369	-6.163240299	0.686186518
	H150	-8.708466545	-7.316920669	1.955063873
	H151	-7.608225216	-2.797947023	2.849376052
	H152	-8.146966183	-5.584640742	4.972828941
	H153	-9.086382773	-2.932114398	3.829376065
	H154	-6.998420823	-6.258161304	6.868745680
	H155	-5.485381439	-5.858628052	4.242851098
	H156	-6.798937805	-4.549382877	7.311924556
	H157	-2.345846574	-1.829987325	3.264614286
	H158	-4.575320677	-3.636935923	3.658010878
	H159	-3.256821247	-6.215637610	3.636980372
	H160	-3.549937280	0.000305958	7.451132177
	H161	-4.734508709	-2.184720481	5.963180649
	H162	-2.774339877	-6.223560882	5.349669132
	H163	-4.077559258	-1.425400587	3.338058594
	H164	-3.271287959	-3.598995014	7.880508880
	H165	-4.691856399	-1.224242004	8.068382241
	H166	-0.202721802	-3.213353870	2.987572326
	H167	-0.387909369	-2.071352180	1.642235201
	H168	1.206057381	-2.753003730	2.006799189
	H169	-0.390546741	-0.896941916	3.885601809
	H170	-2.087305207	-4.703502128	9.544478250
	H171	-0.577053889	1.946326546	4.948756447
	H172	0.067962097	-0.357135355	8.793281034
	H173	0.260492095	2.979493005	9.103019290
	H174	2.024365719	-0.192601458	2.108846535
	H175	1.275663805	0.879461640	3.305269128
	H176	9.693905781	1.855789918	3.558980201
	H177	-1.291866360	3.381531743	5.716261963
	H178	-0.783703245	3.984615775	8.072260292
	H179	0.441792399	3.366105742	5.310878331
	H180	0.975886378	3.962779089	7.802455753
	H181	-0.925415065	1.574277741	7.408890016
	H182	2.012521996	1.854454575	6.579870482
	H183	-0.007687964	-0.222372649	6.069969174
	H184	0.345724185	0.357129147	1.897769755
	H185	1.468154908	4.223139664	0.274416670
	H186	0.807299649	4.521632085	1.908119756
	H187	2.848194254	5.235354446	2.175852560
	H188	3.720919288	4.588099016	4.604744371
	H189	4.842139895	4.048113911	1.288560335
	H190	6.634325765	2.426479120	0.733928225
	H191	7.087886961	1.501410339	4.925219632

	H192	-0.039890816	-2.826134700	5.367439866
	H193	0.850667742	-5.596877676	6.019578057
	H194	1.758297519	-5.090142928	7.464728402
	H195	-1.032628786	-4.330599239	6.785928757
	H196	-1.467707585	-3.148519369	10.150023105
	H197	5.260062213	3.087153371	5.473711991
	H198	2.454911442	-1.982935693	4.065653676
	H199	1.111165183	0.512829686	11.192368240
	H200	0.743724930	-1.191398552	10.815035452
	H201	3.260951764	-1.156608043	9.746047594
	H202	11.468641058	-7.253238028	2.729077343
	H203	-2.334802110	-5.914388296	-3.955684593
	H204	0.919344164	-6.108993025	-2.015007382
	H205	-3.301609003	-6.076176339	-8.217682078
	H206	-1.887414442	-5.183059079	-8.838439457
	H207	-3.966406842	-4.236477166	-6.781991655
	H208	-4.260133401	1.234270121	-9.216494526
	H209	-4.464905056	-4.031357366	-9.267079256
	H210	-3.730068950	-2.171847180	-1.876584508
	H211	-4.186094930	-4.980397277	-0.747631150
	H212	-3.266492371	-0.849659553	-8.059247334
	H213	-6.127616241	-0.142701792	-7.966705539
	H214	-2.145069169	-2.211590405	-9.322384605
	H215	-1.715089848	-5.822713811	-2.313777117
	H216	-2.464481907	-3.350297764	-7.058552497
	H217	-2.436037193	-3.542736636	-10.248903123
	H218	-7.159852023	-3.405849077	-2.086365790
	H219	-3.638492231	-2.435474090	-0.117200252
	H220	-0.105320168	-5.142316387	-7.661644554
	H221	-2.551379544	-4.340513481	-0.788081888
	H222	-3.546946273	2.594405731	3.612172172
	H223	-2.897803202	0.556086312	1.792102613
	H224	-7.687561766	0.705536833	4.791127615
	H225	-7.170997367	-0.095667249	1.958990040
	H226	-6.724781929	4.045705510	3.288535549
	H227	-7.836693757	8.672958188	5.554920789
	H228	-6.373774073	5.097996342	6.263483718
	H229	-8.616822329	5.386895988	4.639887998
	H230	-6.037270026	6.790333621	3.715312526
	H231	-10.714970295	9.538364705	2.570016668
	H232	-3.165621499	4.259687944	1.808750920
	H233	-4.801291266	4.008364765	1.179547664
	H234	-9.422741075	10.423626849	4.586411289
	H235	-8.244607883	5.594983449	2.926822681
	H236	-6.076126746	2.030052550	2.179167280
	H237	-6.628887069	-0.631104371	4.326642340
	H238	-9.920038069	1.911266232	1.023743448
	H239	-2.672043955	1.556187186	-2.335555055
	H240	-10.246361186	0.170326802	1.115315873
	H241	10.498870950	3.137067885	1.712318330
	H242	8.871832381	3.228922671	1.589009876
	H243	1.319767096	5.974264771	-1.395840800
	H244	-0.768155074	5.506386540	0.529545239
	H245	1.703829425	8.046556901	0.320750379
	H246	-3.724751981	3.593291643	-1.079602014
	H247	-5.466047517	4.225391645	-2.044549600
	H248	-7.223296524	3.454622447	-3.213506283
	H249	-5.047217024	5.462569522	-1.131653218
	H250	-6.002734331	1.312019826	-3.294248116
	H251	-7.755969076	1.335202165	-2.925823523
	H252	-0.322582637	11.642010308	-1.575168575
	H253	-1.481414095	12.944259017	-1.240830393
	H254	-1.312877917	10.184703071	-2.913925242
	H255	-2.977904815	9.642309289	-2.711925835
	H256	-4.306506937	9.321804272	-5.319433250
	H257	-3.320578291	4.942848973	-5.268903172
	H258	-7.373286356	8.676068426	0.540138917
	H259	-2.865576852	8.570503309	2.635948108
	H260	-5.029790096	7.809893744	-2.203878789
	H261	0.570996159	5.410617833	-3.795715668
	H262	1.925111562	7.222173782	-3.275845586
	H263	-1.817402787	4.923579556	-3.013834702
	H264	-8.571524616	-0.203728523	-0.435458531
	H265	3.112109531	10.034266025	-4.300970144
	H266	-6.499572887	-0.909250206	-2.288373108
	H267	-0.086787593	6.413574727	-5.087276025
	H268	-3.382104138	7.032719139	0.788911947
	H269	3.026918294	10.328347577	-2.551083205
	H270	-3.654528292	7.899107255	-6.070336324
	H271	-5.567282308	9.011846777	-3.329603675

	H272	-5.664963701	-3.468274711	-2.833148690
	H273	-5.949189025	-3.362077569	-0.134303577
	H274	-6.810342690	10.307467549	2.318453084
	H275	-5.486265107	1.391197233	-0.864367069
	H276	-0.933531744	8.062455145	-3.427302546
	H277	-2.434748049	6.436768243	-5.579472217
	H278	-1.268367159	4.263501660	-4.546914709
	H279	-4.036455818	5.924698631	-3.077542726
	H280	1.112844784	8.891397379	3.044540755
	H281	-0.513490033	9.757000403	0.408135499
	H282	-0.095564064	12.618448403	0.673178242
	H283	2.456146686	9.412369245	2.003228569
	H284	-2.250482347	10.661394050	2.308106473
	H285	3.494523918	-2.781689746	-9.506601892
	H286	3.608274341	-4.268273605	-8.547426549
	H287	7.166739451	-5.063138962	-6.023512236
	H288	3.824578682	-4.833293536	-10.851674542
	H289	2.319614587	-5.319830629	-10.428607692
	H290	5.298358336	4.784637739	-1.273288799
	H291	5.773751586	7.474240394	0.039444935
	H292	4.348560206	8.112303919	-3.909204820
	H293	4.035425213	5.666901549	-0.353782635
	H294	6.684502828	8.050235374	-1.191457165
	H295	4.552286917	2.928499718	-7.045762272
	H296	5.584703160	6.703016387	-2.788087171
	H297	5.721644247	-7.041545054	4.178302589
	H298	7.236235064	-7.924234860	1.660929844
	H299	2.793889681	1.484331303	-6.978294738
	H300	5.337229705	-0.050122627	2.728655257
	H301	-0.411262434	1.693855465	-8.550876280
	H302	-1.675491944	-2.083080553	-4.166641117
	H303	-0.417609473	2.200599453	-6.862756505
	H304	4.735050056	-4.530889611	3.929307278
	H305	1.295006125	0.163064490	-6.606911630
	H306	4.206493961	-5.006427707	2.310763098
	H307	5.044071250	-7.281648980	2.551074560
	H308	6.619978363	-5.455934526	1.696494805
	H309	7.088915610	-5.122055516	3.372828521
	H310	-5.949003092	-0.660698175	-5.682797045
	H311	6.354732423	-9.503258523	4.050429159
	H312	5.391283639	-11.942821363	2.478646864
	H313	-0.292205947	0.007332749	-4.100209816
	H314	7.000733790	-11.818309138	4.910683006
	H315	5.435764929	-12.258085043	4.715767778
	H316	5.637610868	-3.216567695	1.389474425
	H317	-1.305529773	2.175888474	-4.631565763
	H318	-4.029223668	-1.974822048	-4.951678196
	H319	7.300877578	-9.920952989	-2.689943098
	H320	6.073918007	-7.573055491	-4.636741214
	H321	6.492827160	-8.234360728	-0.972826416
	H322	6.314043239	-10.504437415	-3.746080592
	H323	3.810881132	2.735847541	-9.760186445
	H324	6.263116627	-5.159046854	-1.110746843
	H325	2.271536187	-7.920911217	0.408710150
	H326	2.346768194	-1.864597965	-7.771933803
	H327	2.826276134	-9.285363607	-1.406117393
	H328	2.159506867	-6.710098171	-7.400035988
	H329	1.563331641	-5.341050083	-5.356127110
	H330	7.068017305	-12.469919878	2.656142518
	H331	5.634418833	-0.963725323	1.283274383
	H332	8.364464734	-5.851274716	-0.266328288
	H333	7.070139431	-3.832744713	-1.075548385
	H334	9.193428044	-6.942376736	1.677750498
	H335	4.460324514	-2.794617590	4.772563881
	H336	3.191350873	-8.277105556	-4.275246513
	H337	4.549190046	-8.590936715	-3.187251817
	H338	1.549671335	-8.718720064	-2.491263034
	H339	0.548620424	-2.050251442	-6.145048639
	H340	-3.676339681	2.296028437	-5.454965926
	H341	-5.876187488	1.118902280	-5.812534211
	H342	2.295490846	-6.151691333	0.280205838
	H343	8.469700384	-7.385173865	-0.482907193
	H344	7.382965575	-9.301319096	-0.296859278
	H345	4.399145175	-1.104015762	4.623020305
	H346	4.638632702	-7.385731278	-7.425295752
	H347	6.076772409	-5.967475078	-3.928932739
	H348	-0.453305102	-2.806587428	-7.417913453
	H349	3.412487245	4.285084064	-7.169535441
	H350	8.101411946	0.031234376	3.146145146
	H351	7.962741926	0.482002802	1.430771795

	H352	8.112393491	-6.598345288	-7.532543044
	H353	7.473440209	-7.896543060	-6.767204158
	H354	9.982655378	-8.525230505	-3.017868427
	H355	8.055777849	-8.140125952	-4.175896249
	H356	11.119254264	-7.447325380	-5.255231077
	H357	9.885437322	-6.828412927	-2.496565193
	H358	10.662316047	0.213189698	1.129216185
	H359	-2.572745767	13.184322460	4.333014998
	H360	10.662631970	-7.028643466	4.304030272
	H361	10.015287719	-4.585415845	3.236041219
	H362	-4.561919841	12.333431906	2.242190054
	H363	-3.684579131	10.244155728	4.331892594
	H364	-5.307556121	10.943146117	4.193423712
	H365	0.961332206	-3.381403930	-0.845179018
	H366	4.375424217	-1.500806912	-0.330998799
	H367	-6.650765519	6.870231828	-0.647811859
	H368	-0.180872799	-0.880708717	-0.376729457
	H369	1.819534312	-1.376134750	-0.127234124
	H370	2.358838384	-1.921696784	0.103318363
	H371	-2.249495005	-0.213079157	-0.572105373
	Mo372	3.455517478	-4.393276816	-2.776266926
	N373	-7.324743631	-7.234782920	0.386623102
	N374	-8.601613238	-4.663698149	2.783181948
	N375	-7.472405454	-4.922050249	5.347849971
	N376	-4.782233176	-5.717247922	4.970538172
	N377	-3.629021886	-3.364139545	3.912378301
	N378	-3.895788854	-1.649720795	6.197761917
	N379	-2.556071508	-3.051499346	8.367897771
	N380	-0.183542585	-4.356768100	7.352595324
	N381	0.974072489	-2.723630348	5.312155193
	N382	0.950570628	0.119770791	6.173503823
	N383	0.970893983	-0.010998109	9.127505248
	N384	6.139801771	-11.584173386	4.411367565
	N385	6.674707993	-9.298231029	3.098211716
	N386	5.333797141	-3.249883917	2.363907864
	N387	4.836473114	-1.977290676	4.281748516
	N388	5.254580154	-0.940824516	2.243591017
	N389	9.357741289	-7.079169855	2.699536217
	N390	-6.279104192	-2.904506339	-2.217059432
	N391	-2.426757741	-5.553921443	-3.000142053
	N392	-6.388748322	-0.424431945	-1.381067499
	N393	-8.501287042	0.659391085	0.098928582
	N394	-7.660986498	0.325078382	2.755826212
	N395	-5.473259197	2.066964650	3.006833503
	N396	-3.352749186	2.716321142	-0.623873873
	N397	-2.656775478	0.696189772	-0.351750381
	N398	-6.071934493	4.681281045	3.740533292
	N399	-9.626595809	7.709354556	2.914839523
	N400	-9.325994593	9.482501232	4.214561396
	N401	9.662639935	2.582731100	1.513124059
	N402	6.202345043	7.211994975	-0.854379028
	N403	3.821238229	8.471023967	-3.117447735
	N404	1.132399498	7.872301137	-3.288459966
	N405	-3.699588597	6.465072778	-3.894038110
	N406	-4.058423329	8.339986183	-5.252605469
	N407	-4.889374685	8.291530922	-3.095091731
	N408	0.467742917	6.340849081	-0.936107004
	N409	1.022118963	8.215177300	1.066018487
	N410	-0.348171329	10.605207934	0.949371958
	N411	-2.256647123	10.339813988	-2.537081223
	N412	-2.585154480	11.621339510	2.292365161
	N413	2.898264343	-4.490215019	-10.588314779
	N414	1.508076454	-2.417077614	-7.973844352
	N415	0.532341232	0.360737672	-7.275792248
	N416	2.606620660	2.351564409	-7.488594445
	N417	-2.889190850	-2.771355015	-9.755227495
	N418	-4.275509936	-1.006387477	-7.977170207
	N419	7.304670196	-6.909452298	-6.985026432
	N420	3.648548952	-5.568261022	-4.835842468
	N421	2.837576296	-6.424033033	-6.698971409
	N422	8.734342847	-7.375356592	-4.206079615
	N423	-0.101048355	2.387037564	-0.188875144
	N424	0.631860101	1.744710156	-0.760605116
	O425	-6.463761243	-5.281143454	2.313135985
	O426	-6.504584292	-2.871387001	5.117262482
	O427	-4.343928352	-5.084753482	7.118246923
	O428	-1.660211178	-3.998205927	4.867625458
	O429	-2.048220219	-0.566763327	5.414574689
	O430	-1.809687240	-0.976232213	8.970610782
	O431	0.743942571	-4.082435065	9.435204286

	O432 2.998503302 -3.712085682 5.757365733<br>O433 3.107189095 -0.004198020 5.430084110<br>O434 2.714047626 1.428072851 8.902334170<br>O435 3.410547089 -0.566510369 11.695383503<br>O436 7.254652339 -10.393515876 1.146792581<br>O437 8.479837270 -7.769444243 4.709514490<br>O438 12.045812501 -4.751928510 3.172083816<br>O439 -5.711029776 -0.899315581 0.760627000<br>O440 -4.248206580 -4.327706786 -3.593321288<br>O441 -7.223578918 2.499833407 0.538233817<br>O442 -6.730884442 3.092835298 -2.455383630<br>O443 -9.519103528 1.448601416 3.500578915<br>O444 -5.107094132 1.378253529 5.154989490<br>O445 -3.913741408 5.008958665 4.412099265<br>O446 -6.093556754 7.119655000 6.312584049<br>O447 11.144493076 -0.194309362 3.070105184<br>O448 3.912187373 4.554868353 3.649614249<br>O449 3.463159101 8.371596764 -0.869229392<br>O450 0.548294381 10.093983122 -3.344841853<br>O451 -1.631523569 7.213609795 -1.182746190<br>O452 -0.545193024 7.203870690 2.403051734<br>O453 2.469972253 5.676643852 1.388143219<br>O454 1.088634793 11.589118718 2.441141307<br>O455 -2.491337942 13.578908126 1.127208466<br>O456 -3.463906255 11.039048508 -0.727799958<br>O457 -4.558580745 13.617272387 4.546874537<br>O458 -5.684167470 6.880394717 -0.505671061<br>O459 0.715303118 -4.370390785 -8.884465489<br>O460 -1.264406857 -0.578804855 -8.343903570<br>O461 1.116944073 3.772188420 -8.456733214<br>O462 4.809168960 4.479850683 -9.398584375<br>O463 -6.013226656 -2.474231991 -8.136921241<br>O464 -2.198870071 -6.517738324 -5.872432568<br>O465 -0.383568363 -5.653721104 -6.839967867<br>O466 -4.925819415 2.488905265 -7.755957462<br>O467 9.381529513 -5.321298642 -4.911405758<br>O468 12.232984076 -6.837065806 -3.658251103<br>O469 0.034401034 -6.359322117 -1.449959707<br>O470 -0.277725136 -7.475880401 0.476387894<br>O471 4.637565951 -10.893177146 -4.110297324<br>O472 2.384593843 -10.939869459 -3.911438860<br>O473 4.815609451 -5.931340423 -2.010351793<br>O474 4.898277896 -7.663266467 -0.580914734<br>O475 2.253461878 -6.122247915 -2.442062641<br>O476 8.961324887 -6.617348592 -0.079938604<br>O477 6.953110371 -4.673335864 -0.592816628<br>O478 -4.698763651 4.803534208 -1.783375940<br>O479 7.291989730 -10.387992910 -3.554947585<br>O480 7.382171553 -8.671191471 -1.060951956<br>S481 3.195820144 4.939920547 -2.514943168<br>S482 1.624851675 1.996747348 -3.522345562<br>S483 1.522479405 -3.286688307 -3.556548955<br>S484 3.488513633 1.648013885 -0.396018316<br>S485 0.182013521 -0.976876445 -1.690876998<br>S486 3.326343378 -0.699520110 -5.785128446<br>S487 3.469523230 -3.714081167 -0.556661329<br>S488 5.206693507 1.757732418 -3.548100267<br>S489 5.224158509 -3.056772276 -3.626669022<br>S490 6.348030286 -0.900719595 -1.098259418<br>end

TS for 6H2n dissociation (Scheme 3) intermediate 7 to 7d

Fe(139) -2.275<br>Fe(140) -1.094<br>Fe(141) -2.637<br>Fe(142) 2.672<br>Fe(143) 2.773<br>Fe(144) 1.461<br>Fe(145) 0.001<br><br>**S=1/2**	7hns2b6hx6h2ndtc.car_1<br>C1 -7.687087264 -5.451748770 2.146823212<br>C2 -8.267319938 -6.567361014 1.271608882<br>C3 -8.183139354 -3.466661950 3.497480087<br>C4 -7.313093029 -3.730525440 4.722752566<br>C5 -6.663950840 -5.278160624 6.497838762<br>C6 -5.160769149 -5.344111660 6.216929856<br>C7 -3.373826906 -5.694718226 4.596529418<br>C8 -2.807209481 -4.278113504 4.474063649<br>C9 -3.263560025 -1.956159982 3.844495787<br>C10 -3.013456625 -1.323045594 5.221206507<br>C11 -3.747994843 -1.072538240 7.520720599<br>C12 -2.605110565 -1.691932558 8.336498658<br>C13 -1.629401329 -3.746243034 9.242061125<br>C14 -0.236692101 -4.056278754 8.676312444

	C15	1.051375190	-4.751988647	6.696258739
	C16	1.762053777	-3.668242751	5.881689640
	C17	1.527618386	-1.618554696	4.519225034
	C18	1.937786068	-0.432461843	5.422069070
	C19	0.547908069	-1.192557960	3.404938815
	C20	1.069502694	0.037034497	2.649973500
	C21	0.268884819	-2.377589593	2.470801222
	C22	1.183419641	1.291934611	7.023832624
	C23	1.692333283	0.904657262	8.427967119
	C24	-0.072756734	2.196842656	7.072542907
	C25	0.110369843	3.342536639	8.075702569
	C26	-0.392057037	2.750430788	5.677787954
	C27	1.335337978	-0.315948473	10.498090790
	C28	2.797425927	-0.683047338	10.645879304
	C29	6.342297682	-11.691148586	2.961878243
	C30	6.812978871	-10.386342023	2.315974711
	C31	7.067561442	-7.940305579	2.744718955
	C32	8.374209917	-7.586153045	3.482058786
	C33	5.940068880	-6.953743085	3.100324738
	C34	6.271274834	-5.504184218	2.737954841
	C35	5.049100181	-4.594321406	2.873801877
	C36	5.132963787	-2.072503485	2.970704748
	C37	10.653005900	-6.719181541	3.239531318
	C38	10.924575978	-5.232080883	3.207288644
	C39	-5.610141310	-2.678044164	-0.929587439
	C40	-5.910843927	-1.256695501	-0.414468337
	C41	-4.074952233	-2.820656097	-1.050732592
	C42	-3.538967110	-4.251572124	-1.264572667
	C43	-3.432189284	-4.703101772	-2.721188356
	C44	-6.458957544	1.016162580	-1.209704842
	C45	-7.438210861	1.461976076	-0.115428846
	C46	-6.764679144	1.655306188	-2.572392008
	C47	-9.453244653	0.919663996	1.166620199
	C48	-8.873805230	0.920149560	2.585786865
	C49	-6.968373851	0.371935786	4.032414773
	C50	-5.770131568	1.316454703	4.114997251
	C51	-4.389503947	3.037500854	3.062302041
	C52	-4.767961553	4.323601236	3.835865767
	C53	-3.953948448	3.498916942	1.663769358
	C54	-3.443040968	2.448252024	0.734021568
	C55	-2.984198343	1.165975424	0.902092946
	C56	-2.861079277	1.648581932	-1.270432565
	C57	-6.567210655	5.963137645	4.219548486
	C58	-6.314802963	6.071082605	5.723066394
	C59	-8.083486565	6.031648199	3.917921854
	C60	-8.691223803	7.403696668	3.882743854
	C61	-8.492108641	8.500191994	4.699755247
	C62	-9.995905181	8.961413241	3.138957795
	C63	9.519805682	1.522175183	2.515858258
	C64	10.527085429	0.427010425	2.218274022
	C65	8.111508491	0.879279667	2.447907152
	C66	7.010361285	1.855020356	2.790005200
	C67	6.604625058	2.062421539	4.122083685
	C68	6.361750207	2.590302107	1.782753470
	C69	5.577799192	2.959349659	4.438161338
	C70	5.346724565	3.499557184	2.084392150
	C71	4.948481231	3.674150450	3.413165359
	C72	5.130137871	6.847976283	-1.794611036
	C73	4.063722061	7.954139476	-1.876220388
	C74	4.498781596	5.533614331	-1.342913857
	C75	2.894517848	9.567234852	-3.332460430
	C76	1.410547755	9.194115818	-3.316160485
	C77	-0.200837314	7.305340279	-3.135112135
	C78	-0.516156553	6.950091880	-1.660401618
	C79	-0.277007913	6.067921742	-4.058584514
	C80	-1.542965757	5.204143630	-4.041353537
	C81	-2.770823586	5.762843619	-4.780260233
	C82	-4.224780662	7.674504197	-4.094072764
	C83	0.221858675	5.986872855	0.462075875
	C84	0.179010908	7.205545912	1.395257019
	C85	1.263949052	5.001521655	1.026416853
	C86	1.375518787	9.244834453	2.027397661
	C87	0.701627226	10.592323506	1.821756684
	C88	-0.890999744	11.874090883	0.497252077
	C89	-2.081057833	12.427930079	1.323116609
	C90	-1.222439208	11.905403869	-0.996739638
	C91	-2.415622959	11.042488586	-1.383243025
	C92	-3.837864396	11.937300413	2.970233547
	C93	-3.634190171	13.008155938	4.033224993
	C94	-4.421533018	10.647816176	3.615302388

	C95	-4.784351921	9.596839100	2.589355776
	C96	-6.056098220	9.590371190	1.993130255
	C97	-3.852596595	8.635285819	2.163869041
	C98	-6.379370989	8.674255276	0.988268994
	C99	-4.147715856	7.727138696	1.141819442
	C100	-5.412686524	7.763905067	0.559601993
	C101	3.008443303	-3.750418253	-9.331826240
	C102	1.635700151	-3.532843748	-8.721842978
	C103	0.313306167	-2.054495942	-7.225864303
	C104	-0.226996542	-0.688190966	-7.664028321
	C105	0.199798072	1.728269428	-7.641734891
	C106	1.355476296	2.688674641	-7.906296170
	C107	3.684652750	3.320214629	-7.609310186
	C108	4.137138974	3.523279301	-9.041699789
	C109	-3.823829357	-3.303504464	-8.754599106
	C110	-4.806796141	-2.231038056	-8.272018812
	C111	-3.196453123	-4.023273790	-7.538117490
	C112	-2.525671602	-5.344533002	-7.957863654
	C113	-1.707263502	-5.905144913	-6.817363406
	C114	-5.135458196	0.077012358	-7.532855135
	C115	-4.730490800	1.372101230	-8.209582438
	C116	-5.325298529	0.182203468	-5.999007673
	C117	-4.000579158	0.161203785	-5.290100916
	C118	-3.239136033	1.335037764	-5.155650883
	C119	-3.434819470	-1.064822456	-4.896555089
	C120	-1.913795971	1.269173694	-4.715293029
	C121	-2.109549902	-1.129008335	-4.458851606
	C122	-1.336346345	0.034020828	-4.403758275
	C123	7.236710484	-6.119215467	-5.737077693
	C124	8.526065730	-6.226845158	-4.907056093
	C125	6.016733728	-6.505141286	-4.891224592
	C126	4.673824159	-6.263145194	-5.503828660
	C127	4.171067001	-6.789902593	-6.675285027
	C128	2.555171396	-5.719158244	-5.603769038
	C129	9.920146684	-7.487615831	-3.377352685
	C130	11.182268253	-7.217523861	-4.166270101
	C131	0.480722055	-7.018616675	-0.375198012
	C132	1.999015220	-7.084462378	-0.246073851
	C133	2.762095146	-7.164766730	-1.600936133
	C134	2.603242530	-8.582780242	-2.212639901
	C135	3.509797149	-8.884431230	-3.408926366
	C136	3.503702736	-10.367490694	-3.840085242
	C137	4.259968108	-6.931448218	-1.354557673
	C138	3.203258791	-0.692823424	-2.442358875
	Fe139	3.303657521	-1.948841973	-3.949325523
	Fe140	1.812057102	0.725356831	-1.682887207
	Fe141	4.760020999	0.408618955	-1.831046596
	Fe142	3.324746106	0.559922046	-3.966985802
	Fe143	3.492171962	2.680696545	-2.382514265
	Fe144	4.476757976	-2.107140931	-1.707623143
	Fe145	1.948436824	-2.414360398	-1.597613497
	H146	-6.533587991	-7.585642897	0.919779257
	H147	-6.939198036	-6.559564516	-0.281276716
	H148	-9.586103876	-4.759301599	2.540516735
	H149	-9.103251267	-6.158112382	0.681743514
	H150	-8.714651714	-7.314071778	1.949885736
	H151	-7.602727420	-2.798317444	2.844675649
	H152	-8.145669687	-5.583014532	4.968140790
	H153	-9.081773539	-2.926291355	3.824468314
	H154	-6.997685955	-6.257823371	6.864017796
	H155	-5.483133364	-5.857772303	4.239944013
	H156	-6.799012447	-4.549264052	7.308660978
	H157	-2.345032356	-1.827697120	3.260435283
	H158	-4.573664948	-3.635553102	3.654566780
	H159	-3.253761455	-6.213119389	3.635437165
	H160	-3.548878389	0.003575267	7.445339797
	H161	-4.733350540	-2.183926140	5.959703850
	H162	-2.772446353	-6.220484163	5.348430291
	H163	-4.077258309	-1.424434449	3.332025057
	H164	-3.270925994	-3.595415034	7.875044760
	H165	-4.691278683	-1.219992005	8.063886494
	H166	-0.218425011	-3.209406241	2.997369789
	H167	-0.391457334	-2.072131340	1.643470348
	H168	1.194196276	-2.760538221	2.017732133
	H169	-0.395649704	-0.899444802	3.894830218
	H170	-2.088609927	-4.700491225	9.539407773
	H171	-0.575423902	1.949643335	4.947840618
	H172	0.069218193	-0.356285203	8.789220032
	H173	0.262703001	2.980230395	9.102531199
	H174	1.989725976	-0.182075589	2.092534309

	H175	1.285635946	0.881491731	3.321467882
	H176	9.690188472	1.853499046	3.558059991
	H177	-1.290604654	3.384163487	5.716050237
	H178	-0.781480112	3.986235862	8.072666531
	H179	0.443184585	3.369545951	5.310680759
	H180	0.978060971	3.964230661	7.802610451
	H181	-0.923536261	1.576264570	7.407668651
	H182	2.014188445	1.856871152	6.578097261
	H183	-0.007191736	-0.216967765	6.064791826
	H184	0.313271257	0.382588637	1.928952947
	H185	1.467137972	4.226209922	0.273219429
	H186	0.806840234	4.524555824	1.907079748
	H187	2.847024042	5.237873142	2.176403469
	H188	3.721377375	4.591586595	4.607450934
	H189	4.843431276	4.052363514	1.291700284
	H190	6.634330799	2.429324949	0.736609504
	H191	7.085124663	1.500812778	4.927332098
	H192	-0.038281570	-2.822500959	5.368705217
	H193	0.852292104	-5.597096998	6.019285925
	H194	1.759250351	-5.086741057	7.463299044
	H195	-1.031522477	-4.328286989	6.782052998
	H196	-1.468960492	-3.145944818	10.145877692
	H197	5.258247285	3.087493551	5.476060481
	H198	2.453848914	-1.979464518	4.060435771
	H199	1.106119743	0.512529550	11.188691516
	H200	0.747039374	-1.192871799	10.810388048
	H201	3.266063039	-1.148581866	9.748287594
	H202	11.466919068	-7.250655793	2.723633622
	H203	-2.335487793	-5.912753042	-3.957746194
	H204	0.917610579	-6.123893085	-2.026511278
	H205	-3.302408926	-6.077774721	-8.216988247
	H206	-1.889926598	-5.183889992	-8.840244697
	H207	-3.967687136	-4.237049136	-6.782416300
	H208	-4.263740225	1.235399815	-9.213155579
	H209	-4.468392534	-4.032966019	-9.269144369
	H210	-3.731959307	-2.172284798	-1.874519579
	H211	-4.185068017	-4.982174757	-0.747642941
	H212	-3.271718419	-0.849599496	-8.061359864
	H213	-6.133847027	-0.143553458	-7.964780363
	H214	-2.149329322	-2.210619927	-9.321737700
	H215	-1.716166390	-5.823746860	-2.315527997
	H216	-2.466763618	-3.350176124	-7.061425621
	H217	-2.436780823	-3.542651442	-10.247555343
	H218	-7.160607753	-3.406739653	-2.084504252
	H219	-3.638715294	-2.437174106	-0.115797530
	H220	-0.105936159	-5.141198100	-7.665969681
	H221	-2.551121386	-4.340884713	-0.788702630
	H222	-3.547588706	2.594432096	3.611845378
	H223	-2.884177264	0.558009112	1.791401500
	H224	-7.688565380	0.704193087	4.793281470
	H225	-7.169920514	-0.098182729	1.961848491
	H226	-6.726654551	4.042690556	3.287262358
	H227	-7.837076588	8.671501545	5.546571465
	H228	-6.385291983	5.095062819	6.262409874
	H229	-8.621256626	5.382901316	4.632212515
	H230	-6.039737629	6.788198407	3.715150105
	H231	-10.723132565	9.532987029	2.567900002
	H232	-3.170496847	4.260448822	1.806188763
	H233	-4.805160936	4.001792152	1.176950631
	H234	-9.426385548	10.420681796	4.580254838
	H235	-8.245068336	5.593033728	2.920417405
	H236	-6.076430724	2.026739823	2.179271402
	H237	-6.627556355	-0.631111705	4.330367689
	H238	-9.922906721	1.903090152	1.025557063
	H239	-2.665163857	1.557278103	-2.336549522
	H240	-10.245361250	0.161400235	1.116768225
	H241	10.498280480	3.132636363	1.712632116
	H242	8.871646778	3.228639565	1.589997191
	H243	1.319887650	5.978419513	-1.397005569
	H244	-0.767987106	5.512487744	0.530251780
	H245	1.704680050	8.051606355	0.314682353
	H246	-3.725457675	3.590189571	-1.081040561
	H247	-5.469514122	4.224125232	-2.044124676
	H248	-7.227639731	3.451573944	-3.211609683
	H249	-5.050147201	5.460711413	-1.130958715
	H250	-6.002537061	1.311514808	-3.291237663
	H251	-7.755864539	1.331280208	-2.923716767
	H252	-0.328363985	11.646821485	-1.580198064
	H253	-1.490970995	12.945062764	-1.241153764
	H254	-1.312038226	10.177978284	-2.907664205

	H255 -2.973967361 9.628042503 -2.702071315<br>H256 -4.296333217 9.329169371 -5.308456124<br>H257 -3.322511451 4.944504006 -5.267240413<br>H258 -7.376873438 8.674566552 0.541636521<br>H259 -2.867480485 8.572884821 2.633778128<br>H260 -5.039077580 7.806678010 -2.202667643<br>H261 0.569313345 5.411903456 -3.796366324<br>H262 1.925803064 7.223000674 -3.279412241<br>H263 -1.819327788 4.928493559 -3.011062514<br>H264 -8.569365221 -0.208674306 -0.433373256<br>H265 3.113400225 10.033114710 -4.302435332<br>H266 -6.498925160 -0.910587283 -2.285354654<br>H267 -0.089236735 6.414921428 -5.087857226<br>H268 -3.384862402 7.032981827 0.788500627<br>H269 3.030210032 10.330549072 -2.553214472<br>H270 -3.642798819 7.908977875 -6.061776548<br>H271 -5.569507326 9.013594337 -3.326395460<br>H272 -5.665591383 -3.467998492 -2.831255524<br>H273 -5.950187403 -3.363616657 -0.131688008<br>H274 -6.813013178 10.307774157 2.317816162<br>H275 -5.486961584 1.391597260 -0.861334978<br>H276 -0.932126388 8.064607684 -3.428472188<br>H277 -2.438035799 6.438959598 -5.577667296<br>H278 -1.271552573 4.265384350 -4.543563924<br>H279 -4.041994032 5.924124865 -3.077430018<br>H280 1.120723016 8.897532162 3.039488726<br>H281 -0.513247711 9.758374753 0.404463882<br>H282 -0.098768643 12.619981838 0.667844937<br>H283 2.459502654 9.420319535 1.993214556<br>H284 -2.252241380 10.661925670 2.305235738<br>H285 3.494215900 -2.779625145 -9.508508261<br>H286 3.605836280 -4.267466067 -8.552433919<br>H287 7.161369651 -5.057127270 -6.014144478<br>H288 3.823904676 -4.830051476 -10.856008749<br>H289 2.316463743 -5.313643414 -10.437096683<br>H290 5.297098563 4.783558468 -1.273885302<br>H291 5.769275123 7.474872764 0.042661132<br>H292 4.350748466 8.112588638 -3.909067180<br>H293 4.031974385 5.665109576 -0.356408938<br>H294 6.683013143 8.050222323 -1.185439985<br>H295 4.544070206 2.941537305 -7.034300725<br>H296 5.588718883 6.703244772 -2.785473258<br>H297 5.720881900 -7.039069254 4.177583293<br>H298 7.234539299 -7.923469127 1.660283488<br>H299 2.789796221 1.490350087 -6.975806971<br>H300 5.336215252 -0.051599411 2.735161583<br>H301 -0.415123525 1.696319784 -8.549714368<br>H302 -1.672685800 -2.089008574 -4.173573830<br>H303 -0.420635111 2.201249112 -6.861029256<br>H304 4.723618268 -4.532134682 3.918433129<br>H305 1.293356854 0.164301694 -6.608009567<br>H306 4.205446927 -5.010603567 2.298356579<br>H307 5.043049396 -7.280466188 2.550667251<br>H308 6.622205095 -5.454364072 1.696241706<br>H309 7.083010091 -5.117158636 3.374344419<br>H310 -5.950907236 -0.664183863 -5.681035815<br>H311 6.353835435 -9.501256057 4.051154315<br>H312 5.383486492 -11.936607060 2.479774179<br>H313 -0.290471842 0.000698669 -4.103519919<br>H314 6.996421529 -11.819399761 4.909775749<br>H315 5.429924889 -12.255137928 4.716402288<br>H316 5.625757701 -3.213267737 1.380613118<br>H317 -1.306457132 2.171931754 -4.630616023<br>H318 -4.027695969 -1.980446185 -4.953989242<br>H319 7.296584792 -9.919948629 -2.690021176<br>H320 6.077144388 -7.579628856 -4.645276246<br>H321 6.489590026 -8.233803668 -0.972002444<br>H322 6.310123109 -10.506230733 -3.745503763<br>H323 3.819083128 2.724817559 -9.754965071<br>H324 6.256522762 -5.161790717 -1.111963807<br>H325 2.269168324 -7.923463139 0.406242019<br>H326 2.345164357 -1.862783324 -7.775229275<br>H327 2.827001368 -9.297543133 -1.404520342<br>H328 2.164087483 -6.722230438 -7.418359968<br>H329 1.562462191 -5.352106907 -5.376194252<br>H330 7.058811424 -12.471305508 2.653876350<br>H331 5.622331065 -0.960021147 1.281744008<br>H332 8.361603327 -5.850793265 -0.268573436<br>H333 7.061936980 -3.833655589 -1.075657124<br>H334 9.191010270 -6.939704856 1.675380861

	H335	4.464908722	-2.801340708	4.770296057
	H336	3.187406484	-8.281645415	-4.271963664
	H337	4.547213035	-8.597994681	-3.186726787
	H338	1.548320726	-8.733862230	-2.490140425
	H339	0.545910505	-2.050486476	-6.148969265
	H340	-3.678203531	2.291622426	-5.448995086
	H341	-5.877351717	1.115116429	-5.808808025
	H342	2.293265148	-6.155363470	0.271154553
	H343	8.467354826	-7.384691124	-0.484448295
	H344	7.380582931	-9.300893317	-0.297051118
	H345	4.407287742	-1.111135284	4.626067920
	H346	4.642592829	-7.399036954	-7.437899250
	H347	6.065651561	-5.976941377	-3.932540864
	H348	-0.455375663	-2.804416100	-7.423114918
	H349	3.401750158	4.294345802	-7.181659182
	H350	8.097542770	0.030508444	3.147873232
	H351	7.956862599	0.480281531	1.432468864
	H352	8.113446369	-6.579783128	-7.533531676
	H353	7.479214362	-7.884598912	-6.774488177
	H354	9.976482340	-8.525683399	-3.014460339
	H355	8.050024875	-8.135383331	-4.172091482
	H356	11.112943711	-7.453458478	-5.255410025
	H357	9.886090248	-6.827653363	-2.495854039
	H358	10.661570570	0.210370798	1.131027067
	H359	-2.573957539	13.184850456	4.331591024
	H360	10.662851259	-7.027761608	4.299808361
	H361	10.016118700	-4.582547082	3.231035514
	H362	-4.563081033	12.335047272	2.240985496
	H363	-3.686575385	10.245299982	4.330170781
	H364	-5.309043526	10.945056754	4.191600215
	H365	0.980264229	-3.347881323	-0.932902468
	H366	4.336482448	-1.535098529	-0.337647337
	H367	-6.654480774	6.867068652	-0.645250041
	H368	-0.086674334	-0.974156596	-0.345309205
	H369	2.034790112	-1.244747970	0.317430018
	H370	2.493236118	-1.838872596	0.432250439
	H371	-2.227962922	-0.208635005	-0.570673320
	Mo372	3.475220056	-4.413600617	-2.792391321
	N373	-7.329745116	-7.232927810	0.382351999
	N374	-8.602417210	-4.661053942	2.780647510
	N375	-7.471277567	-4.920664913	5.343905688
	N376	-4.780460405	-5.715461241	4.967896907
	N377	-3.627537550	-3.362058220	3.908755133
	N378	-3.895613865	-1.646883127	6.193197980
	N379	-2.555892559	-3.048127749	8.362899659
	N380	-0.182913488	-4.354758528	7.349486538
	N381	0.975686506	-2.721746004	5.310690801
	N382	0.951673360	0.122503595	6.170909357
	N383	0.972560087	-0.011060889	9.123524619
	N384	6.135616567	-11.583280352	4.411310703
	N385	6.673733805	-9.297035687	3.098751614
	N386	5.325889313	-3.250103176	2.356190956
	N387	4.843874622	-1.983576608	4.282464390
	N388	5.253502584	-0.940385513	2.246851516
	N389	9.356149624	-7.076323825	2.697007401
	N390	-6.280005262	-2.905084850	-2.214633303
	N391	-2.427046126	-5.552773880	-3.001910362
	N392	-6.386996062	-0.425286297	-1.378390182
	N393	-8.501104029	0.654555013	0.101083616
	N394	-7.660897066	0.321949753	2.758395056
	N395	-5.473391574	2.064493103	3.006756507
	N396	-3.351096378	2.714328198	-0.625374854
	N397	-2.643476474	0.697900930	-0.352100287
	N398	-6.074111254	4.678947199	3.738742581
	N399	-9.633059745	7.704690977	2.911743001
	N400	-9.330025759	9.479266467	4.209013075
	N401	9.660673988	2.580370294	1.512982702
	N402	6.200406676	7.211671630	-0.849693237
	N403	3.823537423	8.471663532	-3.117438626
	N404	1.133424973	7.873552696	-3.290012652
	N405	-3.703249757	6.465897944	-3.892336116
	N406	-4.061806506	8.343503574	-5.248317955
	N407	-4.894325063	8.290711909	-3.091901396
	N408	0.467968750	6.343861272	-0.936837719
	N409	1.024546363	8.220034221	1.061531429
	N410	-0.348781272	10.606790890	0.945574325
	N411	-2.256895071	10.331122898	-2.532769517
	N412	-2.586510728	11.621933845	2.289924603
	N413	2.897604191	-4.485305957	-10.594563901
	N414	1.506551820	-2.415105592	-7.977852477

N415	0.530774845	0.362713898	-7.276950541
N416	2.602506020	2.356934265	-7.487034468
N417	-2.892177745	-2.771402188	-9.755735889
N418	-4.280600355	-1.007351931	-7.978935179
N419	7.307279126	-6.897049413	-6.987544627
N420	3.647192896	-5.580074359	-4.851708895
N421	2.840094779	-6.435962435	-6.715538988
N422	8.730625405	-7.372562490	-4.202624441
N423	-0.102331853	2.401699849	-0.203030290
N424	0.642497776	1.759352650	-0.763912154
O425	-6.466001273	-5.282788957	2.309895671
O426	-6.502945773	-2.870144546	5.114760865
O427	-4.343871922	-5.082589495	7.115854682
O428	-1.659706187	-3.994453719	4.867384472
O429	-2.051677269	-0.559174930	5.408457820
O430	-1.809231743	-0.973021788	8.965831434
O431	0.742969276	-4.079903478	9.432769683
O432	2.998805009	-3.716574708	5.748481129
O433	3.109423349	-0.006313213	5.431327609
O434	2.714451545	1.430000325	8.901309942
O435	3.409189617	-0.554227322	11.696686961
O436	7.250535239	-10.392856173	1.146846379
O437	8.480775246	-7.767369634	4.707678402
O438	12.046689904	-4.750987664	3.174169221
O439	-5.709678239	-0.900665612	0.763453765
O440	-4.249144152	-4.326670840	-3.593109092
O441	-7.227200829	2.497728857	0.540444586
O442	-6.734543350	3.091156596	-2.453270952
O443	-9.521600014	1.441800162	3.502373575
O444	-5.110453865	1.383319023	5.157757303
O445	-3.915813434	5.009463208	4.408483229
O446	-6.099387560	7.115743347	6.311868370
O447	11.139908609	-0.196488603	3.072677504
O448	3.912080066	4.557501120	3.652236318
O449	3.463882924	8.371840435	-0.869738733
O450	0.550575919	10.095829357	-3.340032487
O451	-1.630607909	7.218552047	-1.183207300
O452	-0.536987501	7.208432639	2.404940456
O453	2.470952663	5.677626848	1.386841899
O454	1.087511401	11.594628115	2.435283996
O455	-2.493025148	13.579737118	1.125403129
O456	-3.467527830	11.036646312	-0.728046616
O457	-4.559412514	13.617561092	4.545329146
O458	-5.687760614	6.877882728	-0.503962741
O459	0.713410374	-4.367759851	-8.889622704
O460	-1.267451796	-0.575590954	-8.343983228
O461	1.111001735	3.775229715	-8.456846208
O462	4.823571026	4.466299108	-9.398355593
O463	-6.016457594	-2.477815047	-8.133877204
O464	-2.196085566	-6.517496552	-5.873427133
O465	-0.383020042	-5.652933409	-6.844205881
O466	-4.929685916	2.487620557	-7.750826527
O467	9.382118815	-5.320895961	-4.911366453
O468	12.232365632	-6.845905422	-3.661293655
O469	0.032585446	-6.369423465	-1.458170807
O470	-0.279989082	-7.475346839	0.475998567
O471	4.636185286	-10.900749488	-4.110693199
O472	2.382353978	-10.944845226	-3.920139213
O473	4.816811647	-5.952165853	-2.025471343
O474	4.892772907	-7.665159324	-0.574842696
O475	2.255419105	-6.138296001	-2.454198415
O476	8.958967731	-6.616488119	-0.082250524
O477	6.947013347	-4.676092175	-0.595531097
O478	-4.702016803	4.802143655	-1.783226792
O479	7.287973872	-10.389010484	-3.553871116
O480	7.378586808	-8.671017246	-1.061359354
S481	3.200027020	4.941689977	-2.522662557
S482	1.642251822	1.994859611	-3.533851829
S483	1.523696395	-3.314937260	-3.579900268
S484	3.507687392	1.662011649	-0.403657203
S485	0.237153623	-0.966225175	-1.671778616
S486	3.318848163	-0.706021243	-5.781104513
S487	3.422380863	-3.695818948	-0.578799790
S488	5.228252192	1.739518122	-3.538775737
S489	5.216773612	-3.057247614	-3.652535290
S490	6.333248755	-0.938491176	-1.078197404
end			

S2BH-6Hx-7Hn-6H2n transverse (Scheme 3) intermediate 8

Fe(139)	-2.423
Fe(140)	-0.968
Fe(141)	-2.542
Fe(142)	2.741
Fe(143)	2.759
Fe(144)	1.230
Fe(145)	0.114

S=1/2

bm52etc6hx7hnexpro6h2ndtd.car_3

C1	-8.585609628	-1.568121366	3.744055622
C2	-9.221931305	-2.956900736	3.858411030
C3	-8.973139446	0.790283704	3.186034138
C4	-8.341880809	1.484296523	4.388278886
C5	-8.160034668	1.632412957	6.822842538
C6	-6.679909301	1.306352842	7.042088085
C7	-4.799072604	-0.167837656	6.545321455
C8	-4.016738485	0.745882502	5.593690138
C9	-4.069519193	1.884536174	3.412003346
C10	-3.930674939	3.314270428	3.956761086
C11	-4.869466271	5.131925918	5.239293294
C12	-3.914352445	5.286046464	6.433139123
C13	-3.305798157	4.449062790	8.661663729
C14	-1.936462408	3.780660348	8.848042993
C15	-0.554535041	1.788375436	8.405734166
C16	0.336338093	1.936941450	7.167629658
C17	0.428589907	2.761336964	4.842287679
C18	0.813721549	4.244059241	4.677142167
C19	-0.352258984	2.123430100	3.665981201
C20	0.159980328	2.537517826	2.273262837
C21	-0.376146322	0.595756568	3.808119856
C22	0.116375388	6.542347485	4.180078870
C23	0.462387411	7.318085966	5.466823258
C24	-1.025736542	7.197852564	3.364770849
C25	-0.820323220	8.711404088	3.231415574
C26	-1.135965437	6.548836059	1.978081924
C27	-0.145167679	7.932687687	7.733148867
C28	1.249119441	7.733238426	8.293038108
C29	3.643543331	-6.105821806	11.528681233
C30	4.480763496	-5.732182200	10.311289203
C31	5.229273741	-3.769202311	8.971313833
C32	6.521064568	-3.065623325	9.428351429
C33	4.247614376	-2.778728383	8.324707633
C34	4.831433165	-2.101248124	7.088988908
C35	3.774430736	-1.337787945	6.294363678
C36	4.453712690	0.351268766	4.559464511
C37	8.922303113	-2.805484099	9.010255857
C38	9.426335164	-1.833891639	7.966639793
C39	-5.778822897	-2.131291175	0.147058622
C40	-5.855315630	-0.784852630	-0.597214204
C41	-4.297142147	-2.421564610	0.476157065
C42	-4.048248441	-3.567348642	1.478435096
C43	-3.899857747	-4.957119946	0.859075187
C44	-5.918672829	0.172347771	-2.873486769
C45	-6.879677070	1.347237326	-2.642515762
C46	-6.009533644	-0.387242504	-4.300237812
C47	-9.071622408	2.066033929	-1.815704371
C48	-8.642531097	3.075844283	-0.745623674
C49	-7.022901668	3.673834978	0.994037384
C50	-5.679810256	4.294545776	0.609227219
C51	-3.868821967	4.524468214	-1.014649742
C52	-4.030952154	5.981730231	-1.505121992
C53	-3.236944540	3.768433783	-2.192979483
C54	-2.870106525	2.341673950	-1.962553673
C55	-2.733390742	1.558422282	-0.845177527
C56	-2.264695939	0.292368710	-2.619202985
C57	-5.470686064	7.471682493	-2.835122398
C58	-5.306509912	8.656799068	-1.885753077
C59	-6.918461436	7.403714908	-3.376372604
C60	-7.269317672	8.345126559	-4.490675579
C61	-6.966985413	9.679777990	-4.682994161
C62	-8.227591919	8.928502200	-6.342693271
C63	9.479676016	2.168757163	2.335924391
C64	10.260071533	1.134810559	3.126378394
C65	7.981259194	1.806171688	2.494376793
C66	7.054324302	2.791728958	1.821819930
C67	6.572638706	3.925727597	2.503786568
C68	6.644920540	2.604200083	0.488420157
C69	5.705769901	4.836133766	1.887556490
C70	5.793491322	3.511285544	-0.145241369
C71	5.320539839	4.624852450	0.557029509
C72	6.553117845	2.867101489	-5.165420629
C73	5.724169615	3.623978182	-6.218896505
C74	5.643276444	2.298366289	-4.072786789
C75	5.005247273	3.707734958	-8.581650750
C76	3.482775378	3.552456550	-8.602791652
C77	1.534929872	2.536869948	-7.427958488

C78	1.040031322	3.398368444	-6.237388594
C79	1.305596260	1.026662928	-7.192301113
C80	-0.092143986	0.538807169	-6.794997760
C81	-1.139987014	0.452079723	-7.916670833
C82	-2.255563391	2.334897687	-9.120897999
C83	1.418001648	4.250769278	-3.974798942
C84	1.526967394	5.764091797	-4.216130543
C85	2.217862284	3.915321400	-2.698891985
C86	3.011047470	7.534343649	-5.025972055
C87	2.624699566	8.331981307	-6.262569731
C88	1.450143914	8.311047986	-8.399539180
C89	0.326769356	9.374976109	-8.469873212
C90	1.257885001	7.249983127	-9.484158310
C91	-0.044656869	6.469934991	-9.366193696
C92	-1.637170988	10.351200092	-7.377166190
C93	-1.314590897	11.839056871	-7.420853091
C94	-2.507685829	10.026297358	-6.133749334
C95	-2.977403900	8.589046228	-6.130591455
C96	-4.158850110	8.227396678	-6.798492864
C97	-2.228854654	7.571299273	-5.517577729
C98	-4.566986421	6.894196335	-6.874365889
C99	-2.606968754	6.227200944	-5.606504110
C100	-3.772359816	5.904433934	-6.295964448
C101	3.021768482	-9.771431866	-2.974496462
C102	1.683191541	-9.055273994	-3.003501650
C103	0.577746905	-6.858099246	-3.344671309
C104	0.348837500	-6.219706345	-4.719541034
C105	1.222934059	-4.601530993	-6.343830423
C106	2.548395318	-4.264380070	-7.020028300
C107	4.932751929	-3.807266336	-6.875436843
C108	5.500076075	-4.781602546	-7.887500723
C109	-3.596845264	-8.509782962	-4.260648520
C110	-4.384923503	-7.371747884	-4.919540980
C111	-3.229354614	-8.118841963	-2.811252194
C112	-2.778947033	-9.344277663	-1.993668226
C113	-2.175684952	-8.909747724	-0.674701232
C114	-4.320460263	-5.257816035	-6.142084571
C115	-3.622983007	-4.924960037	-7.445231576
C116	-4.632598807	-4.036168096	-5.242414353
C117	-3.398956496	-3.602365597	-4.500993387
C118	-2.453937258	-2.758346539	-5.108946552
C119	-3.096983770	-4.182300162	-3.255328465
C120	-1.198713643	-2.574561980	-4.522188653
C121	-1.843097851	-3.999096595	-2.671188892
C122	-0.878914161	-3.226255460	-3.327692520
C123	6.450007785	-8.866519341	2.029994438
C124	7.620707376	-8.418254879	2.915160224
C125	5.116721122	-8.390348073	2.624921185
C126	3.900397614	-8.582308097	1.775163669
C127	3.388399503	-9.770679189	1.298044457
C128	1.949580771	-8.140907935	0.883990109
C129	8.607002985	-8.215390721	5.118116857
C130	9.963090825	-8.700893866	4.655823204
C131	-0.745730932	-4.939313604	4.840885992
C132	0.719117651	-4.966051751	5.266643291
C133	1.559409161	-6.088339079	4.586758593
C134	1.173458346	-7.470555681	5.181223390
C135	2.096692438	-8.634321187	4.806303798
C136	1.815824528	-9.939654069	5.586744800
C137	3.049887109	-5.856603349	4.878986207
C138	3.335771060	-2.613458444	-0.625790379
Fe139	3.307004193	-4.591189902	-0.693173905
Fe140	2.143498862	-1.017645776	-1.322869490
Fe141	4.997689593	-1.515819264	-0.770545317
Fe142	3.735896267	-2.906750571	-2.537719216
Fe143	4.149821614	-0.306038435	-2.958194345
Fe144	4.216744586	-3.021982301	1.088559864
Fe145	1.622139571	-2.850173501	1.050653721
H146	-7.664337538	-3.914412717	4.763620325
H147	-7.721365526	-4.144619090	3.149858438
H148	-10.408969379	-0.787026613	3.189417804
H149	-9.876136668	-3.118032694	2.986582863
H150	-9.890590320	-2.930657984	4.736252445
H151	-8.216998309	0.790122953	2.387663321
H152	-9.414711918	0.312677221	5.678206361
H153	-9.818448908	1.403014202	2.844898509
H154	-8.739718193	1.269239936	7.681558954
H155	-6.842886148	-0.441481817	5.961823562
H156	-8.233486935	2.727968523	6.796848655
H157	-3.072881698	1.572384103	3.074259988

	H158	-5.471432172	0.461111947	4.168883623
	H159	-4.657770559	-1.216919554	6.250767092
	H160	-4.524833146	5.822598588	4.459675957
	H161	-5.838204926	3.268850809	4.745817996
	H162	-4.383508633	-0.032479219	7.551042877
	H163	-4.739104135	1.877276100	2.540006053
	H164	-4.642051498	3.545868659	7.259194367
	H165	-5.873465735	5.439828564	5.560306113
	H166	-0.780559835	0.275760267	4.778549766
	H167	-1.004849804	0.138862994	3.025550593
	H168	0.638211491	0.178358873	3.698352490
	H169	-1.377114306	2.502272335	3.758340244
	H170	-3.981982198	4.046357557	9.430241074
	H171	-1.330011996	5.468915102	2.030758727
	H172	-1.250837213	6.643183927	6.437923595
	H173	-0.817427199	9.216236868	4.208392393
	H174	0.857848530	1.795910241	1.853506046
	H175	0.668097356	3.512419856	2.273979270
	H176	9.643226526	3.151302726	2.819524117
	H177	-1.955360718	7.005664477	1.406141096
	H178	-1.631466718	9.142796845	2.627091912
	H179	-0.202286534	6.705258050	1.413834421
	H180	0.135018004	8.934637779	2.730937716
	H181	-1.968526561	7.026590398	3.914937631
	H182	1.042249355	6.577202280	3.586125284
	H183	-1.138999401	4.796467107	4.279221349
	H184	-0.688618767	2.629375364	1.575106917
	H185	2.331478963	2.822773033	-2.629995446
	H186	1.616088612	4.260268709	-1.845503629
	H187	3.682141109	4.889266835	-1.806367750
	H188	4.168851710	6.186983614	0.488919067
	H189	5.471544757	3.344224987	-1.174241318
	H190	6.972160942	1.716490680	-0.058438801
	H191	6.864098189	4.096380722	3.544339328
	H192	-1.257669736	2.705059191	6.147832930
	H193	-0.755067343	0.716585477	8.545754070
	H194	0.027652015	2.139989175	9.267046009
	H195	-2.560775662	2.112848499	7.786183418
	H196	-3.155803824	5.516587822	8.857651863
	H197	5.325508706	5.697851103	2.442581846
	H198	1.398595856	2.256681730	4.928559030
	H199	-0.325455527	9.010188117	7.586275067
	H200	-0.849670369	7.575210045	8.500071225
	H201	1.738216803	6.772836116	8.007146026
	H202	9.672779884	-3.595480776	9.163511524
	H203	-2.977738068	-6.761223942	1.140382999
	H204	-0.000920731	-5.585463011	3.184500261
	H205	-3.651052448	-9.979375228	-1.780064517
	H206	-2.051126844	-9.938488282	-2.565733527
	H207	-4.095613930	-7.653572629	-2.315364853
	H208	-3.111356344	-5.791453257	-7.927808317
	H209	-4.326465879	-9.332031638	-4.204996751
	H210	-3.767993752	-2.632406775	-0.468489439
	H211	-4.878850946	-3.617478456	2.204037311
	H212	-2.631624298	-6.383806578	-5.434942593
	H213	-5.294374116	-5.675053063	-6.470465907
	H214	-1.700932890	-8.322680164	-5.088773319
	H215	-2.501490122	-5.526664803	2.301158626
	H216	-2.435611613	-7.355441918	-2.834588598
	H217	-2.160532617	-9.875005046	-4.789679057
	H218	-7.339467575	-3.380566536	-0.378874089
	H219	-3.873454565	-1.502442194	0.910449815
	H220	-0.395793578	-9.164024930	-1.469808570
	H221	-3.149827741	-3.340778843	2.071438334
	H222	-3.180730252	4.565755386	-0.158530112
	H223	-2.865958798	1.785468219	0.204124635
	H224	-7.738885911	4.500063029	1.112138535
	H225	-7.112473646	1.832333216	-0.084050689
	H226	-5.945827481	5.525682604	-2.083863967
	H227	-6.365506055	10.382616107	-4.117533441
	H228	-5.602887166	8.436971251	-0.831215505
	H229	-7.622186567	7.516475103	-2.532098850
	H230	-4.757467396	7.585709516	-3.667165880
	H231	-8.798280151	8.937701632	-7.267957506
	H232	-2.328635038	4.320500618	-2.483593388
	H233	-3.924417715	3.810908576	-3.054192491
	H234	-7.548053049	10.945465940	-6.305354565
	H235	-7.072599834	6.386879015	-3.769972575
	H236	-5.634952582	3.306508712	-1.226529986
	H237	-6.906316143	3.210709680	1.986181445

	H238	-9.322294293	2.653518098	-2.709591264
	H239	-1.977412451	-0.562583424	-3.228323274
	H240	-9.992871506	1.575123938	-1.475995603
	H241	10.809161689	2.539795611	0.822352484
	H242	9.246401111	2.676197035	0.368255498
	H243	2.633144655	2.801902958	-5.018571780
	H244	0.355283816	4.051828114	-3.773124181
	H245	3.263676623	5.449865977	-5.262902582
	H246	-2.708811016	1.805478464	-4.050781567
	H247	-4.265454584	1.616212603	-5.502374148
	H248	-6.034691722	0.362330475	-6.111543811
	H249	-3.706403936	3.094509745	-5.696013383
	H250	-5.293557092	-1.221223871	-4.392260100
	H251	-7.024945100	-0.770366174	-4.485321641
	H252	2.129654626	6.581453514	-9.507686409
	H253	1.225687896	7.777509907	-10.450307237
	H254	0.979968577	4.687378749	-9.615787085
	H255	-0.768213427	4.590773480	-9.412885732
	H256	-1.980760371	2.507081362	-11.148547600
	H257	-1.801329976	-0.411254018	-7.747592915
	H258	-5.497629194	6.627621759	-7.381985297
	H259	-1.330208866	7.810976270	-4.943443561
	H260	-3.164603524	3.891050359	-8.118986419
	H261	1.994602301	0.712339247	-6.392297536
	H262	3.610849995	2.247333385	-7.041990614
	H263	-0.486769548	1.128659206	-5.951904517
	H264	-8.287961929	0.068958883	-1.897492244
	H265	5.396745969	3.293624173	-9.520904789
	H266	-6.227968896	-1.908099365	-2.199165127
	H267	1.631056270	0.498821548	-8.103300986
	H268	-1.972573556	5.451584886	-5.175425976
	H269	5.204983340	4.789852427	-8.578559354
	H270	-1.532848509	0.964682734	-10.488310994
	H271	-3.383868813	3.884351538	-9.835861554
	H272	-5.822282560	-4.066499944	-0.557165088
	H273	-6.305129494	-1.967956731	1.104873699
	H274	-4.778126178	8.995622192	-7.267453466
	H275	-4.924147617	0.619448767	-2.735138077
	H276	0.982747129	2.892641240	-8.302718046
	H277	-0.632037905	0.282751577	-8.874286987
	H278	0.046280027	-0.482729249	-6.415058934
	H279	-2.468552501	1.924140784	-7.140731970
	H280	2.603396984	8.059588674	-4.149986129
	H281	1.430888181	6.788058735	-6.877299015
	H282	2.351075374	8.885799690	-8.662999807
	H283	4.106895818	7.563926931	-4.957514371
	H284	-0.295894484	8.871027934	-6.606394350
	H285	3.706444917	-9.303637848	-3.697035872
	H286	3.442908945	-9.581925848	-1.964978755
	H287	6.612673051	-8.367633947	1.063596460
	H288	3.714093733	-11.685370698	-3.061554832
	H289	2.119431480	-11.579615302	-2.706289263
	H290	6.279200372	1.773460860	-3.347414635
	H291	7.068403649	4.584419482	-4.188661571
	H292	6.209322786	2.217125854	-7.638915538
	H293	5.117522390	3.119723609	-3.567061431
	H294	8.218887155	4.069451340	-5.233527454
	H295	5.670025906	-3.696000591	-6.064234981
	H296	7.093231190	2.040119825	-5.652346065
	H297	3.944658982	-2.029859939	9.074846870
	H298	5.470002513	-4.562617255	8.252075616
	H299	3.682509113	-4.480356547	-5.281565155
	H300	5.113210145	1.484996709	2.980250668
	H301	0.645898254	-5.218670735	-7.042059245
	H302	-1.613001459	-4.466583017	-1.710749132
	H303	0.680677716	-3.646623170	-6.241547384
	H304	3.403900716	-0.477277891	6.864035323
	H305	1.939035004	-5.002477200	-4.334843271
	H306	2.908833375	-1.986392155	6.084297888
	H307	3.351314958	-3.354253874	8.044206102
	H308	5.273412883	-2.861830353	6.427862836
	H309	5.641076440	-1.405607834	7.362866100
	H310	-5.426605340	-4.334582605	-4.542646550
	H311	4.127027001	-3.832616023	10.823555847
	H312	2.695451430	-6.502470705	11.133515000
	H313	0.118417917	-3.101270805	-2.911646669
	H314	4.096416970	-4.865482055	13.109845762
	H315	2.490944154	-5.132501628	12.926916054
	H316	4.787405642	-1.637932666	4.471397742
	H317	-0.443931875	-1.940269702	-4.992864872

	H318	-3.842890000	-4.800908904	-2.749694608
	H319	5.524118656	-9.121519327	6.781046127
	H320	4.941994763	-8.921140946	3.577509845
	H321	4.928434044	-6.659306017	6.504122414
	H322	4.524673255	-10.217265555	6.314083924
	H323	5.112627862	-5.825389596	-7.803521664
	H324	5.278965675	-4.598944904	4.184909870
	H325	0.778582737	-5.035132460	6.359425627
	H326	2.641547303	-7.303713537	-3.462331959
	H327	1.174876225	-7.350487546	6.275548956
	H328	1.497533651	-10.127780569	0.355794634
	H329	1.037716002	-7.653783728	0.562959825
	H330	4.156576579	-6.947639613	12.023650403
	H331	5.529768905	-0.198816461	2.866858601
	H332	7.117531684	-4.649663536	5.663894489
	H333	6.345895953	-3.759098188	3.436616062
	H334	7.552634080	-4.023859222	7.887697614
	H335	3.195124529	1.278702992	5.942862038
	H336	1.978143841	-8.862739001	3.736404552
	H337	3.149527469	-8.358737908	4.963658574
	H338	0.138658421	-7.704328407	4.888372969
	H339	0.732560132	-6.077971695	-2.582509057
	H340	-2.692721627	-2.292919460	-6.068346687
	H341	-5.022286414	-3.239101053	-5.893849557
	H342	1.133717080	-3.987884143	4.968930640
	H343	6.953608865	-5.865119671	6.621890179
	H344	5.507073389	-6.945272273	7.904428809
	H345	3.991365425	2.328758874	4.829292405
	H346	3.781816382	-10.778082035	1.341486449
	H347	5.201834485	-7.329017855	2.886184104
	H348	-0.307462392	-7.444307452	-3.088829122
	H349	4.805545231	-2.822863087	-7.351546548
	H350	7.765997327	1.763215634	3.572329329
	H351	7.823770037	0.799469861	2.074151907
	H352	7.350391548	-10.577223515	1.351074887
	H353	6.416449128	-10.827577041	2.670755701
	H354	8.430194170	-8.642736261	6.118472925
	H355	6.727532687	-9.122784708	4.681545950
	H356	9.949990062	-9.663578996	4.090693441
	H357	8.622917942	-7.118219338	5.220985440
	H358	10.483766652	0.197909919	2.559469201
	H359	-0.281892068	12.114033434	-7.099416930
	H360	8.821560351	-2.244938793	9.956809383
	H361	8.630919685	-1.324798100	7.370125924
	H362	-2.214580409	10.117348830	-8.286835322
	H363	-1.923974496	10.246193333	-5.226009736
	H364	-3.368616749	10.709726625	-6.149242469
	H365	0.441587582	-3.081325014	1.971512121
	H366	4.175034550	-1.597193336	1.541477955
	H367	-5.058368722	4.494317992	-6.700031394
	H368	-0.189484208	-0.973324207	0.164526428
	H369	1.962111210	-1.261852928	1.230317039
	H370	1.339096573	-1.460016220	1.798462676
	H371	-2.144408115	-0.502909024	-0.698156468
	Mo372	2.877231989	-5.246954028	1.923732855
	N373	-8.307736606	-4.077520514	3.985121974
	N374	-9.451969980	-0.556759238	3.444411332
	N375	-8.724906630	1.057538841	5.614051698
	N376	-6.226667373	0.127305988	6.544511889
	N377	-4.599764577	0.940956622	4.382883682
	N378	-4.952903002	3.781936276	4.708876338
	N379	-3.953823486	4.297025782	7.364476120
	N380	-1.810997184	2.515511380	8.353354648
	N381	-0.258808538	2.518223149	6.099601724
	N382	-0.187812912	5.134013772	4.448098066
	N383	-0.371268936	7.159113486	6.525340995
	N384	3.356284402	-4.966124367	12.411195712
	N385	4.590893419	-4.405282301	10.110388245
	N386	4.339227347	-0.898981209	5.017783207
	N387	3.915214842	1.395057828	5.223445375
	N388	5.142565555	0.572042453	3.424923361
	N389	7.623311502	-3.367003368	8.696274818
	N390	-6.375929345	-3.196885123	-0.665964501
	N391	-3.087536774	-5.810382679	1.509312859
	N392	-6.092145400	-0.918622470	-1.928696050
	N393	-8.095625063	1.039034289	-2.137395224
	N394	-7.587708105	2.731468883	0.046664481
	N395	-5.124395295	3.920774018	-0.585081472
	N396	-2.572582454	1.524109133	-3.045091227
	N397	-2.369625215	0.300563839	-1.284260990

	N398	-5.218583166	6.235862902	-2.109834158
	N399	-8.058248373	7.888060044	-5.534644969
	N400	-7.581685053	10.029158454	-5.866219731
	N401	9.886974205	2.109585111	0.930879702
	N402	7.547745019	3.745523570	-4.531620300
	N403	5.691520063	3.075400207	-7.468951092
	N404	2.960280144	2.722269723	-7.677037561
	N405	-1.977467831	1.651714981	-8.011114180
	N406	-1.887747101	1.902108322	-10.339207716
	N407	-2.867208186	3.538639539	-9.031629565
	N408	1.832677311	3.449623254	-5.128704879
	N409	2.564473815	6.153872118	-5.009773927
	N410	1.696886940	7.750729774	-7.083675674
	N411	0.057936771	5.140639600	-9.640719170
	N412	-0.420472287	9.551112685	-7.352153695
	N413	2.853825366	-11.189246042	-3.304131059
	N414	1.726983696	-7.753479857	-3.355850568
	N415	1.271456960	-5.295373786	-5.068396394
	N416	3.688251909	-4.271892025	-6.283570995
	N417	-2.498101716	-8.968631780	-5.118337884
	N418	-3.654995615	-6.362693135	-5.476408522
	N419	6.457271702	-10.320174069	1.781186915
	N420	2.987574288	-7.566506136	1.494108975
	N421	2.166524023	-9.467142170	0.740683000
	N422	7.539893780	-8.677789234	4.247951658
	N423	0.487541903	1.384792000	-1.700782432
	N424	1.121480034	0.458765172	-1.562928349
	O425	-7.378184644	-1.350804015	3.950219798
	O426	-7.522450622	2.404865735	4.219571148
	O427	-5.951194070	2.101198553	7.659314394
	O428	-2.941287022	1.280359233	5.916268578
	O429	-2.917307047	3.989400916	3.720820548
	O430	-3.199678207	6.297228735	6.539721301
	O431	-1.042019684	4.360910817	9.474883305
	O432	1.510512489	1.520221598	7.187981589
	O433	2.006082901	4.580938708	4.758096222
	O434	1.455564203	8.062627761	5.523673608
	O435	1.782197524	8.525265184	9.056024118
	O436	4.967643399	-6.626538259	9.589957841
	O437	6.515440079	-2.282021537	10.393704597
	O438	10.609765869	-1.597338265	7.780650550
	O439	-5.692364514	0.315169412	-0.033100438
	O440	-4.521239692	-5.287651230	-0.177164652
	O441	-6.522942071	2.510840591	-2.898836068
	O442	-5.698880001	0.653753223	-5.245635642
	O443	-9.266382156	4.139495657	-0.635872805
	O444	-5.132703992	5.096210964	1.373048600
	O445	-3.108274063	6.798793296	-1.440617499
	O446	-4.933895341	9.765438821	-2.228296633
	O447	10.596272026	1.272714773	4.294049272
	O448	4.458201452	5.474104559	-0.109895903
	O449	5.129972634	4.683763754	-5.953198512
	O450	2.815592639	4.172317108	-9.454122729
	O451	-0.038642576	4.008240906	-6.319557390
	O452	0.747480819	6.551328526	-3.664130965
	O453	3.499793244	4.546422678	-2.704465218
	O454	3.123142505	9.441357867	-6.485803600
	O455	0.177384883	10.059051929	-9.492618975
	O456	-1.121076488	7.021750978	-9.095886974
	O457	-2.129683199	12.681834567	-7.761401193
	O458	-4.120398550	4.544821166	-6.431662607
	O459	0.627334349	-9.653367121	-2.682315153
	O460	-0.605251959	-6.552819588	-5.452287263
	O461	2.540481962	-3.934881040	-8.217047639
	O462	6.339726619	-4.472763007	-8.717290831
	O463	-5.627320868	-7.362827596	-4.922056015
	O464	-2.847678307	-8.567414647	0.292018759
	O465	-0.828907530	-8.856440137	-0.613743532
	O466	-3.651088693	-3.826721791	-7.983613394
	O467	8.632846133	-7.871019969	2.435518986
	O468	11.013900665	-8.140037366	4.936402796
	O469	-0.964847482	-5.266081317	3.561007148
	O470	-1.657462672	-4.592740118	5.590063074
	O471	2.831788231	-10.593018995	6.013313067
	O472	0.608515734	-10.286532008	5.720632771
	O473	3.838250893	-5.739829323	3.839884739
	O474	3.463349873	-5.820682309	6.052792804
	O475	1.325826883	-6.016776526	3.180708196
	O476	7.544131616	-5.081190235	6.444436111
	O477	5.985898650	-3.919604788	4.330388354

	O478 -3.429850278 2.150310844 -5.591208083<br>O479 5.487930503 -10.077978653 6.556517696<br>O480 5.709776526 -7.088898787 6.945645130<br>S481 4.365004955 1.128182688 -4.726536793<br>S482 2.285219697 -1.482361906 -3.524414187<br>S483 1.281482517 -5.000171505 0.229067364<br>S484 3.890731456 0.463762812 -0.881131560<br>S485 0.216416757 -2.070535484 -0.528584789<br>S486 3.703925552 -5.119985032 -2.801511086<br>S487 2.902647027 -3.119661396 2.858325370<br>S488 5.780848731 -1.908538641 -2.800233241<br>S489 4.929607265 -5.152625984 0.727894907<br>S490 6.238910347 -1.928264180 0.987634488<br>end

TS for 6H2n dissociation (Scheme 3)

Fe(139) -2.208<br>Fe(140) -1.043<br>Fe(141) -2.653<br>Fe(142) 2.652<br>Fe(143) 2.795<br>Fe(144) 1.490<br>Fe(145) -0.089<br><br>**S=1/2**	bm52etc6hx7hnexpro6h2ndtc_1_53138.97

S2BH-6Hx-7Hn-6H2n (8) to S2BH-6H2x-7Hn-6Hn (9) (Scheme 3)

TS

Fe(139) -2.452<br>Fe(140) -1.141<br>Fe(141) -2.557<br>Fe(142) 2.715<br>Fe(143) 2.790<br>Fe(144) 1.495<br>Fe(145) 0.110<br><br>**S=1/2**	2n2x2bh7hn6hx6h2nxcc.car_1<br>C1 -7.819266158 -5.384432283 -0.367390969<br>C2 -7.966888406 -6.483409337 -1.424276431<br>C3 -8.923627000 -3.530021984 0.803616660<br>C4 -8.556414831 -3.862622651 2.247260860<br>C5 -8.499295227 -5.549567748 4.019266094<br>C6 -6.988286005 -5.654764472 4.250840071<br>C7 -4.773408369 -5.912735832 3.257912864<br>C8 -4.230941740 -4.494890482 3.447278453<br>C9 -4.483847737 -2.123847731 2.902377762<br>C10 -4.740216824 -1.624810523 4.331290683<br>C11 -6.228992403 -1.532322440 6.238144880<br>C12 -5.389790877 -2.181311579 7.347418580<br>C13 -4.612493151 -4.278944749 8.349933389<br>C14 -3.095198151 -4.503067876 8.272474004<br>C15 -1.160983550 -4.976891521 6.821877099<br>C16 -0.308529473 -3.811204749 6.315814613<br>C17 -0.333942215 -1.523678406 5.394635596<br>C18 -0.418191125 -0.352737434 6.385369338<br>C19 -0.767683507 -1.170352183 3.946835967<br>C20 -0.065030627 0.102893634 3.460217899<br>C21 -0.487003177 -2.339631403 2.996461787<br>C22 -1.828180522 1.287606788 7.538625737<br>C23 -1.758326095 0.853170790 9.015020936<br>C24 -3.108460882 2.082940045 7.190451186<br>C25 -3.356601559 3.195434173 8.214962908<br>C26 -3.003012236 2.665779562 5.774957177<br>C27 -2.514052939 -0.644719470 10.766443094<br>C28 -1.121498181 -0.884422894 11.314961533<br>C29 5.074996271 -11.841094633 4.162435410<br>C30 5.828081531 -10.553550518 3.838757807<br>C31 6.025964570 -8.166242898 4.515852795<br>C32 7.090332155 -7.849291417 5.584245386<br>C33 4.838066013 -7.191950526 4.610619109<br>C34 5.244834875 -5.743255479 4.349020631<br>C35 4.039622579 -4.817397551 4.193755556<br>C36 4.142583574 -2.307613751 4.196233678<br>C37 9.291153067 -6.837187865 5.977686078<br>C38 9.435931384 -5.334686707 6.087768171<br>C39 -4.951731394 -2.506283717 -2.715996579

	C40	-5.387595424	-1.092703737	-2.285821826
	C41	-3.459068431	-2.676774540	-2.348600302
	C42	-2.904962060	-4.114857010	-2.404520281
	C43	-2.369774222	-4.566206553	-3.763668624
	C44	-5.670739686	1.199788025	-3.147340538
	C45	-6.913499722	1.632364471	-2.358193520
	C46	-5.572977153	1.876433652	-4.521076187
	C47	-9.228120072	1.110533302	-1.746334099
	C48	-9.102752068	1.005160764	-0.222407455
	C49	-7.718291314	0.340191640	1.688393568
	C50	-6.585414581	1.246185913	2.172557938
	C51	-4.962896156	3.008114547	1.674129584
	C52	-5.520954835	4.285430455	2.345152002
	C53	-4.114944982	3.506548753	0.492995589
	C54	-3.358928339	2.478161715	-0.280326245
	C55	-2.943673928	1.197488115	-0.011909957
	C56	-2.259184935	1.714106096	-2.068293931
	C57	-7.309208763	5.975826217	2.241573040
	C58	-7.436535022	6.073166047	3.760517962
	C59	-8.696730651	6.073750849	1.562931021
	C60	-9.271185645	7.451600660	1.404861930
	C61	-9.350732223	8.505129601	2.294549763
	C62	-10.288666881	9.053927235	0.363420027
	C63	8.505955577	1.470512409	5.096845187
	C64	9.572121851	0.392710208	5.019044185
	C65	7.192422398	0.836732380	4.573309289
	C66	6.012687825	1.778099606	4.626128876
	C67	5.214161863	1.886303909	5.779230796
	C68	5.668107978	2.561964744	3.512597651
	C69	4.099982991	2.731867349	5.815821984
	C70	4.568013702	3.420604652	3.538358718
	C71	3.778431736	3.496601758	4.688449634
	C72	5.525816578	6.897084279	-0.102259734
	C73	4.510540748	8.005184920	-0.425543686
	C74	4.812827552	5.554842454	0.060242521
	C75	3.789989537	9.682213058	-2.092242720
	C76	2.383774084	9.308498345	-2.566154487
	C77	0.801819730	7.411724783	-2.913693476
	C78	0.057195870	6.976121126	-1.626074058
	C79	1.036855140	6.232657410	-3.887179162
	C80	-0.149893514	5.352940809	-4.297419823
	C81	-1.132294454	5.943389519	-5.319461162
	C82	-2.716494561	7.845269134	-5.038195879
	C83	0.119691742	5.913015299	0.572625494
	C84	-0.203550645	7.095026581	1.497431168
	C85	0.941269007	4.899525208	1.392718741
	C86	0.758519940	9.075462385	2.562833478
	C87	0.201632018	10.454174681	2.245271097
	C88	-0.856569746	11.875452524	0.567548753
	C89	-2.223326219	12.417130804	1.052644204
	C90	-0.729869712	11.986589927	-0.954909164
	C91	-1.772076457	11.189603719	-1.731932903
	C92	-4.408655730	11.879919572	2.026869147
	C93	-4.506153834	12.910799415	3.141646535
	C94	-5.169145913	10.586164066	2.435872024
	C95	-5.208060356	9.572316308	1.314073641
	C96	-6.214791112	9.638536163	0.338007711
	C97	-4.214009089	8.590776066	1.171167853
	C98	-6.215741636	8.778943687	-0.762499590
	C99	-4.185082225	7.737044566	0.063736765
	C100	-5.185920813	7.846897080	-0.896632290
	C101	5.675058581	-3.625225802	-8.235958929
	C102	4.188946577	-3.391365961	-8.031834275
	C103	2.523464420	-1.908106272	-6.933986681
	C104	2.137239726	-0.523738982	-7.465577259
	C105	2.547367140	1.889494348	-7.237788724
	C106	3.768375692	2.803415742	-7.211258328
	C107	5.968776425	3.358164782	-6.357757828
	C108	6.604164032	3.749984101	-7.675576442
	C109	-0.972744329	-3.068441989	-9.667334402
	C110	-2.033302525	-1.974201393	-9.503639622
	C111	-0.769755736	-3.804680571	-8.322277588
	C112	-0.029275574	-5.141055068	-8.522069807
	C113	0.404440357	-5.714408041	-7.190790290
	C114	-2.542513450	0.321368450	-8.843613512
	C115	-1.949261321	1.619768526	-9.353528563
	C116	-3.198667720	0.412997878	-7.443847524
	C117	-2.167239540	0.366891857	-6.351169718
	C118	-1.478113346	1.527750700	-5.962344504
	C119	-1.769400948	-0.870205615	-5.813813444

	C120	-0.372355996	1.440710169	-5.111083151
	C121	-0.663178745	-0.956177142	-4.965184300
	C122	0.057906610	0.197079250	-4.640085289
	C123	8.638125475	-6.161743545	-3.561724078
	C124	9.623485939	-6.263484233	-2.385903264
	C125	7.209980840	-6.394644262	-3.045960942
	C126	6.100065937	-6.152341982	-4.017714904
	C127	5.946565485	-6.684518959	-5.281129419
	C128	4.095587786	-5.610270132	-4.714368644
	C129	10.509476634	-7.529533633	-0.522152420
	C130	11.945776000	-7.189182923	-0.854300549
	C131	0.610579637	-6.952539485	-0.334538038
	C132	2.024273165	-7.029463035	0.234799367
	C133	3.149796990	-7.097285030	-0.838982978
	C134	3.174133571	-8.504952896	-1.497058512
	C135	4.397113113	-8.799139196	-2.371032232
	C136	4.501181668	-10.267973339	-2.839546360
	C137	4.511176169	-6.873417279	-0.164068786
	C138	3.933569295	-0.611023267	-1.547655835
	Fe139	4.434864475	-1.902401033	-2.945009784
	Fe140	2.409732571	0.803229139	-1.259391653
	Fe141	5.221774922	0.490001605	-0.484425705
	Fe142	4.550989652	0.613587743	-2.973556541
	Fe143	4.221780581	2.762996318	-1.383247351
	Fe144	4.829278053	-1.982025394	-0.425680864
	Fe145	2.270803460	-2.444510287	-0.929304947
	H146	-6.173972106	-7.408397511	-1.123717952
	H147	-6.163734474	-6.365318287	-2.379335335
	H148	-9.772231452	-4.810860826	-0.678961564
	H149	-8.548073179	-6.088332117	-2.272869976
	H150	-8.592643237	-7.272643566	-0.972785754
	H151	-8.196245676	-2.779676482	0.460846457
	H152	-9.321037519	-5.749686239	2.041989148
	H153	-9.917205093	-3.062594897	0.799339416
	H154	-8.951220805	-6.535404166	4.191976789
	H155	-6.648479876	-5.963112684	2.239513701
	H156	-8.887304560	-4.858193581	4.778483533
	H157	-3.422496052	-1.950767439	2.685441596
	H158	-5.568076599	-3.771489146	2.057306011
	H159	-4.352932268	-6.348091942	2.340885262
	H160	-6.068117365	-0.448496556	6.307679922
	H161	-6.619321035	-2.479341731	4.339616878
	H162	-4.426866960	-6.508594563	4.111253681
	H163	-5.082188987	-1.529545832	2.198908747
	H164	-5.694220199	-4.047758669	6.517999991
	H165	-7.289662511	-1.743913428	6.431494514
	H166	-0.990961471	-3.262286478	3.315583236
	H167	-0.843892199	-2.102453187	1.981260801
	H168	0.594335367	-2.542280373	2.933285262
	H169	-1.852967061	-0.982372131	3.965108535
	H170	-5.092857487	-5.267435566	8.396766634
	H171	-2.881140520	1.883870833	5.012743085
	H172	-3.172040406	-0.657889492	8.739739751
	H173	-3.521301046	2.799928859	9.227594630
	H174	1.027588505	-0.034888871	3.424836250
	H175	-0.278916910	0.971776503	4.099419089
	H176	8.360616872	1.723995843	6.164584127
	H177	-3.907658975	3.236893142	5.522853146
	H178	-4.243645766	3.778381985	7.927558099
	H179	-2.141335579	3.350537619	5.707881785
	H180	-2.496482145	3.882646283	8.255545206
	H181	-3.963924989	1.383832578	7.221658007
	H182	-0.959676708	1.950497518	7.408671321
	H183	-2.466119612	-0.201583485	6.117537892
	H184	-0.397373621	0.350657174	2.440259477
	H185	1.338638771	4.129461193	0.717044536
	H186	0.241932168	4.421126585	2.095916977
	H187	2.122168837	5.094119938	2.955094716
	H188	2.197029172	4.288923030	5.494186964
	H189	4.300167594	4.006816063	2.659569102
	H190	6.249643328	2.476339915	2.592397556
	H191	5.454252619	1.287500541	6.661756415
	H192	-2.008234354	-2.761136889	5.901185798
	H193	-1.049596166	-5.800292838	6.100231768
	H194	-0.727985165	-5.305392104	7.775731035
	H195	-3.172819188	-4.685398792	6.200235616
	H196	-4.796130432	-3.760067073	9.298471991
	H197	3.477337648	2.784497609	6.713163368
	H198	0.732031726	-1.774678712	5.368349284
	H199	-3.033445421	0.077278593	11.417088148

	H200	-3.051541476	-1.604569462	10.821376608
	H201	-0.362686546	-1.193647817	10.557911103
	H202	10.247005002	-7.275966098	5.653744314
	H203	-0.981032960	-5.800971654	-4.621435080
	H204	1.510798154	-6.033941173	-1.768804378
	H205	-0.702947164	-5.864221710	-9.003251509
	H206	0.844087190	-4.994151841	-9.174069564
	H207	-1.743476951	-4.001081984	-7.847286426
	H208	-1.200052578	1.490766124	-10.169182226
	H209	-1.440868190	-3.779809656	-10.364325935
	H210	-2.861314360	-2.023973703	-3.006319511
	H211	-3.688753262	-4.832915495	-2.107202060
	H212	-0.620633036	-0.637321862	-8.765809909
	H213	-3.362270540	0.110712144	-9.561513851
	H214	0.812997972	-2.005326323	-9.677957673
	H215	-0.891291403	-5.735032539	-2.865213303
	H216	-0.213584087	-3.148229462	-7.635355544
	H217	0.807028396	-3.330715230	-10.656643809
	H218	-6.074098094	-3.191459904	-4.307623364
	H219	-3.339713308	-2.313826861	-1.316914313
	H220	2.189120584	-4.949009293	-7.504197951
	H221	-2.106108572	-4.220531385	-1.655716146
	H222	-4.335445587	2.517246811	2.431104408
	H223	-3.079976378	0.572617947	0.861359940
	H224	-8.628285383	0.659075168	2.216160098
	H225	-7.296783680	-0.016325301	-0.370961484
	H226	-7.247921145	4.073295425	1.256462181
	H227	-8.995024308	8.632012710	3.310908713
	H228	-7.637183374	5.093865855	4.258406015
	H229	-9.404104606	5.410732488	2.091640179
	H230	-6.657455380	6.792237463	1.891764665
	H231	-10.801202843	9.659337233	-0.380558112
	H232	-3.404832877	4.244073928	0.901415770
	H233	-4.767916463	4.047565684	-0.209962990
	H234	-10.210596607	10.438209845	1.979336476
	H235	-8.592387573	5.666578911	0.545124333
	H236	-6.324453008	2.084238534	0.281851638
	H237	-7.499734322	-0.683651900	2.025334419
	H238	-9.600367348	2.123811211	-1.951110795
	H239	-1.782106408	1.646406295	-3.043537606
	H240	-9.995053804	0.400009114	-2.080177460
	H241	9.646207876	3.133013574	4.728700557
	H242	8.122354997	3.242649079	4.146429886
	H243	1.730731488	5.969497830	-0.868540563
	H244	-0.843349925	5.446553153	0.319522357
	H245	1.575532866	7.962613525	0.963930291
	H246	-3.145232596	3.654584417	-2.085305409
	H247	-4.488641979	4.392566421	-3.507823289
	H248	-5.731634821	3.694484763	-5.233969778
	H249	-4.343934556	5.587895077	-2.464716909
	H250	-4.640835643	1.538776825	-5.004216133
	H251	-6.427648062	1.577303857	-5.147426415
	H252	0.293326142	11.722363445	-1.257906788
	H253	-0.885190646	13.045570193	-1.215508376
	H254	-0.296287494	10.418523294	-2.974952033
	H255	-1.962695928	9.949879779	-3.314785485
	H256	-2.559236514	9.442352581	-6.315812206
	H257	-1.535675678	5.139791185	-5.954972887
	H258	-7.014939757	8.834771416	-1.505683265
	H259	-3.436719765	8.467884087	1.929882206
	H260	-3.961515808	8.011336917	-3.407094227
	H261	1.787640694	5.571862687	-3.424019280
	H262	2.862454908	7.337411092	-2.372954274
	H263	-0.700263312	4.991503350	-3.414379598
	H264	-7.940118203	0.019116513	-3.073578269
	H265	4.290943405	10.215833571	-2.910137815
	H266	-5.397059431	-0.698864104	-4.239533655
	H267	1.509551905	6.647529262	-4.792573185
	H268	-3.365971623	7.027413466	-0.061388702
	H269	3.653357715	10.391891008	-1.262815172
	H270	-1.787120361	7.996170847	-6.871792662
	H271	-4.223448463	9.173745518	-4.661413021
	H272	-4.423263313	-3.263600826	-4.554280027
	H273	-5.532734441	-3.201340766	-2.081616002
	H274	-7.017069162	10.373524509	0.436808247
	H275	-4.833766888	1.549413717	-2.527942691
	H276	0.180165566	8.188947967	-3.371102462
	H277	-0.595134313	6.638090030	-5.977631682
	H278	0.288041044	4.460466044	-4.766433866
	H279	-2.799270210	6.098361967	-3.995637831

	H280	0.195087204	8.684478203	3.423114527
	H281	-0.483406475	9.754658597	0.451713302
	H282	-0.134323327	12.582190071	1.004891014
	H283	1.803726375	9.214432650	2.872439617
	H284	-2.743760645	10.574439308	1.717463012
	H285	6.201336886	-2.660066833	-8.280965517
	H286	6.023223797	-4.135345717	-7.313628480
	H287	8.728146565	-5.125848072	-3.920533674
	H288	6.877011901	-4.716604072	-9.467859566
	H289	5.314071008	-5.206064021	-9.466565422
	H290	5.567662521	4.803153530	0.323027584
	H291	5.599878905	7.396465759	1.877865617
	H292	5.363690958	8.252693674	-2.281066773
	H293	4.070065069	5.621480318	0.867625115
	H294	6.815382119	8.054142384	1.001783909
	H295	6.742272914	2.869019226	-5.745854416
	H296	6.253090502	6.812867874	-0.925215044
	H297	4.368229004	-7.295174332	5.603275291
	H298	6.471278747	-8.091779469	3.515444134
	H299	4.896812053	1.549732979	-5.981984486
	H300	4.348084986	-0.289826337	3.931063726
	H301	2.152185991	1.888983154	-8.260770507
	H302	-0.351357059	-1.924342536	-4.566452435
	H303	1.786303505	2.374116878	-6.603026548
	H304	3.516628546	-4.704720017	5.151320421
	H305	3.274424855	0.283133468	-5.978425900
	H306	3.322116225	-5.236998475	3.469800679
	H307	4.100672472	-7.512872532	3.857298656
	H308	5.839435094	-5.694583182	3.425344052
	H309	5.880622308	-5.362353728	5.163552838
	H310	-3.901858139	-0.427112865	-7.348466963
	H311	4.911392203	-9.771011710	5.436154690
	H312	4.296011286	-11.934695605	3.390693231
	H313	0.944259740	0.146107572	-4.010377885
	H314	5.082729623	-12.201297876	6.189780365
	H315	3.621498808	-12.439990311	5.490366684
	H316	5.125133382	-3.529305569	2.916547784
	H317	0.182057892	2.334830721	-4.820005856
	H318	-2.318425676	-1.776810066	-6.076766832
	H319	7.820572408	-9.893179696	-0.682071818
	H320	7.125654018	-7.436335095	-2.692918596
	H321	6.519480396	-8.226035522	0.765532052
	H322	7.174560125	-10.459973770	-1.977963814
	H323	6.449413550	3.025034490	-8.509635454
	H324	6.294294313	-5.121861370	0.713545339
	H325	2.085080577	-7.881102595	0.923519586
	H326	4.628300854	-1.728953317	-6.924400380
	H327	3.144466664	-9.233113874	-0.671494202
	H328	4.235416088	-6.609126460	-6.566375464
	H329	3.082742887	-5.234897636	-4.779930977
	H330	5.780602382	-12.673350762	4.000598210
	H331	5.306872957	-1.232796390	2.838135973
	H332	8.078444303	-5.885854069	2.061698318
	H333	7.084944548	-3.821850882	1.012790351
	H334	8.307029552	-7.053834419	4.084684000
	H335	2.659557000	-2.943316659	5.535362884
	H336	4.366570188	-8.166032283	-3.270998192
	H337	5.320456253	-8.546296135	-1.832551707
	H338	2.249954872	-8.640744733	-2.079720237
	H339	2.463331857	-1.935015783	-5.834238911
	H340	-1.788496197	2.492816657	-6.369786346
	H341	-3.775891535	1.349736346	-7.420614370
	H342	2.158280725	-6.109698159	0.829690313
	H343	8.249539778	-7.412156195	1.811358408
	H344	7.181154721	-9.340393274	1.605350459
	H345	3.090225797	-1.268178296	5.617798133
	H346	6.616872015	-7.290086537	-5.877519888
	H347	7.048652574	-5.767466993	-2.161873576
	H348	1.828936585	-2.639087409	-7.352364914
	H349	5.650275200	4.270076069	-5.828810137
	H350	6.985322460	-0.055748611	5.181922018
	H351	7.352684773	0.504175031	3.535020528
	H352	9.932486945	-6.856749460	-4.992782162
	H353	8.967536165	-8.028705700	-4.381239116
	H354	10.495625214	-8.579176738	-0.187725196
	H355	9.013894403	-8.211705670	-1.880333372
	H356	12.234128979	-7.368810292	-1.917926372
	H357	10.163676509	-6.906665013	0.318176203
	H358	10.034796732	0.278998357	4.008550781
	H359	-3.612087086	12.976793085	3.805437288

H360	9.068915982	-7.217534184	6.990041405
H361	8.491747305	-4.758784478	5.921154696
H362	-4.887784229	12.312217176	1.132660960
H363	-4.691632106	10.154818665	3.329622003
H364	-6.189697803	10.881774861	2.718872799
H365	1.184442428	-3.502857216	-0.734231693
H366	4.191151206	-1.350837456	0.773689926
H367	-5.985446221	7.036476583	-2.477549696
H368	0.190760676	-0.630547255	-0.393513384
H369	1.939713876	-1.805772553	0.437384656
H370	1.307540643	-2.712419568	0.189286171
H371	-1.793622584	-0.141751098	-1.244373094
Mo372	4.161063862	-4.332006088	-1.760744359
N373	-6.733861483	-7.075049712	-1.912789927
N374	-8.952775957	-4.676545313	-0.091532495
N375	-8.867771519	-5.104829401	2.685285272
N376	-6.225168528	-5.952900125	3.169186877
N377	-4.823554587	-3.525042698	2.706304211
N378	-5.907862224	-2.003074208	4.900083059
N379	-5.225799396	-3.526999214	7.265076440
N380	-2.569902441	-4.689773939	7.027693196
N381	-0.991196765	-2.712296465	5.912833928
N382	-1.655511748	0.152865213	6.631953714
N383	-2.496862229	-0.227743056	9.376200786
N384	4.439937938	-11.829391004	5.487135346
N385	5.547331798	-9.529466736	4.668917372
N386	4.483379568	-3.506843044	3.713489191
N387	3.255876522	-2.180922661	5.204404662
N388	4.721119523	-1.202972300	3.689588645
N389	8.201940179	-7.234406423	5.107683177
N390	-5.192836667	-2.698750551	-4.150332806
N391	-1.354423295	-5.449324989	-3.732965770
N392	-5.565317064	-0.238907538	-3.328576190
N393	-8.011295488	0.856179204	-2.499087589
N394	-8.001114461	0.371135717	0.266346619
N395	-6.000411679	2.075309238	1.253632931
N396	-2.918043601	2.765218994	-1.564279308
N397	-2.278507655	0.751962474	-1.137446484
N398	-6.737739958	4.680549591	1.892970664
N399	-9.860419338	7.807023802	0.202405492
N400	-9.997422847	9.514343492	1.612525658
N401	8.911744991	2.599916631	4.256739788
N402	6.272614873	7.195485840	1.130790932
N403	4.629876735	8.581303646	-1.658988136
N404	2.108003562	7.989121040	-2.615431639
N405	-2.260805572	6.641188299	-4.693993198
N406	-2.251508592	8.495026832	-6.121337207
N407	-3.605839886	8.483030529	-4.242383021
N408	0.778063397	6.319670353	-0.671624348
N409	0.702759664	8.113205635	1.476898787
N410	-0.494701236	10.562824199	1.073607930
N411	-1.304675731	10.574462696	-2.851107007
N412	-3.018333945	11.553804860	1.733407787
N413	5.913230561	-4.375471533	-9.471278511
N414	3.870976657	-2.269536153	-7.353343309
N415	2.743646061	0.507840424	-6.835144724
N416	4.872455828	2.416861126	-6.524573168
N417	0.233740383	-2.552057148	-10.325557210
N418	-1.604465903	-0.771253743	-9.020335770
N419	8.974460074	-7.050998727	-4.687451935
N420	4.930385782	-5.472649062	-3.682862820
N421	4.683087691	-6.328183457	-5.698002408
N422	9.646868235	-7.429770140	-1.686160217
O423	-6.756530757	-5.172925293	0.245107916
O424	-7.999788227	-3.012073759	2.964746553
O425	-6.519342045	-5.492990797	5.389648299
O426	-3.311218138	-4.246598619	4.243135943
O427	-3.913875970	-0.911078936	4.922909114
O428	-4.936348043	-1.497576283	8.281652523
O429	-2.427889760	-4.552433760	9.313466663
O430	0.931547472	-3.923777315	6.279481910
O431	0.612101141	0.089125438	6.919215439
O432	-1.051880027	1.461848773	9.836780689
O433	-0.852226129	-0.821347009	12.504725510
O434	6.599618990	-10.510452177	2.858810801
O435	6.900659757	-8.124295837	6.781649464
O436	10.485225670	-4.770107699	6.353694199
O437	-5.537512989	-0.760364331	-1.094118147
O438	-2.864825283	-4.158618322	-4.839369369
O439	-6.877325428	2.631658410	-1.618226340

	O440 -5.566151168 3.308559629 -4.355023240<br>O441 -9.993665200 1.488094210 0.489797410<br>O442 -6.240857357 1.214726908 3.357943776<br>O443 -4.851975524 4.935168612 3.154107156<br>O444 -7.379959697 7.113695797 4.392362362<br>O445 9.899654496 -0.323839122 5.953998094<br>O446 2.682867291 4.339119468 4.650545371<br>O447 3.641701111 8.366524274 0.386932761<br>O448 1.586218950 10.216271803 -2.873928412<br>O449 -1.149596020 7.236170405 -1.491511518<br>O450 -1.185235656 7.064148663 2.250492723<br>O451 2.005884196 5.544988764 2.093861660<br>O452 0.367365847 11.399150210 3.026537285<br>O453 -2.527589600 13.603234628 0.863380203<br>O454 -2.963778066 11.160675594 -1.393609350<br>O455 -5.501354417 13.593506969 3.330780558<br>O456 -5.115348213 7.015136414 -2.033913627<br>O457 3.343249487 -4.222916449 -8.442376137<br>O458 1.349100570 -0.381206527 -8.422952211<br>O459 3.708757181 3.904717186 -7.784270424<br>O460 7.280955792 4.756554907 -7.818539295<br>O461 -3.226305140 -2.182487060 -9.778810668<br>O462 -0.345004987 -6.344781329 -6.451590910<br>O463 1.669084821 -5.454157434 -6.804231095<br>O464 -2.283948232 2.732560407 -8.971854860<br>O465 10.392651761 -5.328528089 -2.089527919<br>O466 12.765683838 -6.819977651 -0.024801987<br>O467 0.499017023 -6.283903338 -1.488921751<br>O468 -0.367748213 -7.421251848 0.244622008<br>O469 5.657726683 -10.816199168 -2.782718829<br>O470 3.450703382 -10.818001554 -3.275043996<br>O471 5.236993987 -5.888444803 -0.623124753<br>O472 4.889597367 -7.628622174 0.752065924<br>O473 2.912190828 -6.056168736 -1.788540937<br>O474 8.606266612 -6.663543511 2.366548926<br>O475 6.779014582 -4.646991280 1.436854869<br>O476 -3.826538834 4.937432587 -3.002942673<br>O477 8.060177304 -10.353024711 -1.517401940<br>O478 7.398720107 -8.670095021 0.909210544<br>S479 3.936203230 5.031479850 -1.486696323<br>S480 2.839426135 2.075368358 -3.082165140<br>S481 2.569689724 -3.205229624 -3.083086313<br>S482 3.604744020 1.747588904 0.495032664<br>S483 0.792030028 -0.847536638 -1.595774659<br>S484 5.042766157 -0.685917131 -4.695587878<br>S485 3.608139568 -3.647742604 0.378267201<br>S486 6.216896409 1.799463958 -1.963697438<br>S487 6.128925422 -3.029738918 -2.031220372<br>S488 6.448900365 -0.879360650 0.720778449<br>N489 1.027873652 1.917713025 -0.852298828<br>N490 0.142563234 2.585181381 -0.636666958<br>end

TS for 6H2x dissociation 9 to 9d (Scheme 3)

Fe(139) -2.425<br>Fe(140) -1.114<br>Fe(141) -2.578<br>Fe(142) 2.724<br>Fe(143) 2.739<br>Fe(144) 1.573<br>Fe(145) 0.064<br><br>**S=1/2**	2n2x2bh7hn6hn6h2xdtd.car_1<br>C1 -7.842435020 -5.389808388 -0.362660907<br>C2 -7.977994483 -6.490756453 -1.418686332<br>C3 -8.946890889 -3.531274183 0.798969596<br>C4 -8.577565384 -3.860672149 2.243267351<br>C5 -8.530538375 -5.542517087 4.020352744<br>C6 -7.019747280 -5.661241712 4.247047459<br>C7 -4.810744562 -5.942676151 3.244292639<br>C8 -4.239879691 -4.536512067 3.440161558<br>C9 -4.455620939 -2.155837096 2.920507629<br>C10 -4.719506639 -1.656774413 4.347794325<br>C11 -6.221669599 -1.554087991 6.243528116<br>C12 -5.387121946 -2.209707148 7.352139368<br>C13 -4.608043094 -4.311850577 8.341587529<br>C14 -3.087488215 -4.511441147 8.270177159<br>C15 -1.140183909 -4.952083886 6.827384328<br>C16 -0.284509762 -3.789684334 6.321395201<br>C17 -0.303929135 -1.487004982 5.438217399<br>C18 -0.405047791 -0.310456118 6.419222550<br>C19 -0.727092724 -1.142663270 3.983225613<br>C20 -0.011002604 0.121068995 3.490498702<br>C21 -0.450983060 -2.320472736 3.041171973<br>C22 -1.843456222 1.314687376 7.558573115<br>C23 -1.783167240 0.872228092 9.033461780<br>C24 -3.127629638 2.099520384 7.202366865

	C25	-3.385531865	3.212751227	8.223710466
	C26	-3.021497842	2.680051131	5.786030631
	C27	-2.538467474	-0.643240422	10.771043431
	C28	-1.145293669	-0.882888719	11.318665318
	C29	5.088018664	-11.854075135	4.159994193
	C30	5.839765536	-10.564453001	3.836126644
	C31	6.034521910	-8.175151939	4.512582334
	C32	7.102071749	-7.860881706	5.579050963
	C33	4.847950945	-7.199319193	4.615139399
	C34	5.254921191	-5.751339523	4.349104941
	C35	4.051303692	-4.819612243	4.215879457
	C36	4.149477494	-2.311731451	4.182430442
	C37	9.298723101	-6.839616572	5.972156686
	C38	9.425857949	-5.336074043	6.092965909
	C39	-4.970693806	-2.506188752	-2.738859136
	C40	-5.401054731	-1.091007470	-2.308845858
	C41	-3.478279877	-2.677726507	-2.362681070
	C42	-2.924786846	-4.116788435	-2.412676644
	C43	-2.374004443	-4.567555545	-3.766175537
	C44	-5.671377367	1.204724563	-3.165686389
	C45	-6.910794028	1.640476690	-2.372686173
	C46	-5.575235293	1.883458282	-4.538458472
	C47	-9.224652187	1.123987730	-1.751783472
	C48	-9.094930751	1.013452148	-0.228290859
	C49	-7.705141073	0.336931889	1.675888080
	C50	-6.573076757	1.243293336	2.161190153
	C51	-4.964358975	3.019212970	1.667425196
	C52	-5.518442751	4.296996254	2.342737253
	C53	-4.114287626	3.514243482	0.486614852
	C54	-3.364366214	2.481094153	-0.286885658
	C55	-2.958642102	1.196822318	-0.019622502
	C56	-2.249638172	1.717977090	-2.066243031
	C57	-7.315032584	5.982630745	2.259021001
	C58	-7.436897273	6.075276125	3.778380167
	C59	-8.703614221	6.080001774	1.583252178
	C60	-9.274527690	7.458927685	1.424428975
	C61	-9.348994383	8.513488816	2.313345953
	C62	-10.286132024	9.064439366	0.382483779
	C63	8.510577549	1.480697348	5.079325362
	C64	9.585523890	0.412155122	4.996459822
	C65	7.201807456	0.841518366	4.553744613
	C66	6.018173401	1.776642489	4.613151830
	C67	5.217539521	1.871317351	5.765259882
	C68	5.674407800	2.570675972	3.506725906
	C69	4.101142294	2.713978187	5.807653507
	C70	4.572412945	3.426346188	3.538555929
	C71	3.779734013	3.488400884	4.687032197
	C72	5.524226801	6.884114269	-0.110304849
	C73	4.511889358	7.998997323	-0.430444196
	C74	4.808013664	5.548036760	0.051280602
	C75	3.792476296	9.684840657	-2.090547552
	C76	2.386410706	9.310320813	-2.566254720
	C77	0.808607115	7.409022303	-2.915596408
	C78	0.068978815	6.975883901	-1.624451650
	C79	1.040798048	6.232038379	-3.891125201
	C80	-0.147352442	5.354483033	-4.301491754
	C81	-1.129560500	5.946054212	-5.322799566
	C82	-2.714141646	7.846554455	-5.043017165
	C83	0.147756128	5.913111224	0.575362640
	C84	-0.193983442	7.093195556	1.495679385
	C85	0.992714870	4.918524808	1.397075181
	C86	0.751404877	9.077150224	2.568485992
	C87	0.195480926	10.455185926	2.249686602
	C88	-0.858255206	11.871894198	0.567879450
	C89	-2.224365453	12.416763606	1.053576669
	C90	-0.731671275	11.984132256	-0.954136935
	C91	-1.774077295	11.188984910	-1.731821456
	C92	-4.409411481	11.881916020	2.031095660
	C93	-4.505562702	12.911139215	3.147962087
	C94	-5.170725373	10.588316594	2.438847766
	C95	-5.208744957	9.575897041	1.315892561
	C96	-6.212377348	9.646083560	0.337057348
	C97	-4.216152623	8.592775929	1.174335618
	C98	-6.212021610	8.788228869	-0.764527218
	C99	-4.186051679	7.740290630	0.065843682
	C100	-5.184191726	7.853870188	-0.896919351
	C101	5.678175608	-3.635105571	-8.223969442
	C102	4.192175098	-3.397891912	-8.021366347
	C103	2.524995765	-1.911207548	-6.932453182
	C104	2.139438320	-0.526787844	-7.463964709

	C105	2.549490812	1.887256092	-7.228314019
	C106	3.771752044	2.798777649	-7.192637090
	C107	5.989548062	3.312464642	-6.361339861
	C108	6.600758339	3.756003358	-7.676336535
	C109	-0.968618567	-3.072712779	-9.677663832
	C110	-2.028355017	-1.976586735	-9.518121598
	C111	-0.770543418	-3.806184727	-8.329294595
	C112	-0.028752221	-5.143099233	-8.521516276
	C113	0.398661669	-5.714249537	-7.186824306
	C114	-2.537674120	0.320186694	-8.857865715
	C115	-1.939490320	1.617402343	-9.366153281
	C116	-3.188391010	0.414276838	-7.455044437
	C117	-2.155993935	0.370484406	-6.361894767
	C118	-1.473101207	1.533231542	-5.966929924
	C119	-1.755788435	-0.865184084	-5.822752050
	C120	-0.373916945	1.449606601	-5.106414437
	C121	-0.655633265	-0.947778998	-4.965446725
	C122	0.057601069	0.207601477	-4.630748056
	C123	8.640385172	-6.183316312	-3.567043389
	C124	9.627436480	-6.271928381	-2.392423177
	C125	7.211910678	-6.388042163	-3.038685472
	C126	6.102098472	-6.154418848	-4.012711208
	C127	5.949225249	-6.698224033	-5.271469693
	C128	4.099012014	-5.619856126	-4.711279777
	C129	10.511673557	-7.519918069	-0.516267636
	C130	11.948391064	-7.180372326	-0.848017244
	C131	0.616355211	-6.943798081	-0.345089845
	C132	2.023948170	-7.010676013	0.245256364
	C133	3.159609928	-7.066301888	-0.816536248
	C134	3.182559801	-8.471178592	-1.479599080
	C135	4.404725728	-8.769233136	-2.351346743
	C136	4.505249008	-10.237123062	-2.823048787
	C137	4.514996690	-6.833195117	-0.131521357
	C138	3.913198121	-0.622808757	-1.540966771
	Fe139	4.460670591	-1.879653970	-2.968583366
	Fe140	2.412266544	0.862650187	-1.231387329
	Fe141	5.215833220	0.511006830	-0.499230632
	Fe142	4.488188518	0.636566043	-2.971968548
	Fe143	4.213250163	2.791611565	-1.393548504
	Fe144	4.882119615	-1.975019058	-0.427793911
	Fe145	2.430301566	-2.298776354	-1.098532134
	H146	-6.176018553	-7.398300322	-1.114113503
	H147	-6.173181584	-6.356047667	-2.370230159
	H148	-9.793140913	-4.815009527	-0.684045498
	H149	-8.559815378	-6.101000992	-2.269160908
	H150	-8.595923392	-7.286170459	-0.967459727
	H151	-8.219906925	-2.781085890	0.454529276
	H152	-9.359114636	-5.741919462	2.046009179
	H153	-9.941273549	-3.065460769	0.794282221
	H154	-8.990179936	-6.524061271	4.196960477
	H155	-6.689018944	-5.957330930	2.232456845
	H156	-8.909406977	-4.845748417	4.779287949
	H157	-3.388416750	-2.004347023	2.717924836
	H158	-5.583068525	-3.773318968	2.076218781
	H159	-4.402805617	-6.379893524	2.322437617
	H160	-6.057334322	-0.471084647	6.316672845
	H161	-6.608933120	-2.489865091	4.339021104
	H162	-4.470579857	-6.549172401	4.092701103
	H163	-5.032436225	-1.547138255	2.210802614
	H164	-5.693820595	-4.072065485	6.512808152
	H165	-7.283505596	-1.763264671	6.432581379
	H166	-0.957762720	-3.239125092	3.368379046
	H167	-0.807624984	-2.087813003	2.024963147
	H168	0.629152474	-2.523957631	2.967367789
	H169	-1.811316187	-0.947320850	3.993158081
	H170	-5.072941664	-5.308066470	8.370956030
	H171	-2.894834105	1.897548155	5.025112546
	H172	-3.186169315	-0.646702762	8.740578846
	H173	-3.547912934	2.818835157	9.237313283
	H174	1.080333199	-0.027085822	3.460528366
	H175	-0.219943750	0.997439235	4.121204609
	H176	8.364443164	1.726274750	6.148924711
	H177	-3.928028088	3.246937269	5.531202535
	H178	-4.276655085	3.788176338	7.934093294
	H179	-2.162648806	3.368267581	5.719139007
	H180	-2.530537381	3.906333991	8.263123365
	H181	-3.978521150	1.394784972	7.232352656
	H182	-0.980531329	1.987206955	7.439998935
	H183	-2.451609645	-0.180690360	6.130275760
	H184	-0.334840659	0.363004211	2.466316544

H185	1.426766800	4.169970887	0.719423222
H186	0.300589946	4.408796962	2.084969132
H187	2.142408412	5.130479207	2.979141874
H188	2.194679283	4.269690944	5.497041703
H189	4.305639072	4.021560704	2.665492867
H190	6.259884499	2.496290236	2.588039041
H191	5.457615403	1.265740343	6.643257143
H192	-1.979159384	-2.708402855	5.981966675
H193	-1.025050412	-5.777223911	6.108568239
H194	-0.712793471	-5.277588435	7.784881090
H195	-3.149780965	-4.672251657	6.194933281
H196	-4.806390432	-3.809910210	9.296350685
H197	3.476231130	2.756976357	6.704046193
H198	0.762645610	-1.733614393	5.421309013
H199	-3.061442936	0.071742588	11.426654725
H200	-3.072323253	-1.605915848	10.815740143
H201	-0.385350715	-1.185380827	10.560070690
H202	10.258768479	-7.264805010	5.642146841
H203	-0.977325096	-5.799787979	-4.615323005
H204	1.557300878	-6.071066277	-1.794905352
H205	-0.699948329	-5.867599124	-9.004266284
H206	0.847937201	-4.997860834	-9.169417305
H207	-1.745729471	-4.001658033	-7.856874727
H208	-1.183678527	1.486517369	-10.175301024
H209	-1.433752063	-3.784812716	-10.375547798
H210	-2.876236589	-2.027033218	-3.018942308
H211	-3.712722624	-4.834381202	-2.125899726
H212	-0.617819201	-0.641513642	-8.771775551
H213	-3.358559524	0.109738409	-9.573853416
H214	0.816259257	-2.008152484	-9.678833031
H215	-0.902445730	-5.747340371	-2.859867858
H216	-0.216796188	-3.147983681	-7.641897133
H217	0.818686306	-3.335855968	-10.653700835
H218	-6.079407073	-3.187600555	-4.341254340
H219	-3.362141540	-2.311445822	-1.331975845
H220	2.186346892	-4.953020532	-7.493069975
H221	-2.138793461	-4.225759324	-1.651196495
H222	-4.337470414	2.525348712	2.423006859
H223	-3.106637936	0.568817983	0.849661805
H224	-8.614011203	0.649447264	2.209234684
H225	-7.289058884	-0.007411368	-0.385695946
H226	-7.256186789	4.080834111	1.272744241
H227	-8.991675663	8.639535647	3.329268387
H228	-7.630663593	5.093632850	4.274433897
H229	-9.410734020	5.419080194	2.114766174
H230	-6.665424175	6.801024719	1.909778636
H231	-10.797003629	9.671053882	-0.361624258
H232	-3.397784353	4.244920384	0.896222408
H233	-4.763016771	4.061630843	-0.215231639
H234	-10.201655037	10.449529176	1.997427883
H235	-8.601345971	5.670108962	0.566369901
H236	-6.331566506	2.102060867	0.276439919
H237	-7.481406862	-0.688693614	2.003679562
H238	-9.594427884	2.138932884	-1.952869586
H239	-1.762640486	1.651403899	-3.036662005
H240	-9.995208715	0.416920825	-2.084596917
H241	9.641320333	3.152235635	4.727055053
H242	8.117019643	3.259758732	4.146968945
H243	1.748284762	5.977374637	-0.875965878
H244	-0.807161944	5.428144262	0.327221914
H245	1.580084981	7.973458327	0.969828510
H246	-3.134469591	3.658807778	-2.087513490
H247	-4.491817987	4.396886236	-3.513121564
H248	-5.726130839	3.703279491	-5.248418099
H249	-4.348945010	5.592329746	-2.469235270
H250	-4.645094560	1.544244933	-5.024337612
H251	-6.432320761	1.587726718	-5.163232416
H252	0.291401930	11.719981240	-1.257407094
H253	-0.886613462	13.043433888	-1.213789588
H254	-0.298362743	10.420514971	-2.976702776
H255	-1.964910357	9.954451621	-3.319827643
H256	-2.564947717	9.439280675	-6.325973906
H257	-1.534088115	5.143051746	-5.958289503
H258	-7.008154568	8.847456058	-1.510646212
H259	-3.441336385	8.467523186	1.935148291
H260	-3.960837477	8.014467398	-3.412763773
H261	1.791388355	5.569719174	-3.430290856
H262	2.870674089	7.340489888	-2.381620651
H263	-0.698068045	4.992980632	-3.418651772
H264	-7.944829893	0.030450755	-3.085002622

	H265	4.292825695	10.221091814	-2.907238726
	H266	-5.400533297	-0.691647324	-4.261537590
	H267	1.512498567	6.648512893	-4.796278477
	H268	-3.368461991	7.028369587	-0.057162455
	H269	3.654410740	10.393159876	-1.260167061
	H270	-1.790588831	7.994131486	-6.879295441
	H271	-4.222172721	9.174979355	-4.668335007
	H272	-4.426579381	-3.261283042	-4.572911164
	H273	-5.555434560	-3.200865505	-2.107575307
	H274	-7.012922111	10.383111778	0.434153617
	H275	-4.831543577	1.550679831	-2.547736788
	H276	0.184118814	8.185432964	-3.370942471
	H277	-0.592555497	6.640858077	-5.980993270
	H278	0.289612899	4.462242031	-4.771502714
	H279	-2.797463838	6.101071563	-3.998963341
	H280	0.181677248	8.682968152	3.423186928
	H281	-0.483370229	9.752316834	0.455018018
	H282	-0.136434287	12.579674389	1.004662085
	H283	1.793946888	9.215911331	2.886716582
	H284	-2.746933238	10.575123809	1.718333929
	H285	6.206069833	-2.670862758	-8.271915350
	H286	6.024518226	-4.142431546	-7.299283616
	H287	8.739811989	-5.156196038	-3.947940036
	H288	6.879742939	-4.732283587	-9.450160442
	H289	5.316653155	-5.220266624	-9.448458838
	H290	5.560520237	4.791363765	0.305989623
	H291	5.598763258	7.386216081	1.869914052
	H292	5.367735789	8.257763136	-2.283751881
	H293	4.068911108	5.615651847	0.862054080
	H294	6.815129930	8.041157816	0.994243528
	H295	6.775755174	2.801561581	-5.784584674
	H296	6.251826018	6.802235070	-0.933057420
	H297	4.384661796	-7.302069046	5.610940336
	H298	6.476359341	-8.096629740	3.510938859
	H299	4.921650928	1.493161287	-6.039530126
	H300	4.345968436	-0.297459055	3.884231280
	H301	2.154434207	1.892597485	-8.251464942
	H302	-0.341000694	-1.915477239	-4.568032288
	H303	1.788728787	2.368692908	-6.591078456
	H304	3.555639634	-4.691914670	5.186120408
	H305	3.276847670	0.278395891	-5.975889394
	H306	3.310915333	-5.242187865	3.517139581
	H307	4.105326366	-7.519236879	3.866622365
	H308	5.833842580	-5.705038464	3.415554388
	H309	5.907007604	-5.374083366	5.152176925
	H310	-3.891356551	-0.425651710	-7.355925414
	H311	4.916619926	-9.780065524	5.428318043
	H312	4.315483617	-11.950672420	3.382038587
	H313	0.937195196	0.158090754	-3.990235899
	H314	5.077273224	-12.211074162	6.187559733
	H315	3.619517754	-12.441647090	5.476661686
	H316	5.130685234	-3.549793475	2.913129972
	H317	0.172804324	2.347040435	-4.810709987
	H318	-2.298867446	-1.774145556	-6.090067672
	H319	7.834827409	-9.891924431	-0.678203206
	H320	7.117437285	-7.420052880	-2.660092213
	H321	6.520981498	-8.226028593	0.772642613
	H322	7.186800103	-10.447263946	-1.976050436
	H323	6.431621215	3.060263880	-8.532147588
	H324	6.284068543	-5.093371575	0.746648874
	H325	2.078961103	-7.870332778	0.924723874
	H326	4.631483289	-1.730647311	-6.921438126
	H327	3.144836179	-9.202992814	-0.657606502
	H328	4.235482879	-6.633072955	-6.555538198
	H329	3.087474194	-5.242259125	-4.779173676
	H330	5.796462132	-12.685376839	4.005608164
	H331	5.332456897	-1.254224325	2.834018082
	H332	8.078173933	-5.884419039	2.067433623
	H333	7.112956109	-3.814281690	1.038968963
	H334	8.314079963	-7.057826877	4.079486556
	H335	2.656596966	-2.928970234	5.518717748
	H336	4.377569308	-8.134827844	-3.250153148
	H337	5.326720639	-8.519947600	-1.809295361
	H338	2.258968969	-8.599240887	-2.064715191
	H339	2.458769650	-1.942410157	-5.833126930
	H340	-1.784768118	2.498073272	-6.374346512
	H341	-3.765850245	1.350897300	-7.431196441
	H342	2.146181476	-6.102333136	0.859689397
	H343	8.250002072	-7.410279918	1.808751157
	H344	7.182501070	-9.345166923	1.607772673

	H345	3.121458544	-1.260215502	5.608802309
	H346	6.619742327	-7.307019367	-5.864399872
	H347	7.060339130	-5.738147367	-2.168570368
	H348	1.833111050	-2.642185276	-7.355257837
	H349	5.681106071	4.203029927	-5.791608593
	H350	7.001055676	-0.054980683	5.158549151
	H351	7.364025947	0.516582053	3.513389143
	H352	9.919008103	-6.927867583	-4.987873519
	H353	8.934735213	-8.071746903	-4.354144892
	H354	10.499085314	-8.566973291	-0.173839847
	H355	9.025392943	-8.219588089	-1.875343875
	H356	12.237729619	-7.364560198	-1.910777347
	H357	10.162906008	-6.891683222	0.318717775
	H358	10.048617078	0.306182088	3.985280901
	H359	-3.611448398	12.974531406	3.812176998
	H360	9.084088809	-7.230165535	6.982507244
	H361	8.475907326	-4.770233732	5.925567869
	H362	-4.889532011	12.316701352	1.138577754
	H363	-4.693826326	10.156096584	3.332475507
	H364	-6.191413851	10.883980372	2.721393561
	H365	0.574900672	-3.883615705	-0.963231037
	H366	4.250391157	-1.336087423	0.771950908
	H367	-5.983721882	7.046242360	-2.479193813
	H368	0.248188218	-0.615871280	-0.246074213
	H369	1.990429903	-1.873828358	0.290473419
	H370	0.679743000	-3.603362714	-0.264125291
	H371	-1.788838934	-0.138080439	-1.241555936
	Mo372	4.165341518	-4.291294375	-1.760852158
	N373	-6.737793623	-7.070511207	-1.904170694
	N374	-8.976157054	-4.680831135	-0.093281540
	N375	-8.899688313	-5.098543418	2.686412970
	N376	-6.263045557	-5.958704559	3.161141106
	N377	-4.821300992	-3.548761953	2.713211866
	N378	-5.896361269	-2.022955130	4.905696732
	N379	-5.225111326	-3.555194228	7.262386687
	N380	-2.550338367	-4.664156572	7.025214352
	N381	-0.961688189	-2.674132108	5.959121558
	N382	-1.649510616	0.185934699	6.649149510
	N383	-2.519149317	-0.214397060	9.383884360
	N384	4.442332266	-11.837021713	5.479126290
	N385	5.554965277	-9.538777204	4.663090441
	N386	4.490442050	-3.515856012	3.711986149
	N387	3.258616444	-2.172187515	5.183719461
	N388	4.737412515	-1.211261651	3.674480926
	N389	8.211726207	-7.242728537	5.102061026
	N390	-5.199093022	-2.696370774	-4.174950681
	N391	-1.352789159	-5.444448643	-3.728837926
	N392	-5.571705529	-0.234300960	-3.350036331
	N393	-8.011452823	0.867379745	-2.509750082
	N394	-7.992216721	0.377208100	0.254879764
	N395	-6.003206284	2.088272388	1.246711785
	N396	-2.909190274	2.770719983	-1.565363868
	N397	-2.282473765	0.752682426	-1.139312246
	N398	-6.740792960	4.689677091	1.903411555
	N399	-9.863176795	7.815605132	0.222149981
	N400	-9.992261658	9.524656324	1.631037501
	N401	8.909893690	2.619894244	4.249209455
	N402	6.271577954	7.182870159	1.123408767
	N403	4.634006070	8.583358400	-1.660023133
	N404	2.114279875	7.990254213	-2.622535113
	N405	-2.257032399	6.644149005	-4.696031714
	N406	-2.249818989	8.495283353	-6.127157367
	N407	-3.604095874	8.485228667	-4.248150146
	N408	0.796219929	6.325838189	-0.672042725
	N409	0.703980223	8.118830323	1.478695705
	N410	-0.497933469	10.561060704	1.075988970
	N411	-1.306680615	10.575802625	-2.852139269
	N412	-3.020037647	11.554801050	1.735329465
	N413	5.916358496	-4.390130091	-9.455998266
	N414	3.874193776	-2.273778145	-7.345933108
	N415	2.745893344	0.503964874	-6.832298359
	N416	4.889378800	2.380725173	-6.548191539
	N417	0.241914416	-2.556927192	-10.329283578
	N418	-1.600232255	-0.773978011	-9.032849671
	N419	8.958688906	-7.099504139	-4.676395074
	N420	4.933441225	-5.470166622	-3.682255954
	N421	4.684794831	-6.346724992	-5.689793815
	N422	9.651996307	-7.432025773	-1.682707271
	O423	-6.782206609	-5.174510586	0.253561088
	O424	-8.010594268	-3.013348242	2.956410962

	O425	-6.547082431	-5.510977331	5.386085957
	O426	-3.305924251	-4.315668193	4.226909341
	O427	-3.890266318	-0.954755118	4.949668108
	O428	-4.933515544	-1.533003597	8.291311627
	O429	-2.426095725	-4.572175727	9.314372976
	O430	0.952340974	-3.920047003	6.250579216
	O431	0.616160989	0.143699250	6.959967984
	O432	-1.084640027	1.478843923	9.863240710
	O433	-0.875736225	-0.825370285	12.508979885
	O434	6.613643441	-10.521543066	2.857935823
	O435	6.915603782	-8.139735274	6.776081592
	O436	10.466974282	-4.760799611	6.367887253
	O437	-5.550376063	-0.759774163	-1.116641172
	O438	-2.862280190	-4.162491644	-4.845686836
	O439	-6.869196132	2.640243929	-1.634011467
	O440	-5.564021607	3.315173880	-4.369814276
	O441	-9.984021547	1.493710375	0.487875647
	O442	-6.215979639	1.201180971	3.342661043
	O443	-4.841713740	4.946891150	3.145200776
	O444	-7.380838817	7.114168046	4.412987938
	O445	9.919416742	-0.304538670	5.928790492
	O446	2.681617550	4.327803238	4.654597367
	O447	3.643700096	8.359042613	0.383237860
	O448	1.586753390	10.217627042	-2.869625664
	O449	-1.138161189	7.233222118	-1.484693225
	O450	-1.179950022	7.053665823	2.242256371
	O451	2.027800347	5.587564864	2.120765152
	O452	0.358450935	11.401313907	3.030131136
	O453	-2.526941914	13.603295502	0.864552423
	O454	-2.965778264	11.158723067	-1.393393487
	O455	-5.499267780	13.594869432	3.337994209
	O456	-5.113886110	7.023310802	-2.035067464
	O457	3.345547512	-4.228888374	-8.430564541
	O458	1.350779496	-0.382892691	-8.420660861
	O459	3.702274094	3.921474172	-7.720574820
	O460	7.275220917	4.766712605	-7.795798888
	O461	-3.220396813	-2.183397741	-9.798189981
	O462	-0.355049183	-6.341736314	-6.449314907
	O463	1.662158461	-5.456871211	-6.795597050
	O464	-2.274981942	2.730863248	-8.987422538
	O465	10.392771953	-5.332080228	-2.101759998
	O466	12.766988979	-6.807461867	-0.019447032
	O467	0.526861942	-6.350684771	-1.541251987
	O468	-0.372673951	-7.365578090	0.253341406
	O469	5.661865525	-10.785800372	-2.774041540
	O470	3.452152264	-10.785667779	-3.254433189
	O471	5.220060079	-5.822609985	-0.561393199
	O472	4.897129936	-7.604286282	0.771083939
	O473	2.917362212	-6.021651148	-1.766888712
	O474	8.609647234	-6.663035132	2.363791046
	O475	6.779104714	-4.624788290	1.469621513
	O476	-3.831238799	4.944130086	-3.009091839
	O477	8.073215714	-10.350602305	-1.514714446
	O478	7.398703149	-8.674481080	0.911364627
	S479	3.926575416	5.047021256	-1.498168294
	S480	2.801091971	2.120743189	-3.059593224
	S481	2.622283708	-3.161952377	-3.193148481
	S482	3.630999097	1.782032461	0.498818056
	S483	0.762364353	-0.817144603	-1.491190260
	S484	5.071262012	-0.627164335	-4.689978473
	S485	3.495508763	-3.529105423	0.352376177
	S486	6.186437930	1.823245179	-1.997821716
	S487	6.145604320	-3.012985529	-2.046525650
	S488	6.503819407	-0.834596508	0.663713704
	N489	1.027135267	1.932284738	-0.762211268
	N490	0.130826271	2.564987979	-0.493849976
	end			

S2BH-6H2x-7Hn-6Hn (9) to S2BH-6H2x-7H2n (10) (Scheme 3)

TS

	2n2x2bh6h2x6hnto7h2td_1_53159.00			
Fe(139) -2.336	C1	-7.825158916	-5.379483021	-0.366614760
Fe(140) -1.214	C2	-7.970215025	-6.484848746	-1.417778528
Fe(141) -2.433	C3	-8.944751796	-3.530365982	0.800220023
Fe(142) 2.634	C4	-8.578462100	-3.861499134	2.244705389
Fe(143) 2.766	C5	-8.533191228	-5.544158344	4.020919730
	C6	-7.022591496	-5.662734749	4.248408790
	C7	-4.813345966	-5.943705430	3.245580181

Fe(144) 1.312
Fe(145) 0.196

S=1/2

C8	-4.242857576	-4.537247844	3.440787980
C9	-4.459767447	-2.156881804	2.920845189
C10	-4.723081712	-1.658002999	4.348253783
C11	-6.225042156	-1.555043665	6.244483909
C12	-5.390412938	-2.210555455	7.353093282
C13	-4.611166406	-4.312711834	8.342632643
C14	-3.090479332	-4.511631058	8.270876440
C15	-1.142750200	-4.948537204	6.826941083
C16	-0.285715030	-3.787382088	6.317582601
C17	-0.303088601	-1.485562831	5.419688603
C18	-0.405412892	-0.312134969	6.408102003
C19	-0.731129849	-1.142316859	3.970195646
C20	-0.014921484	0.122191469	3.481668894
C21	-0.454534497	-2.322095663	3.031417903
C22	-1.842844737	1.312900048	7.552769320
C23	-1.781493938	0.869395974	9.026071204
C24	-3.128936250	2.097503245	7.200974603
C25	-3.386858366	3.209886551	8.223719200
C26	-3.024871406	2.679123193	5.785232569
C27	-2.532872828	-0.648779425	10.758342218
C28	-1.142140984	-0.886060728	11.312034089
C29	5.076815116	-11.837213628	4.147205999
C30	5.834316821	-10.553911986	3.830335431
C31	6.030606471	-8.171490369	4.513305687
C32	7.098118560	-7.857591901	5.577781532
C33	4.843489226	-7.198108859	4.616010359
C34	5.251523487	-5.751379100	4.349562939
C35	4.049495122	-4.819125020	4.214171301
C36	4.155386034	-2.313588051	4.184221458
C37	9.292167341	-6.833028853	5.964514573
C38	9.420444300	-5.330702176	6.089529761
C39	-4.956289334	-2.502359522	-2.735685506
C40	-5.395383926	-1.089824373	-2.306392441
C41	-3.467536697	-2.676374282	-2.360092981
C42	-2.921090328	-4.117580477	-2.410660383
C43	-2.375034601	-4.569974662	-3.765151991
C44	-5.674804394	1.203784486	-3.164327481
C45	-6.914326353	1.638963569	-2.371377376
C46	-5.578783041	1.882103536	-4.537287761
C47	-9.228057377	1.122237109	-1.750696210
C48	-9.098382856	1.011793119	-0.227194652
C49	-7.708646253	0.335562122	1.677016824
C50	-6.576291513	1.241437072	2.162348257
C51	-4.966142251	3.015941430	1.668348309
C52	-5.520028839	4.293487588	2.343538315
C53	-4.116845745	3.511086905	0.487140158
C54	-3.366768970	2.478078478	-0.286667102
C55	-2.958249510	1.194807653	-0.018701353
C56	-2.249929490	1.716226130	-2.065429416
C57	-7.314125703	5.979767035	2.257807685
C58	-7.437459953	6.073119101	3.777118263
C59	-8.703742581	6.078173833	1.583412904
C60	-9.275365016	7.457079722	1.425136975
C61	-9.351058903	8.511465431	2.314229444
C62	-10.288662286	9.061919927	0.383439341
C63	8.510304935	1.480420004	5.085761778
C64	9.583217902	0.410521265	5.001135912
C65	7.200420805	0.841578261	4.557181703
C66	6.016459942	1.776667513	4.615591796
C67	5.214837979	1.870673547	5.767043681
C68	5.671988432	2.569723459	3.508708971
C69	4.098130259	2.712879565	5.809112265
C70	4.569572542	3.424966537	3.539967951
C71	3.776881127	3.487296041	4.688383071
C72	5.526426551	6.904305991	-0.102902785
C73	4.510316401	8.011143130	-0.425838651
C74	4.810457568	5.563280828	0.053665146
C75	3.788583763	9.687784741	-2.091447882
C76	2.382419107	9.315371301	-2.566363771
C77	0.797972544	7.422722239	-2.916226486
C78	0.062653682	6.981642199	-1.624505059
C79	1.031166186	6.240540861	-3.888829566
C80	-0.154667490	5.359728020	-4.299527443
C81	-1.137079840	5.949311874	-5.322249278
C82	-2.724884198	7.851807481	-5.046794528
C83	0.144495190	5.913371861	0.573419215
C84	-0.197405306	7.092682970	1.495102064
C85	0.988776719	4.917870602	1.395715198
C86	0.746701319	9.078180950	2.566957334
C87	0.191097400	10.458057235	2.250620342

	C88	-0.865985184	11.883176396	0.571837550
	C89	-2.232369356	12.423787033	1.056629197
	C90	-0.737210678	11.988307470	-0.951251181
	C91	-1.777995377	11.190165256	-1.728884584
	C92	-4.417529231	11.886321792	2.032921466
	C93	-4.512089568	12.912956149	3.151266686
	C94	-5.175142812	10.589286484	2.439720279
	C95	-5.211783161	9.576309669	1.316758128
	C96	-6.215671816	9.645567411	0.338055698
	C97	-4.219426272	8.592657759	1.175578133
	C98	-6.215469320	8.787261804	-0.763115595
	C99	-4.189533001	7.739576397	0.067269801
	C100	-5.187723876	7.852883996	-0.895510796
	C101	5.674457372	-3.637149581	-8.226767231
	C102	4.188530528	-3.400372032	-8.023464741
	C103	2.522274107	-1.913207188	-6.933448904
	C104	2.137774145	-0.527564161	-7.462933809
	C105	2.548540173	1.885863722	-7.226126848
	C106	3.769424435	2.800277795	-7.196165346
	C107	5.991288729	3.326597354	-6.380720112
	C108	6.603476012	3.763469448	-7.694428130
	C109	-0.969684251	-3.072819353	-9.672235557
	C110	-2.031066501	-1.977755071	-9.515479598
	C111	-0.772933472	-3.807439063	-8.325830264
	C112	-0.031955158	-5.144514941	-8.520146863
	C113	0.396017511	-5.716277973	-7.186160405
	C114	-2.540747258	0.319070359	-8.856164205
	C115	-1.943280847	1.616253723	-9.365148612
	C116	-3.190522437	0.412601831	-7.453100797
	C117	-2.157661510	0.368061325	-6.359857797
	C118	-1.473944547	1.530319845	-5.964301898
	C119	-1.758481654	-0.867526857	-5.819899525
	C120	-0.377587342	1.446465477	-5.099974340
	C121	-0.661196414	-0.950275843	-4.958280379
	C122	0.050850417	0.204746829	-4.619218069
	C123	8.639432019	-6.183897675	-3.570136774
	C124	9.623275359	-6.271891119	-2.393038594
	C125	7.210644468	-6.389090732	-3.046977473
	C126	6.106997163	-6.161758899	-4.028610251
	C127	5.953814367	-6.710216623	-5.285003504
	C128	4.103615239	-5.627092711	-4.733532255
	C129	10.503843796	-7.516495656	-0.515309183
	C130	11.941722337	-7.179927796	-0.846488173
	C131	0.618742497	-6.905914557	-0.328191809
	C132	2.026796500	-7.017413425	0.244798129
	C133	3.161480352	-7.095759640	-0.818888755
	C134	3.177667259	-8.498300187	-1.474476988
	C135	4.400670742	-8.782808748	-2.349717918
	C136	4.504628764	-10.246760085	-2.826232721
	C137	4.525030174	-6.875126303	-0.137103379
	C138	3.925551357	-0.601629108	-1.550050655
	Fe139	4.384499616	-1.909838863	-2.911432532
	Fe140	2.440265286	0.853098164	-1.230179886
	Fe141	5.195403258	0.498414164	-0.503911594
	Fe142	4.522369875	0.615001212	-2.986488513
	Fe143	4.224483023	2.792871970	-1.400074358
	Fe144	4.639271542	-1.962443776	-0.305361344
	Fe145	2.328701683	-2.585091933	-1.078235440
	H146	-6.174492525	-7.401845085	-1.115846702
	H147	-6.168547389	-6.361264974	-2.373471497
	H148	-9.781447495	-4.819180644	-0.682501453
	H149	-8.556237776	-6.097750498	-2.266835820
	H150	-8.590381897	-7.275061168	-0.960050724
	H151	-8.221618783	-2.775250682	0.459414007
	H152	-9.362069382	-5.742596219	2.046573728
	H153	-9.941844049	-3.070221500	0.793710601
	H154	-8.993033834	-6.525706459	4.197270769
	H155	-6.691604196	-5.959576998	2.234246114
	H156	-8.912720043	-4.847297948	4.779501318
	H157	-3.392778804	-2.004922678	2.717827452
	H158	-5.585963041	-3.776265957	2.076161245
	H159	-4.405477552	-6.380983734	2.323722680
	H160	-6.060816447	-0.472060917	6.317531620
	H161	-6.612594649	-2.490865782	4.340186667
	H162	-4.472997788	-6.549923318	4.094127310
	H163	-5.036578815	-1.546874372	2.212472275
	H164	-5.697400556	-4.073040801	6.514104764
	H165	-7.286866975	-1.764341226	6.433466453
	H166	-0.961743496	-3.239085424	3.362818548
	H167	-0.810364692	-2.092985968	2.014297779

H168	0.625890339	-2.526474696	2.962468830
H169	-1.815464986	-0.947838154	3.983715844
H170	-5.076223307	-5.308894702	8.372322864
H171	-2.898240955	1.896684105	5.024340355
H172	-3.185869063	-0.648241429	8.731572510
H173	-3.549565626	2.815748848	9.237211582
H174	1.076461967	-0.026525039	3.455383027
H175	-0.224978293	0.995503849	4.116338329
H176	8.362560725	1.726125385	6.154709005
H177	-3.931599704	3.245925517	5.531259207
H178	-4.278240752	3.785175639	7.934381157
H179	-2.165990525	3.367254691	5.718541312
H180	-2.532503911	3.904179053	8.263823238
H181	-3.979432962	1.392267617	7.232166605
H182	-0.980890887	1.986494843	7.433989673
H183	-2.453554603	-0.182075988	6.125400952
H184	-0.336583169	0.368463790	2.458094206
H185	1.423273659	4.168296243	0.719273851
H186	0.295489838	4.408969467	2.083261006
H187	2.138884542	5.127100799	2.978021583
H188	2.191703686	4.268256546	5.498412407
H189	4.302174255	4.018751046	2.666057732
H190	6.255824054	2.493811712	2.589172564
H191	5.454204533	1.264503972	6.644761583
H192	-1.978082037	-2.705039807	5.974743873
H193	-1.028128197	-5.774683426	6.108953310
H194	-0.714888438	-5.274121745	7.784273303
H195	-3.153164672	-4.671768157	6.195945573
H196	-4.809382430	-3.810707936	9.297429318
H197	3.472674910	2.755539310	6.705176701
H198	0.763915967	-1.731880334	5.403508819
H199	-3.056881305	0.066155416	11.413543751
H200	-3.067416178	-1.610838455	10.805110684
H201	-0.377466440	-1.187808596	10.558154144
H202	10.252046374	-7.259234740	5.635096386
H203	-0.978346630	-5.802835659	-4.613572293
H204	1.542639118	-6.018568377	-1.769377324
H205	-0.703665197	-5.868428719	-9.003108888
H206	0.844317592	-4.999007207	-9.168535361
H207	-1.748767089	-4.003070111	-7.854780716
H208	-1.187508903	1.485236320	-10.174293828
H209	-1.435314211	-3.784735336	-10.370900057
H210	-2.863772478	-2.027252382	-3.015976861
H211	-3.712768729	-4.831383015	-2.123963319
H212	-0.621354095	-0.642326091	-8.770330736
H213	-3.362246248	0.108679395	-9.571979885
H214	0.814901228	-2.008361872	-9.677357293
H215	-0.898011292	-5.734487056	-2.857103770
H216	-0.219340181	-3.150257990	-7.637642826
H217	0.815080916	-3.336687807	-10.652060646
H218	-6.075174025	-3.184286943	-4.331567957
H219	-3.352973989	-2.311440500	-1.328686259
H220	2.183387108	-4.953957072	-7.492919997
H221	-2.133777284	-4.230269007	-1.651000328
H222	-4.339241023	2.521962509	2.423789191
H223	-3.107735463	0.566540195	0.850344458
H224	-8.617422385	0.648304072	2.210315986
H225	-7.292279399	-0.008606078	-0.384594565
H226	-7.257616606	4.077881204	1.272368460
H227	-8.994219925	8.637802296	3.330276985
H228	-7.632857761	5.091774543	4.273247349
H229	-9.410520519	5.417550951	2.115783256
H230	-6.664285584	6.797887396	1.908383010
H231	-10.800004875	9.668242589	-0.360611822
H232	-3.400132669	4.241701662	0.896431869
H233	-4.766011136	4.058835229	-0.213965111
H234	-10.205071355	10.447039749	1.998375611
H235	-8.603161665	5.668251014	0.566405292
H236	-6.334193748	2.099822717	0.277568178
H237	-7.485206016	-0.689926964	2.005209711
H238	-9.597865958	2.137156940	-1.951811119
H239	-1.764019060	1.650235016	-3.036481856
H240	-9.998650189	0.415146557	-2.083446295
H241	9.639613729	3.152312932	4.728774317
H242	8.114658392	3.256565479	4.148307958
H243	1.743994743	5.979566945	-0.878956131
H244	-0.810332216	5.427995840	0.325565486
H245	1.576115968	7.973215048	0.969673505
H246	-3.137154892	3.655934851	-2.087426694
H247	-4.495825725	4.396297138	-3.512970938

	H248	-5.729428646	3.701735329	-5.247537789
	H249	-4.352365539	5.591833436	-2.469367489
	H250	-4.648474745	1.542843187	-5.022822816
	H251	-6.435638429	1.585865392	-5.162152894
	H252	0.286196152	11.721186386	-1.251448252
	H253	-0.890539856	13.046182730	-1.216851073
	H254	-0.301105285	10.421468083	-2.973184804
	H255	-1.967024808	9.952979866	-3.314737766
	H256	-2.569110869	9.444681851	-6.330130472
	H257	-1.540193989	5.144332614	-5.956222971
	H258	-7.011678807	8.846022786	-1.509106360
	H259	-3.444357041	8.467673712	1.936147407
	H260	-3.965014718	8.018084622	-3.412163602
	H261	1.780963916	5.579629857	-3.423953904
	H262	2.859649181	7.342390189	-2.381140724
	H263	-0.704253297	4.995501045	-3.417087862
	H264	-7.947931121	0.028505778	-3.083558272
	H265	4.289632439	10.223144234	-2.908142565
	H266	-5.400804011	-0.692617689	-4.259355025
	H267	1.505808912	6.651895014	-4.794813421
	H268	-3.371533832	7.028105681	-0.056321999
	H269	3.650926153	10.395641971	-1.260672310
	H270	-1.795098261	7.996441008	-6.878925534
	H271	-4.232695075	9.179649373	-4.666790520
	H272	-4.424246765	-3.262486193	-4.572095049
	H273	-5.542662419	-3.197110130	-2.105009334
	H274	-7.016512668	10.382183464	0.435340469
	H275	-4.835210630	1.550378475	-2.546439819
	H276	0.174263649	8.198663929	-3.371318906
	H277	-0.598455403	6.641827966	-5.981532738
	H278	0.285273079	4.468548362	-4.769625683
	H279	-2.805038810	6.106438642	-4.000217160
	H280	0.177590185	8.683535554	3.421951771
	H281	-0.489992399	9.761696644	0.455017190
	H282	-0.142429541	12.589025411	1.007969654
	H283	1.789316745	9.216229493	2.885477635
	H284	-2.753123803	10.580311908	1.719308339
	H285	6.202365470	-2.673093862	-8.276240808
	H286	6.022157802	-4.143821201	-7.302463361
	H287	8.738769923	-5.156462815	-3.950213475
	H288	6.875874747	-4.735229986	-9.453121778
	H289	5.312695834	-5.223544856	-9.450552418
	H290	5.563112285	4.806100040	0.306794606
	H291	5.599947056	7.392189967	1.879031229
	H292	5.363255576	8.259038478	-2.281270958
	H293	4.070587539	5.625644061	0.864374775
	H294	6.817070626	8.053506637	1.006823357
	H295	6.777570323	2.818673346	-5.800598554
	H296	6.253003413	6.819524773	-0.926547865
	H297	4.380329147	-7.301909600	5.611811391
	H298	6.471966047	-8.093684896	3.511492074
	H299	4.922505292	1.510576923	-6.032497800
	H300	4.344932600	-0.302345113	3.890207966
	H301	2.151743838	1.890805427	-8.248706170
	H302	-0.346510746	-1.918614437	-4.562621423
	H303	1.788117266	2.366724938	-6.588082224
	H304	3.554124210	-4.690233721	5.184349953
	H305	3.271683740	0.275454417	-5.971494031
	H306	3.309309112	-5.242990483	3.515958098
	H307	4.101682266	-7.518922942	3.867075852
	H308	5.831332092	-5.706875567	3.416499631
	H309	5.903414109	-5.374729666	5.153036606
	H310	-3.893701189	-0.427267353	-7.354119976
	H311	4.912263849	-9.777674184	5.426302457
	H312	4.302417713	-11.933722960	3.371802486
	H313	0.928195943	0.154803996	-3.974571390
	H314	5.075465251	-12.204297282	6.174052742
	H315	3.616387573	-12.436112553	5.469393285
	H316	5.125748935	-3.551116145	2.906193999
	H317	0.170246782	2.343720964	-4.805640576
	H318	-2.300735503	-1.776457171	-6.089182146
	H319	7.826734505	-9.884603501	-0.677204906
	H320	7.114336595	-7.418853623	-2.662686055
	H321	6.506025503	-8.219838931	0.778791875
	H322	7.180522151	-10.443013775	-1.975832223
	H323	6.435524169	3.064169952	-8.547418503
	H324	6.289245649	-5.098037913	0.736391106
	H325	2.071226986	-7.880604562	0.919965534
	H326	4.627164979	-1.736144374	-6.918366431
	H327	3.141025706	-9.232284445	-0.655231188

H328	4.239490805	-6.647426423	-6.570108556
H329	3.090656595	-5.252432070	-4.797705401
H330	5.783243055	-12.670737853	3.992346907
H331	5.314550459	-1.248394337	2.816283621
H332	8.068030807	-5.877428611	2.063569537
H333	7.091043987	-3.803823250	1.030345863
H334	8.305074727	-7.054140004	4.074529983
H335	2.658901575	-2.932440475	5.518340112
H336	4.367313037	-8.141345003	-3.243793770
H337	5.322715434	-8.529102885	-1.809421233
H338	2.254129306	-8.617850833	-2.061435770
H339	2.458623854	-1.944076130	-5.834001686
H340	-1.784101398	2.495150283	-6.373184925
H341	-3.768185830	1.349172225	-7.428884221
H342	2.169980383	-6.113298717	0.861192618
H343	8.241359026	-7.403062663	1.804749249
H344	7.182848176	-9.335789258	1.603006280
H345	3.122864629	-1.263864149	5.608327950
H346	6.624042234	-7.320916289	-5.875818916
H347	7.054047910	-5.736475035	-2.180331963
H348	1.828896759	-2.643067109	-7.355650719
H349	5.680719910	4.216161187	-5.810744476
H350	6.997765685	-0.055781180	5.159994777
H351	7.362552059	0.516997597	3.516600728
H352	9.918022917	-6.929747938	-4.989816941
H353	8.932626384	-8.072262486	-4.353924614
H354	10.492009841	-8.563525682	-0.172101015
H355	9.018943763	-8.218970095	-1.873790538
H356	12.231322230	-7.366175230	-1.908992458
H357	10.154873308	-6.887859358	0.319008946
H358	10.045171823	0.304349769	3.989332497
H359	-3.616555112	12.974617715	3.813709862
H360	9.078673321	-7.224476878	6.975086395
H361	8.471060452	-4.763314905	5.924618416
H362	-4.897536719	12.320209723	1.140148565
H363	-4.696375083	10.157606445	3.332619531
H364	-6.196339623	10.882053508	2.723121983
H365	1.121569098	-3.641240222	-1.134943219
H366	3.937161128	-1.299288997	0.876403589
H367	-5.987647742	7.045494892	-2.477441636
H368	0.315871349	-0.663387348	-0.152395586
H369	2.792184398	-1.655215601	0.161289342
H370	1.109235684	-3.308350216	-0.333839323
H371	-1.779881595	-0.136494470	-1.236566010
Mo372	4.185848328	-4.369250376	-1.786811434
N373	-6.735508957	-7.072723377	-1.905839120
N374	-8.963245225	-4.676918657	-0.095094337
N375	-8.900346433	-5.100132777	2.686274824
N376	-6.265581004	-5.960198168	3.162646785
N377	-4.825049790	-3.550046200	2.713688876
N378	-5.899822260	-2.023904210	4.906588633
N379	-5.228711080	-3.556091567	7.263627994
N380	-2.553027043	-4.661419632	7.025624513
N381	-0.960622137	-2.673488376	5.945532372
N382	-1.649631886	0.184790522	6.641172544
N383	-2.513676610	-0.220608131	9.372630715
N384	4.436221973	-11.827324023	5.470581163
N385	5.552244365	-9.533619991	4.663142669
N386	4.491014838	-3.517440156	3.709467795
N387	3.264151054	-2.176914300	5.187256389
N388	4.744709914	-1.213062789	3.681325646
N389	8.204915224	-7.237504455	5.097547920
N390	-5.192718992	-2.694344108	-4.171316902
N391	-1.356841316	-5.450065874	-3.727433049
N392	-5.573108690	-0.235064985	-3.348068383
N393	-8.014766124	0.865529410	-2.508457347
N394	-7.995637640	0.375518796	0.256008668
N395	-6.005525472	2.085528415	1.247732681
N396	-2.912252788	2.767824319	-1.565493821
N397	-2.280321014	0.751234291	-1.138002084
N398	-6.741765266	4.686413342	1.902949496
N399	-9.864510387	7.813521357	0.222961040
N400	-9.995223750	9.522212670	1.632060213
N401	8.908406082	2.618114289	4.253081827
N402	6.272777456	7.195324635	1.131092707
N403	4.629410324	8.587419956	-1.659067106
N404	2.106736893	7.996546970	-2.623393105
N405	-2.266124298	6.649122411	-4.698608617
N406	-2.260695987	8.498531350	-6.131239401
N407	-3.614142245	8.488477662	-4.250080229

	N408	0.791558318	6.328795158	-0.674646819
	N409	0.699449835	8.119463345	1.477386578
	N410	-0.504017376	10.569026330	1.078234882
	N411	-1.309670402	10.577134721	-2.849462719
	N412	-3.027307467	11.559850917	1.736584095
	N413	5.912304948	-4.393237180	-9.458776719
	N414	3.870699000	-2.275600266	-7.349643002
	N415	2.746204049	0.502387263	-6.831148798
	N416	4.893416161	2.387484901	-6.558248062
	N417	0.239469051	-2.557518933	-10.326227165
	N418	-1.603566053	-0.774636201	-9.031748292
	N419	8.957926394	-7.100703356	-4.678069885
	N420	4.939557833	-5.473305117	-3.705583469
	N421	4.689801099	-6.358849981	-5.705800818
	N422	9.646019777	-7.431134631	-1.682583477
	O423	-6.764730745	-5.162700811	0.247442591
	O424	-8.012918437	-3.014854924	2.959756813
	O425	-6.550029689	-5.512220588	5.387383223
	O426	-3.308652050	-4.316010807	4.227080430
	O427	-3.893438224	-0.955935334	4.949493949
	O428	-4.936383276	-1.533740002	8.291993269
	O429	-2.429278574	-4.573566435	9.315114095
	O430	0.951049500	-3.919903401	6.250360763
	O431	0.615388582	0.138028808	6.952823857
	O432	-1.086064001	1.476476096	9.858123508
	O433	-0.879891676	-0.827770591	12.504072175
	O434	6.611287033	-10.510832982	2.854583716
	O435	6.914862606	-8.138083426	6.775009291
	O436	10.462305721	-4.758013440	6.367390684
	O437	-5.548225324	-0.759622435	-1.114428224
	O438	-2.864716229	-4.165960118	-4.844338348
	O439	-6.873058998	2.638515361	-1.632354276
	O440	-5.567889056	3.313744108	-4.368796015
	O441	-9.987413800	1.492026171	0.489042954
	O442	-6.219476938	1.199351069	3.343884371
	O443	-4.844146753	4.943603746	3.146519643
	O444	-7.382652122	7.112230325	4.411430845
	O445	9.916729426	-0.307744157	5.932395921
	O446	2.678316707	4.326653446	4.655761831
	O447	3.639665626	8.369993547	0.385788402
	O448	1.583868896	10.224030920	-2.869127629
	O449	-1.146312324	7.233817474	-1.483386132
	O450	-1.182704136	7.051786052	2.242406318
	O451	2.022624360	5.586850943	2.121257126
	O452	0.355866354	11.401518157	3.033733475
	O453	-2.536652812	13.609766258	0.867635404
	O454	-2.969900656	11.158068433	-1.391466483
	O455	-5.505383730	13.596134525	3.344504978
	O456	-5.117579187	7.022546522	-2.033718522
	O457	3.342083425	-4.232433091	-8.431263383
	O458	1.348656002	-0.382752434	-8.419160579
	O459	3.694959312	3.920941267	-7.727129966
	O460	7.279925278	4.772826960	-7.814389487
	O461	-3.222676116	-2.185684798	-9.796739031
	O462	-0.357473404	-6.343866021	-6.448443570
	O463	1.659686217	-5.459029474	-6.795658569
	O464	-2.279163601	2.729665609	-8.986741688
	O465	10.388092057	-5.331760794	-2.101498385
	O466	12.761127761	-6.808449538	-0.018038158
	O467	0.521125883	-6.207132539	-1.471104922
	O468	-0.371405903	-7.366003568	0.236726224
	O469	5.661272495	-10.795270163	-2.776149303
	O470	3.451577450	-10.794311413	-3.259071980
	O471	5.267848369	-5.914376906	-0.609189663
	O472	4.878137297	-7.625634204	0.794701204
	O473	2.936328078	-6.075191542	-1.793439066
	O474	8.600101223	-6.655284406	2.360184854
	O475	6.769610032	-4.619664247	1.459870528
	O476	-3.834916140	4.944860380	-3.010906276
	O477	8.067000899	-10.337319138	-1.516351687
	O478	7.389450344	-8.661094464	0.907289763
	S479	3.929967609	5.057140542	-1.495822460
	S480	2.825473733	2.103810405	-3.074236225
	S481	2.610554573	-3.256974113	-3.199204229
	S482	3.641470054	1.795443200	0.498759790
	S483	0.861633866	-0.846175623	-1.388005099
	S484	5.013972334	-0.705382490	-4.671468400
	S485	3.514321850	-3.768246776	0.369422469
	S486	6.195715911	1.802453933	-1.994021008
	S487	6.079279377	-2.970038635	-1.912342400

	S488	6.329500595	-0.889765238	0.751228599
	N489	1.038275923	1.940911534	-0.790511797
	N490	0.134297617	2.559938701	-0.511794234
	end			

S2BH-6H2x-7H2n (10) (Scheme 3)

	2bh6hx6h2nto7hntd.car_6			
Fe(139) -2.356	C1	-7.648656644	-5.439242451	2.147392344
Fe(140) -1.278	C2	-8.213475680	-6.570307941	1.281503360
Fe(141) -2.419	C3	-8.186332354	-3.469739819	3.510830410
Fe(142) 2.592	C4	-7.315628530	-3.732736264	4.736181851
Fe(143) 2.825	C5	-6.666086536	-5.282498488	6.509944236
Fe(144) 0.500	C6	-5.163093541	-5.355592822	6.228723179
Fe(145) 0.932	C7	-3.382378011	-5.712182245	4.601507717
	C8	-2.811911438	-4.297723887	4.474699804
	C9	-3.261318171	-1.972945859	3.860857822
	C10	-3.004548323	-1.347846826	5.240201474
	C11	-3.748519652	-1.089792178	7.537725779
**S=1/2**	C12	-2.608104838	-1.709029752	8.356647028
	C13	-1.627910938	-3.761042844	9.260800364
	C14	-0.237226322	-4.063780352	8.686141830
	C15	1.040454207	-4.740345433	6.693390673
	C16	1.751038847	-3.650880976	5.886360880
	C17	1.526413775	-1.619078176	4.499475498
	C18	1.929074753	-0.430065079	5.403703412
	C19	0.572565075	-1.193867075	3.362164981
	C20	1.137468899	0.007662779	2.596179379
	C21	0.304832784	-2.377605297	2.426352442
	C22	1.171836398	1.281476132	7.015668608
	C23	1.685289933	0.891394193	8.418111877
	C24	-0.084577650	2.185201625	7.067601210
	C25	0.099922041	3.330537367	8.071086684
	C26	-0.405105186	2.739302872	5.673335755
	C27	1.335652113	-0.330507170	10.489165637
	C28	2.790894160	-0.729452197	10.623073309
	C29	6.329549814	-11.666432375	2.918605256
	C30	6.817749400	-10.363017496	2.288048251
	C31	7.070199044	-7.922739171	2.732557388
	C32	8.376032290	-7.572269436	3.471141955
	C33	5.944650481	-6.938861447	3.095636777
	C34	6.281342145	-5.490499102	2.737983508
	C35	5.071591095	-4.570890460	2.902532517
	C36	5.164730500	-2.054105851	3.024380992
	C37	10.649725421	-6.695508029	3.230647311
	C38	10.916621796	-5.208259150	3.199585866
	C39	-5.585520461	-2.659300757	-0.941462508
	C40	-5.889443027	-1.236954989	-0.429373938
	C41	-4.051876457	-2.807260124	-1.064628554
	C42	-3.528555609	-4.243576687	-1.276183060
	C43	-3.425695073	-4.699389851	-2.732268654
	C44	-6.450938705	1.030681225	-1.226212471
	C45	-7.427594009	1.478476977	-0.130540774
	C46	-6.761119281	1.668097481	-2.588525878
	C47	-9.445834126	0.949703421	1.150403702
	C48	-8.868475884	0.941181676	2.570174439
	C49	-6.968266772	0.373257655	4.016278081
	C50	-5.765218321	1.311174678	4.102666978
	C51	-4.389677128	3.044021268	3.062818401
	C52	-4.763764262	4.331722129	3.838084192
	C53	-3.946134173	3.502249721	1.665865301
	C54	-3.434098592	2.451845042	0.734863929
	C55	-2.939484903	1.181718842	0.900266700
	C56	-2.846419083	1.662933543	-1.271589253
	C57	-6.558253196	5.976034356	4.235730755
	C58	-6.283702928	6.084064039	5.734679561
	C59	-8.077933541	6.045780312	3.951667367
	C60	-8.679606893	7.420075564	3.913167566
	C61	-8.486901826	8.512337041	4.737252736
	C62	-9.963364987	8.988163905	3.155249716
	C63	9.543465924	1.540133476	2.515072395
	C64	10.552401410	0.447082566	2.213702004
	C65	8.137518026	0.893986247	2.445137999
	C66	7.032125544	1.865800016	2.784150155
	C67	6.620900849	2.071149008	4.114897090
	C68	6.380025576	2.594518319	1.774668480
	C69	5.585805470	2.960040616	4.427564530
	C70	5.357914805	3.496774138	2.072875486
	C71	4.953975601	3.669162043	3.400168754
	C72	5.139648239	6.900940541	-1.804332412

C73	4.067960793	8.002279086	-1.878438741
C74	4.506972018	5.575871550	-1.381249536
C75	2.880148480	9.603987340	-3.333224058
C76	1.396656261	9.230018850	-3.320837456
C77	-0.216434719	7.342388313	-3.134096466
C78	-0.524866071	6.972009204	-1.661946373
C79	-0.288833110	6.105630772	-4.061116942
C80	-1.549099319	5.233140374	-4.046917521
C81	-2.779917631	5.784359910	-4.786639018
C82	-4.236364807	7.697038583	-4.110951322
C83	0.218419160	5.988154881	0.448564155
C84	0.156584355	7.199848563	1.390391594
C85	1.267128705	5.009693905	1.012500829
C86	1.337926878	9.239928980	2.051206093
C87	0.681770251	10.597576159	1.849827489
C88	-0.898096535	11.907733880	0.526508842
C89	-2.093750556	12.458772732	1.344512836
C90	-1.211690078	11.937272942	-0.971674773
C91	-2.409214536	11.085730706	-1.372404366
C92	-3.851801082	11.957884561	2.990485584
C93	-3.650103575	13.025396749	4.056855281
C94	-4.425409900	10.661158497	3.630413678
C95	-4.782141270	9.611478103	2.599952163
C96	-6.051756671	9.605393764	1.999076735
C97	-3.848464098	8.650945611	2.175792810
C98	-6.370097135	8.692821599	0.989719651
C99	-4.139039734	7.745484279	1.149954688
C100	-5.400963937	7.784932387	0.561179689
C101	3.006584905	-3.771195730	-9.307177175
C102	1.626702653	-3.555911047	-8.711520163
C103	0.294820258	-2.065018271	-7.233532946
C104	-0.226449949	-0.689721737	-7.664319457
C105	0.216418293	1.722279318	-7.627190490
C106	1.374410316	2.670988742	-7.921294967
C107	3.701992500	3.314328999	-7.660886965
C108	4.080848853	3.566488323	-9.107110805
C109	-3.802424637	-3.295442509	-8.748681783
C110	-4.785058766	-2.219921170	-8.273390661
C111	-3.185868388	-4.020028853	-7.529654577
C112	-2.524789922	-5.345428490	-7.951223083
C113	-1.716833152	-5.918798060	-6.810076467
C114	-5.115828103	0.091903080	-7.548859475
C115	-4.712774792	1.383140910	-8.233487616
C116	-5.295017735	0.202812006	-6.014261511
C117	-3.972683303	0.174872416	-5.296498703
C118	-3.196048043	1.339914161	-5.167567400
C119	-3.435018317	-1.048352077	-4.861725077
C120	-1.890893282	1.266994346	-4.670454231
C121	-2.130626800	-1.119860584	-4.362294882
C122	-1.346373649	0.034927888	-4.290026029
C123	7.235837017	-6.127354664	-5.736213907
C124	8.525076186	-6.218799445	-4.907126388
C125	6.018652788	-6.449920764	-4.857101548
C126	4.677682703	-6.233064937	-5.480662757
C127	4.199973961	-6.769704285	-6.658276907
C128	2.557227188	-5.712260485	-5.619937524
C129	9.921654455	-7.455734687	-3.367127534
C130	11.187855930	-7.172826435	-4.144759218
C131	0.468974563	-7.104270942	-0.375349708
C132	1.984647026	-7.178146430	-0.241093692
C133	2.770257903	-7.268208291	-1.581147270
C134	2.633536298	-8.678606664	-2.197943281
C135	3.540967927	-8.939918738	-3.402320467
C136	3.547515071	-10.413589416	-3.861684346
C137	4.260161559	-6.993712951	-1.307940747
C138	3.221279853	-0.642168478	-2.450450888
Fe139	3.124013030	-2.039458080	-3.828136515
Fe140	1.914259366	0.780398820	-1.746192401
Fe141	4.764216641	0.428483564	-1.876636867
Fe142	3.363756475	0.479456755	-4.047555902
Fe143	3.557619768	2.717859945	-2.529832119
Fe144	4.183207794	-2.029214768	-1.439756049
Fe145	1.663090235	-2.829377591	-1.566518343
H146	-6.468397971	-7.571950475	0.935539736
H147	-6.891693963	-6.564752148	-0.276882123
H148	-9.560705502	-4.784913858	2.544411773
H149	-9.055241808	-6.177023647	0.688803889
H150	-8.651364825	-7.314975091	1.968248705
H151	-7.620370845	-2.778579847	2.869270690
H152	-8.144081724	-5.588556024	4.977142284

	H153	-9.099062361	-2.955574481	3.841523013
	H154	-7.003589860	-6.259795277	6.879280798
	H155	-5.492067535	-5.863467063	4.251909662
	H156	-6.798836415	-4.550389898	7.318199713
	H157	-2.341831634	-1.849127178	3.277357017
	H158	-4.581704567	-3.645194495	3.667185705
	H159	-3.268460182	-6.232340169	3.640619100
	H160	-3.549896495	-0.013280294	7.464680729
	H161	-4.738002026	-2.187076161	5.970541565
	H162	-2.779260811	-6.238673846	5.351546761
	H163	-4.071367209	-1.432606047	3.351840173
	H164	-3.274891208	-3.613381907	7.898834439
	H165	-4.693286979	-1.238273539	8.078217565
	H166	-0.119138604	-3.239816936	2.958077940
	H167	-0.408927460	-2.091887490	1.637859911
	H168	1.234193544	-2.704117974	1.932699725
	H169	-0.376190945	-0.883502990	3.831397814
	H170	-2.081770024	-4.717544653	9.558864853
	H171	-0.587925041	1.938228546	4.943362556
	H172	0.058672249	-0.362957121	8.788249102
	H173	0.253571572	2.967760878	9.097605992
	H174	2.077723609	-0.246765902	2.083510783
	H175	1.332237561	0.869377377	3.250262682
	H176	9.713963068	1.869552443	3.558030558
	H177	-1.303713277	3.372978416	5.711455961
	H178	-0.791921777	3.974366045	8.069604203
	H179	0.430101991	3.358451633	5.306030873
	H180	0.967264696	3.952421361	7.797162258
	H181	-0.934835851	1.564497999	7.403506698
	H182	2.001259684	1.848614411	6.570374505
	H183	-0.013926045	-0.235424973	6.058973095
	H184	0.419535413	0.343969279	1.833639802
	H185	1.479408142	4.237280803	0.259226259
	H186	0.808728546	4.527077180	1.889868833
	H187	2.853492295	5.240688080	2.157250984
	H188	3.710273704	4.571827285	4.588264266
	H189	4.851768574	4.042946743	1.277134360
	H190	6.653221568	2.431844284	0.729024244
	H191	7.103051474	1.513445098	4.921991762
	H192	-0.049132731	-2.829029712	5.329348071
	H193	0.837030788	-5.579766614	6.010510712
	H194	1.750786316	-5.081475364	7.455314211
	H195	-1.042367742	-4.319298530	6.794047709
	H196	-1.465714691	-3.161488298	10.164836742
	H197	5.261681782	3.085718736	5.464347811
	H198	2.458678382	-1.981733160	4.053998060
	H199	1.131101467	0.504734269	11.179507030
	H200	0.731195499	-1.193687696	10.809823892
	H201	3.245072350	-1.193856699	9.717681660
	H202	11.466543330	-7.225337632	2.717298058
	H203	-2.342748276	-5.926122560	-3.966546841
	H204	0.889170784	-6.257785276	-2.051027209
	H205	-3.307662579	-6.069693429	-8.217760554
	H206	-1.883247730	-5.186630506	-8.829669231
	H207	-3.963117566	-4.229899411	-6.778964578
	H208	-4.252045181	1.241776565	-9.239009597
	H209	-4.447591644	-4.023433410	-9.264347351
	H210	-3.703797486	-2.162515655	-1.888622226
	H211	-4.180359762	-4.967185496	-0.756296135
	H212	-3.250803723	-0.836210328	-8.071611489
	H213	-6.114971388	-0.133800635	-7.974825807
	H214	-2.126435043	-2.204641314	-9.318440408
	H215	-1.740187956	-5.861259834	-2.318534704
	H216	-2.454152791	-3.353796513	-7.046519002
	H217	-2.410196088	-3.543706484	-10.236628377
	H218	-7.139824073	-3.395928416	-2.087157443
	H219	-3.611496764	-2.424116319	-0.131585244
	H220	-0.106964059	-5.167054317	-7.649871860
	H221	-2.541522755	-4.342568171	-0.799574226
	H222	-3.551831750	2.595759621	3.614460472
	H223	-2.822542716	0.576912779	1.789584486
	H224	-7.686708021	0.706759800	4.778362265
	H225	-7.171220087	-0.085650491	1.943859199
	H226	-6.726091580	4.056576910	3.299781158
	H227	-7.842658748	8.678056679	5.593135838
	H228	-6.337330233	5.106831481	6.273268561
	H229	-8.608990758	5.402401066	4.675513787
	H230	-6.037313929	6.800637366	3.723730487
	H231	-10.678716625	9.565554925	2.575189002
	H232	-3.157378372	4.257777134	1.812679565

H233	-4.790461681	4.012879048	1.175711797
H234	-9.407207475	10.438809766	4.610405644
H235	-8.251054856	5.602644805	2.958083448
H236	-6.078878130	2.042021982	2.175932554
H237	-6.633124530	-0.632642123	4.310723900
H238	-9.904756658	1.938410506	1.010465096
H239	-2.653313700	1.575562945	-2.338179048
H240	-10.246238619	0.200384404	1.098705879
H241	10.512409100	3.159620097	1.718633274
H242	8.885321484	3.240804154	1.586297852
H243	1.314603835	6.001872510	-1.413186355
H244	-0.765830333	5.501178030	0.508809605
H245	1.680860989	8.066042619	0.327797760
H246	-3.742559239	3.588789586	-1.080089897
H247	-5.472332515	4.244134154	-2.050798923
H248	-7.213399311	3.464705518	-3.234181361
H249	-5.049438215	5.478877509	-1.135535190
H250	-6.002055611	1.322696562	-3.309813876
H251	-7.754331738	1.345439925	-2.935706486
H252	-0.312956834	11.665690503	-1.542128988
H253	-1.464330523	12.977798144	-1.228405224
H254	-1.314721523	10.242936635	-2.919308593
H255	-2.986073829	9.713448095	-2.730879547
H256	-4.389991849	9.313782308	-5.365086134
H257	-3.329310996	4.961115113	-5.268197696
H258	-7.365767573	8.693934816	0.539157173
H259	-2.865227981	8.586533347	2.649453364
H260	-5.022445209	7.851265679	-2.210488347
H261	0.561247582	5.454280232	-3.798634577
H262	1.910884492	7.258233045	-3.274530071
H263	-1.824864200	4.951812510	-3.017962057
H264	-8.568476176	-0.186754513	-0.447954432
H265	3.099413858	10.074471548	-4.300826296
H266	-6.491079371	-0.897558938	-2.298298714
H267	-0.101842793	6.454926663	-5.089597206
H268	-3.374977090	7.051795543	0.798374962
H269	3.012917445	10.364215169	-2.550411841
H270	-3.762750890	7.879398534	-6.109905323
H271	-5.579925709	9.039447210	-3.343080840
H272	-5.649337327	-3.452622729	-2.842222023
H273	-5.924197543	-3.342192735	-0.140646216
H274	-6.810395124	10.320921439	2.323556240
H275	-5.478417545	1.407264246	-0.881136081
H276	-0.949807955	8.102019315	-3.420507313
H277	-2.450587270	6.459076361	-5.586727128
H278	-1.270923725	4.297280107	-4.551743611
H279	-4.049393881	5.951458518	-3.082593746
H280	1.060531770	8.883829848	3.054289686
H281	-0.539803622	9.788643387	0.425791215
H282	-0.102855326	12.647751966	0.707580891
H283	2.423956131	9.405899937	2.038368332
H284	-2.262208179	10.690730685	2.322375610
H285	3.488097736	-2.799146464	-9.489273461
H286	3.599736822	-4.278008160	-8.517960895
H287	7.176466329	-5.077674194	-6.059545190
H288	3.841304233	-4.854850275	-10.819057117
H289	2.337681896	-5.351547224	-10.402591917
H290	5.308884044	4.831457930	-1.298829079
H291	5.769257978	7.506033342	0.044336596
H292	4.332369894	8.147642251	-3.914087469
H293	4.019674497	5.693958715	-0.402630877
H294	6.689747254	8.093864549	-1.173397146
H295	4.590494283	2.916124460	-7.147308730
H296	5.604039758	6.771340393	-2.794505297
H297	5.728160619	-7.028673906	4.173187334
H298	7.236034829	-7.899177610	1.648219799
H299	2.815303754	1.493584523	-6.982106137
H300	5.366747724	-0.029177674	2.821087669
H301	-0.416253765	1.691475748	-8.523003814
H302	-1.715120127	-2.080440858	-4.048723145
H303	-0.382709002	2.205320916	-6.836653107
H304	4.765589928	-4.517835001	3.953989010
H305	1.281205118	0.146086897	-6.579048084
H306	4.215202979	-4.970326962	2.334305183
H307	5.046008720	-7.260778618	2.545729737
H308	6.613762483	-5.436875525	1.690371173
H309	7.107828404	-5.115071520	3.362685102
H310	-5.924996052	-0.638872551	-5.692240772
H311	6.341370452	-9.488687080	4.023967812
H312	5.367379486	-11.894666230	2.435197825

	H313	-0.316732735	-0.006816844	-3.937435895
	H314	6.987393588	-11.802461752	4.864644424
	H315	5.424661731	-12.249864685	4.671351424
	H316	5.654596633	-3.179463332	1.418134230
	H317	-1.273103661	2.163721360	-4.588987140
	H318	-4.035311419	-1.958690428	-4.925661742
	H319	7.306887376	-9.871606905	-2.702895770
	H320	6.071502435	-7.506956280	-4.544656447
	H321	6.485961253	-8.214462224	-0.960260757
	H322	6.337070006	-10.487223708	-3.760153063
	H323	3.763580864	2.772153687	-9.824458077
	H324	6.214286479	-5.149450819	-1.061640808
	H325	2.245337279	-8.012435796	0.421340129
	H326	2.325658667	-1.882369066	-7.771023427
	H327	2.870594392	-9.399389602	-1.400054114
	H328	2.202416293	-6.720583615	-7.433816300
	H329	1.560805197	-5.345241168	-5.411380706
	H330	7.036539172	-12.453024618	2.604780457
	H331	5.672814644	-0.918788568	1.348223004
	H332	8.355429622	-5.819036291	-0.263440558
	H333	6.987147518	-3.801721427	-1.040766825
	H334	9.188412442	-6.916054945	1.666224659
	H335	4.449650280	-2.796541567	4.802746755
	H336	3.208318290	-8.321373106	-4.250760098
	H337	4.573977650	-8.644189501	-3.173313774
	H338	1.580786439	-8.840077647	-2.477725245
	H339	0.525649067	-2.072510988	-6.156186442
	H340	-3.611470511	2.296302876	-5.494196487
	H341	-5.842832989	1.138433632	-5.824176696
	H342	2.276508227	-6.247570308	0.276233687
	H343	8.465054275	-7.349776704	-0.492044073
	H344	7.412126433	-9.267288951	-0.320490130
	H345	4.408552930	-1.107182497	4.666404765
	H346	4.690551386	-7.372559261	-7.412698000
	H347	6.079984118	-5.862005193	-3.934241609
	H348	-0.480209941	-2.804846018	-7.443010493
	H349	3.443132118	4.273740258	-7.186543532
	H350	8.123886987	0.044869924	3.144428596
	H351	7.985684711	0.495207663	1.429381060
	H352	8.090635448	-6.679833338	-7.515072024
	H353	7.439137112	-7.941121028	-6.701156816
	H354	9.990396748	-8.491026214	-2.998083559
	H355	8.071108741	-8.136675199	-4.179654462
	H356	11.133355988	-7.411719397	-5.234364508
	H357	9.870787292	-6.792227441	-2.489196981
	H358	10.684256455	0.232270802	1.125373452
	H359	-2.589834655	13.200498425	4.356574354
	H360	10.658243077	-7.003469327	4.291551295
	H361	10.006742323	-4.561398041	3.233967471
	H362	-4.580349479	12.353426436	2.263578315
	H363	-3.686197639	10.260758891	4.342366086
	H364	-5.314540731	10.949321516	4.208829175
	H365	0.455546580	-3.839875066	-1.140927609
	H366	3.982320140	-1.305637922	0.073106383
	H367	-6.637452898	6.897639122	-0.654689601
	H368	0.204284989	-0.811924929	-0.033544305
	H369	3.215424343	-1.338354060	-0.213815520
	H370	0.713773219	-3.498823992	-0.409376076
	H371	-2.108743012	-0.155267527	-0.579741078
	Mo372	3.349841920	-4.501250354	-2.736393932
	N373	-7.270272939	-7.233726190	0.397618279
	N374	-8.578186049	-4.663480127	2.778370499
	N375	-7.471303772	-4.925361891	5.354264562
	N376	-4.787184098	-5.728466626	4.978808123
	N377	-3.633943334	-3.376514084	3.920910701
	N378	-3.893382789	-1.663170236	6.209221339
	N379	-2.559891064	-3.065187655	8.385723237
	N380	-0.190156928	-4.346898024	7.355526534
	N381	0.965203681	-2.720778661	5.286811297
	N382	0.942094020	0.114580598	6.157997930
	N383	0.965104776	-0.021351807	9.117598447
	N384	6.123620912	-11.569487709	4.369456155
	N385	6.674299944	-9.279569102	3.077303240
	N386	5.358555374	-3.224239015	2.395919848
	N387	4.853584672	-1.979724208	4.332815131
	N388	5.296080680	-0.911935554	2.319121521
	N389	9.355663546	-7.057339964	2.687175509
	N390	-6.261599418	-2.891707485	-2.222652717
	N391	-2.433722943	-5.565844870	-3.010482118
	N392	-6.377528182	-0.411050802	-1.392487383

	N393	-8.494713054	0.676319887	0.086007437
	N394	-7.661124946	0.332061542	2.742095115
	N395	-5.477082956	2.076003358	3.004376439
	N396	-3.361720034	2.716453659	-0.626016293
	N397	-2.595669585	0.719167410	-0.355855839
	N398	-6.069683633	4.691010608	3.748280665
	N399	-9.604454529	7.730111291	2.929253715
	N400	-9.310891710	9.498137663	4.237244204
	N401	9.680971222	2.600167387	1.513807582
	N402	6.204221789	7.252406749	-0.848943291
	N403	3.812469021	8.511533315	-3.119831365
	N404	1.118743675	7.910057061	-3.286570245
	N405	-3.714188676	6.489525862	-3.901396575
	N406	-4.054690270	8.361059338	-5.266691322
	N407	-4.898245345	8.322354888	-3.109904444
	N408	0.464082374	6.359738066	-0.947282774
	N409	0.994601700	8.226063211	1.070541091
	N410	-0.366741861	10.632189231	0.972033208
	N411	-2.256142294	10.396221335	-2.535457353
	N412	-2.599579845	11.649773305	2.308712326
	N413	2.910260982	-4.518152249	-10.564167104
	N414	1.485825127	-2.427971600	-7.986912518
	N415	0.536122551	0.353311556	-7.263549310
	N416	2.627851095	2.342856905	-7.518064289
	N417	-2.866971806	-2.770419865	-9.749360547
	N418	-4.260015931	-0.993762220	-7.990750070
	N419	7.282248656	-6.959919006	-6.952242519
	N420	3.634116557	-5.556767334	-4.847733052
	N421	2.866612926	-6.430804902	-6.721494002
	N422	8.740144544	-7.362476721	-4.204049114
	N423	-0.083111256	2.469601081	-0.357724398
	N424	0.700054153	1.862269985	-0.901630321
	O425	-6.430290217	-5.250687483	2.311198181
	O426	-6.509688689	-2.870693410	5.132524466
	O427	-4.343714583	-5.098836482	7.126546480
	O428	-1.659913000	-4.019448879	4.858071226
	O429	-2.031136690	-0.600261322	5.431846812
	O430	-1.812994519	-0.989798713	8.986468977
	O431	0.746208882	-4.094635839	9.437192699
	O432	2.990905861	-3.680914531	5.782968504
	O433	3.097201850	0.005875038	5.410448683
	O434	2.711559530	1.412630335	8.886573775
	O435	3.413174565	-0.621626667	11.669621047
	O436	7.270576596	-10.364587217	1.125064279
	O437	8.484069080	-7.760124497	4.695816784
	O438	12.036898507	-4.723581856	3.157012000
	O439	-5.685757327	-0.876270546	0.746332900
	O440	-4.231537873	-4.308242506	-3.607180748
	O441	-7.212535294	2.512634436	0.526483109
	O442	-6.728598370	3.104263348	-2.470545408
	O443	-9.512609889	1.465341012	3.487879217
	O444	-5.098621574	1.359935712	5.142111342
	O445	-3.907624916	5.014567607	4.408589732
	O446	-6.068804320	7.130041079	6.321450109
	O447	11.167171970	-0.177534538	3.065807711
	O448	3.909392107	4.543825533	3.634564102
	O449	3.474073927	8.418287241	-0.867687431
	O450	0.537802902	10.132673853	-3.353331481
	O451	-1.638173660	7.230086904	-1.177865507
	O452	-0.567736004	7.187711926	2.393751892
	O453	2.466367914	5.695233437	1.381316055
	O454	1.080085889	11.589483931	2.472335994
	O455	-2.506674185	13.609993121	1.145910580
	O456	-3.460787564	11.074226475	-0.716433254
	O457	-4.575568774	13.633751917	4.569604544
	O458	-5.671386341	6.905038085	-0.508564246
	O459	0.711356470	-4.399599392	-8.875774281
	O460	-1.257668206	-0.563366778	-8.355994902
	O461	1.128205257	3.750296524	-8.485116928
	O462	4.717697348	4.541800209	-9.469889797
	O463	-5.994802608	-2.466522038	-8.134177509
	O464	-2.216274678	-6.529877857	-5.870182595
	O465	-0.391003974	-5.680360608	-6.831145007
	O466	-4.907476740	2.500884051	-7.778025268
	O467	9.367650407	-5.299940920	-4.904375822
	O468	12.228365620	-6.788176125	-3.629499835
	O469	0.019882968	-6.457942684	-1.465492659
	O470	-0.299738178	-7.538800537	0.477618198
	O471	4.686825985	-10.939301483	-4.118797338
	O472	2.429992846	-10.992606077	-3.976453388

	O473	4.789171247	-5.983539163	-1.936825087
	O474	4.894479018	-7.739528692	-0.536562195
	O475	2.254755316	-6.265562111	-2.465686042
	O476	8.957183867	-6.583088155	-0.085502982
	O477	6.916816223	-4.654220893	-0.569135080
	O478	-4.705099032	4.822312734	-1.791312910
	O479	7.311489878	-10.335116490	-3.569978093
	O480	7.386260760	-8.626736434	-1.075056908
	S481	3.241558323	4.984254461	-2.600701695
	S482	1.709029864	1.981029209	-3.656050700
	S483	1.400761764	-3.481537914	-3.663232403
	S484	3.582555456	1.785614536	-0.513087142
	S485	0.359509867	-0.935065769	-1.373844572
	S486	3.235216273	-0.903383712	-5.730488516
	S487	3.279003777	-3.836599060	-0.472122554
	S488	5.273936172	1.660316833	-3.639579596
	S489	5.096946464	-3.000342082	-3.292453025
	S490	6.137187858	-0.981033224	-0.873359738
	end			

S2BH-6Hx-7Hn-6H2n transverse (8) to S2BH-6H2x-7H2n (10) (Scheme 3)

TS

Fe(139) -2.284	2bh6hx6h2nto7hnte.car_1			
Fe(140) -1.194	C1	-7.672726970	-5.442479303	2.140549288
Fe(141) -2.467	C2	-8.256512658	-6.561801028	1.271658214
Fe(142) 2.585	C3	-8.178856839	-3.463207944	3.499134572
Fe(143) 2.777	C4	-7.310928987	-3.728646164	4.725598723
Fe(144) 1.090	C5	-6.663685958	-5.277725314	6.499839992
Fe(145) 0.370	C6	-5.160311557	-5.343905869	6.219820992
	C7	-3.373110142	-5.694130499	4.599287928
	C8	-2.806644351	-4.277604464	4.476198285
**S=1/2**	C9	-3.263274224	-1.956282405	3.847916925
	C10	-3.012496356	-1.322599193	5.224103698
	C11	-3.747874985	-1.072426081	7.523793476
	C12	-2.605114435	-1.691777086	8.339665139
	C13	-1.628607252	-3.746090273	9.244469664
	C14	-0.235884671	-4.054943098	8.677814515
	C15	1.051608260	-4.746078378	6.695476652
	C16	1.762619705	-3.663324265	5.879130233
	C17	1.529240382	-1.615881073	4.508201400
	C18	1.938288119	-0.430743372	5.414551881
	C19	0.553051732	-1.191970075	3.390530893
	C20	1.083555147	0.021585002	2.618407456
	C21	0.276842786	-2.372868282	2.454614061
	C22	1.184463454	1.291612095	7.019535825
	C23	1.693931779	0.902671702	8.422879775
	C24	-0.071957926	2.196406873	7.071695533
	C25	0.111300809	3.341239635	8.076155942
	C26	-0.392779633	2.751355700	5.677978742
	C27	1.337006202	-0.318409731	10.490854886
	C28	2.799352642	-0.685563869	10.641495036
	C29	6.332933966	-11.676893161	2.953069846
	C30	6.809594415	-10.375577253	2.311575100
	C31	7.066874883	-7.934098565	2.746118593
	C32	8.373452637	-7.580584718	3.482043564
	C33	5.938988278	-6.949524154	3.102803703
	C34	6.271816155	-5.501111931	2.740110942
	C35	5.052149574	-4.589245014	2.876500641
	C36	5.141725363	-2.069899336	2.976430219
	C37	10.649207667	-6.711534651	3.236103374
	C38	10.922494620	-5.225297028	3.206358687
	C39	-5.596499255	-2.672230057	-0.929984183
	C40	-5.902965346	-1.252627640	-0.413840889
	C41	-4.063412228	-2.816405179	-1.051582443
	C42	-3.534197694	-4.249973683	-1.264375400
	C43	-3.430957134	-4.703063794	-2.720886461
	C44	-6.458414108	1.017991618	-1.208170498
	C45	-7.437705272	1.463374332	-0.113861314
	C46	-6.764623206	1.656467954	-2.570911746
	C47	-9.452752356	0.921558462	1.168160689
	C48	-8.873214631	0.921749496	2.587253763
	C49	-6.968117993	0.372616862	4.033886970
	C50	-5.769553265	1.316739414	4.116707783
	C51	-4.388248050	3.037215918	3.064025718
	C52	-4.766793212	4.323855105	3.837430616
	C53	-3.952395074	3.497712666	1.665035855

	C54	-3.441259817	2.446877522	0.734960927
	C55	-2.979619489	1.165660815	0.902923975
	C56	-2.858126096	1.647224177	-1.269263658
	C57	-6.565631893	5.964130472	4.221038749
	C58	-6.312232667	6.072363695	5.724455665
	C59	-8.082033468	6.032871096	3.920235905
	C60	-8.689329027	7.404897000	3.885168275
	C61	-8.490689230	8.501020818	4.702759725
	C62	-9.993889816	8.962468174	3.141479341
	C63	9.524896720	1.525248043	2.522065617
	C64	10.530228884	0.428840334	2.222741089
	C65	8.115941780	0.882612821	2.452475158
	C66	7.014034656	1.857989113	2.793055685
	C67	6.606557301	2.064456455	4.124687378
	C68	6.364485216	2.591795508	1.785389049
	C69	5.578629661	2.960264083	4.440288667
	C70	5.348380996	3.500181694	2.086505796
	C71	4.949453089	3.674852329	3.415039270
	C72	5.137394613	6.867910537	-1.788777203
	C73	4.066797884	7.968693898	-1.871552823
	C74	4.505726680	5.550983674	-1.340378129
	C75	2.892949319	9.573519869	-3.332009754
	C76	1.408495627	9.202679585	-3.315433525
	C77	-0.207974386	7.319703053	-3.132480200
	C78	-0.519999256	6.958289824	-1.658011860
	C79	-0.282527522	6.078879175	-4.054252222
	C80	-1.546289270	5.212102763	-4.038328796
	C81	-2.774574194	5.768499672	-4.778176218
	C82	-4.232302026	7.681282285	-4.095119325
	C83	0.221121501	5.989041508	0.461379180
	C84	0.177472295	7.207131936	1.395864403
	C85	1.263385226	5.003427046	1.025865872
	C86	1.371967813	9.247957737	2.028599126
	C87	0.700217166	10.597680857	1.825349004
	C88	-0.894030640	11.887477773	0.503876148
	C89	-2.085419607	12.438053603	1.328722033
	C90	-1.223249540	11.912550223	-0.991267247
	C91	-2.414974782	11.046831659	-1.378020616
	C92	-3.842748097	11.944833839	2.975380581
	C93	-3.637116000	13.013833928	4.039506212
	C94	-4.422578084	10.652032769	3.618238839
	C95	-4.783596748	9.600443583	2.591585420
	C96	-6.055487534	9.592885285	1.995611378
	C97	-3.851989254	8.638163685	2.166720235
	C98	-6.378825306	8.676509420	0.991104252
	C99	-4.147138496	7.729511698	1.144908069
	C100	-5.411986299	7.766442336	0.562329155
	C101	3.008324202	-3.750708531	-9.329071978
	C102	1.634930821	-3.533713094	-8.719960437
	C103	0.314310740	-2.054365560	-7.223450310
	C104	-0.223737774	-0.687643795	-7.661178597
	C105	0.203095904	1.728736204	-7.639304255
	C106	1.355137286	2.691906170	-7.908858075
	C107	3.684894133	3.331882758	-7.623801742
	C108	4.137079977	3.532220686	-9.055639679
	C109	-3.819102062	-3.300980767	-8.748911895
	C110	-4.803679697	-2.229392884	-8.268329774
	C111	-3.193354387	-4.022221483	-7.533213555
	C112	-2.524517881	-5.343822350	-7.954980064
	C113	-1.706683893	-5.906048772	-6.815230551
	C114	-5.133580254	0.078812103	-7.530417040
	C115	-4.729500618	1.373455230	-8.208139170
	C116	-5.321556627	0.183249343	-5.996511192
	C117	-3.996276015	0.161554702	-5.287809599
	C118	-3.233687724	1.334553872	-5.153584430
	C119	-3.432375199	-1.064213673	-4.891498532
	C120	-1.911077148	1.268514896	-4.704899786
	C121	-2.109859850	-1.128686935	-4.444344468
	C122	-1.336524952	0.034217202	-4.382617059
	C123	7.237083054	-6.115726014	-5.736196760
	C124	8.525308277	-6.223738694	-4.905790193
	C125	6.016990746	-6.495879566	-4.887911131
	C126	4.674946787	-6.252322605	-5.501744656
	C127	4.172646211	-6.786877884	-6.670064873
	C128	2.557155911	-5.706330028	-5.610100895
	C129	9.916823659	-7.480905864	-3.373530698
	C130	11.179534532	-7.213170803	-4.162036651
	C131	0.480447885	-7.025558310	-0.372616126
	C132	1.997951575	-7.095036718	-0.248011553
	C133	2.766686617	-7.188397981	-1.597868634

	C134	2.604255540	-8.599584521	-2.209456923
	C135	3.510830877	-8.890188825	-3.407959795
	C136	3.505643369	-10.371425174	-3.841522448
	C137	4.267515086	-6.955305012	-1.347418842
	C138	3.212472450	-0.672293535	-2.449152755
	Fe139	3.271183854	-1.987560490	-3.884081460
	Fe140	1.851681890	0.725639526	-1.708418806
	Fe141	4.740713074	0.421173207	-1.837471369
	Fe142	3.332513523	0.538102387	-3.994779380
	Fe143	3.498946937	2.698994039	-2.399348068
	Fe144	4.325466535	-2.076600798	-1.522480266
	Fe145	1.893976745	-2.608827571	-1.514208897
	H146	-6.527747611	-7.586920673	0.918773043
	H147	-6.933189156	-6.562997900	-0.284495018
	H148	-9.574307311	-4.761665635	2.540062569
	H149	-9.094650495	-6.154506225	0.683487961
	H150	-8.702467641	-7.304613531	1.955179182
	H151	-7.600363720	-2.789265026	2.850626622
	H152	-8.144319404	-5.581720359	4.968709434
	H153	-9.081285321	-2.928902916	3.825765671
	H154	-6.998047940	-6.257248254	6.865851127
	H155	-5.482183954	-5.857109249	4.242909828
	H156	-6.799286319	-4.548624733	7.310415249
	H157	-2.342613714	-1.832361668	3.267129640
	H158	-4.572923609	-3.635184804	3.655576061
	H159	-3.253448958	-6.212284936	3.638125794
	H160	-3.548994510	0.003719293	7.448434782
	H161	-4.732420602	-2.183525865	5.962388952
	H162	-2.771671482	-6.220223072	5.350968941
	H163	-4.076396877	-1.423970565	3.334593593
	H164	-3.270712182	-3.595353647	7.878074066
	H165	-4.691378807	-1.220180201	8.066548902
	H166	-0.189174412	-3.217457543	2.979024396
	H167	-0.399292226	-2.071943250	1.638855742
	H168	1.206250710	-2.731916965	1.988008091
	H169	-0.388742598	-0.891870594	3.879775929
	H170	-2.087328054	-4.700587076	9.541889009
	H171	-0.576143334	1.950926302	4.947753971
	H172	0.071405152	-0.357573810	8.783216817
	H173	0.263479072	2.978505080	9.102933126
	H174	1.986395161	-0.225328117	2.039719008
	H175	1.326168907	0.866655618	3.279279614
	H176	9.695463492	1.856578267	3.564081975
	H177	-1.291496175	3.384672489	5.717427509
	H178	-0.780785497	3.984717408	8.073500824
	H179	0.442229212	3.370865334	5.311052771
	H180	0.978766313	3.963638166	7.803826575
	H181	-0.922152071	1.575327486	7.407473959
	H182	2.014693656	1.857795671	6.573678917
	H183	-0.006983362	-0.216257994	6.059130623
	H184	0.318619917	0.378553641	1.913090356
	H185	1.467122342	4.227547827	0.273344937
	H186	0.805792850	4.526630446	1.906465994
	H187	2.846161701	5.238482166	2.176903454
	H188	3.721099217	4.591180232	4.608733851
	H189	4.844496189	4.052034353	1.293414941
	H190	6.636017737	2.429646399	0.739172218
	H191	7.086319448	1.502240151	4.929954293
	H192	-0.036411721	-2.819521115	5.360875717
	H193	0.852127779	-5.591576156	6.019325618
	H194	1.760208797	-5.080711201	7.461627114
	H195	-1.031318654	-4.324418791	6.783775887
	H196	-1.468110700	-3.145964292	10.148461565
	H197	5.258123927	3.087687277	5.477984960
	H198	2.455792911	-1.976203127	4.050675234
	H199	1.107577452	0.511404781	11.181615569
	H200	0.748219221	-1.195433940	10.804232119
	H201	3.269792857	-1.151064900	9.744472173
	H202	11.463554960	-7.244209904	2.720975256
	H203	-2.334476672	-5.913708627	-3.957907720
	H204	0.885129302	-6.134386850	-2.024362980
	H205	-3.302541211	-6.075575652	-8.214648328
	H206	-1.889013027	-5.182892599	-8.837469326
	H207	-3.965609310	-4.236403536	-6.778677599
	H208	-4.263223859	1.236161431	-9.211792620
	H209	-4.463646814	-4.030632540	-9.263403829
	H210	-3.717449686	-2.169813337	-1.875243267
	H211	-4.184025647	-4.977007924	-0.746759027
	H212	-3.269872369	-0.847656687	-8.058208986
	H213	-6.132053028	-0.142409537	-7.961724112

	H214	-2.144842189	-2.209037516	-9.319601466
	H215	-1.717217635	-5.823987706	-2.315054896
	H216	-2.463553357	-3.350271148	-7.055021344
	H217	-2.435543228	-3.541141452	-10.244981981
	H218	-7.151480848	-3.401577720	-2.078592715
	H219	-3.626258538	-2.434906467	-0.116423479
	H220	-0.105454696	-5.142212393	-7.663510662
	H221	-2.546422841	-4.344218530	-0.788906502
	H222	-3.546324638	2.593838618	3.613728728
	H223	-2.881493067	0.560017597	1.793839728
	H224	-7.688221013	0.704920800	4.794787374
	H225	-7.169562570	-0.096634606	1.963048764
	H226	-6.725096284	4.043832349	3.288487139
	H227	-7.836112389	8.672116429	5.549953502
	H228	-6.381455250	5.095903552	6.263491159
	H229	-8.619063996	5.384136957	4.635085663
	H230	-6.038181321	6.788788294	3.716147839
	H231	-10.721123383	9.533937161	2.570468836
	H232	-3.168815300	4.259175890	1.807544265
	H233	-4.803505735	4.000759161	1.178215767
	H234	-9.425429643	10.421172637	4.583736235
	H235	-8.243925908	5.593838967	2.922987736
	H236	-6.075466204	2.027018439	2.180945538
	H237	-6.627642824	-0.630508069	4.331883276
	H238	-9.922062509	1.905187995	1.027151959
	H239	-2.662417751	1.555948415	-2.335343745
	H240	-10.245195679	0.163631451	1.118419606
	H241	10.500930693	3.136786663	1.716121302
	H242	8.873573157	3.228796670	1.593354457
	H243	1.318147053	5.985469564	-1.398383492
	H244	-0.768250048	5.513680840	0.530198364
	H245	1.702916323	8.054450498	0.316782435
	H246	-3.724383677	3.587848722	-1.079994515
	H247	-5.471053936	4.226544511	-2.043307747
	H248	-7.227119250	3.452705333	-3.210952603
	H249	-5.051098951	5.462664538	-1.129377317
	H250	-6.002377768	1.312772034	-3.289752919
	H251	-7.755764862	1.332118363	-2.922151754
	H252	-0.327682334	11.650645451	-1.571054474
	H253	-1.491227768	12.950490142	-1.243235514
	H254	-1.310891882	10.184600063	-2.904570441
	H255	-2.972953017	9.634265833	-2.699502168
	H256	-4.299551522	9.334704508	-5.311590373
	H257	-3.325289525	4.948565478	-5.263502104
	H258	-7.376273822	8.676294639	0.544295815
	H259	-2.866878671	8.575079436	2.636558788
	H260	-5.039384335	7.813385284	-2.201480555
	H261	0.564482944	5.425099906	-3.787907148
	H262	1.918624081	7.229818042	-3.276015325
	H263	-1.822263118	4.933819790	-3.008737844
	H264	-8.568693293	-0.207518540	-0.431355737
	H265	3.112754417	10.038498285	-4.302350267
	H266	-6.494775055	-0.908822594	-2.283705244
	H267	-0.091986849	6.422456835	-5.084116714
	H268	-3.384427483	7.034917859	0.791849888
	H269	3.028961864	10.336718511	-2.552666560
	H270	-3.646924205	7.912168054	-6.060970365
	H271	-5.575764300	9.019950189	-3.323171675
	H272	-5.659010852	-3.465966323	-2.830772668
	H273	-5.935935738	-3.358591591	-0.131757019
	H274	-6.812828910	10.309680789	2.320263511
	H275	-5.486786025	1.394280120	-0.859993609
	H276	-0.938531926	8.078585534	-3.425302638
	H277	-2.441465665	6.442658064	-5.577153563
	H278	-1.272113177	4.274437838	-4.541326325
	H279	-4.046227529	5.931362098	-3.076772459
	H280	1.116986002	8.899943659	3.040394554
	H281	-0.517490633	9.770907926	0.407506412
	H282	-0.101514110	12.633023486	0.673989959
	H283	2.456203588	9.422175147	1.995360376
	H284	-2.256358753	10.670623541	2.308914292
	H285	3.493018584	-2.779366304	-9.506775861
	H286	3.606128067	-4.267102090	-8.549539574
	H287	7.163806357	-5.054376789	-6.016584106
	H288	3.823084207	-4.830348551	-10.854339904
	H289	2.316579250	-5.314504531	-10.434298201
	H290	5.304114612	4.800961983	-1.273085668
	H291	5.777059676	7.485434208	0.050969459
	H292	4.349948254	8.118181218	-3.905229930
	H293	4.039227834	5.679434850	-0.353273932

	H294	6.691034339	8.066684754	-1.175650790
	H295	4.546006221	2.956107619	-7.048659349
	H296	5.595455236	6.723362967	-2.780048045
	H297	5.719998617	-7.036555178	4.180028383
	H298	7.233330709	-7.916954633	1.661711858
	H299	2.794421291	1.504223408	-6.975976496
	H300	5.335543490	-0.047635148	2.748058882
	H301	-0.413297947	1.695303882	-8.546008629
	H302	-1.674004723	-2.088657931	-4.157508268
	H303	-0.416850628	2.200885783	-6.857976216
	H304	4.728719620	-4.526789085	3.921792788
	H305	1.289412758	0.166024818	-6.597623747
	H306	4.207333429	-5.005149959	2.302769604
	H307	5.042540751	-7.277131461	2.552705589
	H308	6.622445557	-5.453539597	1.698196388
	H309	7.085351401	-5.116804789	3.375791340
	H310	-5.946765624	-0.663623859	-5.678507368
	H311	6.351855782	-9.496671628	4.049461679
	H312	5.372975540	-11.920008978	2.472484465
	H313	-0.293743447	-0.000171745	-4.070795495
	H314	6.993526963	-11.811331591	4.898899205
	H315	5.427335885	-12.247824030	4.709424876
	H316	5.618348368	-3.206067306	1.378639895
	H317	-1.303776427	2.171426931	-4.620840683
	H318	-4.027124500	-1.978345554	-4.953320437
	H319	7.292917755	-9.908316414	-2.687249713
	H320	6.073666596	-7.570396895	-4.640880629
	H321	6.482047812	-8.226412168	-0.964782965
	H322	6.308773584	-10.498805752	-3.743337344
	H323	3.820719146	2.732158410	-9.767236608
	H324	6.252467094	-5.160059441	-1.117557335
	H325	2.268402248	-7.930885806	0.409264847
	H326	2.345900582	-1.866321102	-7.772678422
	H327	2.826457096	-9.317205308	-1.404315221
	H328	2.164670342	-6.723971983	-7.413923442
	H329	1.565205704	-5.336787355	-5.384496071
	H330	7.047175164	-12.459305322	2.644131329
	H331	5.617011270	-0.951418308	1.281282215
	H332	8.355192690	-5.843118030	-0.268476292
	H333	7.045633470	-3.824149253	-1.073952796
	H334	9.185837274	-6.932914261	1.673814404
	H335	4.461685105	-2.801164420	4.770490369
	H336	3.186340025	-8.283683604	-4.268090632
	H337	4.547254440	-8.601153599	-3.184898932
	H338	1.549118809	-8.743732269	-2.489362552
	H339	0.549147967	-2.052254707	-6.147129488
	H340	-3.671230305	2.290732364	-5.450621764
	H341	-5.873496519	1.116223000	-5.805554091
	H342	2.293038409	-6.164623145	0.267462009
	H343	8.461583690	-7.375757282	-0.485117986
	H344	7.383001925	-9.289796650	-0.301034315
	H345	4.411675314	-1.113030200	4.628774419
	H346	4.644513265	-7.400592946	-7.428547859
	H347	6.067697692	-5.967209036	-3.929467615
	H348	-0.454730580	-2.803357884	-7.422737569
	H349	3.399735933	4.305285355	-7.195933804
	H350	8.100107648	0.033082529	3.151311863
	H351	7.962266063	0.484351045	1.436710366
	H352	8.112172672	-6.582697244	-7.531257263
	H353	7.476643307	-7.884723027	-6.768973946
	H354	9.974196332	-8.518472720	-3.009772678
	H355	8.048564912	-8.132671086	-4.169294732
	H356	11.111085850	-7.451884348	-5.250672373
	H357	9.881595167	-6.820335018	-2.492430515
	H358	10.662387638	0.213251298	1.134761929
	H359	-2.575879562	13.188139952	4.336477245
	H360	10.659222364	-7.020806762	4.297015006
	H361	10.015095116	-4.574367196	3.231968787
	H362	-4.568672654	12.342091378	2.246677572
	H363	-3.686063863	10.250165915	4.332049534
	H364	-5.310694475	10.946806663	4.194850336
	H365	0.889963538	-3.535468713	-0.880495215
	H366	3.952995317	-1.413720879	-0.170632689
	H367	-6.653662419	6.869938895	-0.643053728
	H368	0.015435043	-0.875761579	-0.281818563
	H369	2.937397294	-1.641061414	-0.449631921
	H370	1.880070204	-2.046644041	-0.091676658
	H371	-2.215724838	-0.207411329	-0.570775008
	Mo372	3.427977906	-4.456019956	-2.769368042
	N373	-7.323899788	-7.233188387	0.382141522

N374	-8.590450163	-4.656034617	2.776244733
N375	-7.469465168	-4.920019610	5.344773314
N376	-4.779677530	-5.714600225	4.970803283
N377	-3.627517483	-3.361902760	3.911501867
N378	-3.894961752	-1.646476958	6.196334340
N379	-2.555728281	-3.048025383	8.365939031
N380	-0.182039725	-4.349550341	7.350256687
N381	0.977550723	-2.719892118	5.301455585
N382	0.951916662	0.123917789	6.163891268
N383	0.975488062	-0.014817712	9.117092767
N384	6.130497860	-11.572860212	4.404716726
N385	6.673789004	-9.289934291	3.098394172
N386	5.331395726	-3.246340478	2.358375492
N387	4.850975522	-1.984931382	4.288823987
N388	5.268349845	-0.936866253	2.259121907
N389	9.352864138	-7.069102360	2.695212160
N390	-6.270923471	-2.900654624	-2.213593026
N391	-2.427495083	-5.554662096	-3.001875258
N392	-6.384306716	-0.423064809	-1.376778580
N393	-8.500713196	0.656043081	0.102617579
N394	-7.660663630	0.322900657	2.759820378
N395	-5.472416739	2.064528912	3.008401015
N396	-3.350246995	2.712588356	-0.624618711
N397	-2.637609325	0.697154585	-0.351099087
N398	-6.072797576	4.679947096	3.740328907
N399	-9.630625664	7.705996424	2.913809845
N400	-9.328641893	9.480018088	4.212051681
N401	9.664386083	2.582522901	1.517923094
N402	6.207075428	7.227702764	-0.843351478
N403	3.821700166	8.478416046	-3.114818181
N404	1.128160868	7.883099990	-3.288123216
N405	-3.708515129	6.473057733	-3.892140803
N406	-4.072232230	8.347420361	-5.251196936
N407	-4.899659048	8.297102259	-3.091654281
N408	0.465330197	6.348447919	-0.937784667
N409	1.021502814	8.223211958	1.062330172
N410	-0.351324661	10.617808915	0.950644422
N411	-2.255465196	10.336513928	-2.528251179
N412	-2.591104434	11.630540346	2.294034033
N413	2.897373567	-4.486029409	-10.591147279
N414	1.505642559	-2.415010523	-7.977861990
N415	0.536567410	0.363150422	-7.275768525
N416	2.605936573	2.363619026	-7.496726809
N417	-2.888774571	-2.769803486	-9.751368469
N418	-4.278651669	-1.005172417	-7.976669743
N419	7.305631571	-6.897469103	-6.984406390
N420	3.648295879	-5.561473185	-4.856603090
N421	2.842117037	-6.432162516	-6.714539515
N422	8.728397786	-7.368760950	-4.200246446
N423	-0.082463866	2.387334563	-0.222242639
N424	0.663157267	1.766845971	-0.799824750
O425	-6.451709901	-5.270702734	2.300739582
O426	-6.502015050	-2.868614195	5.120318248
O427	-4.343769278	-5.082833299	7.119192119
O428	-1.659143433	-3.993666416	4.869084504
O429	-2.050684764	-0.559230428	5.410182860
O430	-1.809311235	-0.972840784	8.969085855
O431	0.743784817	-4.080041397	9.434490940
O432	3.000163274	-3.711259916	5.750233600
O433	3.110027553	-0.004141958	5.426274967
O434	2.715034011	1.429411356	8.897016143
O435	3.408693398	-0.556203052	11.693731879
O436	7.249790067	-10.380361849	1.143489064
O437	8.482653211	-7.764009075	4.707191527
O438	12.045527282	-4.746353869	3.174753703
O439	-5.703410604	-0.896724815	0.764257951
O440	-4.248056761	-4.326574029	-3.592450095
O441	-7.226777248	2.499057436	0.542157118
O442	-6.735034063	3.092428192	-2.451904496
O443	-9.520726679	1.443653502	3.503969514
O444	-5.110080973	1.383286540	5.159469863
O445	-3.914171891	5.008833861	4.410441487
O446	-6.097045670	7.117164126	6.313052905
O447	11.142802249	-0.196851482	3.075348401
O448	3.912423871	4.557709356	3.653608014
O449	3.465099132	8.386337398	-0.866233032
O450	0.550211293	10.106044589	-3.339779263
O451	-1.634050521	7.222966652	-1.178303694
O452	-0.538011158	7.208276921	2.405757642
O453	2.470295210	5.679140796	1.387648286

	O454	1.089678489	11.596982792	2.441492046
	O455	-2.498776149	13.589401626	1.131309991
	O456	-3.466953253	11.038974455	-0.723134817
	O457	-4.560974763	13.623817460	4.553191176
	O458	-5.686929298	6.881078614	-0.501746961
	O459	0.712962420	-4.368890963	-8.887889964
	O460	-1.263136074	-0.573557205	-8.341589863
	O461	1.106397816	3.777342146	-8.459089569
	O462	4.824130696	4.474097265	-9.412913147
	O463	-6.013342499	-2.477030979	-8.131989377
	O464	-2.195985816	-6.518617754	-5.871742933
	O465	-0.382467354	-5.653814990	-6.841680688
	O466	-4.928773140	2.489321269	-7.750064111
	O467	9.381829452	-5.318064613	-4.909599753
	O468	12.230033679	-6.842413372	-3.657263828
	O469	0.023525929	-6.372313787	-1.453531988
	O470	-0.279438482	-7.477544425	0.478937240
	O471	4.638124355	-10.904798670	-4.111263359
	O472	2.384041918	-10.948418131	-3.920597956
	O473	4.827637751	-5.988539707	-2.025124673
	O474	4.890927347	-7.690590982	-0.559429271
	O475	2.259822003	-6.175276061	-2.472192256
	O476	8.953539166	-6.607604603	-0.082649495
	O477	6.938981327	-4.670448179	-0.599614210
	O478	-4.703907662	4.805946668	-1.784087078
	O479	7.285964159	-10.374080370	-3.552963421
	O480	7.374489814	-8.656469753	-1.062558729
	S481	3.208102961	4.960969423	-2.521836597
	S482	1.665650305	1.997663266	-3.561436264
	S483	1.491629877	-3.370903802	-3.600507444
	S484	3.526235565	1.690382263	-0.418844731
	S485	0.285947030	-1.000992604	-1.605060349
	S486	3.299394137	-0.788420488	-5.745710487
	S487	3.419656554	-3.901693275	-0.536169474
	S488	5.220206508	1.728516820	-3.557684599
	S489	5.194591356	-3.037826846	-3.453650462
	S490	6.205399516	-0.955827007	-0.947666423
	end			

S2BH-6Hx-7Hn-6H2n transverse (8x) to S2BH-6H2x-7H2n (10x) without 2N2x

Reactant (8x)

	bm522bh6hx7hn6h2nts.car_4			
Fe(139) -2.595	C1	-7.786102525	-5.373583514	-0.372391729
Fe(140) -0.595	C2	-7.938976325	-6.472145360	-1.428929773
Fe(141) -2.294	C3	-8.890994418	-3.524973038	0.806774261
Fe(142) 2.745	C4	-8.509797578	-3.860842491	2.245824810
Fe(143) 2.718	C5	-8.435435597	-5.550827653	4.014432485
Fe(144) 0.830	C6	-6.926871029	-5.623242008	4.271354263
Fe(145) 0.060	C7	-4.679817432	-5.835785982	3.335659275
[	C8	-4.155467066	-4.405784329	3.502750058
**S=1/2**	C9	-4.479040819	-2.056106984	2.906408487
	C10	-4.748558109	-1.557649345	4.332820540
	C11	-6.234679137	-1.517243904	6.246394795
	C12	-5.394280842	-2.163454933	7.357301452
	C13	-4.622877676	-4.259629447	8.370476329
	C14	-3.108001639	-4.499822892	8.285861334
	C15	-1.199774898	-4.995181855	6.803473879
	C16	-0.370888667	-3.831363770	6.258490063
	C17	-0.385588920	-1.567918700	5.311512636
	C18	-0.446004264	-0.417513142	6.333969857
	C19	-0.795084915	-1.164914398	3.873716290
	C20	-0.064728546	0.109347698	3.432465474
	C21	-0.514135322	-2.317409563	2.903545351
	C22	-1.807924003	1.259222099	7.498769810
	C23	-1.727525579	0.831006443	8.976252442
	C24	-3.085371127	2.068746611	7.166145042
	C25	-3.326189324	3.174455780	8.199951962
	C26	-2.985157226	2.663968719	5.755457284
	C27	-2.489585662	-0.652070371	10.738439736
	C28	-1.099369698	-0.884713992	11.295579481
	C29	5.071907994	-11.829153950	4.157563964
	C30	5.823422034	-10.541593909	3.833755454
	C31	6.024134229	-8.154908421	4.512628645
	C32	7.088484550	-7.838584383	5.581491231
	C33	4.835635529	-7.181209519	4.607263534
	C34	5.240264455	-5.732146078	4.343141616

C35	4.032190868	-4.809917127	4.187968806
C36	4.119072727	-2.299790313	4.187951434
C37	9.292706087	-6.833566942	5.974666441
C38	9.438625399	-5.331001649	6.085271573
C39	-4.925221465	-2.488348808	-2.706991432
C40	-5.354976596	-1.074477852	-2.274223740
C41	-3.432878405	-2.664528620	-2.344200140
C42	-2.887842152	-4.105826684	-2.402851082
C43	-2.362941048	-4.563607914	-3.763783486
C44	-5.629403847	1.220105527	-3.132437395
C45	-6.878202975	1.648376292	-2.348627178
C46	-5.535607122	1.894978297	-4.507251560
C47	-9.198597229	1.120889454	-1.758236309
C48	-9.083955495	1.012613138	-0.233704082
C49	-7.711055607	0.344672243	1.683739433
C50	-6.577641079	1.247875448	2.172253887
C51	-4.946698601	3.002641384	1.681817669
C52	-5.516535451	4.265641900	2.365837011
C53	-4.117827096	3.533113916	0.502997099
C54	-3.302832340	2.543040754	-0.256120075
C55	-2.849348927	1.274673671	0.006878060
C56	-2.078420541	1.866126367	-1.996687338
C57	-7.277645190	5.974301215	2.226620605
C58	-7.404976105	6.083803577	3.744779076
C59	-8.666552823	6.075699765	1.550315382
C60	-9.244340596	7.452590277	1.395917298
C61	-9.332466196	8.501748332	2.290032067
C62	-10.271029709	9.052561307	0.359749633
C63	8.507117649	1.479087771	5.094652578
C64	9.572691465	0.400944554	5.016306626
C65	7.194086474	0.844889281	4.568661900
C66	6.013913290	1.786325350	4.613642189
C67	5.212925483	1.900570930	5.764420072
C68	5.668722216	2.560542659	3.493405362
C69	4.095278250	2.741988426	5.792329994
C70	4.564005213	3.413200878	3.509662127
C71	3.771529260	3.495531367	4.657647440
C72	5.503059904	6.910579653	-0.103427377
C73	4.490845341	8.019431640	-0.423492572
C74	4.785668365	5.569311313	0.078147519
C75	3.772234373	9.687542744	-2.100346418
C76	2.371136561	9.305297849	-2.582019138
C77	0.797055305	7.403169874	-2.935736008
C78	0.030786836	6.966899837	-1.663059062
C79	1.045059066	6.225409211	-3.906468650
C80	-0.137568353	5.343680060	-4.323499704
C81	-1.119456915	5.939226473	-5.342073885
C82	-2.697359515	7.844926621	-5.050418541
C83	0.052858869	5.884239152	0.524122043
C84	-0.216309055	7.051555035	1.488597088
C85	0.816509303	4.790232436	1.292356064
C86	0.762461792	9.033759212	2.544054722
C87	0.212786078	10.418879745	2.229746650
C88	-0.843909443	11.869540960	0.568533300
C89	-2.207756887	12.408639847	1.059734894
C90	-0.721847442	11.987953666	-0.954658765
C91	-1.766973145	11.195023354	-1.732534372
C92	-4.394080733	11.867993825	2.022999381
C93	-4.491994017	12.903494940	3.133901379
C94	-5.157606303	10.577216875	2.433566519
C95	-5.201203131	9.565205510	1.310815284
C96	-6.208067280	9.637049289	0.335388048
C97	-4.208933388	8.582630941	1.164357139
C98	-6.210904815	8.782414290	-0.768552487
C99	-4.181814736	7.734226107	0.052930787
C100	-5.182763673	7.849588957	-0.906594878
C101	5.685098801	-3.618784905	-8.265458383
C102	4.203147544	-3.377481568	-8.039940167
C103	2.551145104	-1.896761632	-6.921516070
C104	2.155858447	-0.518410942	-7.462134491
C105	2.549602895	1.898098578	-7.246128839
C106	3.760663255	2.825806265	-7.218748038
C107	5.969335748	3.381586280	-6.380553422
C108	6.606703408	3.764694830	-7.700503908
C109	-0.966487100	-3.064112632	-9.673809908
C110	-2.026840316	-1.969401234	-9.512353191
C111	-0.762855673	-3.799237377	-8.328527876
C112	-0.025087682	-5.136897941	-8.529661726
C113	0.411428384	-5.710271418	-7.199318091
C114	-2.534084238	0.325337805	-8.845193877

C115	-1.939486589	1.625202537	-9.349701344
C116	-3.185832535	0.409087413	-7.442701088
C117	-2.156343142	0.343626645	-6.347977658
C118	-1.455577360	1.494006841	-5.949423645
C119	-1.778843135	-0.899527722	-5.810356249
C120	-0.364852585	1.390586790	-5.080984597
C121	-0.686642329	-1.002391412	-4.944489953
C122	0.040423206	0.140568130	-4.600842127
C123	8.625851384	-6.144128590	-3.550736067
C124	9.615923309	-6.259081463	-2.378849788
C125	7.194043804	-6.354596217	-3.033302245
C126	6.084539797	-6.106353730	-4.006558766
C127	5.927678516	-6.653732341	-5.263182021
C128	4.082274924	-5.563438414	-4.709942034
C129	10.518673497	-7.544550709	-0.534858996
C130	11.953024888	-7.198561673	-0.870896103
C131	0.596295504	-6.913972425	-0.329776411
C132	2.014911796	-6.982278953	0.227078626
C133	3.131535630	-7.038109063	-0.857499303
C134	3.160982047	-8.447926514	-1.512061515
C135	4.387168491	-8.749415165	-2.378891938
C136	4.486760903	-10.222253469	-2.836818273
C137	4.498353456	-6.805860560	-0.193858306
C138	3.966782321	-0.604877136	-1.571031697
Fe139	4.430352110	-1.870934661	-2.994829021
Fe140	2.562288831	0.568094431	-1.256656811
Fe141	4.985500377	0.571000575	-0.356514604
Fe142	4.513416378	0.731392693	-2.952976523
Fe143	3.936219621	2.766894398	-1.244355741
Fe144	4.771783131	-1.964268790	-0.436637684
Fe145	2.178107435	-2.379440586	-0.901531317
H146	-6.148985602	-7.402748413	-1.131181503
H147	-6.137797254	-6.360727539	-2.387751417
H148	-9.742157962	-4.804589063	-0.674667961
H149	-8.520246128	-6.074863733	-2.276517585
H150	-8.567447777	-7.258641819	-0.976356562
H151	-8.171708444	-2.768061481	0.461253713
H152	-9.251941350	-5.756049386	2.035919264
H153	-9.887803453	-3.063989904	0.812332689
H154	-8.868509520	-6.546530360	4.179248700
H155	-6.534288807	-5.935523388	2.269043743
H156	-8.850631349	-4.871900670	4.771171834
H157	-3.425560747	-1.848372839	2.683555062
H158	-5.510902306	-3.751767065	2.100880722
H159	-4.230671453	-6.285329722	2.439357003
H160	-6.086413974	-0.432371027	6.323328601
H161	-6.603907158	-2.459897483	4.342735447
H162	-4.354284564	-6.413819461	4.208822263
H163	-5.099751520	-1.497468657	2.193565796
H164	-5.702904948	-4.033799944	6.537891042
H165	-7.293633193	-1.744375608	6.431330897
H166	-1.023927322	-3.242378794	3.206386566
H167	-0.855593428	-2.063874726	1.887400431
H168	0.567272324	-2.524251020	2.853525390
H169	-1.876779971	-0.956735298	3.883518009
H170	-5.112102094	-5.243612524	8.423838657
H171	-2.864730638	1.888596197	4.986671495
H172	-3.147130212	-0.672748661	8.712837960
H173	-3.490583329	2.773241597	9.210429946
H174	1.025940213	-0.045329038	3.407764622
H175	-0.272893836	0.962613094	4.094563814
H176	8.360888717	1.732695477	6.162125575
H177	-3.891743386	3.235253481	5.510631264
H178	-4.212343926	3.761874167	7.918283707
H179	-2.125346750	3.351439785	5.691786512
H180	-2.464581184	3.859611241	8.243329329
H181	-3.944226115	1.374012179	7.195246517
H182	-0.933140420	1.911144075	7.355216910
H183	-2.488581206	-0.210352331	6.077511226
H184	-0.377816577	0.395823034	2.416006267
H185	1.126443670	3.999761358	0.585546761
H186	0.113575067	4.355640695	2.019200812
H187	2.038322404	4.929936629	2.838807456
H188	2.185412278	4.286227769	5.452665182
H189	4.293091203	3.989564227	2.625587694
H190	6.253337452	2.471053605	2.575454128
H191	5.452777701	1.307953066	6.651156483
H192	-2.078357074	-2.827967284	5.722125054
H193	-1.097174252	-5.831220860	6.094884003
H194	-0.739457529	-5.301355640	7.751788254

	H195	-3.214260095	-4.681059549	6.219604709
	H196	-4.799780911	-3.733209564	9.315984209
	H197	3.471258171	2.799194851	6.688510072
	H198	0.676029852	-1.835584210	5.286654370
	H199	-3.014579488	0.073817514	11.381218317
	H200	-3.024753158	-1.613323756	10.797335524
	H201	-0.334546403	-1.194076032	10.544772152
	H202	10.248034336	-7.273165670	5.649567812
	H203	-0.978428641	-5.801775144	-4.623489200
	H204	1.487594377	-5.987151509	-1.769132349
	H205	-0.701124226	-5.859208230	-9.008800616
	H206	0.846829644	-4.991393557	-9.184008617
	H207	-1.736161076	-3.994134528	-7.852060642
	H208	-1.193269047	1.498644897	-10.168643701
	H209	-1.434543622	-3.776159674	-10.370114801
	H210	-2.833688500	-2.012113822	-3.000525276
	H211	-3.674226375	-4.818934698	-2.099981599
	H212	-0.614455143	-0.636870990	-8.767724330
	H213	-3.355331495	0.119058885	-9.562317652
	H214	0.821522644	-2.006130186	-9.682952904
	H215	-0.881547574	-5.728018766	-2.866800536
	H216	-0.203971985	-3.143103138	-7.643426237
	H217	0.811186641	-3.327655204	-10.666119253
	H218	-6.057230756	-3.164553492	-4.295492443
	H219	-3.307117473	-2.301518495	-1.313520800
	H220	2.193239969	-4.938616070	-7.513725614
	H221	-2.085312944	-4.216150700	-1.658485850
	H222	-4.307700112	2.512623413	2.429438552
	H223	-2.999339483	0.627894820	0.862029659
	H224	-8.622655901	0.667120519	2.206549031
	H225	-7.278074405	-0.010416691	-0.373332091
	H226	-7.217606300	4.078715811	1.230659439
	H227	-8.979703953	8.626994945	3.307566134
	H228	-7.627685762	5.112043294	4.248082257
	H229	-9.371895556	5.411073142	2.079700240
	H230	-6.620619308	6.783716927	1.870384638
	H231	-10.786636879	9.657833620	-0.382196762
	H232	-3.446620480	4.305288137	0.913761835
	H233	-4.792104871	4.040121241	-0.205437807
	H234	-10.204653182	10.430459322	1.981682506
	H235	-8.564598805	5.669797350	0.531694862
	H236	-6.297183806	2.077192229	0.280767505
	H237	-7.498032668	-0.679628669	2.023231221
	H238	-9.573878785	2.132880749	-1.963824338
	H239	-1.559018969	1.829055109	-2.951515451
	H240	-9.960482592	0.407543274	-2.098032503
	H241	9.645751251	3.143026119	4.725940867
	H242	8.122422963	3.249145740	4.140951904
	H243	1.693514060	5.953092229	-0.881443023
	H244	-0.934878022	5.481788987	0.255628299
	H245	1.562896213	7.918392030	0.935050250
	H246	-3.072683266	3.752781094	-2.040399366
	H247	-4.444904781	4.393058764	-3.493315944
	H248	-5.701067854	3.712869041	-5.221058098
	H249	-4.324861607	5.609861259	-2.471421558
	H250	-4.603651440	1.560127067	-4.992322667
	H251	-6.391209169	1.592423879	-5.130986535
	H252	0.300125898	11.723050587	-1.261158381
	H253	-0.876070575	13.047787546	-1.211687120
	H254	-0.296791377	10.425225334	-2.983064034
	H255	-1.962872365	9.957238801	-3.316064833
	H256	-2.553663372	9.440718426	-6.328308591
	H257	-1.529988757	5.138545061	-5.976627724
	H258	-7.009380982	8.842611736	-1.512075439
	H259	-3.433370699	8.454228931	1.923958552
	H260	-3.940755016	8.011364666	-3.416102950
	H261	1.795375515	5.568696512	-3.437891815
	H262	2.847792160	7.334990570	-2.359408644
	H263	-0.688935080	4.978492750	-3.442484807
	H264	-7.898256426	0.037426269	-3.079357363
	H265	4.275953003	10.220385693	-2.917032911
	H266	-5.357918655	-0.677658489	-4.227026544
	H267	1.521615494	6.642122528	-4.808664458
	H268	-3.363482977	7.024480039	-0.076444946
	H269	3.628417510	10.399315830	-1.274114149
	H270	-1.775224809	8.000111123	-6.886413026
	H271	-4.205785263	9.172250248	-4.669191956
	H272	-4.407888167	-3.249863849	-4.547190103
	H273	-5.507255973	-3.182417055	-2.072217713
	H274	-7.008898580	10.373152066	0.436738391

H275	-4.792071111	1.570681624	-2.511335240
H276	0.182504459	8.180597401	-3.402353327
H277	-0.581680189	6.632749906	-6.000978498
H278	0.301803799	4.453605316	-4.795964322
H279	-2.772608349	6.102529378	-4.002453272
H280	0.200879074	8.650467946	3.408789192
H281	-0.484444765	9.746406924	0.428696667
H282	-0.115880943	12.568362876	1.008305038
H283	1.809262784	9.169578254	2.849749237
H284	-2.731423498	10.561073923	1.709276173
H285	6.215476737	-2.656164173	-8.318564884
H286	6.043712808	-4.130448947	-7.348119126
H287	8.730003581	-5.109609375	-3.908916928
H288	6.865564197	-4.714267302	-9.513711324
H289	5.302581743	-5.201259095	-9.487402473
H290	5.547142826	4.809794994	0.295949461
H291	5.602460044	7.408080339	1.875742628
H292	5.351499130	8.263749662	-2.275070678
H293	4.078421288	5.633744671	0.915807076
H294	6.816046969	8.055542562	0.986808789
H295	6.742643550	2.894836192	-5.766066291
H296	6.220866646	6.817606899	-0.933941348
H297	4.366740001	-7.283990156	5.600471933
H298	6.469857267	-8.080120668	3.512346061
H299	4.909829561	1.563969012	-6.015798911
H300	4.314146136	-0.281880858	3.927819862
H301	2.155084105	1.892776388	-8.269498018
H302	-0.387892825	-1.976337912	-4.550560978
H303	1.782887695	2.375201567	-6.612592805
H304	3.512396172	-4.697736651	5.147251300
H305	3.300286302	0.303066929	-5.986211427
H306	3.313621456	-5.234746200	3.468152826
H307	4.097832620	-7.503727507	3.854955068
H308	5.834062962	-5.684056711	3.418975486
H309	5.875852529	-5.349499085	5.157041898
H310	-3.895741966	-0.426091736	-7.354186211
H311	4.911641443	-9.760762312	5.434032392
H312	4.291669616	-11.922236929	3.387125540
H313	0.917799845	0.076847722	-3.957476816
H314	5.083092812	-12.191061964	6.184410970
H315	3.620124838	-12.427819145	5.487595803
H316	5.118554991	-3.517169517	2.915036025
H317	0.198999559	2.277582236	-4.784292063
H318	-2.334291770	-1.799055847	-6.083009596
H319	7.825087266	-9.899854816	-0.679614486
H320	7.097089730	-7.393273565	-2.674151477
H321	6.523691936	-8.215807425	0.740097415
H322	7.173697165	-10.451256856	-1.977048842
H323	6.449750531	3.037168935	-8.531880904
H324	6.286359101	-5.094719419	0.705396128
H325	2.088219746	-7.836335655	0.911323519
H326	4.660038075	-1.717180062	-6.932151680
H327	3.127597554	-9.174631242	-0.684973085
H328	4.214724190	-6.587320656	-6.548040481
H329	3.070858144	-5.185060104	-4.777116561
H330	5.777787342	-12.661125172	3.993987063
H331	5.270213510	-1.211697393	2.823946900
H332	8.071380963	-5.899654236	2.055433750
H333	7.119011079	-3.828617818	1.026872792
H334	8.308607133	-7.052879209	4.080867244
H335	2.632600227	-2.946976959	5.515915258
H336	4.363105634	-8.121823157	-3.282802537
H337	5.308362383	-8.497805052	-1.836127866
H338	2.239807233	-8.584557216	-2.098782737
H339	2.493597374	-1.915063948	-5.821666640
H340	-1.748245238	2.463833718	-6.359078569
H341	-3.755444406	1.350272626	-7.409576893
H342	2.147275671	-6.064900086	0.826073150
H343	8.246880215	-7.424515740	1.805435252
H344	7.152369495	-9.337727921	1.599878443
H345	3.041721608	-1.267482539	5.597423198
H346	6.593854584	-7.272502483	-5.850890165
H347	7.041251760	-5.720060879	-2.153284438
H348	1.856932693	-2.632898221	-7.330872273
H349	5.652610645	4.298231400	-5.858560588
H350	6.984099269	-0.046049224	5.178679381
H351	7.356720546	0.508383627	3.532043504
H352	9.908278438	-6.850823921	-4.985224625
H353	8.933312110	-8.013808886	-4.373032246
H354	10.507024374	-8.596542576	-0.207777225

	H355	9.013630378	-8.214896960	-1.889149441
	H356	12.238255544	-7.370321803	-1.936726958
	H357	10.177803628	-6.927447227	0.311848612
	H358	10.034093720	0.287309580	4.005084982
	H359	-3.597953547	12.971786908	3.797731348
	H360	9.069972113	-7.213875478	6.987150860
	H361	8.495052567	-4.754416466	5.918121270
	H362	-4.870746617	12.298390071	1.126517819
	H363	-4.680207807	10.144143278	3.326351708
	H364	-6.176836697	10.875959960	2.717554127
	H365	1.083246232	-3.411655635	-0.828100570
	H366	4.130741885	-1.371931739	0.776294524
	H367	-5.984600113	7.040649444	-2.488064681
	H368	0.281054218	-0.468852889	-0.013401153
	H369	1.957727743	-1.754364600	0.556545828
	H370	1.377579689	-2.424493874	0.433826086
	H371	-1.535730961	0.029945183	-1.192332336
	Mo372	4.117532480	-4.258099623	-1.768476335
	N373	-6.709168603	-7.068329529	-1.919539927
	N374	-8.920359711	-4.668578870	-0.091078185
	N375	-8.800157551	-5.109573625	2.678706578
	N376	-6.130032254	-5.892150796	3.206547003
	N377	-4.765861101	-3.471377820	2.734948174
	N378	-5.901702581	-1.972736916	4.905688702
	N379	-5.232971599	-3.509653824	7.281993845
	N380	-2.604408461	-4.718899307	7.039705261
	N381	-1.064770624	-2.768224078	5.782202899
	N382	-1.666953503	0.119327507	6.591815335
	N383	-2.466092384	-0.246552382	9.345410396
	N384	4.439155240	-11.817979777	5.483431410
	N385	5.545911906	-9.518462912	4.665655695
	N386	4.468484728	-3.497572249	3.705228234
	N387	3.228334443	-2.180931920	5.193968601
	N388	4.695175961	-1.191904437	3.686347182
	N389	8.202445507	-7.228332376	5.104602455
	N390	-5.171845121	-2.677946948	-4.141667333
	N391	-1.348549665	-5.447567355	-3.734927346
	N392	-5.524628191	-0.217738811	-3.315636163
	N393	-7.974732664	0.872167693	-2.502115953
	N394	-7.986671770	0.375184716	0.260395469
	N395	-5.975857286	2.064248818	1.253432015
	N396	-2.806535156	2.879067425	-1.508676012
	N397	-2.106346336	0.883441013	-1.090381231
	N398	-6.719525998	4.672202877	1.889486351
	N399	-9.833940192	7.809479308	0.194008945
	N400	-9.984513755	9.509683010	1.611191781
	N401	8.912788176	2.608076980	4.253831823
	N402	6.264610112	7.203395947	1.120467227
	N403	4.613180492	8.591778747	-1.658063142
	N404	2.096958559	7.984883375	-2.618093542
	N405	-2.240918958	6.640975571	-4.709257542
	N406	-2.232078885	8.498591704	-6.131141670
	N407	-3.586733107	8.481141096	-4.252829357
	N408	0.729590025	6.294196167	-0.706198567
	N409	0.699481012	8.061464957	1.466161424
	N410	-0.484943532	10.546676862	1.060556469
	N411	-1.304335045	10.584762073	-2.856681721
	N412	-3.003769272	11.540889240	1.733117726
	N413	5.902463423	-4.371345138	-9.503082526
	N414	3.897006473	-2.261775777	-7.343518013
	N415	2.761549800	0.519373073	-6.841122264
	N416	4.871923604	2.441325208	-6.542493536
	N417	0.240215399	-2.548353754	-10.332473686
	N418	-1.597015478	-0.767162083	-9.028398451
	N419	8.948984496	-7.035945093	-4.678150412
	N420	4.917218991	-5.414752301	-3.680637521
	N421	4.665928604	-6.296894199	-5.684653378
	N422	9.647185373	-7.433843932	-1.692558366
	O425	-6.721037658	-5.159587813	0.234384241
	O426	-7.961323033	-3.006125329	2.964888320
	O427	-6.484263751	-5.460021289	5.420891477
	O428	-3.239508449	-4.117951114	4.295476852
	O429	-3.938929436	-0.823539470	4.921040873
	O430	-4.939163259	-1.475515386	8.287494970
	O431	-2.431078475	-4.540389955	9.321503229
	O432	0.872603986	-3.913520982	6.258372532
	O433	0.597426569	-0.008823777	6.869883110
	O434	-1.017592073	1.444540159	9.791421492
	O435	-0.838343125	-0.816911180	12.487456774
	O436	6.591795644	-10.497432029	2.851188222

	O437	6.896985432	-8.110533440	6.779280399
	O438	10.487982804	-4.766772592	6.351508107
	O439	-5.508703569	-0.743380169	-1.082455284
	O440	-2.864129778	-4.161205913	-4.839110968
	O441	-6.850263155	2.644203726	-1.603473149
	O442	-5.534432570	3.327081871	-4.342267502
	O443	-9.978707023	1.495287928	0.473896334
	O444	-6.245108364	1.223389588	3.361142551
	O445	-4.864864653	4.903045620	3.198316964
	O446	-7.333236461	7.128058400	4.369224587
	O447	9.900806419	-0.316100603	5.950461215
	O448	2.670580587	4.331130052	4.608210255
	O449	3.621988297	8.382755879	0.388261052
	O450	1.575279810	10.209089511	-2.905437033
	O451	-1.177169335	7.234403143	-1.545588860
	O452	-1.185849519	7.022075558	2.257903875
	O453	1.954654294	5.342040571	1.955262727
	O454	0.385971789	11.355673603	3.018913143
	O455	-2.510754326	13.596424865	0.878696596
	O456	-2.957328134	11.162060576	-1.389695695
	O457	-5.486439002	13.587246193	3.319560582
	O458	-5.112904146	7.024071644	-2.047429318
	O459	3.347678611	-4.198681598	-8.449504695
	O460	1.361930223	-0.384810369	-8.415768877
	O461	3.686565162	3.931230871	-7.781256254
	O462	7.285116505	4.768759171	-7.849477282
	O463	-3.219360094	-2.176222400	-9.790516088
	O464	-0.335160683	-6.342306604	-6.459255029
	O465	1.676496633	-5.446864689	-6.814605063
	O466	-2.270275846	2.736655176	-8.960459332
	O467	10.384573747	-5.325763831	-2.076165759
	O468	12.774382439	-6.831297187	-0.042282536
	O469	0.471709914	-6.238652546	-1.478013225
	O470	-0.372740409	-7.394496481	0.256892072
	O471	5.641767971	-10.773362977	-2.779643514
	O472	3.433347850	-10.772881652	-3.264577407
	O473	5.211184756	-5.811200968	-0.654228192
	O474	4.894489305	-7.557974647	0.716765195
	O475	2.875630359	-5.998013259	-1.805690809
	O476	8.603323162	-6.674927539	2.358990992
	O477	6.778237354	-4.645002434	1.439949751
	O478	-3.793026155	4.953510237	-2.989553558
	O479	8.059958462	-10.360862903	-1.515795963
	O480	7.389132790	-8.678245631	0.898854780
	S481	3.830099973	5.049872043	-1.422891206
	S482	2.604818932	1.987756973	-2.916754459
	S483	2.505759761	-3.083218158	-3.074896817
	S484	3.211541664	1.648599917	0.582903420
	S485	0.676506110	-0.654880655	-1.306891389
	S486	5.094714862	-0.543110966	-4.669352947
	S487	3.540726615	-3.594854987	0.368601622
	S488	6.013560347	1.956627927	-1.744379077
	S489	6.111242308	-2.985491321	-2.014454945
	S490	6.333496744	-0.796360109	0.728848289
	end			

TS

	bm522bh6hx7hn6h2nts_1_52911.98			
Fe(139) -2.558	C1	-7.792893277	-5.360710115	-0.383967359
Fe(140) -1.008	C2	-7.965621119	-6.459724745	-1.437541732
Fe(141) -2.159	C3	-8.885061402	-3.508740789	0.805527402
Fe(142) 2.752	C4	-8.504845550	-3.846000111	2.244393829
Fe(143) 2.774	C5	-8.429182428	-5.534807578	4.013293858
Fe(144) 0.893	C6	-6.919887934	-5.610181359	4.265464407
Fe(145) 0.217	C7	-4.677769332	-5.830276353	3.320963502
	C8	-4.157012285	-4.399153486	3.489901670
	C9	-4.482643800	-2.050333825	2.883116407
	C10	-4.749769427	-1.542454819	4.306874339
S=1/2	C11	-6.226827888	-1.493439645	6.225891480
	C12	-5.380711048	-2.132418327	7.336615642
	C13	-4.602070352	-4.224377695	8.354416193
	C14	-3.088641271	-4.476492841	8.271815281
	C15	-1.185481157	-5.019365459	6.800668322
	C16	-0.357793051	-3.852414958	6.258829135
	C17	-0.388561595	-1.591606029	5.300476951
	C18	-0.444440903	-0.439357984	6.321265837
	C19	-0.818667969	-1.203561300	3.864586438
	C20	-0.140993750	0.095106657	3.411624911
	C21	-0.510662660	-2.348525143	2.893505811

	C22	-1.795627903	1.245560157	7.485317044
	C23	-1.709877810	0.825629349	8.965079623
	C24	-3.072094556	2.057349831	7.154939352
	C25	-3.308511195	3.164529899	8.188064458
	C26	-2.974663866	2.650821020	5.743289065
	C27	-2.472511219	-0.639204771	10.742012720
	C28	-1.081330152	-0.874781789	11.295339283
	C29	5.066676339	-11.815901186	4.166350227
	C30	5.816395542	-10.528954607	3.841124827
	C31	6.012861019	-8.142856069	4.519003313
	C32	7.072739888	-7.817054493	5.588802752
	C33	4.819163148	-7.176039228	4.605091551
	C34	5.222612805	-5.726392793	4.345084452
	C35	4.015158046	-4.808728619	4.165223266
	C36	4.122249040	-2.300517044	4.186909305
	C37	9.280674100	-6.821623410	5.979192734
	C38	9.452334715	-5.321583769	6.077448924
	C39	-4.927115103	-2.501967470	-2.693148090
	C40	-5.368735923	-1.091246622	-2.260581510
	C41	-3.433205253	-2.670990751	-2.338347779
	C42	-2.886745987	-4.111392786	-2.402546019
	C43	-2.359136052	-4.559560754	-3.765672189
	C44	-5.659231543	1.199845937	-3.122431134
	C45	-6.906989168	1.627672462	-2.337549777
	C46	-5.567053349	1.874843618	-4.497371627
	C47	-9.224640151	1.101195488	-1.739064050
	C48	-9.107657634	1.003026502	-0.214166218
	C49	-7.732917128	0.353271945	1.707644955
	C50	-6.602302263	1.264779661	2.185787365
	C51	-4.956778622	2.997660730	1.667059073
	C52	-5.514387858	4.273552801	2.339292219
	C53	-4.126981067	3.500350623	0.476954006
	C54	-3.331298311	2.489116666	-0.276985184
	C55	-2.898775219	1.214617992	-0.007902938
	C56	-2.101870823	1.789211215	-2.006811951
	C57	-7.286111253	5.977100318	2.208490948
	C58	-7.421225506	6.081958310	3.726511227
	C59	-8.672233161	6.073007083	1.525774265
	C60	-9.254913211	7.447350394	1.368848275
	C61	-9.342391117	8.499309331	2.259541847
	C62	-10.287206671	9.041745447	0.329904503
	C63	8.497121851	1.458073886	5.100291550
	C64	9.549750144	0.367326648	5.025201271
	C65	7.174124586	0.831410020	4.589825003
	C66	6.002749508	1.783415441	4.639669979
	C67	5.212011669	1.909382380	5.796585177
	C68	5.657238905	2.559176800	3.520529030
	C69	4.106747658	2.766920749	5.833405851
	C70	4.565508989	3.428587851	3.545628453
	C71	3.785372620	3.525475764	4.701467852
	C72	5.530405934	6.906888277	-0.096846844
	C73	4.522001624	8.019538279	-0.418243005
	C74	4.805483309	5.569338680	0.081581506
	C75	3.803109423	9.681731748	-2.101756380
	C76	2.394711141	9.310225032	-2.571280623
	C77	0.803042217	7.418366887	-2.905827073
	C78	0.047573735	6.980300834	-1.626692286
	C79	1.039032271	6.238256885	-3.877938599
	C80	-0.148638379	5.359118067	-4.286636202
	C81	-1.127507426	5.947944092	-5.313012620
	C82	-2.714685774	7.848149349	-5.035605977
	C83	0.094361368	5.901173687	0.563300095
	C84	-0.182029128	7.073590313	1.519236244
	C85	0.877180600	4.821687366	1.334386869
	C86	0.783581340	9.073610262	2.553403166
	C87	0.226719982	10.452540236	2.224578675
	C88	-0.843833110	11.878013760	0.550551234
	C89	-2.213186639	12.415568174	1.029737565
	C90	-0.714211294	11.981127995	-0.972706774
	C91	-1.752422090	11.175176657	-1.745854531
	C92	-4.395177293	11.877170935	2.007564315
	C93	-4.494657236	12.917025844	3.114627601
	C94	-5.152920114	10.585019131	2.425146149
	C95	-5.198713386	9.568661162	1.306191195
	C96	-6.212545311	9.632738292	0.337219512
	C97	-4.204991121	8.587740609	1.158151502
	C98	-6.220938817	8.771437996	-0.761812859
	C99	-4.183202854	7.733226151	0.051320456
	C100	-5.191343158	7.840272148	-0.901809311
	C101	5.682769666	-3.609766999	-8.273984145

	C102	4.199233735	-3.371074812	-8.055875289
	C103	2.539796431	-1.889033811	-6.949504176
	C104	2.146545619	-0.508356324	-7.485998372
	C105	2.553762658	1.906384638	-7.279879142
	C106	3.763149290	2.836276210	-7.242657464
	C107	5.943488937	3.433624417	-6.357962110
	C108	6.624119335	3.767663898	-7.669582632
	C109	-0.988218364	-3.061272567	-9.668894330
	C110	-2.049751846	-1.969043101	-9.502785903
	C111	-0.772388487	-3.794024723	-8.324869888
	C112	-0.024220888	-5.124738765	-8.530642943
	C113	0.415126997	-5.700906699	-7.202477012
	C114	-2.554839408	0.326111982	-8.837219565
	C115	-1.958647117	1.624355799	-9.344077187
	C116	-3.205037964	0.413566948	-7.434598418
	C117	-2.170349568	0.366707693	-6.343925983
	C118	-1.485386366	1.528308120	-5.949387086
	C119	-1.769687740	-0.869981706	-5.808850914
	C120	-0.389134733	1.442464356	-5.085852823
	C121	-0.673717108	-0.954597536	-4.945979771
	C122	0.035924491	0.199863493	-4.602640769
	C123	8.634521531	-6.113520714	-3.540078350
	C124	9.620938469	-6.237692850	-2.366022783
	C125	7.203885070	-6.380132279	-3.045740797
	C126	6.098789113	-6.131745696	-4.023329830
	C127	5.944175028	-6.686617639	-5.276780880
	C128	4.109895549	-5.566253126	-4.747265983
	C129	10.518115018	-7.534359036	-0.526641648
	C130	11.953603615	-7.194640892	-0.862481508
	C131	0.605427111	-6.894995448	-0.333281015
	C132	2.021673295	-6.985779916	0.221775396
	C133	3.143376632	-7.074831126	-0.854293959
	C134	3.163284163	-8.487593222	-1.492600688
	C135	4.382764519	-8.776057500	-2.371984839
	C136	4.488455677	-10.242424935	-2.842161630
	C137	4.511698057	-6.847685721	-0.188733915
	C138	3.963508167	-0.599556407	-1.578136752
	Fe139	4.367397649	-1.905455882	-2.948706239
	Fe140	2.546031349	0.570114900	-1.273144847
	Fe141	4.980080699	0.564663713	-0.387893039
	Fe142	4.522974141	0.709329849	-2.975491327
	Fe143	3.975389629	2.776393080	-1.291629238
	Fe144	4.639025401	-1.972117531	-0.308346517
	Fe145	2.221497497	-2.496901187	-0.898343782
	H146	-6.189560530	-7.418737138	-1.145650117
	H147	-6.166598246	-6.379310108	-2.404410237
	H148	-9.751178969	-4.787661478	-0.666945081
	H149	-8.544571123	-6.055797442	-2.283704479
	H150	-8.604351886	-7.235625922	-0.980366335
	H151	-8.161831893	-2.755703507	0.459528839
	H152	-9.249051872	-5.740765669	2.035818634
	H153	-9.879196662	-3.041926249	0.812467974
	H154	-8.864528067	-6.529082973	4.180712395
	H155	-6.536963424	-5.935520839	2.263618567
	H156	-8.840111075	-4.853032503	4.769696511
	H157	-3.429969783	-1.842385362	2.656243689
	H158	-5.506273046	-3.751908699	2.079709995
	H159	-4.232188182	-6.276247884	2.421111158
	H160	-6.079026659	-0.407936798	6.295601873
	H161	-6.602480220	-2.450866854	4.330387127
	H162	-4.346339156	-6.410192479	4.190719922
	H163	-5.105482797	-1.496838428	2.167755389
	H164	-5.676508607	-4.004058748	6.518147208
	H165	-7.284723219	-1.719995859	6.417593895
	H166	-0.980539267	-3.290327718	3.209598603
	H167	-0.880580444	-2.116106509	1.882585093
	H168	0.575778517	-2.514344569	2.817740007
	H169	-1.905750742	-1.029561014	3.885624631
	H170	-5.098416809	-5.204495591	8.415413476
	H171	-2.860691774	1.874232017	4.974849877
	H172	-3.137273337	-0.672888556	8.718203888
	H173	-3.471885792	2.764227948	9.199031613
	H174	0.952956978	-0.020191970	3.355425891
	H175	-0.361103318	0.938366678	4.082794562
	H176	8.360187448	1.724533939	6.165838738
	H177	-3.880327603	3.225123749	5.501496368
	H178	-4.194137680	3.753124953	7.907318641
	H179	-2.112544801	3.335111445	5.675963007
	H180	-2.445715716	3.848359812	8.229531604
	H181	-3.931857984	1.363945975	7.186954184

	H182	-0.919027437	1.893359385	7.334893983
	H183	-2.485978582	-0.220876939	6.065717146
	H184	-0.492547551	0.375272367	2.406591403
	H185	1.198664076	4.034394137	0.629741342
	H186	0.183004483	4.377540737	2.063534250
	H187	2.081961048	4.999557726	2.889563181
	H188	2.218398495	4.341177327	5.508907531
	H189	4.295436544	4.008432622	2.663364878
	H190	6.231170702	2.458970063	2.596932921
	H191	5.450244852	1.313402485	6.681523980
	H192	-2.069464598	-2.862475311	5.714802011
	H193	-1.080917984	-5.854634948	6.091586165
	H194	-0.726926615	-5.325672351	7.749755493
	H195	-3.200159231	-4.710217855	6.211315266
	H196	-4.774223966	-3.689629093	9.296066702
	H197	3.491531677	2.832771711	6.734872811
	H198	0.674949879	-1.852158121	5.266826098
	H199	-2.990795108	0.094198568	11.381565152
	H200	-3.012600093	-1.596926789	10.811273982
	H201	-0.320819410	-1.190045727	10.542959897
	H202	10.230923600	-7.278042624	5.661931844
	H203	-0.969700003	-5.789410269	-4.630504436
	H204	1.496005773	-6.006109801	-1.789647056
	H205	-0.694212019	-5.850292127	-9.013616850
	H206	0.846937000	-4.970213174	-9.183651286
	H207	-1.742211172	-3.997222116	-7.844892150
	H208	-1.198148248	1.494631980	-10.149025496
	H209	-1.459601658	-3.776283782	-10.360147620
	H210	-2.839512156	-2.017399918	-2.998180215
	H211	-3.674216521	-4.826400806	-2.107103664
	H212	-0.635144320	-0.635466217	-8.764459776
	H213	-3.376969694	0.118801056	-9.553179962
	H214	0.796469976	-1.996623822	-9.696801800
	H215	-0.874168112	-5.721189309	-2.873921839
	H216	-0.216409206	-3.133701668	-7.641467382
	H217	0.780647352	-3.319694713	-10.678803553
	H218	-6.062002007	-3.191164506	-4.275203678
	H219	-3.303902699	-2.308856838	-1.307814955
	H220	2.197893366	-4.935744173	-7.523200510
	H221	-2.086601665	-4.226579204	-1.656675150
	H222	-4.319000497	2.511932331	2.418462263
	H223	-3.075298364	0.567056974	0.841586467
	H224	-8.645385984	0.677502591	2.227741755
	H225	-7.302075107	-0.020754483	-0.347876037
	H226	-7.222698868	4.079261453	1.218578119
	H227	-8.986936548	8.628513401	3.275683160
	H228	-7.637678878	5.107014119	4.226393825
	H229	-9.377351995	5.404826018	2.051117515
	H230	-6.631688212	6.790758572	1.857528051
	H231	-10.805531910	9.643589159	-0.412930081
	H232	-3.437054140	4.262895248	0.874336726
	H233	-4.796057162	4.012805722	-0.232675313
	H234	-10.219300061	10.425262392	1.946812495
	H235	-8.563278874	5.668186354	0.507481480
	H236	-6.316347596	2.064143938	0.281828591
	H237	-7.516251391	-0.667142862	2.056438264
	H238	-9.599699299	2.112277020	-1.949611719
	H239	-1.578489048	1.743027579	-2.959280429
	H240	-9.987299959	0.386448070	-2.074097291
	H241	9.646612671	3.109102561	4.703718094
	H242	8.121299466	3.218730296	4.124814617
	H243	1.720281659	5.971050225	-0.859170828
	H244	-0.891435658	5.485418470	0.306815481
	H245	1.589639859	7.947033295	0.955042363
	H246	-3.068940722	3.690779586	-2.057925586
	H247	-4.465807900	4.377520403	-3.489281085
	H248	-5.731269358	3.694112484	-5.207878528
	H249	-4.341651880	5.587865569	-2.463317011
	H250	-4.638017654	1.535260564	-4.984721789
	H251	-6.425667322	1.576908385	-5.119147491
	H252	0.310015273	11.716676893	-1.272203558
	H253	-0.871352712	13.037761856	-1.241414646
	H254	-0.275684905	10.413346686	-2.993134600
	H255	-1.938633680	9.926861917	-3.320011465
	H256	-2.540290971	9.455000233	-6.300995726
	H257	-1.526212335	5.143528594	-5.950244879
	H258	-7.026366897	8.823940262	-1.498464647
	H259	-3.422747792	8.466677138	1.912079707
	H260	-3.963674920	8.011366522	-3.406484855
	H261	1.789200328	5.578976484	-3.412348342

	H262	2.857249555	7.337373959	-2.344650979
	H263	-0.701892182	5.004173859	-3.402678045
	H264	-7.925913767	0.010384081	-3.056372638
	H265	4.306310952	10.204035948	-2.925603020
	H266	-5.383569541	-0.698684607	-4.214358073
	H267	1.512989922	6.650745011	-4.783667710
	H268	-3.362973476	7.026225282	-0.080932652
	H269	3.671616421	10.400118029	-1.279098597
	H270	-1.759544876	8.011534711	-6.856365045
	H271	-4.224992918	9.174675316	-4.660465214
	H272	-4.413518420	-3.258542393	-4.536966064
	H273	-5.501593413	-3.200205944	-2.055977592
	H274	-7.014847937	10.367065411	0.440742189
	H275	-4.822004983	1.553193305	-2.503199623
	H276	0.187120924	8.197465809	-3.366885708
	H277	-0.587916950	6.643004575	-5.968754755
	H278	0.287256200	4.463327625	-4.751118029
	H279	-2.799746113	6.099903952	-3.995757054
	H280	0.223411951	8.695774160	3.421436784
	H281	-0.482273742	9.754090450	0.437107425
	H282	-0.122726415	12.585783274	0.987304514
	H283	1.829489654	9.218496494	2.858194404
	H284	-2.725208664	10.575633982	1.709161877
	H285	6.212481114	-2.646539926	-8.320866258
	H286	6.037341351	-4.124891579	-7.356814169
	H287	8.711681793	-5.065447292	-3.865343572
	H288	6.869953324	-4.697604168	-9.522633306
	H289	5.307419627	-5.187086596	-9.505342460
	H290	5.560207612	4.803657680	0.301246350
	H291	5.631614910	7.406058115	1.882141784
	H292	5.365315043	8.239665920	-2.280734967
	H293	4.095532269	5.637090568	0.916947352
	H294	6.846404472	8.048407644	0.992386713
	H295	6.697774583	2.974502747	-5.699912152
	H296	6.248530583	6.810606109	-0.926663273
	H297	4.342689873	-7.280811018	5.594369735
	H298	6.460907755	-8.070781992	3.519642493
	H299	4.884347946	1.617550265	-5.970352502
	H300	4.321505674	-0.284950069	3.934463329
	H301	2.178343497	1.897121614	-8.310132176
	H302	-0.356628321	-1.924142596	-4.555293296
	H303	1.775802353	2.384703057	-6.660547335
	H304	3.473470180	-4.698861197	5.112396598
	H305	3.296076649	0.309294502	-6.013631347
	H306	3.314770039	-5.235664320	3.429005350
	H307	4.089121058	-7.501143166	3.846380524
	H308	5.831901449	-5.678423531	3.430890682
	H309	5.843273759	-5.339921107	5.168882604
	H310	-3.905829876	-0.428543111	-7.338514487
	H311	4.910644173	-9.751123104	5.447055336
	H312	4.276468623	-11.905452455	3.405686513
	H313	0.912871689	0.149984505	-3.956475634
	H314	5.107315627	-12.171601199	6.193929972
	H315	3.639373197	-12.431165191	5.515901176
	H316	5.106877327	-3.513743030	2.899486050
	H317	0.160543123	2.339744037	-4.794211909
	H318	-2.309250005	-1.779117397	-6.082228164
	H319	7.815940989	-9.892496280	-0.675298243
	H320	7.131484819	-7.431712634	-2.719330085
	H321	6.515462749	-8.214359497	0.753059667
	H322	7.166053543	-10.452184333	-1.971422954
	H323	6.496684700	3.008029663	-8.476597216
	H324	6.294009813	-5.095227626	0.703547261
	H325	2.078996563	-7.830672938	0.918110271
	H326	4.648023098	-1.708421704	-6.951432869
	H327	3.139804146	-9.210222972	-0.661932722
	H328	4.241963254	-6.611261672	-6.574355389
	H329	3.103367428	-5.176251681	-4.825232467
	H330	5.769625116	-12.648260591	3.991685164
	H331	5.258387660	-1.213380062	2.818835134
	H332	8.065056125	-5.888946577	2.061427328
	H333	7.102843823	-3.814674509	1.031351517
	H334	8.297431638	-7.043194097	4.085641699
	H335	2.634485644	-2.946033485	5.513942127
	H336	4.344160788	-8.138531764	-3.268719184
	H337	5.307350340	-8.518981201	-1.837643030
	H338	2.236359723	-8.625844887	-2.069870370
	H339	2.479656526	-1.911532384	-5.849697085
	H340	-1.795440263	2.493369170	-6.358065143
	H341	-3.785362247	1.348281287	-7.406741570

	H342	2.165784921	-6.064851238	0.812252245
	H343	8.237678647	-7.414370052	1.807392578
	H344	7.160289411	-9.330533539	1.604061482
	H345	3.047736966	-1.267867212	5.597308971
	H346	6.604391013	-7.319089717	-5.856583734
	H347	7.024838397	-5.776528098	-2.149314356
	H348	1.847189753	-2.623757022	-7.364635002
	H349	5.605607192	4.367673024	-5.882029199
	H350	6.961717881	-0.054464117	5.206133443
	H351	7.326375295	0.487588722	3.554106154
	H352	9.946369941	-6.728237718	-4.990002218
	H353	9.019462116	-7.945782233	-4.409848858
	H354	10.501205493	-8.587486973	-0.203929326
	H355	9.002926123	-8.187334850	-1.877196908
	H356	12.238506526	-7.368570779	-1.928090551
	H357	10.180092117	-6.918737940	0.322309544
	H358	10.006840513	0.243132196	4.013321283
	H359	-3.598773475	12.990992642	3.775471867
	H360	9.049963026	-7.189205270	6.994502916
	H361	8.523558319	-4.725586371	5.896591275
	H362	-4.875212481	12.301985987	1.110220051
	H363	-4.670424316	10.156621864	3.317519364
	H364	-6.172045132	10.881132110	2.712823620
	H365	1.124409760	-3.485740754	-0.688667357
	H366	3.965393916	-1.364173085	0.909425276
	H367	-5.997934899	7.029310337	-2.479958192
	H368	0.389039193	-0.539055969	0.106774511
	H369	2.668593277	-1.596052557	0.267647020
	H370	1.522114705	-2.469896218	0.409837564
	H371	-1.585958584	-0.051007541	-1.181010665
	Mo372	4.115518653	-4.319164559	-1.776132001
	N373	-6.747492348	-7.076165330	-1.931992336
	N374	-8.922742509	-4.651623966	-0.092641332
	N375	-8.795179098	-5.094595227	2.677406375
	N376	-6.128456893	-5.888790116	3.198904624
	N377	-4.767171056	-3.467006104	2.718656237
	N378	-5.899526151	-1.957163104	4.886776771
	N379	-5.208700881	-3.477391842	7.261685329
	N380	-2.591438079	-4.744170338	7.032477119
	N381	-1.056922577	-2.793108791	5.782473938
	N382	-1.663065955	0.100929778	6.582939041
	N383	-2.452399977	-0.244925592	9.345659181
	N384	4.449499914	-11.809686710	5.499853439
	N385	5.542064470	-9.507931232	4.676604286
	N386	4.457092007	-3.495937584	3.690109132
	N387	3.233535540	-2.181761938	5.194498789
	N388	4.711131066	-1.193095147	3.698884577
	N389	8.188478926	-7.211596959	5.110244774
	N390	-5.180937794	-2.695183259	-4.126467264
	N391	-1.342823073	-5.441080428	-3.740856382
	N392	-5.550259599	-0.237983008	-3.303273470
	N393	-8.002035406	0.848131212	-2.483446331
	N394	-8.009767746	0.369259765	0.283865440
	N395	-5.994236375	2.061673700	1.254205035
	N396	-2.820318818	2.813824741	-1.527331742
	N397	-2.147981542	0.810231579	-1.096293140
	N398	-6.720524906	4.678519913	1.868938640
	N399	-9.848210595	7.798928175	0.167214820
	N400	-9.998190758	9.503678573	1.579009200
	N401	8.907198009	2.573191085	4.243054779
	N402	6.292807031	7.197673353	1.127012429
	N403	4.638126385	8.581779793	-1.658038037
	N404	2.109261105	7.992173217	-2.600215350
	N405	-2.259484115	6.643624408	-4.692484277
	N406	-2.248678152	8.500819235	-6.115713695
	N407	-3.608892175	8.482064191	-4.242369994
	N408	0.757985159	6.311240518	-0.674685293
	N409	0.726128292	8.090203255	1.485568455
	N410	-0.478006972	10.563126574	1.057819457
	N411	-1.284902193	10.564401278	-2.867980478
	N412	-3.004107207	11.553670465	1.716715752
	N413	5.906299041	-4.356340018	-9.514223138
	N414	3.887324308	-2.251197082	-7.369427338
	N415	2.760022580	0.527905238	-6.868928370
	N416	4.854399852	2.481857400	-6.517179178
	N417	0.210703544	-2.542256239	-10.339345322
	N418	-1.619332854	-0.767686590	-9.017339347
	N419	8.995228450	-6.960567421	-4.690246043
	N420	4.938150352	-5.421440945	-3.711228066
	N421	4.691101579	-6.315386116	-5.711455600

	N422 9.646173672 -7.414393295 -1.683129647<br>O425 -6.722060193 -5.153276911 0.214043195<br>O426 -7.956285630 -2.991709563 2.963821902<br>O427 -6.472087553 -5.440487962 5.411832653<br>O428 -3.245512863 -4.110152837 4.286790788<br>O429 -3.941883300 -0.799763854 4.886211542<br>O430 -4.931385002 -1.439658445 8.266126196<br>O431 -2.408329273 -4.488061901 9.305895800<br>O432 0.885956936 -3.929086413 6.261190066<br>O433 0.600933297 -0.033519543 6.855628230<br>O434 -0.993419911 1.441593552 9.772795815<br>O435 -0.814744823 -0.802700563 12.485770470<br>O436 6.580479963 -10.483224073 2.855530125<br>O437 6.877395949 -8.078992131 6.788304287<br>O438 10.511812925 -4.778223129 6.348559516<br>O439 -5.522077922 -0.759684889 -1.068960422<br>O440 -2.860987890 -4.151574531 -4.838567145<br>O441 -6.879092690 2.627444836 -1.597607887<br>O442 -5.557557435 3.306737654 -4.331209822<br>O443 -10.001363371 1.490912637 0.491037757<br>O444 -6.275171947 1.263673110 3.376329803<br>O445 -4.853765314 4.915360674 3.161171685<br>O446 -7.357582430 7.124434574 4.354593854<br>O447 9.871970394 -0.348843311 5.962062001<br>O448 2.699855396 4.381076395 4.662024812<br>O449 3.661577396 8.393229079 0.397438837<br>O450 1.604552119 10.219546216 -2.893355392<br>O451 -1.160451371 7.241900014 -1.501603369<br>O452 -1.149345997 7.044179652 2.291170795<br>O453 2.009059520 5.390163070 1.995480815<br>O454 0.401249928 11.399541909 3.001514324<br>O455 -2.523185700 13.598309239 0.830121234<br>O456 -2.942269931 11.134170599 -1.402651756<br>O457 -5.490677071 13.598325783 3.300594440<br>O458 -5.127102067 7.007231460 -2.037769279<br>O459 3.347994308 -4.197682310 -8.463402771<br>O460 1.347549245 -0.371568065 -8.434706169<br>O461 3.703652705 3.922855078 -7.842676845<br>O462 7.303295526 4.768687594 -7.833770232<br>O463 -3.242758575 -2.175853123 -9.778694901<br>O464 -0.331369160 -6.331534452 -6.460986453<br>O465 1.682404954 -5.443377341 -6.822525061<br>O466 -2.300244429 2.737136329 -8.969154863<br>O467 10.398246183 -5.311194089 -2.063865519<br>O468 12.776380968 -6.831734663 -0.033425518<br>O469 0.488964213 -6.227320468 -1.489241985<br>O470 -0.373801266 -7.346432230 0.258280943<br>O471 5.644551852 -10.791158212 -2.783479207<br>O472 3.437609205 -10.791155694 -3.279027616<br>O473 5.240129858 -5.875634741 -0.667802155<br>O474 4.887023440 -7.591761335 0.737900395<br>O475 2.907736542 -6.056978829 -1.830062812<br>O476 8.594906036 -6.666190977 2.363208065<br>O477 6.771039382 -4.636337250 1.440980712<br>O478 -3.811901959 4.938555246 -2.990516566<br>O479 8.051473136 -10.357638142 -1.508974043<br>O480 7.388197242 -8.668535361 0.902827484<br>S481 3.854857061 5.062485345 -1.428036879<br>S482 2.616117747 1.976660521 -2.952023949<br>S483 2.476407531 -3.165884979 -3.075448208<br>S484 3.228400515 1.672800695 0.526817543<br>S485 0.699795460 -0.765334504 -1.200399880<br>S486 5.037724026 -0.618738963 -4.663679237<br>S487 3.508700659 -3.771101190 0.383828953<br>S488 6.036797972 1.924142684 -1.775665924<br>S489 6.051024611 -2.970182298 -1.917836417<br>S490 6.272800755 -0.811154719 0.740260859<br>end

Product (10x)

Fe(139) -2.529<br>Fe(140) -1.444<br>Fe(141) -2.302<br>Fe(142) 2.801<br>Fe(143) 2.831<br>Fe(144) 0.483	bm522bh6hx7hn6h2ntq.car_5<br>C1 -7.776616653 -5.368336208 -0.373353587<br>C2 -7.927235983 -6.467777429 -1.429237732<br>C3 -8.887648400 -3.523379548 0.805547650<br>C4 -8.508853726 -3.859819072 2.245170745<br>C5 -8.437371600 -5.551359557 4.012702003<br>C6 -6.929075634 -5.625519959 4.270539240<br>C7 -4.682651645 -5.838953600 3.333635239<br>C8 -4.158356778 -4.408851589 3.499771017

Fe(145) 0.980	C9	-4.480149886	-2.058550678	2.906693534
	C10	-4.748111410	-1.561436983	4.333678161
S=1/2	C11	-6.234984176	-1.518526541	6.247229704
	C12	-5.395479837	-2.165450388	7.358465157
	C13	-4.623982207	-4.261853875	8.371560506
	C14	-3.108993003	-4.500548630	8.285800869
	C15	-1.202071914	-4.991809769	6.800507937
	C16	-0.375178104	-3.829272813	6.250840953
	C17	-0.391655248	-1.564983260	5.307426083
	C18	-0.450327685	-0.415345962	6.331025880
	C19	-0.795051036	-1.159576219	3.868468704
	C20	-0.062619549	0.116539695	3.435484315
	C21	-0.510752187	-2.307532910	2.893043766
	C22	-1.810543848	1.259354718	7.500310222
	C23	-1.730097662	0.830729164	8.977865023
	C24	-3.087217479	2.069813062	7.167510196
	C25	-3.327196722	3.175332539	8.201586261
	C26	-2.986613942	2.665336788	5.756903705
	C27	-2.492991293	-0.651172537	10.740873510
	C28	-1.102854048	-0.884721622	11.297523258
	C29	5.057584340	-11.808926233	4.126134574
	C30	5.824150146	-10.527818536	3.815420373
	C31	6.028236805	-8.144358354	4.503043388
	C32	7.093732883	-7.829363483	5.570422899
	C33	4.839319765	-7.172146661	4.603227231
	C34	5.241671438	-5.722207007	4.343169087
	C35	4.031214136	-4.804363288	4.185676948
	C36	4.126488981	-2.299643543	4.241289114
	C37	9.292787094	-6.812067990	5.960386395
	C38	9.433684445	-5.309577408	6.074764076
	C39	-4.921419550	-2.484113235	-2.705977611
	C40	-5.347779331	-1.068915269	-2.272772633
	C41	-3.430365692	-2.665916221	-2.345786468
	C42	-2.892185262	-4.109745281	-2.409064714
	C43	-2.366243646	-4.563780828	-3.771465918
	C44	-5.625173552	1.224772896	-3.132846395
	C45	-6.875220008	1.650875734	-2.349838368
	C46	-5.535670839	1.896624539	-4.509494035
	C47	-9.195265799	1.121082725	-1.761601718
	C48	-9.082650531	1.011720186	-0.237001823
	C49	-7.710762276	0.343707824	1.681499988
	C50	-6.578156600	1.248801210	2.168691062
	C51	-4.954882498	3.010092187	1.673428309
	C52	-5.522391301	4.279250761	2.351329483
	C53	-4.120612921	3.527339330	0.492516484
	C54	-3.268249289	2.543573372	-0.235484260
	C55	-2.735576711	1.315732485	0.072896236
	C56	-1.987370230	1.882054723	-1.942639445
	C57	-7.300065511	5.976855373	2.235764412
	C58	-7.425091077	6.078532314	3.754685643
	C59	-8.687628442	6.077904729	1.557973659
	C60	-9.261002521	7.456319701	1.401760835
	C61	-9.342042858	8.508111334	2.293403561
	C62	-10.278624521	9.059876218	0.362464046
	C63	8.512093929	1.484623394	5.101427203
	C64	9.578421361	0.406770258	5.023564369
	C65	7.199293982	0.850693255	4.575796735
	C66	6.018664115	1.791978597	4.620120648
	C67	5.214187636	1.904280295	5.768908527
	C68	5.676507107	2.567958768	3.500215642
	C69	4.094943561	2.743929290	5.794014292
	C70	4.572087486	3.420711653	3.514781986
	C71	3.774362895	3.498877735	4.659239056
	C72	5.519365754	6.946018706	-0.106358407
	C73	4.501007120	8.051194957	-0.423867318
	C74	4.811228333	5.597490690	0.029539967
	C75	3.771410786	9.716941287	-2.098649730
	C76	2.369973472	9.335651169	-2.579390335
	C77	0.792874983	7.440006401	-2.941937203
	C78	0.034139767	6.998503049	-1.666915466
	C79	1.033932800	6.261273157	-3.914988539
	C80	-0.150958498	5.375627219	-4.320472370
	C81	-1.140051366	5.960124103	-5.339608195
	C82	-2.721066539	7.865524172	-5.053030166
	C83	0.062593036	5.898284828	0.510052260
	C84	-0.212467378	7.062214851	1.478288891
	C85	0.827190720	4.802053581	1.273049965
	C86	0.749758102	9.051687883	2.536726056
	C87	0.197246830	10.438301497	2.229046294
	C88	-0.863260194	11.900791041	0.578064151

C89	-2.228443701	12.438366444	1.067160187
C90	-0.736658321	12.020992340	-0.944861216
C91	-1.776129192	11.221041641	-1.724046764
C92	-4.414582270	11.893290484	2.032186760
C93	-4.514103909	12.923178157	3.147894782
C94	-5.172065857	10.597270047	2.439376447
C95	-5.210280305	9.586116978	1.315199546
C96	-6.216350842	9.655837519	0.338832444
C97	-4.216992974	8.604122187	1.170179573
C98	-6.217863491	8.799368352	-0.763708769
C99	-4.188951168	7.753546607	0.060281042
C100	-5.189350301	7.866499286	-0.900191106
C101	5.673520550	-3.623125746	-8.245548885
C102	4.187066062	-3.387408478	-8.044773980
C103	2.520647694	-1.901242281	-6.953498205
C104	2.139398797	-0.512805741	-7.477115653
C105	2.542599515	1.898675920	-7.231256386
C106	3.760555810	2.817870315	-7.221434431
C107	5.976994426	3.376347431	-6.409288749
C108	6.606177771	3.764060335	-7.730873198
C109	-0.962168657	-3.059764991	-9.665780517
C110	-2.024937981	-1.966837164	-9.508264013
C111	-0.764214495	-3.798450727	-8.321754302
C112	-0.030081727	-5.137427656	-8.527185432
C113	0.407559395	-5.716865083	-7.200103657
C114	-2.535386358	0.330568355	-8.849049878
C115	-1.946266066	1.632449582	-9.355452651
C116	-3.165549645	0.412072774	-7.436539005
C117	-2.132563753	0.337188651	-6.342456639
C118	-1.427720490	1.481369139	-5.931435495
C119	-1.777419729	-0.907111817	-5.791897577
C120	-0.366151900	1.371841714	-5.027751495
C121	-0.715813798	-1.016077978	-4.888372927
C122	0.008096869	0.121383271	-4.520348044
C123	8.621208877	-6.135713010	-3.543328269
C124	9.608369175	-6.239538020	-2.370489584
C125	7.188452493	-6.335366421	-3.021931560
C126	6.092064949	-6.120120202	-4.015150988
C127	5.969802309	-6.672727356	-5.273622003
C128	4.103352228	-5.597435443	-4.765031764
C129	10.502920870	-7.515367251	-0.518885424
C130	11.939207919	-7.177689583	-0.854344335
C131	0.583445308	-6.977893215	-0.352816304
C132	1.994957767	-7.077274720	0.213392376
C133	3.137347585	-7.148519385	-0.843653688
C134	3.185524872	-8.560723456	-1.480619829
C135	4.407550659	-8.830669885	-2.363214635
C136	4.527653610	-10.289711752	-2.858258171
C137	4.482492853	-6.882641123	-0.143878672
C138	3.963069464	-0.566372161	-1.568557388
Fe139	4.285274042	-1.926403294	-2.914481052
Fe140	2.617581950	0.708026807	-1.225030492
Fe141	5.125056032	0.572690337	-0.483372639
Fe142	4.554654088	0.678914336	-3.026446085
Fe143	4.138659199	2.822877987	-1.410954546
Fe144	4.509202288	-1.960029563	-0.269610168
Fe145	2.073244086	-2.683027597	-1.151306888
H146	-6.136910224	-7.395138319	-1.126448473
H147	-6.124779753	-6.355367978	-2.384577722
H148	-9.734469735	-4.805658650	-0.676590412
H149	-8.507202748	-6.071295759	-2.278083980
H150	-8.556247593	-7.254262451	-0.977158828
H151	-8.169006515	-2.765239130	0.461488528
H152	-9.250537118	-5.754892855	2.032644078
H153	-9.885133276	-3.063842577	0.809562078
H154	-8.871587484	-6.546762341	4.176304930
H155	-6.537387908	-5.937510702	2.268276607
H156	-8.852607768	-4.872542892	4.769500571
H157	-3.426459023	-1.852589567	2.683554001
H158	-5.513679757	-3.752553447	2.098286954
H159	-4.233744552	-6.289632013	2.437781047
H160	-6.086089505	-0.433826432	6.325009533
H161	-6.605382631	-2.459227155	4.342944777
H162	-4.356571793	-6.415848838	4.207364705
H163	-5.099993019	-1.497538193	2.194964497
H164	-5.705873110	-4.036193623	6.539896605
H165	-7.294211087	-1.744456467	6.431795889
H166	-1.000696299	-3.240516061	3.205167847
H167	-0.875941702	-2.062287763	1.883281646
H168	0.572052986	-2.497214820	2.815385014

H169	-1.876608765	-0.950904895	3.875262626
H170	-5.112108965	-5.246437839	8.424495336
H171	-2.866574640	1.890508170	4.987393597
H172	-3.151068078	-0.672371971	8.715656668
H173	-3.491361844	2.773471088	9.211813736
H174	1.027699504	-0.040450014	3.409707236
H175	-0.267405473	0.966239194	4.103008789
H176	8.365452180	1.737664499	6.168937716
H177	-3.892609182	3.238055244	5.512879304
H178	-4.213254698	3.762997830	7.920331167
H179	-2.126116048	3.351874197	5.693571533
H180	-2.465436184	3.860314011	8.245180169
H181	-3.946596036	1.375619670	7.196764041
H182	-0.934901811	1.910131939	7.356824278
H183	-2.493091520	-0.210253536	6.079978373
H184	-0.376429964	0.412459415	2.422186706
H185	1.136534416	4.014675730	0.562077259
H186	0.124951261	4.363464724	1.998366095
H187	2.049589154	4.935118861	2.818904647
H188	2.182737611	4.285110946	5.446907700
H189	4.304529132	3.998229373	2.630304256
H190	6.261935619	2.478506914	2.582895852
H191	5.452753923	1.311566564	6.656000476
H192	-2.083914088	-2.824042065	5.721931610
H193	-1.099539717	-5.829909725	6.094342824
H194	-0.739741871	-5.294558643	7.748912450
H195	-3.217085566	-4.678522131	6.219383161
H196	-4.801139489	-3.736445053	9.317630697
H197	3.467929621	2.798628546	6.688314646
H198	0.670182368	-1.833363146	5.284898785
H199	-3.017543222	0.074627749	11.383955274
H200	-3.029082833	-1.611865459	10.800502824
H201	-0.339311565	-1.194814820	10.546028624
H202	10.249524722	-7.247000352	5.633242363
H203	-0.984040427	-5.802476013	-4.637777112
H204	1.492378297	-6.137036449	-1.828193837
H205	-0.708488853	-5.856545625	-9.007753925
H206	0.841290686	-4.992564904	-9.182354983
H207	-1.739333942	-3.992394509	-7.848716295
H208	-1.206925463	1.509675596	-10.181161141
H209	-1.428275577	-3.771834498	-10.363796355
H210	-2.829271024	-2.013719511	-3.000071861
H211	-3.681674587	-4.821203807	-2.110666730
H212	-0.612220909	-0.625110500	-8.783951803
H213	-3.364337290	0.122552168	-9.556452155
H214	0.821662473	-1.992058133	-9.677074563
H215	-0.903182988	-5.759920641	-2.881741046
H216	-0.206096836	-3.147003692	-7.631841521
H217	0.821593807	-3.324316301	-10.646723015
H218	-6.058373304	-3.161298023	-4.291818898
H219	-3.302069135	-2.303403100	-1.315381772
H220	2.192462747	-4.955309072	-7.519083813
H221	-2.093392502	-4.227082238	-1.661506118
H222	-4.319390397	2.523439926	2.426313156
H223	-2.859265431	0.686906340	0.945853851
H224	-8.622553405	0.665306095	2.204519305
H225	-7.276299890	-0.010342175	-0.374934832
H226	-7.237475263	4.078993172	1.241145696
H227	-8.987423886	8.633462413	3.310314060
H228	-7.632511750	5.101916138	4.255062779
H229	-9.395217247	5.414895922	2.086284996
H230	-6.645731909	6.790244625	1.883652745
H231	-10.791199799	9.666086719	-0.380802908
H232	-3.466398385	4.318124540	0.895740942
H233	-4.793447975	4.009388044	-0.234379605
H234	-10.203035900	10.441167912	1.981028173
H235	-8.584221824	5.671798783	0.539638772
H236	-6.305689152	2.080534940	0.277000103
H237	-7.496116118	-0.680048978	2.021308103
H238	-9.569884237	2.133438306	-1.966818913
H239	-1.466573453	1.845303722	-2.896454083
H240	-9.957041326	0.408302623	-2.102775586
H241	9.650367388	3.149380097	4.734695568
H242	8.127722968	3.254977768	4.148146228
H243	1.698748510	5.972679424	-0.903616010
H244	-0.925461588	5.496313808	0.240567775
H245	1.556915187	7.946352574	0.923787945
H246	-3.074782609	3.717191835	-2.044769524
H247	-4.468456314	4.404860984	-3.495605433
H248	-5.706677984	3.709976661	-5.231588229

	H249	-4.342283639	5.614347573	-2.466871008
	H250	-4.601096162	1.566772980	-4.992852872
	H251	-6.388625855	1.585434767	-5.132743362
	H252	0.286961263	11.759477206	-1.248820542
	H253	-0.893712159	13.080353019	-1.202475640
	H254	-0.299297893	10.452935308	-2.970022414
	H255	-1.963306498	9.977025441	-3.303789584
	H256	-2.564194667	9.464273575	-6.328383874
	H257	-1.545852516	5.151954476	-5.967727734
	H258	-7.016210277	8.857948185	-1.507387235
	H259	-3.440863066	8.478167951	1.929605201
	H260	-3.962686944	8.031628610	-3.418921015
	H261	1.787403344	5.604835913	-3.450408102
	H262	2.846474417	7.361769808	-2.371458114
	H263	-0.697361401	5.016066826	-3.433997417
	H264	-7.892138335	0.037826496	-3.080674113
	H265	4.272921272	10.251456793	-2.915713040
	H266	-5.354491094	-0.673785240	-4.226195286
	H267	1.504486363	6.676090293	-4.821438326
	H268	-3.369966362	7.044496974	-0.068111993
	H269	3.628145646	10.427718634	-1.271441105
	H270	-1.790615753	8.018785743	-6.885486774
	H271	-4.226702688	9.194802040	-4.672815613
	H272	-4.410194885	-3.242595150	-4.549528844
	H273	-5.505990870	-3.176110746	-2.071012925
	H274	-7.018169364	10.390984529	0.439284522
	H275	-4.787127473	1.577289237	-2.513264156
	H276	0.178200499	8.220076138	-3.403491998
	H277	-0.606777522	6.650748335	-6.005083847
	H278	0.286862144	4.481930207	-4.788472262
	H279	-2.801115522	6.119126551	-4.008545338
	H280	0.188070796	8.662959897	3.399008387
	H281	-0.510981575	9.775749345	0.428372696
	H282	-0.134193035	12.595282653	1.022872652
	H283	1.795456560	9.189784024	2.845392454
	H284	-2.749203739	10.590523327	1.716209713
	H285	6.200454458	-2.658379726	-8.290641477
	H286	6.020628278	-4.133120218	-7.322879493
	H287	8.725198057	-5.105963985	-3.915839735
	H288	6.875390939	-4.716377009	-9.476859563
	H289	5.310979829	-5.202308691	-9.478312378
	H290	5.572492252	4.841469881	0.258645518
	H291	5.581197607	7.418294192	1.880924970
	H292	5.343856848	8.286438036	-2.283503226
	H293	4.077537379	5.635872997	0.846297805
	H294	6.809910300	8.078306347	1.023526616
	H295	6.754639911	2.894129224	-5.796844226
	H296	6.251081532	6.875691379	-0.926617378
	H297	4.372375081	-7.278580036	5.597019414
	H298	6.471253160	-8.065996338	3.501913615
	H299	4.920451908	1.555709887	-6.034517863
	H300	4.342704237	-0.272080847	4.055617005
	H301	2.130080568	1.902210155	-8.247482587
	H302	-0.434776403	-1.992869814	-4.488984038
	H303	1.790438506	2.378560145	-6.582343372
	H304	3.499969973	-4.707522510	5.140326916
	H305	3.270918178	0.284856826	-5.980238702
	H306	3.324402652	-5.222110713	3.450538432
	H307	4.100871631	-7.493278783	3.851014569
	H308	5.837468416	-5.669486157	3.420963405
	H309	5.873159522	-5.338898337	5.160114474
	H310	-3.878856914	-0.419670532	-7.342328112
	H311	4.907114842	-9.749121469	5.413901296
	H312	4.274710842	-11.885984762	3.356438930
	H313	0.863492797	0.051677591	-3.845760202
	H314	5.072235463	-12.181718261	6.150966233
	H315	3.609247206	-12.417819820	5.455522172
	H316	5.114480968	-3.492794590	2.930076396
	H317	0.201694596	2.254912191	-4.724784427
	H318	-2.330508902	-1.804151813	-6.077815677
	H319	7.813167496	-9.861334221	-0.692195963
	H320	7.091055205	-7.361098833	-2.627521849
	H321	6.503145231	-8.204464819	0.762840194
	H322	7.181981908	-10.445028142	-1.990431292
	H323	6.445007356	3.038398013	-8.563098120
	H324	6.219640752	-5.084019075	0.752115627
	H325	2.043807145	-7.934723709	0.896176109
	H326	4.626024688	-1.731493035	-6.928726542
	H327	3.182421151	-9.277312232	-0.644767678
	H328	4.291923762	-6.623147856	-6.599561157

H329	3.091317825	-5.225701688	-4.859725422
H330	5.753932444	-12.647011065	3.953681059
H331	5.303936027	-1.178658752	2.924688493
H332	8.062154372	-5.867121134	2.057328298
H333	7.035847524	-3.796503803	1.032564129
H334	8.306028445	-7.030915809	4.069491714
H335	2.610439739	-2.964462892	5.527129792
H336	4.369706716	-8.182766493	-3.253077125
H337	5.328150179	-8.576195403	-1.820441210
H338	2.257522569	-8.724193390	-2.051648894
H339	2.453703263	-1.936298646	-5.854376459
H340	-1.698051496	2.453070941	-6.352392332
H341	-3.730935437	1.355462795	-7.392878666
H342	2.132188707	-6.170416523	0.826697718
H343	8.240830795	-7.389589902	1.796223725
H344	7.183710582	-9.315973281	1.588376221
H345	3.039535147	-1.296024050	5.661135275
H346	6.656722662	-7.281944794	-5.847546929
H347	7.026675237	-5.669409040	-2.165642735
H348	1.827584950	-2.628065980	-7.381488946
H349	5.656091382	4.289517756	-5.884024806
H350	6.990239477	-0.039957194	5.186609177
H351	7.362602031	0.514558210	3.539072436
H352	9.900575125	-6.861413002	-4.972919472
H353	8.926422212	-8.017238498	-4.345844295
H354	10.488201505	-8.566053250	-0.187973566
H355	9.007014045	-8.194875412	-1.878039108
H356	12.224384137	-7.355582800	-1.919328124
H357	10.161206992	-6.894300712	0.324411425
H358	10.039736949	0.293164117	4.012333712
H359	-3.619937279	12.988834381	3.811901943
H360	9.075451258	-7.196367414	6.972607876
H361	8.488502332	-4.735473342	5.908268500
H362	-4.893387803	12.325216107	1.137510464
H363	-4.692508454	10.164857183	3.331451539
H364	-6.192903533	10.890051646	2.724240223
H365	0.749829251	-3.631997554	-1.379613528
H366	3.898956417	-1.247441591	1.130240848
H367	-5.991172506	7.057061544	-2.480271235
H368	0.657968638	-0.690184493	0.449445811
H369	3.246892739	-1.267650144	0.616243541
H370	0.801251668	-3.530054860	-0.555460573
H371	-1.284741915	0.131594133	-1.034089650
Mo372	4.035890715	-4.366159224	-1.768400417
N373	-6.695950463	-7.063346772	-1.916738036
N374	-8.913075048	-4.666545633	-0.092992807
N375	-8.799930062	-5.108921400	2.676725770
N376	-6.132740754	-5.895717622	3.205587131
N377	-4.769161424	-3.473312863	2.733546478
N378	-5.902203951	-1.973999945	4.906332363
N379	-5.235118812	-3.511822856	7.283410411
N380	-2.606057027	-4.715151394	7.038517268
N381	-1.069912388	-2.765679039	5.776923677
N382	-1.670804307	0.119781693	6.592929612
N383	-2.469918713	-0.245726560	9.347794617
N384	4.426399246	-11.805277323	5.453294745
N385	5.549290841	-9.507617707	4.652002325
N386	4.466536388	-3.484343378	3.723243354
N387	3.240672742	-2.205591162	5.255549362
N388	4.697655369	-1.178543122	3.766090405
N389	8.202903673	-7.211062828	5.092847250
N390	-5.173319582	-2.673428597	-4.140144259
N391	-1.352723060	-5.449685833	-3.747636114
N392	-5.517680170	-0.213004002	-3.314659528
N393	-7.970377303	0.872663505	-2.503828292
N394	-7.986290646	0.373544191	0.258149634
N395	-5.984408411	2.070465838	1.249706333
N396	-2.784589871	2.864660120	-1.497499906
N397	-1.955522284	0.931992004	-1.002376546
N398	-6.734107006	4.678586641	1.890298243
N399	-9.849320880	7.813654486	0.199444847
N400	-9.988731750	9.518193528	1.612658432
N401	8.918177565	2.614152155	4.261627613
N402	6.257615401	7.223491691	1.135699983
N403	4.616276852	8.623568687	-1.658668888
N404	2.096249585	8.015556917	-2.625979794
N405	-2.266453103	6.659866722	-4.711836826
N406	-2.256866379	8.516519619	-6.135534814
N407	-3.609766614	8.502224265	-4.255804698
N408	0.737974679	6.313552176	-0.719905142

	N409	0.693153807	8.081280489	1.456135446
	N410	-0.505806210	10.573353187	1.063902014
	N411	-1.307905838	10.607544526	-2.844083562
	N412	-3.023443503	11.569789624	1.740493564
	N413	5.912339636	-4.373353850	-9.480748897
	N414	3.869308089	-2.264575100	-7.367816168
	N415	2.747071690	0.514841468	-6.840534044
	N416	4.882509411	2.429342698	-6.564314288
	N417	0.245623720	-2.544829565	-10.322381257
	N418	-1.595684783	-0.758759582	-9.040180124
	N419	8.942507701	-7.042331500	-4.660110108
	N420	4.912332075	-5.437420362	-3.714995756
	N421	4.715207139	-6.328682025	-5.723298511
	N422	9.636277672	-7.410472794	-1.679028730
	O425	-6.712521559	-5.152298855	0.234446551
	O426	-7.961883710	-3.005596209	2.965853522
	O427	-6.486291680	-5.462713201	5.420017017
	O428	-3.241946655	-4.121749634	4.292019498
	O429	-3.937015163	-0.829062882	4.922303162
	O430	-4.940049498	-1.477710845	8.288616329
	O431	-2.431416946	-4.544022335	9.320718897
	O432	0.868419916	-3.912916686	6.246383024
	O433	0.594373164	-0.006267898	6.864360058
	O434	-1.018582949	1.443147676	9.792506677
	O435	-0.840480920	-0.816893147	12.489214686
	O436	6.600560126	-10.485873196	2.839429024
	O437	6.906748802	-8.107948932	6.767484858
	O438	10.481374430	-4.743483043	6.343976780
	O439	-5.501410260	-0.737942533	-1.081178784
	O440	-2.863321705	-4.153000249	-4.844522383
	O441	-6.850339781	2.647067810	-1.605043249
	O442	-5.546088661	3.328973384	-4.349567100
	O443	-9.978186608	1.494141408	0.469788905
	O444	-6.241104409	1.222148836	3.356358344
	O445	-4.860944147	4.924112391	3.170358325
	O446	-7.361053701	7.120512158	4.383505551
	O447	9.907363924	-0.309187603	5.958053895
	O448	2.671153383	4.330812643	4.604414608
	O449	3.632555808	8.410001771	0.390739192
	O450	1.572860364	10.241116542	-2.895618463
	O451	-1.170806170	7.270637808	-1.536618404
	O452	-1.180707832	7.022603690	2.248514463
	O453	1.965817751	5.351761743	1.937565668
	O454	0.374383328	11.371407757	3.021716808
	O455	-2.532979606	13.625601627	0.885305148
	O456	-2.967575806	11.186302706	-1.384886271
	O457	-5.509414223	13.604490605	3.337563116
	O458	-5.119714479	7.038115664	-2.039143323
	O459	3.340990051	-4.217041692	-8.458794875
	O460	1.351044890	-0.363589581	-8.433553483
	O461	3.682710640	3.925115503	-7.779091603
	O462	7.282675244	4.769193670	-7.879189428
	O463	-3.218932629	-2.180719857	-9.775184857
	O464	-0.341037508	-6.346636137	-6.459301026
	O465	1.675127574	-5.464853620	-6.820625198
	O466	-2.272265834	2.741889554	-8.956493575
	O467	10.374919357	-5.303500463	-2.070862290
	O468	12.762417098	-6.812457486	-0.026802684
	O469	0.482492084	-6.349395161	-1.534758673
	O470	-0.406967494	-7.384533430	0.250215394
	O471	5.690539950	-10.825418517	-2.809554133
	O472	3.484579605	-10.843502567	-3.306117842
	O473	5.164253708	-5.857870282	-0.567477854
	O474	4.868388629	-7.648620029	0.762852881
	O475	2.903919989	-6.136047384	-1.829727176
	O476	8.599524860	-6.642184650	2.352652190
	O477	6.733272002	-4.619372955	1.463284489
	O478	-3.814469571	4.964938345	-2.995789298
	O479	8.063900094	-10.319204074	-1.525427649
	O480	7.389521546	-8.640229804	0.894032498
	S481	3.915343953	5.105241371	-1.517205175
	S482	2.685053955	2.014553031	-3.000825711
	S483	2.500799290	-3.265361422	-3.247405396
	S484	3.474026616	1.831044148	0.479778826
	S485	0.810039894	-0.715346127	-0.904032727
	S486	4.988615921	-0.726865534	-4.669127266
	S487	3.274973021	-3.731514536	0.375994435
	S488	6.166209668	1.875645445	-1.940439653
	S489	5.942314050	-2.957416742	-1.752641279
	S490	6.214231314	-0.860119162	0.798318456

	end

6H2x dissociation from S2BH-6H2x-7H2n 10 to 11

TS

Spin densities	2n2xs2bh7h2n6h2xdte.car_1
Fe(139) -2.395<br>Fe(140) -1.369<br>Fe(141) -2.472<br>Fe(142) 2.637<br>Fe(143) 2.808<br>Fe(144) 0.358<br>Fe(145) 1.355<br><br>**S=1/2**	C1 -7.808356706 -5.370632406 -0.368136905<br>C2 -7.941848124 -6.474519569 -1.422004517<br>C3 -8.931429273 -3.524710372 0.797220439<br>C4 -8.568971219 -3.857574349 2.242381051<br>C5 -8.524386332 -5.545369149 4.014236621<br>C6 -7.014327216 -5.663518391 4.246259782<br>C7 -4.802795342 -5.940979037 3.246434857<br>C8 -4.240349693 -4.530972158 3.438486590<br>C9 -4.465455122 -2.153849846 2.910942529<br>C10 -4.724514749 -1.656153001 4.339613591<br>C11 -6.222876977 -1.551503818 6.238694213<br>C12 -5.387777708 -2.205555097 7.347764584<br>C13 -4.609397138 -4.305718276 8.342246625<br>C14 -3.089083836 -4.505468544 8.266963397<br>C15 -1.146908614 -4.943509471 6.816721002<br>C16 -0.296304284 -3.782738400 6.299222351<br>C17 -0.320726365 -1.487428296 5.394866703<br>C18 -0.410965145 -0.314639626 6.382839236<br>C19 -0.747697987 -1.139538960 3.944380953<br>C20 -0.030126380 0.125727873 3.458322670<br>C21 -0.472675868 -2.316794246 3.001611626<br>C22 -1.837203644 1.308374880 7.541704411<br>C23 -1.770219918 0.863499714 9.015325932<br>C24 -3.121945287 2.096314695 7.193777117<br>C25 -3.375859938 3.206154565 8.219637468<br>C26 -3.019828098 2.680946490 5.778825643<br>C27 -2.523180890 -0.651959528 10.753145974<br>C28 -1.131778441 -0.887318236 11.306251720<br>C29 5.068302668 -11.829096077 4.122340462<br>C30 5.837334176 -10.546977063 3.814250857<br>C31 6.038835144 -8.162052722 4.501300980<br>C32 7.105946303 -7.848031391 5.567735356<br>C33 4.851652858 -7.187866667 4.605785768<br>C34 5.256598894 -5.738253112 4.346880926<br>C35 4.047519370 -4.817258075 4.195417896<br>C36 4.162720911 -2.314367963 4.252555684<br>C37 9.298709077 -6.817625672 5.959783443<br>C38 9.423062052 -5.313900150 6.079467292<br>C39 -4.943559743 -2.494923109 -2.725831369<br>C40 -5.376895927 -1.080176792 -2.296769575<br>C41 -3.452660972 -2.672349264 -2.357105437<br>C42 -2.910235444 -4.114897838 -2.414317944<br>C43 -2.371064252 -4.566862504 -3.771840839<br>C44 -5.665258006 1.210697605 -3.159648222<br>C45 -6.907906220 1.640538294 -2.368765575<br>C46 -5.573994260 1.885273142 -4.534780100<br>C47 -9.220958139 1.115058827 -1.753805883<br>C48 -9.095289384 1.004421723 -0.230135104<br>C49 -7.709919313 0.331290621 1.677801994<br>C50 -6.578753198 1.237609890 2.164990897<br>C51 -4.967549781 3.010936550 1.671459791<br>C52 -5.521046213 4.291701592 2.340715220<br>C53 -4.116676072 3.501941639 0.490394760<br>C54 -3.336118614 2.476833993 -0.263321994<br>C55 -2.840406162 1.232105295 0.038470269<br>C56 -2.194289611 1.727535261 -2.030322597<br>C57 -7.315493544 5.977501828 2.254793013<br>C58 -7.438350111 6.070701771 3.774265172<br>C59 -8.704621178 6.076818759 1.579521750<br>C60 -9.275366806 7.456196107 1.421407107<br>C61 -9.350146378 8.510536373 2.310649267<br>C62 -10.287590641 9.061989148 0.380073309<br>C63 8.514568032 1.486111569 5.091580927<br>C64 9.587924324 0.415188837 5.013951981<br>C65 7.205399677 0.848331754 4.565589682<br>C66 6.021250145 1.784054829 4.620642671<br>C67 5.219222083 1.884248002 5.772020760<br>C68 5.676522955 2.570894201 3.509211901<br>C69 4.100498377 2.724125130 5.808060626<br>C70 4.572914849 3.424438181 3.534962163<br>C71 3.778806225 3.491569396 4.682511788

	C72	5.533465588	6.927031515	-0.112179276
	C73	4.510671050	8.030340034	-0.435108752
	C74	4.839181993	5.572910160	0.013885493
	C75	3.786482113	9.714056035	-2.094728708
	C76	2.380093353	9.339638797	-2.567976073
	C77	0.802290718	7.440408375	-2.921082751
	C78	0.069091864	7.001472415	-1.628714332
	C79	1.030139953	6.261977948	-3.897192409
	C80	-0.158743620	5.378827536	-4.296130210
	C81	-1.147307574	5.959954583	-5.318298063
	C82	-2.732600572	7.861500769	-5.040413075
	C83	0.147886627	5.929386401	0.565193822
	C84	-0.194163361	7.107131032	1.489630733
	C85	0.982612941	4.926846362	1.386067012
	C86	0.743280911	9.094151169	2.564668423
	C87	0.185572316	10.472726930	2.249024659
	C88	-0.872243536	11.895959364	0.573809467
	C89	-2.239270731	12.436725856	1.060447681
	C90	-0.745529431	12.012025842	-0.948154282
	C91	-1.785540915	11.213857453	-1.726267316
	C92	-4.423578342	11.893479950	2.034714851
	C93	-4.522676417	12.920468803	3.153410303
	C94	-5.181391198	10.597115659	2.440116557
	C95	-5.215213547	9.584779090	1.316862792
	C96	-6.218388233	9.651382993	0.337197828
	C97	-4.219916050	8.604136729	1.176544515
	C98	-6.215749607	8.791417272	-0.762965423
	C99	-4.187297977	7.750102858	0.069333742
	C100	-5.185924640	7.858739236	-0.893591073
	C101	5.664405919	-3.639054973	-8.226126932
	C102	4.175302275	-3.406626765	-8.041394046
	C103	2.499822447	-1.912375455	-6.973767835
	C104	2.126064521	-0.516364171	-7.481308413
	C105	2.539717878	1.891359143	-7.211195212
	C106	3.769764898	2.793516989	-7.219269124
	C107	5.983249969	3.343691403	-6.396078001
	C108	6.592225564	3.750702434	-7.722278288
	C109	-0.968160042	-3.066083347	-9.665830514
	C110	-2.030128578	-1.972242948	-9.505485936
	C111	-0.775025831	-3.806754628	-8.320861437
	C112	-0.039072424	-5.145376433	-8.521977187
	C113	0.390918844	-5.726727049	-7.192783651
	C114	-2.540573179	0.327474083	-8.851491845
	C115	-1.953716472	1.628493659	-9.363817499
	C116	-3.157510664	0.413786489	-7.432550568
	C117	-2.117745313	0.348625828	-6.343032240
	C118	-1.419531736	1.498453329	-5.934673323
	C119	-1.754857235	-0.891529572	-5.787198213
	C120	-0.356591650	1.399017575	-5.030743487
	C121	-0.691021868	-0.991101930	-4.884957273
	C122	0.027443272	0.152163331	-4.522337602
	C123	8.636327322	-6.166470919	-3.551554897
	C124	9.619752724	-6.252755063	-2.375259833
	C125	7.204640357	-6.354320720	-3.025091496
	C126	6.109990163	-6.148502012	-4.020628746
	C127	5.988490409	-6.698353532	-5.280593671
	C128	4.117663215	-5.636274788	-4.761941484
	C129	10.503382139	-7.505020643	-0.503883247
	C130	11.940557335	-7.168072458	-0.836270902
	C131	0.599926969	-7.071429361	-0.386761687
	C132	2.004124080	-7.127851985	0.203074091
	C133	3.160336673	-7.185495339	-0.838056729
	C134	3.216984302	-8.596768511	-1.476618575
	C135	4.439018322	-8.862953674	-2.357563080
	C136	4.556489552	-10.321273563	-2.853474834
	C137	4.486470589	-6.907990667	-0.112570864
	C138	3.937269848	-0.580911921	-1.554068558
	Fe139	4.327676620	-1.959542051	-2.923586870
	Fe140	2.493186958	0.861549934	-1.305211343
	Fe141	5.237367239	0.503427534	-0.519350519
	Fe142	4.612264517	0.562393816	-3.029241581
	Fe143	4.319881650	2.785264463	-1.491597693
	Fe144	4.539386511	-1.967688664	-0.270104519
	Fe145	2.273502468	-2.455649619	-1.336609023
	H146	-6.143021396	-7.385673302	-1.110377642
	H147	-6.134552441	-6.347751955	-2.370440643
	H148	-9.764959118	-4.813692346	-0.688418859
	H149	-8.521653820	-6.086281814	-2.274713986
	H150	-8.563321087	-7.267224926	-0.970089089
	H151	-8.208011132	-2.768349216	0.458946840

H152	-9.342075836	-5.741487485	2.035017310
H153	-9.928691898	-3.065045265	0.789231767
H154	-8.984573162	-6.527337259	4.187203516
H155	-6.680120937	-5.964455455	2.234182264
H156	-8.906964351	-4.850168579	4.772721235
H157	-3.400242279	-1.997166132	2.702272701
H158	-5.575093083	-3.782166296	2.058686276
H159	-4.392216056	-6.378809096	2.326047779
H160	-6.058097229	-0.468431894	6.310144844
H161	-6.613639280	-2.488780654	4.335859126
H162	-4.460081682	-6.543586052	4.096594104
H163	-5.048206677	-1.546414396	2.205568219
H164	-5.698305706	-4.070147637	6.514703219
H165	-7.284647463	-1.759821304	6.429114042
H166	-0.965839838	-3.239302932	3.338795192
H167	-0.846981875	-2.095219908	1.989380204
H168	0.608108952	-2.513438324	2.916409293
H169	-1.831522105	-0.942942362	3.957125745
H170	-5.074121239	-5.301946566	8.374720320
H171	-2.895471246	1.900487883	5.015470846
H172	-3.174790798	-0.653798237	8.725367313
H173	-3.536452215	2.808673336	9.232137084
H174	1.061145070	-0.023909935	3.425935964
H175	-0.234139415	0.997653603	4.096677939
H176	8.366441751	1.734407866	6.160178081
H177	-3.926895758	3.248708365	5.527938961
H178	-4.267027758	3.783542907	7.934153063
H179	-2.160936693	3.369082269	5.711506399
H180	-2.519997232	3.898615449	8.259579230
H181	-3.973259834	1.392101277	7.224294726
H182	-0.973498593	1.979102693	7.419555211
H183	-2.459486118	-0.182669470	6.115300270
H184	-0.357944650	0.380694407	2.438681437
H185	1.398933830	4.165482316	0.710890915
H186	0.287053924	4.433414589	2.082512189
H187	2.147236056	5.132641900	2.957929319
H188	2.192242042	4.275127249	5.486623670
H189	4.306355427	4.013939634	2.657791595
H190	6.261803479	2.491459041	2.590934008
H191	5.460042951	1.284166325	6.653666205
H192	-1.994167991	-2.711172466	5.940287071
H193	-1.032902632	-5.772493504	6.101890792
H194	-0.713763494	-5.262935766	7.773704564
H195	-3.158755688	-4.665001677	6.192205569
H196	-4.805488403	-3.801867165	9.296465863
H197	3.474361514	2.769903561	6.703474630
H198	0.745981647	-1.736467323	5.373464887
H199	-3.050013164	0.062186697	11.406782134
H200	-3.054972873	-1.615796779	10.798642063
H201	-0.367995296	-1.188551763	10.551323357
H202	10.259673491	-7.240958386	5.629607417
H203	-0.989881673	-5.808881281	-4.633100307
H204	1.506232807	-6.286431551	-1.896963707
H205	-0.714932091	-5.864049164	-9.006732459
H206	0.836236355	-5.000629753	-9.171848871
H207	-1.751782465	-4.001215659	-7.851506412
H208	-1.218357560	1.504594873	-10.192896932
H209	-1.432537255	-3.776204453	-10.366540802
H210	-2.849708098	-2.021604578	-3.011374824
H211	-3.700843470	-4.827803140	-2.122885999
H212	-0.615535097	-0.623722951	-8.793538269
H213	-3.374001006	0.115242842	-9.551545128
H214	0.812941610	-1.992846869	-9.671172894
H215	-0.926053615	-5.788857149	-2.878934873
H216	-0.220650841	-3.155838151	-7.627350473
H217	0.824020061	-3.329922758	-10.633224103
H218	-6.064247948	-3.182047536	-4.319153397
H219	-3.329338775	-2.307195158	-1.327130186
H220	2.180145179	-4.973451464	-7.503447730
H221	-2.118346023	-4.229470574	-1.660318009
H222	-4.342870695	2.519408014	2.430227584
H223	-2.931161170	0.627678047	0.932240719
H224	-8.620318499	0.645470730	2.207636820
H225	-7.286399856	-0.011233605	-0.383086759
H226	-7.260222341	4.073333433	1.272763834
H227	-8.992869118	8.636406971	3.326601846
H228	-7.632007588	5.089072525	4.270433035
H229	-9.412416608	5.416377317	2.110861443
H230	-6.665615229	6.795749363	1.905806924
H231	-10.798668251	9.668776998	-0.363808016

	H232	-3.416926736	4.251590284	0.894853768
	H233	-4.769601723	4.027566847	-0.223957384
	H234	-10.203107100	10.446672973	1.995363537
	H235	-8.603310291	5.667400208	0.562370144
	H236	-6.330275357	2.090926465	0.279485454
	H237	-7.488351019	-0.693759631	2.008674601
	H238	-9.592868768	2.129143161	-1.955421117
	H239	-1.715597871	1.662761686	-3.004513270
	H240	-9.988762707	0.405968081	-2.088850999
	H241	9.649473363	3.152812721	4.735847136
	H242	8.125862989	3.263539228	4.154423054
	H243	1.750299854	6.003185451	-0.885524156
	H244	-0.808056470	5.449558488	0.310827110
	H245	1.575910111	7.996194438	0.963493940
	H246	-3.167487433	3.624968891	-2.090096471
	H247	-4.503849835	4.403552072	-3.516321370
	H248	-5.734960857	3.700716628	-5.253143108
	H249	-4.353390437	5.589123754	-2.464181132
	H250	-4.641349348	1.550981870	-5.019139665
	H251	-6.428483531	1.580594224	-5.158936528
	H252	0.278421521	11.751341382	-1.251480162
	H253	-0.903662376	13.071241448	-1.205979493
	H254	-0.306794801	10.444098965	-2.965986393
	H255	-1.970877155	9.968190772	-3.305185679
	H256	-2.577371971	9.456489005	-6.320831331
	H257	-1.549222634	5.149719709	-5.946287803
	H258	-7.012270528	8.847323547	-1.508928674
	H259	-3.444985904	8.482138252	1.937773383
	H260	-3.972411720	8.032334045	-3.405617927
	H261	1.785549879	5.603132934	-3.438488248
	H262	2.868740553	7.369319301	-2.399794791
	H263	-0.705010272	5.024015341	-3.407756704
	H264	-7.932728980	0.024530441	-3.081639635
	H265	4.285663405	10.250306375	-2.912099876
	H266	-5.388427290	-0.687568748	-4.251050378
	H267	1.495336208	6.677427312	-4.806274129
	H268	-3.367953687	7.040254039	-0.053113941
	H269	3.649219549	10.422505879	-1.264292532
	H270	-1.809319414	8.008865770	-6.877088766
	H271	-4.239261744	9.191439315	-4.663185376
	H272	-4.413474935	-3.250631022	-4.564145002
	H273	-5.528565443	-3.187985104	-2.092572362
	H274	-7.020835890	10.386650789	0.433124365
	H275	-4.826934529	1.562025399	-2.542623912
	H276	0.175646394	8.217090704	-3.372094905
	H277	-0.615272932	6.651479220	-5.983993334
	H278	0.276850209	4.483079101	-4.761849071
	H279	-2.817777769	6.113453310	-3.997922927
	H280	0.173248185	8.697679327	3.418136607
	H281	-0.498414054	9.776342413	0.452858658
	H282	-0.151027214	12.602546120	1.013101602
	H283	1.785015596	9.234695845	2.884744628
	H284	-2.758074991	10.592018403	1.719212447
	H285	6.189523873	-2.673159157	-8.269894018
	H286	6.000993759	-4.142544386	-7.295652505
	H287	8.742257026	-5.143697376	-3.942499685
	H288	6.884837860	-4.735705981	-9.434334310
	H289	5.323263883	-5.228463882	-9.451213638
	H290	5.600202406	4.832833400	0.291539943
	H291	5.564211081	7.403793451	1.875704550
	H292	5.367011975	8.292520344	-2.287144598
	H293	4.074015897	5.617442198	0.802325561
	H294	6.790162907	8.081929568	1.032956613
	H295	6.770738255	2.849387728	-5.806376040
	H296	6.277296788	6.870841019	-0.922187134
	H297	4.386600320	-7.294936941	5.600394787
	H298	6.480066114	-8.080901662	3.499631413
	H299	4.896843632	1.554102305	-5.981058989
	H300	4.384371165	-0.287949414	4.061651717
	H301	2.110989680	1.901995892	-8.220382410
	H302	-0.405012874	-1.964800548	-4.481147169
	H303	1.804311420	2.376228854	-6.547335162
	H304	3.524400622	-4.712771272	5.153676052
	H305	3.244299061	0.265186020	-5.966220692
	H306	3.332404783	-5.236148066	3.469402852
	H307	4.110843995	-7.506172427	3.854743735
	H308	5.849455172	-5.685618667	3.422944183
	H309	5.892296620	-5.358031923	5.161927785
	H310	-3.866956392	-0.419998779	-7.328272940
	H311	4.910665138	-9.765051178	5.405992203

	H312	4.291181556	-11.904168470	3.346497020
	H313	0.884455971	0.088856774	-3.849605396
	H314	5.062877311	-12.199094563	6.147548855
	H315	3.603764355	-12.426959062	5.439318027
	H316	5.119152777	-3.511738586	2.921048558
	H317	0.202333312	2.287434525	-4.727880714
	H318	-2.304463953	-1.792752279	-6.066766004
	H319	7.823614537	-9.860235404	-0.694431345
	H320	7.104137486	-7.373636644	-2.614754088
	H321	6.510689507	-8.214907046	0.779576091
	H322	7.201829695	-10.452543539	-1.994760335
	H323	6.428285099	3.032760292	-8.560151189
	H324	6.208774829	-5.099094297	0.774368780
	H325	2.063581598	-7.980070326	0.892764117
	H326	4.603376180	-1.730704833	-6.956311598
	H327	3.208072662	-9.317978470	-0.644435729
	H328	4.301690279	-6.652164948	-6.599062417
	H329	3.103913970	-5.269380868	-4.853579699
	H330	5.764613321	-12.668455289	3.955734914
	H331	5.335019803	-1.201492572	2.927782328
	H332	8.069427887	-5.861736934	2.064513347
	H333	7.025798923	-3.803822165	1.029400866
	H334	8.312497845	-7.036095507	4.068434814
	H335	2.645282718	-2.957845250	5.549773503
	H336	4.397590546	-8.213337029	-3.245843026
	H337	5.358752435	-8.607630419	-1.814087846
	H338	2.292686547	-8.758746006	-2.052723433
	H339	2.425521607	-1.965009166	-5.875393376
	H340	-1.697997054	2.467674537	-6.355746868
	H341	-3.726095496	1.355172518	-7.388319450
	H342	2.106534926	-6.215008374	0.814359790
	H343	8.249266332	-7.383881434	1.800881634
	H344	7.210277209	-9.320776530	1.594077301
	H345	3.119711783	-1.301885238	5.694288024
	H346	6.677438752	-7.300512874	-5.859225241
	H347	7.046450011	-5.674883992	-2.177628255
	H348	1.807111264	-2.631468309	-7.415561977
	H349	5.674338101	4.249682408	-5.851216885
	H350	7.003500183	-0.046829353	5.172088307
	H351	7.368373400	0.521141304	3.526020732
	H352	9.911765630	-6.926869828	-4.965896745
	H353	8.924227697	-8.063589728	-4.322967907
	H354	10.488643031	-8.552278876	-0.161879829
	H355	9.020571489	-8.203854151	-1.867015476
	H356	12.228261790	-7.351329995	-1.899824746
	H357	10.155529076	-6.876174698	0.330728774
	H358	10.053822983	0.305925088	4.004396471
	H359	-3.629574649	12.983408053	3.818948341
	H360	9.085667917	-7.207912254	6.970719476
	H361	8.472414833	-4.749436778	5.911542400
	H362	-4.904462107	12.328751425	1.142877871
	H363	-4.704079631	10.166003206	3.334057047
	H364	-6.203182086	10.889738665	2.721865910
	H365	0.233668569	-4.169448580	-1.113239455
	H366	3.929727506	-1.238396619	1.163325453
	H367	-5.983947344	7.045772367	-2.472920311
	H368	0.637801989	-0.717061305	0.310512510
	H369	3.290209280	-1.233857607	0.664230166
	H370	0.304804729	-3.877221592	-0.422217164
	H371	-1.518136971	-0.028123739	-1.124021825
	Mo372	4.058430722	-4.390122972	-1.768871997
	N373	-6.702146447	-7.058527860	-1.902509651
	N374	-8.948366543	-4.671062747	-0.098927411
	N375	-8.887756733	-5.098549912	2.679512620
	N376	-6.255011753	-5.964149665	3.162869572
	N377	-4.825691423	-3.549037368	2.707424971
	N378	-5.899550695	-2.022195807	4.901024080
	N379	-5.228707925	-3.551632627	7.262581458
	N380	-2.556258103	-4.657484437	7.020244323
	N381	-0.976648685	-2.675011601	5.918377479
	N382	-1.652219330	0.181448217	6.628113806
	N383	-2.502805691	-0.225084619	9.365905635
	N384	4.425541601	-11.820714725	5.443413346
	N385	5.558100798	-9.525212990	4.648142029
	N386	4.479533167	-3.499351255	3.721595565
	N387	3.297402699	-2.214026685	5.283889152
	N388	4.738078196	-1.195832683	3.774465664
	N389	8.212352775	-7.224074691	5.090896019
	N390	-5.184176372	-2.687579971	-4.160863698
	N391	-1.361947364	-5.458184322	-3.743453841

	N392	-5.557756194	-0.227815248	-3.340401458
	N393	-8.004904234	0.862292088	-2.508298259
	N394	-7.993105286	0.369491966	0.255901974
	N395	-6.006051209	2.080228507	1.251049921
	N396	-2.920004735	2.750416884	-1.558884009
	N397	-2.150633173	0.792143059	-1.075086208
	N398	-6.742887514	4.684077120	1.900008368
	N399	-9.864405356	7.813276756	0.219338942
	N400	-9.993674322	9.521871260	1.628745663
	N401	8.916964778	2.621770092	4.258455454
	N402	6.253190629	7.216520039	1.139947866
	N403	4.629738388	8.613706439	-1.665277274
	N404	2.109781098	8.019338999	-2.631977513
	N405	-2.277153212	6.657489001	-4.694536097
	N406	-2.268082670	8.509980269	-6.124711106
	N407	-3.621678033	8.500011490	-4.245240487
	N408	0.799452501	6.346568999	-0.679909873
	N409	0.699906292	8.136147029	1.474158671
	N410	-0.509820322	10.582805796	1.076882978
	N411	-1.315725790	10.597735050	-2.844092387
	N412	-3.033146911	11.571050906	1.739405143
	N413	5.920536022	-4.396559009	-9.452918512
	N414	3.849172184	-2.272036201	-7.388933852
	N415	2.733006747	0.503145195	-6.829916829
	N416	4.884075082	2.402510337	-6.550992495
	N417	0.242658530	-2.551104744	-10.316787982
	N418	-1.599700942	-0.761112743	-9.045236857
	N419	8.951477549	-7.093895283	-4.652351089
	N420	4.929444878	-5.473558152	-3.714904540
	N421	4.729734751	-6.360586468	-5.724313843
	N422	9.644422349	-7.414712846	-1.669817802
	O423	-6.750023633	-5.149112746	0.248493398
	O424	-8.010619800	-3.010108999	2.961984866
	O425	-6.544359676	-5.509944388	5.385858372
	O426	-3.310866790	-4.300957543	4.228240174
	O427	-3.893253282	-0.954196480	4.939120975
	O428	-4.931189590	-1.526547799	8.283894938
	O429	-2.424661981	-4.567206229	9.309159824
	O430	0.941495446	-3.908971605	6.234597477
	O431	0.616277058	0.134819939	6.916148682
	O432	-1.070229012	1.470348623	9.843724205
	O433	-0.867678379	-0.828210971	12.497756946
	O434	6.617980696	-10.504923120	2.841725278
	O435	6.921836912	-8.130954261	6.764235362
	O436	10.463240091	-4.736898150	6.354481726
	O437	-5.526200931	-0.746476264	-1.105605023
	O438	-2.852769516	-4.147028975	-4.848543602
	O439	-6.873593394	2.639906449	-1.628990282
	O440	-5.574035586	3.317672023	-4.372126525
	O441	-9.987233647	1.482995388	0.483895001
	O442	-6.226970761	1.197855752	3.348207333
	O443	-4.843748636	4.945342836	3.139877400
	O444	-7.383696209	7.109636260	4.408965521
	O445	9.918224130	-0.299452640	5.948999338
	O446	2.678893792	4.327918334	4.643605080
	O447	3.636861361	8.383216996	0.375988721
	O448	1.577782917	10.246914662	-2.864041860
	O449	-1.137173049	7.256537475	-1.483723914
	O450	-1.179297195	7.062077265	2.236981890
	O451	2.032467642	5.585629483	2.097531489
	O452	0.349450335	11.416423506	3.032226391
	O453	-2.543946208	13.623163486	0.874210454
	O454	-2.977635002	11.183037502	-1.389536331
	O455	-5.516967569	13.603264315	3.343685688
	O456	-5.113640525	7.025136398	-2.029569061
	O457	3.336169289	-4.248395576	-8.444127104
	O458	1.344412765	-0.354454656	-8.441003973
	O459	3.710350285	3.888670609	-7.803606527
	O460	7.261853373	4.761488022	-7.867490460
	O461	-3.226274797	-2.188799662	-9.760229492
	O462	-0.363169443	-6.354835644	-6.455787459
	O463	1.657518013	-5.480201056	-6.807118971
	O464	-2.276068551	2.738541015	-8.963604031
	O465	10.381931993	-5.311324430	-2.080989125
	O466	12.760719585	-6.797166970	-0.008346322
	O467	0.510084885	-6.539001560	-1.615947522
	O468	-0.396147156	-7.433454675	0.233218875
	O469	5.718685829	-10.858742781	-2.807770041
	O470	3.510899176	-10.873513755	-3.297404150
	O471	5.145247388	-5.852925408	-0.495357261

	O472 4.877706885 -7.685482155 0.782523707<br>O473 2.930891510 -6.168015923 -1.823371562<br>O474 8.608141981 -6.636593415 2.357424319<br>O475 6.729495054 -4.621351536 1.474336209<br>O476 -3.839517005 4.943884652 -3.010334309<br>O477 8.081490506 -10.315443172 -1.526881737<br>O478 7.404167320 -8.639417220 0.902191796<br>S479 4.021920143 5.056551989 -1.567995720<br>S480 2.947817965 2.068171238 -3.190202852<br>S481 2.563440075 -3.316560959 -3.325152726<br>S482 3.666124936 1.849733812 0.409407077<br>S483 0.725814762 -0.804225982 -1.046007224<br>S484 4.995445264 -0.794548140 -4.699307121<br>S485 3.139401154 -3.648705431 0.290314684<br>S486 6.296691180 1.739190419 -2.014281280<br>S487 5.962475306 -2.977236607 -1.757497232<br>S488 6.274344004 -0.917992431 0.805778668<br>N489 1.101558137 2.042923032 -1.005849572<br>N490 0.236451777 2.739922959 -0.816768981<br>end

7H2n dissociation from S2BH-6H2x-7H2n 10 to 12

TS

Fe(139) -2.376<br>Fe(140) -1.338<br>Fe(141) -2.388<br>Fe(142) 2.626<br>Fe(143) 2.834<br>Fe(144) 0.405<br>Fe(145) 0.972<br>[<br>**S=1/2**	2n2xs2bh6h2x7h2ndtd_1_53174.54<br>C1 -7.803546466 -5.368810928 -0.369899880<br>C2 -7.939404035 -6.473622368 -1.422636337<br>C3 -8.930862799 -3.525288133 0.796573719<br>C4 -8.569236214 -3.858693972 2.241701943<br>C5 -8.525322712 -5.546565420 4.013430786<br>C6 -7.015312540 -5.664830555 4.245620300<br>C7 -4.803816906 -5.942464198 3.245643295<br>C8 -4.240896621 -4.532581718 3.437301908<br>C9 -4.466236291 -2.155272229 2.910222099<br>C10 -4.725289113 -1.657711160 4.338974539<br>C11 -6.223628715 -1.552821499 6.238107868<br>C12 -5.388532923 -2.206891183 7.347178132<br>C13 -4.610114974 -4.307096120 8.341631646<br>C14 -3.089815573 -4.506662800 8.266463116<br>C15 -1.147708625 -4.943921982 6.815985925<br>C16 -0.297015832 -3.783505797 6.297798236<br>C17 -0.320919201 -1.487975847 5.391995156<br>C18 -0.411461420 -0.315645792 6.381009955<br>C19 -0.748254728 -1.140121405 3.942190157<br>C20 -0.030668259 0.125012446 3.456175588<br>C21 -0.473249243 -2.317528914 2.999636426<br>C22 -1.837656119 1.307229103 7.540539940<br>C23 -1.770758670 0.862154264 9.014070172<br>C24 -3.122618016 2.095079142 7.192949762<br>C25 -3.376617887 3.204822907 8.218927707<br>C26 -3.020711787 2.679853286 5.778058618<br>C27 -2.523456756 -0.653568165 10.751484728<br>C28 -1.132113069 -0.888721906 11.305053081<br>C29 5.064949188 -11.824544644 4.117541244<br>C30 5.835787900 -10.544178300 3.811140474<br>C31 6.038052757 -8.161389266 4.500573703<br>C32 7.105055981 -7.847846353 5.566649883<br>C33 4.850686428 -7.188021828 4.605088614<br>C34 5.255689069 -5.738802957 4.346250723<br>C35 4.046847936 -4.817680370 4.195878439<br>C36 4.165721887 -2.315389498 4.262642030<br>C37 9.297224358 -6.816743009 5.957133520<br>C38 9.421701779 -5.313330666 6.077894258<br>C39 -4.939192624 -2.495139692 -2.726234020<br>C40 -5.374735738 -1.081003062 -2.297122030<br>C41 -3.448651610 -2.672123326 -2.357306030<br>C42 -2.905256221 -4.114328881 -2.414112116<br>C43 -2.369047216 -4.568267438 -3.772499001<br>C44 -5.665914616 1.209362763 -3.160355825<br>C45 -6.908559984 1.639063925 -2.369432558<br>C46 -5.574852986 1.883727927 -4.535623765<br>C47 -9.221675027 1.113796003 -1.754567135<br>C48 -9.096067201 1.003156995 -0.230894311<br>C49 -7.710692251 0.329965752 1.677104225<br>C50 -6.579478747 1.236246148 2.164277597<br>C51 -4.968259720 3.009614858 1.670318055<br>C52 -5.521786727 4.291085113 2.339390547<br>C53 -4.116461373 3.498819450 0.488928728<br>C54 -3.335077439 2.473016907 -0.264105965

	C55	-2.837113285	1.229394629	0.038777090
	C56	-2.190675290	1.725119394	-2.030570819
	C57	-7.317669384	5.976736566	2.254757647
	C58	-7.439846889	6.069516486	3.774261508
	C59	-8.706347827	6.075710826	1.579034751
	C60	-9.276826994	7.455078863	1.420893902
	C61	-9.351171262	8.509502377	2.310048197
	C62	-10.288542411	9.060994482	0.379462302
	C63	8.515844994	1.486382365	5.094711227
	C64	9.588139537	0.414629795	5.015501634
	C65	7.206640513	0.848675671	4.567069021
	C66	6.022212027	1.784173828	4.621023240
	C67	5.219403486	1.883600777	5.771838607
	C68	5.676939959	2.570334149	3.509312873
	C69	4.100273438	2.722835188	5.807530563
	C70	4.572793287	3.423283908	3.534667349
	C71	3.778660427	3.490397823	4.682073600
	C72	5.535589482	6.934383790	-0.110699157
	C73	4.511378344	8.034940929	-0.434186632
	C74	4.841100644	5.578698832	0.010521587
	C75	3.785620101	9.715134518	-2.095956613
	C76	2.379311389	9.340852417	-2.568895211
	C77	0.798782977	7.445290915	-2.922602814
	C78	0.067437776	7.003584958	-1.629698559
	C79	1.027021891	6.264735224	-3.897425305
	C80	-0.161011037	5.380328808	-4.296320129
	C81	-1.149752699	5.960550523	-5.318915416
	C82	-2.736553327	7.862816768	-5.042909945
	C83	0.147694239	5.928973319	0.562896790
	C84	-0.194782546	7.106281887	1.487921985
	C85	0.982384718	4.926355626	1.384004379
	C86	0.741455288	9.094439405	2.562676928
	C87	0.183733266	10.473828484	2.248306583
	C88	-0.875500072	11.901038703	0.574803929
	C89	-2.242781424	12.440222655	1.060724642
	C90	-0.747668048	12.013836929	-0.947670544
	C91	-1.786644685	11.213885200	-1.725832167
	C92	-4.427544908	11.896570584	2.034686776
	C93	-4.525689073	12.921876096	3.154455993
	C94	-5.183143485	10.598060328	2.439566695
	C95	-5.215837286	9.585319782	1.316242955
	C96	-6.219227644	9.651211328	0.336695284
	C97	-4.220747862	8.604291341	1.176110218
	C98	-6.216782264	8.791042492	-0.763249388
	C99	-4.188241172	7.749845995	0.069101498
	C100	-5.186968729	7.858419219	-0.893782486
	C101	5.663093760	-3.639855380	-8.223392493
	C102	4.173747724	-3.408569991	-8.041491639
	C103	2.497448166	-1.913869600	-6.976501318
	C104	2.124990703	-0.516822418	-7.481630532
	C105	2.539968353	1.889730649	-7.210022163
	C106	3.769685758	2.792184361	-7.219777303
	C107	5.983898061	3.345540290	-6.401597534
	C108	6.592635801	3.751367566	-7.727629023
	C109	-0.966647723	-3.066140143	-9.662882021
	C110	-2.029037193	-1.972905391	-9.503678624
	C111	-0.774528505	-3.807825161	-8.318971222
	C112	-0.039544610	-5.146703886	-8.522029436
	C113	0.390612048	-5.728386467	-7.193211679
	C114	-2.539870857	0.326663228	-8.850868349
	C115	-1.953763585	1.627644854	-9.363815992
	C116	-3.158077712	0.412719762	-7.432715893
	C117	-2.119017204	0.347597706	-6.342751966
	C118	-1.420668818	1.497385240	-5.934747888
	C119	-1.755966288	-0.892771636	-5.787427861
	C120	-0.357134669	1.397594435	-5.031542118
	C121	-0.691875427	-0.992470547	-4.885553694
	C122	0.027004929	0.150721363	-4.523448910
	C123	8.632906177	-6.167297658	-3.553542736
	C124	9.616597989	-6.252964125	-2.377082529
	C125	7.201282944	-6.353719001	-3.025523943
	C126	6.104378772	-6.149322079	-4.019625413
	C127	5.986426288	-6.696822089	-5.280600287
	C128	4.111010638	-5.641181434	-4.766576475
	C129	10.500183006	-7.503142928	-0.504750037
	C130	11.937802701	-7.167906348	-0.836752280
	C131	0.592734605	-7.032419616	-0.362415896
	C132	2.000935310	-7.131118629	0.211835928
	C133	3.149810962	-7.206071584	-0.836854492
	C134	3.208206586	-8.617643185	-1.472074738

	C135 4.432820156 -8.873761373 -2.354147490<br>C136 4.556304532 -10.329083644 -2.856183654<br>C137 4.489917880 -6.931847129 -0.131245061<br>C138 3.939917987 -0.573373279 -1.554138203<br>Fe139 4.225897626 -1.967205511 -2.908336793<br>Fe140 2.489379172 0.877548497 -1.287036022<br>Fe141 5.208331540 0.480987355 -0.518299295<br>Fe142 4.617935290 0.541320706 -3.029209338<br>Fe143 4.331281988 2.783833224 -1.491028619<br>Fe144 4.529931770 -1.981621127 -0.315444604<br>Fe145 2.110185413 -2.758246520 -1.192471017<br>H146 -6.142375592 -7.386887568 -1.111513667<br>H147 -6.133393401 -6.349556761 -2.372099599<br>H148 -9.761176943 -4.816136711 -0.688670436<br>H149 -8.520390954 -6.086453743 -2.275042255<br>H150 -8.561474405 -7.264667741 -0.969002231<br>H151 -8.208597578 -2.767768077 0.459010490<br>H152 -9.343190099 -5.742502327 2.034201600<br>H153 -9.928913861 -3.067302092 0.788010709<br>H154 -8.985612913 -6.528480273 4.186358845<br>H155 -6.681245824 -5.965789963 2.233617490<br>H156 -8.908006242 -4.851256651 4.771771989<br>H157 -3.401000908 -1.998337884 2.701828508<br>H158 -5.576610705 -3.783403605 2.058654342<br>H159 -4.393303519 -6.380647667 2.325400729<br>H160 -6.058836983 -0.469757380 6.309576363<br>H161 -6.614617054 -2.489784542 4.335206245<br>H162 -4.461211598 -6.544941143 4.095935368<br>H163 -5.048964777 -1.547789122 2.204861787<br>H164 -5.699401017 -4.071511805 6.514355616<br>H165 -7.285408749 -1.761092511 6.428532149<br>H166 -0.966472185 -3.239844104 3.337334883<br>H167 -0.848153377 -2.095906509 1.987484639<br>H168 0.607629752 -2.514011879 2.914973703<br>H169 -1.832098377 -0.943879994 3.955497017<br>H170 -5.074647380 -5.303408648 8.373835149<br>H171 -2.896327723 1.899434876 5.014651922<br>H172 -3.175468723 -0.654751996 8.723827459<br>H173 -3.537189860 2.807309785 9.231413372<br>H174 1.060574823 -0.024710543 3.424062703<br>H175 -0.234680287 0.996643635 4.094890260<br>H176 8.367552072 1.734787755 6.163261142<br>H177 -3.927843683 3.247575986 5.527304245<br>H178 -4.267834382 3.782171120 7.933493630<br>H179 -2.161854964 3.368023212 5.710739722<br>H180 -2.520839838 3.897361397 8.258897161<br>H181 -3.973892630 1.390789110 7.223526284<br>H182 -0.974019295 1.978119300 7.418518879<br>H183 -2.460251878 -0.183510961 6.114095031<br>H184 -0.358369715 0.380427682 2.436550875<br>H185 1.399577807 4.165347232 0.709285582<br>H186 0.286282543 4.432568727 2.079845376<br>H187 2.147065969 5.130226302 2.955944371<br>H188 2.191685346 4.273446004 5.486052233<br>H189 4.305473597 4.011573403 2.656930595<br>H190 6.261577349 2.490575876 2.590632158<br>H191 5.459705852 1.282896604 6.653158992<br>H192 -1.994287433 -2.710729491 5.939907612<br>H193 -1.033530547 -5.773215600 6.101562693<br>H194 -0.714481833 -5.263230179 7.772949840<br>H195 -3.159609721 -4.665623848 6.191704514<br>H196 -4.806450611 -3.803445550 9.295899349<br>H197 3.473828245 2.768281181 6.702711181<br>H198 0.745888706 -1.736889647 5.370716872<br>H199 -3.050293028 0.060823719 11.405059927<br>H200 -3.055240829 -1.617456117 10.796864562<br>H201 -0.367664345 -1.189865575 10.550492034<br>H202 10.258142119 -7.240223058 5.627117718<br>H203 -0.987544146 -5.812581371 -4.631252242<br>H204 1.504762750 -6.206285691 -1.844113301<br>H205 -0.715916161 -5.864752215 -9.007114459<br>H206 0.835461680 -5.001794770 -9.172284275<br>H207 -1.751904616 -4.002492676 -7.850922987<br>H208 -1.218825592 1.503413633 -10.193243902<br>H209 -1.431254054 -3.776295677 -10.363646494<br>H210 -2.845733747 -2.021280797 -3.011517953<br>H211 -3.694403273 -4.827739749 -2.119614922<br>H212 -0.614481381 -0.624629109 -8.792935584<br>H213 -3.373127650 0.114381104 -9.551368174<br>H214 0.814703553 -1.992484574 -9.670666016

	H215	-0.917088726	-5.774265392	-2.875508856
	H216	-0.220215691	-3.157991578	-7.624387669
	H217	0.823984811	-3.330370228	-10.632637635
	H218	-6.062562478	-3.181849271	-4.316933895
	H219	-3.325916160	-2.307953449	-1.326838939
	H220	2.179597975	-4.974163664	-7.504465131
	H221	-2.111274023	-4.227727353	-1.661074858
	H222	-4.344056602	2.518209512	2.429638862
	H223	-2.927739922	0.626067265	0.933201412
	H224	-8.621107803	0.644190582	2.206885745
	H225	-7.287011139	-0.012192702	-0.383787113
	H226	-7.261220107	4.072450548	1.272249007
	H227	-8.993702976	8.635387227	3.325927929
	H228	-7.632951469	5.087756537	4.270584169
	H229	-9.414105457	5.415047068	2.110062236
	H230	-6.667644961	6.794937529	1.905932140
	H231	-10.799288584	9.667952618	-0.364479141
	H232	-3.416729648	4.248855502	0.892944890
	H233	-4.769017588	4.024323331	-0.225939796
	H234	-10.203687998	10.445758393	1.994621517
	H235	-8.604451198	5.666298228	0.561948425
	H236	-6.331193542	2.089518706	0.278717329
	H237	-7.489177181	-0.695078520	2.008016996
	H238	-9.593463899	2.127916206	-1.956246629
	H239	-1.711197292	1.660850361	-3.004263280
	H240	-9.989517192	0.404778833	-2.089670919
	H241	9.650174642	3.154003064	4.736242943
	H242	8.125781005	3.261950633	4.154686288
	H243	1.749490114	6.002718986	-0.888702741
	H244	-0.808145805	5.448802363	0.308733998
	H245	1.574793542	7.995982655	0.962312429
	H246	-3.167623857	3.620750400	-2.091014315
	H247	-4.505975326	4.403098931	-3.517823798
	H248	-5.735879077	3.699190587	-5.254076563
	H249	-4.355012173	5.588693343	-2.465013927
	H250	-4.641978595	1.549837840	-5.019826447
	H251	-6.429212739	1.578677178	-5.159766536
	H252	0.276489524	11.751773976	-1.249373792
	H253	-0.905132259	13.072420183	-1.208311493
	H254	-0.307031619	10.443996709	-2.965134470
	H255	-1.970987559	9.967863302	-3.304655699
	H256	-2.578238810	9.457884982	-6.323096043
	H257	-1.551075298	5.149607352	-5.946451738
	H258	-7.013378111	8.846660346	-1.509188862
	H259	-3.445800859	8.482162267	1.937250160
	H260	-3.974001541	8.032596806	-3.406896866
	H261	1.782177528	5.606334025	-3.437388669
	H262	2.865119108	7.368827348	-2.400797627
	H263	-0.706719989	5.024878261	-3.407899821
	H264	-7.933550932	0.023259383	-3.082557385
	H265	4.284720949	10.251216758	-2.913488624
	H266	-5.388131703	-0.688830705	-4.251371772
	H267	1.493321077	6.678178351	-4.806835283
	H268	-3.368882811	7.039913223	-0.053358540
	H269	3.648053811	10.423572742	-1.265590959
	H270	-1.810339120	8.009104661	-6.878047567
	H271	-4.242697945	9.192653515	-4.663717160
	H272	-4.412452934	-3.252639822	-4.565433847
	H273	-5.523886474	-3.188226991	-2.092606404
	H274	-7.021852039	10.386209332	0.432511146
	H275	-4.827648400	1.561023057	-2.543531801
	H276	0.172326522	8.221938176	-3.373513524
	H277	-0.617227215	6.651324565	-5.984924562
	H278	0.275841534	4.484928305	-4.761880589
	H279	-2.820228590	6.114756907	-3.999358917
	H280	0.171723559	8.697680064	3.416244573
	H281	-0.500808540	9.780508315	0.452033527
	H282	-0.153351559	12.606737479	1.013898367
	H283	1.783190636	9.234629683	2.883024133
	H284	-2.761082123	10.594984437	1.718839323
	H285	6.187859012	-2.673835536	-8.267725255
	H286	5.999600121	-4.143696876	-7.293288117
	H287	8.739402492	-5.144735437	-3.944828172
	H288	6.884129152	-4.736159226	-9.432966516
	H289	5.322639891	-5.228822129	-9.450271320
	H290	5.600727960	4.837407955	0.287833549
	H291	5.564743419	7.405078412	1.878200334
	H292	5.364574038	8.291688459	-2.288108549
	H293	4.074866946	5.619543917	0.798061538
	H294	6.790514424	8.087088670	1.036908887

	H295	6.771747504	2.852466951	-5.811344790
	H296	6.279965630	6.878305940	-0.920281284
	H297	4.385910774	-7.295399746	5.599794993
	H298	6.479194332	-8.080565640	3.498888693
	H299	4.898429024	1.555750469	-5.981060219
	H300	4.384656776	-0.292318118	4.068395995
	H301	2.110717527	1.900649796	-8.219021493
	H302	-0.406218239	-1.966061227	-4.481152354
	H303	1.804862097	2.374215451	-6.545661471
	H304	3.522168779	-4.716462098	5.153650612
	H305	3.245116059	0.261530652	-5.965790256
	H306	3.333645690	-5.234126091	3.466381032
	H307	4.110037564	-7.506601658	3.854018422
	H308	5.847867278	-5.685983440	3.421790934
	H309	5.891808976	-5.359074658	5.161173879
	H310	-3.867609345	-0.421112107	-7.329155676
	H311	4.909927332	-9.764994681	5.404194709
	H312	4.287670918	-11.899914225	3.341970387
	H313	0.883781389	0.088476470	-3.850655978
	H314	5.062399332	-12.198021799	6.142318615
	H315	3.603201289	-12.426051131	5.435671607
	H316	5.116650221	-3.507106187	2.924909373
	H317	0.202831780	2.285367381	-4.728800336
	H318	-2.305769482	-1.793734010	-6.067608212
	H319	7.820650772	-9.857658868	-0.694986697
	H320	7.100487837	-7.372187680	-2.613091475
	H321	6.507471723	-8.210310936	0.778703183
	H322	7.200869326	-10.451161028	-1.996022475
	H323	6.428686132	3.032541439	-8.564737634
	H324	6.216122927	-5.106797938	0.757056750
	H325	2.043285652	-7.988044768	0.895311210
	H326	4.599792469	-1.736198403	-6.952000674
	H327	3.204723174	-9.339809521	-0.641056012
	H328	4.303989740	-6.651829685	-6.604680762
	H329	3.096534410	-5.276301678	-4.857028484
	H330	5.760927409	-12.664036094	3.950716456
	H331	5.315964722	-1.206721641	2.923360697
	H332	8.066830151	-5.860135711	2.061083243
	H333	7.004187528	-3.798602626	1.026771219
	H334	8.310340599	-7.035555692	4.066671889
	H335	2.642545646	-2.960583514	5.552538630
	H336	4.388126335	-8.219373521	-3.238988237
	H337	5.352086853	-8.615994287	-1.810689559
	H338	2.285397941	-8.784310991	-2.049438492
	H339	2.425271583	-1.966356149	-5.878225270
	H340	-1.698892747	2.466610794	-6.356106654
	H341	-3.726732324	1.354050003	-7.389172951
	H342	2.134007659	-6.223372236	0.825170826
	H343	8.247300717	-7.382157199	1.799151226
	H344	7.207739191	-9.317145066	1.591173462
	H345	3.116683233	-1.304970354	5.701314391
	H346	6.679155123	-7.296433232	-5.857093357
	H347	7.045959365	-5.674418396	-2.177919583
	H348	1.803945886	-2.632046657	-7.418245893
	H349	5.674415961	4.251083100	-5.856382565
	H350	7.003453419	-0.047289630	5.171802774
	H351	7.370584793	0.521802512	3.527608123
	H352	9.909322233	-6.928132583	-4.966229498
	H353	8.922751519	-8.064958637	-4.322727703
	H354	10.485355582	-8.550114982	-0.162055858
	H355	9.017911290	-8.204209981	-1.867284585
	H356	12.226296824	-7.352162231	-1.899864629
	H357	10.152526989	-6.873950454	0.329765563
	H358	10.051813442	0.305124304	4.004928064
	H359	-3.631752756	12.983464143	3.819248328
	H360	9.084788455	-7.207526641	6.968051867
	H361	8.471055869	-4.748782418	5.910388540
	H362	-4.908654144	12.331230747	1.142783826
	H363	-4.704884058	10.167140010	3.333136829
	H364	-6.205346778	10.888706787	2.721794588
	H365	0.810766269	-3.757973357	-1.311947587
	H366	3.676216216	-1.150598524	1.258826260
	H367	-5.985242481	7.045545421	-2.473180926
	H368	0.608074938	-0.720302735	0.259314152
	H369	3.043401982	-1.198744526	0.811387251
	H370	0.874794818	-3.574904227	-0.492292027
	H371	-1.517010338	-0.030925471	-1.125084246
	Mo372	4.048627102	-4.431544465	-1.780366687
	N373	-6.701241436	-7.059909040	-1.903893956
	N374	-8.944249901	-4.670492133	-0.100586114

	N375	-8.888204725	-5.099803891	2.678492745
	N376	-6.255995916	-5.965465562	3.162168229
	N377	-4.826196848	-3.550493342	2.706337289
	N378	-5.900345138	-2.023525543	4.900394201
	N379	-5.229307912	-3.552972875	7.261890791
	N380	-2.557032999	-4.658254682	7.019638585
	N381	-0.976803253	-2.675765723	5.915917750
	N382	-1.652731408	0.180415654	6.626636894
	N383	-2.502989579	-0.226771216	9.364305948
	N384	4.423804407	-11.818200678	5.439899849
	N385	5.557757477	-9.524176928	4.646984629
	N386	4.480790396	-3.499095944	3.728182423
	N387	3.295335052	-2.216397779	5.289820873
	N388	4.752590985	-1.198774822	3.794433074
	N389	8.210890401	-7.223663719	5.089089304
	N390	-5.181599360	-2.688174564	-4.161059889
	N391	-1.360844108	-5.460184180	-3.742662905
	N392	-5.557359078	-0.229090537	-3.340694286
	N393	-8.005556048	0.860829841	-2.508922625
	N394	-7.993884483	0.368122425	0.255188381
	N395	-6.007058309	2.079035697	1.250292726
	N396	-2.919394509	2.746107860	-1.560130447
	N397	-2.144261165	0.790364524	-1.074229324
	N398	-6.743950344	4.683533542	1.899215561
	N399	-9.865748882	7.812148432	0.218820936
	N400	-9.994468207	9.520897688	1.628072111
	N401	8.917653865	2.621491217	4.260623427
	N402	6.254297186	7.221021893	1.142194125
	N403	4.629432070	8.616050420	-1.665367598
	N404	2.107743213	8.021115194	-2.633422130
	N405	-2.280418065	6.658735467	-4.696504021
	N406	-2.272151301	8.510296082	-6.127584464
	N407	-3.624705377	8.501109488	-4.246692703
	N408	0.798885308	6.347244491	-0.682361757
	N409	0.698415265	8.136184057	1.472313479
	N410	-0.512757005	10.586315448	1.077079345
	N411	-1.316081729	10.597626715	-2.843390850
	N412	-3.036789089	11.573970225	1.738680268
	N413	5.919891322	-4.396908906	-9.450781833
	N414	3.846469345	-2.272644690	-7.392617833
	N415	2.733223700	0.501189072	-6.829217047
	N416	4.885618487	2.402133468	-6.553726309
	N417	0.243558251	-2.551492937	-10.314804128
	N418	-1.598900098	-0.761734113	-9.043989095
	N419	8.948854635	-7.095647421	-4.653380278
	N420	4.919587447	-5.479176803	-3.716887228
	N421	4.728308887	-6.361616516	-5.727791744
	N422	9.641640930	-7.414521845	-1.671069918
	O423	-6.744876512	-5.146375544	0.245714716
	O424	-8.011057530	-3.011438964	2.961646432
	O425	-6.545298967	-5.511357338	5.385168785
	O426	-3.310902129	-4.302973602	4.226535425
	O427	-3.893776061	-0.956007061	4.938427874
	O428	-4.931998644	-1.527908176	8.283307507
	O429	-2.425354769	-4.568673011	9.308595784
	O430	0.940646186	-3.910507317	6.233020107
	O431	0.615732930	0.133195103	6.914846159
	O432	-1.071156237	1.469109015	9.842671879
	O433	-0.869008498	-0.829568224	12.496511201
	O434	6.617374124	-10.501893193	2.839293657
	O435	6.921539565	-8.131427990	6.763111108
	O436	10.461973344	-4.736927406	6.353765750
	O437	-5.524906755	-0.747968017	-1.105841188
	O438	-2.854815011	-4.151982414	-4.848664214
	O439	-6.874198661	2.638377071	-1.629595263
	O440	-5.575600430	3.316047022	-4.372968427
	O441	-9.987991901	1.481645150	0.483139310
	O442	-6.227359230	1.196288174	3.347354295
	O443	-4.844194634	4.944152866	3.138668099
	O444	-7.384762075	7.108347002	4.408745222
	O445	9.919081423	-0.301285217	5.949372979
	O446	2.678323595	4.326431481	4.643044523
	O447	3.636349969	8.386782340	0.376131511
	O448	1.577181966	10.248571780	-2.864668119
	O449	-1.139115705	7.256872371	-1.483457442
	O450	-1.179614708	7.060363542	2.235702246
	O451	2.031198042	5.585517195	2.096960115
	O452	0.348505140	11.416365263	3.032714371
	O453	-2.547962863	13.626481292	0.874252719
	O454	-2.978903856	11.182209343	-1.389739564

O455	-5.519914239	13.604072784	3.346385342
O456	-5.114986819	7.024881260	-2.029720339
O457	3.335926200	-4.251684656	-8.444576164
O458	1.343551909	-0.353590974	-8.441379694
O459	3.709260884	3.886932639	-7.804717429
O460	7.262941061	4.761680696	-7.873196766
O461	-3.224826041	-2.190149977	-9.759614517
O462	-0.363416263	-6.356220456	-6.456093286
O463	1.657275233	-5.481362872	-6.807922988
O464	-2.276929522	2.737741744	-8.964430271
O465	10.378834624	-5.311372564	-2.083509160
O466	12.758121562	-6.797658268	-0.008481495
O467	0.496633952	-6.412418184	-1.549617925
O468	-0.401079532	-7.432483934	0.238826046
O469	5.719855791	-10.863586408	-2.810986856
O470	3.512477607	-10.882154170	-3.303189479
O471	5.176197264	-5.913259397	-0.561756040
O472	4.869364426	-7.687291271	0.786964230
O473	2.920089358	-6.198616130	-1.828030592
O474	8.605349776	-6.634352011	2.355642232
O475	6.725313914	-4.628023828	1.461533940
O476	-3.841734546	4.944025840	-3.012455998
O477	8.080054226	-10.309646909	-1.528686467
O478	7.401106247	-8.635042243	0.899791167
S479	4.025263953	5.060689246	-1.571870511
S480	2.951074520	2.057050300	-3.181389633
S481	2.508694323	-3.359140268	-3.276357023
S482	3.682772071	1.861888070	0.419060786
S483	0.833247651	-0.831863580	-1.079658047
S484	4.954671417	-0.850195184	-4.680888961
S485	3.308133427	-3.741740551	0.361165110
S486	6.291042878	1.702851178	-2.011548361
S487	5.910683549	-2.964283014	-1.777788627
S488	6.189113669	-0.968781077	0.819606742
N489	1.100388931	2.046403175	-0.989559129
N490	0.229017224	2.740516763	-0.808490792
end			

S2BH-6Hx-7Hn-6H2n (8) to 6Hx-6H2n-7H2n (13)

reactant (8)

Fe(139)	-2.393
Fe(140)	-1.058
Fe(141)	-2.577
Fe(142)	2.691
Fe(143)	2.761
Fe(144)	1.410
Fe(145)	0.110

S=1/2

hd7hns2bhtfa.car_3

C1	-7.682747063	-5.447960212	2.144441182
C2	-8.265812577	-6.562157168	1.269403883
C3	-8.176259782	-3.462508617	3.496012136
C4	-7.307830458	-3.727765883	4.722031953
C5	-6.660713763	-5.276175134	6.496799146
C6	-5.157370434	-5.341256919	6.216404794
C7	-3.369504607	-5.691182896	4.596888738
C8	-2.803983618	-4.274295361	4.472894459
C9	-3.261874454	-1.953678157	3.842206832
C10	-3.011469497	-1.318843614	5.218035321
C11	-3.744381940	-1.068029243	7.518450418
C12	-2.601658564	-1.687824734	8.334127421
C13	-1.627228742	-3.742624739	9.240172019
C14	-0.234141235	-4.052997341	8.675750367
C15	1.055368330	-4.749519208	6.696922007
C16	1.766175418	-3.668068440	5.879279627
C17	1.531619387	-1.615212830	4.520082883
C18	1.942068171	-0.430275826	5.423911367
C19	0.550932452	-1.189575814	3.406438852
C20	1.069686204	0.030256613	2.635688411
C21	0.276817630	-2.371937577	2.471677069
C22	1.187350019	1.294517089	7.024539219
C23	1.696244995	0.906435037	8.428595643
C24	-0.068704113	2.199549139	7.073864539
C25	0.115301196	3.344460931	8.077718829
C26	-0.388133105	2.754182490	5.679488482
C27	1.337780326	-0.313595065	10.499287087
C28	2.801010497	-0.673915407	10.649424299
C29	6.338345212	-11.687391690	2.963782189
C30	6.812933159	-10.383743745	2.318146096
C31	7.070331664	-7.938132412	2.747067223
C32	8.377146360	-7.583545615	3.483515541
C33	5.942486260	-6.952239978	3.102686697
C34	6.272731782	-5.502687680	2.739475154
C35	5.048666437	-4.594163411	2.870309619
C36	5.137463133	-2.072272380	2.973639198
C37	10.655841176	-6.717640222	3.238954403

	C38	10.928221121	-5.230659724	3.208170981
	C39	-5.603738338	-2.676627937	-0.925828700
	C40	-5.904442316	-1.255665333	-0.409762522
	C41	-4.068365969	-2.819781931	-1.047713813
	C42	-3.534135997	-4.251309499	-1.262446585
	C43	-3.429172009	-4.702632803	-2.719258416
	C44	-6.456334414	1.016515454	-1.204541637
	C45	-7.436859061	1.460642639	-0.110616963
	C46	-6.762398381	1.654866981	-2.567596344
	C47	-9.451295745	0.914105905	1.170943512
	C48	-8.872197240	0.915620727	2.590240967
	C49	-6.965873502	0.371981310	4.037310416
	C50	-5.768592663	1.317785252	4.118933356
	C51	-4.387471711	3.036291655	3.062799947
	C52	-4.766288092	4.323363077	3.835183009
	C53	-3.952589544	3.495813994	1.663471976
	C54	-3.436993145	2.446124331	0.735084674
	C55	-2.969591430	1.167019315	0.904220163
	C56	-2.847617024	1.649411955	-1.268386635
	C57	-6.567691040	5.962141782	4.220083598
	C58	-6.318504537	6.069085586	5.723971839
	C59	-8.082857190	6.030011853	3.914819616
	C60	-8.691234559	7.401718587	3.880081018
	C61	-8.491073660	8.498938020	4.695888324
	C62	-9.998673772	8.957870004	3.137997240
	C63	9.523047443	1.522783915	2.521930003
	C64	10.528694572	0.425920929	2.224529161
	C65	8.114160171	0.881219728	2.453485969
	C66	7.013236318	1.857448349	2.795162315
	C67	6.606081465	2.063566971	4.126984846
	C68	6.365069889	2.593289237	1.787957621
	C69	5.579286253	2.960558727	4.443187815
	C70	5.350097954	3.502689712	2.089588745
	C71	4.951250142	3.676796909	3.418304476
	C72	5.135957312	6.853555168	-1.789977502
	C73	4.068410830	7.958338203	-1.873246425
	C74	4.504382359	5.537878722	-1.341728228
	C75	2.898874254	9.568945371	-3.330948398
	C76	1.414941265	9.196703061	-3.312535416
	C77	-0.198691993	7.310190961	-3.133958768
	C78	-0.513115392	6.955048657	-1.658967085
	C79	-0.275760634	6.071662810	-4.055996710
	C80	-1.541680430	5.207768465	-4.036906301
	C81	-2.770097883	5.765495847	-4.775975122
	C82	-4.224202896	7.677496200	-4.088526843
	C83	0.225691302	5.991509348	0.463376045
	C84	0.185666545	7.210736091	1.396173412
	C85	1.266788133	5.005016663	1.027719429
	C86	1.381258570	9.252016835	2.023458549
	C87	0.704310177	10.598383580	1.819224302
	C88	-0.891308780	11.878879930	0.496855564
	C89	-2.081148617	12.431686069	1.324036541
	C90	-1.225249807	11.908823321	-0.996728235
	C91	-2.415854738	11.040798934	-1.380115529
	C92	-3.837653505	11.940852337	2.971393240
	C93	-3.632684901	13.010630137	4.035060170
	C94	-4.420817972	10.650694560	3.615541668
	C95	-4.783535941	9.599180084	2.589719356
	C96	-6.055646915	9.591489282	1.994143743
	C97	-3.851717460	8.637384771	2.164553674
	C98	-6.379262257	8.673938647	0.990682590
	C99	-4.147194463	7.728000145	1.143610841
	C100	-5.412590838	7.763502305	0.562253054
	C101	3.010304196	-3.745805952	-9.329637739
	C102	1.636841293	-3.528611438	-8.721585505
	C103	0.313218289	-2.051586392	-7.225822841
	C104	-0.226516704	-0.684828289	-7.662775624
	C105	0.199648308	1.731446714	-7.638563968
	C106	1.353648624	2.693471615	-7.903603173
	C107	3.682236466	3.328026153	-7.607190019
	C108	4.146042612	3.517117447	-9.038416669
	C109	-3.820100214	-3.302110527	-8.749843277
	C110	-4.803826154	-2.230505715	-8.267420275
	C111	-3.192791270	-4.022561711	-7.533728859
	C112	-2.523220938	-5.343955404	-7.955012670
	C113	-1.702641699	-5.904612538	-6.816201767
	C114	-5.133339857	0.077154267	-7.527646158
	C115	-4.729424466	1.372968792	-8.203274680
	C116	-5.321748006	0.180140858	-5.993477738
	C117	-3.996616578	0.157839766	-5.286009904

	C118	-3.235644961	1.331458365	-5.149113079
	C119	-3.430395957	-1.069247648	-4.896035628
	C120	-1.909929221	1.264595618	-4.710090942
	C121	-2.105055153	-1.134348855	-4.460092120
	C122	-1.332045606	0.028698577	-4.404264898
	C123	7.238475474	-6.114309701	-5.732223868
	C124	8.528243822	-6.223974876	-4.903935667
	C125	6.019713733	-6.506857317	-4.886900366
	C126	4.676117329	-6.259859437	-5.495955594
	C127	4.173654736	-6.788266414	-6.666698344
	C128	2.558169211	-5.713458969	-5.600132121
	C129	9.919853515	-7.486920147	-3.374500305
	C130	11.182310730	-7.222218991	-4.164840263
	C131	0.478628384	-7.019695100	-0.372667647
	C132	1.997446201	-7.085203147	-0.244966408
	C133	2.760170786	-7.166332283	-1.599435966
	C134	2.604603684	-8.583892542	-2.210938386
	C135	3.510778916	-8.886488274	-3.407379019
	C136	3.503540168	-10.369669967	-3.839165771
	C137	4.258589325	-6.926711153	-1.355234581
	C138	3.208215094	-0.686749947	-2.440907249
	Fe139	3.315504869	-1.955643284	-3.958095952
	Fe140	1.817491708	0.708539387	-1.694160658
	Fe141	4.755913806	0.419381334	-1.836549621
	Fe142	3.322442552	0.556032373	-3.974234373
	Fe143	3.492134328	2.685575546	-2.386414202
	Fe144	4.423873232	-2.077762220	-1.674658447
	Fe145	1.867294521	-2.478028480	-1.489447603
	H146	-6.535567390	-7.585943341	0.916783960
	H147	-6.938659311	-6.558686062	-0.284187044
	H148	-9.580450729	-4.753381625	2.539327249
	H149	-9.101081749	-6.150861522	0.680102732
	H150	-8.714599001	-7.307250569	1.948165590
	H151	-7.594171301	-2.795201006	2.843740627
	H152	-8.141832295	-5.579835409	4.966078418
	H153	-9.074287768	-2.920622134	3.822151367
	H154	-6.994594655	-6.256117081	6.862090894
	H155	-5.478629544	-5.856712089	4.239647912
	H156	-6.796377935	-4.547847622	7.308055197
	H157	-2.342799988	-1.827125259	3.258984043
	H158	-4.571351952	-3.633921428	3.652815913
	H159	-3.248572738	-6.210422918	3.636383947
	H160	-3.545011244	0.007998688	7.442799877
	H161	-4.729382862	-2.181459746	5.958803567
	H162	-2.768204253	-6.215841248	5.349636396
	H163	-4.074644079	-1.422111732	3.327974133
	H164	-3.268061543	-3.591078271	7.872646293
	H165	-4.687590388	-1.214986893	8.061933307
	H166	-0.201690886	-3.212362473	2.991523992
	H167	-0.386667527	-2.069473830	1.646423518
	H168	1.213020454	-2.745080827	2.029220814
	H169	-0.391926975	-0.897038104	3.898002167
	H170	-2.087137833	-4.696761540	9.536756635
	H171	-0.571654805	1.954160515	4.948678632
	H172	0.072981582	-0.354492931	8.789012511
	H173	0.268029537	2.981216256	9.104119324
	H174	1.958683617	-0.211827881	2.033804367
	H175	1.335023449	0.867256642	3.298569609
	H176	9.694047792	1.854586976	3.564032364
	H177	-1.286747238	3.387792695	5.718498240
	H178	-0.776234190	3.988551771	8.075572812
	H179	0.447035552	3.373714357	5.312875187
	H180	0.983197330	3.965773122	7.804530120
	H181	-0.919587444	1.579006421	7.408796782
	H182	2.018155200	1.859673397	6.579196394
	H183	-0.003823133	-0.212523117	6.063833345
	H184	0.291188999	0.400141288	1.952673575
	H185	1.469131435	4.229474481	0.274518921
	H186	0.809118388	4.528193280	1.908122581
	H187	2.848881900	5.241083546	2.179472675
	H188	3.725859953	4.596299497	4.612671482
	H189	4.847340625	4.056361956	1.297025544
	H190	6.637646710	2.432481399	0.741808230
	H191	7.085685867	1.500802208	4.931957978
	H192	-0.033297288	-2.817378320	5.373148807
	H193	0.857475251	-5.597190700	6.022795250
	H194	1.763066549	-5.081931814	7.465247927
	H195	-1.027697088	-4.326078867	6.780977847
	H196	-1.467448544	-3.142710841	10.144343899
	H197	5.258971911	3.087698356	5.481015691

	H198	2.458146692	-1.974463513	4.059644455
	H199	1.102961499	0.513623583	11.189407740
	H200	0.753072752	-1.193582972	10.809441017
	H201	3.272537176	-1.140100630	9.753589951
	H202	11.469313274	-7.248630389	2.721969916
	H203	-2.332131886	-5.910597238	-3.957258446
	H204	0.909635221	-6.128295984	-2.026245238
	H205	-3.300957080	-6.076763938	-8.212441144
	H206	-1.889372215	-5.183139253	-8.838759646
	H207	-3.964133426	-4.236261147	-6.778108540
	H208	-4.263531536	1.237517224	-9.207425923
	H209	-4.464072522	-4.031933454	-9.264155446
	H210	-3.724487356	-2.171010512	-1.870825832
	H211	-4.181143702	-4.981066206	-0.745410687
	H212	-3.269671776	-0.848302936	-8.058390025
	H213	-6.131811497	-0.143465063	-7.958800274
	H214	-2.146834729	-2.207832557	-9.318372056
	H215	-1.712084421	-5.821799650	-2.315155040
	H216	-2.462717629	-3.350216614	-7.056587367
	H217	-2.433472770	-3.540538268	-10.243562267
	H218	-7.155012663	-3.404052483	-2.079602959
	H219	-3.631398655	-2.436942631	-0.112790025
	H220	-0.103370450	-5.139002329	-7.667213653
	H221	-2.546042470	-4.341797581	-0.787157030
	H222	-3.545265705	2.594445024	3.612968681
	H223	-2.868146275	0.559759797	1.793882670
	H224	-7.686599693	0.704271503	4.797624492
	H225	-7.165922607	-0.099487333	1.966921342
	H226	-6.724955319	4.041219302	3.287612579
	H227	-7.834643186	8.671215396	5.541479512
	H228	-6.393154026	5.093246447	6.263106120
	H229	-8.621747449	5.380227040	4.627289820
	H230	-6.038682015	6.787126814	3.717088027
	H231	-10.727600662	9.528433891	2.568084428
	H232	-3.171634255	4.260039411	1.804812513
	H233	-4.805278616	3.995159518	1.175744505
	H234	-9.427308493	10.418546649	4.577156955
	H235	-8.242001469	5.592262390	2.916515759
	H236	-6.074043523	2.023848407	2.181647518
	H237	-6.623840266	-0.630228373	4.336495864
	H238	-9.923091127	1.896525711	1.030095408
	H239	-2.648864717	1.558406452	-2.334073633
	H240	-10.241754776	0.154200312	1.120573280
	H241	10.502607318	3.132083577	1.716821965
	H242	8.876013875	3.228557690	1.594313718
	H243	1.322309829	5.981668479	-1.396596853
	H244	-0.764931279	5.518874931	0.533089978
	H245	1.708231638	8.057309020	0.311380564
	H246	-3.722986413	3.586212452	-1.080187230
	H247	-5.468934294	4.223744244	-2.041202011
	H248	-7.228051973	3.450481388	-3.207103657
	H249	-5.048754581	5.459276766	-1.127094112
	H250	-5.999358726	1.311833713	-3.285863563
	H251	-7.752880085	1.329399350	-2.919535677
	H252	-0.331271041	11.652886980	-1.581463641
	H253	-1.497815407	12.947415762	-1.240943922
	H254	-1.307721535	10.171992363	-2.898463043
	H255	-2.966626772	9.614733906	-2.689480818
	H256	-4.284406851	9.336949242	-5.297177747
	H257	-3.320875469	4.946644595	-5.263038122
	H258	-7.377012336	8.673491949	0.544718856
	H259	-2.866266768	8.575923237	2.634059299
	H260	-5.046506740	7.802046677	-2.200601898
	H261	0.570615959	5.415848163	-3.793395841
	H262	1.927862702	7.224708007	-3.280370273
	H263	-1.817223074	4.933314844	-3.006092412
	H264	-8.564457233	-0.212099350	-0.428914937
	H265	3.116980070	10.032911958	-4.301970684
	H266	-6.493858658	-0.910218761	-2.280262880
	H267	-0.088388377	6.416926737	-5.085864584
	H268	-3.384281012	7.033690569	0.790758594
	H269	3.035652638	10.333267779	-2.552955618
	H270	-3.630411552	7.917599613	-6.051239703
	H271	-5.568244994	9.016521849	-3.320291654
	H272	-5.660645289	-3.466316362	-2.827556708
	H273	-5.943041284	-3.362551119	-0.128150596
	H274	-6.812734944	10.308884304	2.318585255
	H275	-5.484974375	1.393579147	-0.855935396
	H276	-0.929416795	8.070007029	-3.427818629
	H277	-2.437474789	6.441401907	-5.573598758

H278	-1.270492803	4.268217234	-4.538002303
H279	-4.043513708	5.924944091	-3.074915071
H280	1.130660482	8.904806885	3.036676529
H281	-0.510762399	9.763456918	0.403041268
H282	-0.099426307	12.625547211	0.665991456
H283	2.464850882	9.429301959	1.985647187
H284	-2.252471434	10.664973098	2.305189867
H285	3.497246692	-2.775047538	-9.503640826
H286	3.605903094	-4.265683585	-8.550792135
H287	7.160323617	-5.051197734	-6.004352231
H288	3.826618392	-4.823781266	-10.854987405
H289	2.316820550	-5.304677974	-10.441126174
H290	5.303014457	4.788483491	-1.270450128
H291	5.769544635	7.478117228	0.050043732
H292	4.355864818	8.114078087	-3.906045865
H293	4.034193224	5.668137264	-0.356859076
H294	6.687414734	8.055034644	-1.174617029
H295	4.537704458	2.957533726	-7.021415935
H296	5.597320037	6.710461501	-2.779731129
H297	5.723629292	-7.037490672	4.180069480
H298	7.236838588	-7.922162986	1.662546522
H299	2.789648689	1.498133841	-6.973005962
H300	5.342423637	-0.050773204	2.740838518
H301	-0.415575324	1.699121281	-8.546187367
H302	-1.668809968	-2.093971075	-4.172628049
H303	-0.421219431	2.203139359	-6.857243619
H304	4.717639071	-4.533624043	3.913345127
H305	1.294430868	0.167026356	-6.607760445
H306	4.208503708	-5.011417283	2.290235848
H307	5.045536520	-7.279950259	2.553563001
H308	6.627261361	-5.453240164	1.698881978
H309	7.081758423	-5.114239323	3.378365472
H310	-5.947173884	-0.666669484	-5.676199050
H311	6.356400572	-9.498848120	4.053984406
H312	5.378125113	-11.928685180	2.482386252
H313	-0.286636615	-0.005179331	-4.103845381
H314	6.995011981	-11.818956803	4.910411166
H315	5.427665468	-12.250684456	4.718659025
H316	5.621031660	-3.208310047	1.378786049
H317	-1.302636857	2.166868727	-4.620559589
H318	-4.023254868	-1.984756319	-4.953992259
H319	7.293649604	-9.917024089	-2.687008096
H320	6.079174672	-7.583790694	-4.652091909
H321	6.489317411	-8.230567425	-0.970709064
H322	6.308994687	-10.506514038	-3.742462438
H323	3.829223553	2.713616559	-9.747055192
H324	6.253805222	-5.161745660	-1.114177429
H325	2.268367425	-7.923366193	0.408111153
H326	2.345287595	-1.859179331	-7.774020159
H327	2.830122847	-9.296299798	-1.401689512
H328	2.166986862	-6.718104980	-7.412733423
H329	1.566591112	-5.342842833	-5.374197631
H330	7.051449143	-12.469677942	2.654168664
H331	5.606398871	-0.955984241	1.278573957
H332	8.361856019	-5.849414991	-0.268826163
H333	7.055343756	-3.831845112	-1.076252167
H334	9.192669307	-6.937928249	1.675900912
H335	4.471270340	-2.805183206	4.771554558
H336	3.190057254	-8.284122681	-4.271397348
H337	4.548359746	-8.601688661	-3.184826104
H338	1.549877648	-8.736967270	-2.487596096
H339	0.545131020	-2.048957193	-6.148847783
H340	-3.674905290	2.288677638	-5.439737399
H341	-5.873286106	1.112906969	-5.801436516
H342	2.292724376	-6.156637531	0.272761349
H343	8.467530917	-7.382723857	-0.482659158
H344	7.380507956	-9.297624807	-0.295665354
H345	4.416749314	-1.115376888	4.631577063
H346	4.644733347	-7.400393842	-7.427116863
H347	6.070181653	-5.987446353	-3.923483708
H348	-0.455570986	-2.801223906	-7.423852619
H349	3.394181710	4.306011742	-7.192209053
H350	8.098793564	0.032118395	3.152975780
H351	7.959267324	0.482583848	1.437998150
H352	8.118777533	-6.561391263	-7.529935338
H353	7.489636313	-7.873288287	-6.780248785
H354	9.973499191	-8.524981707	-3.011251376
H355	8.048454624	-8.131413388	-4.168110256
H356	11.110442236	-7.458161452	-5.253872402
H357	9.888259737	-6.826675550	-2.493194246

	H358	10.662410304	0.209249563	1.137069466
	H359	-2.572043461	13.186075415	4.332808677
	H360	10.666802100	-7.027186326	4.298848621
	H361	10.020213546	-4.580300425	3.229076444
	H362	-4.562723390	12.339234516	2.242371941
	H363	-3.685609523	10.247910370	4.330088834
	H364	-5.308424061	10.947257005	4.192007333
	H365	0.862265614	-3.484312472	-0.982026413
	H366	4.277756726	-1.483247367	-0.311515980
	H367	-6.655146252	6.863401746	-0.639436015
	H368	-0.123431153	-0.832510646	-0.385348262
	H369	2.154879010	-1.574932697	-0.171578270
	H370	1.625359305	-2.230229961	0.054859949
	H371	-2.199106195	-0.201001971	-0.570519366
	Mo372	3.436390971	-4.408467489	-2.781801059
	N373	-7.330859466	-7.230736992	0.379764380
	N374	-8.596626340	-4.655941576	2.778602635
	N375	-7.467264394	-4.918041844	5.342517465
	N376	-4.776327630	-5.712649814	4.967594417
	N377	-3.625924111	-3.359377952	3.908382193
	N378	-3.892621421	-1.642558007	6.191142025
	N379	-2.552662959	-3.044058205	8.360272434
	N380	-0.179268067	-4.351218124	7.348763022
	N381	0.980498162	-2.719796658	5.309677799
	N382	0.955290473	0.125713173	6.170982996
	N383	0.976588650	-0.009893819	9.123607407
	N384	6.134134876	-11.579773107	4.413529618
	N385	6.676783286	-9.294699944	3.101706322
	N386	5.324999088	-3.248833358	2.355378679
	N387	4.854774318	-1.986863335	4.288174158
	N388	5.257021379	-0.939374934	2.252999025
	N389	9.358558469	-7.074272115	2.697498597
	N390	-6.274219671	-2.903101580	-2.210385526
	N391	-2.423635520	-5.551507968	-3.001116623
	N392	-6.382073596	-0.424682462	-1.373306053
	N393	-8.498418719	0.651357200	0.105480080
	N394	-7.658187870	0.319637144	2.763256930
	N395	-5.471140956	2.063015705	3.009079989
	N396	-3.345584110	2.712166058	-0.624493236
	N397	-2.625279104	0.700724859	-0.349702511
	N398	-6.072913677	4.677971598	3.739216175
	N399	-9.635370996	7.701264990	2.910898882
	N400	-9.330829444	9.477030682	4.206203207
	N401	9.664752937	2.579927434	1.518008380
	N402	6.203328112	7.216269736	-0.841439934
	N403	3.827557090	8.473355965	-3.115235699
	N404	1.136434432	7.876414928	-3.289474656
	N405	-3.703933702	6.467828422	-3.888784333
	N406	-4.059270526	8.348789658	-5.241106486
	N407	-4.895540987	8.291143452	-3.086062058
	N408	0.470866332	6.347316890	-0.936165195
	N409	1.029358392	8.225748321	1.059385429
	N410	-0.347203469	10.611900418	0.944394208
	N411	-2.253632962	10.323182459	-2.525345145
	N412	-2.586404947	11.625178929	2.290542395
	N413	2.900621531	-4.477401693	-10.594608551
	N414	1.506729644	-2.411285125	-7.977574508
	N415	0.532270923	0.365669623	-7.276314039
	N416	2.601065243	2.364413723	-7.483601615
	N417	-2.889273981	-2.769760052	-9.751426473
	N418	-4.278481821	-1.006268791	-7.975830613
	N419	7.312731139	-6.885252459	-6.987386935
	N420	3.649180329	-5.574282745	-4.845925959
	N421	2.843419286	-6.432487794	-6.709923746
	N422	8.730337009	-7.369734861	-4.199001283
	N423	-0.083382246	2.400759035	-0.219415148
	N424	0.655645678	1.751689457	-0.777099168
	O425	-6.461396271	-5.280188273	2.306113336
	O426	-6.497412065	-2.868186112	5.115206659
	O427	-4.340898175	-5.078800481	7.115448610
	O428	-1.656187534	-3.989208055	4.864611144
	O429	-2.050218426	-0.554147208	5.403415885
	O430	-1.805678916	-0.969150922	8.963585614
	O431	0.744913417	-4.076880531	9.432912416
	O432	3.002172272	-3.720335528	5.742506508
	O433	3.114291401	-0.006471123	5.435879524
	O434	2.718197391	1.431807363	8.902254592
	O435	3.410598574	-0.540708775	11.700978863
	O436	7.250491169	-10.390824258	1.148975113
	O437	8.484477668	-7.764362229	4.709148710

	O438 12.050822191 -4.750531401 3.179439832<br>O439 -5.703888435 -0.900023122 0.768328533<br>O440 -4.247494984 -4.327101385 -3.590104366<br>O441 -7.228014159 2.496813572 0.545402393<br>O442 -6.734448749 3.090702859 -2.448809183<br>O443 -9.521282198 1.436120287 3.506641951<br>O444 -5.110204900 1.388179295 5.162281591<br>O445 -3.913897213 5.009887789 4.406622379<br>O446 -6.101182810 7.112966417 6.313220432<br>O447 11.140128446 -0.198996419 3.078601233<br>O448 3.915463493 4.561000800 3.657283919<br>O449 3.466846460 8.375690170 -0.867706319<br>O450 0.555334734 10.098849545 -3.332641163<br>O451 -1.626951798 7.224029284 -1.180841092<br>O452 -0.526364371 7.213435206 2.408630315<br>O453 2.474406934 5.679886418 1.388656772<br>O454 1.089333158 11.601017478 2.432798932<br>O455 -2.493452238 13.583482874 1.126842848<br>O456 -3.468295430 11.035389151 -0.725877038<br>O457 -4.557163889 13.620339744 4.548006860<br>O458 -5.688279677 6.875876697 -0.499627810<br>O459 0.714841486 -4.363584123 -8.890640156<br>O460 -1.267370328 -0.570873753 -8.341773110<br>O461 1.107304813 3.778959826 -8.455182542<br>O462 4.839203288 4.453761449 -9.397912166<br>O463 -6.013170588 -2.478506291 -8.128541956<br>O464 -2.189560238 -6.517204686 -5.871580934<br>O465 -0.378526702 -5.651692307 -6.845398631<br>O466 -4.928992994 2.487941466 -7.743188995<br>O467 9.386649232 -5.320324413 -4.910085465<br>O468 12.234768083 -6.854953872 -3.661549894<br>O469 0.026751040 -6.376722891 -1.457490043<br>O470 -0.280732083 -7.471004282 0.482657787<br>O471 4.636026032 -10.904038752 -4.107682954<br>O472 2.381933981 -10.945851343 -3.923044628<br>O473 4.810968778 -5.944373603 -2.021668658<br>O474 4.893534187 -7.665215934 -0.580955499<br>O475 2.244417408 -6.144652800 -2.455939050<br>O476 8.959560161 -6.614595147 -0.080656831<br>O477 6.947199938 -4.677254115 -0.600046163<br>O478 -4.701394409 4.801860338 -1.780804567<br>O479 7.286433456 -10.385834740 -3.550992998<br>O480 7.378882803 -8.667323191 -1.059573686<br>S481 3.209004787 4.945092776 -2.525401895<br>S482 1.647238461 1.998738832 -3.538159714<br>S483 1.521638715 -3.288519948 -3.588011355<br>S484 3.509841910 1.667968070 -0.412464647<br>S485 0.191650636 -0.951246863 -1.700850599<br>S486 3.330421337 -0.711881177 -5.789084841<br>S487 3.454333969 -3.741622380 -0.563127470<br>S488 5.227048054 1.739762178 -3.545764122<br>S489 5.217478707 -3.064220604 -3.590631511<br>S490 6.292440710 -0.934610789 -1.020914699<br>end<br>end

TS

Fe(139) -2.246<br>Fe(140) -1.166<br>Fe(141) -2.442<br>Fe(142) 2.537<br>Fe(143) 2.822<br>Fe(144) 0.853<br>Fe(145) 0.436<br><br>**S=1/2**	hd7hns2bhth_1_53134.94<br>C1 -7.684691391 -5.442391927 2.136842593<br>C2 -8.277518915 -6.552211626 1.261857297<br>C3 -8.170125019 -3.454763507 3.488743907<br>C4 -7.304598770 -3.721607765 4.716232062<br>C5 -6.659094901 -5.270865865 6.490254002<br>C6 -5.155374939 -5.333066569 6.211886437<br>C7 -3.362889049 -5.679092263 4.597313107<br>C8 -2.799830824 -4.261142825 4.473685388<br>C9 -3.260814011 -1.942852057 3.833060093<br>C10 -3.015646153 -1.302882308 5.207442162<br>C11 -3.744522639 -1.056652348 7.508524071<br>C12 -2.602681142 -1.677576398 8.324813778<br>C13 -1.632514074 -3.734878202 9.231164050<br>C14 -0.237844186 -4.050557809 8.673229745<br>C15 1.060293805 -4.748684655 6.699746518<br>C16 1.773191016 -3.672619157 5.875195377<br>C17 1.533510336 -1.612383497 4.514679377<br>C18 1.945392314 -0.431100720 5.425844167<br>C19 0.537267170 -1.188926933 3.415914371<br>C20 1.021896171 0.048695896 2.649815522<br>C21 0.269926236 -2.361084353 2.466265959

	C22	1.190086219	1.300194612	7.022698382
	C23	1.696266706	0.911378034	8.426465127
	C24	-0.065893563	2.205982559	7.076014411
	C25	0.119776066	3.349568130	8.081680011
	C26	-0.388685283	2.763389359	5.683783471
	C27	1.333046324	-0.307538278	10.493472879
	C28	2.799590722	-0.648966389	10.652579107
	C29	6.323883639	-11.676252989	2.968006285
	C30	6.801602756	-10.377896934	2.321514923
	C31	7.067020555	-7.936620220	2.751991073
	C32	8.374468027	-7.578823482	3.484049588
	C33	5.936960465	-6.954736123	3.107704859
	C34	6.264208555	-5.506198325	2.740891290
	C35	5.036055178	-4.602621629	2.857289688
	C36	5.137625336	-2.083737520	2.968496610
	C37	10.650372643	-6.714112322	3.230283043
	C38	10.928776494	-5.228897976	3.206492880
	C39	-5.602427035	-2.676982971	-0.920251771
	C40	-5.908333900	-1.258434236	-0.401778434
	C41	-4.069156041	-2.819018517	-1.040854640
	C42	-3.536803732	-4.250309620	-1.257276906
	C43	-3.434174127	-4.700175894	-2.714429835
	C44	-6.459663667	1.015048664	-1.195964840
	C45	-7.442769250	1.456500682	-0.103345331
	C46	-6.764671208	1.652837362	-2.559508252
	C47	-9.454268384	0.897416447	1.179194195
	C48	-8.874085695	0.901639162	2.598173264
	C49	-6.963325969	0.374065188	4.044845139
	C50	-5.770362180	1.324943545	4.126075477
	C51	-4.388818523	3.038434911	3.063461814
	C52	-4.769786309	4.324020866	3.837053398
	C53	-3.959483837	3.493637336	1.661383530
	C54	-3.454237080	2.437671830	0.732828750
	C55	-2.988645664	1.157278292	0.900465300
	C56	-2.843051862	1.644115909	-1.265038222
	C57	-6.576566967	5.958121574	4.219182016
	C58	-6.338168557	6.063369762	5.725149868
	C59	-8.089771005	6.023500027	3.903856071
	C60	-8.701240079	7.393800475	3.870146090
	C61	-8.497781043	8.493054761	4.682350476
	C62	-10.013763257	8.947036630	3.131120143
	C63	9.515945624	1.519213807	2.527482836
	C64	10.519205379	0.419748193	2.232721970
	C65	8.105425147	0.880401193	2.459053649
	C66	7.006544917	1.858452533	2.802245507
	C67	6.598156881	2.061074154	4.134243469
	C68	6.361944080	2.600139773	1.796867569
	C69	5.574673773	2.961126328	4.452370093
	C70	5.350428318	3.512802539	2.100511875
	C71	4.950862121	3.684114193	3.429426771
	C72	5.140319508	6.859692739	-1.777536213
	C73	4.069499028	7.959474466	-1.867910404
	C74	4.508654472	5.542900148	-1.330961792
	C75	2.904017331	9.565583680	-3.331566446
	C76	1.419163645	9.197676749	-3.307034897
	C77	-0.202400780	7.319963459	-3.133211491
	C78	-0.513738015	6.963721503	-1.656926199
	C79	-0.281759221	6.076269987	-4.050667244
	C80	-1.548446001	5.213339242	-4.029493397
	C81	-2.776168682	5.770635160	-4.770576707
	C82	-4.232452874	7.684980356	-4.080474691
	C83	0.229263108	5.998325993	0.464818623
	C84	0.195473087	7.220605714	1.394194806
	C85	1.269028420	5.009283139	1.028785321
	C86	1.389813041	9.266673787	2.007348437
	C87	0.701870489	10.608741899	1.807080625
	C88	-0.901020399	11.886741513	0.488986793
	C89	-2.088682152	12.436977668	1.320173092
	C90	-1.242517126	11.912396361	-1.003614362
	C91	-2.426000556	11.030193006	-1.377542535
	C92	-3.844599494	11.946721572	2.969091339
	C93	-3.633453564	13.012729941	4.034698764
	C94	-4.426686637	10.654542321	3.611810804
	C95	-4.789528193	9.602012233	2.586714178
	C96	-6.063197675	9.591823761	1.994599300
	C97	-3.858627471	8.639140057	2.161478710
	C98	-6.389485939	8.671393742	0.994709075
	C99	-4.156619477	7.726853373	1.143654520
	C100	-5.423834313	7.760023844	0.565980567
	C101	3.005829238	-3.736571009	-9.335086546

	C102	1.631299191	-3.518315170	-8.729328243
	C103	0.305400879	-2.044358410	-7.233187767
	C104	-0.235556842	-0.677482559	-7.668124612
	C105	0.187112097	1.739812564	-7.639508489
	C106	1.336000950	2.709346601	-7.899297961
	C107	3.666471121	3.348945474	-7.605169324
	C108	4.157282354	3.498601459	-9.030809987
	C109	-3.827983448	-3.302658752	-8.745695004
	C110	-4.813556544	-2.232506456	-8.263178067
	C111	-3.199612242	-4.023370398	-7.530707818
	C112	-2.529353956	-5.344336403	-7.952933325
	C113	-1.701151300	-5.899700064	-6.817551978
	C114	-5.144918999	0.074454188	-7.520823754
	C115	-4.736462853	1.371897641	-8.190657583
	C116	-5.336229383	0.172815826	-5.986648622
	C117	-4.009285561	0.150144759	-5.281945682
	C118	-3.249431425	1.324251860	-5.143451868
	C119	-3.435670413	-1.078076881	-4.905761744
	C120	-1.918782517	1.257346107	-4.717944466
	C121	-2.105447549	-1.142627019	-4.484147666
	C122	-1.332353834	0.021099360	-4.425938986
	C123	7.231944077	-6.100917713	-5.723467054
	C124	8.520892368	-6.217033037	-4.894938648
	C125	6.012929042	-6.511246302	-4.888030655
	C126	4.672074987	-6.262617760	-5.501211744
	C127	4.166553983	-6.800240934	-6.666399084
	C128	2.557492743	-5.702083950	-5.615369547
	C129	9.908957698	-7.486784797	-3.368897426
	C130	11.171543048	-7.235406339	-4.163669455
	C131	0.476572393	-7.004182293	-0.373848200
	C132	1.992979052	-7.073915736	-0.249359726
	C133	2.762227307	-7.170187327	-1.599428072
	C134	2.596880713	-8.583874044	-2.206998329
	C135	3.503265412	-8.880331899	-3.403915159
	C136	3.491770013	-10.361697948	-3.836529728
	C137	4.262781354	-6.942714945	-1.351080027
	C138	3.205340599	-0.677174481	-2.446553194
	Fe139	3.307580404	-1.969835727	-3.911108627
	Fe140	1.826029192	0.723167537	-1.726435359
	Fe141	4.735923647	0.423218609	-1.838347030
	Fe142	3.316129556	0.551980328	-3.983349951
	Fe143	3.491635613	2.698080611	-2.386537139
	Fe144	4.327299532	-2.078828373	-1.568454488
	Fe145	1.868669853	-2.627801819	-1.539359080
	H146	-6.554832396	-7.586116612	0.908652417
	H147	-6.949999225	-6.553823655	-0.289912300
	H148	-9.579621214	-4.738311615	2.531596650
	H149	-9.110628574	-6.134502664	0.673866933
	H150	-8.731026938	-7.295532209	1.939876989
	H151	-7.583445197	-2.792693698	2.835248595
	H152	-8.139698827	-5.573607829	4.958717366
	H153	-9.065311653	-2.906821647	3.812574821
	H154	-6.991713213	-6.251885757	6.853736784
	H155	-5.471198252	-5.847686425	4.233984718
	H156	-6.796558496	-4.543874323	7.302439194
	H157	-2.341167874	-1.815073811	3.250562953
	H158	-4.564193856	-3.626875291	3.643305719
	H159	-3.237227091	-6.199291448	3.638011537
	H160	-3.543062420	0.018780445	7.431072859
	H161	-4.727113779	-2.176769150	5.951662810
	H162	-2.763223973	-6.201367989	5.352970478
	H163	-4.075084014	-1.416973720	3.315569369
	H164	-3.266996854	-3.579896470	7.857480775
	H165	-4.687742112	-1.201419449	8.052584720
	H166	-0.185118253	-3.219093073	2.979434030
	H167	-0.399771577	-2.052811485	1.648706423
	H168	1.208822467	-2.705620683	2.005211227
	H169	-0.402054808	-0.917419410	3.925285231
	H170	-2.096825473	-4.687271783	9.526864959
	H171	-0.573371708	1.965151897	4.951555506
	H172	0.071492313	-0.348642730	8.782806165
	H173	0.273328166	2.985622878	9.107765083
	H174	1.810108751	-0.203929756	1.924992018
	H175	1.423209133	0.828264327	3.313081146
	H176	9.686430823	1.852988961	3.569017789
	H177	-1.287249715	3.396796787	5.726562307
	H178	-0.771684039	3.993914261	8.081225657
	H179	0.445617321	3.383942506	5.316986900
	H180	0.987383241	3.971423481	7.808596869
	H181	-0.916089806	1.585201056	7.412354106

H182	2.021013122	1.866359621	6.578736522
H183	-0.003919882	-0.197487469	6.052571229
H184	0.187157562	0.496414579	2.092162035
H185	1.468340769	4.231376961	0.276879426
H186	0.812089071	4.535103900	1.910786171
H187	2.845892776	5.250796647	2.187538898
H188	3.730147059	4.605161821	4.626545551
H189	4.850979786	4.072149114	1.309779103
H190	6.635287468	2.442040316	0.750478931
H191	7.074559657	1.493546321	4.937828502
H192	-0.022920408	-2.813184711	5.381836571
H193	0.865215997	-5.601053849	6.030802469
H194	1.766218930	-5.076359627	7.471795587
H195	-1.022075127	-4.320473378	6.774085559
H196	-1.474233360	-3.134988486	10.135683573
H197	5.253586043	3.085793631	5.490206216
H198	2.458569115	-1.967301655	4.048625099
H199	1.083634785	0.515380943	11.184577986
H200	0.758694433	-1.195500806	10.800796303
H201	3.279011343	-1.115618769	9.761314473
H202	11.461862552	-7.245922630	2.710256511
H203	-2.333718575	-5.903379985	-3.954820388
H204	0.882066059	-6.120688137	-2.030223688
H205	-3.306639735	-6.079775033	-8.204005954
H206	-1.900640463	-5.184508423	-8.840480975
H207	-3.970423490	-4.237915117	-6.774854249
H208	-4.263765651	1.238755067	-9.192053251
H209	-4.471405119	-4.032698844	-9.260429997
H210	-3.723482246	-2.168216779	-1.860820703
H211	-4.182626147	-4.980709941	-0.739293231
H212	-3.281248719	-0.849518136	-8.051613246
H213	-6.142250935	-0.143079539	-7.955802874
H214	-2.158991074	-2.202375153	-9.315966471
H215	-1.714736192	-5.814066326	-2.313018574
H216	-2.470080115	-3.350269553	-7.053844015
H217	-2.438805626	-3.539162948	-10.237332529
H218	-7.157327671	-3.402535437	-2.071993232
H219	-3.632901995	-2.437993580	-0.105310737
H220	-0.108620648	-5.129704245	-7.677784428
H221	-2.547758036	-4.340295128	-0.784132718
H222	-3.543380053	2.599513966	3.611052498
H223	-2.907502339	0.545176058	1.788635360
H224	-7.686024165	0.704664313	4.803955660
H225	-7.159309750	-0.099395620	1.973734993
H226	-6.725975256	4.039073060	3.283401012
H227	-7.838287227	8.667234826	5.525209266
H228	-6.424601535	5.088225438	6.263913436
H229	-8.632270265	5.369749224	4.609979256
H230	-6.045842844	6.785005365	3.721177390
H231	-10.745830879	9.515787091	2.563413370
H232	-3.169777783	4.250081611	1.798485325
H233	-4.809360791	4.002073946	1.178141704
H234	-9.436240839	10.411627145	4.563860781
H235	-8.241017190	5.588766798	2.903008751
H236	-6.072180886	2.020459450	2.184490763
H237	-6.617014360	-0.626171541	4.345710311
H238	-9.933366147	1.876517054	1.040126601
H239	-2.639981973	1.558427121	-2.330006330
H240	-10.239241008	0.131811268	1.128603746
H241	10.499992272	3.122920353	1.716274176
H242	8.873338212	3.225473716	1.597888998
H243	1.322845799	5.988218904	-1.396975595
H244	-0.762493217	5.528782904	0.540136871
H245	1.716748443	8.062373287	0.302428602
H246	-3.722877098	3.580815981	-1.081160174
H247	-5.475005841	4.221077977	-2.038958386
H248	-7.236567640	3.446809576	-3.199148241
H249	-5.058269731	5.456072826	-1.124574103
H250	-5.998368673	1.312939554	-3.275721053
H251	-7.752912197	1.323685076	-2.914462384
H252	-0.349067973	11.662527951	-1.591863419
H253	-1.525340042	12.948097631	-1.247826769
H254	-1.302683128	10.144686247	-2.873755254
H255	-2.952767097	9.566488453	-2.654312529
H256	-4.264839515	9.356580626	-5.271450776
H257	-3.326524868	4.950855705	-5.256574412
H258	-7.388389487	8.669427791	0.551236868
H259	-2.872097085	8.578263503	2.628653065
H260	-5.069599260	7.792017144	-2.197693308
H261	0.563505433	5.420195686	-3.784190727

	H262	1.923670498	7.222767529	-3.287582799
	H263	-1.824624380	4.940800634	-2.998358252
	H264	-8.563329158	-0.219831234	-0.424216618
	H265	3.120411111	10.023387758	-4.306046722
	H266	-6.492057782	-0.911004438	-2.273076584
	H267	-0.092308556	6.415119556	-5.082331804
	H268	-3.395058751	7.030728394	0.791194270
	H269	3.044671340	10.333702365	-2.558101021
	H270	-3.593465391	7.946356721	-6.024053193
	H271	-5.574948021	9.022789081	-3.307413120
	H272	-5.664169679	-3.467137846	-2.822140611
	H273	-5.943178379	-3.364446446	-0.124063981
	H274	-6.819915128	10.309324875	2.319355373
	H275	-5.489386611	1.393509183	-0.846069943
	H276	-0.931520871	8.079186219	-3.429475959
	H277	-2.442444544	6.443489180	-5.570370314
	H278	-1.277838193	4.272164442	-4.528238897
	H279	-4.053949361	5.927688541	-3.074463468
	H280	1.152063844	8.920640814	3.024090979
	H281	-0.509551685	9.772126214	0.388703565
	H282	-0.109970072	12.635131122	0.652900963
	H283	2.471656856	9.451414445	1.958603908
	H284	-2.262400199	10.668212239	2.298342612
	H285	3.497197131	-2.766669999	-9.501552287
	H286	3.597448981	-4.263393903	-8.557552141
	H287	7.149544454	-5.034152713	-5.979482651
	H288	3.821048339	-4.809871650	-10.864421114
	H289	2.306639049	-5.283318195	-10.459186014
	H290	5.305359447	4.790978800	-1.265615774
	H291	5.765316945	7.476398484	0.066214982
	H292	4.364185805	8.110794775	-3.900288542
	H293	4.041059097	5.668472922	-0.344229765
	H294	6.686060591	8.062222809	-1.153039280
	H295	4.511975780	2.991946892	-6.995957508
	H296	5.604154355	6.714015473	-2.765825631
	H297	5.719686394	-7.038987657	4.185423432
	H298	7.231556492	-7.922597361	1.667169488
	H299	2.772467992	1.523067000	-6.961161132
	H300	5.342811840	-0.060986702	2.749223012
	H301	-0.427913727	1.711003665	-8.547399761
	H302	-1.661718173	-2.104032115	-4.215010214
	H303	-0.436774458	2.201982208	-6.855093357
	H304	4.692446357	-4.540408068	3.895896009
	H305	1.282309779	0.175177853	-6.612978547
	H306	4.203179679	-5.024767470	2.270727393
	H307	5.040637656	-7.286714782	2.559973761
	H308	6.626201737	-5.460303844	1.702835462
	H309	7.066575845	-5.111268469	3.384086748
	H310	-5.961262000	-0.675618175	-5.672546496
	H311	6.361065298	-9.497526032	4.062882730
	H312	5.361950806	-11.918927452	2.491416172
	H313	-0.283807886	-0.022531703	-4.131957668
	H314	6.988303814	-11.817866159	4.912569785
	H315	5.416671808	-12.235283097	4.727865628
	H316	5.605189923	-3.215890284	1.364170412
	H317	-1.314489029	2.162414389	-4.633838120
	H318	-4.027657042	-1.994200632	-4.965450968
	H319	7.282791780	-9.915975892	-2.680526469
	H320	6.075827641	-7.591040183	-4.667658262
	H321	6.473017634	-8.227789061	-0.961578915
	H322	6.295946440	-10.503204792	-3.735434287
	H323	3.846949802	2.680642394	-9.724924264
	H324	6.253766483	-5.158159677	-1.119681380
	H325	2.261293283	-7.908124409	0.409681878
	H326	2.339284653	-1.851927680	-7.776745115
	H327	2.817547093	-9.299328966	-1.399410649
	H328	2.159406284	-6.727154966	-7.412113906
	H329	1.567667765	-5.325109832	-5.392991320
	H330	7.034789278	-12.461532710	2.659296118
	H331	5.618240108	-0.958866765	1.280313573
	H332	8.348428095	-5.848862284	-0.268731636
	H333	7.058041472	-3.828998274	-1.073559916
	H334	9.183330383	-6.936043456	1.672209055
	H335	4.459804811	-2.819867158	4.762464514
	H336	3.181763981	-8.273333301	-4.264560923
	H337	4.540725480	-8.595207205	-3.180333520
	H338	1.541450456	-8.727409456	-2.486338982
	H339	0.533256626	-2.042721317	-6.155316505
	H340	-3.694458767	2.282441680	-5.422875316
	H341	-5.890101759	1.103917474	-5.792587671

	H342	2.287787474	-6.143994773	0.266176945
	H343	8.454078685	-7.382889675	-0.484058460
	H344	7.366669144	-9.295319802	-0.293742682
	H345	4.427524952	-1.129898953	4.630935801
	H346	4.633344175	-7.423483775	-7.420169789
	H347	6.056373133	-6.004788030	-3.917682180
	H348	-0.461376100	-2.794131744	-7.438081510
	H349	3.370440004	4.335061521	-7.217491142
	H350	8.088387244	0.030363469	3.157325653
	H351	7.949565627	0.483265903	1.443124762
	H352	8.111595442	-6.515015795	-7.529143740
	H353	7.493687015	-7.842142082	-6.795772465
	H354	9.956176793	-8.524630740	-3.003693550
	H355	8.034947989	-8.124033991	-4.161153290
	H356	11.091921567	-7.468524506	-5.252738450
	H357	9.883834567	-6.824792467	-2.488868308
	H358	10.656430393	0.203164997	1.145637914
	H359	-2.571495030	13.182052404	4.331692145
	H360	10.664821624	-7.027910701	4.289405656
	H361	10.023949638	-4.574256159	3.221360222
	H362	-4.570399222	12.347208095	2.242177673
	H363	-3.690813380	10.252012035	4.325784991
	H364	-5.314259391	10.950350479	4.188705572
	H365	0.876438851	-3.623943834	-1.024066646
	H366	4.070028304	-1.385105968	-0.209796425
	H367	-6.670592281	6.850926303	-0.624734879
	H368	1.229431997	-1.460908946	-0.541629332
	H369	3.122798164	-1.618523677	-0.585742172
	H370	1.735712657	-2.239127814	-0.028203414
	H371	-2.114849640	-0.177606083	-0.553185719
	Mo372	3.439489025	-4.447918549	-2.791026794
	N373	-7.347479666	-7.225872014	0.370972061
	N374	-8.595425110	-4.647212896	2.772654995
	N375	-7.464881423	-4.912351202	5.335616049
	N376	-4.770851323	-5.703079447	4.963771763
	N377	-3.620711664	-3.349464681	3.902466858
	N378	-3.893597369	-1.631997136	6.181816193
	N379	-2.552774790	-3.033854211	8.347927624
	N380	-0.176593260	-4.348575280	7.346510066
	N381	0.990705333	-2.720121259	5.309704137
	N382	0.957317834	0.131870382	6.167665388
	N383	0.975808786	-0.006563819	9.117740292
	N384	6.127183154	-11.569715092	4.420078887
	N385	6.677476001	-9.291865856	3.109418086
	N386	5.311866194	-3.257798632	2.341828998
	N387	4.855840404	-2.004218715	4.283441001
	N388	5.266318079	-0.947252111	2.256047441
	N389	9.351852350	-7.068995414	2.693583317
	N390	-6.275978698	-2.902541769	-2.204487647
	N391	-2.425515200	-5.544546804	-2.998997384
	N392	-6.382458923	-0.425816294	-1.365544713
	N393	-8.501261382	0.642723746	0.112010903
	N394	-7.654232604	0.317469921	2.770057468
	N395	-5.470240934	2.062689045	3.012406364
	N396	-3.349971633	2.706675369	-0.625138055
	N397	-2.627434425	0.691172574	-0.349139864
	N398	-6.077228529	4.675900846	3.739498199
	N399	-9.650602631	7.690189263	2.905061761
	N400	-9.340832348	9.469366812	4.194610066
	N401	9.659420655	2.573676124	1.520986156
	N402	6.203026866	7.221069981	-0.825129553
	N403	3.831039916	8.469322665	-3.112325985
	N404	1.135417091	7.878613140	-3.292560576
	N405	-3.711505696	6.474104874	-3.885263774
	N406	-4.065280456	8.362173880	-5.229744085
	N407	-4.910281025	8.288952100	-3.077399282
	N408	0.469710950	6.350780608	-0.936984690
	N409	1.037741454	8.234362509	1.049699717
	N410	-0.351051902	10.619563406	0.933528047
	N411	-2.251923535	10.290485360	-2.506930109
	N412	-2.594841851	11.629011706	2.284686009
	N413	2.896595331	-4.459080422	-10.604778898
	N414	1.501044348	-2.402867508	-7.982000257
	N415	0.527435789	0.372901525	-7.287409837
	N416	2.583972627	2.386390682	-7.474702834
	N417	-2.898466333	-2.768999016	-9.747785443
	N418	-4.289790366	-1.008285415	-7.970216991
	N419	7.309598352	-6.852700364	-6.989381825
	N420	3.649591173	-5.560568170	-4.861928118
	N421	2.838967038	-6.436156984	-6.714519186

	N422 8.718554849 -7.363924110 -4.190992431<br>N423 -0.079656327 2.340304812 -0.139726763<br>N424 0.645741991 1.754528912 -0.777524248<br>O425 -6.462664235 -5.284079306 2.299781486<br>O426 -6.494473316 -2.862667423 5.111112304<br>O427 -4.341264452 -5.069709767 7.112927362<br>O428 -1.654970082 -3.972980981 4.871136232<br>O429 -2.061645732 -0.529094254 5.391091758<br>O430 -1.808787052 -0.959610862 8.957795544<br>O431 0.737147959 -4.077469124 9.435781661<br>O432 3.008026864 -3.736899006 5.730554930<br>O433 3.120693603 -0.016252899 5.449339144<br>O434 2.715912912 1.437644771 8.904471302<br>O435 3.402828040 -0.504627076 11.706567866<br>O436 7.232521737 -10.386085287 1.149852243<br>O437 8.486777964 -7.758443870 4.709374731<br>O438 12.054435449 -4.754987754 3.191624123<br>O439 -5.717137399 -0.907131207 0.779395939<br>O440 -4.255148825 -4.328628080 -3.584251473<br>O441 -7.238160463 2.493266943 0.552853256<br>O442 -6.742892340 3.088753433 -2.440127476<br>O443 -9.528263992 1.414433270 3.515641487<br>O444 -5.116327323 1.405247098 5.171575237<br>O445 -3.920239897 5.010969395 4.412272004<br>O446 -6.115488103 7.105243747 6.316219734<br>O447 11.125698313 -0.207844768 3.088643250<br>O448 3.919617059 4.572743161 3.670986118<br>O449 3.461746057 8.376953644 -0.866290977<br>O450 0.561659676 10.102056962 -3.315848056<br>O451 -1.625910984 7.234573244 -1.176269014<br>O452 -0.511121045 7.227541666 2.410362267<br>O453 2.480606601 5.679067110 1.386777077<br>O454 1.081456710 11.611958935 2.422770019<br>O455 -2.500568965 13.589402277 1.125495293<br>O456 -3.481106725 11.028317175 -0.727705226<br>O457 -4.554491136 13.625207950 4.550412357<br>O458 -5.702739606 6.869286271 -0.492371457<br>O459 0.708222798 -4.350903140 -8.902321194<br>O460 -1.280010221 -0.562447419 -8.340316763<br>O461 1.086181707 3.793321194 -8.451439242<br>O462 4.865570787 4.420767671 -9.398461108<br>O463 -6.022806481 -2.482049176 -8.126336952<br>O464 -2.180429461 -6.513347158 -5.869772982<br>O465 -0.377847558 -5.640934509 -6.853407453<br>O466 -4.939113067 2.485697215 -7.729490673<br>O467 9.382616200 -5.316582048 -4.900387051<br>O468 12.230713413 -6.881712005 -3.664719032<br>O469 0.021799901 -6.357101900 -1.459423665<br>O470 -0.285999243 -7.449613939 0.479020732<br>O471 4.622220801 -10.900754682 -4.102932571<br>O472 2.367114076 -10.932148460 -3.918556163<br>O473 4.826835181 -5.983155997 -2.038305142<br>O474 4.883261270 -7.672155272 -0.556526568<br>O475 2.260806456 -6.158590296 -2.474694516<br>O476 8.947329246 -6.613630896 -0.085166052<br>O477 6.935792038 -4.670554067 -0.594647884<br>O478 -4.709559839 4.803202310 -1.781474861<br>O479 7.273575633 -10.381642269 -3.546179522<br>O480 7.362940791 -8.663002981 -1.056227248<br>S481 3.209511553 4.957636888 -2.512142526<br>S482 1.652691925 2.014588271 -3.561596678<br>S483 1.528652254 -3.341488002 -3.622816351<br>S484 3.496751069 1.673783756 -0.408617262<br>S485 0.230763271 -0.927864008 -1.687269180<br>S486 3.313379939 -0.746347557 -5.751303828<br>S487 3.431668963 -3.884836462 -0.545919713<br>S488 5.210933656 1.735193987 -3.540141801<br>S489 5.215713115 -3.036313327 -3.473783586<br>S490 6.222600941 -0.947745128 -0.966674033<br>end

product (13)

Fe(139) 0.578 -2.263<br>Fe(140) 0.421 -0.905<br>Fe(141) 0.552 -2.531<br>Fe(142) 0.508 2.593<br>Fe(143) 0.558 2.904	hd7hns2bhth.car_5<br>C1 -7.679193696 -5.446363924 2.144960485<br>C2 -8.262054531 -6.560927817 1.269722528<br>C3 -8.176348258 -3.461374044 3.497009221<br>C4 -7.309637958 -3.727097044 4.724107376<br>C5 -6.662837661 -5.276753312 6.497623875<br>C6 -5.159205607 -5.341908249 6.218612485

Fe(144) 0.242 0.785<br>Fe(145) 0.105 0.120<br><br>**S=1/2**	C7 -3.370341270 -5.689925179 4.599529532<br>C8 -2.804003626 -4.273083357 4.477296065<br>C9 -3.260852328 -1.951343971 3.845054040<br>C10 -3.011261200 -1.317962387 5.221802085<br>C11 -3.746661250 -1.068770869 7.521243951<br>C12 -2.603444408 -1.688413436 8.336280237<br>C13 -1.627220701 -3.743010061 9.240438169<br>C14 -0.233947827 -4.050836565 8.674860465<br>C15 1.054708888 -4.742992840 6.694035326<br>C16 1.764782818 -3.663106745 5.874132712<br>C17 1.530604002 -1.611155024 4.509321187<br>C18 1.941809795 -0.426976770 5.413458322<br>C19 0.547416725 -1.186844489 3.398198465<br>C20 1.073651158 0.020990774 2.614793106<br>C21 0.259487062 -2.376364023 2.478335798<br>C22 1.187272977 1.294522571 7.017581992<br>C23 1.696898505 0.903814474 8.420499611<br>C24 -0.069690094 2.198122254 7.069276947<br>C25 0.113818809 3.341362191 8.075005185<br>C26 -0.390775106 2.754551476 5.676113400<br>C27 1.337105986 -0.317206113 10.489770361<br>C28 2.799951890 -0.679224062 10.641206308<br>C29 6.325528694 -11.677386544 2.950140796<br>C30 6.806775987 -10.375361981 2.309445175<br>C31 7.066952773 -7.933115511 2.745106135<br>C32 8.374059926 -7.576681580 3.479577442<br>C33 5.939054009 -6.949629297 3.104454117<br>C34 6.272116424 -5.500839875 2.743981206<br>C35 5.050933839 -4.590559515 2.876588800<br>C36 5.149090653 -2.073106696 2.994179070<br>C37 10.648560562 -6.702634669 3.231273537<br>C38 10.921044197 -5.215987290 3.202092979<br>C39 -5.605850396 -2.673269806 -0.925206131<br>C40 -5.910611541 -1.254289585 -0.406664498<br>C41 -4.072008331 -2.814119616 -1.049620193<br>C42 -3.539785379 -4.245089849 -1.268801888<br>C43 -3.443709209 -4.698715321 -2.725301989<br>C44 -6.457142617 1.019499850 -1.203585684<br>C45 -7.439050977 1.463819546 -0.111338869<br>C46 -6.764914490 1.654476945 -2.567830776<br>C47 -9.453544384 0.916731213 1.169240370<br>C48 -8.875606133 0.916352354 2.589070645<br>C49 -6.972806316 0.368198798 4.038312102<br>C50 -5.775552155 1.313953773 4.120224297<br>C51 -4.394241309 3.027481893 3.055268301<br>C52 -4.769122464 4.323289766 3.820352980<br>C53 -3.954723773 3.464298302 1.649853132<br>C54 -3.408266144 2.412223596 0.735703569<br>C55 -2.846901895 1.170543402 0.913179279<br>C56 -2.768812940 1.630980983 -1.259291354<br>C57 -6.573576940 5.962472643 4.222780412<br>C58 -6.319860106 6.067113192 5.726031614<br>C59 -8.088688703 6.030703300 3.918703049<br>C60 -8.695376280 7.403085867 3.883647134<br>C61 -8.493527519 8.500234069 4.699113107<br>C62 -9.999554671 8.961212569 3.140328424<br>C63 9.517153689 1.524073828 2.515881285<br>C64 10.523936021 0.427964576 2.219100068<br>C65 8.109024918 0.882114060 2.447330934<br>C66 7.007226716 1.857252631 2.789661504<br>C67 6.602799285 2.064610389 4.122193120<br>C68 6.355390420 2.590301231 1.782711955<br>C69 5.575770196 2.961098775 4.439179237<br>C70 5.339244346 3.498104776 2.085002227<br>C71 4.944252209 3.675119841 3.414809893<br>C72 5.134991315 6.864512453 -1.790288987<br>C73 4.066255058 7.967814048 -1.872107577<br>C74 4.503382205 5.545306360 -1.349287745<br>C75 2.895260488 9.577205695 -3.330477929<br>C76 1.410651591 9.207391645 -3.312412328<br>C77 -0.206294710 7.323960391 -3.132282346<br>C78 -0.518666907 6.967644771 -1.657285286<br>C79 -0.285156587 6.084811496 -4.054136403<br>C80 -1.550904409 5.220201560 -4.034354199<br>C81 -2.779861948 5.775670248 -4.774180930<br>C82 -4.237668719 7.686221191 -4.089998745<br>C83 0.219802422 5.994526767 0.461246182<br>C84 0.180910403 7.211673604 1.396836525<br>C85 1.261292608 5.006151244 1.022150947<br>C86 1.373843137 9.254455064 2.024792579

C87	0.699634615	10.602750300	1.821922613
C88	-0.896524421	11.888684494	0.502252383
C89	-2.087157705	12.440472151	1.328284614
C90	-1.227843179	11.917079624	-0.992036478
C91	-2.418233736	11.048946785	-1.376773571
C92	-3.844184113	11.947672029	2.975277847
C93	-3.638958755	13.016199428	4.040018922
C94	-4.425332981	10.655409046	3.617982550
C95	-4.786434172	9.603988703	2.591364310
C96	-6.058199491	9.595606131	1.995135771
C97	-3.854404508	8.642018768	2.166972398
C98	-6.381003118	8.678189617	0.991210124
C99	-4.149172367	7.732475044	1.146032868
C100	-5.414000718	7.767705609	0.563142471
C101	3.004796691	-3.747820958	-9.333672519
C102	1.631156179	-3.530888673	-8.725110517
C103	0.312028941	-2.049285477	-7.226974105
C104	-0.221638018	-0.682769944	-7.669911475
C105	0.198853949	1.734615508	-7.636314557
C106	1.350270549	2.697071809	-7.909388182
C107	3.679500858	3.337224312	-7.620807142
C108	4.140619096	3.525495744	-9.052500752
C109	-3.827362570	-3.302349972	-8.753997079
C110	-4.811477457	-2.230155941	-8.273188123
C111	-3.200082544	-4.021802054	-7.537333627
C112	-2.528763981	-5.342794842	-7.957926064
C113	-1.708946734	-5.904446226	-6.819316795
C114	-5.142570059	0.078244243	-7.535494828
C115	-4.736012736	1.372505956	-8.212046095
C116	-5.341270184	0.183461000	-6.003331690
C117	-4.017854433	0.166969715	-5.294041161
C118	-3.260284511	1.343082544	-5.163600824
C119	-3.446850358	-1.059100872	-4.905917511
C120	-1.930671190	1.280187026	-4.735536882
C121	-2.120466942	-1.117410242	-4.475677390
C122	-1.348108222	0.046659261	-4.430030641
C123	7.228226496	-6.098668608	-5.719561389
C124	8.519851135	-6.211375660	-4.894745568
C125	6.008268109	-6.471801966	-4.864128900
C126	4.665655228	-6.227682277	-5.476844590
C127	4.169890029	-6.768301909	-6.645256952
C128	2.550743697	-5.676396828	-5.604316137
C129	9.912334578	-7.477047676	-3.369210079
C130	11.174927411	-7.214955249	-4.160236952
C131	0.453738037	-7.071046791	-0.365789329
C132	1.970937327	-7.114058708	-0.251820869
C133	2.739112238	-7.198097001	-1.602448364
C134	2.594394223	-8.612277978	-2.215598944
C135	3.507528391	-8.899897279	-3.409735386
C136	3.508227089	-10.381026804	-3.849762559
C137	4.231593131	-6.937523219	-1.339873079
C138	3.201891853	-0.673507418	-2.451714219
Fe139	3.325011933	-1.974396564	-3.887676245
Fe140	1.770556702	0.650840373	-1.745859725
Fe141	4.774230136	0.442024221	-1.903129068
Fe142	3.320838064	0.544449070	-4.018882821
Fe143	3.481777909	2.683048329	-2.458665086
Fe144	4.287324395	-2.027370005	-1.465005945
Fe145	1.684599267	-2.598189985	-1.541549144
H146	-6.530611210	-7.582553270	0.917993987
H147	-6.935804108	-6.557747928	-0.284395529
H148	-9.577790983	-4.752056788	2.536387837
H149	-9.097461228	-6.150513306	0.679953342
H150	-8.710900913	-7.306149125	1.948543927
H151	-7.594528517	-2.792744768	2.845950202
H152	-8.141788363	-5.580717832	4.964747749
H153	-9.075497686	-2.920929618	3.822548053
H154	-6.996935548	-6.256703349	6.862731168
H155	-5.479153888	-5.852489685	4.240546231
H156	-6.799101548	-4.548307253	7.308671862
H157	-2.342403803	-1.822863689	3.260976388
H158	-4.567703587	-3.632884807	3.651015191
H159	-3.249259948	-6.208128094	3.638457301
H160	-3.548245564	0.007435464	7.446473657
H161	-4.732135836	-2.178774827	5.959523916
H162	-2.769272979	-6.215607654	5.351770770
H163	-4.073010377	-1.420253165	3.330089386
H164	-3.269157909	-3.591865997	7.873962215
H165	-4.689817850	-1.217107284	8.064436094
H166	-0.237142585	-3.200074183	3.006335989

	H167	-0.380776527	-2.070520236	1.636502165
	H168	1.197092368	-2.773791911	2.061061960
	H169	-0.392107311	-0.882691592	3.889596567
	H170	-2.085608592	-4.698138952	9.536389701
	H171	-0.573684149	1.955483113	4.944134731
	H172	0.072261964	-0.355166727	8.780477642
	H173	0.266954370	2.976573845	9.100809511
	H174	1.974611681	-0.227745319	2.033864647
	H175	1.322042983	0.869488956	3.269300432
	H176	9.687361660	1.855513303	3.558205489
	H177	-1.290159920	3.386809894	5.716573516
	H178	-0.778065263	3.984843250	8.073936805
	H179	0.443323618	3.375634630	5.309788284
	H180	0.981335164	3.963678074	7.802766740
	H181	-0.919699730	1.576137107	7.403746466
	H182	2.017377971	1.861167750	6.572700937
	H183	-0.003435940	-0.211684480	6.054938976
	H184	0.307605328	0.367358952	1.906596724
	H185	1.458702486	4.229262517	0.268921716
	H186	0.806045338	4.530575449	1.904347282
	H187	2.838338562	5.246850469	2.178179798
	H188	3.722621861	4.595950365	4.611343116
	H189	4.834535664	4.050530130	1.292722388
	H190	6.627723814	2.429662534	0.736465396
	H191	7.084603273	1.503764475	4.927300868
	H192	-0.033249941	-2.815431201	5.363592068
	H193	0.856118464	-5.590818416	6.020425649
	H194	1.763594103	-5.074843353	7.461521372
	H195	-1.027765753	-4.319481934	6.779280106
	H196	-1.467745920	-3.143920860	10.145297817
	H197	5.258257312	3.089983322	5.477665109
	H198	2.454134741	-1.970778254	4.043976921
	H199	1.102779296	0.510375845	11.179919203
	H200	0.751483253	-1.196719349	10.799657085
	H201	3.271834466	-1.145323568	9.745323020
	H202	11.461802120	-7.233025820	2.713163898
	H203	-2.351257866	-5.913254324	-3.964024726
	H204	0.817305855	-6.194741797	-2.033912514
	H205	-3.305470847	-6.076232202	-8.216751419
	H206	-1.893653945	-5.181529377	-8.840710698
	H207	-3.971565984	-4.235825914	-6.781949606
	H208	-4.262971669	1.234484180	-9.212574764
	H209	-4.471328305	-4.032251019	-9.268490758
	H210	-3.726189617	-2.161066789	-1.867489304
	H211	-4.181303301	-4.976091351	-0.746149418
	H212	-3.278260176	-0.848582389	-8.058552161
	H213	-6.138797650	-0.142363561	-7.972809461
	H214	-2.153889649	-2.207733144	-9.322823150
	H215	-1.738069272	-5.828598067	-2.319893571
	H216	-2.471513560	-3.347752779	-7.060091511
	H217	-2.438783621	-3.542217903	-10.245462130
	H218	-7.159445004	-3.401602965	-2.077267536
	H219	-3.631818728	-2.431847513	-0.116595739
	H220	-0.109864243	-5.138731063	-7.669647753
	H221	-2.546914502	-4.332648839	-0.802877974
	H222	-3.555050341	2.588950201	3.612647346
	H223	-2.697546273	0.584166867	1.810160074
	H224	-7.693865984	0.699972801	4.798500927
	H225	-7.169686677	-0.100578320	1.966762596
	H226	-6.732187005	4.040720628	3.290506640
	H227	-7.836379237	8.671755292	5.544338637
	H228	-6.390072860	5.090360564	6.264306654
	H229	-8.628068725	5.381276075	4.631001320
	H230	-6.045947762	6.788849880	3.720494614
	H231	-10.727293031	9.532708425	2.569859615
	H232	-3.185077434	4.241544599	1.785475943
	H233	-4.809115535	3.948201911	1.149686744
	H234	-9.426603547	10.421408340	4.579220293
	H235	-8.247805571	5.593438381	2.920334503
	H236	-6.080793711	2.013341971	2.181253571
	H237	-6.630470122	-0.634166783	4.336527548
	H238	-9.924292478	1.899805886	1.029091589
	H239	-2.566356564	1.545166537	-2.324417901
	H240	-10.244785064	0.157582321	1.118155067
	H241	10.497447581	3.131773013	1.709557944
	H242	8.870732272	3.231393503	1.591236057
	H243	1.315397123	5.989747170	-1.399230634
	H244	-0.770752275	5.521541166	0.530804648
	H245	1.700503286	8.062064184	0.311109481
	H246	-3.741675971	3.524250904	-1.086630394

H247	-5.485633833	4.228346644	-2.044392519
H248	-7.231469660	3.445184293	-3.218667684
H249	-5.054117551	5.453524528	-1.123361520
H250	-5.998188685	1.315823450	-3.284206929
H251	-7.752767021	1.321387350	-2.920624065
H252	-0.332843573	11.659248300	-1.574426362
H253	-1.499115572	12.955262343	-1.239467636
H254	-1.311205783	10.183998975	-2.898148015
H255	-2.970271878	9.626757576	-2.690405129
H256	-4.296188713	9.344199250	-5.301274624
H257	-3.328551064	4.955388465	-5.260976416
H258	-7.378672001	8.677614638	0.544871628
H259	-2.869528050	8.580185599	2.637498944
H260	-5.052973627	7.814124319	-2.199845294
H261	0.561586802	5.429447167	-3.791273515
H262	1.920199853	7.234397583	-3.279911058
H263	-1.827082961	4.945547043	-3.003796967
H264	-8.563436347	-0.211040719	-0.427935210
H265	3.114289238	10.040616119	-4.301576627
H266	-6.489270658	-0.906265338	-2.279430671
H267	-0.097691505	6.429465831	-5.084280169
H268	-3.387017409	7.036625691	0.794515824
H269	3.033259321	10.341430217	-2.552593217
H270	-3.644088727	7.923076713	-6.053296984
H271	-5.582054242	9.025045336	-3.321044772
H272	-5.666426480	-3.461165984	-2.827979482
H273	-5.944799034	-3.361116404	-0.128616280
H274	-6.815779899	10.312426007	2.319554466
H275	-5.485170796	1.396295444	-0.856108845
H276	-0.936418433	8.084356246	-3.425195521
H277	-2.447637127	6.451311847	-5.572257639
H278	-1.279166753	4.280400084	-4.534675093
H279	-4.057937720	5.932400071	-3.075536105
H280	1.121215537	8.906442024	3.037154235
H281	-0.520485001	9.772441420	0.407205837
H282	-0.104375266	12.634647127	0.672510061
H283	2.457695787	9.430378389	1.988897020
H284	-2.259363523	10.672957648	2.307840760
H285	3.490242898	-2.776598565	-9.509896079
H286	3.601404714	-4.265678781	-8.554272514
H287	7.159537881	-5.038002215	-6.003250704
H288	3.820688043	-4.827196298	-10.858888325
H289	2.312262652	-5.309239245	-10.442337827
H290	5.302189065	4.796214325	-1.278722075
H291	5.766651099	7.486525231	0.051116012
H292	4.349672827	8.119822813	-3.905244501
H293	4.031156247	5.670204529	-0.364409685
H294	6.686838420	8.062774658	-1.172113198
H295	4.536934350	2.969128333	-7.035985164
H296	5.596962952	6.723927041	-2.780083238
H297	5.721185594	-7.037139211	4.181750014
H298	7.231906762	-7.914907687	1.660364595
H299	2.788005983	1.510274312	-6.975802317
H300	5.366399277	-0.048694786	2.789682595
H301	-0.419968885	1.699178122	-8.541351128
H302	-1.675897863	-2.069389102	-4.175283651
H303	-0.419376799	2.205806262	-6.852993216
H304	4.721669229	-4.531428584	3.919899014
H305	1.264706073	0.177118772	-6.574207009
H306	4.209083498	-5.003371754	2.296160817
H307	5.041493944	-7.275600240	2.555202354
H308	6.626623140	-5.451181939	1.703659229
H309	7.082190610	-5.115174451	3.383191137
H310	-5.966029920	-0.664574389	-5.687435734
H311	6.351443786	-9.496160417	4.048335312
H312	5.362666811	-11.911419363	2.470632658
H313	-0.308190210	-0.001174023	-4.110305441
H314	6.987044458	-11.813484077	4.894982604
H315	5.419213711	-12.244894965	4.706769614
H316	5.670554381	-3.206422448	1.402776803
H317	-1.325598894	2.184780163	-4.652318178
H318	-4.038896704	-1.975877527	-4.957237709
H319	7.290013024	-9.905015426	-2.688803118
H320	6.065025800	-7.546121341	-4.615481825
H321	6.476361397	-8.226448465	-0.967922188
H322	6.311008269	-10.499499967	-3.747653829
H323	3.823044653	2.722927881	-9.761250924
H324	6.219464683	-5.147392127	-1.076776754
H325	2.257627845	-7.943883153	0.404587673
H326	2.343608834	-1.865511914	-7.777458090

	H327	2.823773418	-9.324448130	-1.407450549
	H328	2.169144270	-6.705896039	-7.403952350
	H329	1.557676386	-5.302275705	-5.391691285
	H330	7.033851255	-12.463075150	2.637624445
	H331	5.738112942	-0.941439351	1.347854060
	H332	8.344386135	-5.840191435	-0.260399374
	H333	7.045468315	-3.833635052	-1.055302237
	H334	9.183877878	-6.926828059	1.670687866
	H335	4.410136519	-2.805457249	4.770008143
	H336	3.186607084	-8.293194716	-4.270958705
	H337	4.542088913	-8.610789970	-3.178693626
	H338	1.541889057	-8.773642373	-2.495929593
	H339	0.544043419	-2.045262126	-6.150049248
	H340	-3.705030619	2.298932346	-5.450918047
	H341	-5.897559187	1.114701440	-5.816474044
	H342	2.257700374	-6.180943823	0.262194356
	H343	8.458325484	-7.370858003	-0.487494267
	H344	7.378796020	-9.287359043	-0.300083769
	H345	4.407748453	-1.111982115	4.641538514
	H346	4.645195126	-7.388846511	-7.396314755
	H347	6.063625121	-5.939710160	-3.907177403
	H348	-0.458867428	-2.796623692	-7.425936147
	H349	3.389359033	4.314805593	-7.206492937
	H350	8.094816547	0.032485910	3.146208712
	H351	7.955762529	0.484914500	1.430923594
	H352	8.094916433	-6.565097090	-7.518601197
	H353	7.467037092	-7.868668819	-6.754329877
	H354	9.965339236	-8.514857845	-3.005022166
	H355	8.043197256	-8.123523977	-4.166161832
	H356	11.102872011	-7.453170315	-5.248728785
	H357	9.881570963	-6.815684058	-2.488738117
	H358	10.660570014	0.211719255	1.131925234
	H359	-2.578058261	13.190275848	4.338032954
	H360	10.661202729	-7.012816391	4.291156385
	H361	10.013654297	-4.564830678	3.224914405
	H362	-4.569927069	12.345958863	2.246897411
	H363	-3.689210780	10.253623966	4.332124260
	H364	-5.313368965	10.950445908	4.194584357
	H365	0.647775798	-3.646903131	-1.261510144
	H366	4.140373303	-1.458305467	0.116652775
	H367	-6.654609304	6.867040334	-0.638878081
	H368	1.257048233	-1.726093081	-0.190348331
	H369	3.460961941	-1.189029365	-0.243478916
	H370	1.547368024	-2.447581262	0.107065272
	H371	-1.851044956	-0.099326950	-0.551556147
	Mo372	3.335143833	-4.438210625	-2.731090769
	N373	-7.326676865	-7.229618910	0.380561146
	N374	-8.594413612	-4.654722971	2.777890861
	N375	-7.468460299	-4.918583260	5.342810473
	N376	-4.777240250	-5.711773323	4.969644858
	N377	-3.623739365	-3.357415712	3.911083610
	N378	-3.894054810	-1.642610648	6.193395899
	N379	-2.554011197	-3.044680139	8.361803338
	N380	-0.179075471	-4.344573593	7.346875851
	N381	0.980753631	-2.716197754	5.301943228
	N382	0.955755981	0.127206601	6.161724569
	N383	0.976961819	-0.013052904	9.114315941
	N384	6.124709005	-11.573149723	4.400968558
	N385	6.675131926	-9.289565914	3.097609875
	N386	5.333579980	-3.244786198	2.366602449
	N387	4.824024050	-1.994235515	4.295741950
	N388	5.305929203	-0.932098517	2.287809470
	N389	9.351250279	-7.061365647	2.692228576
	N390	-6.279558467	-2.899157762	-2.209335083
	N391	-2.442425058	-5.552794069	-3.008702622
	N392	-6.383559166	-0.421641570	-1.371351837
	N393	-8.500729214	0.654211034	0.103982150
	N394	-7.663798772	0.316990199	2.763366067
	N395	-5.479978970	2.057360306	3.009660809
	N396	-3.346575405	2.663530099	-0.629343742
	N397	-2.469736071	0.709819471	-0.335895372
	N398	-6.077192574	4.678933494	3.735570081
	N399	-9.638342595	7.703952913	2.913800340
	N400	-9.331434197	9.479585789	4.208787028
	N401	9.658078583	2.581334585	1.512298756
	N402	6.201290195	7.224431236	-0.839882867
	N403	3.823135307	8.481097012	-3.114166052
	N404	1.129882720	7.887740875	-3.288485153
	N405	-3.715897792	6.477135945	-3.888070282
	N406	-4.074894096	8.355194393	-5.244654949

	N407	-4.907753406	8.301010934	-3.087602960
	N408	0.464065660	6.353329622	-0.937817503
	N409	1.022164217	8.228998801	1.059827309
	N410	-0.353243808	10.620138723	0.948702334
	N411	-2.256786838	10.334309275	-2.523963763
	N412	-2.592595765	11.633392825	2.294191562
	N413	2.894829329	-4.481437575	-10.597318177
	N414	1.503008796	-2.413675090	-7.980583079
	N415	0.533545119	0.369046514	-7.273834353
	N416	2.600357071	2.371045873	-7.492863211
	N417	-2.895792302	-2.770735791	-9.755445020
	N418	-4.286741703	-1.006330492	-7.979293942
	N419	7.291672103	-6.881734713	-6.967630506
	N420	3.635621144	-5.530306314	-4.841158608
	N421	2.841758099	-6.410134721	-6.701779508
	N422	8.722253905	-7.358951464	-4.192887210
	N423	-0.054667277	2.418395923	-0.223483336
	N424	0.623114983	1.743346859	-0.819783483
	O425	-6.458107549	-5.280236036	2.309957750
	O426	-6.501092719	-2.867177749	5.120238536
	O427	-4.343515029	-5.081122597	7.118980452
	O428	-1.656616245	-3.989328617	4.871805296
	O429	-2.049622242	-0.555074381	5.410042773
	O430	-1.807091530	-0.970157724	8.965800653
	O431	0.745225320	-4.076503578	9.431858676
	O432	3.001793738	-3.717308220	5.739292434
	O433	3.114361201	-0.001661016	5.425278957
	O434	2.719415178	1.427878342	8.894441849
	O435	3.409187806	-0.546423340	11.692703492
	O436	7.244925379	-10.380256685	1.140504412
	O437	8.484801576	-7.759080170	4.704595248
	O438	12.043953453	-4.736631602	3.173370090
	O439	-5.721707495	-0.902805408	0.774653296
	O440	-4.262319580	-4.324208715	-3.595889966
	O441	-7.232708167	2.500743897	0.544621823
	O442	-6.748583018	3.090901764	-2.451005989
	O443	-9.524784486	1.437934647	3.505077864
	O444	-5.117103166	1.385189033	5.163580354
	O445	-3.912537903	5.010166798	4.384681537
	O446	-6.100930479	7.110321248	6.315508457
	O447	11.133736172	-0.197431714	3.074268217
	O448	3.909873460	4.559629956	3.655508572
	O449	3.466299540	8.385329788	-0.865573098
	O450	0.552852262	10.111364519	-3.333012829
	O451	-1.630243190	7.240184741	-1.176024471
	O452	-0.526989381	7.210925009	2.412058973
	O453	2.473021542	5.676064043	1.378051889
	O454	1.088638311	11.603337421	2.436099405
	O455	-2.499553772	13.592172469	1.131163636
	O456	-3.470393445	11.042121726	-0.722116221
	O457	-4.563069481	13.626040339	4.553609225
	O458	-5.687679691	6.880031906	-0.498904794
	O459	0.708745758	-4.365174124	-8.894081429
	O460	-1.249839604	-0.569190395	-8.365955338
	O461	1.102116141	3.780184884	-8.464166181
	O462	4.833734690	4.462396683	-9.412393362
	O463	-6.021439364	-2.477695375	-8.139097462
	O464	-2.195624803	-6.519096050	-5.875745200
	O465	-0.384641518	-5.650805456	-6.847495827
	O466	-4.940999782	2.488785788	-7.757491348
	O467	9.378617346	-5.308112015	-4.899717601
	O468	12.227767984	-6.847648518	-3.657944467
	O469	-0.023251013	-6.455367162	-1.461382643
	O470	-0.295110710	-7.512926702	0.500501067
	O471	4.643826222	-10.907811838	-4.119213464
	O472	2.389354664	-10.961492919	-3.937365299
	O473	4.763412047	-5.926115020	-1.971615529
	O474	4.872364752	-7.683504066	-0.577918841
	O475	2.222990637	-6.181173009	-2.470315060
	O476	8.949979179	-6.602174682	-0.085599967
	O477	6.914505007	-4.665685029	-0.561760802
	O478	-4.715384271	4.803497018	-1.787138205
	O479	7.287256703	-10.370391731	-3.554723592
	O480	7.369695905	-8.654047668	-1.061575105
	S481	3.210446909	4.959510327	-2.541074184
	S482	1.615307946	1.938131067	-3.590031991
	S483	1.492876914	-3.271209718	-3.658592906
	S484	3.480872043	1.668060192	-0.480612600
	S485	0.169040165	-0.912117893	-1.814929694
	S486	3.355602065	-0.803131055	-5.752740490

	S487 3.261089118 -3.792465594 -0.510767273<br>S488 5.222354214 1.743364205 -3.615551328<br>S489 5.187686794 -3.060113593 -3.295580265<br>S490 6.239288760 -0.925970452 -1.010125634<br>end

S2BH-6H2x-7H2n (10) to 6Hx-6H2n-7H2n (13)

reactant (10)

Fe(139) -2.351<br>Fe(140) -1.293<br>Fe(141) -2.416<br>Fe(142) 2.594<br>Fe(143) 2.820<br>Fe(144) 0.504<br>Fe(145) 0.928<br><br>**S=1/2**	hd7hns2bhtm.car_7<br>C1 -7.657859162 -5.440656350 2.146443055<br>C2 -8.230663142 -6.564946541 1.277017642<br>C3 -8.181173247 -3.465632864 3.506567538<br>C4 -7.313720193 -3.729769707 4.733898609<br>C5 -6.666059068 -5.279834470 6.507436259<br>C6 -5.163068422 -5.349508218 6.226038641<br>C7 -3.381622508 -5.702845374 4.599380271<br>C8 -2.813239333 -4.287563101 4.473737273<br>C9 -3.262103064 -1.963463694 3.854156241<br>C10 -3.006199404 -1.335878628 5.232621650<br>C11 -3.747364148 -1.080234412 7.531513985<br>C12 -2.606795679 -1.699714445 8.350149769<br>C13 -1.628837218 -3.751998028 9.256126745<br>C14 -0.238619768 -4.057494841 8.682230631<br>C15 1.038207935 -4.738731229 6.690509030<br>C16 1.748383589 -3.654215476 5.876874837<br>C17 1.523013839 -1.612951057 4.502434594<br>C18 1.928148820 -0.424534400 5.406390316<br>C19 0.567174381 -1.188233550 3.365963415<br>C20 1.137115865 0.012650544 2.597633367<br>C21 0.293252157 -2.374628365 2.434140761<br>C22 1.175639153 1.289441434 7.017449881<br>C23 1.688704454 0.898690426 8.419814363<br>C24 -0.079874053 2.194817400 7.069396792<br>C25 0.104915781 3.339444650 8.073426883<br>C26 -0.399498984 2.749965620 5.675170346<br>C27 1.336759832 -0.322992498 10.490494503<br>C28 2.795197778 -0.708550072 10.631121516<br>C29 6.327032411 -11.667502103 2.925906992<br>C30 6.817276798 -10.365497942 2.293589297<br>C31 7.070781258 -7.924260696 2.736798524<br>C32 8.377307500 -7.571621380 3.473865991<br>C33 5.943701547 -6.940866768 3.099097949<br>C34 6.279515791 -5.490845886 2.745386506<br>C35 5.064702395 -4.575033330 2.898242680<br>C36 5.159573631 -2.059005733 3.026301407<br>C37 10.650456734 -6.692416756 3.230595636<br>C38 10.915599880 -5.204497010 3.197780162<br>C39 -5.591872278 -2.660144170 -0.932333434<br>C40 -5.892867468 -1.237745126 -0.419237667<br>C41 -4.059024180 -2.807934539 -1.057898706<br>C42 -3.534988349 -4.242712799 -1.274696280<br>C43 -3.434879389 -4.695395322 -2.731763235<br>C44 -6.453657620 1.029605232 -1.217986957<br>C45 -7.432389815 1.476200378 -0.123394743<br>C46 -6.765298310 1.662393916 -2.582127274<br>C47 -9.450068285 0.940907768 1.156277363<br>C48 -8.873283506 0.933876791 2.576526601<br>C49 -6.971867230 0.370846450 4.023203035<br>C50 -5.768657294 1.309343098 4.107535683<br>C51 -4.393134917 3.038798187 3.060391289<br>C52 -4.772614612 4.329791705 3.828150428<br>C53 -3.949807515 3.491692216 1.660823342<br>C54 -3.385194802 2.450524663 0.748833638<br>C55 -2.815803990 1.213766812 0.931128993<br>C56 -2.774731912 1.656408181 -1.249195084<br>C57 -6.571543438 5.966990458 4.230099500<br>C58 -6.302469752 6.073622860 5.730385930<br>C59 -8.089973905 6.039320717 3.941004299<br>C60 -8.689548282 7.414818484 3.903668483<br>C61 -8.488419736 8.509447017 4.722748368<br>C62 -9.978260648 8.981892296 3.152048207<br>C63 9.536554998 1.539230064 2.513728591<br>C64 10.543856056 0.444540347 2.213316638<br>C65 8.129222066 0.894562436 2.444269001<br>C66 7.024843511 1.867292409 2.784572596<br>C67 6.616770332 2.074214948 4.116055350<br>C68 6.371390856 2.595657996 1.775730260<br>C69 5.583426646 2.964555864 4.430025542

	C70	5.350631868	3.498983330	2.075231858
	C71	4.950215541	3.673270320	3.403330630
	C72	5.138876322	6.893904610	-1.804558517
	C73	4.067740633	7.995444354	-1.880091667
	C74	4.506507948	5.569872163	-1.382399718
	C75	2.883614036	9.599330764	-3.334306344
	C76	1.400235344	9.226380580	-3.320338695
	C77	-0.213414690	7.341005899	-3.133661631
	C78	-0.517552325	6.976965113	-1.659707652
	C79	-0.293045692	6.101881719	-4.056239783
	C80	-1.555355488	5.231674266	-4.033208729
	C81	-2.787755118	5.781339485	-4.771780699
	C82	-4.247159254	7.691364756	-4.092673939
	C83	0.228524275	5.998168254	0.453629323
	C84	0.168180367	7.214013729	1.391779256
	C85	1.278582524	5.024267785	1.021000641
	C86	1.351646948	9.252147509	2.041120591
	C87	0.687587645	10.607837862	1.839878887
	C88	-0.898092070	11.905850479	0.520782505
	C89	-2.092367487	12.456673657	1.338912645
	C90	-1.218016729	11.934796742	-0.978896071
	C91	-2.413546942	11.079340811	-1.374953467
	C92	-3.851245419	11.958889179	2.985240933
	C93	-3.649300910	13.026620542	4.051165569
	C94	-4.428091703	10.664196624	3.625462598
	C95	-4.783852160	9.612681729	2.596318404
	C96	-6.053758764	9.603397898	1.995850628
	C97	-3.849193160	8.652136427	2.173995206
	C98	-6.371749444	8.686813067	0.990096359
	C99	-4.139288368	7.742884686	1.151412669
	C100	-5.402090212	7.777868457	0.564879621
	C101	3.003542559	-3.761407919	-9.313774478
	C102	1.623849321	-3.546779265	-8.718250628
	C103	0.293668225	-2.056749966	-7.238772097
	C104	-0.229902909	-0.682058639	-7.669136421
	C105	0.208576547	1.730567042	-7.627373887
	C106	1.366370216	2.680716972	-7.918954513
	C107	3.692926223	3.325738881	-7.647650905
	C108	4.106248135	3.553008578	-9.088160811
	C109	-3.809413225	-3.293400851	-8.751112030
	C110	-4.792956865	-2.219992128	-8.273193961
	C111	-3.191138428	-4.018360392	-7.533711666
	C112	-2.527895612	-5.342401186	-7.955741752
	C113	-1.717490616	-5.912921699	-6.814782166
	C114	-5.123540887	0.091701012	-7.546000797
	C115	-4.723954061	1.384325593	-8.230557239
	C116	-5.296610711	0.199970599	-6.011028240
	C117	-3.971294724	0.169404207	-5.298716808
	C118	-3.193082181	1.333571540	-5.168439539
	C119	-3.434205329	-1.054735188	-4.867106312
	C120	-1.887366749	1.258700230	-4.673928000
	C121	-2.128442399	-1.128645602	-4.370337589
	C122	-1.343270944	0.024987073	-4.295764109
	C123	7.239690773	-6.118874248	-5.731396271
	C124	8.526935980	-6.211673950	-4.898641639
	C125	6.018835590	-6.466232430	-4.866587970
	C126	4.680849331	-6.250318880	-5.496006565
	C127	4.208866521	-6.787697289	-6.675575299
	C128	2.562187720	-5.729650412	-5.643437588
	C129	9.922434120	-7.454640456	-3.361083385
	C130	11.185876419	-7.180237540	-4.146756048
	C131	0.462187034	-7.124079936	-0.400286176
	C132	1.978537528	-7.186804319	-0.258018211
	C133	2.768516531	-7.272775811	-1.596072591
	C134	2.634293262	-8.686438774	-2.206914574
	C135	3.543120788	-8.954092214	-3.409559772
	C136	3.551083799	-10.427687827	-3.870242263
	C137	4.255855126	-6.994963967	-1.312934314
	C138	3.214529282	-0.647744261	-2.449942847
	Fe139	3.119945340	-2.041171583	-3.822628430
	Fe140	1.905439597	0.789035361	-1.764285618
	Fe141	4.757826569	0.421406443	-1.881386294
	Fe142	3.373904742	0.485888072	-4.049209283
	Fe143	3.568618793	2.721430911	-2.519859477
	Fe144	4.171907119	-2.040969616	-1.431255036
	Fe145	1.657929900	-2.847858353	-1.553827137
	H146	-6.492806704	-7.577475789	0.926904008
	H147	-6.908039528	-6.560943136	-0.280583388
	H148	-9.565215814	-4.771571801	2.541495034
	H149	-9.070996481	-6.165606145	0.686327704

	H150	-8.672054488	-7.309961869	1.961106897
	H151	-7.607740273	-2.783728785	2.862147883
	H152	-8.144140045	-5.584609939	4.974151169
	H153	-9.088569258	-2.940502757	3.834107953
	H154	-7.001984544	-6.258403936	6.874521982
	H155	-5.491168550	-5.858109835	4.249095614
	H156	-6.800164005	-4.549628845	7.317081150
	H157	-2.342579924	-1.839808519	3.270708030
	H158	-4.581042953	-3.636759287	3.661145027
	H159	-3.266415164	-6.222739866	3.638482458
	H160	-3.548758636	-0.003776338	7.458186648
	H161	-4.734491158	-2.182405738	5.966563184
	H162	-2.777963920	-6.228564073	5.349563073
	H163	-4.071442316	-1.424070245	3.342946043
	H164	-3.275789590	-3.603739322	7.894307163
	H165	-4.692178973	-1.228616216	8.071857168
	H166	-0.142559757	-3.227294595	2.971091295
	H167	-0.414582508	-2.090519920	1.639852227
	H168	1.222878743	-2.715544754	1.951124197
	H169	-0.381292153	-0.873404847	3.834925982
	H170	-2.083987291	-4.707509395	9.555415566
	H171	-0.582499953	1.949588063	4.944518635
	H172	0.064757068	-0.359586702	8.786285372
	H173	0.257770596	2.976029676	9.099779042
	H174	2.079142412	-0.246575004	2.091768623
	H175	1.332222269	0.872556439	3.252540861
	H176	9.706903038	1.869289210	3.556716879
	H177	-1.297955268	3.383671189	5.714015834
	H178	-0.786530711	3.983679426	8.071543021
	H179	0.435859483	3.369235654	5.308652159
	H180	0.972697319	3.960860875	7.800134714
	H181	-0.930792695	1.574377065	7.404561642
	H182	2.005920858	1.855177856	6.571726059
	H183	-0.015644280	-0.221631575	6.058992044
	H184	0.423590701	0.351610470	1.833547827
	H185	1.496116668	4.252979079	0.267250579
	H186	0.822259242	4.540253561	1.896559875
	H187	2.862994471	5.260389204	2.166525479
	H188	3.714204159	4.583097535	4.594536743
	H189	4.844273844	4.046111654	1.280372649
	H190	6.643287530	2.432561290	0.729884727
	H191	7.100045090	1.516877958	4.922662472
	H192	-0.050277904	-2.815237158	5.346698785
	H193	0.834639385	-5.581222340	6.011520036
	H194	1.749735074	-5.077001357	7.452722199
	H195	-1.043697312	-4.317399877	6.790762732
	H196	-1.465838044	-3.151526163	10.159370064
	H197	5.262602112	3.092556033	5.467444159
	H198	2.453959004	-1.976016336	4.054893233
	H199	1.122200932	0.510097084	11.180567265
	H200	0.738400240	-1.191581269	10.808256605
	H201	3.255699684	-1.174026026	9.728752843
	H202	11.467012643	-7.221105394	2.715951033
	H203	-2.354213458	-5.919669598	-3.970580527
	H204	0.884513404	-6.277157194	-2.074079370
	H205	-3.308797805	-6.069211705	-8.220611579
	H206	-1.887788048	-5.182845001	-8.835142433
	H207	-3.967531791	-4.229089130	-6.782626336
	H208	-4.264861899	1.244136740	-9.237111251
	H209	-4.454798062	-4.020949317	-9.267337732
	H210	-3.714175156	-2.159448796	-1.880194059
	H211	-4.183221042	-4.969717604	-0.755244334
	H212	-3.260491720	-0.834305515	-8.079196578
	H213	-6.124893933	-0.133052643	-7.967991229
	H214	-2.133196749	-2.201889464	-9.321582957
	H215	-1.747711544	-5.859384926	-2.324219017
	H216	-2.459909394	-3.350760788	-7.051340238
	H217	-2.420359401	-3.539665879	-10.240930004
	H218	-7.147717956	-3.394862182	-2.077879074
	H219	-3.617552916	-2.424478596	-0.125858031
	H220	-0.108880906	-5.159440197	-7.656844475
	H221	-2.546621073	-4.340024287	-0.800723156
	H222	-3.554486227	2.598484401	3.618290091
	H223	-2.641055085	0.640421541	1.832172463
	H224	-7.690286028	0.705316856	4.785058661
	H225	-7.173796972	-0.090067574	1.951296265
	H226	-6.737635151	4.041564058	3.304477045
	H227	-7.837216413	8.676877609	5.573222434
	H228	-6.359862688	5.095931308	6.268210988
	H229	-8.624734111	5.395992804	4.662305249

	H230	-6.047766371	6.791516159	3.720953529
	H231	-10.698102053	9.558428627	2.576874576
	H232	-3.193768023	4.279697972	1.805166459
	H233	-4.808148356	3.961645023	1.156999690
	H234	-9.408968147	10.436155244	4.598530691
	H235	-8.260567434	5.597945588	2.946192857
	H236	-6.077723302	2.028401447	2.176492272
	H237	-6.636559984	-0.634767093	4.319328482
	H238	-9.912681235	1.928032392	1.016526188
	H239	-2.584006145	1.561546936	-2.315604123
	H240	-10.247668763	0.188532142	1.103590304
	H241	10.508458105	3.155757012	1.713333152
	H242	8.881210521	3.241114918	1.585037597
	H243	1.319427262	6.002664882	-1.410427087
	H244	-0.756554539	5.510909244	0.517357631
	H245	1.688897840	8.075135388	0.321728698
	H246	-3.753047978	3.546113096	-1.079446767
	H247	-5.492155801	4.235636192	-2.045708929
	H248	-7.229073329	3.451822196	-3.237792723
	H249	-5.054561812	5.458418447	-1.122017937
	H250	-5.999520027	1.324168848	-3.299688562
	H251	-7.753017584	1.326693510	-2.932797642
	H252	-0.319801331	11.667270678	-1.552354804
	H253	-1.475411798	12.975154521	-1.232068050
	H254	-1.316337957	10.228731971	-2.913966603
	H255	-2.983210547	9.691932926	-2.718221880
	H256	-4.353056403	9.327111698	-5.331971919
	H257	-3.334491504	4.957896022	-5.255613760
	H258	-7.367447724	8.685769831	0.539576716
	H259	-2.865522610	8.590922557	2.647430548
	H260	-5.037093126	7.839783002	-2.193825562
	H261	0.557267757	5.448529938	-3.796975748
	H262	1.913621312	7.254028380	-3.282919479
	H263	-1.828263871	4.956711308	-3.002032184
	H264	-8.567943326	-0.191672338	-0.441882143
	H265	3.103012355	10.068695993	-4.302306928
	H266	-6.493864991	-0.900160517	-2.287885048
	H267	-0.109968626	6.446798318	-5.087491179
	H268	-3.374748852	7.049354276	0.801157258
	H269	3.017836873	10.358909651	-2.551313741
	H270	-3.719841908	7.893568895	-6.078904789
	H271	-5.590563002	9.032604556	-3.324056128
	H272	-5.657664536	-3.450929705	-2.834537477
	H273	-5.932160522	-3.343149784	-0.132229905
	H274	-6.812969884	10.319153910	2.318356820
	H275	-5.481248752	1.407566361	-0.872600968
	H276	-0.945944841	8.101321202	-3.420090198
	H277	-2.458692875	6.457069989	-5.570943621
	H278	-1.280309419	4.292362100	-4.533640405
	H279	-4.062442955	5.940915595	-3.070662902
	H280	1.084051463	8.898573128	3.049842509
	H281	-0.533360135	9.787933864	0.422439287
	H282	-0.104013885	12.648295576	0.696769559
	H283	2.438307929	9.422908394	2.022094314
	H284	-2.261020669	10.689485265	2.320143486
	H285	3.484746205	-2.788795723	-9.493601787
	H286	3.596840218	-4.270138941	-8.525802640
	H287	7.173604955	-5.065086146	-6.039976100
	H288	3.837438757	-4.846519669	-10.825445882
	H289	2.328573767	-5.334447502	-10.417301542
	H290	5.309363722	4.826955152	-1.300491405
	H291	5.760087139	7.498295605	0.044721450
	H292	4.338364707	8.144926632	-3.914389125
	H293	4.015460713	5.684608874	-0.406276502
	H294	6.688680470	8.085381897	-1.167388248
	H295	4.568557639	2.940259207	-7.102608080
	H296	5.603814318	6.762984388	-2.794594262
	H297	5.723765530	-7.033616838	4.175634081
	H298	7.235997189	-7.901484511	1.652350284
	H299	2.805605449	1.503707275	-6.976602217
	H300	5.362116068	-0.034345839	2.833362024
	H301	-0.422524971	1.699465244	-8.524271249
	H302	-1.713849393	-2.090402805	-4.059724220
	H303	-0.393653471	2.213121236	-6.838550829
	H304	4.748793561	-4.522549806	3.946541008
	H305	1.275554115	0.156078208	-6.580775529
	H306	4.214839594	-4.979915963	2.323913759
	H307	5.047081286	-7.262789214	2.545976474
	H308	6.621884114	-5.435724135	1.701270308
	H309	7.098554938	-5.114001629	3.378624072

	H310	-5.925467884	-0.642055902	-5.687591983
	H311	6.344402080	-9.490564465	4.029689508
	H312	5.364105402	-11.894686703	2.443235075
	H313	-0.310501929	-0.018469083	-3.950426015
	H314	6.985607742	-11.806547351	4.871419145
	H315	5.421345448	-12.248808915	4.678599300
	H316	5.650333365	-3.180264677	1.418136628
	H317	-1.269342029	2.155138364	-4.593249768
	H318	-4.035391456	-1.964393195	-4.932707080
	H319	7.304533861	-9.873929127	-2.697804797
	H320	6.081915152	-7.527624381	-4.570520938
	H321	6.481451771	-8.217702059	-0.955772632
	H322	6.339292272	-10.496272426	-3.756028646
	H323	3.787878122	2.754932483	-9.802353186
	H324	6.200414742	-5.156612359	-1.061728255
	H325	2.246269258	-8.019689771	0.405330302
	H326	2.324862917	-1.873748188	-7.779985071
	H327	2.872648171	-9.402608497	-1.404561847
	H328	2.220916637	-6.743423847	-7.462856732
	H329	1.563120943	-5.368174650	-5.438658590
	H330	7.032458574	-12.455676967	2.612356910
	H331	5.671987431	-0.915987453	1.358516472
	H332	8.350715157	-5.820838552	-0.258816828
	H333	6.972348000	-3.808770645	-1.034901645
	H334	9.188190782	-6.916423504	1.667508369
	H335	4.453805306	-2.806767118	4.805211521
	H336	3.212717601	-8.337175723	-4.260286994
	H337	4.576334062	-8.659195845	-3.178779457
	H338	1.581711216	-8.853744915	-2.484806644
	H339	0.524862222	-2.063795684	-6.161290576
	H340	-3.608729748	2.290222552	-5.494495936
	H341	-5.842639374	1.135920832	-5.817215308
	H342	2.265414779	-6.254251595	0.259035386
	H343	8.459462473	-7.350942451	-0.490154233
	H344	7.408233680	-9.270823693	-0.315527128
	H345	4.417741146	-1.116152707	4.676847116
	H346	4.702789882	-7.389260140	-7.429111340
	H347	6.065785776	-5.890252381	-3.935030161
	H348	-0.480721132	-2.797814434	-7.447720693
	H349	3.419563873	4.291808247	-7.195887725
	H350	8.115425338	0.044871892	3.142979160
	H351	7.975828955	0.495904289	1.428592227
	H352	8.112605613	-6.638777495	-7.513259979
	H353	7.465392661	-7.916090713	-6.719280662
	H354	9.987235839	-8.490196200	-2.992733023
	H355	8.067425895	-8.128354300	-4.170408965
	H356	11.120992676	-7.420437897	-5.235837154
	H357	9.879616381	-6.790541758	-2.482968916
	H358	10.675521168	0.229021220	1.125554047
	H359	-2.588931601	13.201873761	4.351291576
	H360	10.661305357	-7.000114435	4.291434596
	H361	10.004533406	-4.559203741	3.230071541
	H362	-4.576750496	12.355732192	2.255946640
	H363	-3.691810514	10.263474564	4.339688826
	H364	-5.318105014	10.954529293	4.200899715
	H365	0.450679544	-3.859053715	-1.216219370
	H366	3.959183981	-1.335990056	0.073622975
	H367	-6.639246647	6.882951165	-0.644510571
	H368	0.346272128	-0.848027192	0.034106226
	H369	3.190719276	-1.375364508	-0.227611483
	H370	0.683367560	-3.556300465	-0.450218237
	H371	-1.899728638	-0.091599673	-0.534601681
	Mo372	3.335681246	-4.506594578	-2.735312342
	N373	-7.291991365	-7.231085608	0.389963827
	N374	-8.582256175	-4.659070385	2.778269637
	N375	-7.471382154	-4.921883957	5.352115366
	N376	-4.786555081	-5.721640871	4.976136471
	N377	-3.633755914	-3.367406010	3.915742088
	N378	-3.892224533	-1.653879953	6.203219977
	N379	-2.560163920	-3.055857795	8.380602191
	N380	-0.191380156	-4.342457185	7.352081755
	N381	0.963966976	-2.713418361	5.292291324
	N382	0.942135035	0.123452982	6.159854731
	N383	0.969730688	-0.016088576	9.117593111
	N384	6.122209774	-11.570077931	4.377159254
	N385	6.677120428	-9.281599953	3.082882460
	N386	5.347757677	-3.227102800	2.393340876
	N387	4.856763509	-1.988683724	4.336823781
	N388	5.290268564	-0.913490437	2.325638461
	N389	9.355817784	-7.055872443	2.688736756

	N390	-6.269380180	-2.890915126	-2.213514754
	N391	-2.443623961	-5.562147919	-3.013288225
	N392	-6.378508059	-0.411608465	-1.383169952
	N393	-8.497724592	0.671605560	0.092149961
	N394	-7.664830977	0.327228007	2.749139323
	N395	-5.476365603	2.066059414	3.004792555
	N396	-3.345917332	2.690225823	-0.618144109
	N397	-2.460349920	0.745622832	-0.320468728
	N398	-6.080638953	4.682348604	3.742837666
	N399	-9.622594277	7.722924983	2.926693085
	N400	-9.315980077	9.494564073	4.226815356
	N401	9.674920167	2.598308635	1.511717962
	N402	6.200331540	7.244500787	-0.845936571
	N403	3.814160852	8.505238127	-3.121414075
	N404	1.121936658	7.906265488	-3.292975706
	N405	-3.724689451	6.482745511	-3.886052464
	N406	-4.073780886	8.354994782	-5.248755632
	N407	-4.910844120	8.313695429	-3.091348028
	N408	0.471736156	6.364439169	-0.944390994
	N409	1.006258476	8.236231189	1.066425168
	N410	-0.361290126	10.633128872	0.965735747
	N411	-2.258598388	10.382687153	-2.533464020
	N412	-2.597408231	11.649021359	2.304976305
	N413	2.907706271	-4.504563171	-10.573132101
	N414	1.484074032	-2.418822075	-7.993071075
	N415	0.529440488	0.361901872	-7.263825448
	N416	2.618355624	2.354087277	-7.510610020
	N417	-2.875281575	-2.766299047	-9.751999508
	N418	-4.269088696	-0.992551080	-7.993522412
	N419	7.303149920	-6.933089217	-6.959072103
	N420	3.635027982	-5.573250876	-4.865645039
	N421	2.876591217	-6.449443445	-6.744504730
	N422	8.737155589	-7.354658275	-4.192623820
	N423	-0.049651440	2.539299648	-0.388693045
	N424	0.697641121	1.889758236	-0.933748353
	O425	-6.438237328	-5.261214745	2.311804540
	O426	-6.507302524	-2.868467051	5.131001821
	O427	-4.343991440	-5.090851843	7.123670979
	O428	-1.662812923	-4.007850309	4.860928506
	O429	-2.036152753	-0.582858290	5.421514783
	O430	-1.809821724	-0.980788913	8.978091815
	O431	0.744602423	-4.088288524	9.433518205
	O432	2.986205256	-3.698443697	5.755901627
	O433	3.097927881	0.006774337	5.414030056
	O434	2.713702752	1.421633846	8.889415799
	O435	3.412548998	-0.592209162	11.679973617
	O436	7.268610060	-10.368873649	1.129985146
	O437	8.486541829	-7.757723429	4.698693931
	O438	12.035293669	-4.718356292	3.156341014
	O439	-5.690679807	-0.878067021	0.757055805
	O440	-4.240500442	-4.301196439	-3.605542953
	O441	-7.219847499	2.510982869	0.533675960
	O442	-6.751512838	3.098470829	-2.466344686
	O443	-9.519050587	1.457250013	3.493625668
	O444	-5.105288575	1.364661726	5.148725511
	O445	-3.917064922	5.020517968	4.389999103
	O446	-6.087648025	7.118551224	6.319057032
	O447	11.157160561	-0.180725340	3.065979945
	O448	3.907590039	4.550139794	3.639758993
	O449	3.471002581	8.410044904	-0.870511938
	O450	0.540629148	10.128463037	-3.345900675
	O451	-1.627822848	7.240227783	-1.171999378
	O452	-0.553556071	7.203846869	2.397141817
	O453	2.476364862	5.713910674	1.390379407
	O454	1.083826282	11.602937560	2.459276818
	O455	-2.507147602	13.607449401	1.140500599
	O456	-3.464565494	11.068406006	-0.718517330
	O457	-4.575118580	13.635114231	4.564158801
	O458	-5.672951262	6.892228860	-0.500091277
	O459	0.707619822	-4.389077943	-8.883658932
	O460	-1.259964868	-0.556458331	-8.362312073
	O461	1.120615075	3.759024000	-8.484562844
	O462	4.765919834	4.513092912	-9.449642038
	O463	-6.001188677	-2.469664686	-8.126957813
	O464	-2.214432822	-6.524286903	-5.873889354
	O465	-0.391841268	-5.671557435	-6.837121349
	O466	-4.918886781	2.501106994	-7.773157017
	O467	9.373481264	-5.296255717	-4.896683995
	O468	12.233181433	-6.802390174	-3.640536269
	O469	0.012803153	-6.489538634	-1.497447366

	O470 -0.305132415 -7.560785094 0.452431272<br>O471 4.691816119 -10.953004308 -4.122927211<br>O472 2.435057185 -11.007618313 -3.993061816<br>O473 4.782666931 -5.976567857 -1.930610849<br>O474 4.887188639 -7.742040834 -0.540150973<br>O475 2.253074190 -6.270641788 -2.479275983<br>O476 8.953444836 -6.585222915 -0.083659755<br>O477 6.905318480 -4.664267351 -0.568848074<br>O478 -4.719174314 4.805471070 -1.785646238<br>O479 7.312820462 -10.337813173 -3.564830407<br>O480 7.382670822 -8.629703348 -1.068991817<br>S481 3.246992364 4.985723302 -2.609348780<br>S482 1.732207001 1.998541502 -3.676172323<br>S483 1.393666634 -3.472408959 -3.659768713<br>S484 3.578296869 1.778785980 -0.511327732<br>S485 0.370774391 -0.943582531 -1.323281465<br>S486 3.251011225 -0.909049032 -5.726057420<br>S487 3.271338556 -3.858563752 -0.464484627<br>S488 5.279706057 1.672001936 -3.636931558<br>S489 5.081582369 -3.014643421 -3.286394153<br>S490 6.124675876 -0.988983202 -0.891777141<br>end

TS

Fe(139) -2.201<br>Fe(140) -1.246<br>Fe(141) -2.494<br>Fe(142) 2.527<br>Fe(143) 2.834<br>Fe(144) 0.980<br>Fe(145) 0.349<br><br>**S=1/2**	6hx7h2n2bhto6hnte_1_53147.63<br>C1 -7.672231178 -5.443611303 2.143398350<br>C2 -8.253615632 -6.561984464 1.272066335<br>C3 -8.177117714 -3.462851088 3.500027020<br>C4 -7.311105254 -3.728830963 4.727758751<br>C5 -6.665227486 -5.279720487 6.500803471<br>C6 -5.161616529 -5.346461446 6.221868506<br>C7 -3.374744136 -5.696767542 4.600660319<br>C8 -2.808018918 -4.280447677 4.476818974<br>C9 -3.259774175 -1.958868666 3.848192763<br>C10 -3.010461606 -1.324740843 5.224595425<br>C11 -3.747076549 -1.073777188 7.524135803<br>C12 -2.604543680 -1.693116585 8.340297341<br>C13 -1.626807182 -3.747091735 9.244060745<br>C14 -0.234017889 -4.053815000 8.676631257<br>C15 1.052287261 -4.742582303 6.693013994<br>C16 1.761665108 -3.660872916 5.874499337<br>C17 1.529149537 -1.616641470 4.501970647<br>C18 1.937996725 -0.431728109 5.406734320<br>C19 0.553989607 -1.189064257 3.382895436<br>C20 1.081784937 0.027243943 2.612598698<br>C21 0.279783513 -2.363489896 2.438268802<br>C22 1.184168452 1.287857491 7.012669872<br>C23 1.695756361 0.896786554 8.414905005<br>C24 -0.072107048 2.192305201 7.067129647<br>C25 0.113165350 3.334423625 8.074112426<br>C26 -0.394245241 2.750809273 5.675037802<br>C27 1.337768503 -0.323719003 10.485331677<br>C28 2.800137723 -0.688066601 10.635746735<br>C29 6.319714895 -11.673666280 2.944135065<br>C30 6.805867155 -10.372749146 2.306643136<br>C31 7.065727218 -7.931888301 2.745283101<br>C32 8.372592790 -7.575725349 3.479723146<br>C33 5.937580475 -6.949423280 3.105519222<br>C34 6.270772098 -5.500955434 2.745494965<br>C35 5.050993059 -4.589868753 2.880030881<br>C36 5.151416752 -2.072765321 2.994114105<br>C37 10.645801660 -6.699791381 3.230829976<br>C38 10.917935871 -5.213328714 3.202010413<br>C39 -5.599726860 -2.672338884 -0.924494037<br>C40 -5.904177431 -1.252561339 -0.407757193<br>C41 -4.065995548 -2.815890167 -1.048861227<br>C42 -3.537356775 -4.248503698 -1.266710516<br>C43 -3.438670259 -4.700663053 -2.723499590<br>C44 -6.457635557 1.018847791 -1.204274183<br>C45 -7.438107453 1.463573417 -0.111004401<br>C46 -6.766130453 1.654922299 -2.567800264<br>C47 -9.452602744 0.918715534 1.170369296<br>C48 -8.873468291 0.918355007 2.589714263<br>C49 -6.969638094 0.369469677 4.037599369<br>C50 -5.772058508 1.314587443 4.121336904<br>C51 -4.391598135 3.033539989 3.064764474<br>C52 -4.768142200 4.321817982 3.837255011<br>C53 -3.959000549 3.485283438 1.662451780<br>C54 -3.436446838 2.432186711 0.739249058

	C55	-2.913454815	1.174246901	0.913938830
	C56	-2.818696909	1.641193813	-1.256904228
	C57	-6.569189567	5.962927486	4.226029258
	C58	-6.314165629	6.070339126	5.728748327
	C59	-8.084613318	6.030650029	3.923070070
	C60	-8.691775417	7.402720388	3.887772451
	C61	-8.491931681	8.499004882	4.704840306
	C62	-9.995468325	8.961116339	3.144064379
	C63	9.519010917	1.523380394	2.517506209
	C64	10.525434664	0.427114677	2.220000217
	C65	8.110956953	0.881256066	2.449472794
	C66	7.008955222	1.856148351	2.791921115
	C67	6.604202632	2.063320937	4.124408696
	C68	6.357114476	2.588980177	1.784902383
	C69	5.576646619	2.959203319	4.441166107
	C70	5.340955499	3.496727091	2.087100611
	C71	4.945151265	3.673042918	3.416642821
	C72	5.136611291	6.871647055	-1.786935906
	C73	4.066361632	7.973803239	-1.868550694
	C74	4.504400278	5.551852853	-1.349099016
	C75	2.892082572	9.580060915	-3.328233576
	C76	1.407286869	9.210690427	-3.310720748
	C77	-0.210749448	7.328354072	-3.128799292
	C78	-0.521957286	6.966686386	-1.654629519
	C79	-0.287990262	6.088975414	-4.051454991
	C80	-1.552310760	5.222372587	-4.033357760
	C81	-2.781420752	5.776393505	-4.774153955
	C82	-4.241035941	7.687559599	-4.091949254
	C83	0.219466131	5.992466022	0.462496626
	C84	0.176842106	7.209343980	1.398495348
	C85	1.262004657	5.005938655	1.024388795
	C86	1.369097826	9.251705053	2.030486443
	C87	0.697509100	10.601625024	1.828171829
	C88	-0.897552412	11.891813925	0.508707980
	C89	-2.089202845	12.442137864	1.333666280
	C90	-1.226687979	11.918414649	-0.986324866
	C91	-2.417505921	11.051381172	-1.372925739
	C92	-3.846417215	11.947473971	2.980397575
	C93	-3.641017654	13.015480095	4.045272142
	C94	-4.425528687	10.653875282	3.622604166
	C95	-4.786043205	9.602662763	2.595446142
	C96	-6.057863588	9.594770671	1.999336785
	C97	-3.854184735	8.640660949	2.170562992
	C98	-6.380825999	8.678168421	0.994899952
	C99	-4.149130801	7.731729886	1.148977709
	C100	-5.413911465	7.768043033	0.566038470
	C101	3.003538317	-3.754317958	-9.331722874
	C102	1.629431303	-3.537402466	-8.724574376
	C103	0.310031056	-2.054930806	-7.228236277
	C104	-0.224184516	-0.686546005	-7.665533938
	C105	0.202830003	1.729862332	-7.636471743
	C106	1.353785529	2.691634483	-7.914175738
	C107	3.680995839	3.338863075	-7.629729872
	C108	4.135175341	3.531995593	-9.062625337
	C109	-3.826412160	-3.303366171	-8.751710636
	C110	-4.811166295	-2.231672592	-8.271043882
	C111	-3.199969019	-4.023515078	-7.535162254
	C112	-2.529066865	-5.344411812	-7.956125699
	C113	-1.710810076	-5.906519620	-6.816620569
	C114	-5.142976910	0.076962074	-7.534189009
	C115	-4.737537051	1.371351214	-8.211557326
	C116	-5.332272465	0.182356043	-6.000600724
	C117	-4.007063433	0.164107754	-5.292261215
	C118	-3.248305193	1.339123592	-5.158250607
	C119	-3.438853375	-1.060985715	-4.898094672
	C120	-1.922951434	1.275562033	-4.716952620
	C121	-2.114835648	-1.121868785	-4.458144749
	C122	-1.342330527	0.042552347	-4.404068283
	C123	7.231820262	-6.108941121	-5.721844699
	C124	8.521719634	-6.215980526	-4.894678882
	C125	6.012665999	-6.485328803	-4.867532105
	C126	4.671921680	-6.247117675	-5.484210933
	C127	4.179638934	-6.785545497	-6.654915938
	C128	2.556630335	-5.698753243	-5.614410617
	C129	9.913079740	-7.474095498	-3.363973888
	C130	11.175928761	-7.211250619	-4.154679023
	C131	0.457999611	-7.063826272	-0.367859552
	C132	1.974861712	-7.117161277	-0.246818014
	C133	2.750131969	-7.207315802	-1.593238257
	C134	2.601836650	-8.620106784	-2.206694937

C135	3.512647272	-8.905408484	-3.403494934
C136	3.512239532	-10.385172905	-3.847319206
C137	4.242840516	-6.954271647	-1.322796051
C138	3.205696893	-0.666252334	-2.456071432
Fe139	3.273204480	-1.989243157	-3.874436178
Fe140	1.827607219	0.723227897	-1.771310604
Fe141	4.764686474	0.440069233	-1.905691761
Fe142	3.340777643	0.520725265	-4.035186169
Fe143	3.507421763	2.700275655	-2.472029381
Fe144	4.300631993	-2.046518569	-1.487720264
Fe145	1.759283634	-2.623721283	-1.567281331
H146	-6.522294031	-7.584234332	0.920804671
H147	-6.928995723	-6.562133539	-0.283664015
H148	-9.573285242	-4.759556196	2.539451287
H149	-9.090731007	-6.154778320	0.682438631
H150	-8.700112708	-7.305591829	1.954142270
H151	-7.597196108	-2.789621157	2.851982631
H152	-8.142438993	-5.583351201	4.966214390
H153	-9.079064024	-2.927255215	3.825889812
H154	-7.000304869	-6.259287050	6.866057427
H155	-5.483732381	-5.857349301	4.244540276
H156	-6.801168783	-4.550828723	7.311489237
H157	-2.338886414	-1.836290020	3.266706927
H158	-4.573045389	-3.636266885	3.654137880
H159	-3.255317850	-6.215864187	3.639901927
H160	-3.548599695	0.002521194	7.449589966
H161	-4.731257025	-2.184571025	5.962507557
H162	-2.772946898	-6.221925928	5.352673670
H163	-4.069513470	-1.424771043	3.332151667
H164	-3.269918391	-3.596932441	7.878764658
H165	-4.690695000	-1.221999770	8.066629034
H166	-0.165339227	-3.223772306	2.955731150
H167	-0.405032293	-2.056971568	1.633419399
H168	1.212590138	-2.702620506	1.960963113
H169	-0.389805537	-0.892604829	3.871145237
H170	-2.084072561	-4.702619844	9.540290987
H171	-0.577464111	1.952834109	4.942023063
H172	0.071856391	-0.362216242	8.776781781
H173	0.266767063	2.968408721	9.099455662
H174	1.984272878	-0.221041675	2.032948512
H175	1.326256701	0.871583395	3.273584071
H176	9.689758965	1.854849933	3.559759618
H177	-1.293498700	3.383240765	5.717037520
H178	-0.778332582	3.978619254	8.074669953
H179	0.439722949	3.372313592	5.309087555
H180	0.980880933	3.956534705	7.801957154
H181	-0.922122788	1.570554275	7.401952104
H182	2.013824910	1.854502024	6.566999144
H183	-0.007646897	-0.217511226	6.048362132
H184	0.320204343	0.376987583	1.901436049
H185	1.463520964	4.230750370	0.270819687
H186	0.805342392	4.527969037	1.904664158
H187	2.841844909	5.243151422	2.178367443
H188	3.720032760	4.590666977	4.612206169
H189	4.835846034	4.048080372	1.294374183
H190	6.628022659	2.427312437	0.738459608
H191	7.085879544	1.502456406	4.929529999
H192	-0.037529119	-2.825815178	5.343900114
H193	0.852755399	-5.589750626	6.018710401
H194	1.761859196	-5.075082258	7.459524681
H195	-1.029932111	-4.317763189	6.781644027
H196	-1.466902146	-3.148041713	10.148858548
H197	5.258624761	3.087771102	5.479511590
H198	2.454713308	-1.976520753	4.041305002
H199	1.105576360	0.504816100	11.175072033
H200	0.750680614	-1.201904453	10.796531482
H201	3.270909240	-1.154422636	9.739573284
H202	11.459594504	-7.230322019	2.713449930
H203	-2.346814822	-5.915719806	-3.961888150
H204	0.832911995	-6.184685524	-2.030229487
H205	-3.305966958	-6.077246944	-8.216072328
H206	-1.893399533	-5.182784783	-8.838403317
H207	-3.971855780	-4.237830683	-6.780312657
H208	-4.268081780	1.233773367	-9.213895568
H209	-4.469906949	-4.033247729	-9.266686047
H210	-3.719674514	-2.165434996	-1.868660590
H211	-4.183011517	-4.977553049	-0.746540867
H212	-3.278414779	-0.849016182	-8.059762280
H213	-6.140559072	-0.144738864	-7.966777032
H214	-2.151962327	-2.209649335	-9.319685041

	H215	-1.733411724	-5.831808548	-2.317883221
	H216	-2.471158370	-3.350361783	-7.057174755
	H217	-2.439641982	-3.541961882	-10.245264253
	H218	-7.153532233	-3.403277355	-2.074155002
	H219	-3.625179129	-2.434251627	-0.115840265
	H220	-0.109612474	-5.143183111	-7.665583570
	H221	-2.546697877	-4.340801981	-0.797002802
	H222	-3.548810032	2.591888506	3.614235629
	H223	-2.788102857	0.579148632	1.808388135
	H224	-7.690608885	0.701281035	4.797866548
	H225	-7.167964442	-0.098034027	1.965744721
	H226	-6.727803011	4.041365849	3.293945559
	H227	-7.836511369	8.669880085	5.551468880
	H228	-6.382432577	5.094184194	6.267971523
	H229	-8.622818673	5.381523517	4.636562084
	H230	-6.041595634	6.787815529	3.721324333
	H231	-10.722529282	9.532994843	2.573140039
	H232	-3.177507832	4.250065564	1.800140207
	H233	-4.811603731	3.983266925	1.173049907
	H234	-9.425637368	10.419780884	4.585894653
	H235	-8.244647447	5.592409738	2.925181591
	H236	-6.075888218	2.019702133	2.183950830
	H237	-6.628335329	-0.633391937	4.335269161
	H238	-9.922632333	1.902133094	1.030176829
	H239	-2.620360419	1.553373088	-2.322418290
	H240	-10.244585460	0.160300114	1.120042391
	H241	10.497179358	3.132932948	1.711753969
	H242	8.870204946	3.228975216	1.591434040
	H243	1.314558859	5.992601413	-1.398826439
	H244	-0.770006476	5.517191219	0.530681543
	H245	1.697844911	8.060938121	0.316394177
	H246	-3.749538824	3.555264692	-1.080864002
	H247	-5.482193989	4.229991223	-2.044202385
	H248	-7.231085540	3.448131096	-3.214114177
	H249	-5.056654821	5.459513544	-1.125868593
	H250	-6.002055254	1.313919611	-3.285858159
	H251	-7.755833328	1.325871752	-2.918974447
	H252	-0.330937438	11.658180916	-1.566562945
	H253	-1.496015038	12.956361174	-1.236763503
	H254	-1.311970742	10.190601817	-2.898780097
	H255	-2.972596641	9.636206157	-2.692997578
	H256	-4.304492674	9.342141340	-5.307252029
	H257	-3.330300621	4.955010305	-5.259092991
	H258	-7.378335618	8.677907871	0.548254918
	H259	-2.869154475	8.577944840	2.640712255
	H260	-5.048265428	7.819053650	-2.198626054
	H261	0.559234055	5.434365678	-3.787768465
	H262	1.915849334	7.237529939	-3.276382838
	H263	-1.828726989	4.946299825	-3.003216429
	H264	-8.565281726	-0.209806239	-0.427755807
	H265	3.111555303	10.043432079	-4.299283403
	H266	-6.491879582	-0.908224776	-2.278727662
	H267	-0.099573356	6.433678903	-5.081349196
	H268	-3.386616179	7.036649885	0.796662463
	H269	3.029428927	10.344428408	-2.550417767
	H270	-3.654044815	7.919051821	-6.057325260
	H271	-5.585061549	9.025687091	-3.320526118
	H272	-5.661501145	-3.461725969	-2.826789689
	H273	-5.938641996	-3.358722474	-0.126724741
	H274	-6.815389593	10.311414152	2.324138069
	H275	-5.486038785	1.396273718	-0.857210030
	H276	-0.941270896	8.088579420	-3.420166160
	H277	-2.449348815	6.450557902	-5.573607071
	H278	-1.278384122	4.283427587	-4.534313236
	H279	-4.056155791	5.936150912	-3.074519921
	H280	1.114021850	8.902979373	3.042048792
	H281	-0.522599473	9.775043082	0.412151347
	H282	-0.105112935	12.637267300	0.679734075
	H283	2.453377011	9.425848388	1.997032441
	H284	-2.260433215	10.673932222	2.312197235
	H285	3.488345292	-2.783202450	-9.509652076
	H286	3.600708876	-4.270523467	-8.551674955
	H287	7.159134808	-5.048690112	-6.006538060
	H288	3.820107477	-4.833642731	-10.856947962
	H289	2.313318461	-5.318714264	-10.437484630
	H290	5.303492645	4.803409522	-1.274053516
	H291	5.768808830	7.490154506	0.055507143
	H292	4.346032733	8.122100284	-3.902220735
	H293	4.026398713	5.674936478	-0.366691098
	H294	6.687437056	8.069904170	-1.167372282

	H295	4.541664366	2.970379081	-7.050078996
	H296	5.598833790	6.732225738	-2.776846327
	H297	5.719933034	-7.037521963	4.182807535
	H298	7.230330010	-7.912620154	1.660542655
	H299	2.793215854	1.516006784	-6.969235540
	H300	5.370801804	-0.048953468	2.786406080
	H301	-0.419605900	1.695835292	-8.539173645
	H302	-1.674684118	-2.078109140	-4.164979851
	H303	-0.411467773	2.202392730	-6.850874918
	H304	4.723215021	-4.529742749	3.923904469
	H305	1.279947531	0.167564204	-6.589477655
	H306	4.207366449	-5.000563435	2.300683010
	H307	5.040214065	-7.275294472	2.555894958
	H308	6.623729318	-5.451011256	1.704652898
	H309	7.082097824	-5.115870892	3.383439511
	H310	-5.957028141	-0.664648479	-5.682034597
	H311	6.346986939	-9.495593760	4.045556508
	H312	5.355748597	-11.903422263	2.464893954
	H313	-0.298475803	0.002320229	-4.096784690
	H314	6.983450380	-11.812062582	4.888160991
	H315	5.416354299	-12.246020085	4.700869872
	H316	5.662191583	-3.208382812	1.400695141
	H317	-1.317907331	2.180465957	-4.632634814
	H318	-4.030696653	-1.977270741	-4.954518361
	H319	7.289236254	-9.900490261	-2.687541032
	H320	6.072122917	-7.558182804	-4.614137777
	H321	6.475297422	-8.224691904	-0.960961026
	H322	6.311568590	-10.498971188	-3.745709189
	H323	3.819268601	2.728483002	-9.770985381
	H324	6.219763520	-5.143398877	-1.071131195
	H325	2.252697740	-7.948000527	0.412896006
	H326	2.340681135	-1.870969862	-7.778324947
	H327	2.831519558	-9.334035549	-1.400186381
	H328	2.180088164	-6.722060633	-7.418171216
	H329	1.563647277	-5.324017358	-5.402581602
	H330	7.025223993	-12.461413548	2.630135570
	H331	5.744824643	-0.946764498	1.348250053
	H332	8.344781123	-5.834959463	-0.259745768
	H333	7.038550342	-3.822180019	-1.050040910
	H334	9.181240814	-6.924535746	1.670752506
	H335	4.413202309	-2.803073706	4.769417207
	H336	3.189841114	-8.296740483	-4.262610686
	H337	4.547744710	-8.616275926	-3.174478835
	H338	1.548395025	-8.778243368	-2.485781293
	H339	0.545495099	-2.054116784	-6.151998412
	H340	-3.689401089	2.294675802	-5.451728719
	H341	-5.886411770	1.114172817	-5.810416729
	H342	2.262187069	-6.184259660	0.267697854
	H343	8.455199727	-7.366162804	-0.487237083
	H344	7.384887571	-9.283136715	-0.302361377
	H345	4.406090042	-1.110707587	4.638023216
	H346	4.657582243	-7.401608358	-7.407722744
	H347	6.063563677	-5.949481255	-3.912489800
	H348	-0.461625044	-2.801377490	-7.427440014
	H349	3.390997853	4.314391799	-7.210303064
	H350	8.096869631	0.031260388	3.147899464
	H351	7.957810411	0.484452962	1.432901932
	H352	8.103175978	-6.578502023	-7.517910351
	H353	7.471964831	-7.880461422	-6.752805195
	H354	9.968353902	-8.511311655	-2.998306396
	H355	8.047033875	-8.126988898	-4.162570084
	H356	11.104495581	-7.451334990	-5.242919325
	H357	9.879865814	-6.811774262	-2.484433446
	H358	10.660608119	0.211048605	1.132602479
	H359	-2.580087894	13.189429133	4.343193993
	H360	10.658685595	-7.009997043	4.290907053
	H361	10.010397319	-4.562584735	3.226976776
	H362	-4.572780812	12.344975007	2.252261968
	H363	-3.688549486	10.252271379	4.335997042
	H364	-5.313630908	10.947191576	4.199941400
	H365	0.777002454	-3.647321449	-1.096936442
	H366	4.190026854	-1.337795905	0.064222855
	H367	-6.655256405	6.870550061	-0.638037826
	H368	0.808209309	-1.550666883	-0.499795949
	H369	1.678978578	-2.215280683	-0.070998179
	H370	3.417610317	-1.320011591	-0.187056980
	H371	-1.978165980	-0.128695850	-0.544797063
	Mo372	3.354105009	-4.459593838	-2.743717293
	N373	-7.318862803	-7.232226086	0.383605771
	N374	-8.589746751	-4.655556497	2.777252087

	N375	-7.469792904	-4.921138880	5.345292592
	N376	-4.781042366	-5.717580949	4.972864374
	N377	-3.628450740	-3.363349702	3.914373219
	N378	-3.893498712	-1.647879367	6.196429457
	N379	-2.554896313	-3.049411987	8.366368413
	N380	-0.180714290	-4.345195045	7.348216914
	N381	0.976713900	-2.720846442	5.292576438
	N382	0.951523579	0.121145049	6.156114876
	N383	0.976806902	-0.020130404	9.109877394
	N384	6.120067598	-11.572096598	4.395620187
	N385	6.674003855	-9.288277741	3.096203484
	N386	5.336184082	-3.245232706	2.368373929
	N387	4.827094809	-1.992033642	4.295501080
	N388	5.308800205	-0.933002301	2.285994455
	N389	9.349021069	-7.059482621	2.692143572
	N390	-6.274458156	-2.899957946	-2.207839037
	N391	-2.438718900	-5.556524431	-3.006089473
	N392	-6.382950471	-0.422348585	-1.371702818
	N393	-8.500692875	0.655030482	0.104581983
	N394	-7.660934122	0.319865597	2.762700924
	N395	-5.476173895	2.061765947	3.013417464
	N396	-3.363506851	2.688961730	-0.623242687
	N397	-2.550717992	0.708679532	-0.335482815
	N398	-6.074677826	4.678658148	3.743254013
	N399	-9.633249242	7.704289824	2.916705923
	N400	-9.329389538	9.478551217	4.214231940
	N401	9.659161332	2.580699724	1.513883058
	N402	6.202798620	7.230560029	-0.836460177
	N403	3.820440883	8.484568633	-3.111073085
	N404	1.125848697	7.891376827	-3.284291169
	N405	-3.716885113	6.479625753	-3.888806551
	N406	-4.081547744	8.353740907	-5.248351842
	N407	-4.908333877	8.303550479	-3.088466433
	N408	0.463164529	6.353614269	-0.936392298
	N409	1.017945292	8.227641301	1.063722227
	N410	-0.354950126	10.622064268	0.954685591
	N411	-2.256909063	10.340505074	-2.522617978
	N412	-2.594775965	11.634051519	2.298556988
	N413	2.893832125	-4.489852972	-10.594382733
	N414	1.499483126	-2.417377421	-7.985009289
	N415	0.536195177	0.363376315	-7.275325838
	N416	2.605279748	2.368657475	-7.499432655
	N417	-2.894758385	-2.771101426	-9.752670447
	N418	-4.286948091	-1.007259482	-7.979401764
	N419	7.298305588	-6.893739122	-6.968537970
	N420	3.640422604	-5.552589409	-4.849167915
	N421	2.850808405	-6.429544723	-6.712742418
	N422	8.724595950	-7.361016063	-4.189553641
	N423	-0.062611348	2.424182146	-0.257965978
	N424	0.656396810	1.800283567	-0.861567618
	O425	-6.451485673	-5.272433673	2.305590203
	O426	-6.503913735	-2.868599739	5.125540788
	O427	-4.345337910	-5.086087032	7.121706873
	O428	-1.659787156	-3.997858523	4.868663193
	O429	-2.048608591	-0.561245968	5.412019672
	O430	-1.808689368	-0.974560485	8.970137944
	O431	0.745850895	-4.080906109	9.432783893
	O432	3.000110542	-3.707396950	5.749743327
	O433	3.109540648	-0.003953572	5.418751361
	O434	2.718660405	1.421067435	8.887757229
	O435	3.410002152	-0.556797241	11.687413479
	O436	7.247527713	-10.376800350	1.139053718
	O437	8.483945900	-7.759296081	4.704533232
	O438	12.040865346	-4.733843881	3.172043981
	O439	-5.709818410	-0.897920161	0.771653804
	O440	-4.254514805	-4.322298392	-3.595122311
	O441	-7.229924852	2.499737826	0.545187583
	O442	-6.743946988	3.091299363	-2.450368098
	O443	-9.521993730	1.439382562	3.506440179
	O444	-5.113315652	1.382720295	5.164856690
	O445	-3.914759054	5.006761988	4.409411343
	O446	-6.097715938	7.115064171	6.317228840
	O447	11.136223334	-0.198211976	3.074412275
	O448	3.909828384	4.556602212	3.656758288
	O449	3.467189546	8.391557401	-0.861676185
	O450	0.549891183	10.115015034	-3.333872106
	O451	-1.634318688	7.233350206	-1.172789876
	O452	-0.534728489	7.207757956	2.411096361
	O453	2.470626127	5.679596603	1.384806936
	O454	1.088138782	11.600786142	2.443948747

	O455 -2.502423616 13.593638195 1.136707550<br>O456 -3.469350704 11.042646626 -0.717872319<br>O457 -4.565028169 13.625277652 4.559047600<br>O458 -5.688400744 6.882534250 -0.497670657<br>O459 0.708261243 -4.373592059 -8.891549134<br>O460 -1.258997976 -0.570196843 -8.351846526<br>O461 1.103808089 3.772322346 -8.472990491<br>O462 4.822032598 4.473010004 -9.423736273<br>O463 -6.020734008 -2.480033769 -8.135016931<br>O464 -2.199285165 -6.519963115 -5.873284566<br>O465 -0.386228824 -5.654421350 -6.843405187<br>O466 -4.938688926 2.487349334 -7.754896309<br>O467 9.378040144 -5.310284230 -4.899707194<br>O468 12.228110958 -6.841979593 -3.652568804<br>O469 -0.010872476 -6.435469475 -1.460343111<br>O470 -0.297265188 -7.506870876 0.491613683<br>O471 4.647507791 -10.912563662 -4.116463940<br>O472 2.392808967 -10.963982818 -3.938034891<br>O473 4.782128553 -5.946879626 -1.955611580<br>O474 4.873968804 -7.703641406 -0.555352473<br>O475 2.240492070 -6.192538038 -2.466331141<br>O476 8.947929914 -6.598384962 -0.084434359<br>O477 6.916078743 -4.658022303 -0.560882942<br>O478 -4.713656095 4.807046999 -1.785741180<br>O479 7.287522756 -10.365233534 -3.553733248<br>O480 7.370349240 -8.647752090 -1.062000955<br>S481 3.221294849 4.966680538 -2.551080556<br>S482 1.677914253 1.986601360 -3.644847934<br>S483 1.481412560 -3.334209430 -3.659644923<br>S484 3.519278773 1.698917345 -0.484909506<br>S485 0.146637151 -0.894114508 -1.693465720<br>S486 3.325479339 -0.831860712 -5.754454006<br>S487 3.281930179 -3.808461321 -0.516821057<br>S488 5.226696792 1.724655467 -3.629987861<br>S489 5.166040903 -3.044669153 -3.329525881<br>S490 6.240041963 -0.929332057 -1.006051941<br>end

S2BH-2N2x-7Hn-26br-6N2x (16)

Fe(139) -2.362<br>Fe(140) 0.040<br>Fe(141) -2.461<br>Fe(142) 2.412<br>Fe(143) 2.551<br>Fe(144) 0.730<br>Fe(145) -0.013<br><br>**S=1/2**	2n2xs2bh26h6n2x.car_3<br>C1 -7.849560119 -5.382886675 -0.380357780<br>C2 -8.027005815 -6.475124277 -1.438670802<br>C3 -8.905456333 -3.511142463 0.805624162<br>C4 -8.531144322 -3.842079846 2.248033900<br>C5 -8.451622491 -5.524031597 4.022158214<br>C6 -6.938653067 -5.620863858 4.247153588<br>C7 -4.723260605 -5.865959195 3.253162633<br>C8 -4.205867576 -4.439409008 3.443837708<br>C9 -4.492365710 -2.076873624 2.854043817<br>C10 -4.752149104 -1.559191698 4.276720575<br>C11 -6.214229996 -1.483140291 6.202250420<br>C12 -5.361538658 -2.113744243 7.312052798<br>C13 -4.568817538 -4.199960067 8.330755346<br>C14 -3.055442834 -4.456286558 8.254484871<br>C15 -1.142874465 -5.049820848 6.821225146<br>C16 -0.291688803 -3.878179359 6.323744921<br>C17 -0.346255800 -1.597132134 5.380898694<br>C18 -0.416847516 -0.429904650 6.376879948<br>C19 -0.836226042 -1.253703934 3.951108520<br>C20 -0.236503786 0.064281805 3.451819881<br>C21 -0.521213292 -2.392928065 2.976060194<br>C22 -1.794189074 1.246652933 7.513597321<br>C23 -1.713536292 0.836259800 8.996091698<br>C24 -3.069194183 2.051925526 7.168618856<br>C25 -3.306883397 3.167930883 8.191737554<br>C26 -2.966061248 2.633098590 5.752161052<br>C27 -2.476664065 -0.613322351 10.783339558<br>C28 -1.084143974 -0.867658612 11.324279017<br>C29 5.090319876 -11.842920821 4.213815231<br>C30 5.824660438 -10.550208876 3.869438757<br>C31 6.007442551 -8.157143762 4.529716951<br>C32 7.061333786 -7.819762122 5.602154686<br>C33 4.809378726 -7.195455978 4.604412105<br>C34 5.215401067 -5.745453545 4.353931052<br>C35 4.012896453 -4.824029858 4.163283312<br>C36 4.142006012 -2.315951034 4.185568991<br>C37 9.274972948 -6.839830479 5.996399890<br>C38 9.461808639 -5.341359551 6.084639500<br>C39 -4.972803185 -2.535867570 -2.687896938

	C40	-5.405326664	-1.122492088	-2.253789853
	C41	-3.477027691	-2.706466269	-2.333938970
	C42	-2.928578450	-4.146216098	-2.405255458
	C43	-2.376075934	-4.567755441	-3.766637446
	C44	-5.683051586	1.172469055	-3.113081978
	C45	-6.928045407	1.604115006	-2.326132182
	C46	-5.587013399	1.850663925	-4.486287909
	C47	-9.244309995	1.080341012	-1.719448022
	C48	-9.123074123	0.992849553	-0.194197377
	C49	-7.743861289	0.361879203	1.730468569
	C50	-6.620067190	1.284860759	2.203803649
	C51	-4.966964720	3.008770337	1.673750836
	C52	-5.526247815	4.283678633	2.343944054
	C53	-4.136735137	3.510090282	0.481619365
	C54	-3.391500132	2.475692420	-0.290077682
	C55	-3.080127782	1.163221327	-0.046400051
	C56	-2.223110923	1.705326391	-2.034135898
	C57	-7.309471397	5.974292050	2.213133786
	C58	-7.456522329	6.074460568	3.730833640
	C59	-8.689758405	6.065774255	1.518532999
	C60	-9.272831563	7.439067342	1.355302655
	C61	-9.353181345	8.496849756	2.239571854
	C62	-10.305638252	9.029627529	0.311161198
	C63	8.484145284	1.430466125	5.092673026
	C64	9.532950663	0.335115909	5.031631020
	C65	7.152501662	0.809368499	4.605258887
	C66	5.992311018	1.773485011	4.671254039
	C67	5.204442240	1.895113679	5.830800137
	C68	5.660375430	2.573780580	3.565798436
	C69	4.114626774	2.771754109	5.882138397
	C70	4.586769174	3.464309514	3.607210398
	C71	3.806609100	3.553755496	4.762748920
	C72	5.544025981	6.848591602	-0.086791991
	C73	4.540000996	7.964476847	-0.420115540
	C74	4.816545532	5.522168790	0.133145299
	C75	3.841886316	9.644367603	-2.093962980
	C76	2.429018758	9.286062020	-2.561290573
	C77	0.831016217	7.397522447	-2.892515483
	C78	0.087845711	6.977249958	-1.598380864
	C79	1.049922590	6.211914440	-3.858791849
	C80	-0.149794747	5.346362843	-4.257043724
	C81	-1.123177801	5.938155399	-5.287496796
	C82	-2.712511631	7.837233235	-5.014746695
	C83	0.173093668	5.919780615	0.607428413
	C84	-0.141184555	7.100580285	1.537436872
	C85	1.015688475	4.900601677	1.401092723
	C86	0.807285977	9.105382941	2.573188157
	C87	0.242399959	10.474764718	2.228531246
	C88	-0.837625545	11.855991148	0.532640980
	C89	-2.209684083	12.397947952	1.004272552
	C90	-0.706696853	11.950250067	-0.989956691
	C91	-1.742422378	11.140169507	-1.760067959
	C92	-4.387437377	11.872200292	1.999634405
	C93	-4.491058212	12.919404550	3.100067193
	C94	-5.147214877	10.583817294	2.423480719
	C95	-5.197274215	9.563940631	1.308293288
	C96	-6.215960172	9.624866818	0.344160752
	C97	-4.203000174	8.583961044	1.158037182
	C98	-6.228781001	8.760574937	-0.752416101
	C99	-4.185248422	7.726697517	0.053143580
	C100	-5.199165981	7.829965426	-0.894443714
	C101	5.682507638	-3.608257673	-8.264217462
	C102	4.197800555	-3.369851018	-8.055734123
	C103	2.527798509	-1.880122464	-6.977652420
	C104	2.130467188	-0.502944615	-7.524084744
	C105	2.539771969	1.915317521	-7.314486735
	C106	3.750288150	2.843314208	-7.249346411
	C107	5.906668720	3.429024736	-6.297548619
	C108	6.637646826	3.743756380	-7.587161834
	C109	-1.013930371	-3.066731979	-9.672932725
	C110	-2.074481984	-1.975940689	-9.492664391
	C111	-0.785724991	-3.798439102	-8.329021596
	C112	-0.023053336	-5.120931035	-8.532298395
	C113	0.412717355	-5.693286821	-7.200578868
	C114	-2.578991825	0.318082099	-8.830071431
	C115	-1.975118717	1.614465603	-9.332011178
	C116	-3.274006574	0.414535859	-7.449225009
	C117	-2.239800943	0.417716612	-6.364392859
	C118	-1.589349836	1.609514757	-6.004576927
	C119	-1.757664512	-0.803128101	-5.862838386

	C120	-0.417801672	1.566746011	-5.245376974
	C121	-0.590082999	-0.842849629	-5.101393999
	C122	0.106753079	0.340316179	-4.834891509
	C123	8.655583184	-6.116194669	-3.524680014
	C124	9.644504886	-6.240909945	-2.355813309
	C125	7.233512066	-6.437997293	-3.039176767
	C126	6.124461994	-6.179480375	-4.006977976
	C127	5.967235793	-6.720417009	-5.266002292
	C128	4.128739424	-5.621666689	-4.709071933
	C129	10.525562186	-7.532761004	-0.506656371
	C130	11.963736521	-7.202325782	-0.839323016
	C131	0.641672473	-7.004928336	-0.392005315
	C132	2.033888310	-6.971843825	0.235943510
	C133	3.176212455	-7.019918082	-0.820480049
	C134	3.211537809	-8.433791496	-1.474619147
	C135	4.423323453	-8.743979661	-2.356105259
	C136	4.503204825	-10.216382071	-2.817733319
	C137	4.532523243	-6.784607601	-0.143164687
	C138	3.918913030	-0.647476741	-1.557868941
	Fe139	4.448943707	-1.841085550	-3.010213417
	Fe140	2.345574058	0.525569725	-0.918184028
	Fe141	5.279296245	0.484558713	-0.532091681
	Fe142	4.258029014	0.696286218	-2.920618906
	Fe143	4.159221104	2.725317478	-1.228396478
	Fe144	4.985955797	-1.966267176	-0.533172484
	Fe145	2.353141435	-2.209823702	-1.098852624
	H146	-6.251391011	-7.436652099	-1.149736510
	H147	-6.226062227	-6.390883033	-2.402281171
	H148	-9.799543861	-4.782314134	-0.658179784
	H149	-8.603428211	-6.064990689	-2.283424315
	H150	-8.667042098	-7.252668817	-0.986639289
	H151	-8.161949798	-2.780369099	0.453514269
	H152	-9.281752789	-5.734583755	2.049992023
	H153	-9.888136162	-3.020257275	0.807725504
	H154	-8.898294800	-6.510758713	4.201394002
	H155	-6.601837525	-5.954000671	2.238018448
	H156	-8.836416664	-4.831523987	4.781693692
	H157	-3.436812289	-1.880661268	2.627887097
	H158	-5.526534730	-3.757778492	2.021537638
	H159	-4.298746348	-6.293878546	2.334602298
	H160	-6.058817152	-0.397663963	6.257249269
	H161	-6.606742661	-2.465688688	4.320411317
	H162	-4.368081192	-6.458527401	4.105184228
	H163	-5.108134196	-1.511314378	2.141237624
	H164	-5.634145856	-3.985237610	6.488495242
	H165	-7.271542338	-1.700285942	6.407438257
	H166	-0.944593626	-3.351280131	3.306925806
	H167	-0.942872803	-2.173127975	1.982794853
	H168	0.565882417	-2.513195038	2.846140729
	H169	-1.928264762	-1.126574480	4.007561983
	H170	-5.067646105	-5.178589181	8.397758480
	H171	-2.854029179	1.850027684	4.989981612
	H172	-3.145661112	-0.661226830	8.758654485
	H173	-3.474177321	2.775133176	9.204942626
	H174	0.855158230	-0.008993270	3.327862371
	H175	-0.448554326	0.903717958	4.129585336
	H176	8.356940332	1.712029457	6.156180555
	H177	-3.869410494	3.208577205	5.504287157
	H178	-4.189545409	3.757343687	7.903721385
	H179	-2.101331968	3.313808629	5.680266674
	H180	-2.441810560	3.849158696	8.230778366
	H181	-3.929182993	1.358801163	7.203227627
	H182	-0.918740953	1.895829281	7.364441393
	H183	-2.459870729	-0.230510125	6.093995312
	H184	-0.659951704	0.318803264	2.469927965
	H185	1.420852048	4.152840223	0.701452328
	H186	0.334671168	4.394067972	2.100063930
	H187	2.155340359	5.133400509	2.993467915
	H188	2.242478114	4.370040307	5.574895405
	H189	4.325656938	4.060620926	2.733431021
	H190	6.230937516	2.476359849	2.639920759
	H191	5.433605244	1.283516854	6.707453553
	H192	-1.997789250	-2.865353680	5.857078045
	H193	-1.033693793	-5.869489938	6.095769817
	H194	-0.710721039	-5.383253486	7.773522728
	H195	-3.155889202	-4.753165135	6.197880640
	H196	-4.740457180	-3.658708717	9.268839395
	H197	3.499694300	2.833012581	6.783949316
	H198	0.721377211	-1.834506516	5.322115669
	H199	-2.976673904	0.131634285	11.423928000

	H200	-3.031420899	-1.561239010	10.863691666
	H201	-0.335094095	-1.191963103	10.564311834
	H202	10.222907487	-7.307690086	5.688780904
	H203	-0.979083768	-5.784262608	-4.635844527
	H204	1.584391372	-6.102222072	-1.829420231
	H205	-0.683738779	-5.852805992	-9.018953057
	H206	0.850167176	-4.958529700	-9.180409153
	H207	-1.751849492	-4.011750269	-7.846093841
	H208	-1.193469708	1.481450859	-10.115558024
	H209	-1.487645506	-3.781695231	-10.361793325
	H210	-2.886471157	-2.054062404	-2.999677842
	H211	-3.721978293	-4.865549116	-2.137738264
	H212	-0.657417614	-0.641282389	-8.756447779
	H213	-3.384473103	0.112776383	-9.565991148
	H214	0.764340682	-1.990390510	-9.708440222
	H215	-0.901991678	-5.758725611	-2.880742412
	H216	-0.235516489	-3.131889395	-7.646697329
	H217	0.754444844	-3.315998461	-10.686889284
	H218	-6.096883163	-3.234719126	-4.272039297
	H219	-3.348293464	-2.348979354	-1.300924622
	H220	2.198356968	-4.934192061	-7.516764199
	H221	-2.145308280	-4.273572034	-1.645057528
	H222	-4.326849437	2.524976360	2.424710331
	H223	-3.300886763	0.519331361	0.794732113
	H224	-8.658584212	0.682909537	2.248472285
	H225	-7.317872497	-0.031531857	-0.323797744
	H226	-7.236643461	4.074738591	1.229498547
	H227	-8.992832019	8.630862087	3.253392350
	H228	-7.666041080	5.096040614	4.226978427
	H229	-9.398691672	5.397123150	2.038279145
	H230	-6.655750426	6.791454780	1.869090673
	H231	-10.825120236	9.628775401	-0.433025923
	H232	-3.420324758	4.246633663	0.880697321
	H233	-4.798506726	4.049948584	-0.214296465
	H234	-10.227411235	10.422573999	1.919628645
	H235	-8.570812986	5.659166435	0.502070244
	H236	-6.324683022	2.063411310	0.291760268
	H237	-7.521216912	-0.655189839	2.085266683
	H238	-9.621898781	2.089368500	-1.935050155
	H239	-1.701205612	1.637310923	-2.986517717
	H240	-10.006294247	0.362212772	-2.048555428
	H241	9.649812504	3.062250678	4.669749088
	H242	8.119654684	3.189297555	4.110554765
	H243	1.765335858	5.975432818	-0.843968759
	H244	-0.793590407	5.450777494	0.372687610
	H245	1.632950188	7.973841466	0.990157065
	H246	-3.047343815	3.672048189	-2.054517192
	H247	-4.480828311	4.357872933	-3.478185694
	H248	-5.759979889	3.672919425	-5.186943885
	H249	-4.358565260	5.573286400	-2.457212287
	H250	-4.658811655	1.508949170	-4.973810620
	H251	-6.446407420	1.557720692	-5.109084675
	H252	0.318265321	11.687548508	-1.287936657
	H253	-0.866583896	13.005759438	-1.262252580
	H254	-0.263176827	10.380535595	-3.001986289
	H255	-1.923127696	9.885808233	-3.327806219
	H256	-2.519849832	9.455206166	-6.264033404
	H257	-1.517970910	5.135646979	-5.929402105
	H258	-7.037824378	8.809724529	-1.485339584
	H259	-3.416402573	8.466418628	1.908191600
	H260	-3.976586704	7.992912176	-3.395636181
	H261	1.795002533	5.545501455	-3.394841880
	H262	2.893850292	7.315640747	-2.367402044
	H263	-0.704145287	5.003473203	-3.369213961
	H264	-7.950673968	-0.013188183	-3.038709984
	H265	4.348793351	10.165302135	-2.916390386
	H266	-5.418257284	-0.726467806	-4.207064844
	H267	1.523226173	6.614771136	-4.769319967
	H268	-3.364591067	7.020027526	-0.080093332
	H269	3.717241106	10.360590188	-1.268062878
	H270	-1.730570347	8.016402757	-6.821387828
	H271	-4.224856772	9.163305708	-4.645515892
	H272	-4.446792567	-3.278286903	-4.534154733
	H273	-5.546949729	-3.232233376	-2.049347514
	H274	-7.018405439	10.358746454	0.449642961
	H275	-4.845608724	1.523442776	-2.493584038
	H276	0.212506180	8.175457750	-3.353215987
	H277	-0.580195770	6.634802249	-5.938623344
	H278	0.275208536	4.443316293	-4.715194769
	H279	-2.809206391	6.083247526	-3.987011855

	H280	0.243057626	8.726376257	3.438226865
	H281	-0.459232450	9.735887741	0.454976260
	H282	-0.123859467	12.575813384	0.962791101
	H283	1.850387356	9.257333095	2.883740908
	H284	-2.711694946	10.571265937	1.727499387
	H285	6.213795539	-2.645423924	-8.299590171
	H286	6.029089846	-4.130987650	-7.348166793
	H287	8.699091156	-5.059690928	-3.827965520
	H288	6.876496561	-4.686261072	-9.514987146
	H289	5.314815082	-5.178371855	-9.504641642
	H290	5.568410568	4.762962920	0.381350188
	H291	5.652889538	7.380565986	1.884271412
	H292	5.407214779	8.203389324	-2.271852478
	H293	4.102292293	5.624144472	0.961510764
	H294	6.853692707	8.023584321	0.977568281
	H295	6.633945343	2.966434748	-5.612276238
	H296	6.258260920	6.734275650	-0.917418202
	H297	4.322031353	-7.303345981	5.587821128
	H298	6.459343318	-8.090605373	3.531653911
	H299	4.828466684	1.623775420	-5.937279916
	H300	4.368494139	-0.297032172	3.906390425
	H301	2.174616430	1.918880602	-8.348246489
	H302	-0.211419793	-1.798685258	-4.732455775
	H303	1.757114074	2.388546118	-6.696628440
	H304	3.458390053	-4.714131146	5.102887271
	H305	3.282924037	0.313314216	-6.057187523
	H306	3.319766633	-5.244610938	3.416636039
	H307	4.089269597	-7.519371278	3.836021807
	H308	5.835353063	-5.694689111	3.447059767
	H309	5.827769041	-5.361658304	5.185283088
	H310	-3.952550733	-0.445627998	-7.353834543
	H311	4.920607029	-9.766725214	5.473756077
	H312	4.299125308	-11.949219594	3.456256084
	H313	1.051016876	0.321863560	-4.296418013
	H314	5.135666187	-12.178907867	6.244862905
	H315	3.665821973	-12.445291023	5.572083265
	H316	5.115249331	-3.540278938	2.900192176
	H317	0.114067773	2.482543154	-4.987250907
	H318	-2.282107509	-1.732836698	-6.094397843
	H319	7.825296486	-9.920662470	-0.671079732
	H320	7.189850683	-7.504701361	-2.759173308
	H321	6.536471162	-8.227037257	0.767522466
	H322	7.170250974	-10.470555991	-1.967128344
	H323	6.546075196	2.966505770	-8.383045086
	H324	6.297832122	-5.055114311	0.770326352
	H325	2.128982987	-7.788333294	0.962112375
	H326	4.636811064	-1.689405155	-6.978439165
	H327	3.198534773	-9.146236009	-0.635407263
	H328	4.257075849	-6.635810938	-6.552902632
	H329	3.119087674	-5.238526717	-4.779160936
	H330	5.801215293	-12.669878903	4.047073792
	H331	5.335893345	-1.268059380	2.852911719
	H332	8.075164551	-5.893732643	2.084585676
	H333	7.143639778	-3.795804086	1.085135258
	H334	8.299508365	-7.063097114	4.099919875
	H335	2.666766473	-2.945809388	5.531253138
	H336	4.396950081	-8.119239096	-3.261374511
	H337	5.350468300	-8.499473968	-1.821428893
	H338	2.281373798	-8.592797303	-2.042215574
	H339	2.463253033	-1.895936890	-5.877910740
	H340	-1.971253535	2.562786555	-6.376031023
	H341	-3.876202347	1.335324398	-7.451656998
	H342	2.111461169	-6.024224943	0.795625728
	H343	8.255708502	-7.419740016	1.822919173
	H344	7.165486612	-9.350250365	1.622513449
	H345	3.100877898	-1.270957855	5.613608988
	H346	6.627961314	-7.341150444	-5.856661452
	H347	7.039188937	-5.878446143	-2.116468200
	H348	1.840712177	-2.620919400	-7.391013801
	H349	5.563670140	4.369957754	-5.839877216
	H350	6.944599598	-0.072078751	5.229182010
	H351	7.290862405	0.461966267	3.568708001
	H352	9.982778019	-6.660741874	-4.989226385
	H353	9.095490618	-7.917342688	-4.431158808
	H354	10.502533593	-8.584347315	-0.179462082
	H355	9.012887965	-8.183472327	-1.860633350
	H356	12.250355172	-7.383416937	-1.903286513
	H357	10.187329429	-6.912069471	0.338394343
	H358	9.996644120	0.201173905	4.024407262
	H359	-3.593993192	13.005294519	3.757863747

	H360	9.034435980	-7.196438411	7.013260006
	H361	8.541693778	-4.735313836	5.894000862
	H362	-4.866037797	12.292290443	1.099210538
	H363	-4.664851896	10.158307971	3.317247886
	H364	-6.165264382	10.883497709	2.711014635
	N365	0.921597081	-3.175626349	-0.533610624
	H366	4.419664946	-1.334007859	0.697800952
	H367	-6.014011181	7.015634777	-2.466857370
	H368	-0.054647314	-0.882230746	-1.029332799
	H369	2.178954068	-1.011258643	0.034356965
	N370	0.003612725	-3.726464852	-0.181578375
	H371	-1.976069471	-0.215758679	-1.253461679
	Mo372	4.219010532	-4.250154233	-1.769490919
	N373	-6.808426419	-7.090513344	-1.935398271
	N374	-8.969149162	-4.660877127	-0.083572030
	N375	-8.830476102	-5.085222218	2.689798561
	N376	-6.175862047	-5.924717408	3.166251696
	N377	-4.799204754	-3.488306278	2.679788171
	N378	-5.900624396	-1.964628948	4.866096147
	N379	-5.173608468	-3.456557863	7.234609908
	N380	-2.552592932	-4.762214668	7.024963357
	N381	-0.983996559	-2.789340699	5.913316927
	N382	-1.646398980	0.096061456	6.622477103
	N383	-2.463602412	-0.225997180	9.384869471
	N384	4.476519788	-11.825074082	5.548187141
	N385	5.544534780	-9.524067853	4.696847007
	N386	4.469049587	-3.514155535	3.694361272
	N387	3.254598673	-2.181310375	5.190142672
	N388	4.740013481	-1.216117895	3.686077187
	N389	8.184717002	-7.228016401	5.124177413
	N390	-5.222326105	-2.728915401	-4.120441479
	N391	-1.357409373	-5.446670934	-3.744187321
	N392	-5.577680481	-0.265970153	-3.294727188
	N393	-8.023711906	0.824986670	-2.466147054
	N394	-8.022207843	0.366224121	0.306599090
	N395	-6.004198558	2.068852686	1.264801591
	N396	-2.845091288	2.780810246	-1.530599946
	N397	-2.368046735	0.717335029	-1.141519343
	N398	-6.735569531	4.679899810	1.875072044
	N399	-9.870817075	7.784454703	0.154160599
	N400	-10.009661719	9.498571861	1.555972462
	N401	8.898065555	2.533171813	4.220814860
	N402	6.312212245	7.167607863	1.128354781
	N403	4.666655369	8.531901909	-1.657461476
	N404	2.142035067	7.968585592	-2.607726174
	N405	-2.259654060	6.631449586	-4.672995095
	N406	-2.239754446	8.495840084	-6.087932821
	N407	-3.613827794	8.466879113	-4.226251591
	N408	0.813120232	6.328657913	-0.644417833
	N409	0.760679867	8.122584241	1.504905217
	N410	-0.465449029	10.555876659	1.061039999
	N411	-1.272574853	10.530754921	-2.882425438
	N412	-2.996199477	11.547489163	1.711058055
	N413	5.912272050	-4.346544159	-9.508046962
	N414	3.878987629	-2.242996270	-7.384459354
	N415	2.744284160	0.534823986	-6.913300231
	N416	4.815390319	2.487788871	-6.487500612
	N417	0.180003082	-2.541314121	-10.348433726
	N418	-1.645231407	-0.779606832	-8.991476816
	N419	9.039868935	-6.928135821	-4.693111560
	N420	4.963232516	-5.482178401	-3.677420351
	N421	4.708916934	-6.353864269	-5.687322609
	N422	9.660109475	-7.414273230	-1.666996848
	N423	0.109335634	1.888659658	0.431206468
	N424	0.977316519	1.357329499	-0.060429307
	O425	-6.774907160	-5.186337430	0.215248402
	O426	-7.976235382	-2.986347267	2.960565200
	O427	-6.465907082	-5.444000119	5.381982112
	O428	-3.310184090	-4.170342986	4.259589275
	O429	-3.944462528	-0.807983280	4.845456565
	O430	-4.921091037	-1.416882785	8.242878029
	O431	-2.374761777	-4.435977744	9.287916426
	O432	0.949781883	-3.975205685	6.303757789
	O433	0.617924027	-0.005223989	6.916151183
	O434	-0.992464169	1.452131108	9.799763462
	O435	-0.804518460	-0.797465643	12.511719056
	O436	6.581063669	-10.504859288	2.877841832
	O437	6.855764753	-8.062428501	6.804062189
	O438	10.526375545	-4.809239431	6.358600112
	O439	-5.552908326	-0.792225622	-1.060865153

	O440	-2.863498168	-4.135981039	-4.836955262
	O441	-6.895122180	2.606367674	-1.590053455
	O442	-5.571140416	3.281718696	-4.315244679
	O443	-10.016467014	1.483889371	0.509052447
	O444	-6.303503701	1.304757756	3.396738674
	O445	-4.866136751	4.931657651	3.161118307
	O446	-7.405576765	7.115638685	4.362438238
	O447	9.848696061	-0.372146662	5.977245825
	O448	2.735419519	4.427125549	4.735785698
	O449	3.674354927	8.340335826	0.388501700
	O450	1.637861997	10.199484552	-2.866493847
	O451	-1.118612770	7.239166333	-1.462987570
	O452	-1.117702687	7.070738072	2.297206981
	O453	2.079640347	5.543261527	2.107934083
	O454	0.411543325	11.437743798	2.986980351
	O455	-2.524918210	13.575437636	0.783986901
	O456	-2.931898169	11.095028658	-1.416941874
	O457	-5.491150154	13.595467873	3.283832210
	O458	-5.141586098	6.993292847	-2.027951874
	O459	3.350764200	-4.203005118	-8.458046541
	O460	1.326688185	-0.372396555	-8.469929800
	O461	3.712406203	3.927809404	-7.855280582
	O462	7.318411531	4.744727583	-7.745248975
	O463	-3.267977868	-2.179557915	-9.768375384
	O464	-0.337870776	-6.320360894	-6.459892895
	O465	1.678795037	-5.437553648	-6.816196346
	O466	-2.331212780	2.729017824	-8.977024628
	O467	10.430368036	-5.319607161	-2.060118566
	O468	12.786194780	-6.839878046	-0.009652919
	O469	0.553686722	-6.435444662	-1.595780038
	O470	-0.334505380	-7.484159443	0.184518439
	O471	5.650323703	-10.783563159	-2.760908814
	O472	3.444924103	-10.751185391	-3.253225680
	O473	5.246747290	-5.782386815	-0.593474349
	O474	4.917612628	-7.539110437	0.769628940
	O475	2.922296442	-5.973670313	-1.760220741
	O476	8.608451244	-6.671603299	2.379926250
	O477	6.792232811	-4.605535138	1.503292384
	O478	-3.828234518	4.926019095	-2.986276915
	O479	8.058140902	-10.384031644	-1.505979331
	O480	7.401068699	-8.692840311	0.919794718
	S481	3.870914295	4.968434528	-1.358566856
	S482	2.515264787	2.071226325	-2.611317752
	S483	2.653561144	-3.196783032	-3.165633134
	S484	3.773316699	1.606542767	0.657123298
	S485	0.850169438	-0.791366139	-2.044971864
	S486	4.904406195	-0.480552083	-4.685397562
	S487	3.608054253	-3.401586103	0.348360443
	S488	6.031650368	1.848707650	-2.135002149
	S489	6.182417623	-2.979556170	-2.179030573
	S490	6.650228334	-0.843278062	0.521697533
	end			

TS for dissociation of 6N2x (16) to (16d)

Fe(139) -2.286	2n2xs2bh26h6n2xdissd_1_53290.44			
Fe(140) 0.059	C1	-7.845186067	-5.377521067	-0.384702185
Fe(141) -2.544	C2	-8.027447596	-6.468662974	-1.443469468
Fe(142) 2.108	C3	-8.890307602	-3.502623873	0.804803104
Fe(143) 2.770	C4	-8.504240039	-3.833430896	2.243556117
Fe(144) 1.134	C5	-8.417823363	-5.513949886	4.019735503
Fe(145) -0.397	C6	-6.906600570	-5.602546484	4.250955312
	C7	-4.686878491	-5.840836920	3.274038290
	C8	-4.180032178	-4.408685355	3.450346096
	C9	-4.486262933	-2.052996136	2.841533093
S=1/2	C10	-4.750372112	-1.529783299	4.261650796
	C11	-6.207690018	-1.466573106	6.191775077
	C12	-5.355947785	-2.094287255	7.304924633
	C13	-4.563511814	-4.179267994	8.329396884
	C14	-3.051758284	-4.445616413	8.250434492
	C15	-1.151410616	-5.074495984	6.814946783
	C16	-0.308748842	-3.899367304	6.307773290
	C17	-0.375421860	-1.625046975	5.354109229
	C18	-0.436872749	-0.465465663	6.360159208
	C19	-0.868625427	-1.271297303	3.929445728
	C20	-0.282728605	0.054377834	3.432785852
	C21	-0.540943556	-2.397661470	2.945745555
	C22	-1.788630628	1.229398636	7.498595518
	C23	-1.700777913	0.826847028	8.983096115
	C24	-3.060509533	2.042395046	7.158358372

	C25	-3.292144137	3.157913241	8.183345929
	C26	-2.956971453	2.625059017	5.742582930
	C27	-2.466640352	-0.604606505	10.784621454
	C28	-1.073996144	-0.863182405	11.324156666
	C29	5.088655119	-11.837847860	4.218699141
	C30	5.822495370	-10.544444897	3.869220456
	C31	6.005045052	-8.147099596	4.525150290
	C32	7.056073001	-7.806155190	5.599900616
	C33	4.804054167	-7.187893566	4.596420689
	C34	5.203332066	-5.734844334	4.349181934
	C35	3.995873576	-4.820334236	4.153775087
	C36	4.129244227	-2.311744889	4.202401876
	C37	9.274774439	-6.838162418	5.998706990
	C38	9.467012694	-5.339908674	6.084561224
	C39	-4.967437730	-2.537514223	-2.678397727
	C40	-5.401950993	-1.123848332	-2.245136618
	C41	-3.467597295	-2.700572247	-2.331555328
	C42	-2.911742198	-4.138371095	-2.400426501
	C43	-2.366846968	-4.569247359	-3.763053281
	C44	-5.684434199	1.170081895	-3.106819561
	C45	-6.931116889	1.599368967	-2.321580454
	C46	-5.589135793	1.847009674	-4.480886545
	C47	-9.246815330	1.072200712	-1.717041315
	C48	-9.126832585	0.989451349	-0.191484074
	C49	-7.748823344	0.366791127	1.736604681
	C50	-6.625711565	1.291510723	2.207523488
	C51	-4.965355003	3.005727723	1.672811148
	C52	-5.524741018	4.282321796	2.340396816
	C53	-4.134589669	3.502785786	0.479668123
	C54	-3.389339799	2.467817297	-0.292149296
	C55	-3.069536737	1.156654657	-0.049941815
	C56	-2.220652311	1.705046489	-2.038768966
	C57	-7.308860280	5.971703837	2.206503968
	C58	-7.464271200	6.071293387	3.723334012
	C59	-8.687427550	6.063230073	1.508426154
	C60	-9.272204774	7.435824439	1.345018251
	C61	-9.352729539	8.494899016	2.227744491
	C62	-10.309362713	9.022888478	0.300031176
	C63	8.482402341	1.429445273	5.098076693
	C64	9.530828636	0.332843489	5.048293407
	C65	7.150403555	0.806478908	4.614692669
	C66	5.990916347	1.771333520	4.678318997
	C67	5.204592892	1.898875706	5.838466788
	C68	5.658879107	2.566967255	3.569597901
	C69	4.116102117	2.777590520	5.887157121
	C70	4.587208079	3.459912431	3.608380843
	C71	3.808407304	3.555718022	4.764772131
	C72	5.545804802	6.852817215	-0.090385092
	C73	4.546688231	7.976928288	-0.420102009
	C74	4.817530761	5.525073057	0.101437702
	C75	3.844446504	9.653631541	-2.097210282
	C76	2.431497894	9.292060460	-2.563289385
	C77	0.836649676	7.400453012	-2.892719564
	C78	0.095726379	6.984543256	-1.596723176
	C79	1.051506553	6.214739692	-3.859359551
	C80	-0.151181730	5.349862959	-4.251101631
	C81	-1.125249560	5.937831032	-5.283509099
	C82	-2.713976132	7.836789802	-5.009936838
	C83	0.183205889	5.930686063	0.609308603
	C84	-0.133031106	7.112084655	1.537581831
	C85	1.029769597	4.914961169	1.403270234
	C86	0.813354396	9.118779242	2.570653386
	C87	0.244311322	10.485411472	2.223497018
	C88	-0.837905028	11.857570805	0.525610179
	C89	-2.210790823	12.399394459	0.996121905
	C90	-0.707901996	11.954663111	-0.996344265
	C91	-1.741992399	11.140709902	-1.764036538
	C92	-4.386115664	11.873333740	1.994445740
	C93	-4.496244109	12.924383511	3.090632573
	C94	-5.146803195	10.585972147	2.419004911
	C95	-5.197121248	9.565821762	1.304323643
	C96	-6.217092024	9.624204656	0.341364616
	C97	-4.201365490	8.587649971	1.154096334
	C98	-6.230704519	8.757094419	-0.753123913
	C99	-4.183938144	7.728092234	0.051250514
	C100	-5.200443199	7.826944477	-0.894099081
	C101	5.681806426	-3.605658804	-8.266720688
	C102	4.196797493	-3.366782762	-8.057319809
	C103	2.526323093	-1.881571449	-6.970942874
	C104	2.131555247	-0.501386428	-7.510843088

	C105	2.535131321	1.915514025	-7.293921736
	C106	3.744586071	2.845676868	-7.238393738
	C107	5.902708865	3.440220347	-6.295338773
	C108	6.650158802	3.741641328	-7.578960233
	C109	-1.013719729	-3.060258665	-9.671581890
	C110	-2.074926930	-1.970679356	-9.486357955
	C111	-0.785805420	-3.795230036	-8.328465086
	C112	-0.022393781	-5.117559934	-8.532115792
	C113	0.416190570	-5.691635678	-7.201972594
	C114	-2.582805512	0.322737376	-8.822218708
	C115	-1.985957470	1.622834566	-9.322918577
	C116	-3.263142460	0.412937720	-7.434142275
	C117	-2.225750171	0.401868289	-6.351561205
	C118	-1.564645351	1.584707460	-5.982344847
	C119	-1.763263312	-0.825488073	-5.847509827
	C120	-0.408142844	1.527259595	-5.200765873
	C121	-0.610223450	-0.880202220	-5.065395523
	C122	0.093306145	0.294437460	-4.780119826
	C123	8.657915529	-6.110141284	-3.529030396
	C124	9.645871357	-6.238804141	-2.359450880
	C125	7.235273060	-6.443492910	-3.052186236
	C126	6.127690517	-6.179291746	-4.020443585
	C127	5.967754731	-6.716073844	-5.281640295
	C128	4.135694690	-5.608883473	-4.718599260
	C129	10.523783705	-7.537837733	-0.512371360
	C130	11.962678650	-7.210023418	-0.843829559
	C131	0.644675142	-6.989618551	-0.388473083
	C132	2.044110617	-6.991278706	0.224168698
	C133	3.185236484	-7.044361612	-0.829879642
	C134	3.204445479	-8.454598914	-1.484538927
	C135	4.410729083	-8.760728590	-2.373074160
	C136	4.491747142	-10.232737793	-2.835032108
	C137	4.535703731	-6.806965511	-0.141403726
	C138	3.924641604	-0.644629300	-1.558794328
	Fe139	4.501942413	-1.833832954	-2.992646815
	Fe140	2.329976961	0.543543465	-0.940163728
	Fe141	5.280833325	0.491257664	-0.530418643
	Fe142	4.252973277	0.695594758	-2.921975948
	Fe143	4.160013836	2.749049678	-1.295333767
	Fe144	4.987925343	-1.981345630	-0.471754747
	Fe145	2.494818834	-2.100168496	-1.158609777
	H146	-6.254742899	-7.436678523	-1.156413328
	H147	-6.227695951	-6.393015734	-2.410721098
	H148	-9.795459345	-4.772292701	-0.653267794
	H149	-8.602246794	-6.055291653	-2.287706310
	H150	-8.670736710	-7.243371409	-0.991594591
	H151	-8.151255471	-2.771106883	0.445699029
	H152	-9.246905671	-5.729197048	2.047094234
	H153	-9.873249166	-3.012796440	0.816418536
	H154	-8.859384297	-6.503562661	4.197638883
	H155	-6.558528714	-5.941958123	2.244806408
	H156	-8.811389414	-4.822950383	4.776560699
	H157	-3.432852306	-1.846971511	2.613406425
	H158	-5.499860810	-3.751040775	2.017094346
	H159	-4.247363020	-6.280193347	2.368264985
	H160	-6.055703923	-0.380677710	6.247010613
	H161	-6.594927628	-2.453859738	4.312307158
	H162	-4.338304747	-6.417936265	4.139209678
	H163	-5.107383693	-1.499593341	2.123951842
	H164	-5.619950855	-3.966166207	6.481166093
	H165	-7.264693401	-1.686557882	6.395362427
	H166	-0.955045210	-3.363942104	3.264650749
	H167	-0.964014942	-2.165193509	1.956375392
	H168	0.548424723	-2.503184561	2.818253858
	H169	-1.961166262	-1.155290398	3.987313969
	H170	-5.071436427	-5.152774030	8.402792014
	H171	-2.845374838	1.842596916	4.979912356
	H172	-3.140057824	-0.663797450	8.761062519
	H173	-3.459144271	2.764847241	9.196535746
	H174	0.804671165	-0.017382466	3.274108137
	H175	-0.475160266	0.885240656	4.126876415
	H176	8.353599336	1.716675610	6.160060016
	H177	-3.859848047	3.201259125	5.495018252
	H178	-4.173312616	3.750594267	7.897398071
	H179	-2.091622170	3.304975603	5.672208895
	H180	-2.424719766	3.836115498	8.221558690
	H181	-3.923236165	1.352799994	7.193696796
	H182	-0.908820152	1.870667793	7.339522651
	H183	-2.477468219	-0.238574526	6.080689158
	H184	-0.735203783	0.324286512	2.466740294

	H185	1.440365269	4.171485613	0.702450298
	H186	0.351301806	4.401588316	2.099762173
	H187	2.165783555	5.150193999	2.997231403
	H188	2.248560969	4.381781250	5.576310633
	H189	4.326801812	4.053512439	2.732439996
	H190	6.228630142	2.463722885	2.643915802
	H191	5.433950431	1.291090056	6.717792347
	H192	-2.019862373	-2.915314123	5.795457747
	H193	-1.043159498	-5.898225688	6.093738842
	H194	-0.709431944	-5.400322066	7.765373257
	H195	-3.168172645	-4.788136598	6.203250478
	H196	-4.729201060	-3.629952882	9.263737125
	H197	3.502177123	2.843519914	6.789385598
	H198	0.692149797	-1.862044092	5.289143094
	H199	-2.960321943	0.148389005	11.420766081
	H200	-3.026797336	-1.548561627	10.873253021
	H201	-0.326484115	-1.192843567	10.564524280
	H202	10.221583377	-7.309392834	5.692757313
	H203	-0.969234441	-5.780292770	-4.638723377
	H204	1.586814516	-6.137584138	-1.856613835
	H205	-0.683068468	-5.849854939	-9.018074975
	H206	0.850072977	-4.954635889	-9.181006696
	H207	-1.751732027	-4.008677640	-7.845119725
	H208	-1.213781527	1.495706739	-10.116829488
	H209	-1.486877558	-3.772689716	-10.363275809
	H210	-2.883169674	-2.045197247	-2.999376983
	H211	-3.697932866	-4.859693514	-2.118940903
	H212	-0.657954084	-0.630702752	-8.758203770
	H213	-3.393958874	0.116374193	-9.551138874
	H214	0.763054083	-1.982486995	-9.699008516
	H215	-0.883930108	-5.758837600	-2.884829101
	H216	-0.235324110	-3.128329108	-7.646600473
	H217	0.758933751	-3.306474294	-10.678605076
	H218	-6.096816304	-3.236133856	-4.258967622
	H219	-3.337093617	-2.339621695	-1.300079585
	H220	2.201586844	-4.933366681	-7.521269014
	H221	-2.123522143	-4.258140507	-1.644807421
	H222	-4.325847728	2.523010133	2.425022129
	H223	-3.287694151	0.510520428	0.790263621
	H224	-8.664251020	0.689659366	2.252214818
	H225	-7.318608942	-0.030273762	-0.316060960
	H226	-7.233903041	4.071229471	1.224674268
	H227	-8.989828033	8.631215383	3.240380138
	H228	-7.677692880	5.092437100	4.217155940
	H229	-9.396763384	5.393571375	2.026486547
	H230	-6.653734583	6.788847147	1.865316688
	H231	-10.831070125	9.619792553	-0.444335750
	H232	-3.417126664	4.239389428	0.876988453
	H233	-4.795950229	4.042357521	-0.216986486
	H234	-10.229275766	10.419264562	1.905215595
	H235	-8.566619173	5.657233356	0.491895171
	H236	-6.323047210	2.059341978	0.292194456
	H237	-7.526402182	-0.648953539	2.095527447
	H238	-9.625931585	2.079966776	-1.935903628
	H239	-1.698824983	1.639236521	-2.991243895
	H240	-10.007014484	0.351563579	-2.044910451
	H241	9.652905495	3.055642628	4.672969836
	H242	8.125276950	3.185924854	4.109117313
	H243	1.772607203	5.981619270	-0.847761138
	H244	-0.782730898	5.460438559	0.374020557
	H245	1.637151722	7.990678968	0.984189499
	H246	-3.053458469	3.667888226	-2.055799530
	H247	-4.481644997	4.354533308	-3.477408897
	H248	-5.769013824	3.669397636	-5.181577030
	H249	-4.358895288	5.567557215	-2.454433665
	H250	-4.660568956	1.505732830	-4.968164060
	H251	-6.448114492	1.553002667	-5.103768998
	H252	0.317557523	11.696806912	-1.296346805
	H253	-0.872748877	13.010184611	-1.265693107
	H254	-0.260020198	10.378890445	-2.999826337
	H255	-1.917298949	9.872971129	-3.320969377
	H256	-2.504948032	9.463869175	-6.245775061
	H257	-1.518288061	5.132913987	-5.923407307
	H258	-7.040687486	8.803903710	-1.485253787
	H259	-3.412818549	8.473941787	1.902522309
	H260	-3.986045665	7.988971688	-3.396404892
	H261	1.798067195	5.548071104	-3.398558481
	H262	2.899980391	7.321510659	-2.366888271
	H263	-0.705999577	5.015633076	-3.360282849
	H264	-7.950666085	-0.019303231	-3.036649020

	H265	4.349840459	10.172887417	-2.921644202
	H266	-5.416029209	-0.728332746	-4.198893557
	H267	1.520949648	6.617777470	-4.771860428
	H268	-3.362027899	7.023019043	-0.081813552
	H269	3.719520374	10.372437028	-1.273860939
	H270	-1.714180865	8.025988251	-6.806707762
	H271	-4.226799134	9.162958985	-4.644310748
	H272	-4.447295243	-3.286393433	-4.523372261
	H273	-5.534959821	-3.235097656	-2.035465839
	H274	-7.019715155	10.358077074	0.445922415
	H275	-4.848245844	1.524031137	-2.487513970
	H276	0.217514299	8.178479942	-3.352862527
	H277	-0.583963220	6.633634843	-5.936882353
	H278	0.269768719	4.441883738	-4.703699973
	H279	-2.814566528	6.079848318	-3.987417647
	H280	0.251647960	8.739435963	3.437157704
	H281	-0.459904193	9.737355351	0.454206814
	H282	-0.125424471	12.578487827	0.956389175
	H283	1.856698585	9.274230138	2.878617497
	H284	-2.707379791	10.575348207	1.728676149
	H285	6.214283167	-2.643325529	-8.296643491
	H286	6.025940259	-4.133263471	-7.352681223
	H287	8.697557247	-5.051393335	-3.824894634
	H288	6.880244797	-4.679076143	-9.514849530
	H289	5.319159244	-5.170902467	-9.516332233
	H290	5.568797318	4.764258296	0.347035142
	H291	5.629012044	7.366177597	1.885163276
	H292	5.402418865	8.205806593	-2.278165713
	H293	4.097133721	5.611145502	0.926423992
	H294	6.849664551	8.007632687	1.005202440
	H295	6.622146378	2.991532623	-5.593197314
	H296	6.268631964	6.750915415	-0.915083157
	H297	4.314045632	-7.298599128	5.578248065
	H298	6.458780450	-8.081215452	3.527950080
	H299	4.836804692	1.627886789	-5.936484963
	H300	4.365220326	-0.288884380	3.962258235
	H301	2.164421678	1.916251668	-8.325599835
	H302	-0.253522177	-1.839024697	-4.683076790
	H303	1.755195231	2.389916907	-6.673635707
	H304	3.432465768	-4.718698072	5.088880024
	H305	3.277945321	0.314094426	-6.035898962
	H306	3.310832626	-5.243395216	3.400962423
	H307	4.087187815	-7.514015813	3.825857115
	H308	5.827103823	-5.678135374	3.445007136
	H309	5.809208133	-5.348340534	5.184082886
	H310	-3.945576796	-0.443929619	-7.337705419
	H311	4.922393042	-9.756214784	5.473562534
	H312	4.294116265	-11.943109812	3.464107951
	H313	1.023211335	0.267719997	-4.217033079
	H314	5.141858530	-12.169161491	6.249755927
	H315	3.670496838	-12.440685509	5.582492015
	H316	5.090061119	-3.522635196	2.897132546
	H317	0.126945974	2.436665898	-4.924619576
	H318	-2.295839152	-1.747476471	-6.090943206
	H319	7.818194565	-9.917385128	-0.677391725
	H320	7.196109533	-7.513208508	-2.782749862
	H321	6.540225438	-8.228506560	0.764992792
	H322	7.162075140	-10.472537143	-1.972420470
	H323	6.572657228	2.953427444	-8.365866588
	H324	6.301210525	-5.077410548	0.749264237
	H325	2.124103951	-7.819790760	0.939070586
	H326	4.635380024	-1.694672883	-6.965049455
	H327	3.172005254	-9.184511545	-0.660230865
	H328	4.256116621	-6.621178641	-6.565954635
	H329	3.127739169	-5.221116925	-4.785274757
	H330	5.798141666	-12.665046172	4.048385778
	H331	5.311644660	-1.243390496	2.870619801
	H332	8.079024237	-5.895899993	2.075995128
	H333	7.138088115	-3.806701685	1.050873876
	H334	8.301986238	-7.060502035	4.098961359
	H335	2.656080809	-2.949213884	5.546224250
	H336	4.366059430	-8.132601469	-3.275421061
	H337	5.344239225	-8.510036631	-1.851473817
	H338	2.274850547	-8.581061637	-2.059409536
	H339	2.458180076	-1.904587561	-5.871630081
	H340	-1.928002377	2.542347126	-6.361575929
	H341	-3.860590082	1.336799018	-7.425138708
	H342	2.125674664	-6.053114108	0.798611598
	H343	8.250538519	-7.423603581	1.820672003
	H344	7.174975453	-9.349932606	1.615775371

	H345	3.076679351	-1.273270105	5.626517437
	H346	6.625599774	-7.335851500	-5.877746176
	H347	7.033756827	-5.892901676	-2.125565737
	H348	1.838706577	-2.618347044	-7.390890197
	H349	5.550148725	4.386255431	-5.855692721
	H350	6.944235294	-0.070826839	5.245158049
	H351	7.285700625	0.452653232	3.579950113
	H352	9.989814822	-6.634892226	-4.998027702
	H353	9.112078765	-7.901893764	-4.448193453
	H354	10.497462385	-8.590241641	-0.188252374
	H355	9.007087000	-8.178519980	-1.866647288
	H356	12.249518999	-7.390483278	-1.907823285
	H357	10.186368313	-6.918660867	0.334214672
	H358	10.000226519	0.191808893	4.044635057
	H359	-3.600711429	13.019615358	3.749357973
	H360	9.030486558	-7.191955252	7.015546321
	H361	8.549755257	-4.730492821	5.891548216
	H362	-4.863573669	12.290083432	1.091886722
	H363	-4.665606973	10.159826847	3.313202782
	H364	-6.164892916	10.886639941	2.705822908
	N365	0.626502792	-3.515718916	-0.367357519
	H366	4.459431306	-1.333531762	0.765703065
	H367	-6.017851533	7.004801699	-2.461761250
	H368	-0.017035372	-0.976366878	-0.841194487
	H369	2.216006176	-0.946798125	0.026743001
	N370	-0.251553376	-3.980896352	0.130086410
	H371	-1.940974908	-0.207871493	-1.259774717
	Mo372	4.208904821	-4.250522638	-1.758603494
	N373	-6.811948297	-7.089216293	-1.941257523
	N374	-8.961802106	-4.652803129	-0.083057903
	N375	-8.794798323	-5.078578445	2.685286255
	N376	-6.137474165	-5.906922911	3.174903456
	N377	-4.777367036	-3.469304591	2.675767334
	N378	-5.891169888	-1.947838642	4.856468139
	N379	-5.163866050	-3.436633112	7.229567942
	N380	-2.561355813	-4.794205617	7.026861300
	N381	-1.008962343	-2.823622356	5.876819501
	N382	-1.659187254	0.073419381	6.611193516
	N383	-2.454226236	-0.228521822	9.382899174
	N384	4.479372372	-11.818134775	5.554844103
	N385	5.543993219	-9.515334361	4.694428581
	N386	4.445160215	-3.505128831	3.691939680
	N387	3.259051865	-2.188280461	5.224490973
	N388	4.717831051	-1.206214413	3.706517343
	N389	8.184777601	-7.223179970	5.123647544
	N390	-5.220099239	-2.733420915	-4.109656073
	N391	-1.339439381	-5.438498896	-3.744894850
	N392	-5.575575374	-0.268283366	-3.286668436
	N393	-8.024582897	0.816807806	-2.461165427
	N394	-8.025695105	0.365525442	0.312369610
	N395	-6.003526064	2.066324871	1.265480773
	N396	-2.848540546	2.776144804	-1.534022098
	N397	-2.357184830	0.715417946	-1.147361929
	N398	-6.733924526	4.677551067	1.870005409
	N399	-9.872838920	7.778208922	0.144348430
	N400	-10.012059673	9.494381856	1.543556462
	N401	8.901097508	2.527479330	4.222877148
	N402	6.299189445	7.154555547	1.138510457
	N403	4.671296408	8.543910678	-1.657878682
	N404	2.147674762	7.973831664	-2.610383014
	N405	-2.263675456	6.629492053	-4.671209948
	N406	-2.239651610	8.498398857	-6.080299948
	N407	-3.618283800	8.465128337	-4.223479400
	N408	0.822958061	6.338678191	-0.642852621
	N409	0.766829474	8.135620051	1.503115802
	N410	-0.465300537	10.560023563	1.056583456
	N411	-1.270368233	10.526632548	-2.883074920
	N412	-2.994027703	11.550859826	1.709376271
	N413	5.916109653	-4.338829991	-9.512655199
	N414	3.878059561	-2.244313897	-7.377507469
	N415	2.740369947	0.534970138	-6.891915840
	N416	4.817260990	2.492581525	-6.485515220
	N417	0.181764251	-2.532965236	-10.342134633
	N418	-1.645196892	-0.770627586	-8.994674978
	N419	9.048977817	-6.911234177	-4.703144037
	N420	4.970610714	-5.475453611	-3.687462534
	N421	4.710224093	-6.341952215	-5.700374109
	N422	9.658758528	-7.413469372	-1.672809369
	N423	0.099934781	1.999699626	0.296624991
	N424	0.958396606	1.412843203	-0.141004207

	O425	-6.767260118	-5.181286961	0.205460735
	O426	-7.950503990	-2.975492094	2.954494688
	O427	-6.439000784	-5.421448481	5.387295683
	O428	-3.289318798	-4.127690719	4.267773125
	O429	-3.949883535	-0.765652845	4.822470057
	O430	-4.921510326	-1.394558831	8.236397109
	O431	-2.358976623	-4.392279909	9.274104741
	O432	0.933986791	-3.982047917	6.300327170
	O433	0.603675141	-0.055180664	6.899648109
	O434	-0.973240644	1.445823321	9.778411400
	O435	-0.792980353	-0.789541872	12.511112192
	O436	6.576708501	-10.501770958	2.876112293
	O437	6.843970681	-8.040268584	6.802444944
	O438	10.533532205	-4.811609068	6.358313784
	O439	-5.549910528	-0.793190025	-1.052422105
	O440	-2.867738179	-4.151142226	-4.831855705
	O441	-6.901947248	2.602044364	-1.586053061
	O442	-5.574717322	3.278196665	-4.310966319
	O443	-10.021696378	1.481016183	0.509444322
	O444	-6.314334673	1.320348999	3.401777391
	O445	-4.865475942	4.931582579	3.157169399
	O446	-7.417466217	7.111879084	4.355762509
	O447	9.840048930	-0.368959720	6.000083185
	O448	2.738509975	4.430603880	4.734903128
	O449	3.687665800	8.358940918	0.392670986
	O450	1.638317129	10.204156736	-2.868215729
	O451	-1.109795685	7.249399734	-1.458379340
	O452	-1.108798676	7.081835430	2.298356778
	O453	2.089159329	5.562382342	2.112896925
	O454	0.412445454	11.452187529	2.977384936
	O455	-2.529270611	13.574785375	0.769967159
	O456	-2.931379032	11.094719597	-1.420549896
	O457	-5.500386082	13.595703841	3.269570406
	O458	-5.144057552	6.986735100	-2.025355534
	O459	3.349125888	-4.196897834	-8.465082482
	O460	1.332484819	-0.367399339	-8.460303266
	O461	3.697642070	3.930479966	-7.842881296
	O462	7.328784140	4.743353200	-7.741687308
	O463	-3.270431851	-2.178970539	-9.750056554
	O464	-0.333189711	-6.319174388	-6.460085737
	O465	1.683695331	-5.439093799	-6.821032886
	O466	-2.338906993	2.734767477	-8.956636236
	O467	10.433966775	-5.319839002	-2.062469577
	O468	12.785415881	-6.850017982	-0.013417089
	O469	0.559450159	-6.448737365	-1.607915170
	O470	-0.342831847	-7.404645896	0.217809808
	O471	5.639575907	-10.798754830	-2.780089493
	O472	3.431210148	-10.768583215	-3.264791300
	O473	5.234319934	-5.782834871	-0.559687558
	O474	4.927035937	-7.577983280	0.755724715
	O475	2.940767787	-6.004491247	-1.780024340
	O476	8.605565936	-6.676136577	2.377951456
	O477	6.803071208	-4.620304306	1.474611585
	O478	-3.828690074	4.921013990	-2.984263607
	O479	8.048433207	-10.387744325	-1.509271843
	O480	7.408588790	-8.690460886	0.914827712
	S481	3.889447677	5.007073147	-1.414003995
	S482	2.507267586	2.051557902	-2.681312650
	S483	2.679334549	-3.152928809	-3.168494324
	S484	3.752479417	1.649937457	0.603187415
	S485	0.749998132	-0.864143246	-1.956990626
	S486	4.950257790	-0.473742164	-4.667744379
	S487	3.539105581	-3.389395492	0.340886058
	S488	6.038198234	1.827176023	-2.146907931
	S489	6.190472240	-2.992619127	-2.110873744
	S490	6.640096385	-0.810097009	0.574616240
	end			

TS for dissociation of 2N2x

Fe(139)	-2.478	6n2xs2bh26h2n2xdte.car_1			
Fe(140)	0.484	C1	-7.844176850	-5.384305534	-0.379418761
Fe(141)	-2.489	C2	-8.019062112	-6.479024845	-1.436011984
Fe(142)	2.664	C3	-8.910274431	-3.515931643	0.804606307
Fe(143)	2.380	C4	-8.539695530	-3.847025696	2.247859547
Fe(144)	0.329	C5	-8.468034847	-5.529778714	4.021376617
Fe(145)	0.030	C6	-6.955189990	-5.630809872	4.244727844
		C7	-4.743646008	-5.885594192	3.244959361
		C8	-4.214898235	-4.463142938	3.438688180
		C9	-4.484564277	-2.096760691	2.863983712

S = 1/2	C10 -4.743005635 -1.581610637 4.287610256<br>C11 -6.212380594 -1.497116647 6.207515317<br>C12 -5.359627766 -2.131406211 7.315375761<br>C13 -4.570269845 -4.221221509 8.328065990<br>C14 -3.056179153 -4.473669289 8.256114271<br>C15 -1.130194467 -5.035044336 6.826027722<br>C16 -0.271987632 -3.867067962 6.330421668<br>C17 -0.316618036 -1.579036919 5.395008937<br>C18 -0.398265765 -0.409528506 6.389387807<br>C19 -0.807700668 -1.240439528 3.966317354<br>C20 -0.200462549 0.071212179 3.461233832<br>C21 -0.500800887 -2.386483909 2.997471783<br>C22 -1.792882917 1.259051257 7.521636716<br>C23 -1.717938375 0.842443264 9.002211829<br>C24 -3.072041303 2.057661972 7.174942373<br>C25 -3.315268245 3.174267264 8.196450288<br>C26 -2.969616289 2.636522979 5.757574765<br>C27 -2.479055066 -0.622513403 10.775902129<br>C28 -1.086577290 -0.873913953 11.320173847<br>C29 5.091979901 -11.847298879 4.205192259<br>C30 5.829912643 -10.556101064 3.865307447<br>C31 6.015528493 -8.165337523 4.530271836<br>C32 7.071579908 -7.830980878 5.601078843<br>C33 4.818531097 -7.202368914 4.608693737<br>C34 5.225473889 -5.753137503 4.355950457<br>C35 4.024833322 -4.829112283 4.167053290<br>C36 4.157106735 -2.321953497 4.160557488<br>C37 9.281189283 -6.841187609 5.991941903<br>C38 9.462536570 -5.342401423 6.082767516<br>C39 -4.967452282 -2.535395284 -2.697973890<br>C40 -5.401870400 -1.122694817 -2.264005934<br>C41 -3.472997718 -2.711836983 -2.337711678<br>C42 -2.930850272 -4.154308902 -2.409909044<br>C43 -2.372137926 -4.573588786 -3.769800322<br>C44 -5.680630988 1.172628392 -3.122993606<br>C45 -6.923089355 1.605749935 -2.333351779<br>C46 -5.581993419 1.852289796 -4.495268763<br>C47 -9.238690016 1.085490080 -1.722529574<br>C48 -9.116068437 0.994902427 -0.197587920<br>C49 -7.739295189 0.353525104 1.725508474<br>C50 -6.615701472 1.274559587 2.201015282<br>C51 -4.970578373 3.005401465 1.664968058<br>C52 -5.521364035 4.281696558 2.339007395<br>C53 -4.153908170 3.499233162 0.461384115<br>C54 -3.443643315 2.453619375 -0.327796475<br>C55 -3.146976929 1.135588333 -0.090833965<br>C56 -2.258208254 1.689093352 -2.063014811<br>C57 -7.301599549 5.974871597 2.214586392<br>C58 -7.446921916 6.075457309 3.732318812<br>C59 -8.685039247 6.068309866 1.526201923<br>C60 -9.267439602 7.441792509 1.364160942<br>C61 -9.350112982 8.498615185 2.249459417<br>C62 -10.298181321 9.032717014 0.319207935<br>C63 8.491808320 1.432084672 5.086187370<br>C64 9.541642793 0.337422233 5.017971662<br>C65 7.160625638 0.811143821 4.596134997<br>C66 5.999186889 1.773106031 4.663393393<br>C67 5.210445892 1.891362620 5.822473069<br>C68 5.665495050 2.572970207 3.558173665<br>C69 4.117910332 2.764666122 5.874160199<br>C70 4.589244190 3.460300177 3.599796627<br>C71 3.808017820 3.546111128 4.754945629<br>C72 5.541273526 6.849217051 -0.092659236<br>C73 4.537364927 7.963925438 -0.422448386<br>C74 4.815317651 5.518557353 0.136712850<br>C75 3.841222220 9.642393637 -2.096452166<br>C76 2.428253129 9.284505749 -2.562375539<br>C77 0.831605248 7.394817497 -2.893501539<br>C78 0.089083840 6.970509450 -1.601018253<br>C79 1.053662944 6.211147005 -3.861461809<br>C80 -0.143815409 5.344742008 -4.263595515<br>C81 -1.117502484 5.937393622 -5.292940503<br>C82 -2.708285122 7.834567627 -5.019945486<br>C83 0.183392959 5.922174544 0.608263716<br>C84 -0.142862892 7.101645571 1.533994246<br>C85 1.045675526 4.924496885 1.412712095<br>C86 0.805740818 9.100991365 2.575540674<br>C87 0.241726046 10.470246451 2.232310635<br>C88 -0.832882968 11.853336999 0.535579199<br>C89 -2.204188867 12.394680560 1.008398099

C90	-0.700932748	11.948395461	-0.986756729
C91	-1.737544744	11.140676057	-1.758194070
C92	-4.381784375	11.869072352	2.003449550
C93	-4.482574232	12.913678246	3.105997049
C94	-5.143065142	10.580834808	2.425625794
C95	-5.193825406	9.562406739	1.309077476
C96	-6.211136935	9.625017539	0.343640935
C97	-4.200256558	8.581770484	1.159125561
C98	-6.223033061	8.761851622	-0.753930568
C99	-4.181174327	7.725620980	0.053552536
C100	-5.193749454	7.830669241	-0.895264430
C101	5.685548840	-3.613207366	-8.266834055
C102	4.200159237	-3.374832677	-8.059337501
C103	2.527983921	-1.880938598	-6.989302162
C104	2.131763463	-0.504140874	-7.537010476
C105	2.545989055	1.913562200	-7.333896944
C106	3.757839733	2.839788634	-7.263118394
C107	5.912846657	3.421848540	-6.305582560
C108	6.635292894	3.745705486	-7.597838592
C109	-1.007411962	-3.074033858	-9.675801170
C110	-2.066729918	-1.981678520	-9.498715711
C111	-0.778952659	-3.802570215	-8.330315961
C112	-0.016942446	-5.126059970	-8.530835468
C113	0.416431737	-5.699022216	-7.198657618
C114	-2.569119030	0.312628720	-8.838650382
C115	-1.960326766	1.607175046	-9.338779309
C116	-3.267149102	0.412361790	-7.459471223
C117	-2.233982406	0.422717872	-6.373343351
C118	-1.591784134	1.618799639	-6.012591123
C119	-1.743713541	-0.794607483	-5.871297508
C120	-0.420205962	1.584013353	-5.252429361
C121	-0.575084950	-0.826563837	-5.110756656
C122	0.113964870	0.360600290	-4.843019725
C123	8.658819163	-6.123344909	-3.524036830
C124	9.648075449	-6.244236678	-2.354686804
C125	7.235598385	-6.436622140	-3.036238282
C126	6.128174472	-6.187534291	-4.008759675
C127	5.973059619	-6.729469922	-5.267514143
C128	4.129713376	-5.637924593	-4.712513680
C129	10.531945197	-7.531961442	-0.504476654
C130	11.969215240	-7.199085979	-0.838052053
C131	0.643970041	-7.001779033	-0.386749086
C132	2.039064127	-6.966553304	0.236533051
C133	3.184208317	-7.023917601	-0.814530082
C134	3.224642616	-8.441498288	-1.453543073
C135	4.436638605	-8.745171379	-2.336720455
C136	4.516046863	-10.213475077	-2.810617431
C137	4.536567553	-6.771757444	-0.131529374
C138	3.923858065	-0.648950260	-1.561520819
Fe139	4.411869900	-1.851334389	-3.046659919
Fe140	2.477584882	0.477631118	-1.029172740
Fe141	5.220602995	0.481176642	-0.440662871
Fe142	4.320800636	0.715918480	-2.931480296
Fe143	4.036230407	2.691649300	-1.154431583
Fe144	4.955543381	-1.973238189	-0.562909615
Fe145	2.331912648	-2.218404328	-1.108618603
H146	-6.242980121	-7.439843730	-1.147703958
H147	-6.218234557	-6.393715383	-2.400283675
H148	-9.795439690	-4.792017927	-0.660420433
H149	-8.597682981	-6.072059772	-2.280886309
H150	-8.656817651	-7.256459625	-0.980752429
H151	-8.169763221	-2.780717636	0.455320018
H152	-9.294258385	-5.738088015	2.047211758
H153	-9.895486767	-3.030561890	0.803815456
H154	-8.917154744	-6.515823291	4.199860669
H155	-6.624471991	-5.957224278	2.234211855
H156	-8.851303817	-4.836774846	4.781434182
H157	-3.426202504	-1.911958484	2.641283987
H158	-5.537383656	-3.764995226	2.024991421
H159	-4.323950908	-6.312630805	2.323795207
H160	-6.057493958	-0.411812200	6.266232220
H161	-6.606906985	-2.470983168	4.322249749
H162	-4.389853395	-6.483157705	4.094177680
H163	-5.093957094	-1.521964119	2.153379559
H164	-5.637412175	-4.000493199	6.486989130
H165	-7.269765682	-1.714410342	6.412048518
H166	-0.933166544	-3.339080598	3.334014562
H167	-0.919364146	-2.169077544	2.002322472
H168	0.585367318	-2.516975950	2.870773116
H169	-1.899338441	-1.107856887	4.023001947

	H170	-5.067443416	-5.200959754	8.390768334
	H171	-2.854403335	1.852180352	4.996956255
	H172	-3.146252892	-0.656850835	8.751109446
	H173	-3.482555188	2.782829402	9.210112915
	H174	0.892272511	-0.006162605	3.347441947
	H175	-0.417653998	0.918944947	4.127213682
	H176	8.365520858	1.707374393	6.151570447
	H177	-3.873386388	3.210509090	5.507774883
	H178	-4.199686289	3.760180281	7.906895766
	H179	-2.105543188	3.318051798	5.685869264
	H180	-2.452653031	3.858307897	8.235745540
	H181	-3.929380917	1.361209119	7.210760859
	H182	-0.921645452	1.915595646	7.378786491
	H183	-2.444035419	-0.226112843	6.103498065
	H184	-0.620195094	0.307111367	2.474077979
	H185	1.475049035	4.188149710	0.716437841
	H186	0.367151478	4.410231338	2.106413792
	H187	2.170117936	5.147260626	3.004904247
	H188	2.243102442	4.361917835	5.568807870
	H189	4.329049331	4.059731491	2.727768839
	H190	6.237442064	2.479279758	2.632810584
	H191	5.440453228	1.279864034	6.699007261
	H192	-1.973137775	-2.833659916	5.896785767
	H193	-1.019559109	-5.855155255	6.101204788
	H194	-0.703370322	-5.369233651	7.780431003
	H195	-3.140723328	-4.739442867	6.193525191
	H196	-4.744509571	-3.684341030	9.268301189
	H197	3.502277114	2.823665818	6.775871753
	H198	0.750964877	-1.815429730	5.341256514
	H199	-2.981801023	0.118542146	11.419359268
	H200	-3.030689076	-1.573078919	10.849637895
	H201	-0.332076769	-1.194243387	10.563142496
	H202	10.230224496	-7.305263990	5.682460765
	H203	-0.971080368	-5.786539294	-4.637782162
	H204	1.564082651	-6.092286015	-1.824437650
	H205	-0.677523673	-5.857536579	-9.018128090
	H206	0.857376288	-4.964500890	-9.177648098
	H207	-1.744939633	-4.013895108	-7.846082196
	H208	-1.169070571	1.472112600	-10.112011247
	H209	-1.482200319	-3.790358319	-10.362196518
	H210	-2.875547910	-2.060193521	-2.998343136
	H211	-3.731597932	-4.869636649	-2.153908313
	H212	-0.649532353	-0.650029051	-8.756594576
	H213	-3.372516061	0.107016912	-9.576377013
	H214	0.769671479	-1.996698289	-9.716851736
	H215	-0.899256790	-5.762740726	-2.882005299
	H216	-0.227903713	-3.134525930	-7.650316152
	H217	0.760864752	-3.326744366	-10.689578918
	H218	-6.087429298	-3.231095232	-4.285081674
	H219	-3.347924375	-2.358731993	-1.302737804
	H220	2.202442648	-4.938491013	-7.510433650
	H221	-2.155584909	-4.290039194	-1.642803890
	H222	-4.324326675	2.519657817	2.409754828
	H223	-3.401373312	0.480497930	0.733394733
	H224	-8.653853301	0.673044135	2.244724483
	H225	-7.313009715	-0.032930400	-0.330568002
	H226	-7.236277322	4.073341561	1.233699859
	H227	-8.992715577	8.631849871	3.264485315
	H228	-7.656834852	5.096990229	4.228686590
	H229	-9.391213515	5.400851541	2.051070787
	H230	-6.646544124	6.789852424	1.868741927
	H231	-10.816214100	9.632132378	-0.425562832
	H232	-3.405571078	4.209323173	0.848741644
	H233	-4.816914901	4.065859328	-0.211657973
	H234	-10.222063724	10.425115664	1.927994110
	H235	-8.572240345	5.660089894	0.509702671
	H236	-6.337777953	2.065206246	0.291013655
	H237	-7.515624161	-0.664819586	2.075634424
	H238	-9.613945713	2.095772442	-1.936431557
	H239	-1.716852846	1.626309773	-3.004059225
	H240	-10.002500971	0.369624672	-2.052143919
	H241	9.654228511	3.068563874	4.674696554
	H242	8.125666191	3.194637433	4.111819553
	H243	1.772674008	5.972681328	-0.843812516
	H244	-0.780336872	5.443624231	0.375668472
	H245	1.629390093	7.976258254	0.986425302
	H246	-3.070613826	3.666829815	-2.075519435
	H247	-4.474740693	4.362356646	-3.483125704
	H248	-5.748641770	3.676714120	-5.193033285
	H249	-4.350825261	5.575838450	-2.458351160

	H250	-4.654216695	1.508283724	-4.982290957
	H251	-6.442005676	1.563665717	-5.119054978
	H252	0.323799551	11.684894991	-1.285035250
	H253	-0.859586802	13.004415965	-1.257299976
	H254	-0.258936247	10.382887909	-3.002749777
	H255	-1.919853981	9.891608211	-3.331209806
	H256	-2.523066443	9.446865705	-6.276262130
	H257	-1.512338570	5.135489128	-5.935765235
	H258	-7.030664216	8.812875658	-1.488459101
	H259	-3.414802307	8.463602765	1.910060634
	H260	-3.967158337	7.995379336	-3.397186508
	H261	1.799643452	5.544733819	-3.398791027
	H262	2.893825883	7.314950973	-2.364687289
	H263	-0.698537651	4.997131746	-3.377885433
	H264	-7.948538786	-0.009624299	-3.044305066
	H265	4.348232746	10.162240968	-2.919116244
	H266	-5.414498514	-0.726107972	-4.216761673
	H267	1.527444364	6.617108338	-4.770080429
	H268	-3.360392385	7.019010511	-0.079509852
	H269	3.718242759	10.358245304	-1.270161246
	H270	-1.737374367	8.005490721	-6.832111163
	H271	-4.219018937	9.161753185	-4.651172317
	H272	-4.436440081	-3.276722308	-4.543269296
	H273	-5.544936686	-3.231299926	-2.061794025
	H274	-7.012839907	10.359843426	0.447818415
	H275	-4.843507414	1.523042148	-2.504212597
	H276	0.211726997	8.172687160	-3.352644879
	H277	-0.575407770	6.635520772	-5.943227568
	H278	0.283618861	4.444489194	-4.724517369
	H279	-2.799639160	6.082278071	-3.987752905
	H280	0.240934321	8.719017968	3.438843047
	H281	-0.456301423	9.731572844	0.458172969
	H282	-0.117399909	12.570760503	0.966975206
	H283	1.848900344	9.250509838	2.886218827
	H284	-2.707974367	10.565581361	1.722585717
	H285	6.217518780	-2.650879521	-8.305741658
	H286	6.031485525	-4.133617128	-7.349522958
	H287	8.706906723	-5.068816351	-3.833062124
	H288	6.883125556	-4.696970920	-9.508595563
	H289	5.322655659	-5.188429781	-9.504096628
	H290	5.572285475	4.760680091	0.376122594
	H291	5.663367857	7.386519081	1.875570952
	H292	5.407275413	8.202095476	-2.272423358
	H293	4.114467786	5.623356069	0.976256822
	H294	6.865397168	8.016620061	0.960545448
	H295	6.645554859	2.952664392	-5.630456151
	H296	6.250225861	6.731177601	-0.927587994
	H297	4.333458643	-7.310239102	5.593386006
	H298	6.465646112	-8.096409483	3.531624404
	H299	4.832045042	1.617091444	-5.948258715
	H300	4.371172333	-0.309216902	3.858133691
	H301	2.190580337	1.914801304	-8.371278208
	H302	-0.188660987	-1.779595086	-4.743380897
	H303	1.758626195	2.391948267	-6.725752248
	H304	3.479176352	-4.707352571	5.110576670
	H305	3.294913765	0.311221997	-6.076673112
	H306	3.323498498	-5.253383799	3.430281476
	H307	4.096441016	-7.525737000	3.841804825
	H308	5.842137956	-5.704831830	3.446874553
	H309	5.841246988	-5.369301689	5.184735635
	H310	-3.943059213	-0.449700925	-7.361631864
	H311	4.924972220	-9.774699123	5.469691028
	H312	4.302549318	-11.952463099	3.445704380
	H313	1.061119786	0.346820320	-4.308284977
	H314	5.133695886	-12.189402696	6.235297596
	H315	3.665455070	-12.453266287	5.559686423
	H316	5.134684871	-3.562949548	2.891785667
	H317	0.102051474	2.504861038	-4.993002654
	H318	-2.261758195	-1.727562804	-6.104582413
	H319	7.838771974	-9.918660013	-0.664266086
	H320	7.189820673	-7.498940264	-2.741007634
	H321	6.543399745	-8.221914809	0.774864523
	H322	7.184526745	-10.467090163	-1.961808919
	H323	6.533491209	2.978225747	-8.401249160
	H324	6.298324885	-5.046333880	0.775171323
	H325	2.129117609	-7.779602717	0.967073573
	H326	4.635740454	-1.691786574	-6.981985944
	H327	3.203249723	-9.159913378	-0.618983998
	H328	4.263193821	-6.651860167	-6.554857926
	H329	3.119514346	-5.256374376	-4.781233282

H330	5.802120887	-12.674828490	4.039164067
H331	5.356365756	-1.281066909	2.821159506
H332	8.076595888	-5.895709321	2.084963277
H333	7.133398557	-3.781579684	1.100012623
H334	8.302852109	-7.065597801	4.097540110
H335	2.683594111	-2.940058403	5.513284290
H336	4.399726469	-8.111740163	-3.235630663
H337	5.367789044	-8.500504856	-1.807862331
H338	2.298563020	-8.590941226	-2.029375319
H339	2.462371519	-1.894705973	-5.889626875
H340	-1.981419693	2.569099922	-6.384489992
H341	-3.872526882	1.331184258	-7.466214551
H342	2.109643403	-6.011988110	0.785816124
H343	8.251915496	-7.423507674	1.824156181
H344	7.169366648	-9.353401646	1.622465082
H345	3.129285539	-1.266101180	5.588071915
H346	6.635320647	-7.347615491	-5.858394523
H347	7.040361256	-5.861894314	-2.122880402
H348	1.840848616	-2.622122171	-7.401752296
H349	5.576663896	4.360441733	-5.837713171
H350	6.953521821	-0.071524106	5.218423571
H351	7.299148256	0.465537983	3.559008962
H352	9.984388083	-6.683841607	-4.985058677
H353	9.087447395	-7.932036797	-4.422157771
H354	10.511811775	-8.583522425	-0.177743724
H355	9.020182533	-8.187678342	-1.857156342
H356	12.256423195	-7.379575395	-1.901736024
H357	10.192699823	-6.912587904	0.341462864
H358	10.002174310	0.206513805	4.008496912
H359	-3.585719418	12.994795458	3.765306692
H360	9.044953416	-7.200672685	7.008618931
H361	8.539506802	-4.739950063	5.894929010
H362	-4.860503046	12.290537673	1.103768648
H363	-4.661916438	10.152378907	3.318749826
H364	-6.160627655	10.881858921	2.713333308
N365	0.890843460	-3.171786244	-0.531962996
H366	4.408954131	-1.295625919	0.654724241
H367	-6.005042567	7.019243815	-2.471737900
H368	-0.007035755	-0.758779718	-0.874519570
H369	2.196015679	-1.000525378	0.010708800
N370	-0.016196850	-3.722645814	-0.152505920
H371	-2.030961908	-0.240301413	-1.296097947
Mo372	4.196077233	-4.262618009	-1.792968773
N373	-6.800222463	-7.093213209	-1.932875345
N374	-8.965979086	-4.664761620	-0.085985995
N375	-8.845321166	-5.088906027	2.689081919
N376	-6.196266865	-5.934820905	3.161508158
N377	-4.806750801	-3.503761133	2.683244963
N378	-5.898567291	-1.975978364	4.870635346
N379	-5.175228695	-3.474592518	7.234257774
N380	-2.540441537	-4.743842737	7.023173139
N381	-0.957335305	-2.770599836	5.928909594
N382	-1.632225572	0.111982870	6.627019868
N383	-2.463869124	-0.226920511	9.380155080
N384	4.475881334	-11.832538317	5.538810305
N385	5.550755596	-9.531528990	4.694511758
N386	4.483578184	-3.525730538	3.682346337
N387	3.266084240	-2.177036825	5.160749693
N388	4.758271258	-1.226211090	3.655728162
N389	8.190634403	-7.232283083	5.122019216
N390	-5.212610689	-2.726205740	-4.131583557
N391	-1.354886320	-5.453798627	-3.746318255
N392	-5.575546540	-0.265877895	-3.304491075
N393	-8.019893265	0.828541841	-2.471452738
N394	-8.016062463	0.365075685	0.301242979
N395	-6.013325074	2.069671659	1.262889852
N396	-2.880287887	2.764692574	-1.559860893
N397	-2.418428372	0.695247353	-1.178821514
N398	-6.731510625	4.680201837	1.874691225
N399	-9.862616428	7.787954409	0.161976428
N400	-10.005285835	9.500628105	1.565087281
N401	8.905188132	2.539909931	4.220859128
N402	6.317211403	7.166173815	1.117150370
N403	4.663521858	8.528741538	-1.660906876
N404	2.141575862	7.967057704	-2.606405706
N405	-2.253653354	6.629829260	-4.676803417
N406	-2.235444965	8.490758281	-6.094723584
N407	-3.607562190	8.466252919	-4.230973313
N408	0.819181683	6.329574949	-0.645664261
N409	0.758278056	8.123355692	1.503964198

	N410	-0.463326335	10.552197344	1.063317971
	N411	-1.268313865	10.532496311	-2.881348559
	N412	-2.991258124	11.542425553	1.712572486
	N413	5.919266546	-4.356238796	-9.506894472
	N414	3.879836808	-2.244599550	-7.393989954
	N415	2.744350240	0.533785051	-6.925817801
	N416	4.818438351	2.484056589	-6.495407037
	N417	0.185891908	-2.551376691	-10.353840391
	N418	-1.637096742	-0.787004297	-8.994372398
	N419	9.039442511	-6.943495322	-4.688224845
	N420	4.963504014	-5.495286242	-3.681313409
	N421	4.713499314	-6.368255681	-5.689471155
	N422	9.666171958	-7.416898084	-1.664908866
	N423	-0.392984156	2.454125490	0.816231261
	N424	0.262959535	1.833808863	0.171302665
	O425	-6.770179178	-5.183753065	0.216030436
	O426	-7.983738824	-2.992663281	2.961524545
	O427	-6.479937862	-5.458007006	5.379154541
	O428	-3.311410986	-4.202707536	4.248577590
	O429	-3.928655963	-0.841790157	4.862432844
	O430	-4.915702307	-1.437986311	8.247267025
	O431	-2.385945152	-4.478457223	9.296661931
	O432	0.968446999	-3.974117331	6.301935205
	O433	0.631654798	0.023163005	6.931113534
	O434	-1.004533033	1.456921596	9.813653478
	O435	-0.812667132	-0.805242056	12.509048160
	O436	6.588721139	-10.511188923	2.875361948
	O437	6.870832049	-8.081096410	6.802354726
	O438	10.525335458	-4.806485681	6.356166502
	O439	-5.550485722	-0.793367418	-1.070780324
	O440	-2.856440087	-4.140030906	-4.840939011
	O441	-6.886389430	2.607422290	-1.596379058
	O442	-5.561304461	3.283273574	-4.321983539
	O443	-10.007520272	1.486569190	0.507425773
	O444	-6.286550912	1.283414768	3.390460531
	O445	-4.855244388	4.927023937	3.153210712
	O446	-7.395525972	7.116850421	4.363048623
	O447	9.859409977	-0.373968032	5.959756167
	O448	2.732994121	4.415771344	4.727642033
	O449	3.673768198	8.341633925	0.387450561
	O450	1.636985102	10.197543615	-2.868535210
	O451	-1.120348998	7.220363010	-1.467275986
	O452	-1.119535501	7.065167635	2.293394958
	O453	2.087099936	5.578900630	2.130567820
	O454	0.409546130	11.432399464	2.991872693
	O455	-2.517713363	13.573212814	0.791503439
	O456	-2.927154217	11.096556265	-1.415153174
	O457	-5.481618555	13.591432752	3.289011166
	O458	-5.134132324	6.995360827	-2.029878958
	O459	3.354503603	-4.211755535	-8.457180602
	O460	1.328425396	-0.374206721	-8.483823037
	O461	3.724394216	3.923409648	-7.871373114
	O462	7.321444067	4.744022050	-7.750197254
	O463	-3.259201399	-2.182150007	-9.781322737
	O464	-0.335494928	-6.327547383	-6.460292418
	O465	1.681342259	-5.442728809	-6.811424849
	O466	-2.321048440	2.722582029	-8.991170430
	O467	10.432043793	-5.321221690	-2.059144538
	O468	12.791869861	-6.835495119	-0.009155182
	O469	0.545696976	-6.407955186	-1.580628007
	O470	-0.326783890	-7.497338220	0.184333415
	O471	5.663008080	-10.782052539	-2.760583507
	O472	3.455111477	-10.743306359	-3.246562372
	O473	5.239139585	-5.761652456	-0.579029979
	O474	4.931077841	-7.519779314	0.783043082
	O475	2.930447331	-5.998534244	-1.775473796
	O476	8.606983452	-6.675395353	2.380356445
	O477	6.794142673	-4.600886036	1.510379420
	O478	-3.822060542	4.926901209	-2.987397903
	O479	8.071862490	-10.381709455	-1.499402211
	O480	7.405734853	-8.692343066	0.922924050
	S481	3.836551331	4.953835314	-1.335368621
	S482	2.502304296	2.045644325	-2.633534127
	S483	2.604543364	-3.200688134	-3.177752664
	S484	3.581901559	1.536007225	0.696221630
	S485	0.789838542	-0.725250562	-1.979299863
	S486	4.933373146	-0.472508088	-4.704664535
	S487	3.576658755	-3.403328549	0.333541034
	S488	6.012112939	1.890828583	-1.961190889
	S489	6.149284236	-2.989766268	-2.199528427

	S490 6.630862993 -0.902476115 0.525902978<br>end

S2BH-2N2x-7Hn-6N2x (17)

Fe(139) -2.339<br>Fe(140) -0.525<br>Fe(141) -2.529<br>Fe(142) 2.681<br>Fe(143) 2.496<br>Fe(144) 1.386<br>Fe(145) 0.856<br><br>S=1	2n2xs2bh6n2xdtb.car_3
	C1 -7.853151593 -5.389689808 -0.377550289<br>C2 -8.027405878 -6.481528921 -1.437285086<br>C3 -8.908589429 -3.516320229 0.805650299<br>C4 -8.534142931 -3.846273894 2.248091060<br>C5 -8.457060170 -5.527690704 4.023301338<br>C6 -6.944046777 -5.626292424 4.245400112<br>C7 -4.732227861 -5.876212169 3.247056041<br>C8 -4.209032192 -4.451404788 3.440495732<br>C9 -4.486516376 -2.086507191 2.859551163<br>C10 -4.744465364 -1.571138432 4.283201752<br>C11 -6.210692628 -1.490027303 6.205625122<br>C12 -5.358189914 -2.122790909 7.314564645<br>C13 -4.567976397 -4.210730439 8.330581434<br>C14 -3.054722908 -4.466544728 8.256164719<br>C15 -1.135962619 -5.045689790 6.824384048<br>C16 -0.281269887 -3.874813165 6.330498532<br>C17 -0.333671436 -1.589224700 5.398091892<br>C18 -0.409248319 -0.419371701 6.390826781<br>C19 -0.816657883 -1.247938831 3.964481627<br>C20 -0.209352702 0.067003286 3.462930249<br>C21 -0.502882846 -2.391578144 2.993433508<br>C22 -1.794335632 1.253484484 7.524039888<br>C23 -1.717123122 0.840774720 9.006546020<br>C24 -3.069837802 2.056433537 7.175004202<br>C25 -3.309950110 3.173801258 8.196239945<br>C26 -2.964530110 2.634745264 5.757722793<br>C27 -2.481010028 -0.613387785 10.789453202<br>C28 -1.087445549 -0.869796110 11.328801725<br>C29 5.093079940 -11.841861706 4.206858960<br>C30 5.831926366 -10.549856900 3.866665729<br>C31 6.013271268 -8.157698777 4.528681330<br>C32 7.069367680 -7.820971197 5.599151464<br>C33 4.815622360 -7.195197256 4.606381223<br>C34 5.220158841 -5.745020294 4.352582323<br>C35 4.015276872 -4.827413698 4.158977854<br>C36 4.144685273 -2.320502354 4.173461573<br>C37 9.280479547 -6.834647811 5.990202099<br>C38 9.464404562 -5.336246744 6.081197098<br>C39 -4.978428425 -2.537225578 -2.694494538<br>C40 -5.405995011 -1.122914616 -2.259716244<br>C41 -3.484225260 -2.714718440 -2.337236617<br>C42 -2.943328459 -4.155978399 -2.415047812<br>C43 -2.379797983 -4.569170998 -3.774364929<br>C44 -5.678267968 1.173658035 -3.117339410<br>C45 -6.922711595 1.606834291 -2.330028536<br>C46 -5.582156911 1.851002006 -4.490853674<br>C47 -9.238766205 1.083915091 -1.721309298<br>C48 -9.116708200 0.994407254 -0.196278792<br>C49 -7.736936278 0.359354837 1.726461728<br>C50 -6.613600268 1.282636937 2.200296809<br>C51 -4.968328325 3.014212620 1.670874278<br>C52 -5.527390855 4.290738636 2.341071899<br>C53 -4.135037878 3.509392436 0.478203866<br>C54 -3.387758126 2.473003136 -0.290536975<br>C55 -3.061309156 1.164291226 -0.043232148<br>C56 -2.207545112 1.711350277 -2.030359889<br>C57 -7.317881278 5.977820163 2.220682241<br>C58 -7.461322381 6.076259276 3.738680004<br>C59 -8.697860980 6.068369395 1.526474186<br>C60 -9.278174375 7.442404760 1.361663969<br>C61 -9.356312229 8.500957201 2.245138396<br>C62 -10.302770033 9.035774960 0.314277850<br>C63 8.490789254 1.430886221 5.087867530<br>C64 9.539633040 0.335493797 5.023657131<br>C65 7.158961537 0.809370429 4.599893235<br>C66 5.998101020 1.772750996 4.667491168<br>C67 5.209702372 1.891212392 5.826749950<br>C68 5.667063116 2.576107517 3.563916636<br>C69 4.119485259 2.767166077 5.879278004<br>C70 4.593526307 3.466515428 3.607034619<br>C71 3.811542113 3.551541334 4.761581607<br>C72 5.553607740 6.851923686 -0.091736805

	C73	4.550028832	7.971564059	-0.420060326
	C74	4.822681011	5.527449595	0.110349343
	C75	3.845554456	9.649463185	-2.094272467
	C76	2.431846942	9.291776232	-2.560938794
	C77	0.831106841	7.405616365	-2.892680571
	C78	0.088602352	6.981808864	-1.600422969
	C79	1.049072187	6.220703319	-3.861294662
	C80	-0.150123169	5.354612410	-4.261730985
	C81	-1.123523282	5.945797904	-5.292156387
	C82	-2.714119833	7.843804733	-5.021251190
	C83	0.176547299	5.925851679	0.605330546
	C84	-0.142465324	7.105900191	1.533886622
	C85	1.025686492	4.913461159	1.401379930
	C86	0.806050812	9.108443641	2.571654268
	C87	0.240318368	10.479162787	2.231549256
	C88	-0.837470395	11.864675104	0.537193872
	C89	-2.210085817	12.407537457	1.006958771
	C90	-0.703731811	11.957104618	-0.985451912
	C91	-1.737676301	11.145506138	-1.756650691
	C92	-4.388060679	11.882610794	2.004617053
	C93	-4.488957380	12.925370332	3.108601100
	C94	-5.145720358	10.590875767	2.426811913
	C95	-5.194742268	9.571762662	1.310355502
	C96	-6.213499816	9.632544421	0.346251633
	C97	-4.200863787	8.591361363	1.159939011
	C98	-6.226414811	8.768480595	-0.750503777
	C99	-4.183004300	7.733968986	0.055077328
	C100	-5.196733678	7.837692644	-0.892892920
	C101	5.685561700	-3.608800387	-8.254527047
	C102	4.198876735	-3.372335315	-8.053302122
	C103	2.523170990	-1.883782826	-6.983406514
	C104	2.127205117	-0.503689422	-7.522292233
	C105	2.544069852	1.910957673	-7.311472670
	C106	3.756920727	2.835674012	-7.247084789
	C107	5.915068371	3.419363434	-6.299503535
	C108	6.639206710	3.741672833	-7.591018014
	C109	-1.007794302	-3.070115833	-9.675139506
	C110	-2.069872455	-1.979781661	-9.501325948
	C111	-0.780771183	-3.799168854	-8.330146631
	C112	-0.018173640	-5.121602640	-8.532378760
	C113	0.416144481	-5.695846585	-7.200808080
	C114	-2.575258177	0.316440408	-8.837895225
	C115	-1.972845893	1.613498941	-9.340973580
	C116	-3.237204344	0.408647348	-7.441004638
	C117	-2.196168258	0.398399162	-6.358289713
	C118	-1.538986414	1.582867234	-5.984513629
	C119	-1.742304815	-0.825042774	-5.836872740
	C120	-0.402441250	1.530907459	-5.173154046
	C121	-0.608986989	-0.874503728	-5.023029183
	C122	0.084386555	0.302041078	-4.721433983
	C123	8.668921040	-6.121519656	-3.533506000
	C124	9.648743886	-6.237781762	-2.357303274
	C125	7.244621582	-6.446153671	-3.057614808
	C126	6.147695625	-6.208767241	-4.043845001
	C127	6.005702258	-6.762464229	-5.299260770
	C128	4.157010141	-5.667260136	-4.770902836
	C129	10.523870351	-7.520348398	-0.499931863
	C130	11.962270842	-7.192540154	-0.832760660
	C131	0.653996962	-7.045365992	-0.407013633
	C132	2.047586536	-6.990644773	0.211044740
	C133	3.197977210	-7.063154511	-0.831893570
	C134	3.242223283	-8.484471930	-1.457731133
	C135	4.450751464	-8.778828372	-2.346665826
	C136	4.533048854	-10.244303387	-2.826259722
	C137	4.544257577	-6.806812872	-0.131458001
	C138	3.910475174	-0.641962750	-1.553889233
	Fe139	4.428018544	-1.870660510	-2.995934805
	Fe140	2.437244717	0.768763820	-0.964544816
	Fe141	5.266545871	0.495104840	-0.552545537
	Fe142	4.320633361	0.682573863	-2.955084878
	Fe143	4.206983068	2.767178883	-1.307101577
	Fe144	4.869052755	-2.000896789	-0.484722950
	Fe145	2.330116498	-2.392055905	-1.258258770
	H146	-6.249669885	-7.439048165	-1.148500240
	H147	-6.226413177	-6.392125446	-2.400147512
	H148	-9.801620024	-4.786099298	-0.659483196
	H149	-8.604316904	-6.072899375	-2.282621506
	H150	-8.665850988	-7.261253985	-0.986925309
	H151	-8.164736823	-2.786624962	0.452406376
	H152	-9.291828549	-5.736975238	2.052509497

H153	-9.891081565	-3.025181853	0.808436328
H154	-8.904871129	-6.514181882	4.202649949
H155	-6.612744165	-5.956106989	2.235301666
H156	-8.840552758	-4.834451341	4.783059507
H157	-3.429389348	-1.897043986	2.634393540
H158	-5.531195100	-3.760829515	2.022835262
H159	-4.309955934	-6.301971119	2.326393492
H160	-6.055737271	-0.404593254	6.262704664
H161	-6.604182753	-2.468159721	4.322569822
H162	-4.376474747	-6.471779476	4.096856901
H163	-5.098858859	-1.515664662	2.148148265
H164	-5.634190107	-3.992375696	6.488360583
H165	-7.268078819	-1.707080444	6.410404434
H166	-0.932407435	-3.346403904	3.326962223
H167	-0.921364616	-2.174024136	1.997527984
H168	0.584394070	-2.518800303	2.869467947
H169	-1.908611011	-1.116790562	4.015424465
H170	-5.066131978	-5.189787552	8.395598791
H171	-2.851195183	1.850054865	4.997560282
H172	-3.147914216	-0.657165465	8.764259427
H173	-3.477669508	2.782699335	9.210185787
H174	0.884437869	-0.008870454	3.354417201
H175	-0.427394071	0.910718498	4.134011096
H176	8.362382138	1.710194313	6.152692819
H177	-3.867107056	3.209925366	5.507197186
H178	-4.192908254	3.762041496	7.906607009
H179	-2.099361429	3.314587217	5.686196546
H180	-2.445300157	3.855613475	8.235033981
H181	-3.929099417	1.362458044	7.209813186
H182	-0.919958768	1.905154543	7.377499665
H183	-2.452856662	-0.227719932	6.104992875
H184	-0.621407690	0.317387616	2.473604775
H185	1.445882363	4.178096499	0.699691962
H186	0.345608877	4.392707562	2.090872154
H187	2.156858086	5.140495563	2.999222927
H188	2.246213975	4.366074068	5.574797103
H189	4.333518186	4.065702160	2.735004660
H190	6.238532613	2.481316651	2.638448980
H191	5.438446815	1.278024784	6.702338993
H192	-1.987256412	-2.853111073	5.884917231
H193	-1.026671565	-5.864221017	6.097605761
H194	-0.706339243	-5.380875999	7.777161682
H195	-3.146640675	-4.747317770	6.195898633
H196	-4.740178948	-3.671151221	9.269666175
H197	3.504198769	2.826426842	6.780835333
H198	0.733877444	-1.826377824	5.345207658
H199	-2.979090871	0.131915407	11.431747341
H200	-3.036104714	-1.561717588	10.868038967
H201	-0.338251132	-1.193191945	10.568297132
H202	10.228645378	-7.300194006	5.679446682
H203	-0.975368672	-5.779264794	-4.643820739
H204	1.548442401	-6.163960682	-1.868971948
H205	-0.677606962	-5.854316551	-9.019995156
H206	0.856406936	-4.959592596	-9.179081940
H207	-1.747481863	-4.011403997	-7.848495328
H208	-1.194433764	1.481722061	-10.128058880
H209	-1.480096659	-3.786089749	-10.363374277
H210	-2.890454515	-2.061377933	-2.998565723
H211	-3.743460323	-4.873376048	-2.163955699
H212	-0.653800461	-0.643539426	-8.768769072
H213	-3.393268276	0.110731782	-9.558546511
H214	0.770379862	-1.992230632	-9.711847851
H215	-0.913218640	-5.770861973	-2.889509523
H216	-0.230397159	-3.130416214	-7.650054091
H217	0.761201477	-3.321012017	-10.687475400
H218	-6.097149093	-3.234850874	-4.284195701
H219	-3.357706608	-2.362400048	-1.302811066
H220	2.204046621	-4.939141079	-7.514062290
H221	-2.170403300	-4.295287331	-1.647183521
H222	-4.329621097	2.530506982	2.423210038
H223	-3.279070187	0.520079019	0.798261676
H224	-8.651522178	0.678506614	2.245941271
H225	-7.312494992	-0.031250588	-0.328649501
H226	-7.241785493	4.078932935	1.234594055
H227	-8.996811610	8.634172463	3.259406333
H228	-7.665047194	5.096747533	4.235004668
H229	-9.407072043	5.400533250	2.046881130
H230	-6.665434795	6.795993128	1.876700753
H231	-10.818908980	9.635760578	-0.431508859
H232	-3.419082874	4.247661907	0.876144069

	H233	-4.794870608	4.047395996	-0.221039739
	H234	-10.222405825	10.429709170	1.921618233
	H235	-8.578430303	5.660886213	0.510461824
	H236	-6.326431362	2.069009017	0.290146512
	H237	-7.512783769	-0.658086405	2.079091180
	H238	-9.615425363	2.093555774	-1.935743020
	H239	-1.682864056	1.644295519	-2.981214864
	H240	-10.001535446	0.366746857	-2.050815036
	H241	9.655235753	3.064565792	4.667981486
	H242	8.124594127	3.191765106	4.110549499
	H243	1.768305467	5.983115731	-0.848545751
	H244	-0.788022069	5.452370528	0.370011715
	H245	1.629502049	7.981279604	0.985364041
	H246	-3.047423052	3.671031251	-2.053962988
	H247	-4.483909269	4.362828192	-3.484265435
	H248	-5.751943310	3.671856315	-5.195491114
	H249	-4.363784007	5.578175306	-2.461902208
	H250	-4.652580740	1.511068658	-4.977285126
	H251	-6.440288977	1.555400630	-5.114542507
	H252	0.321601777	11.693177638	-1.281076538
	H253	-0.862521707	13.012418264	-1.259399061
	H254	-0.255684131	10.388027764	-2.997082755
	H255	-1.914795012	9.891102923	-3.325915762
	H256	-2.521883198	9.459954701	-6.272908438
	H257	-1.517857574	5.142519279	-5.933003161
	H258	-7.035469544	8.818012153	-1.483652410
	H259	-3.414158800	8.473627565	1.909709113
	H260	-3.973326703	8.002708714	-3.399430793
	H261	1.794278750	5.553364046	-3.398790453
	H262	2.894461488	7.320207197	-2.366350705
	H263	-0.705340409	5.011226809	-3.374741504
	H264	-7.946308243	-0.010291274	-3.040779140
	H265	4.351552791	10.171093252	-2.916927603
	H266	-5.418486421	-0.725472850	-4.212667506
	H267	1.523008816	6.625351130	-4.770711945
	H268	-3.362512888	7.027014770	-0.078221108
	H269	3.721088150	10.365383999	-1.268166360
	H270	-1.734518779	8.019364421	-6.829814000
	H271	-4.225740926	9.170797560	-4.651862238
	H272	-4.446193772	-3.275393582	-4.542080618
	H273	-5.557665427	-3.232309098	-2.058637146
	H274	-7.015749120	10.366539628	0.451400750
	H275	-4.840458063	1.523270935	-2.497907995
	H276	0.212726853	8.184878938	-3.351840125
	H277	-0.581158014	6.642565314	-5.943886209
	H278	0.276210725	4.451965071	-4.720069348
	H279	-2.810248600	6.090172255	-3.992423564
	H280	0.243423596	8.727102583	3.436707628
	H281	-0.461395471	9.743395669	0.458058214
	H282	-0.122822105	12.583069182	0.968699074
	H283	1.849568789	9.259309319	2.881218782
	H284	-2.713623384	10.580710655	1.726802031
	H285	6.215696290	-2.645059280	-8.289980403
	H286	6.028937500	-4.131981629	-7.337113765
	H287	8.711244719	-5.066297538	-3.842197531
	H288	6.884677842	-4.688150599	-9.499643782
	H289	5.323590896	-5.178070651	-9.499516586
	H290	5.570130735	4.763418625	0.357673380
	H291	5.658357703	7.375118071	1.880875741
	H292	5.407540004	8.206057069	-2.275812657
	H293	4.102421902	5.618950274	0.935876003
	H294	6.867646452	8.013760527	0.981159171
	H295	6.646096632	2.955515827	-5.619057532
	H296	6.268172915	6.741941455	-0.922474407
	H297	4.330570209	-7.302866877	5.591149388
	H298	6.462748023	-8.089608930	3.529821588
	H299	4.839210805	1.610913246	-5.943161070
	H300	4.373386506	-0.303306273	3.909326089
	H301	2.184426724	1.912161513	-8.347087046
	H302	-0.256510720	-1.832950790	-4.634798329
	H303	1.760510774	2.389559885	-6.699318445
	H304	3.465037268	-4.711063581	5.100350261
	H305	3.287416600	0.303117723	-6.056725447
	H306	3.320131216	-5.256471520	3.419494702
	H307	4.094027291	-7.519818091	3.839622627
	H308	5.839362545	-5.696522067	3.445310290
	H309	5.832458167	-5.358850354	5.182886314
	H310	-3.920147350	-0.446972330	-7.336551043
	H311	4.923471152	-9.767299646	5.468139321
	H312	4.302664611	-11.943861753	3.447614858

	H313	0.995898276	0.275639311	-4.128153845
	H314	5.137336363	-12.179550857	6.237778421
	H315	3.668681478	-12.446954994	5.563146397
	H316	5.123555552	-3.549892457	2.892919659
	H317	0.127523170	2.443378177	-4.896902629
	H318	-2.265305175	-1.750188060	-6.089827719
	H319	7.829418913	-9.907595526	-0.670843149
	H320	7.203327284	-7.507844889	-2.758761563
	H321	6.535548293	-8.219326007	0.783125478
	H322	7.183368008	-10.469729147	-1.968195666
	H323	6.541205847	2.972604200	-8.393329272
	H324	6.292596354	-5.059622988	0.770961085
	H325	2.140899449	-7.786897194	0.957571492
	H326	4.629740873	-1.694989760	-6.968837051
	H327	3.234656842	-9.192871832	-0.615383618
	H328	4.307885898	-6.699415007	-6.603419481
	H329	3.147143539	-5.287607216	-4.852280799
	H330	5.801907125	-12.669943281	4.037886131
	H331	5.343126921	-1.259626689	2.843358449
	H332	8.067661412	-5.889431771	2.086908336
	H333	7.140319182	-3.799462991	1.089525518
	H334	8.301255699	-7.057370390	4.095130464
	H335	2.669699015	-2.949638963	5.521677991
	H336	4.405080777	-8.138991528	-3.240695938
	H337	5.381878145	-8.530029860	-1.820218783
	H338	2.314746600	-8.654057923	-2.025808771
	H339	2.457995827	-1.907108622	-5.883805427
	H340	-1.890649417	2.538227551	-6.381352973
	H341	-3.834656264	1.332519233	-7.426520245
	H342	2.110968821	-6.026104480	0.742895392
	H343	8.250535758	-7.413721530	1.819838755
	H344	7.179676698	-9.345069339	1.621040499
	H345	3.116858783	-1.277516987	5.610849549
	H346	6.674266824	-7.384936702	-5.879211558
	H347	7.031868052	-5.869534932	-2.149255483
	H348	1.837049306	-2.622098445	-7.403130563
	H349	5.572996780	4.358467969	-5.836860556
	H350	6.951243887	-0.073543186	5.222084171
	H351	7.294710891	0.462535189	3.562753666
	H352	10.006472118	-6.674841142	-4.986242044
	H353	9.110396753	-7.927350406	-4.428731068
	H354	10.498797705	-8.570636517	-0.169487549
	H355	9.017142375	-8.178644109	-1.857848154
	H356	12.248678561	-7.377375089	-1.896409088
	H357	10.183426533	-6.897515471	0.342830444
	H358	10.002134732	0.203585809	4.016581232
	H359	-3.592281203	13.005034892	3.767732183
	H360	9.043146038	-7.194686615	7.006797277
	H361	8.543022886	-4.731648858	5.892475443
	H362	-4.869256107	12.303859569	1.106441737
	H363	-4.661751577	10.164220978	3.319393253
	H364	-6.164494496	10.887910632	2.715866686
	N365	0.862520463	-3.338058592	-0.701102668
	H366	4.357008272	-1.536450417	0.847023581
	H367	-6.011069509	7.026849440	-2.467430077
	H368	0.104249453	-0.840985991	-0.629106841
	N369	-0.072147907	-3.814863687	-0.293577568
	H370	-1.920388248	-0.199427324	-1.240247624
	Mo371	4.198304503	-4.313071526	-1.823737058
	N372	-6.807107210	-7.093488392	-1.933966151
	N373	-8.972603741	-4.666506729	-0.082641311
	N374	-8.837699269	-5.088125099	2.691225245
	N375	-6.184620478	-5.930552378	3.162621498
	N376	-4.802139954	-3.495776206	2.681509219
	N377	-5.896853202	-1.970149722	4.869391450
	N378	-5.172816797	-3.465869223	7.235566223
	N379	-2.545407857	-4.754415905	7.024651401
	N380	-0.972300606	-2.781848306	5.928051460
	N381	-1.640765837	0.103956055	6.632295114
	N382	-2.467100711	-0.222982926	9.391953635
	N383	4.478305990	-11.825274516	5.540959780
	N384	5.550357463	-9.524235477	4.694156025
	N385	4.464293307	-3.519270214	3.676795368
	N386	3.255642817	-2.185366303	5.176898438
	N387	4.747363890	-1.219930578	3.681879814
	N388	8.188995323	-7.223242851	5.119891793
	N389	-5.223718041	-2.728079618	-4.128627317
	N390	-1.360278648	-5.447870401	-3.752285837
	N391	-5.573776564	-0.264649862	-3.299784279
	N392	-8.018911454	0.828450007	-2.469116491

	N393	-8.016158921	0.366240094	0.302842915
	N394	-6.005129019	2.073970531	1.262902530
	N395	-2.844408096	2.780276505	-1.531445463
	N396	-2.341910052	0.723149003	-1.136248713
	N397	-6.740161865	4.684968349	1.878849371
	N398	-9.872195603	7.788924168	0.158990959
	N399	-10.007938441	9.504408485	1.559490444
	N400	8.903253867	2.536111171	4.219158263
	N401	6.319764847	7.160840585	1.126993195
	N402	4.671571227	8.538167964	-1.658119991
	N403	2.143124653	7.974725358	-2.606501957
	N404	-2.260508099	6.638133233	-4.677832923
	N405	-2.243141185	8.500086554	-6.096648772
	N406	-3.614016074	8.474791400	-4.232592193
	N407	0.816398778	6.335599643	-0.646542790
	N408	0.757802394	8.129144790	1.500988186
	N409	-0.468330152	10.562937427	1.064873474
	N410	-1.265712750	10.536191623	-2.878276715
	N411	-2.997659540	11.557145572	1.712416694
	N412	5.921367813	-4.346879710	-9.497648535
	N413	3.875220244	-2.242453979	-7.390420668
	N414	2.742294158	0.530704415	-6.907128278
	N415	4.824162445	2.477101783	-6.489299901
	N416	0.186582502	-2.545439973	-10.350916161
	N417	-1.640516000	-0.779538282	-9.010902944
	N418	9.061213251	-6.938976001	-4.695059212
	N419	4.981032808	-5.512719495	-3.733135837
	N420	4.749458388	-6.407087620	-5.735757160
	N421	9.661324990	-7.407146988	-1.662796727
	N422	0.139023718	2.028057827	0.366093622
	N423	1.056819866	1.596719341	-0.136871333
	O424	-6.779392667	-5.193673950	0.219891420
	O425	-7.975731003	-2.991588545	2.959346989
	O426	-6.468598475	-5.450938338	5.379357758
	O427	-3.308588098	-4.186910027	4.252835251
	O428	-3.931969660	-0.827095155	4.855138365
	O429	-4.915954220	-1.427862516	8.246082188
	O430	-2.380198247	-4.461914826	9.293873615
	O431	0.959882351	-3.975508229	6.305411271
	O432	0.623525750	0.011103006	6.929239622
	O433	-0.998240053	1.456188199	9.812455607
	O434	-0.806383697	-0.801096547	12.515959090
	O435	6.592763218	-10.504813910	2.878536949
	O436	6.868525022	-8.069915279	6.800668047
	O437	10.528169197	-4.802306299	6.354735857
	O438	-5.552933116	-0.793661739	-1.066496696
	O439	-2.860213296	-4.131057226	-4.845228568
	O440	-6.888871886	2.609253864	-1.594189060
	O441	-5.569842513	3.282424112	-4.321470073
	O442	-10.009243033	1.485454649	0.508133367
	O443	-6.291563566	1.296141189	3.391928685
	O444	-4.864057900	4.938750008	3.155700420
	O445	-7.410214079	7.116903327	4.371031017
	O446	9.856080987	-0.373411975	5.967960748
	O447	2.737896830	4.422273280	4.734765343
	O448	3.688247743	8.348637540	0.392239825
	O449	1.641898196	10.206438656	-2.866400206
	O450	-1.118799458	7.238970325	-1.464788239
	O451	-1.118574966	7.073673492	2.294006478
	O452	2.077148336	5.562798269	2.119920388
	O453	0.410066495	11.440021399	2.992568832
	O454	-2.523619110	13.585364160	0.786119694
	O455	-2.927464851	11.098854900	-1.414719651
	O456	-5.487483633	13.603604719	3.293896077
	O457	-5.139306534	7.001832436	-2.027208089
	O458	3.355619610	-4.209088790	-8.456845472
	O459	1.323271506	-0.368195522	-8.467739278
	O460	3.720294320	3.921372428	-7.851155039
	O461	7.321846364	4.742238145	-7.744832596
	O462	-3.263519911	-2.187216210	-9.773811195
	O463	-0.336685726	-6.321698818	-6.460906345
	O464	1.682293528	-5.444242066	-6.815865580
	O465	-2.324233055	2.727104864	-8.978438550
	O466	10.431225040	-5.313976786	-2.060221417
	O467	12.784671619	-6.829829843	-0.003265864
	O468	0.555282949	-6.483689261	-1.620025532
	O469	-0.319983143	-7.522883814	0.171224116
	O470	5.679896354	-10.813168406	-2.772883665
	O471	3.474371364	-10.772957476	-3.268725226
	O472	5.254369224	-5.803654421	-0.574076955

	O473	4.917147640	-7.553647688	0.794531343
	O474	2.958726924	-6.046986125	-1.808066145
	O475	8.604882292	-6.666100877	2.378083271
	O476	6.778358851	-4.603999221	1.507225012
	O477	-3.832921048	4.933860089	-2.993514780
	O478	8.070000712	-10.369877653	-1.504401423
	O479	7.406506809	-8.681841482	0.922038981
	S480	3.895359008	5.003742926	-1.403345578
	S481	2.603030570	2.131265303	-2.741952037
	S482	2.686102862	-3.229826575	-3.317216308
	S483	3.826962308	1.730324336	0.619489055
	S484	0.906808108	-0.709666796	-1.730431705
	S485	4.915519935	-0.542794586	-4.701762037
	S486	3.499790347	-3.506467096	0.292570067
	S487	6.108475703	1.835696298	-2.123478742
	S488	6.149927294	-2.990906583	-2.083650850
	S489	6.520767145	-0.854500355	0.612935165
	end			

TS for dissociation of 6N2x (17 to (17d)

	2n2xs2bh6n2xdteS1ta_1_53241.47			
Fe(139) -2.365	C1	-7.852151539	-5.387200928	-0.379668365
Fe(140) -0.758	C2	-8.029195270	-6.476601933	-1.440938502
Fe(141) -2.579	C3	-8.894629325	-3.509573420	0.805940335
Fe(142) 2.702	C4	-8.510966611	-3.839628573	2.245532878
Fe(143) 2.620	C5	-8.426698869	-5.519675454	4.022196383
Fe(144) 1.178	C6	-6.914743277	-5.611573600	4.249439866
Fe(145) 1.232	C7	-4.697795574	-5.852661109	3.265365708
	C8	-4.186486515	-4.422649830	3.446727710
	C9	-4.484524114	-2.064021692	2.847143981
	C10	-4.746121100	-1.543702624	4.268588163
	C11	-6.205550756	-1.474852579	6.196926708
S=1	C12	-5.353555650	-2.103745017	7.309028253
	C13	-4.561254056	-4.189387386	8.331299586
	C14	-3.049068437	-4.452051037	8.253155686
	C15	-1.144093822	-5.068477097	6.819336572
	C16	-0.297744637	-3.894207524	6.317989053
	C17	-0.360613393	-1.617081917	5.371198103
	C18	-0.426228500	-0.452578579	6.370957548
	C19	-0.849104641	-1.268471627	3.941189828
	C20	-0.257084795	0.054763542	3.444273261
	C21	-0.524861857	-2.400507444	2.960277854
	C22	-1.787646180	1.235486844	7.507939447
	C23	-1.702973405	0.830286551	8.992087999
	C24	-3.060270482	2.045683289	7.164701183
	C25	-3.294191477	3.161895127	8.188423417
	C26	-2.955657426	2.626748321	5.748358434
	C27	-2.467804253	-0.607954810	10.788719399
	C28	-1.074937218	-0.866037912	11.327419717
	C29	5.095830197	-11.844377729	4.221814878
	C30	5.829277936	-10.550985749	3.872476159
	C31	6.010769446	-8.153663003	4.527193319
	C32	7.063043861	-7.813876356	5.600766271
	C33	4.811335413	-7.192592790	4.599885277
	C34	5.212538483	-5.740316387	4.350797254
	C35	4.005512373	-4.824969294	4.156340553
	C36	4.135306179	-2.317046466	4.193590210
	C37	9.279005095	-6.839825166	5.997388370
	C38	9.469247394	-5.341663045	6.084729133
	C39	-4.975536253	-2.540247813	-2.684272841
	C40	-5.405148994	-1.126133269	-2.249799861
	C41	-3.477791713	-2.708215885	-2.334959819
	C42	-2.929634105	-4.148069707	-2.409808944
	C43	-2.371777692	-4.569677470	-3.769073710
	C44	-5.679418951	1.169474504	-3.108990637
	C45	-6.925113070	1.600448697	-2.322967518
	C46	-5.583625728	1.846722317	-4.482715194
	C47	-9.241034858	1.074576766	-1.717361695
	C48	-9.120372783	0.989655374	-0.191953332
	C49	-7.741913160	0.363571995	1.734640335
	C50	-6.618763023	1.287863651	2.206574412
	C51	-4.962742765	3.007078267	1.673236901
	C52	-5.522180236	4.283505355	2.341916723
	C53	-4.131873270	3.503627306	0.479935795
	C54	-3.386899727	2.467872126	-0.291604188
	C55	-3.065508541	1.157271858	-0.047549014
	C56	-2.211988367	1.705384978	-2.034323798
	C57	-7.308763958	5.972770627	2.211970511
	C58	-7.459140335	6.071771101	3.729065698

	C59	-8.687169029	6.063409080	1.514434823
	C60	-9.270594827	7.436353809	1.350635245
	C61	-9.350499485	8.495201278	2.233653671
	C62	-10.303746914	9.025466218	0.304915965
	C63	8.484655680	1.426297008	5.090251496
	C64	9.535161526	0.331723131	5.038391869
	C65	7.152600815	0.803673701	4.607403723
	C66	5.993995138	1.768841063	4.673878317
	C67	5.208218713	1.894076102	5.834469586
	C68	5.663104130	2.568529963	3.567737007
	C69	4.120049583	2.773035200	5.885535593
	C70	4.591724013	3.461697782	3.608976131
	C71	3.811943137	3.553423518	4.764936853
	C72	5.551565805	6.840955404	-0.091063084
	C73	4.552236674	7.965675267	-0.420168970
	C74	4.818190964	5.519129697	0.104132594
	C75	3.850143063	9.647165986	-2.092912092
	C76	2.437366045	9.285463754	-2.560190567
	C77	0.841579990	7.395956488	-2.892007520
	C78	0.099052753	6.979094735	-1.596919369
	C79	1.056832813	6.210526978	-3.859565252
	C80	-0.145550561	5.346657148	-4.253264617
	C81	-1.119239301	5.936996359	-5.284561289
	C82	-2.708808463	7.836470963	-5.011587103
	C83	0.184516136	5.926226990	0.609852961
	C84	-0.131497454	7.107138167	1.538394984
	C85	1.030868704	4.911476286	1.405358378
	C86	0.816681170	9.111418459	2.573254530
	C87	0.248603530	10.478452663	2.226381923
	C88	-0.831866925	11.853105288	0.528663197
	C89	-2.204142374	12.395678028	0.999932595
	C90	-0.701620365	11.950274033	-0.993487791
	C91	-1.736407770	11.138479754	-1.762722351
	C92	-4.380909958	11.871842133	1.997699001
	C93	-4.488508614	12.921268057	3.095366829
	C94	-5.141961020	10.584368332	2.422112702
	C95	-5.192616759	9.564440068	1.307252624
	C96	-6.212384786	9.624200752	0.344232984
	C97	-4.197558780	8.585491808	1.156602122
	C98	-6.225980356	8.758485424	-0.751173267
	C99	-4.180168376	7.727214520	0.052710471
	C100	-5.196011908	7.828270970	-0.893110318
	C101	5.686520211	-3.608373837	-8.265571024
	C102	4.201296121	-3.369479246	-8.057231697
	C103	2.529289080	-1.882654982	-6.976808782
	C104	2.130808589	-0.503542454	-7.517181687
	C105	2.538614859	1.914213976	-7.300947139
	C106	3.749826011	2.841924453	-7.240353632
	C107	5.906799369	3.429875875	-6.290581912
	C108	6.648215839	3.738988013	-7.576206075
	C109	-1.012184546	-3.065814862	-9.677399229
	C110	-2.073769703	-1.975647300	-9.494128953
	C111	-0.783456323	-3.797575790	-8.332814193
	C112	-0.018227379	-5.119754763	-8.533108360
	C113	0.418903910	-5.691648193	-7.201244056
	C114	-2.580342307	0.318793952	-8.829661939
	C115	-1.979480702	1.618019733	-9.329014572
	C116	-3.250785935	0.409238689	-7.436107152
	C117	-2.211254786	0.397574520	-6.352620931
	C118	-1.550279621	1.580311589	-5.980590293
	C119	-1.752394984	-0.827621122	-5.840262454
	C120	-0.403543491	1.524680624	-5.183944959
	C121	-0.608664670	-0.880904145	-5.041896831
	C122	0.089757591	0.293494658	-4.744483144
	C123	8.664807121	-6.114787453	-3.527594182
	C124	9.650953583	-6.240479216	-2.357028008
	C125	7.241734149	-6.446227023	-3.052270292
	C126	6.140270815	-6.191240371	-4.029104959
	C127	5.985662425	-6.735562440	-5.287647874
	C128	4.148590335	-5.631978781	-4.736263524
	C129	10.528305807	-7.535031385	-0.508374624
	C130	11.967191299	-7.206818411	-0.840350971
	C131	0.659770877	-7.011645692	-0.401997678
	C132	2.054136485	-6.983553469	0.219395278
	C133	3.200964862	-7.036098418	-0.829094939
	C134	3.225325805	-8.449350826	-1.477490566
	C135	4.430332980	-8.758805002	-2.366101341
	C136	4.506619040	-10.231235871	-2.828872829
	C137	4.544874944	-6.793764474	-0.126691754
	C138	3.917085956	-0.653890888	-1.557983278

	Fe139	4.542961486	-1.838529318	-3.015440087
	Fe140	2.399093793	0.770814769	-1.016399622
	Fe141	5.269797535	0.515152192	-0.514728145
	Fe142	4.322269327	0.696988318	-2.934669695
	Fe143	4.145598154	2.771523332	-1.294106985
	Fe144	4.923629346	-1.964473517	-0.485887992
	Fe145	2.539486646	-2.203377144	-1.338966082
	H146	-6.253021442	-7.436614408	-1.151219229
	H147	-6.227940336	-6.391246055	-2.403691722
	H148	-9.799514955	-4.777266050	-0.654540829
	H149	-8.603636561	-6.063812366	-2.285598006
	H150	-8.670482285	-7.254468307	-0.991957140
	H151	-8.152369829	-2.781524974	0.446601896
	H152	-9.259206316	-5.733308904	2.050861502
	H153	-9.876050353	-3.016726630	0.815314309
	H154	-8.870163779	-6.508231773	4.201052037
	H155	-6.572176409	-5.948570822	2.241685221
	H156	-8.816162402	-4.827825219	4.780267420
	H157	-3.429986248	-1.863514008	2.619355476
	H158	-5.507720510	-3.755413453	2.018784755
	H159	-4.264081155	-6.287973597	2.354750200
	H160	-6.052463730	-0.389104259	6.252322232
	H161	-6.594999091	-2.460178684	4.316703109
	H162	-4.346225467	-6.435141698	4.125730464
	H163	-5.103081212	-1.504906090	2.131819583
	H164	-5.620910464	-3.975201873	6.484946992
	H165	-7.262693719	-1.693661469	6.401153454
	H166	-0.943470281	-3.363374884	3.283760201
	H167	-0.947168511	-2.175190461	1.968108056
	H168	0.563946860	-2.512321049	2.833655505
	H169	-1.941717698	-1.147737072	3.996117138
	H170	-5.065808985	-5.164792610	8.402115298
	H171	-2.843684239	1.843457376	4.986497776
	H172	-3.140078624	-0.662712586	8.764436514
	H173	-3.461395740	2.769342187	9.201780536
	H174	0.832920100	-0.016856398	3.303012112
	H175	-0.458931575	0.889098381	4.131591126
	H176	8.356458438	1.711370255	6.152801635
	H177	-3.858222076	3.202865525	5.499583457
	H178	-4.175755426	3.753478961	7.901445724
	H179	-2.090117692	3.306398501	5.677625986
	H180	-2.427383441	3.840892205	8.226756463
	H181	-3.922241738	1.355089036	7.199676647
	H182	-0.909132855	1.879359924	7.352816775
	H183	-2.467896838	-0.237241958	6.090722506
	H184	-0.696609603	0.319659901	2.470809644
	H185	1.445556151	4.171130560	0.704768590
	H186	0.351557777	4.396026361	2.099703219
	H187	2.165976391	5.143146381	3.000257667
	H188	2.251433990	4.377181140	5.577815447
	H189	4.332725981	4.059442593	2.735456151
	H190	6.234762188	2.470639977	2.642718534
	H191	5.438082855	1.285079358	6.712771125
	H192	-2.008010617	-2.898005449	5.828614228
	H193	-1.035257856	-5.889749492	6.095502411
	H194	-0.706612339	-5.398581795	7.770364325
	H195	-3.159083162	-4.778085180	6.201858046
	H196	-4.728915711	-3.643171741	9.267132827
	H197	3.506794853	2.837866997	6.788296285
	H198	0.706944752	-1.854578181	5.310752951
	H199	-2.962821449	0.142345563	11.426685145
	H200	-3.026575740	-1.552906165	10.874107499
	H201	-0.327503661	-1.194437798	10.567229919
	H202	10.226324506	-7.309656633	5.691184963
	H203	-0.968336979	-5.778410569	-4.640581871
	H204	1.584415982	-6.158455270	-1.873287807
	H205	-0.677454833	-5.853737734	-9.018616203
	H206	0.855066422	-4.957237887	-9.181010011
	H207	-1.748899534	-4.011362703	-7.848505595
	H208	-1.203364964	1.489646480	-10.118839041
	H209	-1.485200504	-3.780029469	-10.368180578
	H210	-2.890313861	-2.053178397	-3.000622029
	H211	-3.720877978	-4.868538115	-2.141774423
	H212	-0.656596026	-0.637201167	-8.764380368
	H213	-3.395126164	0.113402659	-9.555053190
	H214	0.764121365	-1.987358313	-9.705242874
	H215	-0.897215896	-5.766583568	-2.886849097
	H216	-0.233079615	-3.128764726	-7.652732085
	H217	0.761055453	-3.313214445	-10.682233636
	H218	-6.099449130	-3.239756822	-4.268318168

	H219	-3.346176631	-2.350255471	-1.302499594
	H220	2.205792211	-4.934321594	-7.518414971
	H221	-2.151640777	-4.277021318	-1.646235073
	H222	-4.323029522	2.524309295	2.425277132
	H223	-3.285058867	0.511779531	0.792792701
	H224	-8.657102023	0.685393444	2.251352086
	H225	-7.313842072	-0.032619206	-0.318853631
	H226	-7.232472644	4.072615101	1.228221201
	H227	-8.989473866	8.630517434	3.247089580
	H228	-7.669729099	5.092911937	4.223767716
	H229	-9.396452936	5.393987967	2.032655185
	H230	-6.654168705	6.789964035	1.869612017
	H231	-10.823810993	9.623347838	-0.439854408
	H232	-3.413681027	4.239626722	0.877241420
	H233	-4.792681745	4.043871869	-0.216682452
	H234	-10.223668486	10.421040506	1.910906064
	H235	-8.566042455	5.657238745	0.498002347
	H236	-6.320456163	2.060778187	0.292698020
	H237	-7.519039009	-0.652694849	2.091734097
	H238	-9.619797393	2.082834528	-1.934601309
	H239	-1.688111987	1.639286679	-2.985557098
	H240	-10.001809693	0.354839808	-2.045824078
	H241	9.655151160	3.053469040	4.667603064
	H242	8.126292027	3.186592778	4.108361315
	H243	1.777085480	5.979028325	-0.844302625
	H244	-0.781618279	5.456185462	0.374529468
	H245	1.640809962	7.982464180	0.987308564
	H246	-3.047915731	3.666888793	-2.055344632
	H247	-4.479476353	4.355345525	-3.477737360
	H248	-5.760706922	3.668541406	-5.184640735
	H249	-4.357131325	5.569623764	-2.455721198
	H250	-4.654708973	1.505855364	-4.969625134
	H251	-6.442219056	1.552164336	-5.105886697
	H252	0.323555345	11.690809137	-1.293132466
	H253	-0.864766891	13.006017931	-1.263037303
	H254	-0.255628210	10.376455673	-3.000703172
	H255	-1.914103186	9.876047884	-3.323916824
	H256	-2.505502416	9.459889206	-6.252802594
	H257	-1.513100602	5.133334195	-5.925493592
	H258	-7.035356365	8.806502214	-1.483852163
	H259	-3.409645086	8.469987122	1.905446342
	H260	-3.977080630	7.989809864	-3.395560847
	H261	1.802957491	5.543292363	-3.398828317
	H262	2.903512005	7.313323934	-2.362236457
	H263	-0.699873611	5.009575836	-3.363257234
	H264	-7.945893602	-0.017535539	-3.036719204
	H265	4.355040771	10.168348167	-2.916587517
	H266	-5.415390815	-0.728387414	-4.202873232
	H267	1.527653786	6.613930127	-4.771146272
	H268	-3.358678059	7.021718388	-0.081230320
	H269	3.724357644	10.364777333	-1.268564638
	H270	-1.716324734	8.020842126	-6.812050474
	H271	-4.221838726	9.162330803	-4.644157200
	H272	-4.449486751	-3.284600677	-4.530014184
	H273	-5.546706997	-3.236913420	-2.043536715
	H274	-7.014620725	10.358376844	0.449187205
	H275	-4.842244112	1.521042131	-2.489677331
	H276	0.223811719	8.174511041	-3.352090513
	H277	-0.576907117	6.633063247	-5.936830188
	H278	0.275868176	4.440038851	-4.707904918
	H279	-2.807736660	6.080408990	-3.987143489
	H280	0.254384479	8.731936634	3.439259157
	H281	-0.453873069	9.732689684	0.455271981
	H282	-0.118458848	12.572962649	0.959683713
	H283	1.859832896	9.265808386	2.882067162
	H284	-2.704545370	10.571130898	1.729396928
	H285	6.218595063	-2.645940253	-8.298020236
	H286	6.031647708	-4.133900410	-7.350666844
	H287	8.705042973	-5.056932947	-3.826739051
	H288	6.884334581	-4.683838508	-9.513436734
	H289	5.322839009	-5.175923462	-9.511940372
	H290	5.563336071	4.750633563	0.344162255
	H291	5.643422085	7.363006537	1.881725638
	H292	5.407242091	8.198941953	-2.277837409
	H293	4.099250188	5.606947325	0.930127353
	H294	6.856125550	8.005213205	0.992044868
	H295	6.629665626	2.973157937	-5.597050114
	H296	6.271123170	6.734225911	-0.918134729
	H297	4.322729200	-7.302094621	5.582558175
	H298	6.463470536	-8.088371475	3.529519137

H299	4.838495744	1.618276234	-5.940068139
H300	4.371225718	-0.294976237	3.946988089
H301	2.171468967	1.917314060	-8.333952358
H302	-0.249504351	-1.840932637	-4.664610366
H303	1.757947537	2.389856956	-6.682454132
H304	3.447146157	-4.718119635	5.093982777
H305	3.290860367	0.308047977	-6.052803801
H306	3.316828628	-5.250224277	3.408398707
H307	4.093074238	-7.518067047	3.830429921
H308	5.835034898	-5.685777273	3.445842176
H309	5.820308062	-5.354021779	5.184406688
H310	-3.934075886	-0.446700006	-7.336590993
H311	4.926727919	-9.762617618	5.475087317
H312	4.303676744	-11.952534788	3.465191009
H313	1.011239329	0.263224136	-4.166245869
H314	5.144262499	-12.177475432	6.252604937
H315	3.674482885	-12.446943126	5.581926999
H316	5.098923062	-3.532479873	2.892251230
H317	0.129876165	2.436644364	-4.912875839
H318	-2.280105398	-1.750866942	-6.089438296
H319	7.826180943	-9.921329251	-0.673718169
H320	7.202023677	-7.513511426	-2.774019177
H321	6.543769003	-8.232199986	0.772248295
H322	7.171481967	-10.476984880	-1.968985936
H323	6.564788108	2.955923642	-8.367623957
H324	6.291063502	-5.063969924	0.772668409
H325	2.140224471	-7.804119114	0.942818682
H326	4.637067898	-1.694087548	-6.966813767
H327	3.199969180	-9.170951595	-0.646106334
H328	4.275859421	-6.650876410	-6.576930244
H329	3.140073084	-5.247298444	-4.807559785
H330	5.807284583	-12.670494922	4.054827270
H331	5.323612193	-1.252996050	2.863260312
H332	8.079678105	-5.897359535	2.079722083
H333	7.154104880	-3.807806523	1.061450154
H334	8.305309564	-7.063336675	4.098857717
H335	2.662925247	-2.951343504	5.540695229
H336	4.388027664	-8.131865862	-3.269434931
H337	5.363858562	-8.510766088	-1.843466192
H338	2.295207005	-8.588866806	-2.048920412
H339	2.462788140	-1.905039553	-5.877150987
H340	-1.908143884	2.537399833	-6.367389094
H341	-3.848710298	1.332972694	-7.424259658
H342	2.123479769	-6.038198366	0.784726901
H343	8.256040287	-7.424070147	1.822539168
H344	7.181830088	-9.354833340	1.619542802
H345	3.091020228	-1.276558858	5.622393293
H346	6.646633612	-7.355877576	-5.879352528
H347	7.033650798	-5.887701830	-2.131373617
H348	1.842409411	-2.621612596	-7.394018135
H349	5.559535625	4.373146457	-5.840893248
H350	6.947464607	-0.074778311	5.236365251
H351	7.287648508	0.451720822	3.572102702
H352	9.997200364	-6.647690556	-4.993357573
H353	9.113709266	-7.910395063	-4.441472858
H354	10.504014941	-8.587304183	-0.183437578
H355	9.014708807	-8.181167527	-1.863252685
H356	12.253888077	-7.388325871	-1.904321680
H357	10.190090685	-6.915723107	0.337684977
H358	10.004635054	0.193016495	4.034420991
H359	-3.592465069	13.012762857	3.753892446
H360	9.036530364	-7.194704323	7.014227818
H361	8.551016596	-4.733143892	5.893134632
H362	-4.857999556	12.289422857	1.095276914
H363	-4.660431606	10.158086556	3.316053763
H364	-6.159806088	10.885279355	2.709424581
N365	0.552919346	-3.677107931	-0.629346583
H366	4.361589377	-1.344861701	0.744166997
H367	-6.012960589	7.010529418	-2.463134421
H368	0.076309994	-0.921173496	-0.600960390
N369	-0.345652428	-3.973308447	-0.044196052
H370	-1.925612536	-0.205468675	-1.251579723
Mo371	4.230896418	-4.262335989	-1.807388630
N372	-6.810072581	-7.091231680	-1.937125332
N373	-8.967863408	-4.660866083	-0.080887974
N374	-8.806063248	-5.083155585	2.688786921
N375	-6.148987330	-5.916109780	3.171086949
N376	-4.782891068	-3.478256717	2.676967684
N377	-5.890105815	-1.956222425	4.861328259
N378	-5.163220281	-3.446330970	7.232842452

	N379	-2.553959269	-4.784570855	7.027047080
	N380	-0.995680908	-2.812894538	5.896807809
	N381	-1.651265265	0.081049510	6.619702738
	N382	-2.455704820	-0.227314626	9.387994282
	N383	4.483594320	-11.824540929	5.556846806
	N384	5.548530284	-9.521479415	4.696246358
	N385	4.452769894	-3.511514623	3.686825351
	N386	3.261264022	-2.189854888	5.211898299
	N387	4.726758845	-1.212920686	3.697713459
	N388	8.189095046	-7.226584637	5.123607988
	N389	-5.224569964	-2.734762277	-4.116388265
	N390	-1.344685968	-5.439686637	-3.747990451
	N391	-5.574047792	-0.268804203	-3.290345547
	N392	-8.019727385	0.819785769	-2.463015298
	N393	-8.019336108	0.364766454	0.310548235
	N394	-6.000439830	2.067298310	1.265868305
	N395	-2.843418931	2.775965118	-1.532108449
	N396	-2.348131442	0.716009367	-1.142109267
	N397	-6.732454291	4.678692085	1.873868673
	N398	-9.869009268	7.780132815	0.149327174
	N399	-10.007325832	9.496019855	1.549009872
	N400	8.901880465	2.527036517	4.217727572
	N401	6.310764810	7.149894700	1.132910268
	N402	4.678715965	8.538020766	-1.655121119
	N403	2.152988380	7.967361694	-2.607804943
	N404	-2.257333582	6.629590893	-4.671771695
	N405	-2.234623798	8.496774496	-6.082816356
	N406	-3.612037381	8.465059870	-4.224325898
	N407	0.825305392	6.333439122	-0.641923195
	N408	0.770119584	8.128909368	1.505372261
	N409	-0.459995929	10.554515277	1.058820381
	N410	-1.265535381	10.526180960	-2.883121476
	N411	-2.989009563	11.547345177	1.711665343
	N412	5.919992714	-4.343819573	-9.510636128
	N413	3.881523175	-2.244214797	-7.383323512
	N414	2.740066609	0.532966366	-6.899519111
	N415	4.818566162	2.486470273	-6.483728019
	N416	0.183565937	-2.538937272	-10.348199740
	N417	-1.643793323	-0.775636804	-9.002860059
	N418	9.055236586	-6.920105381	-4.698820854
	N419	4.981386441	-5.487831077	-3.704854942
	N420	4.727650129	-6.368639066	-5.711182277
	N421	9.663832591	-7.414014381	-1.668872795
	N422	0.101365416	2.006382442	0.337957927
	N423	1.010504569	1.571269726	-0.179457275
	O424	-6.775366079	-5.192262842	0.213935867
	O425	-7.954184097	-2.982634464	2.955238006
	O426	-6.444133971	-5.431959543	5.384775585
	O427	-3.293004413	-4.146153244	4.262688759
	O428	-3.941670061	-0.785481247	4.832182790
	O429	-4.916825733	-1.405122191	8.240277608
	O430	-2.360377495	-4.411160515	9.280185452
	O431	0.944465337	-3.982129427	6.304743948
	O432	0.612185170	-0.035554231	6.909208624
	O433	-0.977578114	1.447867444	9.790486393
	O434	-0.793531646	-0.793709252	12.514351489
	O435	6.585973482	-10.508822454	2.881190163
	O436	6.853856291	-8.051651962	6.803127931
	O437	10.535147396	-4.812370185	6.359111183
	O438	-5.552841314	-0.796959713	-1.056674583
	O439	-2.862646343	-4.143402474	-4.839105120
	O440	-6.893983536	2.602868225	-1.587105904
	O441	-5.570193794	3.277881385	-4.312989278
	O442	-10.014714785	1.480967426	0.509945004
	O443	-6.304493191	1.312397065	3.400154887
	O444	-4.862011014	4.932170251	3.158511299
	O445	-7.409675342	7.112384528	4.361379798
	O446	9.846756472	-0.370543933	5.989064415
	O447	2.741358300	4.427382839	4.736467418
	O448	3.691197952	8.344139068	0.392357119
	O449	1.644699751	10.197849610	-2.865702698
	O450	-1.107031517	7.242368702	-1.460399376
	O451	-1.107754864	7.077165967	2.298622524
	O452	2.087984446	5.558858866	2.117728767
	O453	0.416286208	11.444597338	2.981103518
	O454	-2.521393545	13.571788866	0.775606969
	O455	-2.925973712	11.093411085	-1.419742884
	O456	-5.491236703	13.594123379	3.276304255
	O457	-5.139678397	6.990472949	-2.025792543
	O458	3.354517911	-4.201881790	-8.462560994

	O459 1.327597835 -0.370297052 -8.463465895<br>O460 3.707842244 3.927252958 -7.844409255<br>O461 7.328294442 4.740175123 -7.735795489<br>O462 -3.268879090 -2.184027355 -9.759488178<br>O463 -0.332190329 -6.317237976 -6.459231411<br>O464 1.685822989 -5.439955830 -6.819239979<br>O465 -2.331763643 2.730294561 -8.963245869<br>O466 10.437518075 -5.320247700 -2.059867850<br>O467 12.790203444 -6.846508098 -0.010342240<br>O468 0.575158447 -6.489030579 -1.631349084<br>O469 -0.328565399 -7.431365494 0.198492888<br>O470 5.652667734 -10.801114062 -2.774644571<br>O471 3.444899681 -10.763501680 -3.260185915<br>O472 5.236316543 -5.762578284 -0.530422020<br>O473 4.930644536 -7.572148762 0.768510924<br>O474 2.960153032 -5.999509487 -1.784840754<br>O475 8.610293026 -6.675726621 2.378944746<br>O476 6.803258524 -4.609569169 1.494150383<br>O477 -3.827017122 4.923827855 -2.986416518<br>O478 8.058157216 -10.389662852 -1.506271190<br>O479 7.412377206 -8.693972270 0.918330696<br>S480 3.887102155 5.017469441 -1.413136761<br>S481 2.583884053 2.130986395 -2.780537819<br>S482 2.753156902 -3.163855203 -3.321454532<br>S483 3.743706554 1.709973793 0.615061292<br>S484 0.822670019 -0.783107231 -1.735107668<br>S485 4.982660688 -0.467135072 -4.705960720<br>S486 3.454653766 -3.391028464 0.288728117<br>S487 6.087441799 1.873898624 -2.067886322<br>S488 6.211802583 -2.996075814 -2.073697140<br>S489 6.609452733 -0.830876022 0.573073878<br>end

TS for dissociation of 2N2x

Fe(139) -2.369<br>Fe(140) -0.960<br>Fe(141) -2.530<br>Fe(142) 2.784<br>Fe(143) 2.747<br>Fe(144) 1.481<br>Fe(145) 0.896<br><br>S=1	s2bh6n2x2n2xdtb_1_53238.29<br>C1 -7.837608000 -5.382444178 -0.383880999<br>C2 -8.021449363 -6.474380544 -1.441986515<br>C3 -8.887552329 -3.509702485 0.805701637<br>C4 -8.503918069 -3.840432293 2.245110391<br>C5 -8.418862591 -5.521196535 4.021068112<br>C6 -6.907305677 -5.611528208 4.250612204<br>C7 -4.688744580 -5.850888188 3.270254987<br>C8 -4.180674428 -4.419543652 3.449537034<br>C9 -4.482540091 -2.062839643 2.844510602<br>C10 -4.745442160 -1.540504369 4.265030951<br>C11 -6.203785349 -1.474938064 6.194560006<br>C12 -5.351964463 -2.103252122 7.307215184<br>C13 -4.559373540 -4.188453986 8.330710793<br>C14 -3.047429392 -4.453021708 8.251982955<br>C15 -1.145110733 -5.076591931 6.817466025<br>C16 -0.301051329 -3.901582293 6.313450924<br>C17 -0.366331778 -1.626872961 5.361191087<br>C18 -0.429608228 -0.464122821 6.364247554<br>C19 -0.855978844 -1.277702469 3.932087854<br>C20 -0.267731248 0.048290898 3.437429428<br>C21 -0.527840015 -2.407504425 2.949072351<br>C22 -1.785612729 1.226939661 7.503657183<br>C23 -1.699366981 0.823619581 8.988514802<br>C24 -3.057469120 2.039101279 7.161894255<br>C25 -3.290024243 3.155086235 8.186168312<br>C26 -2.953194664 2.621005992 5.745870852<br>C27 -2.464810369 -0.610175583 10.788640339<br>C28 -1.072123254 -0.868840482 11.327133414<br>C29 5.091345846 -11.835803669 4.213417054<br>C30 5.827911290 -10.545800145 3.867769577<br>C31 6.010579209 -8.152028244 4.526439898<br>C32 7.062172886 -7.812303653 5.599872954<br>C33 4.809864938 -7.193292075 4.598495543<br>C34 5.210063342 -5.740930919 4.350441742<br>C35 4.002882993 -4.826313356 4.155383860<br>C36 4.135046283 -2.317923988 4.199276969<br>C37 9.278436780 -6.840522598 5.995081637<br>C38 9.470434149 -5.343047879 6.083628067<br>C39 -4.961233964 -2.541808451 -2.679097478<br>C40 -5.396646915 -1.128775100 -2.246318181<br>C41 -3.463990075 -2.707276911 -2.330158925<br>C42 -2.909432340 -4.145060417 -2.399017785<br>C43 -2.365233508 -4.572571648 -3.762070538<br>C44 -5.679551703 1.164389607 -3.108199495

	C45	-6.925121893	1.594664758	-2.321825608	
	C46	-5.584358901	1.841488636	-4.481982578	
	C47	-9.241098430	1.068677794	-1.716635206	
	C48	-9.120703359	0.984964787	-0.191201807	
	C49	-7.742658325	0.360530835	1.736277932	
	C50	-6.620013703	1.285323071	2.207785487	
	C51	-4.963542114	3.002888263	1.673013248	
	C52	-5.522243160	4.280546322	2.341478794	
	C53	-4.134014221	3.498285677	0.478719308	
	C54	-3.395728950	2.461057972	-0.296766820	
	C55	-3.075182237	1.149583909	-0.053167341	
	C56	-2.220073311	1.699127161	-2.040838551	
	C57	-7.310964375	5.969574177	2.212045698	
	C58	-7.461730312	6.067690390	3.729037957	
	C59	-8.687829982	6.058900869	1.511973651	
	C60	-9.271017131	7.431658793	1.347771257	
	C61	-9.349823784	8.490808191	2.230438310	
	C62	-10.304399471	9.020596596	0.302255844	
	C63	8.490208360	1.425907918	5.102802790	
	C64	9.537335536	0.328423621	5.048561606	
	C65	7.158030073	0.802761217	4.616277972	
	C66	5.997307584	1.766572801	4.678059723	
	C67	5.210109614	1.892919571	5.837713347	
	C68	5.664845873	2.562184442	3.569276549	
	C69	4.120834357	2.770611656	5.886497184	
	C70	4.592423564	3.454375404	3.608379251	
	C71	3.813001834	3.548846948	4.764380922	
	C72	5.555902257	6.858421700	-0.085401553	
	C73	4.552632793	7.976288200	-0.417648898	
	C74	4.826127978	5.531332207	0.103393994	
	C75	3.849187964	9.649287611	-2.096427282	
	C76	2.436176693	9.289480764	-2.562475776	
	C77	0.836390101	7.405405751	-2.893869918	
	C78	0.098100663	6.983666448	-1.597794006	
	C79	1.052404548	6.215690878	-3.858179391	
	C80	-0.148549953	5.348756835	-4.251069121	
	C81	-1.123058286	5.936585862	-5.283648579	
	C82	-2.714604077	7.837590899	-5.013874350	
	C83	0.192785768	5.933002341	0.609059203	
	C84	-0.130003631	7.111826709	1.536432007	
	C85	1.052094753	4.933611629	1.411232243	
	C86	0.816501107	9.115465643	2.570997729	
	C87	0.247565535	10.483240103	2.225855077	
	C88	-0.837488050	11.863680630	0.530669154	
	C89	-2.210544171	12.403209625	1.000022073	
	C90	-0.704892949	11.953935606	-0.992373143	
	C91	-1.737347464	11.137380048	-1.760487033	
	C92	-4.387949402	11.877631365	1.997792086	
	C93	-4.494749652	12.923947082	3.097355469	
	C94	-5.143686050	10.585555190	2.420879438	
	C95	-5.191917450	9.564514541	1.306132440	
	C96	-6.212361054	9.622293742	0.343547514	
	C97	-4.196926085	8.585104115	1.156237545	
	C98	-6.226442729	8.755288972	-0.750722132	
	C99	-4.180214573	7.725275366	0.053327887	
	C100	-5.196418411	7.825275044	-0.892151049	
	C101	5.685991642	-3.609668190	-8.256842997	
	C102	4.200471656	-3.372005168	-8.053255145	
	C103	2.528704033	-1.886292408	-6.974289089	
	C104	2.132883780	-0.506106931	-7.512620786	
	C105	2.540733252	1.910043530	-7.295808546	
	C106	3.750668192	2.839285627	-7.239035279	
	C107	5.909817553	3.435550597	-6.299233241	
	C108	6.654170851	3.738125416	-7.582634925	
	C109	-1.005557718	-3.065401267	-9.666362318	
	C110	-2.068104404	-1.976522255	-9.485324967	
	C111	-0.778861146	-3.800703936	-8.324638136	
	C112	-0.016625176	-5.123235074	-8.530507757	
	C113	0.421748027	-5.697999755	-7.200662496	
	C114	-2.576297694	0.317024830	-8.822367291	
	C115	-1.978990861	1.616613975	-9.323878074	
	C116	-3.252655094	0.406270441	-7.432002322	
	C117	-2.215438579	0.393877673	-6.347514493	
	C118	-1.553891250	1.576808853	-5.977327022	
	C119	-1.755712674	-0.832048275	-5.837551184	
	C120	-0.403847392	1.520264575	-5.185969306	
	C121	-0.608911743	-0.885803668	-5.043644230	
	C122	0.091739660	0.288471714	-4.751072694	
	C123	8.664832348	-6.116813532	-3.530401925	
	C124	9.650468251	-6.242288128	-2.359839343	

	C125	7.243340268	-6.452551834	-3.055060092
	C126	6.141008193	-6.198188348	-4.030657776
	C127	5.985979553	-6.736855345	-5.291583209
	C128	4.148394247	-5.636685764	-4.735696685
	C129	10.523193683	-7.534380016	-0.509246845
	C130	11.963536386	-7.210186442	-0.840593059
	C131	0.652055715	-6.999788615	-0.387981584
	C132	2.051305607	-6.989261639	0.222174271
	C133	3.196045870	-7.055778586	-0.827664154
	C134	3.214904651	-8.465997855	-1.477975076
	C135	4.421447180	-8.768162515	-2.367562255
	C136	4.501989986	-10.238854181	-2.832103937
	C137	4.547348812	-6.824070542	-0.134421010
	C138	3.927718958	-0.637434213	-1.554164239
	Fe139	4.449217834	-1.863134373	-2.994577852
	Fe140	2.537984276	0.689311147	-1.044999490
	Fe141	5.254425682	0.513449959	-0.540344509
	Fe142	4.379051129	0.696015671	-2.985962104
	Fe143	4.116492057	2.761921779	-1.282085499
	Fe144	4.899723675	-1.979067231	-0.467954736
	Fe145	2.364484587	-2.389721959	-1.239857397
	H146	-6.249495969	-7.441770039	-1.154392542
	H147	-6.222296838	-6.397815053	-2.408236398
	H148	-9.789027154	-4.780008088	-0.653523721
	H149	-8.597494387	-6.062209872	-2.286038065
	H150	-8.664495455	-7.248862277	-0.989297528
	H151	-8.147899341	-2.778344964	0.447894312
	H152	-9.248627651	-5.735491035	2.048584697
	H153	-9.870670658	-3.020141464	0.815364908
	H154	-8.861444832	-6.510252384	4.199347460
	H155	-6.561837844	-5.949597001	2.243884660
	H156	-8.810485475	-4.829793877	4.778496796
	H157	-3.428459922	-1.859940490	2.616595850
	H158	-5.499281403	-3.757439135	2.016571857
	H159	-4.251419160	-6.287705503	2.362180496
	H160	-6.051311248	-0.389122877	6.250028403
	H161	-6.591450711	-2.462120184	4.314998803
	H162	-4.338459928	-6.430522956	4.133006541
	H163	-5.102590311	-1.506912470	2.127949177
	H164	-5.617288709	-3.974933403	6.483299527
	H165	-7.260887701	-1.694347443	6.398344616
	H166	-0.944097445	-3.371086893	3.272857235
	H167	-0.947656492	-2.187079060	1.954501216
	H168	0.561380218	-2.516171595	2.825439961
	H169	-1.948565538	-1.158068053	3.988133180
	H170	-5.065598252	-5.162905134	8.402780058
	H171	-2.841655975	1.838010947	4.983641435
	H172	-3.137803548	-0.668382749	8.764773241
	H173	-3.457130130	2.762272697	9.199410358
	H174	0.821080541	-0.022286571	3.286471677
	H175	-0.465611812	0.880646799	4.128643568
	H176	8.360842591	1.711861842	6.164821175
	H177	-3.855871909	3.197252846	5.497738168
	H178	-4.171349088	3.747306080	7.899722658
	H179	-2.087711471	3.300806248	5.675393547
	H180	-2.422802646	3.833552627	8.224400796
	H181	-3.920068218	1.349345940	7.196866470
	H182	-0.906051181	1.869095670	7.347093461
	H183	-2.470806520	-0.243617358	6.086493089
	H184	-0.719188788	0.312351794	2.468925393
	H185	1.490480452	4.207926306	0.712159619
	H186	0.373101029	4.407443627	2.096665191
	H187	2.177246006	5.148658784	3.007638758
	H188	2.253495775	4.376008245	5.576539054
	H189	4.334267629	4.050266343	2.733298722
	H190	6.234149022	2.461118095	2.643136189
	H191	5.439340871	1.284724807	6.716750244
	H192	-2.012053712	-2.913022222	5.810530946
	H193	-1.036454573	-5.898821499	6.094637591
	H194	-0.705431323	-5.404975412	7.768091474
	H195	-3.160975961	-4.788268099	6.202834857
	H196	-4.725977090	-3.640674662	9.265785086
	H197	3.507107486	2.836328770	6.788857925
	H198	0.701247302	-1.864488897	5.299834387
	H199	-2.959485260	0.141238824	11.425928140
	H200	-3.024770488	-1.554340218	10.876107614
	H201	-0.325684485	-1.198249650	10.566619757
	H202	10.225582315	-7.311820305	5.690005837
	H203	-0.966317919	-5.785033298	-4.635571690
	H204	1.557971932	-6.160925242	-1.869112135

	H205	-0.677882044	-5.854760650	-9.016943754
	H206	0.855653061	-4.960233399	-9.179692696
	H207	-1.745582645	-4.014334444	-7.843001751
	H208	-1.206317943	1.488631125	-10.117212441
	H209	-1.478931537	-3.778355699	-10.357879294
	H210	-2.878719037	-2.053108860	-2.998629661
	H211	-3.696523684	-4.866697904	-2.119718533
	H212	-0.651343917	-0.636945518	-8.758262941
	H213	-3.388824049	0.110622523	-9.549855310
	H214	0.771549189	-1.987700262	-9.699109974
	H215	-0.884521549	-5.757822378	-2.880944986
	H216	-0.228449069	-3.135323076	-7.641546651
	H217	0.763728306	-3.312981697	-10.678065500
	H218	-6.091277024	-3.240844886	-4.259528008
	H219	-3.333849577	-2.347359558	-1.298200985
	H220	2.207542594	-4.938358909	-7.518771475
	H221	-2.119268929	-4.262463189	-1.644382474
	H222	-4.323851397	2.521141528	2.425821624
	H223	-3.291251436	0.504126685	0.788311287
	H224	-8.658032844	0.682961771	2.252228673
	H225	-7.313446796	-0.036459833	-0.316757443
	H226	-7.232464660	4.069196181	1.228411749
	H227	-8.987929989	8.626411172	3.243497222
	H228	-7.671901430	5.088589743	4.223278313
	H229	-9.397705496	5.388921524	2.028599270
	H230	-6.656475329	6.787143075	1.870286358
	H231	-10.825043409	9.618286358	-0.442277131
	H232	-3.412250524	4.231743746	0.874399732
	H233	-4.793752003	4.041606176	-0.216134302
	H234	-10.223481290	10.416400986	1.908100544
	H235	-8.564347057	5.652984558	0.495684219
	H236	-6.320745466	2.056217654	0.292731476
	H237	-7.520185623	-0.655500128	2.094331782
	H238	-9.620094573	2.076626443	-1.934865459
	H239	-1.695254811	1.634117068	-2.992506391
	H240	-10.001468050	0.348406319	-2.044813263
	H241	9.659378145	3.053047062	4.672660722
	H242	8.130128116	3.181255157	4.111772465
	H243	1.780523131	5.980453160	-0.849991109
	H244	-0.772944757	5.458266151	0.379222030
	H245	1.640684592	7.986081012	0.985206353
	H246	-3.053425926	3.662763629	-2.059512566
	H247	-4.481531021	4.352101470	-3.479381085
	H248	-5.762963007	3.663329993	-5.183651988
	H249	-4.359165916	5.566898893	-2.456732947
	H250	-4.655306693	1.501268190	-4.968964362
	H251	-6.442780999	1.546477813	-5.105010383
	H252	0.320926672	11.692678339	-1.289015535
	H253	-0.867182859	13.008181360	-1.267651857
	H254	-0.254251999	10.376303741	-2.997476895
	H255	-1.912304693	9.872316634	-3.319935829
	H256	-2.503142729	9.462299007	-6.252323931
	H257	-1.515314077	5.130712973	-5.922935279
	H258	-7.036241650	8.801958190	-1.482884342
	H259	-3.408674156	8.469891095	1.904813445
	H260	-3.981339029	7.987840863	-3.397283559
	H261	1.798659763	5.550327857	-3.394257765
	H262	2.898887188	7.315373218	-2.365604982
	H263	-0.702621102	5.010959692	-3.361024495
	H264	-7.945661512	-0.023034947	-3.036316146
	H265	4.355130543	10.168448635	-2.920544698
	H266	-5.411892207	-0.734892783	-4.200006902
	H267	1.524530271	6.615141499	-4.770890195
	H268	-3.358967167	7.019427090	-0.080147074
	H269	3.724262304	10.367377389	-1.272506501
	H270	-1.713612951	8.021857424	-6.809258512
	H271	-4.226894209	9.163098936	-4.643451407
	H272	-4.441799830	-3.290557666	-4.524567029
	H273	-5.531723027	-3.239098417	-2.038057190
	H274	-7.015143948	10.355778610	0.448576322
	H275	-4.843285919	1.517976066	-2.489010425
	H276	0.217806890	8.182994832	-3.354012912
	H277	-0.579985950	6.630807229	-5.937208661
	H278	0.274908643	4.442193310	-4.704781993
	H279	-2.812874903	6.080603858	-3.989371143
	H280	0.254980014	8.734512099	3.437115721
	H281	-0.458137654	9.742417641	0.453954139
	H282	-0.122980537	12.582754573	0.960666716
	H283	1.860005700	9.268827905	2.879368615
	H284	-2.708881806	10.578384270	1.730274450

	H285	6.217956048	-2.647140812	-8.287172573
	H286	6.030464442	-4.137351069	-7.342993794
	H287	8.702578997	-5.058350981	-3.827754621
	H288	6.885315858	-4.682408354	-9.508325022
	H289	5.323143323	-5.173615264	-9.508913344
	H290	5.574086138	4.763875411	0.338746874
	H291	5.641830283	7.366412526	1.890085160
	H292	5.407039454	8.200882929	-2.275878753
	H293	4.110700558	5.614204915	0.933099027
	H294	6.859180456	8.013782683	1.008557379
	H295	6.630486914	2.984267294	-5.599459380
	H296	6.276511879	6.753386359	-0.911799167
	H297	4.320655884	-7.304193097	5.580704798
	H298	6.463698040	-8.086949788	3.528922902
	H299	4.844473170	1.619805885	-5.941189368
	H300	4.371710760	-0.295807543	3.958053467
	H301	2.171508000	1.911643974	-8.328222483
	H302	-0.247533799	-1.845946280	-4.668285668
	H303	1.760652840	2.385350956	-6.676381301
	H304	3.442015749	-4.722545870	5.091825449
	H305	3.290438969	0.302556477	-6.044879203
	H306	3.316440682	-5.250398097	3.404616300
	H307	4.092868872	-7.519665004	3.828231657
	H308	5.833258226	-5.686000760	3.445872991
	H309	5.817184027	-5.354963534	5.184689006
	H310	-3.935746715	-0.450132940	-7.335931050
	H311	4.926960019	-9.761694281	5.473256306
	H312	4.297014983	-11.943252132	3.459415567
	H313	1.016814504	0.259343043	-4.179254432
	H314	5.146508316	-12.173879824	6.244363793
	H315	3.675504722	-12.444689745	5.578240262
	H316	5.100907203	-3.531263939	2.898710739
	H317	0.133294667	2.430329072	-4.915674935
	H318	-2.284190996	-1.754919594	-6.086498229
	H319	7.821227553	-9.916866863	-0.675064849
	H320	7.205458728	-7.520137401	-2.777866926
	H321	6.537249319	-8.228990003	0.771654870
	H322	7.166169719	-10.473397065	-1.971134550
	H323	6.574086873	2.950655325	-8.369902290
	H324	6.302946129	-5.079182118	0.757246839
	H325	2.128744821	-7.811965417	0.943965822
	H326	4.634418323	-1.699830221	-6.956489271
	H327	3.186376766	-9.192444693	-0.651151292
	H328	4.274632597	-6.645893736	-6.579786216
	H329	3.139793094	-5.251883476	-4.803876270
	H330	5.800610273	-12.663996124	4.045042785
	H331	5.320822001	-1.250887110	2.868515116
	H332	8.077579263	-5.895736847	2.075986828
	H333	7.143681684	-3.809013544	1.051004995
	H334	8.304549262	-7.064019854	4.097169210
	H335	2.661414974	-2.955052468	5.543941516
	H336	4.375730308	-8.138264682	-3.268802804
	H337	5.355016633	-8.516589307	-1.846403396
	H338	2.285897963	-8.597171605	-2.052971635
	H339	2.463965692	-1.908712280	-5.874629585
	H340	-1.912612473	2.533911816	-6.363428722
	H341	-3.850444832	1.330073165	-7.421875057
	H342	2.133127119	-6.046225205	0.788943304
	H343	8.253167092	-7.423120552	1.819394748
	H344	7.184197723	-9.349177826	1.613419153
	H345	3.085436380	-1.279479037	5.625809759
	H346	6.646651657	-7.354629282	-5.885914308
	H347	7.035606639	-5.896678730	-2.132971606
	H348	1.841098131	-2.623843122	-7.392581299
	H349	5.557937655	4.378910725	-5.853612920
	H350	6.950347024	-0.076108740	5.244078526
	H351	7.295678824	0.450566153	3.581247620
	H352	9.997372331	-6.646016229	-4.997338784
	H353	9.116178277	-7.910356985	-4.445148300
	H354	10.498837877	-8.586575486	-0.183363298
	H355	9.011648010	-8.182245503	-1.864408841
	H356	12.250310102	-7.393660877	-1.904379901
	H357	10.185626266	-6.914356504	0.336302013
	H358	10.003848629	0.189360026	4.043280352
	H359	-3.597062208	13.014509606	3.753457689
	H360	9.035470418	-7.195548005	7.012110531
	H361	8.552953672	-4.733615335	5.892137240
	H362	-4.865490876	12.294109157	1.095309428
	H363	-4.659634438	10.160696732	3.314155605
	H364	-6.162613548	10.881642275	2.709284252

	N365	0.919913627	-3.321096321	-0.673969456
	H366	4.383004639	-1.396067309	0.811445468
	H367	-6.014921565	7.005730993	-2.460580688
	H368	0.008473772	-0.884221525	-0.729938230
	N369	-0.007827133	-3.824670015	-0.274755887
	H370	-1.947123663	-0.218267889	-1.260627156
	Mo371	4.220642129	-4.305923670	-1.801106426
	N372	-6.806250260	-7.094969150	-1.939857106
	N373	-8.955861281	-4.659441610	-0.082678005
	N374	-8.796561212	-5.085066950	2.687028491
	N375	-6.139698404	-5.916238438	3.173569371
	N376	-4.777572815	-3.477973592	2.677078213
	N377	-5.887614402	-1.955943723	4.859033592
	N378	-5.160650064	-3.445636321	7.231563909
	N379	-2.555092282	-4.794816096	7.027332413
	N380	-1.000676590	-2.823396650	5.886846027
	N381	-1.653045796	0.071970507	6.615425516
	N382	-2.453144714	-0.231968298	9.387417022
	N383	4.483061071	-11.820251271	5.551350049
	N384	5.549480516	-9.519233976	4.695259122
	N385	4.451262796	-3.511794914	3.689952879
	N386	3.261451335	-2.193323440	5.218404481
	N387	4.726071779	-1.213087678	3.704683702
	N388	8.188671040	-7.226858785	5.121881340
	N389	-5.214723955	-2.737496540	-4.110744677
	N390	-1.340335434	-5.444433710	-3.742958987
	N391	-5.571224212	-0.273812974	-3.288035267
	N392	-8.019316129	0.813603266	-2.461478358
	N393	-8.019348304	0.360874058	0.312044171
	N394	-6.000203459	2.062433722	1.265763671
	N395	-2.850784053	2.769664720	-1.536709826
	N396	-2.354465458	0.709746801	-1.146896676
	N397	-6.732638393	4.675740375	1.873873793
	N398	-9.870072778	7.775071845	0.146782851
	N399	-10.006862036	9.491532498	1.545944723
	N400	8.906498066	2.523597075	4.226200876
	N401	6.310995027	7.159401574	1.141439150
	N402	4.676267160	8.540766085	-1.656179989
	N403	2.149870561	7.972343109	-2.610766951
	N404	-2.262571578	6.630124068	-4.673437608
	N405	-2.239604579	8.496842673	-6.084843492
	N406	-3.617222001	8.464904430	-4.225403820
	N407	0.828627144	6.337415347	-0.645801924
	N408	0.769636730	8.134140567	1.502602127
	N409	-0.463829508	10.562827204	1.059900194
	N410	-1.264647203	10.523675246	-2.879526008
	N411	-2.995112571	11.554210631	1.711107076
	N412	5.921128683	-4.342113695	-9.504117945
	N413	3.880101342	-2.246099716	-7.382564764
	N414	2.743338344	0.529131548	-6.893892216
	N415	4.823975350	2.486281231	-6.486981965
	N416	0.188463428	-2.538755109	-10.340061220
	N417	-1.638924971	-0.776303324	-8.994484663
	N418	9.056065081	-6.920162112	-4.702463683
	N419	4.981525023	-5.498521812	-3.703556878
	N420	4.727933584	-6.368092221	-5.713528058
	N421	9.660872742	-7.414932875	-1.670599768
	N422	-0.259941140	2.454943817	0.463150203
	N423	0.595070654	1.973916807	-0.070612797
	O424	-6.760679048	-5.186773966	0.207763628
	O425	-7.949689352	-2.982888035	2.956157081
	O426	-6.438394226	-5.430888216	5.386478949
	O427	-3.289904589	-4.140148881	4.267409484
	O428	-3.943210856	-0.778399659	4.826591398
	O429	-4.916527353	-1.403841019	8.238478218
	O430	-2.356247867	-4.404854852	9.277032256
	O431	0.941351489	-3.986066419	6.303559687
	O432	0.610046275	-0.050550793	6.902939595
	O433	-0.972261250	1.442160851	9.784600304
	O434	-0.790004279	-0.795818751	12.513838272
	O435	6.584942859	-10.503198149	2.876819393
	O436	6.852743196	-8.048965064	6.802443220
	O437	10.536962281	-4.815468529	6.358947889
	O438	-5.544443188	-0.797134460	-1.053650142
	O439	-2.862546619	-4.151150388	-4.831645730
	O440	-6.893676093	2.597046771	-1.585671544
	O441	-5.571869407	3.272693921	-4.312109163
	O442	-10.015142518	1.476416541	0.510303664
	O443	-6.306510841	1.313022159	3.401376818
	O444	-4.860755715	4.928401054	3.157560495

	O445	-7.411818069	7.108000492	4.361692474
	O446	9.848209235	-0.376130130	5.997772213
	O447	2.742132757	4.422652310	4.734225922
	O448	3.689680395	8.355179310	0.392908977
	O449	1.643651757	10.202727790	-2.866784374
	O450	-1.108620677	7.241861671	-1.457056807
	O451	-1.105735472	7.076399853	2.297081662
	O452	2.091394849	5.588478613	2.137442880
	O453	0.417184003	11.447007502	2.983100444
	O454	-2.528728484	13.578635863	0.773827481
	O455	-2.927014689	11.089637763	-1.418164209
	O456	-5.497595961	13.595591500	3.281677370
	O457	-5.141062243	6.986713638	-2.024332671
	O458	3.355160757	-4.205445232	-8.460949215
	O459	1.331144562	-0.370983220	-8.460189043
	O460	3.704507291	3.924472811	-7.842961566
	O461	7.333972425	4.739215243	-7.744047450
	O462	-3.262991988	-2.185226784	-9.751956727
	O463	-0.328321068	-6.325303196	-6.459131755
	O464	1.688910124	-5.445695517	-6.819605982
	O465	-2.331714199	2.728857548	-8.958201779
	O466	10.437878500	-5.322707642	-2.062713415
	O467	12.787578898	-6.851633011	-0.010742774
	O468	0.561069222	-6.474560009	-1.617641931
	O469	-0.334167800	-7.416331122	0.216008821
	O470	5.649079247	-10.806593685	-2.777146713
	O471	3.441132654	-10.772569052	-3.263693515
	O472	5.260904394	-5.818561457	-0.565703995
	O473	4.921245276	-7.590725990	0.775107097
	O474	2.962767326	-6.025870789	-1.789099225
	O475	8.606861718	-6.674441101	2.376264663
	O476	6.800287651	-4.615618192	1.481507461
	O477	-3.829037458	4.921211699	-2.988279118
	O478	8.053619641	-10.379684914	-1.510633546
	O479	7.410189673	-8.685556025	0.914080849
	S480	3.886692962	5.026558644	-1.409213270
	S481	2.569246323	2.072547827	-2.789426836
	S482	2.702718414	-3.235770696	-3.275076794
	S483	3.699558345	1.669501770	0.623755407
	S484	0.907151180	-0.718866037	-1.743258727
	S485	4.966716096	-0.561357152	-4.718836658
	S486	3.535280203	-3.495136731	0.315359201
	S487	6.094353476	1.888197160	-2.057885002
	S488	6.168255485	-2.992923438	-2.086119727
	S489	6.588449626	-0.840262700	0.552178190
	end			

S2BH-2N2x-6N2x-7Hn-6H2n (19)

Fe(139) -2.434	2bh7h2n6n2x6h2ndtc.car_3			
Fe(140) -1.189	C1	-7.723696989	-5.509427589	-0.35079954
	C2	-7.904282260	-6.591523449	-1.42056787
Fe(141) -2.449	C3	-8.818262486	-3.702587427	0.90387668
Fe(142) 2.553	C4	-8.414666072	-4.058152654	2.33236334
	C5	-8.267192329	-5.778303974	4.06527537
Fe(143) 2.773	C6	-6.748290870	-5.873499642	4.24452775
Fe(144) 0.548	C7	-4.562530067	-6.068468780	3.17939539
	C8	-4.055960222	-4.640856946	3.40142222
Fe(145) -0.018	C9	-4.385601223	-2.266076749	2.87995514
	C10	-4.626771889	-1.781117211	4.31721023
	C11	-6.033077427	-1.790183958	6.28310349
**S=0**	C12	-5.118591697	-2.415286591	7.34597000
	C13	-4.243079673	-4.503777380	8.28880753
	C14	-2.723764493	-4.710402815	8.19172131
	C15	-0.805555164	-5.235192604	6.73918284
	C16	0.008145629	-4.046404633	6.21784466
	C17	-0.165061146	-1.733799711	5.33547537
	C18	-0.236507571	-0.583523685	6.35317513
	C19	-0.766973418	-1.423895235	3.94395042
	C20	-0.291830988	-0.072830190	3.39883463
	C21	-0.444840482	-2.548566320	2.95470658
	C22	-1.631412762	1.021458245	7.57351304
	C23	-1.505580676	0.572319707	9.04179113
	C24	-2.938610426	1.796264726	7.28354513
	C25	-3.178091181	2.881259427	8.33890200
	C26	-2.896406622	2.412152199	5.87828355
	C27	-2.196550063	-0.932628768	10.81062403
	C28	-0.783641569	-1.183468524	11.30071431
	C29	5.425623369	-11.800367016	3.73833917
	C30	6.125046198	-10.483910752	3.42216324

	C31	6.273371575	-8.114483469	4.16370342
	C32	7.338764633	-7.776774087	5.22313544
	C33	5.048339472	-7.193129708	4.28683419
	C34	5.405688597	-5.725898082	4.06662963
	C35	4.171667442	-4.846503965	3.87743027
	C36	4.315501128	-2.342789104	4.05340836
	C37	9.534456235	-6.754254469	5.59788035
	C38	9.698369599	-5.256198031	5.73196228
	C39	-4.965601695	-2.555868991	-2.63137024
	C40	-5.403474068	-1.160738496	-2.14700618
	C41	-3.470307960	-2.761926967	-2.31694523
	C42	-2.968583021	-4.210976511	-2.49387252
	C43	-2.394175153	-4.533410123	-3.87199582
	C44	-5.786677978	1.142531378	-2.94184062
	C45	-7.024867862	1.513258633	-2.11572946
	C46	-5.745369722	1.854697525	-4.30034627
	C47	-9.310292725	0.909431663	-1.47220577
	C48	-9.153592923	0.805565291	0.04902398
	C49	-7.725963600	0.156799734	1.93476637
	C50	-6.609557189	1.089814467	2.40528498
	C51	-4.996083321	2.850293501	1.87848592
	C52	-5.571317687	4.094082568	2.59723339
	C53	-4.205695410	3.398049113	0.67957271
	C54	-3.450303435	2.407412335	-0.14084973
	C55	-3.049784931	1.111471760	0.06476290
	C56	-2.351751197	1.721389258	-1.96267088
	C57	-7.398861709	5.746437220	2.55131261
	C58	-7.511354902	5.814150871	4.07244490
	C59	-8.799013086	5.820442072	1.89369918
	C60	-9.409139325	7.185923606	1.76692933
	C61	-9.504896273	8.220304823	2.67746748
	C62	-10.480890515	8.779298491	0.76821086
	C63	8.569654334	1.489715239	4.94381708
	C64	9.637116766	0.416335732	4.83514915
	C65	7.237830060	0.841848026	4.48538685
	C66	6.056089678	1.776644284	4.58745437
	C67	5.295812389	1.869032772	5.76782648
	C68	5.680556992	2.582979781	3.49927631
	C69	4.192916695	2.725895570	5.85954672
	C70	4.594198470	3.454265850	3.58086412
	C71	3.845211602	3.517921208	4.75918663
	C72	5.416453227	7.011244734	-0.00827380
	C73	4.374177646	8.105737071	-0.29473822
	C74	4.739122189	5.648789055	0.11649836
	C75	3.598620976	9.807762368	-1.90965511
	C76	2.176982872	9.437548509	-2.33879678
	C77	0.603652739	7.538783636	-2.70143283
	C78	-0.075831194	7.059392788	-1.39332257
	C79	0.808902739	6.372193384	-3.69835597
	C80	-0.389425099	5.496188335	-4.08143401
	C81	-1.390822645	6.086863510	-5.08632683
	C82	-3.024112505	7.936806472	-4.72432172
	C83	0.111343678	5.937285864	0.77138639
	C84	-0.234826620	7.081869780	1.73571966
	C85	1.016874117	4.942991430	1.52966662
	C86	0.666141062	9.087480423	2.81199519
	C87	0.046493570	10.448553216	2.52923342
	C88	-1.093857079	11.853621875	0.89058384
	C89	-2.464379183	12.351261730	1.40600699
	C90	-0.997750153	11.978749562	-0.63287416
	C91	-2.031013371	11.160399041	-1.39869707
	C92	-4.615948917	11.745344263	2.41376788
	C93	-4.714161707	12.758736280	3.54415260
	C94	-5.334863321	10.427483679	2.82193410
	C95	-5.382226347	9.431552699	1.68451813
	C96	-6.421888229	9.489134179	0.74242165
	C97	-4.372553747	8.473933165	1.49653016
	C98	-6.439342809	8.644571017	-0.36841914
	C99	-4.360771412	7.635239728	0.37714264
	C100	-5.396518215	7.734584863	-0.54675992
	C101	5.557352899	-3.274555746	-8.47102471
	C102	4.070728814	-3.074282038	-8.24068467
	C103	2.386561176	-1.643269104	-7.10611782
	C104	1.965569231	-0.250417396	-7.58855429
	C105	2.332397426	2.160878557	-7.30763690
	C106	3.512113588	3.128151476	-7.25734050
	C107	5.674517746	3.771720827	-6.35332299
	C108	6.401779631	4.127974775	-7.63305674
	C109	-1.187194904	-2.819194091	-9.71013313
	C110	-2.273841382	-1.767932576	-9.46156090

C111	-0.907990333	-3.597610828	-8.40507085
C112	-0.098412933	-4.879741465	-8.67394209
C113	0.370176122	-5.485930965	-7.36906852
C114	-2.818984434	0.500582293	-8.73844751
C115	-2.275467378	1.827408990	-9.23015431
C116	-3.423359834	0.532761131	-7.31336877
C117	-2.339214505	0.515224204	-6.27375795
C118	-1.697028909	1.703517139	-5.88601597
C119	-1.846383026	-0.711511320	-5.79771039
C120	-0.540798698	1.655504032	-5.10250815
C121	-0.692965131	-0.757381951	-5.01211538
C122	-0.018074019	0.425724392	-4.69232160
C123	8.716685560	-5.814144352	-3.89803180
C124	9.732603081	-5.947760840	-2.75275574
C125	7.312709441	-6.181324734	-3.39261590
C126	6.176451254	-5.924572939	-4.32930059
C127	6.001574531	-6.441292642	-5.59706719
C128	4.153966120	-5.391021906	-4.97806949
C129	10.652472141	-7.252730553	-0.93294477
C130	12.087328235	-6.916939951	-1.27445765
C131	0.790507005	-7.094561598	-0.65529852
C132	2.193499707	-7.013353051	-0.06061191
C133	3.324716033	-7.022800844	-1.12502790
C134	3.395043929	-8.421976425	-1.79254826
C135	4.603299736	-8.652823540	-2.70058836
C136	4.715927694	-10.098935173	-3.22854086
C137	4.683729092	-6.755743500	-0.46104257
C138	3.901413407	-0.467641465	-1.67170873
Fe139	4.325447536	-1.751448954	-3.11692009
Fe140	2.423316408	0.949460850	-1.33333984
Fe141	5.215728934	0.579656238	-0.61743511
Fe142	4.450869672	0.777556644	-3.08744446
Fe143	4.208892075	2.897534439	-1.42636956
Fe144	4.693082767	-1.896499114	-0.60814661
Fe145	2.172516695	-2.511967108	-0.96110496
H146	-6.115666154	-7.541519472	-1.17267245
H147	-6.123108015	-6.483850884	-2.41626451
H148	-9.703259368	-4.979871746	-0.56070565
H149	-8.495378713	-6.174140277	-2.25175097
H150	-8.533568018	-7.379100876	-0.97021756
H151	-8.104470338	-2.939297136	0.56113410
H152	-9.138138020	-5.959623570	2.10702547
H153	-9.814914088	-3.242087803	0.92916842
H154	-8.707038781	-6.768949262	4.24104302
H155	-6.468676072	-6.137865991	2.21765632
H156	-8.631530993	-5.098494879	4.84628438
H157	-3.335632355	-2.055272798	2.64079196
H158	-5.407157160	-3.940011558	2.01517631
H159	-4.160774909	-6.459234355	2.23469105
H160	-5.919519684	-0.700793475	6.35936387
H161	-6.458839760	-2.730615903	4.38787079
H162	-4.178221103	-6.681352232	4.00359477
H163	-5.015229929	-1.690919238	2.18724953
H164	-5.348958403	-4.267933566	6.47210117
H165	-7.073041649	-2.054135977	6.52037016
H166	-0.805967725	-3.524007572	3.30642788
H167	-0.920903901	-2.354366557	1.98045597
H168	0.641554741	-2.619319084	2.78456515
H169	-1.857839021	-1.369841544	4.07489786
H170	-4.712900603	-5.497525135	8.33311742
H171	-2.772402940	1.652691881	5.09446818
H172	-2.920899567	-0.942127606	8.80313401
H173	-3.312548206	2.460349978	9.34595650
H174	0.770434871	-0.102242851	3.10745562
H175	-0.424700546	0.745404506	4.12207966
H176	8.466721811	1.752648595	6.01419627
H177	-3.831704426	2.950606526	5.67000186
H178	-4.081188400	3.455109010	8.08557881
H179	-2.065142352	3.132033483	5.79819962
H180	-2.328860403	3.581948418	8.37498339
H181	-3.775901539	1.076376389	7.32773163
H182	-0.778669492	1.700150345	7.42291330
H183	-2.290854597	-0.429481032	6.12615451
H184	-0.873624356	0.193239828	2.50206986
H185	1.452416927	4.234946468	0.80904246
H186	0.369280405	4.383523464	2.22119843
H187	2.163621016	5.180904598	3.11312571
H188	2.299783789	4.306152969	5.63148360
H189	4.302780504	4.061049447	2.72323244
H190	6.227753947	2.507166833	2.55718246

	H191	5.560592191	1.252010133	6.63104864
	H192	-1.734669492	-3.036696966	5.96633190
	H193	-0.682220211	-6.058300319	6.02012206
	H194	-0.357057843	-5.549654503	7.69084472
	H195	-2.832249244	-4.996361001	6.13067231
	H196	-4.419387948	-3.992937836	9.24286258
	H197	3.600679718	2.767456104	6.77751026
	H198	0.907140025	-1.924428624	5.21507880
	H199	-2.682281782	-0.205889047	11.48227597
	H200	-2.738913993	-1.888460927	10.88541212
	H201	-0.058261144	-1.485898019	10.50933372
	H202	10.484945175	-7.195257058	5.25879557
	H203	-0.960251436	-5.661455520	-4.80342141
	H204	1.620262926	-6.153905880	-2.11660188
	H205	-0.739352230	-5.615390072	-9.18131648
	H206	0.760335214	-4.661908965	-9.32488383
	H207	-1.856139070	-3.872040318	-7.91744843
	H208	-1.543684004	1.739022254	-10.06730375
	H209	-1.661307065	-3.518164976	-10.41586943
	H210	-2.878843316	-2.081372870	-2.95219341
	H211	-3.797772969	-4.918547960	-2.32017418
	H212	-0.866730840	-0.395558999	-8.78478695
	H213	-3.658114933	0.289770130	-9.43334987
	H214	0.563790193	-1.696740475	-9.75586285
	H215	-0.897354534	-5.739093726	-3.05079296
	H216	-0.373439053	-2.939774845	-7.70184100
	H217	0.555558367	-2.985055695	-10.78337616
	H218	-6.118798452	-3.229842174	-4.20893437
	H219	-3.312397126	-2.469882503	-1.26743914
	H220	2.139233407	-4.700994999	-7.70266081
	H221	-2.220842522	-4.434260627	-1.72254775
	H222	-4.330878758	2.363505019	2.60534257
	H223	-3.205519002	0.441415127	0.90069773
	H224	-8.634458639	0.448908278	2.47952157
	H225	-7.332836920	-0.179001586	-0.13550278
	H226	-7.302205437	3.873465402	1.51781957
	H227	-9.139165067	8.337968698	3.69131056
	H228	-7.695100313	4.823506762	4.55409766
	H229	-9.478851797	5.127601814	2.41999965
	H230	-6.773221842	6.585749816	2.20781029
	H231	-11.019348002	9.384360369	0.04267583
	H232	-3.500339727	4.146174154	1.07681841
	H233	-4.902632018	3.934594484	0.01580048
	H234	-10.417743957	10.136142888	2.40767176
	H235	-8.698351160	5.434195968	0.86745710
	H236	-6.362368424	1.908702750	0.50355676
	H237	-7.475105780	-0.863564722	2.25975991
	H238	-9.725241282	1.908420934	-1.66677621
	H239	-1.880598915	1.695803930	-2.94293402
	H240	-10.055411545	0.170446555	-1.79442689
	H241	9.689065498	3.148443975	4.50524697
	H242	8.136949146	3.255530631	4.00301337
	H243	1.655056964	6.088674225	-0.73058393
	H244	-0.837690552	5.430674664	0.54521705
	H245	1.492952076	8.032031009	1.17908857
	H246	-3.245770318	3.653968023	-1.89791587
	H247	-4.684466968	4.364920488	-3.27308657
	H248	-6.010940381	3.684197462	-4.95378499
	H249	-4.548413731	5.536828059	-2.20473742
	H250	-4.816943088	1.555504003	-4.81456171
	H251	-6.606489224	1.545205635	-4.91264792
	H252	0.025747443	11.739792582	-0.95409495
	H253	-1.180733892	13.035143155	-0.88541212
	H254	-0.551476167	10.446189230	-2.66636479
	H255	-2.204861133	9.913655578	-2.97498225
	H256	-2.909055857	9.601739770	-5.91393114
	H257	-1.770973134	5.291331070	-5.74518927
	H258	-7.263334183	8.690543796	-1.08481184
	H259	-3.567866661	8.359338407	2.22799120
	H260	-4.283657737	7.986388754	-3.09388067
	H261	1.570007968	5.705152715	-3.26086382
	H262	2.689094327	7.467082471	-2.26410371
	H263	-0.922594233	5.142696616	-3.18472256
	H264	-8.015545233	-0.120043250	-2.84260870
	H265	4.072828740	10.351986590	-2.73674643
	H266	-5.499974356	-0.722105983	-4.08939930
	H267	1.258821540	6.794713083	-4.61188181
	H268	-3.531777760	6.943881385	0.21803082
	H269	3.487515417	10.508270329	-1.06867946
	H270	-2.078724513	8.216367524	-6.53355041

H271	-4.556894276	9.218365914	-4.28042283
H272	-4.477615518	-3.226790360	-4.52002934
H273	-5.532133957	-3.270413904	-2.00499279
H274	-7.236649071	10.204857382	0.87689370
H275	-4.947492913	1.506485543	-2.33344783
H276	-0.045796997	8.314343330	-3.11826766
H277	-0.877455229	6.816917528	-5.72532576
H278	0.036393874	4.599644021	-4.55235888
H279	-3.067481950	6.140387316	-3.76865577
H280	0.145979475	8.659744986	3.68149023
H281	-0.653011059	9.748577482	0.74239539
H282	-0.383894305	12.577620227	1.31976529
H283	1.712367712	9.266268725	3.09719989
H284	-2.920742119	10.488273060	2.06327372
H285	6.073317865	-2.303219127	-8.46119613
H286	5.922206579	-3.843394604	-7.59075721
H287	8.731086727	-4.749195058	-4.17456441
H288	6.758487668	-4.258840500	-9.79094794
H289	5.208798861	-4.784515545	-9.79156944
H290	5.515904044	4.906798814	0.34084289
H291	5.486106205	7.445865723	1.98585474
H292	5.185326389	8.402077941	-2.16209479
H293	4.008814963	5.667287611	0.93726889
H294	6.678293809	8.166602572	1.12983114
H295	6.413652136	3.336293619	-5.66300240
H296	6.140695342	6.971721819	-0.83680943
H297	4.581552132	-7.342076367	5.27490885
H298	6.701495983	-8.004949206	3.15905033
H299	4.667286920	1.923634183	-6.00908525
H300	4.555939404	-0.315997375	3.92651908
H301	1.930498639	2.180513295	-8.32739382
H302	-0.306932401	-1.718432258	-4.66466316
H303	1.556253747	2.592468815	-6.65257154
H304	3.586021417	-4.788089981	4.80193779
H305	3.140918695	0.532625839	-6.12114175
H306	3.514445294	-5.276055161	3.10374817
H307	4.325145671	-7.520396326	3.52247607
H308	6.028435443	-5.636347064	3.16417469
H309	5.998814570	-5.336313432	4.90933451
H310	-4.080014716	-0.343212074	-7.21027319
H311	5.263299240	-9.784058304	5.08651092
H312	4.610900465	-11.895560915	3.00418203
H313	0.905448029	0.407677453	-4.11633488
H314	5.546067895	-12.210835010	5.75430695
H315	4.060499222	-12.484187539	5.12121636
H316	5.191403457	-3.480349301	2.63183042
H317	-0.021455770	2.571336787	-4.81448474
H318	-2.356394607	-1.641305998	-6.06087874
H319	8.025637530	-9.676384923	-1.12109195
H320	7.297737444	-7.255782285	-3.13955760
H321	6.729558032	-8.057796733	0.39414328
H322	7.375325438	-10.237136535	-2.42067409
H323	6.292978519	3.390986974	-8.46387851
H324	6.432114417	-4.966048326	0.42301974
H325	2.323770895	-7.823971235	0.66632484
H326	4.488678221	-1.418601373	-7.11744229
H327	3.408884275	-9.162012884	-0.97676094
H328	4.260641627	-6.362937884	-6.84414012
H329	3.134458011	-5.031061866	-5.01129930
H330	6.144236205	-12.608674412	3.51993545
H331	5.373441389	-1.193499622	2.66593400
H332	8.234554380	-5.728275690	1.73131275
H333	7.153659419	-3.638470677	0.77581093
H334	8.520186474	-6.941030742	3.71778332
H335	2.913055661	-3.042213394	5.43782137
H336	4.536532353	-7.984116147	-3.57306949
H337	5.534123983	-8.400180687	-2.17435655
H338	2.466033659	-8.583426958	-2.36070492
H339	2.332039723	-1.702761749	-6.00719576
H340	-2.086229981	2.661095030	-6.23959210
H341	-4.043601301	1.439182310	-7.24566174
H342	2.233764642	-6.061689316	0.49595337
H343	8.441714362	-7.243548079	1.43675027
H344	7.415148831	-9.184199870	1.18962862
H345	3.343562220	-1.376084121	5.57344554
H346	6.661203030	-7.035164800	-6.21692837
H347	7.126330852	-5.651633041	-2.45176346
H348	1.703588697	-2.374482135	-7.54338566
H349	5.289775134	4.694336558	-5.89094889
H350	7.067496507	-0.047852601	5.10874762

	H351	7.355209318	0.503420125	3.44315936
	H352	10.015863567	-6.278220498	-5.41337257
	H353	9.195425245	-7.578401833	-4.85482246
	H354	10.634956705	-8.305924583	-0.61034337
	H355	9.135010689	-7.908246553	-2.27786506
	H356	12.368151775	-7.090850768	-2.34132259
	H357	10.320173520	-6.637528052	-0.08179217
	H358	10.082325469	0.311372328	3.81584735
	H359	-3.816390635	12.819319943	4.20373080
	H360	9.324343578	-7.148365012	6.60830422
	H361	8.775172781	-4.656171295	5.53772172
	H362	-5.124710726	12.175491393	1.53555274
	H363	-4.824488200	9.993800264	3.69587000
	H364	-6.354523465	10.694842880	3.13433493
	H365	4.046469201	-1.270074467	0.60252086
	H366	-6.243876530	6.914857126	-2.09816788
	H367	0.165757713	-0.600839350	-0.58731465
	H368	2.480852038	-1.504444635	0.41292870
	H369	1.738914795	-1.873366255	0.52735010
	H370	-1.928774561	-0.185026217	-1.22963060
	Mo371	4.213003014	-4.216065433	-1.98209094
	N372	-6.690201308	-7.191603530	-1.94358921
	N373	-8.857981132	-4.828810858	-0.01553398
	N374	-8.684245429	-5.316430938	2.75156268
	N375	-6.016324460	-6.139224488	3.13330017
	N376	-4.680899994	-3.676552201	2.67803891
	N377	-5.747019299	-2.231345667	4.92763235
	N378	-4.883833385	-3.747885604	7.22130379
	N379	-2.220290841	-4.979065229	6.95200949
	N380	-0.722138060	-2.951850250	5.90041948
	N381	-1.473399427	-0.099556720	6.64585138
	N382	-2.228655300	-0.512240869	9.42156924
	N383	4.856760635	-11.845563197	5.09332675
	N384	5.855668993	-9.498135899	4.29984344
	N385	4.574080347	-3.506595505	3.44779923
	N386	3.538772126	-2.282150419	5.15751587
	N387	4.869541333	-1.215918650	3.57450097
	N388	8.434480265	-7.140184333	4.73912493
	N389	-5.251392101	-2.709726335	-4.06397399
	N390	-1.348462238	-5.380150284	-3.89585500
	N391	-5.636283946	-0.285837194	-3.16123633
	N392	-8.098436570	0.701683901	-2.24790201
	N393	-8.034151601	0.185241600	0.51720633
	N394	-6.021434109	1.898215305	1.46927654
	N395	-3.000589488	2.752945490	-1.40825671
	N396	-2.381503628	0.716156452	-1.07779664
	N397	-6.801731361	4.473313388	2.16876683
	N398	-10.022954830	7.547574370	0.57881459
	N399	-10.184775405	9.224951413	2.02155656
	N400	8.930802681	2.612719317	4.07564042
	N401	6.161347846	7.288625194	1.23066093
	N402	4.456813061	8.703897878	-1.52047539
	N403	1.918452969	8.118230067	-2.45109821
	N404	-2.540422649	6.728125421	-4.43883203
	N405	-2.560058811	8.661085925	-5.75985113
	N406	-3.938084269	8.504565294	-3.90520474
	N407	0.702872700	6.405735960	-0.48446237
	N408	0.625769220	8.139136415	1.71307501
	N409	-0.683349800	10.546774704	1.37677317
	N410	-1.563187669	10.567973285	-2.53090251
	N411	-3.224994806	11.458516476	2.08744124
	N412	5.787573285	-3.940296580	-9.75649662
	N413	3.737306702	-1.968478993	-7.54439215
	N414	2.582236778	0.774211125	-6.95595611
	N415	4.621007301	2.788541420	-6.55187624
	N416	-0.026782142	-2.238958861	-10.39731730
	N417	-1.858357001	-0.558814961	-8.98304651
	N418	9.093469030	-6.587300711	-5.09453576
	N419	5.007126687	-5.259540067	-3.96060442
	N420	4.727365284	-6.089243960	-5.98361773
	N421	9.773457805	-7.131323122	-2.08244627
	N422	0.110028570	2.525150991	-0.39078635
	N423	-0.251795375	-4.047017999	-0.33513749
	N424	1.015287890	1.961825357	-0.76731662
	N425	0.682983727	-3.509121623	-0.65785418
	O426	-6.640948938	-5.289185019	0.22140414
	O427	-7.864101688	-3.208878545	3.05577076
	O428	-6.242378504	-5.725800984	5.36914576
	O429	-3.144764475	-4.382783386	4.20313697
	O430	-3.826602816	-1.018284975	4.88120711

	O431	-4.674860593	-1.729919099	8.28361322
	O432	-2.032153365	-4.681779954	9.21704362
	O433	1.243310840	-4.137297225	6.09389060
	O434	0.800167938	-0.146305718	6.87679698
	O435	-0.774771630	1.179486767	9.84286790
	O436	-0.463188490	-1.130859198	12.47853459
	O437	6.842417013	-10.383337735	2.40650188
	O438	7.166877879	-8.066219220	6.42016254
	O439	10.748460705	-4.718596746	6.04792446
	O440	-5.523222594	-0.860620259	-0.94304089
	O441	-2.891943737	-4.057082560	-4.91806559
	O442	-7.008739956	2.496506157	-1.35407831
	O443	-5.776729489	3.281104201	-4.09859263
	O444	-10.038566919	1.271972967	0.77822572
	O445	-6.272933458	1.094685950	3.59269614
	O446	-4.907026867	4.734309117	3.41703918
	O447	-7.461044335	6.844241862	4.72155541
	O448	9.981990864	-0.305610964	5.75917836
	O449	2.760215289	4.373903723	4.77484955
	O450	3.516615761	8.442251387	0.54011304
	O451	1.351598454	10.344307906	-2.56342919
	O452	-1.284667405	7.273023777	-1.20787030
	O453	-1.195115909	6.989175719	2.51134137
	O454	2.054668798	5.618481079	2.24408202
	O455	0.199725187	11.389314260	3.31787263
	O456	-2.801856114	13.531686312	1.23697363
	O457	-3.216212778	11.093252197	-1.04347977
	O458	-5.710739286	13.433370739	3.74927551
	O459	-5.352597201	6.920329538	-1.69836658
	O460	3.237844724	-3.918896565	-8.65159088
	O461	1.140881112	-0.095955711	-8.51219007
	O462	3.407220730	4.227587142	-7.82521820
	O463	7.093770577	5.126855811	-7.74898240
	O464	-3.473877877	-2.016260244	-9.66346745
	O465	-0.363141950	-6.134540390	-6.62933973
	O466	1.642671600	-5.234602382	-7.00618738
	O467	-2.625589967	2.920184134	-8.80751490
	O468	10.513737866	-5.022318405	-2.45770491
	O469	12.913841461	-6.556746590	-0.44780799
	O470	0.644713109	-6.506986287	-1.85228380
	O471	-0.156978393	-7.609226410	-0.06666339
	O472	5.876654138	-10.640401971	-3.20462439
	O473	3.664280799	-10.639320746	-3.67311534
	O474	5.379997627	-5.756500876	-0.94374707
	O475	5.093505018	-7.492646183	0.45508861
	O476	3.035161315	-5.993292400	-2.07170537
	O477	8.785897778	-6.500522960	2.00718421
	O478	6.901766121	-4.501315873	1.16029695
	O479	-4.025023190	4.923786953	-2.77922968
	O480	8.265876345	-10.100716608	-1.97457472
	O481	7.615922895	-8.495758582	0.50633045
	S482	3.859498776	5.163403241	-1.43910281
	S483	2.781910872	2.293517608	-3.10031050
	S484	2.501535246	-3.110245799	-3.17583722
	S485	3.664065895	1.827384841	0.43730063
	S486	0.897029697	-0.750982251	-1.72709515
	S487	4.835191394	-0.473609463	-4.84927177
	S488	3.642975615	-3.699189237	0.21287954
	S489	6.177316110	1.918976436	-2.10074913
	S490	6.084622529	-2.793250946	-2.23135046
	S491	6.360546398	-0.879357159	0.57533061
	end			

TS for 6H2n dissociation (19) to (19d)

	2bh7h2n6n2x6h2ndtd_1_53339.82			
Fe(139) -2.448	C1	-7.727274701	-5.511934438	-0.355441196
Fe(140) -1.144	C2	-7.924661950	-6.588900596	-1.427221227
Fe(141) -2.541	C3	-8.797530660	-3.696596786	0.906810353
Fe(142) 2.387	C4	-8.389342694	-4.049878221	2.334145664
Fe(143) 2.647	C5	-8.228535206	-5.767414251	4.068686136
Fe(144) 0.704	C6	-6.709354849	-5.856598909	4.245309578
Fe(145) 0.356	C7	-4.523909848	-6.041356527	3.181581892
	C8	-4.037278736	-4.606697774	3.402196746
	C9	-4.390985940	-2.239207650	2.854253491
	C10	-4.633808406	-1.746448422	4.289059626
**S=0**	C11	-6.025560238	-1.758753753	6.263407588
	C12	-5.110415638	-2.377606811	7.329977003
	C13	-4.235028353	-4.463047912	8.283500778
	C14	-2.718552492	-4.692242099	8.186546654

	C15	-0.811863472	-5.271609547	6.740250785
	C16	0.002477026	-4.078838842	6.226915829
	C17	-0.184358306	-1.775905623	5.319459480
	C18	-0.247852179	-0.626546288	6.339111994
	C19	-0.815586067	-1.474675933	3.937303195
	C20	-0.420977582	-0.098611594	3.381481288
	C21	-0.473631605	-2.581106391	2.932855610
	C22	-1.620979805	0.995720607	7.557402259
	C23	-1.486720023	0.557267555	9.028530259
	C24	-2.923544852	1.780143667	7.271789517
	C25	-3.155283998	2.863770737	8.329978230
	C26	-2.879134453	2.400566115	5.868372447
	C27	-2.181374033	-0.920732433	10.819259371
	C28	-0.768567000	-1.172026203	11.308668787
	C29	5.432584867	-11.802216915	3.769775395
	C30	6.125267049	-10.485114598	3.439591613
	C31	6.266475106	-8.109180933	4.167306674
	C32	7.327992186	-7.763767622	5.228655476
	C33	5.035507957	-7.195305741	4.283635189
	C34	5.382058330	-5.726197483	4.062006226
	C35	4.140638881	-4.857667923	3.869309850
	C36	4.312090614	-2.359417593	4.081937267
	C37	9.535745342	-6.768004621	5.603593787
	C38	9.714470661	-5.271596395	5.738669285
	C39	-4.967003578	-2.561478355	-2.610314700
	C40	-5.406936326	-1.166418886	-2.128536238
	C41	-3.466680525	-2.754736942	-2.308618825
	C42	-2.955484298	-4.201638962	-2.478006173
	C43	-2.393959664	-4.532472010	-3.859340451
	C44	-5.792347039	1.134431255	-2.928352394
	C45	-7.031725663	1.504721645	-2.103593749
	C46	-5.751221045	1.844391692	-4.287973805
	C47	-9.317401544	0.897417170	-1.462343584
	C48	-9.162213285	0.799871703	0.059233695
	C49	-7.736991351	0.161626090	1.949553581
	C50	-6.623589164	1.099782508	2.417498670
	C51	-4.993840978	2.842118338	1.880900786
	C52	-5.569588871	4.087067662	2.596564065
	C53	-4.208016814	3.389248492	0.679062997
	C54	-3.446431166	2.400894420	-0.137710363
	C55	-3.040232112	1.107496666	0.071139864
	C56	-2.336752939	1.718601000	-1.954155272
	C57	-7.393838209	5.742324109	2.539532163
	C58	-7.514214605	5.808109943	4.060360418
	C59	-8.790304494	5.816082759	1.874285459
	C60	-9.406134037	7.179448633	1.751831604
	C61	-9.499217767	8.214180956	2.662146320
	C62	-10.493708150	8.766664146	0.760584815
	C63	8.562651921	1.484145937	4.949551964
	C64	9.624354836	0.404043942	4.848228146
	C65	7.228806607	0.839230968	4.495382667
	C66	6.048900053	1.776179456	4.597615980
	C67	5.288801439	1.869437405	5.778116990
	C68	5.674758071	2.583588040	3.509676348
	C69	4.188583019	2.729643500	5.870845084
	C70	4.591288035	3.458297602	3.592553512
	C71	3.843553475	3.524679283	4.771706290
	C72	5.407080482	6.996577703	-0.003560599
	C73	4.370048291	8.095626592	-0.293944248
	C74	4.723849578	5.637706615	0.122020841
	C75	3.598992909	9.794710012	-1.913489970
	C76	2.177867397	9.421689995	-2.341692161
	C77	0.606166776	7.521581135	-2.702361383
	C78	-0.074974824	7.050810000	-1.391632788
	C79	0.809388441	6.351324596	-3.694953628
	C80	-0.391496442	5.477815564	-4.075220064
	C81	-1.390983151	6.069183192	-5.081825041
	C82	-3.017355194	7.925496105	-4.716076297
	C83	0.110509055	5.940331389	0.779835710
	C84	-0.229720213	7.089738616	1.740175130
	C85	1.013256837	4.946327547	1.541848344
	C86	0.680479272	9.093530237	2.810582342
	C87	0.059087970	10.451665580	2.519921846
	C88	-1.089592641	11.840919344	0.875782459
	C89	-2.460905340	12.337704126	1.391114800
	C90	-0.998274674	11.962604224	-0.647844494
	C91	-2.032147478	11.139295312	-1.406871844
	C92	-4.608243657	11.734426433	2.408000568
	C93	-4.707448299	12.750556736	3.536180283
	C94	-5.328698127	10.418080300	2.817294840

	C95	-5.378437180	9.421540686	1.680661185
	C96	-6.421288815	9.476174575	0.741852989
	C97	-4.367574690	8.465705841	1.490384444
	C98	-6.440747587	8.630443644	-0.368265288
	C99	-4.357389741	7.626441850	0.371525860
	C100	-5.396385228	7.722676151	-0.548988386
	C101	5.558074605	-3.268583452	-8.479110829
	C102	4.072824078	-3.067091697	-8.240230996
	C103	2.392421197	-1.639859350	-7.095196850
	C104	1.964202552	-0.249974215	-7.581409625
	C105	2.325787650	2.163664769	-7.304137460
	C106	3.500280372	3.137272751	-7.245043732
	C107	5.657371438	3.786418964	-6.330762385
	C108	6.418526912	4.114830888	-7.598298325
	C109	-1.193698358	-2.814137679	-9.708475891
	C110	-2.280137009	-1.763436006	-9.457100889
	C111	-0.912466987	-3.592591029	-8.403306602
	C112	-0.102055536	-4.874167179	-8.672868769
	C113	0.369814414	-5.479316523	-7.368598633
	C114	-2.826995598	0.500428696	-8.723685307
	C115	-2.283596787	1.829378985	-9.209445890
	C116	-3.440758930	0.527040867	-7.302083055
	C117	-2.356573390	0.504937978	-6.263623659
	C118	-1.707976526	1.690771415	-5.880004758
	C119	-1.859709474	-0.724668281	-5.799310431
	C120	-0.535355959	1.637515487	-5.121703398
	C121	-0.688293701	-0.776056345	-5.041588880
	C122	-0.001893747	0.404071969	-4.737442573
	C123	8.725103340	-5.811642815	-3.892054531
	C124	9.739016361	-5.952939375	-2.746076204
	C125	7.322081774	-6.203437695	-3.402757195
	C126	6.188618012	-5.943711696	-4.341511350
	C127	6.014850082	-6.462813733	-5.608196330
	C128	4.171592384	-5.400663206	-4.995953327
	C129	10.656105209	-7.268992533	-0.932105459
	C130	12.090992463	-6.938530795	-1.278628090
	C131	0.795005916	-7.027050906	-0.655789610
	C132	2.199531867	-6.962029493	-0.061472770
	C133	3.326088503	-6.983262334	-1.131752469
	C134	3.382608506	-8.385572272	-1.796166005
	C135	4.583042484	-8.633526381	-2.710039921
	C136	4.685773810	-10.084703349	-3.227432468
	C137	4.686161480	-6.722720176	-0.469873987
	C138	3.904646824	-0.484198629	-1.669927487
	Fe139	4.340543915	-1.728690839	-3.132915035
	Fe140	2.415587752	0.969592810	-1.322625099
	Fe141	5.244412575	0.588303619	-0.624585983
	Fe142	4.417232264	0.791363936	-3.057139608
	Fe143	4.205937810	2.902910208	-1.415046602
	Fe144	4.826098436	-1.888540701	-0.647301494
	Fe145	2.200869850	-2.399614261	-1.098857536
	H146	-6.140690122	-7.553079926	-1.200266949
	H147	-6.151842503	-6.488919195	-2.438401806
	H148	-9.704983072	-4.968716962	-0.547185504
	H149	-8.519813985	-6.162717604	-2.251030913
	H150	-8.555778717	-7.373498021	-0.974634801
	H151	-8.077212008	-2.942522551	0.556996754
	H152	-9.099571137	-5.955920239	2.111188242
	H153	-9.788882960	-3.224916778	0.936739235
	H154	-8.665557492	-6.758762318	4.247497942
	H155	-6.429831875	-6.140403289	2.220286061
	H156	-8.592989134	-5.086670661	4.848804663
	H157	-3.345682386	-2.009897597	2.610949357
	H158	-5.371998982	-3.935588296	1.987705301
	H159	-4.116799236	-6.428708310	2.237805873
	H160	-5.910003149	-0.669105476	6.332911637
	H161	-6.453452758	-2.718089873	4.377464576
	H162	-4.132976323	-6.648326834	4.007096273
	H163	-5.030981084	-1.679984006	2.158067357
	H164	-5.325959524	-4.231686521	6.457744475
	H165	-7.065208293	-2.019592028	6.505226390
	H166	-0.798245372	-3.571226406	3.279680507
	H167	-0.968461840	-2.393442974	1.967040291
	H168	0.612144880	-2.614935613	2.746477621
	H169	-1.903464246	-1.466898471	4.094933495
	H170	-4.720134777	-5.449058424	8.339444647
	H171	-2.755831586	1.643194692	5.082396604
	H172	-2.914033011	-0.947883253	8.813580723
	H173	-3.290566833	2.441368124	9.336225232
	H174	0.559322326	-0.126442288	2.880910359

	H175	-0.384497009	0.680770376	4.155545323
	H176	8.461301928	1.754682313	6.018263912
	H177	-3.813637838	2.941462687	5.661917832
	H178	-4.055447404	3.443409661	8.079370898
	H179	-2.046599230	3.119483828	5.791716786
	H180	-2.301885495	3.559338943	8.366257007
	H181	-3.765219716	1.065483732	7.313980598
	H182	-0.763140349	1.665609865	7.396383815
	H183	-2.300220885	-0.445733589	6.109648154
	H184	-1.165880792	0.227396177	2.638290872
	H185	1.449040134	4.236832325	0.823531137
	H186	0.363802584	4.389088515	2.233193659
	H187	2.159724911	5.183784290	3.125990700
	H188	2.302262877	4.315957512	5.645971391
	H189	4.301260974	4.066650741	2.735458573
	H190	6.222161542	2.507670922	2.567661371
	H191	5.553010104	1.252396981	6.641553818
	H192	-1.746467888	-3.096244276	5.917867772
	H193	-0.691495329	-6.090533034	6.016004682
	H194	-0.362595350	-5.592603530	7.689234102
	H195	-2.839778575	-5.026073924	6.134661414
	H196	-4.403069596	-3.938066607	9.231240677
	H197	3.596404826	2.772565035	6.788754595
	H198	0.888006175	-1.961360963	5.186985042
	H199	-2.662913631	-0.184296786	11.483286849
	H200	-2.727344760	-1.873263496	10.907227057
	H201	-0.045726490	-1.485941378	10.519579807
	H202	10.482153212	-7.216847692	5.264072941
	H203	-0.961929485	-5.657678239	-4.795708055
	H204	1.650845137	-6.098717093	-2.116633894
	H205	-0.743487178	-5.610699671	-9.178426971
	H206	0.754921090	-4.655513685	-9.325927728
	H207	-1.859846490	-3.867689370	-7.914235233
	H208	-1.550144246	1.744080180	-10.045497366
	H209	-1.667265276	-3.512915074	-10.414690947
	H210	-2.886684987	-2.074323979	-2.954501010
	H211	-3.777903585	-4.913060418	-2.287571011
	H212	-0.874181931	-0.396003899	-8.766797162
	H213	-3.662838680	0.291361339	-9.423580621
	H214	0.557371563	-1.692918138	-9.749726345
	H215	-0.876910412	-5.716891439	-3.043009986
	H216	-0.377205758	-2.934063149	-7.701024087
	H217	0.548325297	-2.975992084	-10.783417572
	H218	-6.130190066	-3.243292059	-4.176064730
	H219	-3.301599783	-2.453763031	-1.262624623
	H220	2.138885242	-4.694297327	-7.705271145
	H221	-2.196816128	-4.411147872	-1.713306880
	H222	-4.326493639	2.359595750	2.608788228
	H223	-3.195038350	0.436779852	0.906436298
	H224	-8.647220762	0.455872118	2.490170173
	H225	-7.339973237	-0.182236231	-0.119758734
	H226	-7.293938549	3.870043862	1.505877481
	H227	-9.125712481	8.334366924	3.672860563
	H228	-7.702651834	4.817181248	4.539613627
	H229	-9.470640469	5.117453576	2.392434619
	H230	-6.766209418	6.581880138	2.200214770
	H231	-11.041993617	9.368229390	0.039707972
	H232	-3.507512466	4.144537500	1.071163311
	H233	-4.909294171	3.917552444	0.013373263
	H234	-10.424237841	10.125010256	2.398426772
	H235	-8.682422244	5.435835138	0.846369533
	H236	-6.355512263	1.892189576	0.507651092
	H237	-7.487242512	-0.856885126	2.281971340
	H238	-9.736464054	1.893825821	-1.661326412
	H239	-1.860342104	1.693881555	-2.931883364
	H240	-10.058718608	0.153833776	-1.782659605
	H241	9.691637502	3.133531228	4.501211512
	H242	8.140730765	3.246516998	3.997487855
	H243	1.653780272	6.081074701	-0.724154272
	H244	-0.840938451	5.436911461	0.557219072
	H245	1.505682853	8.028104524	1.183221181
	H246	-3.243454669	3.646501856	-1.895334923
	H247	-4.682106815	4.352281056	-3.266110884
	H248	-6.014025555	3.674026610	-4.943065750
	H249	-4.543502279	5.525388940	-2.199649312
	H250	-4.824030288	1.542865042	-4.802882202
	H251	-6.613571666	1.536270193	-4.899105678
	H252	0.024614605	11.726449776	-0.972599713
	H253	-1.185826559	13.017933148	-0.901427760
	H254	-0.553958742	10.419385288	-2.670805021

	H255	-2.205661619	9.877733554	-2.970047316
	H256	-2.889729754	9.599624568	-5.891238384
	H257	-1.772024996	5.273556096	-5.739958196
	H258	-7.267427048	8.673910311	-1.081775429
	H259	-3.560417768	8.353098651	2.219424729
	H260	-4.291638386	7.966574130	-3.095645759
	H261	1.569359633	5.684334504	-3.255799917
	H262	2.691911591	7.451263040	-2.266943959
	H263	-0.926100371	5.129543963	-3.177306662
	H264	-8.019003104	-0.130880059	-2.829951817
	H265	4.073168697	10.335808597	-2.742582520
	H266	-5.507552769	-0.733183933	-4.071937055
	H267	1.260272087	6.769836186	-4.609774841
	H268	-3.527246133	6.936963031	0.210314515
	H269	3.487233278	10.498349111	-1.075274382
	H270	-2.051582083	8.219999943	-6.512617402
	H271	-4.545997107	9.212489463	-4.272496855
	H272	-4.490726020	-3.241905187	-4.498229078
	H273	-5.522364332	-3.277402380	-1.975954817
	H274	-7.237083134	10.190381392	0.878260011
	H275	-4.953536311	1.500277923	-2.320301290
	H276	-0.042502068	8.295916854	-3.123346699
	H277	-0.875445764	6.796700467	-5.721797391
	H278	0.030668415	4.578263777	-4.543871815
	H279	-3.069823856	6.125086366	-3.768632388
	H280	0.160582069	8.670635643	3.682590157
	H281	-0.644616666	9.736389554	0.740270117
	H282	-0.382120762	12.569922062	1.300778899
	H283	1.726924830	9.273921897	3.093770700
	H284	-2.909161149	10.479087922	2.068000540
	H285	6.074642087	-2.297450065	-8.473020618
	H286	5.927450297	-3.836469632	-7.600066790
	H287	8.730496165	-4.742188615	-4.150920872
	H288	6.749428824	-4.258944246	-9.803120194
	H289	5.197632279	-4.778712872	-9.795254542
	H290	5.497869191	4.892848106	0.346311280
	H291	5.470409567	7.430959780	1.991035999
	H292	5.186537621	8.388642067	-2.159653333
	H293	3.993139894	5.660004861	0.942355234
	H294	6.667134214	8.149641082	1.139429347
	H295	6.376682867	3.365323413	-5.611314134
	H296	6.134339143	6.954415982	-0.829361294
	H297	4.566351984	-7.344867901	5.270326348
	H298	6.696445830	-8.002335549	3.163096627
	H299	4.659399389	1.930564389	-5.999720418
	H300	4.588205091	-0.333596614	3.981576108
	H301	1.927710517	2.186377721	-8.325372756
	H302	-0.296842510	-1.738580308	-4.704357979
	H303	1.544615307	2.590034458	-6.651301271
	H304	3.547773466	-4.806221326	4.789531391
	H305	3.155371089	0.536436810	-6.128340854
	H306	3.490953316	-5.289740218	3.091033585
	H307	4.316891306	-7.528427928	3.517502071
	H308	6.005253745	-5.632030721	3.160307326
	H309	5.970891169	-5.330466732	4.904940038
	H310	-4.097480122	-0.349733603	-7.205951028
	H311	5.264936624	-9.777083983	5.100766693
	H312	4.618675686	-11.909010046	3.036375646
	H313	0.939901156	0.378918592	-4.193503254
	H314	5.552814137	-12.190967527	5.790472583
	H315	4.068986954	-12.476536886	5.158785776
	H316	5.150815711	-3.483590838	2.629206827
	H317	-0.011160848	2.551988120	-4.839203264
	H318	-2.377715767	-1.651894080	-6.054968715
	H319	8.006740362	-9.701018083	-1.121375625
	H320	7.316795215	-7.282256405	-3.169154343
	H321	6.727190696	-8.070778002	0.388419595
	H322	7.350837066	-10.250326069	-2.422239662
	H323	6.322419540	3.364817841	-8.419514882
	H324	6.431667157	-4.961736490	0.432964289
	H325	2.322043502	-7.776888007	0.662203336
	H326	4.494598880	-1.412987255	-7.113901110
	H327	3.395062809	-9.120343080	-0.976224455
	H328	4.278862109	-6.381243905	-6.860241642
	H329	3.154387376	-5.034075472	-5.035730253
	H330	6.155185932	-12.608975321	3.559865677
	H331	5.400951446	-1.213647552	2.723202780
	H332	8.232323744	-5.750014053	1.733069814
	H333	7.182798151	-3.652620336	0.791019061
	H334	8.524423626	-6.955276475	3.720941175

H335	2.907692082	-3.060086684	5.462445647
H336	4.515224005	-7.973671430	-3.588906291
H337	5.518896776	-8.382144146	-2.192356857
H338	2.448531353	-8.543717574	-2.356155440
H339	2.343876649	-1.695768309	-5.995764367
H340	-2.102262031	2.650129408	-6.222587097
H341	-4.060832486	1.433535907	-7.234065560
H342	2.255931021	-6.011877729	0.496647168
H343	8.439115237	-7.264838296	1.434656107
H344	7.400217318	-9.200805691	1.193108618
H345	3.369173702	-1.405188346	5.628978861
H346	6.671849091	-7.064140220	-6.223801833
H347	7.123503525	-5.690994358	-2.454932867
H348	1.709618457	-2.375013787	-7.525984200
H349	5.263240015	4.719096488	-5.897190877
H350	7.058388285	-0.048994189	5.120693755
H351	7.345370767	0.499406461	3.453545429
H352	10.040646283	-6.234196765	-5.407006418
H353	9.232956445	-7.553289946	-4.874958449
H354	10.635047836	-8.323442694	-0.613966324
H355	9.127163908	-7.907611274	-2.272328862
H356	12.367682807	-7.112464600	-2.346496762
H357	10.330797817	-6.655904406	-0.076630357
H358	10.070392301	0.291879301	3.830236622
H359	-3.808742302	12.815342785	4.194232377
H360	9.322569130	-7.160642908	6.613535142
H361	8.800164131	-4.661451746	5.534578716
H362	-5.116037846	12.163662086	1.528797487
H363	-4.818839015	9.984096640	3.691367751
H364	-6.347740202	10.687506955	3.129914373
H365	4.188989155	-1.300223966	0.567368773
H366	-6.246590121	6.896890285	-2.096332918
H367	0.124469041	-0.546764817	-0.680270553
H368	2.177357700	-1.178529512	0.489401039
H369	1.699202530	-1.758601315	0.700930599
H370	-1.913689411	-0.187281586	-1.220269969
Mo371	4.253243707	-4.179852383	-2.012995870
N372	-6.719670558	-7.195049247	-1.964096722
N373	-8.854606404	-4.825020751	-0.008184774
N374	-8.649721448	-5.309205546	2.755007468
N375	-5.977016531	-6.127763655	3.135552400
N376	-4.662772712	-3.655747211	2.662164524
N377	-5.743760658	-2.207354530	4.909702366
N378	-4.866452267	-3.708912614	7.208512589
N379	-2.226613861	-5.014075132	6.954902542
N380	-0.733579303	-2.995259019	5.887393657
N381	-1.479848182	-0.132762854	6.635644044
N382	-2.215737899	-0.516992173	9.424912172
N383	4.863069745	-11.835569962	5.124709289
N384	5.855561147	-9.494370426	4.311471356
N385	4.531798710	-3.513109351	3.443526116
N386	3.545878629	-2.305631705	5.192900847
N387	4.889064927	-1.234885479	3.621519313
N388	8.432842318	-7.144071167	4.743664247
N389	-5.261864722	-2.722355147	-4.040084470
N390	-1.344079680	-5.373568214	-3.886675445
N391	-5.640548650	-0.294095301	-3.144664815
N392	-8.103735267	0.691070235	-2.235697910
N393	-8.043153768	0.181368221	0.531224046
N394	-6.018485909	1.887650671	1.474705658
N395	-2.993402386	2.747176513	-1.403656302
N396	-2.365709277	0.714158708	-1.068219257
N397	-6.795666772	4.469380232	2.159070820
N398	-10.031662522	7.536943961	0.568516750
N399	-10.189457909	9.214843075	2.011028796
N400	8.930585624	2.599462179	4.074374334
N401	6.148220484	7.272486602	1.238278393
N402	4.457014597	8.692102815	-1.520042735
N403	1.921202464	8.101880093	-2.454686945
N404	-2.540230903	6.712888932	-4.436610615
N405	-2.542040088	8.657893731	-5.740823224
N406	-3.936383809	8.489314905	-3.899987715
N407	0.702140646	6.400537408	-0.479094176
N408	0.638512287	8.140163458	1.716308740
N409	-0.673453593	10.539511664	1.368275046
N410	-1.565808427	10.540613284	-2.536415264
N411	-3.217047678	11.448284932	2.082289383
N412	5.779896748	-3.936664522	-9.764725716
N413	3.742388684	-1.964226934	-7.537258236
N414	2.580232928	0.777629989	-6.954087851

N415	4.606485874	2.801827690	-6.533380024
N416	-0.033642493	-2.231439304	-10.393992941
N417	-1.865467229	-0.557823354	-8.968545463
N418	9.119996261	-6.559714113	-5.098945550
N419	5.023089683	-5.270974838	-3.977061722
N420	4.743278257	-6.105746488	-5.998858623
N421	9.773037440	-7.137551008	-2.077360352
N422	0.111195697	2.568578655	-0.387174747
N423	-0.185996991	-3.970177820	-0.415844144
N424	1.013355374	1.985768115	-0.731189850
N425	0.744354758	-3.419134265	-0.726488351
O426	-6.637906142	-5.297525268	0.205963372
O427	-7.842994033	-3.195862543	3.055111335
O428	-6.201584321	-5.696904943	5.367434747
O429	-3.144125613	-4.333497264	4.219061726
O430	-3.841192494	-0.967687490	4.841072995
O431	-4.676448622	-1.687162262	8.268536397
O432	-2.017779956	-4.632094513	9.204134317
O433	1.241032148	-4.156540958	6.129251839
O434	0.792803503	-0.198478103	6.862683382
O435	-0.745223121	1.166729291	9.817890717
O436	-0.446810742	-1.110312131	12.485926683
O437	6.838574488	-10.389396952	2.420588843
O438	7.146028803	-8.035710801	6.428370715
O439	10.767691334	-4.746786938	6.065489825
O440	-5.525475182	-0.862930796	-0.925180130
O441	-2.906602846	-4.068160622	-4.903936014
O442	-7.017422965	2.488868093	-1.343003614
O443	-5.779496018	3.271128189	-4.087857833
O444	-10.048206299	1.268325711	0.785925413
O445	-6.302947061	1.126946233	3.609031787
O446	-4.908792722	4.725760608	3.420278462
O447	-7.465101016	6.837269536	4.711053730
O448	9.962849616	-0.316484623	5.775903334
O449	2.765332630	4.388852890	4.791096293
O450	3.511527591	8.435615081	0.538505666
O451	1.350411119	10.326529911	-2.565523433
O452	-1.283457125	7.267869982	-1.207838942
O453	-1.192355642	7.007080194	2.514046387
O454	2.051643889	5.620490110	2.256381013
O455	0.211858679	11.398244874	3.301734578
O456	-2.802991598	13.515330824	1.212913872
O457	-3.216348392	11.072372570	-1.048494694
O458	-5.705287955	13.423653430	3.740705728
O459	-5.353864630	6.907002338	-1.699836058
O460	3.237372713	-3.908682147	-8.652055786
O461	1.133140319	-0.100939209	-8.500685858
O462	3.391142417	4.237929831	-7.809861343
O463	7.119175300	5.107597551	-7.715027641
O464	-3.479831852	-2.008903551	-9.664399558
O465	-0.362016403	-6.127540734	-6.627044449
O466	1.643150374	-5.228691706	-7.008882883
O467	-2.636127264	2.920583179	-8.784478327
O468	10.527720857	-5.033635285	-2.451566063
O469	12.922034773	-6.583063496	-0.454404725
O470	0.660737154	-6.447668582	-1.855606699
O471	-0.158162954	-7.527857218	-0.062434219
O472	5.844997647	-10.629527709	-3.211137022
O473	3.629202427	-10.625528328	-3.659857017
O474	5.382958548	-5.716364990	-0.937499648
O475	5.099863890	-7.465372014	0.439594904
O476	3.043490765	-5.950380057	-2.075613193
O477	8.784695228	-6.522888190	2.005321615
O478	6.906244127	-4.507910136	1.174902797
O479	-4.020901105	4.909571749	-2.772393414
O480	8.242069854	-10.129437771	-1.974104061
O481	7.607550281	-8.518393601	0.505461471
S482	3.843851514	5.154128551	-1.433457489
S483	2.780883666	2.308010402	-3.066535171
S484	2.546531275	-3.091696741	-3.252209633
S485	3.676826802	1.815741524	0.445486369
S486	0.867099573	-0.679056953	-1.815640599
S487	4.844039537	-0.395822053	-4.853606065
S488	3.630970055	-3.563570016	0.155811370
S489	6.150580228	1.946323414	-2.133291909
S490	6.139881874	-2.781915500	-2.334758631
S491	6.453827911	-0.827897345	0.534821077
end			

S2BH-2N2x-6N2x-7H2n-6Hn (20)

Fe(139) -2.478<br>Fe(140) -1.305<br>Fe(141) -2.396<br>Fe(142) 2.586<br>Fe(143) 2.824<br>Fe(144) 0.496<br>Fe(145) -0.022<br><br>**S=0**	2n2xs2bh6n2x6hn7h2ndtb.car_3<br>C1 -7.704033043 -5.495879958 -0.353864500<br>C2 -7.878272118 -6.580095705 -1.422324466<br>C3 -8.810735957 -3.696885463 0.900843491<br>C4 -8.414415577 -4.054295321 2.331107407<br>C5 -8.270768980 -5.780396693 4.058934527<br>C6 -6.752567001 -5.877651464 4.244027499<br>C7 -4.564146229 -6.071591066 3.182085182<br>C8 -4.059387357 -4.643400086 3.403685649<br>C9 -4.385692274 -2.269588464 2.880131073<br>C10 -4.627137069 -1.786068693 4.317990300<br>C11 -6.034328337 -1.794818391 6.283017208<br>C12 -5.119449149 -2.420183343 7.345615798<br>C13 -4.240125054 -4.507297056 8.287524600<br>C14 -2.720229724 -4.707269171 8.188697279<br>C15 -0.802915306 -5.222934300 6.732263418<br>C16 0.004876067 -4.034398726 6.203529352<br>C17 -0.170293934 -1.725348627 5.314697177<br>C18 -0.237062493 -0.575013172 6.332432675<br>C19 -0.767564624 -1.413611859 3.921408761<br>C20 -0.281663986 -0.066698154 3.376286437<br>C21 -0.453192476 -2.541814301 2.933326494<br>C22 -1.629575426 1.024058608 7.563622789<br>C23 -1.501058609 0.569734352 9.030533317<br>C24 -2.938631692 1.797455907 7.278320582<br>C25 -3.177505578 2.879598225 8.336439664<br>C26 -2.901658202 2.416240246 5.874099948<br>C27 -2.193529085 -0.938026830 10.797618185<br>C28 -0.781044550 -1.187425864 11.288577342<br>C29 5.408832196 -11.789221829 3.709957641<br>C30 6.124649007 -10.478838440 3.406396626<br>C31 6.279755753 -8.112467047 4.156977207<br>C32 7.345865718 -7.775172663 5.215797660<br>C33 5.054293115 -7.192033519 4.284495377<br>C34 5.410090148 -5.724039280 4.070585261<br>C35 4.175924218 -4.845701025 3.880344370<br>C36 4.329717278 -2.349568069 4.110433936<br>C37 9.535818521 -6.740769602 5.590664723<br>C38 9.690319094 -5.241821296 5.726643042<br>C39 -4.949570997 -2.540551244 -2.633873651<br>C40 -5.382627947 -1.145051798 -2.147649395<br>C41 -3.457535548 -2.763566334 -2.322145579<br>C42 -2.979714076 -4.217975085 -2.515796490<br>C43 -2.397929438 -4.530570987 -3.892427582<br>C44 -5.781045164 1.155074894 -2.943376931<br>C45 -7.020979381 1.519417551 -2.116784203<br>C46 -5.745498696 1.865265671 -4.302658990<br>C47 -9.305216259 0.907481768 -1.476165843<br>C48 -9.151141967 0.802537573 0.045157208<br>C49 -7.727358522 0.152591289 1.933076839<br>C50 -6.611479733 1.085775141 2.404342792<br>C51 -4.995826636 2.843896253 1.873342420<br>C52 -5.567221557 4.093082876 2.587591267<br>C53 -4.204349542 3.381349820 0.671120208<br>C54 -3.433421569 2.391887582 -0.137819649<br>C55 -2.984737101 1.114157927 0.085159354<br>C56 -2.305031983 1.721638677 -1.947350870<br>C57 -7.398285962 5.744388008 2.549643208<br>C58 -7.507705694 5.810705716 4.071076897<br>C59 -8.799069545 5.818508823 1.893379770<br>C60 -9.408647478 7.184290499 1.767272025<br>C61 -9.504318409 8.217984571 2.678640174<br>C62 -10.478815544 8.779125739 0.769284011<br>C63 8.576480571 1.501176571 4.946632252<br>C64 9.643517059 0.426668759 4.840412320<br>C65 7.246195595 0.853671701 4.488245477<br>C66 6.062676247 1.786267794 4.587129752<br>C67 5.298659269 1.876996558 5.765092589<br>C68 5.686500602 2.588590840 3.496241106<br>C69 4.191063806 2.728488120 5.851498118<br>C70 4.596020334 3.455142455 3.572649537<br>C71 3.843108528 3.517341977 4.748777017<br>C72 5.416437003 7.041395777 -0.022071005<br>C73 4.370538693 8.132501178 -0.304533231<br>C74 4.749759774 5.667383411 0.068465687<br>C75 3.587881400 9.833800624 -1.916621062<br>C76 2.167392659 9.458432665 -2.343886541<br>C77 0.598467891 7.556442019 -2.708656254

	C78	-0.077780363	7.076948762	-1.399178489
	C79	0.802344944	6.388975485	-3.705037754
	C80	-0.396065165	5.509338142	-4.081417047
	C81	-1.401157321	6.095252054	-5.085762009
	C82	-3.035389755	7.944281974	-4.723380394
	C83	0.111454973	5.948790982	0.762103880
	C84	-0.235518036	7.088407759	1.732589517
	C85	1.015033933	4.951360155	1.516600166
	C86	0.657281389	9.097042077	2.810899170
	C87	0.037854632	10.458739311	2.530329332
	C88	-1.103436259	11.867383089	0.895374737
	C89	-2.474220600	12.363144272	1.412081769
	C90	-1.008163289	11.996062845	-0.627698724
	C91	-2.040987144	11.177665178	-1.394397976
	C92	-4.625932564	11.751118005	2.416729501
	C93	-4.726030403	12.763348725	3.548063022
	C94	-5.341432687	10.431142103	2.823857011
	C95	-5.386499252	9.436071156	1.685668873
	C96	-6.424932572	9.493334279	0.742202400
	C97	-4.376008761	8.479172194	1.498495264
	C98	-6.440947798	8.648808338	-0.368726396
	C99	-4.362902393	7.640267525	0.379298319
	C100	-5.397786192	7.738884308	-0.545714705
	C101	5.551695886	-3.279712152	-8.469411507
	C102	4.063645222	-3.079642847	-8.245769851
	C103	2.379931377	-1.648315333	-7.106767722
	C104	1.963843181	-0.250778788	-7.578269315
	C105	2.325579457	2.158931281	-7.278049269
	C106	3.510081192	3.123090878	-7.260331582
	C107	5.677964668	3.781133788	-6.378420845
	C108	6.398709865	4.131748620	-7.663325543
	C109	-1.180817085	-2.813147773	-9.705958410
	C110	-2.268106216	-1.762482628	-9.456382416
	C111	-0.906955019	-3.598160871	-8.403071593
	C112	-0.102973561	-4.882501125	-8.678451170
	C113	0.366619582	-5.496338607	-7.377860315
	C114	-2.817515493	0.506717291	-8.735595732
	C115	-2.280179699	1.835186800	-9.230235837
	C116	-3.400091544	0.535407287	-7.300629879
	C117	-2.315197187	0.498916322	-6.258164598
	C118	-1.654811674	1.674441616	-5.861222486
	C119	-1.854980410	-0.734397859	-5.765729171
	C120	-0.524610888	1.607858848	-5.040298859
	C121	-0.728866842	-0.799284630	-4.940961893
	C122	-0.044410184	0.371177464	-4.597053406
	C123	8.693150729	-5.800743486	-3.877942933
	C124	9.713306810	-5.934201220	-2.736539432
	C125	7.285450629	-6.145319756	-3.361223683
	C126	6.153251359	-5.898560258	-4.305680444
	C127	5.997959394	-6.424933931	-5.571639193
	C128	4.150261600	-5.349689101	-4.999900614
	C129	10.645507897	-7.246634974	-0.928361452
	C130	12.078961011	-6.908973875	-1.273745086
	C131	0.771987128	-7.212287268	-0.697658949
	C132	2.164144779	-7.080592191	-0.095942973
	C133	3.310262319	-7.087835122	-1.141393447
	C134	3.400388179	-8.482016178	-1.812888842
	C135	4.615777941	-8.693363979	-2.715619107
	C136	4.744351321	-10.134310786	-3.255737334
	C137	4.656061321	-6.805845350	-0.455499372
	C138	3.915419459	-0.441950194	-1.678144560
	Fe139	4.264366897	-1.781784519	-3.062834123
	Fe140	2.448189494	0.939767484	-1.325724532
	Fe141	5.230280381	0.607565922	-0.674665796
	Fe142	4.488220107	0.739221009	-3.139956306
	Fe143	4.230823146	2.917472439	-1.509537130
	Fe144	4.586987804	-1.856802466	-0.479139388
	Fe145	2.075142467	-2.703312769	-0.993486361
	H146	-6.089644750	-7.528332356	-1.166898793
	H147	-6.093652015	-6.473260548	-2.412757290
	H148	-9.687276630	-4.980860945	-0.563845717
	H149	-8.467163440	-6.165261892	-2.256426091
	H150	-8.508050802	-7.367076009	-0.972424443
	H151	-8.096347235	-2.932314760	0.562658190
	H152	-9.128416820	-5.957938262	2.094280428
	H153	-9.808359266	-3.238686519	0.921516844
	H154	-8.713191057	-6.770513939	4.231333437
	H155	-6.468681371	-6.139817645	2.218484750
	H156	-8.637465816	-5.100968203	4.839053161
	H157	-3.335208050	-2.059227873	2.642781561

	H158	-5.401127179	-3.943857771	2.006683360
	H159	-4.161435696	-6.463002104	2.238089668
	H160	-5.920931888	-0.705447119	6.360272947
	H161	-6.461135589	-2.732147970	4.386554711
	H162	-4.180406924	-6.683756313	4.007075228
	H163	-5.014026771	-1.693214631	2.187297327
	H164	-5.350388623	-4.273404427	6.473018067
	H165	-7.074196105	-2.059096285	6.520175341
	H166	-0.812101487	-3.515595767	3.292177961
	H167	-0.935310863	-2.350884257	1.961742376
	H168	0.629966398	-2.612041855	2.746802349
	H169	-1.857915030	-1.349489823	4.050827289
	H170	-4.704989295	-5.503356496	8.329697558
	H171	-2.778682930	1.658251314	5.088637878
	H172	-2.918934563	-0.942395250	8.790825715
	H173	-3.308751688	2.456283239	9.342923518
	H174	0.781444476	-0.100394654	3.090514193
	H175	-0.417904125	0.754439829	4.094991979
	H176	8.473598534	1.764668178	6.017281209
	H177	-3.838771926	2.953486054	5.670529828
	H178	-4.082085052	3.452707741	8.086665295
	H179	-2.071899673	3.137607760	5.792310960
	H180	-2.329166384	3.581403108	8.371737110
	H181	-3.774227895	1.075541204	7.322195431
	H182	-0.777508298	1.703991574	7.413891970
	H183	-2.292027028	-0.424265138	6.115000154
	H184	-0.858746068	0.195154519	2.475691272
	H185	1.443495375	4.240151263	0.794982365
	H186	0.367585318	4.395017315	2.211023507
	H187	2.171175830	5.182424710	3.093403426
	H188	2.296896278	4.304859350	5.618395684
	H189	4.305374210	4.060612363	2.713561457
	H190	6.237789076	2.514079994	2.556547737
	H191	5.563819701	1.263245726	6.630539702
	H192	-1.739770081	-3.028088526	5.947586356
	H193	-0.677573031	-6.049059877	6.016737597
	H194	-0.350960678	-5.531330654	7.684428504
	H195	-2.830698112	-4.985904166	6.126817315
	H196	-4.418101969	-3.999013173	9.242619111
	H197	3.594741984	2.768565579	6.767058932
	H198	0.902005247	-1.917939181	5.193929850
	H199	-2.682253410	-0.214409466	11.470707692
	H200	-2.733774783	-1.895343175	10.869020881
	H201	-0.054786110	-1.490211383	10.497831065
	H202	10.489895892	-7.174521103	5.252141424
	H203	-0.974651886	-5.675905605	-4.823954251
	H204	1.556596740	-6.297639473	-2.193023822
	H205	-0.747811031	-5.613600132	-9.187540160
	H206	0.755168531	-4.665165348	-9.330430833
	H207	-1.856607606	-3.870725002	-7.917465661
	H208	-1.559592608	1.749954161	-10.077604367
	H209	-1.653244980	-3.508900260	-10.415590932
	H210	-2.857952839	-2.085317967	-2.951636197
	H211	-3.820585260	-4.915436199	-2.357573767
	H212	-0.862347246	-0.384337422	-8.791395139
	H213	-3.663739283	0.292343810	-9.420056747
	H214	0.572989430	-1.693594491	-9.742568446
	H215	-0.940683480	-5.781386436	-3.073152816
	H216	-0.370576551	-2.946760942	-7.695038538
	H217	0.565072972	-2.978119864	-10.774329060
	H218	-6.101722529	-3.218493051	-4.210754350
	H219	-3.291815547	-2.480739780	-1.271543433
	H220	2.135937825	-4.714331729	-7.712712038
	H221	-2.241549834	-4.463205861	-1.742627282
	H222	-4.332194498	2.358421398	2.602739610
	H223	-3.118079716	0.451697485	0.930838486
	H224	-8.636837215	0.445863144	2.475554554
	H225	-7.329231685	-0.178883888	-0.137379350
	H226	-7.303134674	3.870411068	1.516655511
	H227	-9.139043144	8.334585233	3.692768326
	H228	-7.689917119	4.819940993	4.553094093
	H229	-9.478629390	5.125844916	2.420078487
	H230	-6.773558172	6.584562767	2.206588177
	H231	-11.016357364	9.385163803	0.043895134
	H232	-3.504948153	4.138529013	1.061934729
	H233	-4.901521875	3.906276052	-0.001065822
	H234	-10.416285180	10.134402470	2.409997351
	H235	-8.699078157	5.432246766	0.867148878
	H236	-6.362867690	1.900910917	0.502028765
	H237	-7.477992911	-0.867860108	2.259087668

H238	-9.722349373	1.905649653	-1.670632862
H239	-1.827769472	1.699765091	-2.924607639
H240	-10.047930030	0.166710557	-1.800062007
H241	9.697889538	3.158716877	4.510445197
H242	8.147000553	3.267057145	4.006512537
H243	1.654877170	6.111499766	-0.739865657
H244	-0.837897086	5.444181302	0.532727450
H245	1.484225016	8.050516139	1.171950964
H246	-3.255525146	3.628704348	-1.903666473
H247	-4.690519103	4.371697814	-3.274990936
H248	-6.011200929	3.692555474	-4.961720547
H249	-4.548927069	5.536170967	-2.199460396
H250	-4.816182351	1.570165424	-4.817693514
H251	-6.605586920	1.550141589	-4.913786553
H252	0.015465769	11.759569729	-0.950037561
H253	-1.192831042	13.052636192	-0.877731077
H254	-0.560900424	10.464971779	-2.662260085
H255	-2.213856756	9.931095081	-2.971186512
H256	-2.923847675	9.607628425	-5.914069494
H257	-1.780027048	5.297009186	-5.741922679
H258	-7.264420622	8.694624324	-1.085837928
H259	-3.572243425	8.364960157	2.231010932
H260	-4.293826632	7.993843002	-3.092635319
H261	1.566877576	5.724779940	-3.269292777
H262	2.686183636	7.488705074	-2.279142288
H263	-0.926485913	5.159481278	-3.181653088
H264	-8.005043471	-0.117788420	-2.844716832
H265	4.059054227	10.379618627	-2.744313240
H266	-5.491479017	-0.710286179	-4.090221660
H267	1.247650678	6.811984693	-4.620522787
H268	-3.534216786	6.948207204	0.221571527
H269	3.475060568	10.534313365	-1.075874812
H270	-2.096736950	8.222465938	-6.535627091
H271	-4.566706802	9.226580823	-4.279110716
H272	-4.462003262	-3.201210230	-4.527821839
H273	-5.521532882	-3.252025729	-2.009291460
H274	-7.240168519	10.208685029	0.875836638
H275	-4.943003607	1.523495323	-2.336053197
H276	-0.053245545	8.331108780	-3.123773728
H277	-0.891343158	6.825573585	-5.727163616
H278	0.028314786	4.610757873	-4.550302525
H279	-3.077893203	6.146697269	-3.767735435
H280	0.135528884	8.667108939	3.678352202
H281	-0.663181308	9.762814205	0.742312551
H282	-0.394012726	12.590791305	1.326291502
H283	1.703034802	9.275499571	3.098291488
H284	-2.929401896	10.497678171	2.062228851
H285	6.068024053	-2.308338184	-8.455876404
H286	5.911248981	-3.849125575	-7.586977203
H287	8.718786793	-4.739456767	-4.166218360
H288	6.758750893	-4.265373237	-9.782437584
H289	5.208099542	-4.788666405	-9.791429192
H290	5.531830845	4.932051048	0.295521152
H291	5.453642047	7.450195306	1.979370264
H292	5.180937552	8.435912995	-2.170328998
H293	4.008140581	5.667770365	0.879672775
H294	6.660155794	8.180302536	1.151770660
H295	6.422732830	3.352614778	-5.689530182
H296	6.154004062	7.023248409	-0.839158815
H297	4.588774712	-7.344108188	5.272642132
H298	6.706313116	-7.999941229	3.151959912
H299	4.670712287	1.939269141	-6.002948261
H300	4.579572866	-0.320172005	4.028777161
H301	1.899010121	2.177381290	-8.287917415
H302	-0.372228391	-1.765941544	-4.577988312
H303	1.567581865	2.594877548	-6.604969244
H304	3.579408562	-4.802298692	4.798857335
H305	3.115859203	0.527263816	-6.087865690
H306	3.528942545	-5.261389773	3.090672691
H307	4.330334885	-7.517200174	3.519985088
H308	6.036237133	-5.629162440	3.171220643
H309	5.999026425	-5.336026168	4.916858461
H310	-4.065088743	-0.333980451	-7.196035553
H311	5.259651375	-9.780300754	5.070362367
H312	4.593161939	-11.865753072	2.975012389
H313	0.853370466	0.334338943	-3.980651691
H314	5.526429710	-12.212855806	5.722527051
H315	4.040751936	-12.477096471	5.086990327
H316	5.213092962	-3.464280989	2.670224102
H317	0.004348863	2.516787061	-4.746840872

H318	-2.371864876	-1.657153457	-6.038873788
H319	8.018513491	-9.656086216	-1.127409515
H320	7.264650171	-7.212729634	-3.082243669
H321	6.713929343	-8.058766762	0.394870970
H322	7.388001864	-10.233226682	-2.432218743
H323	6.287461332	3.391014957	-8.491043505
H324	6.371591479	-4.977168737	0.445237938
H325	2.302663889	-7.872551319	0.647735583
H326	4.480738729	-1.429075232	-7.120511717
H327	3.422336128	-9.219482682	-0.995311830
H328	4.285413172	-6.344509790	-6.854295655
H329	3.138185813	-4.972225843	-5.060725125
H330	6.117465884	-12.604361469	3.482903903
H331	5.433928796	-1.180143242	2.784251151
H332	8.220480532	-5.722910843	1.731355717
H333	7.119984701	-3.657026349	0.761090724
H334	8.523594939	-6.934447907	3.710437301
H335	2.874361171	-3.058186253	5.437843234
H336	4.547218923	-8.018937287	-3.583873469
H337	5.540566021	-8.436673591	-2.181287318
H338	2.476775597	-8.658405898	-2.384363889
H339	2.328270177	-1.718786581	-6.008120822
H340	-2.009588221	2.638079841	-6.234582152
H341	-4.011368974	1.446828591	-7.220526605
H342	2.176765458	-6.119252500	0.445636475
H343	8.436513994	-7.233258581	1.428232204
H344	7.425672655	-9.173724116	1.180460600
H345	3.343078060	-1.407460299	5.633670902
H346	6.666450862	-7.027757992	-6.173498883
H347	7.105375020	-5.592974651	-2.431976295
H348	1.693398008	-2.374298595	-7.547241944
H349	5.293614432	4.705616208	-5.919193214
H350	7.076253650	-0.036144628	5.111443561
H351	7.366628368	0.515793620	3.446237182
H352	9.984471467	-6.287386807	-5.392758230
H353	9.157561819	-7.577776163	-4.821833764
H354	10.629409795	-8.300863430	-0.608992285
H355	9.128171079	-7.901939569	-2.273571062
H356	12.356727073	-7.079076823	-2.341991000
H357	10.316320127	-6.634379240	-0.073759532
H358	10.088900238	0.319917486	3.821780475
H359	-3.829162890	12.823642582	4.209005423
H360	9.330065224	-7.138070659	6.600642532
H361	8.772629358	-4.645144646	5.499480683
H362	-5.136175864	12.181066428	1.539235625
H363	-4.829309153	9.998384275	3.697160114
H364	-6.361503358	10.695706494	3.136880712
H365	3.976964843	-1.133033087	0.926359739
H366	-6.243674027	6.915236322	-2.095867124
H367	0.325653917	-0.537419915	-0.196030863
H368	3.292398916	-1.238773764	0.488005513
H369	1.783273863	-2.192765088	0.392657305
H370	-1.771159668	-0.141759347	-1.179858637
Mo371	4.087952815	-4.262996154	-1.969934025
N372	-6.661992953	-7.179994985	-1.940101171
N373	-8.842844887	-4.822106793	-0.019765350
N374	-8.681070359	-5.315493477	2.744155703
N375	-6.017840337	-6.143748436	3.134663396
N376	-4.682138171	-3.679877742	2.677655968
N377	-5.748348814	-2.235216419	4.927318787
N378	-4.884552510	-3.752728987	7.221330983
N379	-2.217698996	-4.970959656	6.947658551
N380	-0.727291202	-2.942681374	5.881796491
N381	-1.472922247	-0.093280395	6.631585966
N382	-2.225135042	-0.514658880	9.409238991
N383	4.838757397	-11.840620101	5.063873779
N384	5.860923975	-9.496201944	4.289757624
N385	4.584324800	-3.498648344	3.477524004
N386	3.525869711	-2.305181767	5.194213874
N387	4.909209993	-1.214682265	3.676721781
N388	8.439345480	-7.134437530	4.731613621
N389	-5.238006979	-2.692560323	-4.066848261
N390	-1.371583457	-5.401550993	-3.918035494
N391	-5.622945328	-0.271925226	-3.162300193
N392	-8.091349946	0.703341588	-2.249634469
N393	-8.033073205	0.181129131	0.514896386
N394	-6.023466868	1.893172330	1.468086506
N395	-2.996773399	2.735055284	-1.410713665
N396	-2.298009758	0.727324069	-1.050435749
N397	-6.799597889	4.472110065	2.163701034

	N398	-10.021607328	7.547246607	0.579139810
	N399	-10.183349641	9.223515752	2.023211495
	N400	8.940119833	2.623628855	4.079219328
	N401	6.140752894	7.301475063	1.233080415
	N402	4.450912435	8.733579908	-1.528607188
	N403	1.912955260	8.138442141	-2.460478429
	N404	-2.551885835	6.735218791	-4.438238205
	N405	-2.569405701	8.669387752	-5.757901092
	N406	-3.948099147	8.512771084	-3.903586462
	N407	0.702860038	6.422340918	-0.491889593
	N408	0.618746636	8.150821435	1.709949028
	N409	-0.692474790	10.559553227	1.378451386
	N410	-1.572789246	10.585356689	-2.526126143
	N411	-3.234512987	11.467582096	2.090019356
	N412	5.788127265	-3.945289678	-9.753607459
	N413	3.728410829	-1.972065832	-7.553711379
	N414	2.575484071	0.770295259	-6.931877915
	N415	4.626502663	2.792464510	-6.562564632
	N416	-0.017082626	-2.232769144	-10.386899767
	N417	-1.853896577	-0.548981289	-8.988836695
	N418	9.060753629	-6.587925028	-5.068835912
	N419	4.982430450	-5.218022614	-3.964376642
	N420	4.735748094	-6.064524810	-5.986838800
	N421	9.761754260	-7.121636493	-2.073750230
	N422	0.143507480	2.627851060	-0.509944446
	N423	-0.331489007	-4.233335311	-0.419953981
	N424	1.037520424	2.016935202	-0.832796914
	N425	0.619862163	-3.703657558	-0.717461534
	O426	-6.622823645	-5.266751379	0.217311903
	O427	-7.873489913	-3.204823831	3.061025056
	O428	-6.250693944	-5.731577484	5.370654569
	O429	-3.151749237	-4.384067586	4.209362725
	O430	-3.825601602	-1.025950586	4.884134975
	O431	-4.675589378	-1.735073462	8.283422023
	O432	-2.027780185	-4.679398072	9.213525505
	O433	1.240472429	-4.124037520	6.078143583
	O434	0.801677680	-0.137612720	6.852165416
	O435	-0.768126948	1.173858901	9.832008704
	O436	-0.461841139	-1.135113475	12.466772365
	O437	6.847893183	-10.378239774	2.395032567
	O438	7.175847980	-8.066007152	6.412736156
	O439	10.726864843	-4.700659268	6.078863524
	O440	-5.500007997	-0.844468586	-0.944030582
	O441	-2.868225554	-4.023061529	-4.936451603
	O442	-7.009613542	2.502197581	-1.354386164
	O443	-5.784746937	3.291772458	-4.103372097
	O444	-10.037478302	1.268371227	0.773536769
	O445	-6.275929515	1.091554827	3.592254067
	O446	-4.898545007	4.735740393	3.402215069
	O447	-7.456362020	6.840646148	4.720322890
	O448	9.986025876	-0.295870734	5.764954824
	O449	2.755703059	4.369890534	4.760560514
	O450	3.509714679	8.460827234	0.530304022
	O451	1.338265573	10.362934823	-2.562813600
	O452	-1.286161182	7.288542345	-1.210975229
	O453	-1.191818934	6.986429062	2.511770543
	O454	2.059011465	5.621964439	2.225818433
	O455	0.191820025	11.397953812	3.320567825
	O456	-2.812053921	13.544037160	1.246748578
	O457	-3.226136322	11.110102751	-1.038949999
	O458	-5.723453284	13.437097647	3.752598343
	O459	-5.352185784	6.923856398	-1.696530673
	O460	3.233056734	-3.925624350	-8.658326803
	O461	1.147589183	-0.088613290	-8.507634337
	O462	3.400196870	4.213522853	-7.843974601
	O463	7.089659310	5.130910461	-7.784880040
	O464	-3.468411217	-2.016976149	-9.649131817
	O465	-0.366869470	-6.146375632	-6.639526545
	O466	1.641143289	-5.251889579	-7.018225279
	O467	-2.623495311	2.926259077	-8.797439312
	O468	10.490571026	-5.006297405	-2.438508225
	O469	12.907960637	-6.549885143	-0.449102763
	O470	0.622325910	-6.682328847	-1.925184807
	O471	-0.174341070	-7.717627609	-0.102895606
	O472	5.910512989	-10.663895292	-3.233620646
	O473	3.699549729	-10.681653017	-3.707248184
	O474	5.328408772	-5.783757043	-0.903874808
	O475	5.065711367	-7.560224740	0.449164937
	O476	3.025857730	-6.053297545	-2.089687402
	O477	8.780988107	-6.490682710	1.999786484

O478	6.860377374	-4.506741122	1.167483252
O479	-4.028672454	4.924990141	-2.778078716
O480	8.271897790	-10.078734133	-1.977965614
O481	7.611058195	-8.478926994	0.499321498
S482	3.897821259	5.185405237	-1.506590258
S483	2.792617249	2.234347767	-3.157096315
S484	2.444978633	-3.123419955	-3.239423037
S485	3.696737711	1.890309176	0.385888120
S486	0.870769718	-0.792266937	-1.416048617
S487	4.856474131	-0.586387713	-4.833392302
S488	3.556186710	-3.742722299	0.236376972
S489	6.185197343	1.909677063	-2.177847847
S490	5.969789060	-2.809091088	-2.029512981
S491	6.297900849	-0.830104640	0.609670070
end			

TS for 7H2n dissociation (20) to (20d)

Fe(139)	-2.492
Fe(140)	-1.258
Fe(141)	-2.399
Fe(142)	2.616
Fe(143)	2.785
Fe(144)	0.457
Fe(145)	-0.015

S=0

2n2xs2bh6n2x6hn7h2ndtc_1_53354.56

C1	-7.703118237	-5.495726977	-0.360076786
C2	-7.895687579	-6.578043577	-1.427729060
C3	-8.794372271	-3.692672927	0.904025145
C4	-8.396060037	-4.048954821	2.333538139
C5	-8.241918418	-5.773065344	4.062202047
C6	-6.723320718	-5.864935267	4.244548277
C7	-4.535791870	-6.050275608	3.181467238
C8	-4.046317092	-4.616743148	3.403580897
C9	-4.391118147	-2.248085163	2.861106786
C10	-4.633275658	-1.758211451	4.296931100
C11	-6.029010014	-1.769427510	6.268905655
C12	-5.113192103	-2.389993061	7.333706773
C13	-4.234493928	-4.475187344	8.283048810
C14	-2.716268407	-4.691495338	8.184690109
C15	-0.805479066	-5.247719422	6.734254640
C16	0.006867155	-4.058084350	6.211574345
C17	-0.177257545	-1.756787362	5.299159877
C18	-0.239319359	-0.608068684	6.319843336
C19	-0.803176967	-1.453269637	3.915183661
C20	-0.380588116	-0.085966150	3.361849528
C21	-0.472548753	-2.566592223	2.914335612
C22	-1.617712831	1.003990439	7.548195951
C23	-1.482908190	0.557621288	9.016795990
C24	-2.924423678	1.783296036	7.267375940
C25	-3.158375950	2.863926459	8.328131166
C26	-2.886537762	2.406076903	5.864912776
C27	-2.178187360	-0.930687443	10.799034990
C28	-0.766718524	-1.179432180	11.293253424
C29	5.411437842	-11.785076916	3.732133239
C30	6.121798144	-10.473877412	3.419829755
C31	6.271124345	-8.104560598	4.161159800
C32	7.334945068	-7.760511445	5.220023400
C33	5.041098095	-7.190331722	4.283290840
C34	5.391023585	-5.722267491	4.063714155
C35	4.153806358	-4.849598661	3.868806659
C36	4.329137475	-2.357241279	4.106742470
C37	9.534958120	-6.747903371	5.591574335
C38	9.706142509	-5.251059011	5.730386048
C39	-4.947844324	-2.546386616	-2.615035598
C40	-5.387080439	-1.151952125	-2.131872391
C41	-3.451658181	-2.756548809	-2.313912888
C42	-2.965254040	-4.209429700	-2.500167806
C43	-2.398643690	-4.531820433	-3.880766433
C44	-5.788142040	1.145568811	-2.932586935
C45	-7.028677381	1.511124606	-2.107430227
C46	-5.750688873	1.853938423	-4.292927330
C47	-9.313055790	0.899313236	-1.466935961
C48	-9.159168842	0.799464589	0.054602575
C49	-7.735621892	0.158348487	1.945217271
C50	-6.621901052	1.095663227	2.413866113
C51	-4.993229108	2.838771385	1.875939960
C52	-5.566174218	4.086805081	2.589944963
C53	-4.205331246	3.379385947	0.672532465
C54	-3.438770372	2.390227753	-0.140338421
C55	-3.009084882	1.105189122	0.075424019
C56	-2.313971406	1.716648260	-1.951029821
C57	-7.392423069	5.742084090	2.539571830
C58	-7.508613623	5.807908355	4.060562764
C59	-8.790775869	5.815555169	1.878324048
C60	-9.405181468	7.179502482	1.755284246
C61	-9.499799341	8.213520484	2.666311950

	C62	-10.488319511	8.768766134	0.762441684
	C63	8.568134552	1.491144858	4.951999016
	C64	9.630830631	0.412097478	4.848557135
	C65	7.235642365	0.845778671	4.496545392
	C66	6.053795211	1.780550616	4.596250603
	C67	5.291154572	1.872890928	5.775177212
	C68	5.679230169	2.585418660	3.506601705
	C69	4.187933749	2.729682809	5.864333901
	C70	4.593110914	3.457085792	3.586075175
	C71	3.842699892	3.522478691	4.763600786
	C72	5.412709694	7.031007711	-0.009032552
	C73	4.369413407	8.123752795	-0.296569984
	C74	4.740108860	5.661873851	0.089986829
	C75	3.591105267	9.818750603	-1.917151228
	C76	2.169581523	9.445678900	-2.343092431
	C77	0.596712037	7.546585002	-2.704754337
	C78	-0.081283117	7.069995883	-1.395007174
	C79	0.800227636	6.376999464	-3.698893262
	C80	-0.398508740	5.498603275	-4.076354927
	C81	-1.401217397	6.084115045	-5.083474466
	C82	-3.031432383	7.937870736	-4.720945065
	C83	0.107351699	5.948251746	0.770128193
	C84	-0.233739301	7.092633899	1.736994220
	C85	1.008937241	4.949493674	1.526132590
	C86	0.668459839	9.100651651	2.808339263
	C87	0.048093254	10.460939652	2.522964580
	C88	-1.099898999	11.860113463	0.884099632
	C89	-2.472006454	12.354299038	1.398910516
	C90	-1.006363632	11.983361518	-0.639507191
	C91	-2.037974059	11.158408504	-1.400386238
	C92	-4.619971970	11.743986288	2.411645446
	C93	-4.720444005	12.757355223	3.541965113
	C94	-5.335466655	10.424308704	2.819562010
	C95	-5.382365305	9.428654487	1.681835653
	C96	-6.423806303	9.483472229	0.741481072
	C97	-4.371164336	8.472949282	1.492586799
	C98	-6.441879726	8.637930064	-0.368746817
	C99	-4.359882069	7.633416784	0.373880908
	C100	-5.397593947	7.729726147	-0.548140038
	C101	5.552854965	-3.275507086	-8.479820659
	C102	4.066188058	-3.074956081	-8.248719304
	C103	2.388058520	-1.644588979	-7.101605205
	C104	1.966877908	-0.250036912	-7.577491420
	C105	2.327244454	2.161983048	-7.288804265
	C106	3.504330080	3.134742405	-7.257813153
	C107	5.665849137	3.800372960	-6.364304424
	C108	6.417885310	4.129725678	-7.637158750
	C109	-1.187609446	-2.813013054	-9.705443740
	C110	-2.275349206	-1.762986533	-9.456495456
	C111	-0.910115982	-3.594425927	-8.401395800
	C112	-0.103680476	-4.877732899	-8.674967618
	C113	0.368136208	-5.487969524	-7.373437765
	C114	-2.823972733	0.503997130	-8.728502942
	C115	-2.284474792	1.833510166	-9.217799085
	C116	-3.415360566	0.528647722	-7.297458296
	C117	-2.329496640	0.496910930	-6.257366071
	C118	-1.674579441	1.676148160	-5.863075819
	C119	-1.854875679	-0.735356352	-5.775995704
	C120	-0.531438335	1.614446517	-5.060330892
	C121	-0.715088136	-0.795327618	-4.970541937
	C122	-0.033468968	0.379078249	-4.633862195
	C123	8.705972567	-5.799468881	-3.882120749
	C124	9.723720565	-5.939993842	-2.739767113
	C125	7.299483732	-6.168660926	-3.379944581
	C126	6.169307061	-5.914579376	-4.324427862
	C127	6.009059515	-6.447584259	-5.586872659
	C128	4.169348981	-5.358141577	-5.017962975
	C129	10.646949118	-7.257883636	-0.930503806
	C130	12.081857066	-6.926523361	-1.275720197
	C131	0.775863341	-7.127040569	-0.677101817
	C132	2.177381326	-7.025372967	-0.087471329
	C133	3.314024793	-7.057822183	-1.143347453
	C134	3.385361442	-8.456558019	-1.804961066
	C135	4.591247061	-8.677864568	-2.717785413
	C136	4.710944574	-10.121580591	-3.251315486
	C137	4.665311433	-6.781681213	-0.465148440
	C138	3.920179601	-0.450163352	-1.682512073
	Fe139	4.280921545	-1.773263538	-3.085602815
	Fe140	2.461310638	0.935943834	-1.307144874
	Fe141	5.241064080	0.617988540	-0.676669628

	Fe142	4.486276807	0.754488385	-3.142557993
	Fe143	4.230316659	2.920754887	-1.495444034
	Fe144	4.632746793	-1.841331531	-0.504385521
	Fe145	2.107041130	-2.675427976	-1.070212505
	H146	-6.113861533	-7.544527558	-1.193197169
	H147	-6.120316740	-6.484421141	-2.435286054
	H148	-9.685929393	-4.973944995	-0.552224912
	H149	-8.489764541	-6.156730986	-2.254912832
	H150	-8.527821590	-7.360262011	-0.972339769
	H151	-8.076473409	-2.932748941	0.562415736
	H152	-9.100574592	-5.956166399	2.098573905
	H153	-9.788996735	-3.227735626	0.927503717
	H154	-8.681555892	-6.763984618	4.237219322
	H155	-6.441268516	-6.143319564	2.220544539
	H156	-8.608749153	-5.093168198	4.841921027
	H157	-3.344174196	-2.025006166	2.619493150
	H158	-5.375868477	-3.939619470	1.986113680
	H159	-4.130349258	-6.436601517	2.236526719
	H160	-5.914176477	-0.679799752	6.340484837
	H161	-6.457603670	-2.722041898	4.379958027
	H162	-4.145267805	-6.659225798	4.005723507
	H163	-5.027311583	-1.683188649	2.166172020
	H164	-5.333319682	-4.244057284	6.461613720
	H165	-7.068574882	-2.031518273	6.509759099
	H166	-0.802444755	-3.551683012	3.270968572
	H167	-0.970659317	-2.381075794	1.949933872
	H168	0.610791899	-2.605995932	2.716120296
	H169	-1.891628276	-1.426708894	4.069594282
	H170	-4.710844523	-5.465576639	8.334393262
	H171	-2.764211232	1.650220952	5.077303262
	H172	-2.907427464	-0.949306507	8.793132416
	H173	-3.291940244	2.438955598	9.333555827
	H174	0.630874471	-0.115843012	2.927938172
	H175	-0.402138528	0.707385571	4.122627933
	H176	8.466706705	1.759289623	6.021206256
	H177	-3.823232121	2.944689645	5.662788883
	H178	-4.060392768	3.441504785	8.079416006
	H179	-2.056083337	3.127121106	5.785956140
	H180	-2.307041422	3.561949651	8.365419307
	H181	-3.762841784	1.064813395	7.310330873
	H182	-0.762980245	1.678777585	7.390748205
	H183	-2.292927708	-0.436834658	6.097995795
	H184	-1.073472701	0.220641168	2.562303517
	H185	1.437593571	4.238197486	0.805021980
	H186	0.360172745	4.394445337	2.219879646
	H187	2.162856033	5.181414152	3.105686175
	H188	2.296388090	4.308741215	5.632647226
	H189	4.303507293	4.064283746	2.727940787
	H190	6.228467699	2.509688758	2.565710542
	H191	5.555235959	1.257620066	6.639960581
	H192	-1.740693708	-3.070944192	5.911211661
	H193	-0.683768109	-6.070796276	6.014734653
	H194	-0.354671675	-5.562056368	7.684785394
	H195	-2.833787669	-5.005467023	6.129145194
	H196	-4.406271794	-3.956501741	9.233591071
	H197	3.593808089	2.771936616	6.781037582
	H198	0.894714556	-1.944157470	5.166181114
	H199	-2.664480297	-0.198167303	11.463745256
	H200	-2.721604895	-1.885205677	10.880647264
	H201	-0.040048840	-1.491097802	10.506666675
	H202	10.483488355	-7.191200487	5.250531876
	H203	-0.968718604	-5.664556261	-4.815978140
	H204	1.566536647	-6.247028492	-2.186655370
	H205	-0.747393988	-5.611270105	-9.181978329
	H206	0.753505854	-4.659835840	-9.328009616
	H207	-1.858620929	-3.868186552	-7.913800909
	H208	-1.557646204	1.749491867	-10.059862376
	H209	-1.660190385	-3.510998473	-10.413433822
	H210	-2.861088282	-2.077978821	-2.951692735
	H211	-3.799946450	-4.910367479	-2.324964459
	H212	-0.870096644	-0.389340211	-8.781080095
	H213	-3.667389858	0.293204410	-9.418266968
	H214	0.565143374	-1.692926784	-9.744666187
	H215	-0.909352272	-5.745412466	-3.063908147
	H216	-0.373560162	-2.939887691	-7.696419083
	H217	0.556078418	-2.976726567	-10.777272850
	H218	-6.113642797	-3.228146740	-4.178735256
	H219	-3.281831262	-2.467630879	-1.265419843
	H220	2.136868397	-4.705086172	-7.710939496
	H221	-2.216268109	-4.441429637	-1.733532623

	H222	-4.327533602	2.356119174	2.605355145
	H223	-3.156427407	0.436294207	0.913612665
	H224	-8.646001879	0.452002551	2.485831740
	H225	-7.336466709	-0.181977984	-0.123871799
	H226	-7.294069824	3.869782899	1.504994403
	H227	-9.128979428	8.332470727	3.678147780
	H228	-7.695036839	4.816996121	4.540714679
	H229	-9.470111394	5.118595974	2.399920764
	H230	-6.765989632	6.581940047	2.198631757
	H231	-11.033651524	9.371654210	0.040359300
	H232	-3.505457572	4.136286457	1.062845124
	H233	-4.905275988	3.905650811	0.003990671
	H234	-10.422297258	10.125501041	2.401788036
	H235	-8.686313237	5.433376936	0.850844913
	H236	-6.356683986	1.890160383	0.504739798
	H237	-7.485536858	-0.860594762	2.275798696
	H238	-9.731792258	1.896164913	-1.664581208
	H239	-1.836696919	1.693650615	-2.928446390
	H240	-10.054461167	0.156504562	-1.788827751
	H241	9.695376242	3.142514927	4.506702922
	H242	8.143993087	3.254674614	4.003907757
	H243	1.649890971	6.101404933	-0.733976485
	H244	-0.844205275	5.446126475	0.544515956
	H245	1.492580165	8.043809220	1.174959360
	H246	-3.246988784	3.632331423	-1.900956038
	H247	-4.687256171	4.360529087	-3.270533264
	H248	-6.014101919	3.681792825	-4.952025386
	H249	-4.546224282	5.528992195	-2.199444127
	H250	-4.822593891	1.555195832	-4.807809508
	H251	-6.612013439	1.541580935	-4.903502667
	H252	0.017420889	11.748122882	-0.962280126
	H253	-1.194502720	13.038458417	-0.893479542
	H254	-0.557650592	10.443012729	-2.665504066
	H255	-2.208270404	9.899457636	-2.966781332
	H256	-2.907381931	9.609002197	-5.900681299
	H257	-1.779988215	5.284866232	-5.738578484
	H258	-7.267607860	8.681685156	-1.083337662
	H259	-3.565082904	8.360339441	2.222789335
	H260	-4.300273723	7.980819147	-3.097132864
	H261	1.562813959	5.712179541	-3.260648093
	H262	2.683682050	7.474628083	-2.272620160
	H263	-0.931142083	5.150375837	-3.177346992
	H264	-8.011955347	-0.127381065	-2.833753731
	H265	4.063998969	10.359302138	-2.747348304
	H266	-5.497981840	-0.722133882	-4.075119997
	H267	1.248163707	6.797192682	-4.614417955
	H268	-3.530203685	6.943078798	0.214088362
	H269	3.480428569	10.522984722	-1.079308447
	H270	-2.072717283	8.227251135	-6.521925859
	H271	-4.560109973	9.224480797	-4.276031152
	H272	-4.475904936	-3.222333744	-4.507490031
	H273	-5.508649552	-3.260062615	-1.982634920
	H274	-7.239795783	10.197578645	0.876990033
	H275	-4.950545123	1.515162505	-2.325395614
	H276	-0.053065940	8.321572861	-3.122656116
	H277	-0.888674857	6.811457931	-5.725989505
	H278	0.025974547	4.598915747	-4.543178016
	H279	-3.080767796	6.137634703	-3.771063287
	H280	0.148798306	8.675005996	3.679142974
	H281	-0.657573168	9.755453441	0.740406985
	H282	-0.392351956	12.587340088	1.311741469
	H283	1.714934826	9.280084597	3.092262632
	H284	-2.919308364	10.492637787	2.066875748
	H285	6.068946507	-2.304244627	-8.471185293
	H286	5.918277609	-3.843514446	-7.599400789
	H287	8.721328897	-4.733396805	-4.153586064
	H288	6.751397481	-4.264521321	-9.799474041
	H289	5.199665169	-4.784790277	-9.799282261
	H290	5.518371688	4.922778643	0.317765445
	H291	5.455387700	7.447655925	1.990496442
	H292	5.178617925	8.413217478	-2.164832738
	H293	4.000111206	5.668334549	0.902558465
	H294	6.661807941	8.171958517	1.157339765
	H295	6.392587242	3.386833793	-5.648094521
	H296	6.148385084	7.005916384	-0.827762712
	H297	4.574664073	-7.340873614	5.271131308
	H298	6.698133549	-7.994870483	3.156030007
	H299	4.670801378	1.948832755	-6.005810601
	H300	4.604384942	-0.330712618	4.028588548
	H301	1.910853840	2.179419898	-8.302882629

H302	-0.343128456	-1.760789602	-4.619908927
H303	1.559594632	2.593051084	-6.623522635
H304	3.555948745	-4.802202689	4.785916492
H305	3.130928213	0.535044691	-6.100325264
H306	3.507977532	-5.272033007	3.082290483
H307	4.319630560	-7.521127317	3.518847978
H308	6.016845035	-5.629034216	3.163850951
H309	5.978890541	-5.328775474	4.908294122
H310	-4.075756532	-0.344776704	-7.196360373
H311	5.259364663	-9.772131881	5.083152828
H312	4.593258273	-11.868680569	3.000492870
H313	0.876009831	0.345997251	-4.035267108
H314	5.538640506	-12.191589813	5.748014374
H315	4.052754736	-12.472055850	5.119579819
H316	5.186439714	-3.471521330	2.652396803
H317	-0.005486039	2.525935069	-4.769390187
H318	-2.370732197	-1.659488537	-6.047030525
H319	8.002544113	-9.671591205	-1.126999582
H320	7.286416687	-7.242786065	-3.126559952
H321	6.712409274	-8.062900687	0.390666006
H322	7.362138760	-10.242162430	-2.429044479
H323	6.321298631	3.378069308	-8.456457570
H324	6.390792503	-4.971809209	0.434273782
H325	2.308649812	-7.821513403	0.655712382
H326	4.489196638	-1.427138643	-7.120137237
H327	3.409028954	-9.189859700	-0.983900551
H328	4.293989997	-6.367744666	-6.865611886
H329	3.157535132	-4.980248242	-5.078592559
H330	6.121872469	-12.598881647	3.507791256
H331	5.443224747	-1.201719380	2.782220793
H332	8.219425281	-5.733327634	1.730767190
H333	7.143239815	-3.657398163	0.768062005
H334	8.520943082	-6.938556319	3.710846743
H335	2.888460077	-3.060641832	5.451452817
H336	4.516749950	-8.006847478	-3.588141089
H337	5.521563415	-8.420500203	-2.193164039
H338	2.454866246	-8.628646843	-2.366574651
H339	2.342154573	-1.709771425	-6.002506960
H340	-2.044754044	2.638623316	-6.224145732
H341	-4.031544760	1.437078223	-7.219125724
H342	2.216784485	-6.063122586	0.452144589
H343	8.431626330	-7.245128499	1.426005657
H344	7.414991105	-9.181192924	1.181906501
H345	3.362567246	-1.410054137	5.638753947
H346	6.671709929	-7.059331905	-6.185887361
H347	7.108766302	-5.638161608	-2.440092695
H348	1.700545337	-2.373458127	-7.535007642
H349	5.268365289	4.731997965	-5.931276186
H350	7.065572333	-0.042989003	5.120988365
H351	7.355105104	0.506401160	3.454939698
H352	10.010343341	-6.246158023	-5.398788027
H353	9.197969449	-7.555444993	-4.850704512
H354	10.626329206	-8.312374489	-0.612468043
H355	9.121871618	-7.900311250	-2.273016419
H356	12.359652732	-7.099007601	-2.343551073
H357	10.320688760	-6.645132093	-0.075138352
H358	10.076990547	0.302203288	3.830311130
H359	-3.822671241	12.819802779	4.201566333
H360	9.326363479	-7.144130954	6.601040787
H361	8.794101234	-4.643355732	5.510033449
H362	-5.129692669	12.173084315	1.533544000
H363	-4.822798760	9.991496853	3.692567379
H364	-6.355190630	10.689357387	3.133523039
H365	3.885797144	-0.998545859	1.146390476
H366	-6.246848178	6.902032944	-2.094666858
H367	0.294320720	-0.560441232	-0.304090075
H368	3.200583540	-1.098705118	0.790198908
H369	1.821156411	-2.137202422	0.302626029
H370	-1.823051762	-0.164373771	-1.196615158
Mo371	4.112663726	-4.249066462	-1.991748370
N372	-6.690324053	-7.187484134	-1.959277615
N373	-8.836560133	-4.818105262	-0.015160703
N374	-8.655759197	-5.311071108	2.747474692
N375	-5.988978487	-6.134759412	3.135786690
N376	-4.670544011	-3.662931617	2.666247137
N377	-5.746445835	-2.215897054	4.914580850
N378	-4.872475616	-3.721762010	7.211813802
N379	-2.220296160	-4.991956491	6.949439998
N380	-0.727808126	-2.974831166	5.869648707
N381	-1.472090586	-0.118557311	6.620087892

	N382	-2.208645350	-0.521650462	9.406036778
	N383	4.846023330	-11.829827788	5.088501246
	N384	5.858650167	-9.489165483	4.300621995
	N385	4.556169110	-3.503644616	3.458545671
	N386	3.537385270	-2.308859371	5.198606214
	N387	4.922585433	-1.228990944	3.675442941
	N388	8.433232722	-7.130020738	4.733602894
	N389	-5.246151903	-2.705319998	-4.045116552
	N390	-1.357020206	-5.383720573	-3.908462330
	N391	-5.629606950	-0.281907086	-3.148063664
	N392	-8.098653571	0.694461613	-2.239589291
	N393	-8.040794245	0.179972588	0.526680899
	N394	-6.020016184	1.886302954	1.471777338
	N395	-2.992165168	2.736270279	-1.409027305
	N396	-2.324716872	0.716457076	-1.060518583
	N397	-6.793974133	4.469322029	2.156578136
	N398	-10.026958890	7.538759888	0.570573905
	N399	-10.187113715	9.215529387	2.014155045
	N400	8.934723793	2.608484611	4.079150699
	N401	6.140398207	7.294830880	1.243146219
	N402	4.450928436	8.717956504	-1.523818996
	N403	1.912310161	8.126047585	-2.456612838
	N404	-2.552288963	6.726257074	-4.439542671
	N405	-2.558906492	8.667800519	-5.748885650
	N406	-3.948805105	8.502580359	-3.903837186
	N407	0.698239680	6.415766551	-0.486606577
	N408	0.626979050	8.149585390	1.711773611
	N409	-0.684703174	10.555736812	1.372152104
	N410	-1.569786079	10.561647944	-2.530083503
	N411	-3.228251249	11.461468971	2.085178193
	N412	5.781581653	-3.942627314	-9.765065672
	N413	3.735067717	-1.968793916	-7.552401536
	N414	2.582081979	0.774719441	-6.940012117
	N415	4.619855971	2.807709964	-6.556404002
	N416	-0.025687599	-2.231526951	-10.388856868
	N417	-1.861283485	-0.552277953	-8.981438971
	N418	9.089193891	-6.563363700	-5.083282855
	N419	5.004408402	-5.223842093	-3.984887085
	N420	4.747932493	-6.083605145	-6.001324910
	N421	9.763798353	-7.126965780	-2.075800384
	N422	0.142737724	2.572038125	-0.448598826
	N423	-0.311636921	-4.152713798	-0.403356952
	N424	1.048657149	1.986381874	-0.789474882
	N425	0.644552715	-3.658766819	-0.743095943
	O426	-6.615246634	-5.270946653	0.200068806
	O427	-7.858928656	-3.196085802	3.062324485
	O428	-6.219274925	-5.709015837	5.368769223
	O429	-3.152091970	-4.346193611	4.220182989
	O430	-3.838066986	-0.984759929	4.852830896
	O431	-4.675133023	-1.700997292	8.271496921
	O432	-2.018489377	-4.642161244	9.205020461
	O433	1.245064706	-4.139890046	6.108503174
	O434	0.801579449	-0.178548814	6.841977825
	O435	-0.744438463	1.164931040	9.810836144
	O436	-0.449728020	-1.118465542	12.471732372
	O437	6.841052302	-10.375367433	2.405458971
	O438	7.159633139	-8.040199824	6.418852566
	O439	10.751352783	-4.723650106	6.078981405
	O440	-5.504477210	-0.848509178	-0.928538515
	O441	-2.896344897	-4.052105362	-4.925336535
	O442	-7.017534647	2.494870602	-1.346239820
	O443	-5.784540969	3.280757090	-4.094625539
	O444	-10.045780087	1.266993882	0.781278417
	O445	-6.298692532	1.119429678	3.604796548
	O446	-4.902627837	4.725999684	3.410992834
	O447	-7.458971078	6.837284178	4.710627145
	O448	9.970140478	-0.310211238	5.774647582
	O449	2.762637241	4.383968749	4.779753881
	O450	3.510319376	8.459292943	0.537161579
	O451	1.342311383	10.351283140	-2.564716711
	O452	-1.289513720	7.284098713	-1.207771943
	O453	-1.191730786	6.999970164	2.515343400
	O454	2.053048120	5.619104830	2.236863591
	O455	0.202748785	11.403673237	3.309060590
	O456	-2.814473405	13.532527427	1.224690167
	O457	-3.222303080	11.088640780	-1.042852415
	O458	-5.718187675	13.430545362	3.746702161
	O459	-5.353945866	6.913691654	-1.698596108
	O460	3.232944348	-3.918936695	-8.660183606
	O461	1.144482509	-0.092390977	-8.502274897

	O462 3.388397369 4.228131092 -7.834519352<br>O463 7.113951079 5.125238815 -7.758506607<br>O464 -3.475295192 -2.014619809 -9.655403239<br>O465 -0.364108134 -6.137358267 -6.633070268<br>O466 1.642404030 -5.241525944 -7.015330496<br>O467 -2.632053467 2.923885626 -8.786701453<br>O468 10.508956597 -5.018130711 -2.443750096<br>O469 12.912013084 -6.570764992 -0.450762382<br>O470 0.624706947 -6.609232999 -1.910422990<br>O471 -0.172614200 -7.607392630 -0.065614500<br>O472 5.874496950 -10.656706564 -3.229582098<br>O473 3.662021497 -10.665924602 -3.697281854<br>O474 5.337603446 -5.759823173 -0.913872365<br>O475 5.075893084 -7.534066894 0.440410490<br>O476 3.036908048 -6.031737659 -2.101975097<br>O477 8.777620365 -6.503663865 1.998316773<br>O478 6.871990006 -4.507643269 1.166447773<br>O479 -4.025308883 4.916015851 -2.776053855<br>O480 8.248705751 -10.099698545 -1.977015900<br>O481 7.605504612 -8.491172183 0.497546814<br>S482 3.880789507 5.183339372 -1.481735974<br>S483 2.787400043 2.241969742 -3.132703478<br>S484 2.479688007 -3.130334476 -3.297498315<br>S485 3.717455655 1.888625321 0.394707967<br>S486 0.888486293 -0.781691561 -1.507407150<br>S487 4.869560813 -0.563435866 -4.843489522<br>S488 3.564845629 -3.677446244 0.207277476<br>S489 6.183357912 1.930961738 -2.184735095<br>S490 5.997127264 -2.797105431 -2.029056445<br>S491 6.310531366 -0.822213638 0.609674853<br>end

S2BH-2N2x-6N2x-7H2n-6Hn (20) to 2N2x-6N2x-7H2n-6H2n (21)

TS

Fe(139) -2.372<br>Fe(140) -1.309<br>Fe(141) -2.457<br>Fe(142) 2.454<br>Fe(143) 2.885<br>Fe(144) 0.567<br>Fe(145) -0.007<br><br>**S=0**	2n2x6n2x6h2n7hns2bhrte_3_53345.36<br>C1 -7.709454700 -5.495266195 -0.354374381<br>C2 -7.889701754 -6.577814800 -1.423845692<br>C3 -8.809422590 -3.693633757 0.902944311<br>C4 -8.412517614 -4.050854492 2.332954873<br>C5 -8.265407106 -5.776555589 4.060682617<br>C6 -6.747028712 -5.871752908 4.244770048<br>C7 -4.559154795 -6.061990806 3.182043575<br>C8 -4.059600564 -4.631809910 3.402917298<br>C9 -4.393691785 -2.259611910 2.873541996<br>C10 -4.634049779 -1.775290561 4.311270183<br>C11 -6.037370627 -1.783443255 6.278632375<br>C12 -5.122115336 -2.407141375 7.341687242<br>C13 -4.242604993 -4.493287508 8.286030914<br>C14 -2.723227529 -4.698699482 8.187466679<br>C15 -0.808053338 -5.229249724 6.733128462<br>C16 0.001220343 -4.041111164 6.204384840<br>C17 -0.178002792 -1.735205042 5.307897998<br>C18 -0.242613923 -0.585780492 6.327093222<br>C19 -0.786713943 -1.425615091 3.917845079<br>C20 -0.322569376 -0.071607170 3.368777506<br>C21 -0.465038989 -2.548966066 2.925663161<br>C22 -1.629538903 1.018239486 7.557123453<br>C23 -1.497877856 0.566206498 9.024465153<br>C24 -2.937986674 1.793389629 7.274007511<br>C25 -3.174876500 2.874397643 8.333639529<br>C26 -2.901454767 2.414176421 5.870591655<br>C27 -2.192065327 -0.934405666 10.797076550<br>C28 -0.780038802 -1.183038063 11.289396137<br>C29 5.404431263 -11.787249075 3.719013123<br>C30 6.117131807 -10.475598159 3.411550959<br>C31 6.271064232 -8.108306704 4.159891602<br>C32 7.336382842 -7.768376313 5.218820614<br>C33 5.044180016 -7.189793700 4.285991100<br>C34 5.398418307 -5.721915362 4.070299137<br>C35 4.163982940 -4.845074946 3.878353822<br>C36 4.323164902 -2.348086705 4.102134386<br>C37 9.530062262 -6.740775104 5.592115539<br>C38 9.690895562 -5.242277745 5.728785825<br>C39 -4.955319861 -2.541551653 -2.625452777<br>C40 -5.393660496 -1.147422308 -2.139475839<br>C41 -3.461587029 -2.758248311 -2.318561807<br>C42 -2.981832364 -4.212720326 -2.510597572<br>C43 -2.406604452 -4.531286557 -3.888697847

	C44	-5.788438502	1.153219930	-2.936585432
	C45	-7.029516312	1.518253914	-2.112246409
	C46	-5.752135423	1.861797362	-4.296952297
	C47	-9.313479079	0.905440459	-1.472110262
	C48	-9.159557518	0.801661418	0.049191711
	C49	-7.738478345	0.153706111	1.938957760
	C50	-6.623917671	1.088386556	2.409886930
	C51	-5.000864484	2.837498081	1.871837463
	C52	-5.569574567	4.090046622	2.583776984
	C53	-4.210280159	3.363779902	0.664516716
	C54	-3.430284331	2.371654490	-0.136363986
	C55	-2.933690815	1.114241059	0.105109612
	C56	-2.264177495	1.714658986	-1.927309974
	C57	-7.400158339	5.744533227	2.545500381
	C58	-7.511495078	5.810156953	4.066582819
	C59	-8.800455571	5.818327642	1.888088928
	C60	-9.412051710	7.183421949	1.763415663
	C61	-9.507508270	8.217136420	2.674751903
	C62	-10.487509689	8.776162709	0.767586073
	C63	8.566127421	1.497839868	4.947665393
	C64	9.632120991	0.422371511	4.841478345
	C65	7.235422465	0.849965295	4.491362752
	C66	6.051654521	1.782642719	4.589793778
	C67	5.289098562	1.875163912	5.768799708
	C68	5.675865486	2.585737956	3.499054270
	C69	4.183863720	2.729432839	5.856381590
	C70	4.587218601	3.454338972	3.576668368
	C71	3.836398632	3.519113486	4.754158059
	C72	5.410210071	7.035552895	-0.014444274
	C73	4.365105422	8.127431591	-0.299884677
	C74	4.742271794	5.664096786	0.083697457
	C75	3.585013176	9.827920585	-1.914433160
	C76	2.164033107	9.453840278	-2.341691830
	C77	0.593721516	7.552960304	-2.705934823
	C78	-0.083390581	7.076376222	-1.395953331
	C79	0.796960094	6.385081235	-3.701673869
	C80	-0.402024072	5.506583510	-4.078570761
	C81	-1.405771578	6.092772537	-5.084199405
	C82	-3.038283199	7.943190790	-4.721184773
	C83	0.103860822	5.949370718	0.766051431
	C84	-0.240249647	7.090557419	1.735492402
	C85	1.006190484	4.950652628	1.520454022
	C86	0.656467657	9.098968474	2.810954982
	C87	0.036780656	10.459944023	2.528040085
	C88	-1.106472289	11.863951595	0.891318272
	C89	-2.477667819	12.359226912	1.407896175
	C90	-1.012344747	11.991968448	-0.631875561
	C91	-2.045147070	11.171912681	-1.396454255
	C92	-4.627900928	11.747929797	2.415083939
	C93	-4.728569637	12.761035502	3.545645206
	C94	-5.344285983	10.428696308	2.822639294
	C95	-5.390266086	9.433456334	1.684574871
	C96	-6.429661660	9.489903711	0.742115246
	C97	-4.379368063	8.477161166	1.496616979
	C98	-6.446114335	8.645077826	-0.368706479
	C99	-4.366661522	7.638131801	0.377612138
	C100	-5.402301832	7.735832871	-0.546729134
	C101	5.546449936	-3.275999042	-8.476340035
	C102	4.059154956	-3.076731899	-8.246617153
	C103	2.378093059	-1.643224312	-7.103457831
	C104	1.962240636	-0.247520175	-7.581587406
	C105	2.318725134	2.163893842	-7.282615711
	C106	3.501235420	3.130217825	-7.261702007
	C107	5.665915277	3.790392686	-6.373091356
	C108	6.399862314	4.133215922	-7.652750517
	C109	-1.190335037	-2.812432407	-9.707095564
	C110	-2.277174826	-1.761438844	-9.457161059
	C111	-0.914765852	-3.595398135	-8.403123161
	C112	-0.108936796	-4.879193395	-8.676614719
	C113	0.361816849	-5.490504191	-7.375307593
	C114	-2.825616829	0.507238485	-8.734310921
	C115	-2.286721758	1.835536597	-9.227129564
	C116	-3.418645796	0.535910176	-7.304713421
	C117	-2.334637472	0.505807886	-6.263630577
	C118	-1.678826561	1.684935923	-5.871852144
	C119	-1.864093038	-0.726303193	-5.778166504
	C120	-0.535819467	1.622613348	-5.069043349
	C121	-0.728446158	-0.785054882	-4.968453371
	C122	-0.041313481	0.387719563	-4.638660018
	C123	8.696519114	-5.801537449	-3.882388231

	C124	9.714217286	-5.936285816	-2.738921282
	C125	7.290513999	-6.157536551	-3.370964623
	C126	6.157668192	-5.907170662	-4.312963880
	C127	5.998710627	-6.430741169	-5.579472364
	C128	4.150991973	-5.359919644	-4.999254926
	C129	10.641978789	-7.249907324	-0.928725568
	C130	12.076084115	-6.914287300	-1.273392559
	C131	0.769133872	-7.171117666	-0.686059219
	C132	2.167507186	-7.057656064	-0.090931193
	C133	3.306523458	-7.068805631	-1.145339957
	C134	3.387566354	-8.465819871	-1.815430116
	C135	4.599990508	-8.687388998	-2.720088436
	C136	4.724659021	-10.131499947	-3.254583030
	C137	4.657287510	-6.793984028	-0.468707714
	C138	3.911618986	-0.445827684	-1.681558370
	Fe139	4.288314061	-1.763172998	-3.057567509
	Fe140	2.414058361	0.925850842	-1.381869622
	Fe141	5.262109868	0.604691334	-0.690508234
	Fe142	4.478260866	0.749762352	-3.131040167
	Fe143	4.220309440	2.911877140	-1.514436783
	Fe144	4.644123100	-1.870868873	-0.476414603
	Fe145	2.103054446	-2.597103262	-1.006063104
	H146	-6.102992763	-7.531399522	-1.175202558
	H147	-6.108584306	-6.475270858	-2.420425305
	H148	-9.691634871	-4.975093392	-0.559857605
	H149	-8.479826066	-6.160194207	-2.255571524
	H150	-8.520739040	-7.363478835	-0.973040002
	H151	-8.093351337	-2.931134300	0.563182423
	H152	-9.122273011	-5.956185663	2.095914702
	H153	-9.805821846	-3.232418131	0.924712862
	H154	-8.706626140	-6.767148201	4.233735764
	H155	-6.464083509	-6.138059293	2.219531103
	H156	-8.632215924	-5.097301032	4.840907082
	H157	-3.344949610	-2.044578102	2.633109518
	H158	-5.397948464	-3.940715567	1.999837198
	H159	-4.155499212	-6.451844862	2.237772174
	H160	-5.923574926	-0.693926118	6.353804943
	H161	-6.466425906	-2.724358663	4.384278064
	H162	-4.172801511	-6.672726851	4.006942856
	H163	-5.025593099	-1.686912741	2.181054380
	H164	-5.349789119	-4.260848427	6.469551966
	H165	-7.077136910	-2.046790482	6.517299917
	H166	-0.817471092	-3.526653683	3.280719625
	H167	-0.945467722	-2.356113916	1.953577087
	H168	0.621087068	-2.611522773	2.749091812
	H169	-1.876916211	-1.376345656	4.056302718
	H170	-4.711145424	-5.487541237	8.331685542
	H171	-2.779376935	1.657881157	5.083315884
	H172	-2.918678344	-0.943630329	8.790773925
	H173	-3.306765605	2.449784635	9.339437437
	H174	0.725933334	-0.105184865	3.034693581
	H175	-0.421019506	0.739216700	4.104829772
	H176	8.464145064	1.762750390	6.017762111
	H177	-3.838527353	2.951990827	5.668497556
	H178	-4.078542841	3.449201646	8.084715047
	H179	-2.071617159	3.135660181	5.789348664
	H180	-2.325433076	3.574710441	8.369928480
	H181	-3.774652952	1.072718417	7.317745970
	H182	-0.776826843	1.696765058	7.404772230
	H183	-2.296690169	-0.426005351	6.106659496
	H184	-0.936192575	0.208502003	2.498712024
	H185	1.433417925	4.238635475	0.799101944
	H186	0.358372572	4.395853897	2.215356208
	H187	2.161109662	5.183288909	3.098460177
	H188	2.292466386	4.308661959	5.625479582
	H189	4.296703639	4.059974623	2.717654146
	H190	6.226637915	2.511328389	2.558964999
	H191	5.554663696	1.261832789	6.634497869
	H192	-1.744830908	-3.041872586	5.935124126
	H193	-0.684262754	-6.055375307	6.017393013
	H194	-0.356421492	-5.538500862	7.685081399
	H195	-2.835923443	-4.989842859	6.127522931
	H196	-4.418474566	-3.981377187	9.239534044
	H197	3.589523437	2.771845124	6.772944799
	H198	0.894073070	-1.925586203	5.182214717
	H199	-2.680986861	-0.208166996	11.466901074
	H200	-2.733022141	-1.891040118	10.871698566
	H201	-0.053828302	-1.489362753	10.500269852
	H202	10.481833705	-7.177992293	5.251897552
	H203	-0.979491419	-5.670018414	-4.821862328

	H204	1.554130908	-6.258839750	-2.180825298
	H205	-0.752931034	-5.611913985	-9.184374036
	H206	0.748802919	-4.661602101	-9.329047363
	H207	-1.863851346	-3.868536618	-7.916559592
	H208	-1.559814002	1.749653115	-10.069023581
	H209	-1.663195958	-3.509150954	-10.415833118
	H210	-2.864811218	-2.077779672	-2.947653174
	H211	-3.820054322	-4.911772259	-2.344814698
	H212	-0.872061168	-0.386320891	-8.784419897
	H213	-3.668036290	0.295090666	-9.424798801
	H214	0.561953310	-1.690763905	-9.745137123
	H215	-0.935014091	-5.767541438	-3.070976742
	H216	-0.379127245	-2.941836655	-7.696476329
	H217	0.556491427	-2.977675575	-10.773943882
	H218	-6.113185030	-3.219916900	-4.197923870
	H219	-3.291757817	-2.471442214	-1.269855902
	H220	2.130375970	-4.705597159	-7.710696476
	H221	-2.239780950	-4.453511523	-1.740318999
	H222	-4.337842736	2.354226001	2.603049776
	H223	-3.058058818	0.456486865	0.955989839
	H224	-8.648937161	0.447650121	2.479534236
	H225	-7.336333082	-0.178734282	-0.130826295
	H226	-7.303357612	3.872633677	1.509253583
	H227	-9.139971313	8.334572071	3.687941453
	H228	-7.695135643	4.819435214	4.548035363
	H229	-9.479776749	5.124029330	2.413094495
	H230	-6.775618541	6.585263950	2.203270815
	H231	-11.028120431	9.381021247	0.043489054
	H232	-3.511548374	4.125119674	1.048894691
	H233	-4.907945948	3.884692944	-0.010315093
	H234	-10.423778891	10.131843735	2.407970631
	H235	-8.699227347	5.433504659	0.861388206
	H236	-6.366535722	1.891264047	0.504156951
	H237	-7.487929221	-0.866406137	2.265591384
	H238	-9.731576281	1.903081744	-1.667277299
	H239	-1.781027987	1.698488096	-2.901983461
	H240	-10.055703432	0.163944099	-1.795441419
	H241	9.689840779	3.152287108	4.507233746
	H242	8.137992325	3.264218663	4.006944399
	H243	1.647183145	6.106483083	-0.736352731
	H244	-0.846233573	5.446004250	0.537415048
	H245	1.481759633	8.048710973	1.173623685
	H246	-3.269261223	3.596390026	-1.909644238
	H247	-4.696285365	4.370077640	-3.274695605
	H248	-6.016175397	3.688199064	-4.959252586
	H249	-4.550844169	5.529778734	-2.196066860
	H250	-4.822281794	1.566522163	-4.810662573
	H251	-6.611935116	1.545963157	-4.908078952
	H252	0.011233046	11.756334729	-0.955030109
	H253	-1.198561927	13.048295375	-0.882186223
	H254	-0.565579104	10.457604417	-2.663162819
	H255	-2.217644941	9.920314322	-2.969021349
	H256	-2.923778163	9.609475346	-5.907983933
	H257	-1.784473710	5.294132024	-5.740059843
	H258	-7.270419339	8.690239682	-1.084864330
	H259	-3.574962555	8.363657540	2.228524734
	H260	-4.301392432	7.989292075	-3.093872509
	H261	1.560770772	5.720279665	-3.265548418
	H262	2.680913000	7.483689947	-2.274338379
	H263	-0.933959188	5.157511396	-3.179481467
	H264	-8.011470293	-0.120955438	-2.838490661
	H265	4.056973520	10.371894826	-2.742939344
	H266	-5.492876866	-0.710913600	-4.081804074
	H267	1.243237682	6.807434427	-4.617046799
	H268	-3.538069297	6.946144181	0.219320752
	H269	3.473133109	10.529695543	-1.074641270
	H270	-2.093976255	8.226090462	-6.530199282
	H271	-4.569945793	9.225763144	-4.277778036
	H272	-4.474310571	-3.208144301	-4.518867391
	H273	-5.522543169	-3.254995917	-1.998267239
	H274	-7.245182513	10.204822957	0.876525513
	H275	-4.949957506	1.520959391	-2.329465306
	H276	-0.056861585	8.328118038	-3.122401410
	H277	-0.894840694	6.822009035	-5.725943908
	H278	0.021885720	4.607226205	-4.546510950
	H279	-3.083808144	6.143884881	-3.768034124
	H280	0.135227080	8.670957975	3.679671878
	H281	-0.666034902	9.759107845	0.742406537
	H282	-0.397894027	12.588918928	1.321181337
	H283	1.702425546	9.277966281	3.097173460

	H284	-2.930541720	10.494947776	2.063830368
	H285	6.062196578	-2.304496779	-8.465910208
	H286	5.910456062	-3.843691042	-7.594858005
	H287	8.716343695	-4.738159422	-4.163722896
	H288	6.748695697	-4.263643654	-9.792819451
	H289	5.197611776	-4.786098495	-9.795646462
	H290	5.522965014	4.928082705	0.313400792
	H291	5.450195566	7.452897702	1.984539918
	H292	5.174536258	8.425790021	-2.167097170
	H293	4.000343148	5.668597027	0.894527860
	H294	6.653609453	8.182374828	1.152173758
	H295	6.403296779	3.367031177	-5.673755728
	H296	6.146262060	7.013484487	-0.832900367
	H297	4.578554805	-7.341109657	5.274038136
	H298	6.697936391	-7.996611020	3.154928249
	H299	4.657384275	1.950207533	-5.997273289
	H300	4.600711984	-0.316526127	4.024041744
	H301	1.894477140	2.181960732	-8.293255436
	H302	-0.366874886	-1.746013701	-4.595022197
	H303	1.558499422	2.596309448	-6.609988303
	H304	3.567529528	-4.797792244	4.796497990
	H305	3.096731967	0.537554889	-6.082071492
	H306	3.516188472	-5.263553857	3.091089884
	H307	4.320448038	-7.516417696	3.521942567
	H308	6.024883720	-5.628011279	3.170916744
	H309	5.987033008	-5.331854441	4.915810764
	H310	-4.080409306	-0.336326932	-7.201608660
	H311	5.254977018	-9.776898956	5.076217447
	H312	4.587673350	-11.866689093	2.985211452
	H313	0.856799381	0.340849276	-4.023388538
	H314	5.525543822	-12.205009703	5.732693713
	H315	4.039447649	-12.473570287	5.099815824
	H316	5.209324557	-3.470358548	2.669397951
	H317	-0.009649350	2.533846006	-4.776964235
	H318	-2.382825307	-1.649451506	-6.046460594
	H319	8.008316325	-9.663553938	-1.127663114
	H320	7.272839681	-7.227936719	-3.103141395
	H321	6.712259141	-8.058346781	0.392565964
	H322	7.373912242	-10.237841605	-2.431408392
	H323	6.295197936	3.388520097	-8.477337268
	H324	6.389335320	-4.979561064	0.432852861
	H325	2.305054533	-7.855937478	0.648328327
	H326	4.479374325	-1.422392192	-7.127940168
	H327	3.408888655	-9.199839282	-0.994713272
	H328	4.283837612	-6.350158022	-6.857131813
	H329	3.135804636	-4.990330230	-5.058645807
	H330	6.113986644	-12.601233945	3.492591832
	H331	5.487001025	-1.193702848	2.810070499
	H332	8.217695975	-5.723443473	1.732307368
	H333	7.130852111	-3.658823770	0.762748998
	H334	8.516863066	-6.933783232	3.712316602
	H335	2.875388268	-3.051913059	5.440043921
	H336	4.532309935	-8.017127574	-3.591438271
	H337	5.527040827	-8.432091483	-2.188808956
	H338	2.460887029	-8.642023490	-2.382344308
	H339	2.328058988	-1.706006734	-6.004613541
	H340	-2.047753298	2.646983432	-6.235203243
	H341	-4.033720967	1.445254731	-7.229388370
	H342	2.193462168	-6.097918017	0.453687106
	H343	8.431806306	-7.234505666	1.428901330
	H344	7.417459778	-9.175099415	1.182725674
	H345	3.347526990	-1.400309807	5.629425621
	H346	6.665021455	-7.033272353	-6.183703382
	H347	7.107441245	-5.614743199	-2.436990792
	H348	1.689765376	-2.370640421	-7.538530082
	H349	5.276910281	4.717559176	-5.923023416
	H350	7.067020096	-0.038908129	5.116492581
	H351	7.356743896	0.511679266	3.449463336
	H352	9.991887990	-6.270787445	-5.399420086
	H353	9.172663732	-7.569817184	-4.836405432
	H354	10.624198430	-8.304155987	-0.609837012
	H355	9.124351479	-7.901733001	-2.275251602
	H356	12.354176639	-7.085281242	-2.341408238
	H357	10.313326946	-6.637317291	-0.074142777
	H358	10.078152036	0.316442799	3.822738053
	H359	-3.831642000	12.822494736	4.206460162
	H360	9.324064347	-7.137501391	6.601952498
	H361	8.775171680	-4.641408951	5.503777017
	H362	-5.137642123	12.177516122	1.537123398
	H363	-4.832446737	9.995712823	3.696037850

H364	-6.364143960	10.694213021	3.135814366
H365	4.160797553	-1.247057720	0.986993459
H366	-6.248211536	6.909771025	-2.095295682
H367	1.464472017	-1.489052328	0.042510843
H368	3.509642299	-1.108212560	0.500636419
H369	1.855831061	-2.081382167	0.553471934
H370	-1.565927086	-0.067702060	-1.097747094
Mo371	4.119382383	-4.246995176	-1.963900926
N372	-6.677001091	-7.180896940	-1.946203843
N373	-8.845831784	-4.819144857	-0.017073721
N374	-8.677162135	-5.312581044	2.745980280
N375	-6.012615831	-6.138892817	3.135421331
N376	-4.683511881	-3.671856372	2.673745763
N377	-5.752900519	-2.226512875	4.923462561
N378	-4.884979988	-3.739450321	7.218005261
N379	-2.222788898	-4.975295522	6.948274930
N380	-0.732221362	-2.953046920	5.875331515
N381	-1.477029805	-0.100811082	6.626324565
N382	-2.222621978	-0.516260541	9.406928928
N383	4.836339526	-11.835639690	5.073882374
N384	5.854351759	-9.492343190	4.294353194
N385	4.572256909	-3.499600207	3.470217556
N386	3.520869456	-2.297400545	5.185204902
N387	4.898309036	-1.216184028	3.657399437
N388	8.431024691	-7.130260658	4.733993892
N389	-5.247672900	-2.695684633	-4.057645202
N390	-1.369577771	-5.390233790	-3.914867995
N391	-5.631932814	-0.274347292	-3.154387975
N392	-8.099804100	0.701761747	-2.245819552
N393	-8.042257872	0.180326199	0.520313857
N394	-6.031740589	1.888601556	1.471638251
N395	-3.000752149	2.710535234	-1.413194362
N396	-2.222946756	0.732996860	-1.018775065
N397	-6.800860056	4.472317183	2.158876821
N398	-10.028508749	7.545103120	0.576670713
N399	-10.189827527	9.221396385	2.020716831
N400	8.929611150	2.618711564	4.078318965
N401	6.136887470	7.302368697	1.238173153
N402	4.446635458	8.727076142	-1.524670632
N403	1.908668487	8.134065947	-2.457715074
N404	-2.556747180	6.733220648	-4.438022795
N405	-2.569215278	8.671038029	-5.752783734
N406	-3.952877170	8.510441857	-3.902350599
N407	0.696195342	6.421136648	-0.488374906
N408	0.616653007	8.150935198	1.711827455
N409	-0.693800973	10.557801118	1.376185296
N410	-1.577469943	10.578178812	-2.527921716
N411	-3.236408262	11.464645052	2.088950192
N412	5.778308714	-3.943159283	-9.760465412
N413	3.725554724	-1.970287708	-7.551716617
N414	2.571925834	0.775788489	-6.936514940
N415	4.616946277	2.800097086	-6.562006805
N416	-0.027006389	-2.232032990	-10.388914348
N417	-1.862847426	-0.549667046	-8.985628785
N418	9.070063545	-6.579101013	-5.077384370
N419	4.987283979	-5.228817666	-3.966230495
N420	4.734706690	-6.072079956	-5.989250071
N421	9.759001480	-7.122927471	-2.074628481
N422	0.149060523	2.636223776	-0.524558874
N423	-0.317220592	-4.155050770	-0.454938282
N424	1.021555381	2.013371721	-0.872811261
N425	0.626612311	-3.627519341	-0.765039048
O426	-6.626262139	-5.270136955	0.215007188
O427	-7.873138236	-3.200996163	3.062947155
O428	-6.244087351	-5.722868606	5.370540214
O429	-3.155209657	-4.368803328	4.211356876
O430	-3.833290175	-1.012496604	4.874704572
O431	-4.680153834	-1.720952561	8.279606759
O432	-2.028790572	-4.663581173	9.210662806
O433	1.237920650	-4.128003151	6.085123826
O434	0.797420189	-0.152237591	6.847834871
O435	-0.761932034	1.170666227	9.822993436
O436	-0.461797170	-1.127236078	12.467859024
O437	6.837586556	-10.375074762	2.398093378
O438	7.164221128	-8.055548333	6.416275930
O439	10.730507323	-4.706470370	6.079446169
O440	-5.518551135	-0.848623914	-0.936098334
O441	-2.891169699	-4.041705384	-4.934207958
O442	-7.020214382	2.501780890	-1.350814529
O443	-5.792607146	3.288764817	-4.099552690

O444	-10.046861373	1.267926124	0.776711001
O445	-6.293592116	1.101374728	3.599617235
O446	-4.902065006	4.729865762	3.401727257
O447	-7.460805490	6.839944817	4.716450757
O448	9.973463792	-0.300683714	5.766009153
O449	2.752165923	4.374850067	4.768186254
O450	3.505561289	8.459432571	0.534778492
O451	1.335787609	10.358944015	-2.561711356
O452	-1.290916720	7.292182036	-1.207328428
O453	-1.196759240	6.992497375	2.514960439
O454	2.051528845	5.620709385	2.229657219
O455	0.190619777	11.400975563	3.316304457
O456	-2.816962306	13.539376918	1.240077161
O457	-3.229948160	11.104162320	-1.040061692
O458	-5.726197236	13.434642247	3.749857763
O459	-5.356282397	6.920086798	-1.696986972
O460	3.228039454	-3.923446739	-8.656384627
O461	1.150458236	-0.089502856	-8.515185774
O462	3.391719121	4.220969366	-7.844631293
O463	7.093030294	5.130854977	-7.773649047
O464	-3.477326071	-2.014376091	-9.653503418
O465	-0.369624148	-6.142165608	-6.636528608
O466	1.635535315	-5.241473204	-7.014632063
O467	-2.635396275	2.927154655	-8.800218640
O468	10.492855008	-5.009784297	-2.440644573
O469	12.904923766	-6.556312789	-0.448200206
O470	0.615119422	-6.632691936	-1.910299211
O471	-0.175643239	-7.671816694	-0.085860967
O472	5.890068121	-10.662406632	-3.231065174
O473	3.678562276	-10.678268400	-3.703412892
O474	5.338680223	-5.780064867	-0.930950230
O475	5.067507840	-7.537081006	0.442954449
O476	3.022079642	-6.028798694	-2.088158002
O477	8.776590510	-6.492343432	2.000804934
O478	6.859813743	-4.507251422	1.165525223
O479	-4.032633542	4.921433619	-2.778762946
O480	8.258965059	-10.086817281	-1.978715258
O481	7.606398845	-8.481896882	0.500709648
S482	3.889289033	5.183063808	-1.490058745
S483	2.803318759	2.250069145	-3.193641238
S484	2.477436760	-3.108902970	-3.212305587
S485	3.672449674	1.850322464	0.359660999
S486	0.843983062	-0.704287410	-1.626067755
S487	4.840252453	-0.573940866	-4.826843955
S488	3.581152826	-3.735656520	0.235681733
S489	6.179952500	1.930406373	-2.196948443
S490	6.006184292	-2.806172571	-2.052077540
S491	6.387634397	-0.813872628	0.568004907
end			

2N2x-6N2x-7H2n-6H2n (21)

Fe(139) -2.362
Fe(140) -1.330
Fe(141) -2.409
Fe(142) 2.443
Fe(143) 2.889
Fe(144) 0.527
Fe(145) -0.025

S=0

2n2x6n2x6h2n7hns2bhrte.car_5

C1	-7.713039936	-5.500329825	-0.342232117
C2	-7.871022588	-6.582950477	-1.414130275
C3	-8.830439113	-3.700213977	0.900362943
C4	-8.435726047	-4.058522288	2.331097003
C5	-8.305324851	-5.784244571	4.060463041
C6	-6.787763899	-5.888111967	4.246696859
C7	-4.599690094	-6.092894457	3.184085462
C8	-4.073682206	-4.671868712	3.401864666
C9	-4.376078878	-2.291758840	2.901002295
C10	-4.616650779	-1.817178702	4.341388642
C11	-6.040588370	-1.819778576	6.295500215
C12	-5.129291989	-2.452408211	7.356811122
C13	-4.251925480	-4.543744510	8.289070985
C14	-2.730975350	-4.730534046	8.192123264
C15	-0.804011318	-5.191311062	6.728867547
C16	0.001569311	-4.004333794	6.195791293
C17	-0.162318335	-1.689698194	5.330703709
C18	-0.239328815	-0.536783854	6.344005038
C19	-0.728276151	-1.372187513	3.923161533
C20	-0.185062466	-0.042519452	3.390941215
C21	-0.423407997	-2.517639754	2.952362413
C22	-1.648993419	1.047637982	7.576270169
C23	-1.526405780	0.583854452	9.039909991
C24	-2.961638897	1.813914906	7.288728861
C25	-3.205514219	2.897136706	8.344650945
C26	-2.927044699	2.429368541	5.882848619
C27	-2.213994471	-0.947580750	10.790992310

	C28	-0.800195559	-1.195566848	11.279699293
	C29	5.405233941	-11.795952351	3.690391685
	C30	6.123337609	-10.483943864	3.393436805
	C31	6.285160533	-8.122392884	4.153806834
	C32	7.352643750	-7.790475854	5.212237260
	C33	5.064449171	-7.196353676	4.287221718
	C34	5.429272047	-5.729171519	4.081272654
	C35	4.202901861	-4.837170341	3.906578586
	C36	4.328456632	-2.337071677	4.104388693
	C37	9.530915735	-6.730740822	5.590775257
	C38	9.664792317	-5.228934484	5.721428219
	C39	-4.962088358	-2.532082647	-2.655351128
	C40	-5.388073985	-1.135845376	-2.164251753
	C41	-3.474302886	-2.772230701	-2.331617207
	C42	-3.015530114	-4.230255493	-2.539020903
	C43	-2.415390699	-4.531838678	-3.910612289
	C44	-5.777147933	1.168266554	-2.953964745
	C45	-7.017999693	1.530923452	-2.127859548
	C46	-5.744420565	1.876433955	-4.314655521
	C47	-9.301798896	0.916061918	-1.489869829
	C48	-9.148498984	0.802649617	0.031653724
	C49	-7.725051544	0.139828687	1.916076906
	C50	-6.605554959	1.066284225	2.391355400
	C51	-5.000582540	2.838809013	1.865761953
	C52	-5.568617710	4.095480042	2.569788013
	C53	-4.196391702	3.357902080	0.663550865
	C54	-3.371665555	2.375932965	-0.105579905
	C55	-2.786460366	1.165820521	0.179547965
	C56	-2.197876489	1.713665233	-1.889452318
	C57	-7.409133039	5.741629168	2.556384630
	C58	-7.508926082	5.805160382	4.077816987
	C59	-8.812330689	5.818483853	1.907530010
	C60	-9.415494986	7.187383224	1.780658694
	C61	-9.511014033	8.220696621	2.692762274
	C62	-10.474236715	8.788286772	0.779544963
	C63	8.575433984	1.510730147	4.933306421
	C64	9.647699178	0.442115610	4.823749233
	C65	7.248416673	0.857960471	4.472373058
	C66	6.060661063	1.786071214	4.569890580
	C67	5.297598954	1.876414007	5.748667977
	C68	5.680907714	2.585432309	3.477897707
	C69	4.187301266	2.724117724	5.834364744
	C70	4.587507226	3.448434006	3.553936702
	C71	3.836042013	3.510267800	4.730925122
	C72	5.416343735	7.041062662	-0.033459202
	C73	4.367577789	8.130910535	-0.312294871
	C74	4.754228705	5.665579389	0.055130276
	C75	3.583921571	9.841341532	-1.913854762
	C76	2.164017330	9.464956287	-2.342244782
	C77	0.598303236	7.561629562	-2.712509803
	C78	-0.076096853	7.084544785	-1.401262658
	C79	0.799280186	6.395119863	-3.709768370
	C80	-0.401278013	5.516487973	-4.082289455
	C81	-1.409326574	6.104159036	-5.083305669
	C82	-3.047302215	7.947115234	-4.720222286
	C83	0.111353756	5.947791537	0.755605477
	C84	-0.241934814	7.082097742	1.729051947
	C85	1.016801767	4.952745142	1.510989365
	C86	0.642821898	9.089471117	2.815568271
	C87	0.026003836	10.452703515	2.537621998
	C88	-1.107853948	11.868389075	0.905172190
	C89	-2.477322119	12.365076335	1.424852688
	C90	-1.012257547	12.004327897	-0.617452794
	C91	-2.047673277	11.194642346	-1.389607133
	C92	-4.632584017	11.753579566	2.420117615
	C93	-4.731521719	12.765669886	3.551273086
	C94	-5.351656821	10.434903492	2.826147124
	C95	-5.394542346	9.439494775	1.688264998
	C96	-6.428854848	9.499347259	0.740430230
	C97	-4.385283384	8.480436864	1.504976080
	C98	-6.441921218	8.655084102	-0.370844079
	C99	-4.369752455	7.641465079	0.385908846
	C100	-5.400151019	7.742588886	-0.543996486
	C101	5.544729737	-3.279807761	-8.470313383
	C102	4.056814999	-3.081104766	-8.246690598
	C103	2.373063245	-1.646606316	-7.107780365
	C104	1.959326549	-0.248952038	-7.581415293
	C105	2.315261670	2.162052366	-7.270489292
	C106	3.508098183	3.116497716	-7.263450753
	C107	5.683498851	3.760260931	-6.392850752

C108	6.368869558	4.132173581	-7.692083108
C109	-1.184093158	-2.815493828	-9.713542759
C110	-2.269649070	-1.762682578	-9.465131292
C111	-0.912683134	-3.600510448	-8.409966163
C112	-0.108033787	-4.884978927	-8.683231878
C113	0.359029046	-5.498644535	-7.381553669
C114	-2.815350436	0.510149555	-8.751661496
C115	-2.274255687	1.834595769	-9.252972450
C116	-3.403909955	0.549037680	-7.319712592
C117	-2.323610456	0.514206196	-6.272535029
C118	-1.656084094	1.687900894	-5.883111121
C119	-1.871203691	-0.718761136	-5.771913240
C120	-0.525962464	1.618610138	-5.062143109
C121	-0.748980477	-0.784334837	-4.943085221
C122	-0.055570567	0.382658701	-4.607180735
C123	8.680934035	-5.802484294	-3.881331919
C124	9.704293014	-5.931020699	-2.739806298
C125	7.272447051	-6.113380374	-3.345745093
C126	6.133506806	-5.867070148	-4.282154021
C127	5.976141336	-6.378732327	-5.553797386
C128	4.114026545	-5.345077782	-4.953564329
C129	10.643413432	-7.235915215	-0.930227497
C130	12.076021918	-6.892175994	-1.274177905
C131	0.760700269	-7.274509974	-0.703840202
C132	2.152285079	-7.131795570	-0.097327392
C133	3.296188617	-7.098739442	-1.146260612
C134	3.402589516	-8.483850737	-1.837237280
C135	4.634156897	-8.692389927	-2.719398329
C136	4.771366309	-10.133132022	-3.261681008
C137	4.639664368	-6.813729074	-0.460793323
C138	3.902775917	-0.441739060	-1.680545070
Fe139	4.258893824	-1.789797308	-3.034988161
Fe140	2.409698788	0.946169580	-1.390272096
Fe141	5.222508223	0.594400806	-0.672163138
Fe142	4.494023370	0.716246527	-3.141217846
Fe143	4.239766694	2.906848155	-1.545062238
Fe144	4.564348115	-1.870849005	-0.450862395
Fe145	2.042479745	-2.668218233	-0.947469482
H146	-6.074935700	-7.511549836	-1.143056856
H147	-6.080246392	-6.459185411	-2.390742786
H148	-9.690666853	-4.979669338	-0.577532905
H149	-8.456443870	-6.172372962	-2.252534134
H150	-8.497049470	-7.376080109	-0.970500726
H151	-8.120806595	-2.930392388	0.564713709
H152	-9.166252770	-5.956732255	2.096568809
H153	-9.831071653	-3.248770120	0.918834493
H154	-8.751040670	-6.773181632	4.231010029
H155	-6.503511342	-6.131389198	2.218840558
H156	-8.670767923	-5.104589063	4.840884699
H157	-3.322359446	-2.095407083	2.667369082
H158	-5.427607972	-3.945668202	2.029679699
H159	-4.201528781	-6.493701450	2.241879085
H160	-5.927669108	-0.730955551	6.380229443
H161	-6.466202827	-2.734660163	4.388908766
H162	-4.224320815	-6.707050130	4.011597095
H163	-4.993986664	-1.700478318	2.211357527
H164	-5.372244703	-4.303979262	6.481548788
H165	-7.081486695	-2.085358855	6.526606501
H166	-0.829850121	-3.475751228	3.303982530
H167	-0.868769962	-2.311840326	1.966197663
H168	0.662939858	-2.629769495	2.808966314
H169	-1.820298062	-1.277914006	4.025596932
H170	-4.707496606	-5.544237439	8.321586128
H171	-2.803229218	1.669819940	5.098899225
H172	-2.935708558	-0.935286413	8.782893714
H173	-3.332929082	2.475910173	9.352409751
H174	0.904788817	-0.081558857	3.233663710
H175	-0.402613599	0.796016358	4.068861951
H176	8.470363439	1.769864433	6.004919440
H177	-3.864786551	2.965202562	5.678313961
H178	-4.113778488	3.464096952	8.094766964
H179	-2.098248409	3.151492975	5.798513404
H180	-2.361464967	3.603994574	8.376885013
H181	-3.794081093	1.088216102	7.334313622
H182	-0.800984758	1.733968830	7.434610659
H183	-2.295513648	-0.408939452	6.126889900
H184	-0.646956339	0.185005654	2.418598733
H185	1.445296578	4.240022171	0.791509383
H186	0.370498346	4.397049531	2.206971683
H187	2.174369535	5.186758436	3.085594724

	H188	2.289450849	4.297080430	5.602278832
	H189	4.295041688	4.053765562	2.695529275
	H190	6.232752556	2.512599453	2.538324439
	H191	5.565995110	1.265231491	6.614905876
	H192	-1.738429635	-2.979989879	5.979701185
	H193	-0.676598502	-6.019468620	6.015601126
	H194	-0.351870912	-5.496913160	7.682085003
	H195	-2.830260123	-4.956447101	6.122642665
	H196	-4.435821216	-4.046951894	9.249186805
	H197	3.593186854	2.765275656	6.751022090
	H198	0.910930851	-1.884188587	5.223401116
	H199	-2.705377368	-0.234303826	11.470337967
	H200	-2.749359160	-1.907796443	10.849945666
	H201	-0.071291451	-1.485900027	10.485626864
	H202	10.490432451	-7.153182913	5.254643524
	H203	-0.992444551	-5.685354518	-4.836004348
	H204	1.527937055	-6.328801021	-2.186452622
	H205	-0.751755610	-5.616458441	-9.193190379
	H206	0.751707309	-4.668096255	-9.333340389
	H207	-1.863203760	-3.872714219	-7.926047269
	H208	-1.548801329	1.742842725	-10.095770419
	H209	-1.657049852	-3.510169902	-10.423979562
	H210	-2.861323002	-2.092236812	-2.945660772
	H211	-3.868069737	-4.918767074	-2.405818378
	H212	-0.862783547	-0.385395108	-8.799896494
	H213	-3.659169502	0.294851190	-9.438898994
	H214	0.569109576	-1.694480398	-9.748969008
	H215	-0.992652848	-5.818884699	-3.087187206
	H216	-0.378398411	-2.948588122	-7.700928633
	H217	0.565341418	-2.982812850	-10.775878469
	H218	-6.099967067	-3.204228862	-4.245014876
	H219	-3.311306052	-2.496478878	-1.279069149
	H220	2.127696080	-4.711647406	-7.709457264
	H221	-2.295365746	-4.500740479	-1.757996927
	H222	-4.346400769	2.354236408	2.603832454
	H223	-2.837021301	0.547489161	1.067887307
	H224	-8.633290127	0.432267326	2.460606095
	H225	-7.327079020	-0.180199652	-0.155854635
	H226	-7.319635020	3.863878210	1.524955461
	H227	-9.149981622	8.334893288	3.708795747
	H228	-7.685716381	4.813583500	4.559785445
	H229	-9.492591683	5.131519986	2.440318308
	H230	-6.784981563	6.581710496	2.211473788
	H231	-11.005555617	9.397183132	0.051988702
	H232	-3.528216694	4.146112723	1.046711281
	H233	-4.893393660	3.843603534	-0.037244819
	H234	-10.412609708	10.141805138	2.421793531
	H235	-8.719864796	5.428601849	0.882101785
	H236	-6.370473674	1.899317482	0.495846873
	H237	-7.477634125	-0.882668318	2.236070235
	H238	-9.717898046	1.915651730	-1.678753814
	H239	-1.733979254	1.687203428	-2.872260142
	H240	-10.045453040	0.177905709	-1.817118444
	H241	9.687633134	3.176296644	4.503016599
	H242	8.136636377	3.277536801	3.999001365
	H243	1.654916838	6.115497150	-0.745029083
	H244	-0.834498048	5.439031040	0.521899311
	H245	1.472974288	8.052746199	1.171372193
	H246	-3.281628250	3.550918428	-1.915264443
	H247	-4.709850053	4.380910862	-3.282586237
	H248	-6.015613017	3.698673357	-4.983912441
	H249	-4.552829171	5.523928805	-2.189907083
	H250	-4.810116410	1.589672968	-4.825304049
	H251	-6.599530373	1.549691670	-4.926754334
	H252	0.010802714	11.766293522	-0.940648165
	H253	-1.193581571	13.062822898	-0.862273049
	H254	-0.569439531	10.479035286	-2.658102251
	H255	-2.225224222	9.959925300	-2.976336496
	H256	-2.945452187	9.604076472	-5.921852603
	H257	-1.786616257	5.307391541	-5.742356430
	H258	-7.261999344	8.703419790	-1.091676584
	H259	-3.584991963	8.364171921	2.240928749
	H260	-4.296898146	7.999307634	-3.084794862
	H261	1.565050396	5.730804882	-3.276435374
	H262	2.686912190	7.495951336	-2.289105386
	H263	-0.928711276	5.166360258	-3.181047401
	H264	-7.999238445	-0.106198958	-2.858269718
	H265	4.053810946	10.392713353	-2.738526879
	H266	-5.483278016	-0.692448115	-4.105226027
	H267	1.242432088	6.818144987	-4.626502057

	H268	-3.543057179	6.946305760	0.231658824
	H269	3.469553369	10.537110331	-1.069399915
	H270	-2.125845986	8.214108173	-6.542636115
	H271	-4.580634144	9.225760039	-4.274464173
	H272	-4.458941882	-3.175904484	-4.551051427
	H273	-5.547101169	-3.240866973	-2.039741543
	H274	-7.243157291	10.216499530	0.871184880
	H275	-4.937153074	1.533811909	-2.346904206
	H276	-0.054393942	8.337025688	-3.126547552
	H277	-0.901472460	6.838084595	-5.722335544
	H278	0.020821142	4.617614485	-4.552984194
	H279	-3.085255021	6.149493418	-3.760469117
	H280	0.116227365	8.656106569	3.678406442
	H281	-0.670156289	9.763737789	0.744453354
	H282	-0.397036586	12.588917535	1.338879314
	H283	1.687253467	9.266040335	3.108716887
	H284	-2.942384034	10.495417145	2.055467851
	H285	6.060675248	-2.308454002	-8.461266343
	H286	5.905920888	-3.845399403	-7.586459185
	H287	8.722931942	-4.746176888	-4.186426746
	H288	6.754729379	-4.266493397	-9.781654083
	H289	5.206302195	-4.796322113	-9.786900025
	H290	5.536950299	4.932432062	0.285332485
	H291	5.454039114	7.448775246	1.968565447
	H292	5.180664737	8.448716703	-2.174655602
	H293	4.008816339	5.663633692	0.862891134
	H294	6.656151960	8.184682316	1.140372398
	H295	6.445246433	3.317889220	-5.732903948
	H296	6.153754188	7.027871985	-0.850767330
	H297	4.599785032	-7.350820415	5.275384859
	H298	6.711389725	-8.007466248	3.148979150
	H299	4.658824349	1.934565965	-5.996351090
	H300	4.563243891	-0.304038084	3.999250583
	H301	1.881950136	2.181218725	-8.277436441
	H302	-0.404500180	-1.746821952	-4.557677457
	H303	1.564636382	2.602681320	-6.592560930
	H304	3.620269872	-4.789104986	4.833754013
	H305	3.071620933	0.534083209	-6.063065783
	H306	3.541954092	-5.241826228	3.123174902
	H307	4.337203960	-7.513089112	3.522445084
	H308	6.051191704	-5.633198126	3.179042152
	H309	6.026684649	-5.351446382	4.926432774
	H310	-4.073052463	-0.317203993	-7.213112849
	H311	5.258917852	-9.790937971	5.059759123
	H312	4.593190375	-11.868730974	2.950524888
	H313	0.825556470	0.331333956	-3.966823095
	H314	5.510776200	-12.235311711	5.699046785
	H315	4.024814442	-12.479322699	5.055945412
	H316	5.263397830	-3.465044650	2.704448872
	H317	0.010018964	2.526153395	-4.776895024
	H318	-2.395543898	-1.639329374	-6.037204610
	H319	8.037731548	-9.645570761	-1.125537307
	H320	7.238776313	-7.174273750	-3.045449665
	H321	6.715082149	-8.055625502	0.395638432
	H322	7.415595777	-10.221911887	-2.434597601
	H323	6.241835249	3.401567450	-8.526261053
	H324	6.368172796	-4.979711427	0.448802538
	H325	2.304510801	-7.937197732	0.631799852
	H326	4.474414401	-1.421851295	-7.136813170
	H327	3.409563358	-9.232871761	-1.029148731
	H328	4.252023277	-6.314136579	-6.821547124
	H329	3.093608106	-4.987964670	-5.003005407
	H330	6.114812869	-12.609762440	3.463939830
	H331	5.445126899	-1.172767685	2.778842532
	H332	8.218815320	-5.710407294	1.733787176
	H333	7.116535445	-3.658627597	0.755337973
	H334	8.522383492	-6.933150009	3.709590277
	H335	2.856159275	-3.045811906	5.417032653
	H336	4.584578332	-8.017048004	-3.588065124
	H337	5.549986259	-8.440662022	-2.167781574
	H338	2.490143085	-8.655812878	-2.427358387
	H339	2.320649115	-1.714032157	-6.008968983
	H340	-2.004350815	2.651876094	-6.261863536
	H341	-4.011827543	1.463493606	-7.248746780
	H342	2.151233332	-6.180654216	0.462326864
	H343	8.438913509	-7.220386808	1.429901007
	H344	7.431241439	-9.168416212	1.180748834
	H345	3.305410231	-1.386440373	5.598805624
	H346	6.648675804	-6.965270014	-6.166246870
	H347	7.111608230	-5.540673700	-2.425450631

	H348	1.684954645	-2.372157715	-7.546940132
	H349	5.315989098	4.679723530	-5.910485129
	H350	7.080694721	-0.033473289	5.094474117
	H351	7.371603517	0.521432878	3.430096851
	H352	9.955456659	-6.339644103	-5.393978679
	H353	9.101468456	-7.603561399	-4.801858369
	H354	10.632296124	-8.289427863	-0.608781531
	H355	9.137203770	-7.905701229	-2.280122616
	H356	12.355006204	-7.060931391	-2.342488699
	H357	10.310335709	-6.623590489	-0.077622533
	H358	10.094407097	0.341713156	3.805012564
	H359	-3.835311863	12.824568491	4.212763539
	H360	9.327200638	-7.127739572	6.600622860
	H361	8.738023259	-4.645778025	5.494339989
	H362	-5.141134288	12.184576126	1.541913642
	H363	-4.843137078	10.001798687	3.701258706
	H364	-6.372226580	10.701727570	3.135645880
	H365	4.017757226	-1.214953711	0.973680314
	H366	-6.239557682	6.920969494	-2.097658314
	H367	1.389945455	-1.540316972	0.087810694
	H368	3.390619447	-1.084654308	0.456311828
	H369	1.703465082	-2.205180466	0.592681397
	H370	-1.343898444	0.022801082	-0.994679495
	Mo371	4.073127509	-4.263284125	-1.922115924
	N372	-6.644816422	-7.171903570	-1.922223965
	N373	-8.853623726	-4.824456504	-0.021643949
	N374	-8.713309164	-5.317166159	2.745558660
	N375	-6.053864884	-6.148919199	3.135765390
	N376	-4.692876190	-3.696328265	2.689126132
	N377	-5.749931350	-2.252824442	4.938528922
	N378	-4.900803803	-3.785861835	7.228349901
	N379	-2.218451758	-4.939649012	6.944587511
	N380	-0.726908192	-2.905670277	5.890565482
	N381	-1.479135935	-0.062645101	6.637940203
	N382	-2.244466348	-0.509461268	9.405584080
	N383	4.829357846	-11.850966878	5.041024576
	N384	5.860424427	-9.504263030	4.280319122
	N385	4.626865922	-3.494796010	3.506200643
	N386	3.492307294	-2.283879277	5.161501190
	N387	4.895308547	-1.201715864	3.657321625
	N388	8.440700225	-7.139271728	4.729746332
	N389	-5.240159527	-2.675075527	-4.091323610
	N390	-1.397992223	-5.414925647	-3.932640038
	N391	-5.621855062	-0.258582589	-3.176342351
	N392	-8.088451436	0.715192886	-2.264105956
	N393	-8.031396429	0.177089524	0.498235554
	N394	-6.030459920	1.889436277	1.461864271
	N395	-2.987243202	2.684143663	-1.404807055
	N396	-2.073437845	0.777900348	-0.941360151
	N397	-6.809331622	4.468929845	2.163604129
	N398	-10.021270923	7.554743747	0.590156732
	N399	-10.182748533	9.229999202	2.035284690
	N400	8.933116743	2.638091145	4.070066924
	N401	6.141091867	7.303163530	1.221567450
	N402	4.449445873	8.740972281	-1.531779876
	N403	1.912763535	8.144986007	-2.467483013
	N404	-2.560627927	6.740186370	-4.432624211
	N405	-2.589130359	8.666929120	-5.762389437
	N406	-3.954546823	8.519698191	-3.896510164
	N407	0.704555735	6.427872861	-0.495341739
	N408	0.607756779	8.148512920	1.710394178
	N409	-0.699917599	10.558223712	1.383421245
	N410	-1.580559438	10.605330536	-2.523436802
	N411	-3.241342105	11.466937287	2.095034224
	N412	5.782615532	-3.950162945	-9.752551576
	N413	3.720701106	-1.971904959	-7.557292089
	N414	2.560954768	0.772419963	-6.924442212
	N415	4.624460307	2.779114583	-6.568522785
	N416	-0.018682828	-2.236429931	-10.393139247
	N417	-1.853989297	-0.549497837	-8.998207747
	N418	9.026834151	-6.614992113	-5.061513422
	N419	4.953476800	-5.212589895	-3.923409393
	N420	4.705040299	-6.034646211	-5.954886327
	N421	9.760602255	-7.117867296	-2.076658988
	N422	0.196012835	2.794762966	-0.662060586
	N423	-0.345401824	-4.298654934	-0.491266797
	N424	1.034947283	2.098031991	-0.952937191
	N425	0.577104352	-3.710141075	-0.751663765
	O426	-6.638169426	-5.273441223	0.241747391
	O427	-7.887225429	-3.213497684	3.060686978

O428	-6.286856562	-5.755071120	5.375439043
O429	-3.150303661	-4.428522947	4.194638291
O430	-3.805226536	-1.077716558	4.921552815
O431	-4.680611617	-1.773132360	8.296558939
O432	-2.046688562	-4.737203596	9.223000843
O433	1.235111675	-4.101559877	6.052918284
O434	0.795878642	-0.090910353	6.863975354
O435	-0.800997728	1.184994848	9.850430207
O436	-0.481430795	-1.153651074	12.458109068
O437	6.848349261	-10.380619567	2.383367958
O438	7.186708393	-8.089406359	6.407656288
O439	10.694572916	-4.671503309	6.067125238
O440	-5.512088665	-0.840237922	-0.959961571
O441	-2.862027953	-4.005426302	-4.954916081
O442	-7.008610826	2.512154065	-1.363191041
O443	-5.801848298	3.302504038	-4.120247959
O444	-10.034775074	1.266087695	0.761980780
O445	-6.258777531	1.053020909	3.576472306
O446	-4.890996921	4.746378610	3.370463350
O447	-7.455208190	6.834321038	4.728299563
O448	9.993687403	-0.281314780	5.746152041
O449	2.747477832	4.361159517	4.743921526
O450	3.504167030	8.452280582	0.522753080
O451	1.332587475	10.369082534	-2.553245636
O452	-1.282909136	7.301218854	-1.210107848
O453	-1.196881984	6.972202955	2.508737548
O454	2.061287732	5.625904919	2.217952339
O455	0.178653038	11.389573287	3.330943191
O456	-2.811259913	13.548518587	1.269434810
O457	-3.234189395	11.132559798	-1.037196782
O458	-5.728229110	13.440759231	3.755595926
O459	-5.350145812	6.927320703	-1.693686408
O460	3.227579595	-3.930200843	-8.654559933
O461	1.157432078	-0.087593581	-8.522637596
O462	3.407988170	4.204553492	-7.853435992
O463	7.053490786	5.135368886	-7.818401913
O464	-3.470243829	-2.015296181	-9.659259935
O465	-0.373861706	-6.152898685	-6.646562902
O466	1.631012423	-5.247621738	-7.015049872
O467	-2.619703921	2.929138829	-8.831011018
O468	10.473774938	-4.997256805	-2.440118552
O469	12.903055748	-6.530219939	-0.448897908
O470	0.602425348	-6.734611274	-1.927813427
O471	-0.176567431	-7.800969456	-0.114397812
O472	5.941904388	-10.653190044	-3.242778801
O473	3.729288523	-10.686436851	-3.711470777
O474	5.307610616	-5.786312250	-0.911306183
O475	5.061189179	-7.561031944	0.441825588
O476	2.992721267	-6.047576054	-2.066521609
O477	8.782091102	-6.476984997	2.001328133
O478	6.849439546	-4.501905537	1.169914832
O479	-4.042203980	4.923111721	-2.784252778
O480	8.297085622	-10.061417432	-1.977588059
O481	7.614119963	-8.469840615	0.502940874
S482	3.912511825	5.182025494	-1.525283884
S483	2.829389143	2.224281672	-3.226581306
S484	2.420054469	-3.114032903	-3.158135636
S485	3.655063777	1.888145184	0.340933466
S486	0.819984499	-0.696581923	-1.518502618
S487	4.858211657	-0.637381999	-4.809909513
S488	3.547394139	-3.753897127	0.289704724
S489	6.190122918	1.876512403	-2.185948873
S490	5.957595999	-2.819775320	-1.988428735
S491	6.288111078	-0.832876081	0.633983569
end			
